# Insights into archaeal evolution and symbiosis from the genomes of a nanoarchaeon and its inferred crenarchaeal host from Obsidian Pool, Yellowstone National Park

**DOI:** 10.1186/1745-6150-8-9

**Published:** 2013-04-22

**Authors:** Mircea Podar, Kira S Makarova, David E Graham, Yuri I Wolf, Eugene V Koonin, Anna-Louise Reysenbach

**Affiliations:** 1 Biosciences Division, Oak Ridge National Laboratory, Oak Ridge, TN, 37830, USA; 2 Department of Microbiology, University of Tennessee, Knoxville, TN, 37996, USA; 3 National Center for Biotechnology Information, National Library of Medicine, National Institutes of Health, Bethesda, MD, 20894, USA; 4 Department of Biology, Portland State University, Portland, OR, 97207, USA

**Keywords:** Archaea evolution, Single cell genomics, Symbiosis, Hyperthermophiles, Split genes

## Abstract

**Background:**

A single cultured marine organism, *Nanoarchaeum equitans*, represents the *Nanoarchaeota* branch of symbiotic Archaea, with a highly reduced genome and unusual features such as multiple split genes.

**Results:**

The first terrestrial hyperthermophilic member of the *Nanoarchaeota* was collected from Obsidian Pool, a thermal feature in Yellowstone National Park, separated by single cell isolation, and sequenced together with its putative host, a *Sulfolobales* archaeon. Both the new *Nanoarchaeota* (Nst1) and *N. equitans* lack most biosynthetic capabilities, and phylogenetic analysis of ribosomal RNA and protein sequences indicates that the two form a deep-branching archaeal lineage. However, the Nst1 genome is more than 20% larger, and encodes a complete gluconeogenesis pathway as well as the full complement of archaeal flagellum proteins. With a larger genome, a smaller repertoire of split protein encoding genes and no split non-contiguous tRNAs, Nst1 appears to have experienced less severe genome reduction than *N. equitans.* These findings imply that, rather than representing ancestral characters, the extremely compact genomes and multiple split genes of *Nanoarchaeota* are derived characters associated with their symbiotic or parasitic lifestyle. The inferred host of Nst1 is potentially autotrophic, with a streamlined genome and simplified central and energetic metabolism as compared to other *Sulfolobales*.

**Conclusions:**

Comparison of the *N. equitans* and Nst1 genomes suggests that the marine and terrestrial lineages of *Nanoarchaeota* share a common ancestor that was already a symbiont of another archaeon. The two distinct *Nanoarchaeota*-host genomic data sets offer novel insights into the evolution of archaeal symbiosis and parasitism, enabling further studies of the cellular and molecular mechanisms of these relationships.

**Reviewers:**

This article was reviewed by Patrick Forterre, Bettina Siebers (nominated by Michael Galperin) and Purification Lopez-Garcia

## Background

A decade after their discovery, the *Nanoarchaeota* are still represented by a single cultured organism, *Nanoarchaeum equitans*[1]. An obligate extracellular symbiont (or possibly an ectoparasite) of the marine hyperthermophilic crenarchaeon *Ignicoccus hospitalis*[2], *N. equitans* provides unique opportunities to study molecular, cellular and evolutionary mechanisms of specific associations between Archaea. With its highly reduced genome, devoid of virtually any primary biosynthetic functions and resembling bacteria that are obligate symbionts and parasites, *N. equitans* must acquire metabolic precursors from its host through yet unknown mechanisms.

The phylogenetic placement of *N. equitans* among the Archaea has been controversial. The multiple, fragmented protein-coding regions and especially the presence of non-contiguous split tRNA genes [3] have been interpreted as evidence that *N.* equitans represents an ancient phylum-level lineage that maintained some ancestral gene structures and features of genome organization [4,5]. Challenging this view, phylogenetic reconstruction using concatenated protein sequences and analysis of the distribution of gene families among the major archaeal lineages, pointed to a potentially close evolutionary relationship between *N. equitans* and the *Thermococcales*, a basal order of the *Euryarchaeota*[6]. Distinguishing between these contrasting hypotheses and teasing apart genomic idiosyncrasies caused by rapid evolution from ancient characters has been hampered by the absence of genomic data from additional members of the *Nanoarchaeota*.

Using primers designed from the small subunit rRNA sequence of *N. equitans*, amplification of SSU rRNA genes from environmental samples resulted in identification of additional lineages of the *Nanoarchaeota* in a wide range of high temperature environments. These novel sequences were not only from deep-sea hydrothermal vents (East Pacific Rise), but also from continental samples collected in Yellowstone National Park, USA (Obsidian Pool) and Kamchatka, Russia (Uzon Caldera) [7]. Notably, the non-marine SSU rRNA sequences were substantially divergent from that of *N. equitans* (83% identity) indicating that the *Nanoarchaeota* is a distinct, diverse taxon, with an as yet unclear position within the Archaea. The diversity of the Nanoarchaeota was subsequently expanded by the discovery of additional uncultured lineages in samples collected from thermal sites in central Asia, New Zealand and Chile as well as several distinct phylotypes from mesophilic high salinity environments from South Africa and Mongolia [8]. Moreover, recent pyrosequencing studies have shown that species related to *N. equitans* are present at many deep-sea hydrothermal vent sites, from the Mid Atlantic Ridge to the southwestern Pacific Eastern Lau Spreading Center and can reach a significant fraction of the archaeal population [9,10]. *Ignicoccus* was also present in many of these marine samples and a direct association with *Nanoarchaeota* was detected in actively forming chimneys on East Pacific Rise [11], suggesting that different marine *Nanoarchaeum* species might colonize specific *Ignicoccus* hosts. Indeed, in the laboratory, *N. equitans* is only able to grow in co-culture with *I. hospitalis*. Both were isolated from a hydrothermal site north of Iceland but *N. equitans* failed to grow with related species (*I. islandicus* and *I. pacificus*) or other Archaea [12]. So far, no *Ignicoccus* has been isolated from or detected in terrestrial samples, suggesting that the *Nanoarchaeota* from continental sites either depend on other hosts or live independently. Characterization of additional *Nanoarchaeota* is essential for a better understanding of the evolutionary history and biology of this remarkable group of *Archaea*.

Here we describe the near-complete genome of a thermophilic member of the *Nanoarchaeota* from Obsidian Pool (Yellowstone National Park) together with the nearly complete genome of its likely host that represents a distinct group within the *Sulfolobales* (*Crenarchaeota*). Although these organisms have not yet been isolated in pure culture, the genomic data from a continental member of the *Nanoarchaeota* helps to distinguish between alternative evolutionary scenarios proposed for these *Archaea*.

## Results

### Genomes of a nanoarchaeon and its apparent host from Obsidian Pool

**Figure 1:**
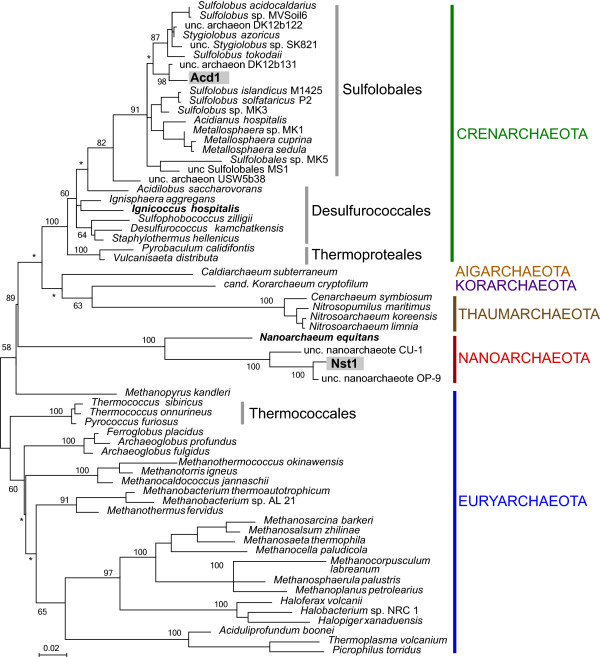
**Maximum likelihood of the*Archaea*based on SSU rRNA (872 sites) and the relationship of Nst1 and Acd1 with representative cultured and uncultured Archaea.** The branch numbers indicate bootstrap support, shown only for major clades (* is <50%).

A fresh microbial community sample collected from Obsidian Pool was labeled with a fluorescent polyclonal antibody developed against *N. equitans*. Using flow cytometry, single cell-size fluorescent particles were individually isolated and used for genomic amplification (MDA) with phi29 DNA polymerase. Five genomic products tested positive when subjected to SSU rRNA gene amplification using *Nanoarchaeota*-specific PCR primers but also yielded PCR products with a universal archaeal primers set that excludes *Nanoarchaeota*. Direct sequencing of the *Nanoarchaeota* amplicons assigned all five genomes to a unique organism, with SSU rRNA sequences 98% identical to the previously described clone OP9 [7] and 81% identical to *N. equitans* (Figure 1). The archaeal amplicon sequences also identified a member of the *Sulfolobales* in all five MDA products, most closely related (96-97% identity) to uncultured organisms from other thermal acidic environments (Figure 1).

**Figure 2:**
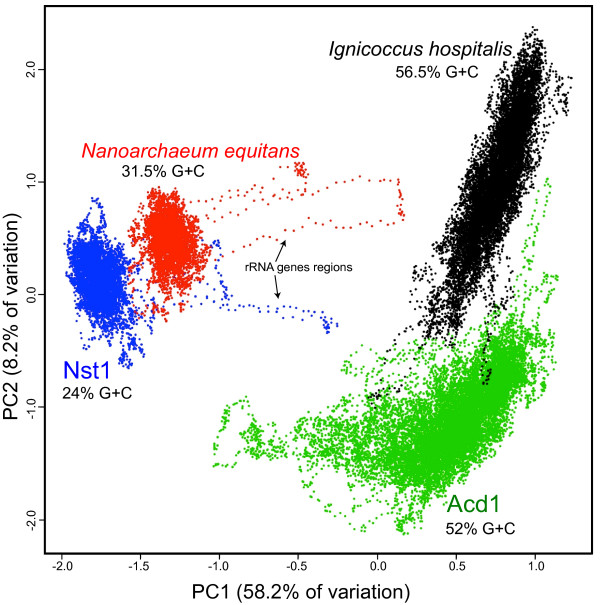
**Composition analysis (sliding window of kmer tetranucleotide frequencies) of the Nst1 and Acd1 genomes, compared to those of*N.equitans and I.hospitalis*.**

The amplified genomic DNAs were sequenced using Illumina HiSeq, yielding ~10 Gbp each. The sequence was digitally normalized and assembled *de novo*. Kmer tetranucleotide frequency analysis of the resulting 109–158 contigs from each dataset (0.9-1.2 Mbp of assembled sequences) partitioned the sequences in two distinct populations, with average G+C contents of 52% and 24%, respectively (Additional file 1). Combined secondary assembly and curation of the contigs corresponding to each of the two populations resulted in an Obsidian Pool nanoarchaeal draft genome (*Nanoarchaeota* Nst1, referred herein as “Nst1”) consisting of 7 contigs (totaling 0.593 Mbp, 24% G+C content) and the tentatively assigned host (*Sulfolobales* Acd1, referred herein as “Acd1”) consisting of 8 contigs (totaling 1.51 Mbp, 52% G+C content) (Figure 2). Gene prediction resulted in identification and annotation of 656 protein encoding ORFs in Nst1 and 1692 in Acd1 as well as that of all rRNA genes and nearly all essential tRNAs. The recently updated database of archaeal clusters of orthologous genes (arCOGs) [13,14] was also used as a framework for annotation and genomic comparisons (Additional file 2). In Nst1, 72% (473 out of 656) of the predicted proteins were assigned to at least one arCOG. This is the lowest coverage among all Archaea including *N. equitans* and *Cenarchaeum symbiosum* which both have ~74% arCOG coverage. In Acd1, arCOGs were identified for 1567 out of the 1692 ORFs (~92%).

The level of completeness of the two genomes was estimated from the fraction of universally conserved genes that are represented in the given sample [15]. Because *Nanoarchaeota* form a deep archaeal branch, the relevant set of conserved genes for Nst1 genome reconstruction includes the 138 arCOGs that are represented in all *Archaea*. The Nst1 genome contains members of 126 of those arCOGs, resulting in an estimate of at least 91% genomic completeness, with 692 to 761 genes for the full gene complement (at 95% confidence). For Acd1, which is unequivocally classified within *Crenarchaeota*, the relevant conserved gene set includes 352 ubiquitous arCOGs. The Acd1 gene set includes 349 of these, an assembly that is estimated to be 99% complete, with the 95% confidence interval of 1696 to 1730 genes.

### An updated phylogeny of the *Nanoarchaeota*

**Figure 3:**
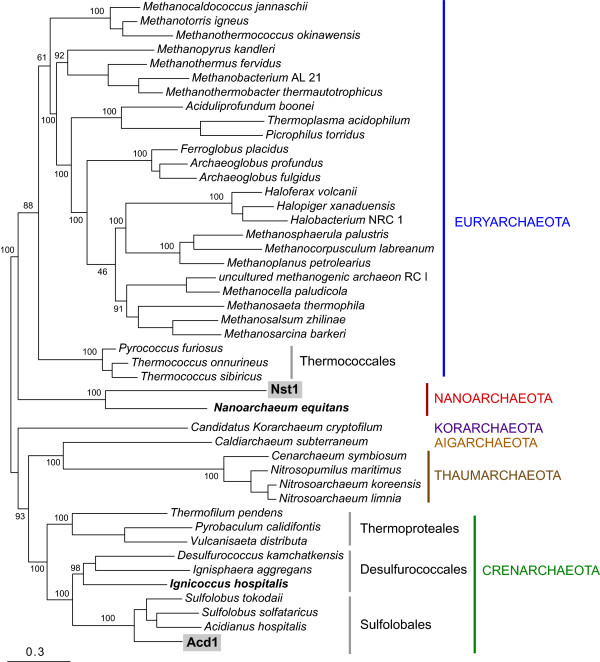
**Maximum likelihood phylogeny of the*Archaea*based on concatenated ribosomal protein gene sequences (8077 sites) from representative complete genomes.**

The ribosomal protein sequences from Nst1 and Acd1 were added to concatenated alignments of 56 ribosomal proteins that are universally conserved in the complete archaeal genomes [16]. All of these proteins were identified in the Acd1 genome whereas 4 were missing from the Nst1 draft genome (L15E, S8E, S11 and S27E). Maximum likelihood tree topologies were identical between reconstructions using the entire dataset (122 genomes) and a representative group of 45 archaeal genomes, recovering the same phylogenetic relationships between the major archaeal taxa (Figure 3). Both reconstructions showed Nst1 to be a sister taxon of *N. equitans*, most likely representing a distinct family of *Nanoarchaeota*. The two members of *Nanoarchaeota* comprised a distinct, deep branching taxon that was not closely related to any other archaeal clades. Both trees agreed on the position of Acd1 as a close outgroup to the *Sulfolobales*, conceivably representing a previously unknown family within this order of the *Crenarchaeota*. A separate phylogeny based on four concatenated RNA polymerase subunits supports these conclusions (Additional file 3).

### Inferred physiological features of Nst1 and evolutionary genomics of the *Nanoarchaeota*

**Figure 4:**
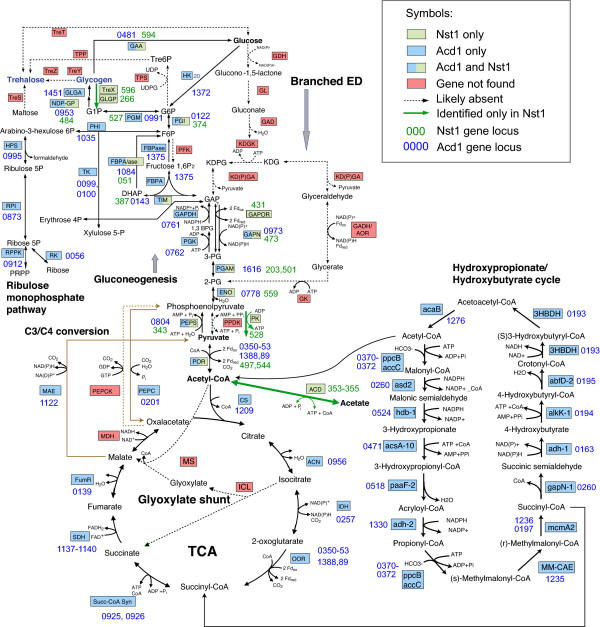
**Reconstruction of the central carbon metabolism of Acd1 and Nst1 in comparison with pathways of thermoacidophilic Archaea (based on Zaparty and Siebers, 2011)[**[32]**].**

Nst1 has a substantially larger genome than *N. equitans* (~592 vs. 491 kb). Nevertheless, functional gene annotation and metabolic reconstruction indicate that neither *Nanoarchaeota* can be physiologically autonomous and both depend on the association with another organism, *I. hospitalis* for *N. equitans* and most likely the Acd1 for Nst1. In particular, similar to *N. equitans*, the reduced genome of Nst1 does not encode functional pathways for *de novo* biosynthesis of lipids, amino acids, coenzymes or nucleotides. However, several metabolic functions that are missing in *N. equitans* are predicted for Nst1. Most notably, the Nst1 genome encodes a complete gluconeogenic pathway, as well as glycosyltransferase enzymes that are likely to be involved in polysaccharide production (Figure 4). There is no evidence for a modified Entner-Doudoroff pathway in this organism, therefore carbohydrate metabolism apparently proceeds through the typical Embden-Meyerhof-Parnas pathway. Three intermediates in this pathway (glucose, fructose-6-phosphate, and pyruvate) require separate enzymes for phosphorylation or dephosphorylation reactions that determine whether carbon flow is glycolytic or gluconeogenic. No glucokinase or glucose-6-phosphatase was identified, suggesting that only activated sugars or polysaccharides enter or leave the Nst1 cell. No member of the three classes of glycolytic phosphofructokinases was identified. Instead, Nst1 encodes a gluconeogenic class V bifunctional fructose-1,6-bisphosphate aldolase/phosphatase (Say, 2010) that cannot catalyze the glycolytic reaction. Furthermore, Nst1 encodes both a glycolytic non-phosphorylating NADP-dependent glyceraldehyde-3-phosphate dehydrogenase and a glyceraldehyde-3-phosphate ferredoxin oxidoreductase (van der Oost, 1998). Finally, Nst1 encodes both glycolytic pyruvate kinase and gluconeogenic phosphoenolpyruvate synthase. Under anoxic conditions, the Nst1 cells could accumulate sufficient pools of reduced, low-potential ferredoxin to support the carboxylation of acetyl-CoA, produced from acetate by an ADP-forming acetyl-CoA synthetase, through the activity of a two-subunit pyruvate ferredoxin oxidoreductase. Therefore triose phosphates could be oxidized using glycolytic enzymes at high reduction potentials to produce limited ATP through pyruvate-kinase mediated phosphorylation; alternatively, at low reduction potentials, triose phosphates could be utilized to produce sugars from exogenous acetate using gluconeogenic enzymes.

The presence of genes for phosphoglucomutase, glucose-1-phosphate thymidylyltransferase, nucleotide sugar reductase and epimerase, and an oligosaccharide transferase suggest that Nst1 cells produce diverse nucleotide-sugars that are likely to be involved in post-translational modification of proteins. A complete set of genes for glycogen synthesis and breakdown was also found (Figure 4). Such rich carbohydrate chemistry is unexpected in this inferred obligate symbiont/parasite, and further studies will be required to determine the specificity and products of these diverse enzymes.

Amino acid activation in the *Nanoarchaeota* shows several cases of non-orthologous gene displacement. Most of the *Archaea* use a non-discriminating glutamyl-tRNA synthetase to form Glu-tRNA^Gln^, which is transamidated to Gln-tRNA^Gln^ by the GatDE protein complex. This was experimentally confirmed in *N. equitans*[17] and the required genes are also present in Nst1. In addition, *N. equitans* encodes an analogous enzymatic machinery for Asp-tRNA^Asn^ transamidation. By contrast, Nst1 apparently uses an asparaginyl tRNA synthetase to form Asn-tRNA^Asn^ directly. Nst1 and *N. equitans* encode unrelated lysyl-tRNA synthetases of class II and class I, respectively. In this case, *N. equitans* probably retained the ancestral class I LysRS that is found in most archaea. The source of the class II enzyme in Nst1 was probably a member of the *Crenarchaeota*, although not Acd1 (Additional file 4). For amino acid metabolism, Nst1 encodes a *Thermococcus*-like asparagine synthetase and a putative glutamine amidotransferase but lacks the glutamate dehydrogenase found in *N. equitans*. All other amino acids apparently have to be acquired from the environment or the host cell.

Nucleotide biotransformation capabilities also substantially differ between the two *Nanoarchaeota*. Specifically, *N.* equitans encodes a flavin-dependent thymidylate synthase (ThyX), and an anaerobic ribonucleoside-triphosphate reductase. In contrast, *Nst1* encodes the non-homologous, folate-dependent thymidylate synthase (ThyA) and an adenosylcobalamin-dependent ribonucleotide reductase. Notably, the ribonucloetide reductase gene encompasses an intein in the same position as one of the two inteins in the homologouos gene of *Pyrococcus furiosus.* Both *Nanoarchaeota* encode an adenylate kinase (arCOG01039)(Nst432 and Neq139) although the *N. equitans* sequence is highly divergent.

**Figure 5:**
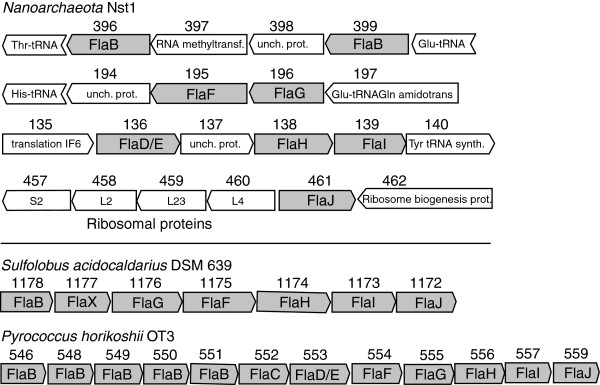
**Organization of archaeal flagellum (archaellum) genes in Nst1 and comparison to operons from*Crenarchaeota*(*S.a.*) and*Euryarchaeota*(*P.h.*).** The archaella genes are in shaded rectangles. The numbers represent the gene loci in the genomes. Several paralogs of FlaI (Nst1_088, 227 and 260) and one FlaG paralog (Nst1_560) are not shown.

A key cellular structure that, judging from the genome sequence, is present in Nst1 but not in *N. equitans* is the archaeal flagellum (archaellum) [18]. Genes encoding all the essential subunits of the archaellum have been identified (Figure 5), including two archaeal flagellins and the gene responsible for flagellar assembly, function and regulation (*flaD/E,F,G,H,I,J, FleN*). The presence of the flaD/E gene, so far only identified in *Euryarchaeota,* and the topology of the phylogenetic tree of the regulatory subunit *flaH* (Additional file 5) are compatible with a euryarchaeal type-archaellum in Nst1.

The overall gene content of *Nanoarchaeota* further supports its affinity with Euryarchaeota. Among the 283 arCOGs that are represented in both *N. equitans* and Nst1*,* 17 arCOGs are missing in *Crenarchaeota* but present in the majority of the *Euryarchaeota*. This includes genes encoding proteins involved in key cellular functions such cell division (FtsZ, SepF) as well as nucleic acids and protein processing (e.g. DNA polymerase II subunits, ERCC4-like helicase, ribosomal protein L41E, pre-protein translocase subunits SecF and SecD)(Additional file 6). This observation complements the results of phylogenetic analyses and is best compatible with a common ancestry of *Nanoarchaeota* and *Euryarchaeota*. Of the 321 *Nst1* genes with orthologs in *N. equitans*, only 15 have no confidently identifiable homologs in other sequenced archaeal genomes and hence represent a putative genomic signature of the *Nanoarchaeota*. Only one of these genes, encoding a homolog of a bacterial 16S rRNA methyltransferase [19], has a defined function. The remaining *Nanoarchaeota*-specific proteins are presently uncharacterized. Of the 183 predicted protein-coding genes of *Nst1* that were not assigned to arCOGs, 27 had homologs in *N. equitans*, and another 26 showed statistically significant similarity to proteins from other *Archaea* that have not yet been classified into families. The inferred common gain in *Nanoarchaeota* includes only 8 genes, in particular two components of the Type II/IV secretory pathway and the tRNA (uracil-54, C5)-methyltransferase both of which are otherwise present only in *Euryarchaeaota*.

**Figure 6:**
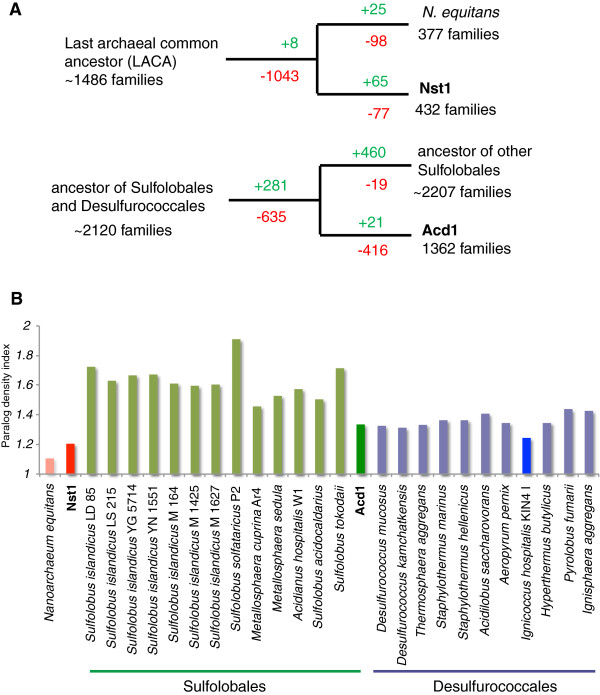
**Evolution of gene families in*Nanoarchaeota*and*Sulfolobales.*** (**A**) Predicted pattern of arCOG gene families gains (green) and losses (red) in the *Nanoarchaeota* and the *Sulfolobales* lineages. (**B**) Paralog density in the *Crenarchaeota* and *Nanoarchaeota* genomes.

Maximum parsimony reconstruction of gene loss and gene gain events suggests extensive loss of genes from all functional categories in the *Nanoarchaeota* branch. Both genomes lack 46 of the 218 arCOGs from the archaeal core gene set (Figure 6 and Additional file 7). The Nst1 genome shows the second lowest paralog density among the *Archaea*, surpassed only by *N. equitans* (Figure 6). The majority of the functionally characterized genes inferred to have been lost is related to central metabolism or encode apparently dispensable functions in the informational systems that are also missing in some other *Archaea*. Although Nst1 appears to retain substantial carbon metabolism functions, it lacks 16 of the core archaeal genes present in *N. equitans*, including radical SAM and pyruvate-formate lyase-activating enzymes as well as the archaeal V-type ATPase. Although at present we cannot rule out that some of these genes are contained in the missing genomic regions, the lack of any of the multiple membrane ATP synthase subunits is intriguing, possibly indicating that Nst1 has lost this otherwise ubiquitous enzyme complex. Both *Nanoarchaeota* apparently lack a functional membrane respiratory complex but Nst1 encodes several proteins implicated in scavenging of oxidative molecules, including cytochrome d ubiquinol oxidase subunit 1, superoxide reductase, and both subunits of alkyl hydroperoxide reductase. Finally, Nst1 lacks the CRISPR-Cas type I-B system that is active in *N. equitans*[20]. The absence of this system is extremely unusual in a hyperthermophile [21] and is unlikely to be caused by low sequence coverage of the respective genomic loci because CRISPR systems consist of many protein-coding genes and DNA elements [21].

**Figure 7:**
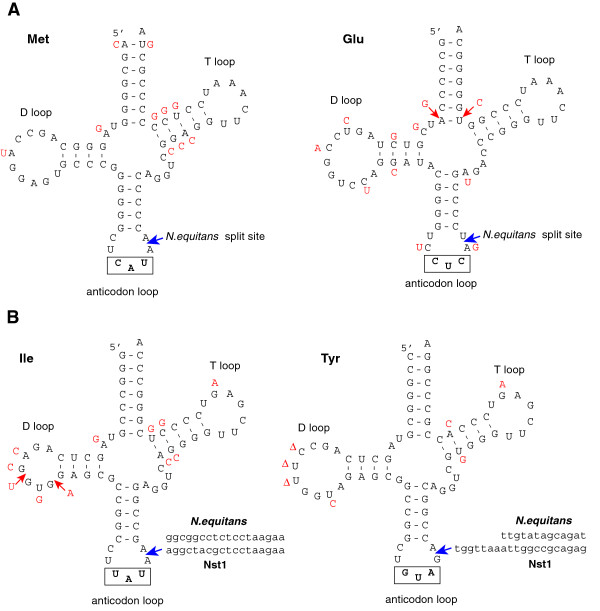
**Comparison between tRNAs of*N. equitans*and Nst1.** (**A**) Secondary structure models of Nst1 single transcript tRNAs that are transcribed from split genes in *N. equitans*. In red are the nucleotide differences present in *N. equitans*. (**B**) Secondary structures of tRNAs that are interrupted by an intron in both *Nanoarchaeota*, using the Nst1 sequence frames. The intron sequences and their insertion site (blue arrow) are indicated as well as the nucleotide polymorphisms in *N. equitans* (Insertions-red arrows, deletions-Δ).

As expected, the repertoire of informational genes shows a much stronger conservation between the two *Nanoarchaeota* than the set of genes for metabolic enzymes. Nevertheless, several notable differences were observed. Analysis of the Nst1 genome identified 41 tRNAs; the tRNA^Phe^ is currently missing, probably due to the incompleteness of the genome sequence. Among the detected tRNAs, only two contain introns, and the positions of both introns are shared between the two *Nanoarchaeota* (tRNA^Ile^ and tRNA^Tyr^). The high sequence identity extends also to the intronic region although there are some differences between the D loop structures of the two species (Figure 7). The two *Nanoarchaeota* share more than 20 typical archaeal genes encoding RNA-modifying enzymes as well as putative snoRNAs that target RNA modifications. The catalytic subunit of the tRNA splicing endonuclease was readily identifiable and shared 45% sequence identity with the *N. equitans* ortholog (Neq205). It is unclear whether the Nst1 enzyme functions as a heterotetramer similar to the counterpart in *N. equitans*[22,23] because we did not identify a structural subunit beta gene. Unlike *N. equitans*, Nst1 also encodes a ribonuclease P complex that otherwise is ubiquitous among cellular life forms. We identified three genes encoding protein subunits and the RNA component indicating that the tRNA maturation pathway in this organism is similar to that in other *Archaea* and not drastically changed as it is in *N. equitans*[24]. Phylogenetic analysis of two of the subunits (p21 and p29) revealed affinities to the euryarchaeal enzyme whereas p30 appeared divergent. The RNA component of the RNAse P is considerably shorter than other archaeal counterparts, including type T variants from *Thermoproteales*[25] and could not be folded into a typical secondary structure for this molecule. Thus, the RNAse P complex of Nst1 appears to have undergone partial degradation compared to complete loss in *N. equitans.* Another interesting difference between the two genomes involves a component of the replication machinery. Recently is has been shown that all *Archaea* (with the single exception of *Caldivirga maquilingensis*) encode RecJ-like proteins with a DHH hydrolase domain that is predicted to be either active or inactivated. Analogous to eukaryotic CDC45, the RecJ homologs are predicted subunits of the CMG (CDC45/RecJ, MCM, GINS) complex involved in replication initiation [26]. Among the two Nanoarchaeota, *Nst1* encodes a putative ancestral form of RecJ with active DHH domain whereas *N. equitans* apparently acquired a highly diverged RecJ homolog in which the DHH domain is severely disrupted [26].

The most parsimonious explanation for the extensive common gene loss is the early evolution of host dependency (possibly parasitism) in *Nanoarchaeota* (prior to the radiation of *N. equitans* and Nst1 from their common ancestor) followed by differential loss after the lineage split, although presently it is impossible to strictly rule out massive parallel gene loss. Supporting the former scenario is another distinctive feature of the *Nanoarchaeota*, the large number and type of split protein genes (proteins that are encoded by one gene in most, if not all organisms, but are separated in two genes in *Nanoarchaeota*, requiring post-translational assembly). The genome of *N. equitans* harbors 10 split protein-coding genes, the largest number among the *Archaea*[5]. In Nst1 we identified 8 such genes, with 6 being split in both genomes (Table 1). Among these, three are split in the same position and are intact in all other archaeal genomes, suggesting gene splitting events that occurred after the separation of the *Nanoarchaeota* from the rest of the *Archaea* but predates the radiation of the *N. equitans* and Nst1 lineages. For two other genes, those encoding archaeosine tRNA-guanine transglycosylase and RNA polymerase subunit B, the split is likely to be ancestral because the split site is shared by many diverse archaea. Each of the two nanoarchaeal genomes also encompasses unique split protein genes, indicating that, along with gene loss, the process of gene splitting continued in each of the two lineages after their divergence.

**Table 1:** **Notable genomic differences between*N. equitans*and Nst1**

**Features**	**N. equitans**	**Nst1**	**Note**
**Split Proteins**			
Reverse gyrase	NEQ 318-434	Nst 337-402	Same site
Glu-tRNA^Gln^ amidotransferase	NEQ 245-396	Nst 197-449	Same site
Predicted RNA-binding protein	NEQ 438-506	Nst 176-251	Same site
Archaeosine tRNA-guanine transglycosylase	NEQ 124-305	Nst 096-232	Same site
RNA polymerase subunit B	NEQ 156-173	Nst 632-633	Same site
Large helicase-related protein	NEQ 003-409	Nst 172-239	Different site
DNA polymerase I	NEQ 068-528	Nst 417	Not split in Nst1
Topoisomerase I	NEQ 045-324	Nst 174	Not split in Nst1
P-loop ATPase-acetyltransferase fusion protein	NEQ 096-495	Nst 401	Not split in Nst1
Alanyl-tRNA synthetase	NEQ 211-547	Nst 054	Not split in Nst1
Diphthamide synthase sub. DPH2	-	Nst 222-440	Absent in *N.eq.*
Uncharacterized conserved protein (arCOG04253)	-	Nst 474-480	Absent in *N.eq.*
**tRNAs**			
*cis*-spliced tRNAs	Ile, Met, Trp, Tyr	Ile, Tyr	
*trans*-joined tRNAs	iMet, His, Lys, Gln, Glu (2)	none	
RNase P	Absent	Present	
Gluconeogenesis-Glycolysis	Absent	Present	
Polyamine biosynthesis	Absent	Present	
ATP synthase	Present	Absent	
Glutamate dehydrogenase	Present	Absent	

* Split in other archaea also.

In addition to the split protein-coding genes, 6 tRNAs of *N. equitans* are synthesized as halves from separated genes and subsequently trans spliced [3,27] (Table 1). The orthologs of these tRNAs in Nst1 are represented by regular, full-length genes that share ~85% nucleotide identity between the two organisms (Figure 7). We did not identify any split tRNAs genes in the Nst1 genome.

Taken together, the results of genome analysis indicate a unique propensity for gene splitting in the *Nanoarchaeota* that is most dramatically manifested in the *N. equitans* lineage. Along with the disintegration of the conserved operon structure (such as the ribosomal operons), the absence of any substantial synteny between the two *Nanoarchaeota* genomes and extensive gene loss, gene splitting seems to reflect the ongoing genome rearrangement and could be a consequence of the parasitic lifestyle of these *Nanoarchaeota*. Conceivably, these parasitic/symbiotic *Archaea* have extremely low characteristic effective population sizes resulting in a sharply increased role of genetic drift and consequent extraordinary genome fluidity.

### The inferred host of Nst1 is a typical member of the *Sulfolobales* with a streamlined genome

The arCOGs coverage (~92%) for Acd1 is within the range of other completely sequenced genomes of *Sulfolobales* (91-99%). Overall, the gene content of Acd1 is typically archaeal and most similar to that of other *Crenarchaeaota*. Of the 218 core archaeal genes, 217 were identified in the current Acd1 genome assembly, with only the cysteine desulfurase activator ATPase (arCOG04236) missing. Furthermore, the genome encompasses 349 of the 352 families that are shared by all 35 available genomes of *Crenarchaeaota* and span all the essential metabolic functions. This high level of coverage points to a near complete genomic assembly for this organism. Indeed, even among the gene families that are shared by all other sequenced *Sulfolobales*, the Acd1 genome encompasses 91% (1002 out of 1102). Among the well-characterized *Sulfolobales* signature genes (present in *Sulfolobales* only) that were identified in Acd1 are the chromosomal protein Sac7d, a distinct paralog of the crenarchaeal cell division protein ESCRT-III, two subunits of the terminal oxidase (DoxE and DoxD), and phosphomevalonate kinase, a possible relic of the ancestral isoprenoid biosynthesis pathway [28]. More than half of the nearly 100 *Sulfolobales* signature genes remain completely uncharacterized.

By estimating the genes loss and gain for Acd1 only a few genes were inferred to have been acquired, most of them uncharacterized (Figure 6). In contrast, the number of the genes that were likely lost exceeds 400 (Additional file 7). Conversely, for the branch leading to the rest of *Sulfolobales,* gene gain dramatically exceeds gene loss (Figure 6). Several remarkable examples of entire functional systems missing in Acd1 include two terminal oxidase complexes (SoxABCD and SoxEFGHM), fatty acid beta oxidation-related enzymes, the entire archaellum and the CRISPR-Cas system type III locus. One CRISPR-Cas system (Type I-D) is present and is most likely functional, based on the identification of several large CRISPR arrays. Predictably, the functional categories that were minimally affected by gene loss are translation, DNA replication and repair, nucleotide metabolism, signal transduction and secretion (Additional file 7). A comparative analysis of the mean number of paralogs in arCOGs shows a conspicuous deficit in Acd1, the same trend that was previously observed for *I. hospitalis*, the host of *N. equitans* (Figure 6) [29]. The reduced number of transposable elements also parallels the trend in *I. hospitalis* and is compatible with the genome streamlining hypothesis. We did not detect strong evidence of gene exchange between Nst1 and its apparent host, Acd1. Only three genes in Nst1 are significantly more similar to homologs from the host than to homologs from other Archaea and are therefore candidates for direct gene transfer between Nst1 and Acd1. These putative transferred genes encode 3-dehydroquinate dehydratase AroD (Additional file 5) and two uncharacterized proteins that so far are present exclusively in *Crenarchaeaota*.

Despite all the apparent gene loss, most of the functional capabilities of typical *Sulfolobales* seem to be preserved in Acd1, albeit in some cases in a minimal version. A type I NADH dehydrogenase complex was detected that similar to the other *Sulfolobales* lacks the NADH binding and oxidizing subunits (NuoEFG) which are predicted to transfer electrons from ferredoxin to quinones in the respiratory chain. NADH and succinate oxidation by a predicted type II NADH:quinone oxidoreductase and a succinate oxidoreductase complex (Sdh) that are encoded in the Acd1 genome could provide an alternative pathway to supply electrons to quinones. Genes encoding a heme-copper, *aa*_3_-type cytochrome oxidase (DoxBCE) and an iron-sulfur cytochrome b558/566 complex III (soxNL-cbsAB), all with high sequence similarity to homologues from other *Sulfolobales*. However, the SoxABCD and SoxEFGHM complexes that are present in the majority of other *Sulfolobales*[30] are missing in Acd1.

Most of the Acd1 central metabolism could be reconstructed based on genome analysis. Genes encoding all the enzymes of the oxidative TCA cycle, the archaeal gluconeogenic EMP pathway, glycogen synthesis and breakdown, the reversed ribulose monophosphate (RuMP) as well as C3/C4 interconversion were identified by comparison with the other *Sulfolobales*[31-33] (Figure 4). In addition, a complete hydroxypropionate/hydroxybutyrate carbon fixation pathway was assembled, suggesting that Acd1 is capable of chemoautotrophic growth. Unusually for a member of *Sulfolobales*, however, the branched Entner-Doudoroff glycolytic pathway appears to be missing, as well as the glyoxylate shunt and all enzymes for maltose and trehalose utilization (Figure 4). Although, because the genome is not complete, we cannot however rule out the possibility that some of the genes that are actually present in Acd1 are missing in the current assembly, it appears extremely unlikely that any of these pathways was missed in its entirety due to the genome incompleteness. Most of the sugar ABC and secondary transporters found in other *Sulfolobales* are also missing in Acd1, suggestive of a minimal carbohydrate metabolism. In contrast, we identified complete sets of genes for iron, manganese, phosphate and potassium transporters and sodium-calcium exchange pumps, suggesting that Acd1 is capable of uptake of most small molecules that are usually transported by free-living organisms. The genome also contains the *SlaAB* operon that encodes the S-layer proteins characterized in the other *Sulfolobales*[34], indicating that Acd1 likely has the same type of cell surface architecture.

## Discussion

Despite multiple ultrastructural, biochemical and functional genomic studies, the nature of the relationship and the mechanisms of interaction between *I. hospitalis* and *N. equitans* remain poorly understood. So far, there is no evidence for a beneficial role of *N. equitans* for its host, suggestive of a parasitic as opposed to a mutualistic relationship [12]. Experimental and genomic inferences point to the two archaea having co-evolved, with *N. equitans* using *I. hospitalis* as its sole host [12,29]. The difficulty in the characterization of their interaction and phylogenetic placing of *N. equitans* comes in large part from the unusual features of the *N. equitans* genome that make it problematic to differentiate between two distinct scenarios for the origin of the *Nanoarchaeota.* Under the first scenario, *Nanoarchaeota* represent an ancient lineage with many ancestral features whereas the alternative involves relatively recent, rapid evolution and genomic collapse driven by the parasitic lifestyle.

The analysis of the Nst1 genome described here addresses some of these questions and provides an evolutionary perspective on archaeal parasitism/symbiosis. The Nst1 genome lacks split tRNA genes but encompasses split protein genes, so that the existence of both unique and shared gene splits between the two *Nanoarchaeota* provides insight into the evolution of this feature. It now appears most likely that genome reduction, probably by intrachromosomal recombination and deletion events, led to stochastic fragmentation of multiple genes, with those split in locations compatible with functional enzymatic reconstitution *in trans* being retained. The presence of several genes with the same split site between the two *Nanoarchaeota* implies that this process predated the separation of the two lineages. Together with multiple common gene losses, these shared gene splits suggest that the most recent common ancestor of the terrestrial and marine *Nanoarchaeota,* represented by the two current representatives, already was a symbiont or parasite that was undergoing genome shrinkage. Given the large evolutionary distance between *N. equitans* and Nst1 and the fact that they inhabit different environments and appear to employ highly diverged *Crenarchaeota* as hosts, the radiation of the two lineages probably was an ancient event. Genome contraction apparently has continued after ecological separation and by either host specialization or switching. The larger genome of Nst1 and the presence of some primary metabolic functions indicate that the terrestrial nanoarchaeon has not reached the advanced genomic collapse stage that is characteristic of its marine sister group. The complete absence of split tRNA genes in Nst1 and the presence of the RNase P machinery is compatible with this scenario, indicating that these unique alterations of the *N. equitans* translation system evolved more recently as a result of the extreme genomic degradation in this lineage. Genome sequencing of other members of the *Nanorchaeota* should aid in further elaborating this scenario.

Phylogenetic analysis of the two *Nanoarchaeota* revealed a strongly supported, deep branching clade that was originally proposed to represent a distinct phylum [1]. Clear affinities to the *Euryarcheaota*, in terms of shared gene content, are maintained, as previously pointed out [6], but such deep affinities might predate phyla divergence, such as those between *Korarchaeota* and *Crenarchaeota*[35] and between *Thaumarchaeota* and *Crenarchaeota*[36]. Conceivably, the evolutionary driving force that led to the separation of *Nanoarchaeota* from the *Euryarchaeota* was an ancient symbiotic event, with the corollary that all members of the *Nanoarcheaota* could be symbionts or parasites. Similar to *N. equitans,* Nst1 apparently relies on an external source of almost all building blocks, with the probable exception of some amount of ATP and NADH that could be produced by glycolysis. The absence of an ATP synthase, while extraordinary, remains to be confirmed by genome closure. It is notable, however, that even in *N. equitans*, the assembly of a functional ATP synthase complex has not been yet demonstrated and remains uncertain given the absence of the genes for several subunits [37], even though the present ones are expressed [38].

Although a specific host-symbiont/parasite association between Acd1 and Nst1 requires formal proof by isolation and cultivation of the two organisms in the laboratory, the results presented here strongly suggest a relationship between these two organisms. The lack of readily detectable genomic and physiological complementarity between *N. equitans* and its host implied that *N. equitans* is a parasite rather than a mutualistic symbiont [5,12,29]. Despite the initial hypothesis that large membrane vesicles may transfer proteins and lipids from the host to *N. equitans*[5], whole cell proteomic measurements have not found evidence of significant amounts of biosynthetic enzymes being transported from *Ignicoccus* to *N. equitans*[38]. The transfer of small molecules from *I. hospitalis* (confirmed for lipids and aminoacids [12,39]) must occur therefore through membrane transporters or a specialized structure that may be present at the point of contact between the cells [40]. The second archaeal parasite(symbiont)-host pair described here adds further complexity to the question how these relationships are established and maintained. The membrane organization and gene content of *I. hospitalis* and Acd1 differ substantially. Genome analysis indicates that Acd1 is closely similar to other *Sulfolobales* and likely has the characteristic S-layer membrane [34], distinct from the unique double membrane that is a hallmark feature of *Ignicoccus*[41], genus so far exclusively marine*.* Thus, the two *Nanoarchaeota* might have evolved independent mechanisms to interact with their hosts and/or share common genes and structures for acquiring metabolic precursors that remain to be characterized. Although Nst1 generally resembles *N. equitans* in lacking readily detectable inferred functionalities that could complement functions missing in the host, a major exception is the archaellum that is encoded in the Nst1 genome but apparently not in the genome of Acd1. Cellular appendages have been observed occasionally in *N. equitans*[42] but their nature and roles are unknown, and the genome lacks recognizable archaeallum genes [18]. The predicted Nst1 archaeallum might provide motility or attachment capabilities for the nanoarchaeon cell and perhaps even its associated host, a possibility that remains to be explored once the cultivation of these organisms is achieved.

Despite the substantial differences in the gene repertoires and the lack of specific common trends, the two hosts of *Nanoarchaea* do share a prominent common trait, genome streamlining. Indeed, *I. hospitalis* has the smallest genome amongst *Crenarchaeota*, whereas the Acd1 genome, even though larger, is by far the smallest among the *Sulfolobales*. Reconstruction of genome evolution indicates that the reduced gene repertoire is not an ancestral feature but a derived one caused by extensive gene loss. The shrinking of functional capabilities, in particular in defense systems, might have made these organisms vulnerable to parasite infestation or conversely, the host genome shrinkage was a result of the relationship with their nanoarchaeal companions; these two possibilities are not necessarily mutually exclusive. Characterization of additional archaeal parasite/symbiont-host systems from such geochemically diverse ecosystems as marine and terrestrial thermal habitats will show how general these trends are and what evolutionary forces and mechanisms drive them.

## Conclusion

For a decade, the only specific association between two Archaea involved the ectoparasite *Nanoarchaeum equitans* and its marine hyperthermophile host, *Ignicoccus hospitalis*. *N. equitans* is a deep archaeal lineage, with a tiny genome, enriched in split genes, and lacking primary metabolism. We sequenced the genome of the first hyperthermophilic *Nanoarchaeota* from a terrestrial environment (Yellowstone National Park) and its likely archaeal host. A larger genome, fewer split genes, and existing carbohydrate metabolism indicate that nanoarchaeal symbiosis predates the divergence of terrestrial and marine lineages, and resulted in distinct gene loss and fragmentation. This second symbiotic system enhances understanding of interspecies interaction and evolution of archaea and will guide future research on characterizing the molecular and cellular mechanism involved in these archaeal symbiotic associations.

## Methods

### Environmental sample collection and analysis

Samples consisting of a mix of water and gravel sediment (T=82°C, pH=5.2-5.5) were collected from Obsidian Pool from the Mud Volcano area of Yellowstone National Park, Wyoming (USA) using glass bottles with butyl rubber stoppers with no air head space. The samples were reduced with sodium sulfide (0.02%) on site and were stored at 4°C. Sample aliquots were used for DNA extraction using a MoBio PowerSoil kit (Carlsbad, CA). The presence of *Nanoarchaeota* was confirmed by specific amplification of the SSU rRNA gene with the primers 7mcF and 1511 mcR [7] and Sanger sequencing and by 454 pyrosequencing of the V4 region of the SSU rRNA (9003 total archaeal sequences) as previously described [9].

### Immunofluorescence staining and single-cell sorting

A rabbit polyclonal antibody against *N. equitans* was developed using cells purified from a late stage *I.hospitalis-N.equitans* co-culture (generously provided by H. Huber, Univ. of Regensburg). The antibody was fluorescently labeled with DyLight488 or DyLight650 (Thermo Scientific, Rockford IL). To optimize the staining conditions we used fresh laboratory co-cultures of *I.hospitalis*-*N.equitans*[12]. Cells were sedimented by centrifugation (10 min at 8,000 g) and resuspended in 500 μl blocking buffer (5% goat serum in phosphate buffered saline) for 30 minutes. Fluorescently-labeled antibody was then added (1:100–1:500 dilution) and incubation was continued in the dark for 30 minutes. Co-staining with a different labeled rabbit antibody developed against pure *I.hospitalis* cells were also conducted. After centrifugation and washing three times with blocking buffer, the cells were suspended in UV-irradiated PBS and used for epifluorescence microscopy or flow cytometry. A similar procedure was also applied to Obsidian Pool environmental samples except that staining was only performed with the anti-*N.equitans* antibody and un-labeled *I.hospitalis* was added to the blocking solution (50 μg/ml) to minimize potential background from conserved archaeal proteins shared between *Nanoarchaeota* and other environmental *Archaea* in the Obsidian Pool.

Flow cytometry and single cell sorting were performed using a Cytopeia Influx Model 208S (Cytopeia, Seattle, WA) equipped with two lasers (488 and 641 nm). Prior to use, the sorting chamber was sterilized using the built-in germicidal UV lamp and the fluidic lines were cleaned using a 10% bleach solution followed by rinsing with sterile, DNA-free water and PBS similar to what has been previously described [43,44]. A fluidic line cleaning was performed between each different sample to avoid cross-contamination. The sorting gates were determined using the control *I. hospitalis-N. equitans* and by comparing the fluorescence scatter plot intensity and distribution at different antibody dilutions when applied to the Obsidian Pool samples. Single fluorescent particles were deposited in 3 μl of DNA-free Tris–HCl 10 mM pH 8.0, 1 mM EDTA (TE) in individual wells of 96 well plates*.*

### Multiple displacement amplification and taxonomic analysis

The single cell-type particles were subjected to genomic multiple displacement amplification (MDA) [45]. To minimize the risk of contamination with exogenous DNA, all disposable plastic ware, water and reagents (except the DNA polymerase, dNTPs, DTT and the hexanucleotides) were exposed to UV for 30 minutes in a Stratalinker (Stratagene, La Jolla CA). The cells were lysed and their DNA denatured by addition of 3 μL of 0.13 M KOH, 3.3 mM EDTA, 27.7 mM DTT, heated to 95°C for 30 sec followed by cooling on ice for 10 min. 3 μL neutralization buffer (0.13 M HCl, 0.42 M Tris-Cl pH 7.0, 0.18 M Tris-Cl 8.0) was then added followed by 11 μL of MDA master mix that contained 90.9 μM random hexamers containing two phosphorothioate bonds on the 3′ end (Integrated DNA Technologies, Coralville, IA, USA), 1.09 mM dNTPs (Roche Indianapolis, IN, USA), 1.8× phi29 DNA polymerase buffer (New England BioLabs, Ipswich, MA, USA), 4 mM DTT (Roche) and 100 U phi29 DNA polymerase, purified in house (DNA polymerase clone courtesy of Dr. Paul Blainey, Stanford University) [46]. Amplification was conducted for 10 hrs at 30°C followed by enzyme inactivation at 80°C for 20 min.

To determine which MDA reactions yielded products and to identify their taxonomic affiliation, 1:100 dilutions of the SAG products were used for SSU rRNA gene amplification in a 96-well plate format using both *Nanoarchaeota*-specific primers (7mcF-1511mcR) and broad *Archaea* primers (excluding *Nanoarchaeota*)(515AF2-1492R). Control reactions were conducted using single cell-type particles isolated from a *N. equitans - I. hospitalis* co-culture using the antibody-based sorting approach and yielded ~20% Nanoarchaeota-positive SAGs. For the Obsidian Pool sample, five SAGs tested positive for *Nanoarchaeota* and they also yielded products when amplified with the general *Archaea* primers. The amplicons were sequenced bidirectionally using Sanger chemistry. The sequencing chromatograms were assembled into complete SSU rRNA genes and were analyzed using the Geneious software (Biomatters Ltd). All individual amplicons were homogeneous, with single peak chromatograms, indicative that they did not represent multiple organisms. Because for all five SAGs the SSU rRNA sequences corresponded to the same *Nanoarchaeota* and the same distinct associated archaeon, an uncultured *Sulfolobales* that is present in very low abundance in Obsidian Pool, we infered that the sorted single cell-type particles contained two organisms, the *Nanoarcheota* Nst1 physically attached to a cell of another species, its likely *Sulfolobales* Acd1 host. Based on pyrosequence data, both organisms were present at low but comparable levels in the archaeal population (2.4% Nst1 and 6% Acd1, each at >97% sequence identity to the single cell SSU rRNA data).

Phylogenetic reconstructions based on rRNA sequences were conducted by first aligning the sequences using NAST [47], masking the highly variable loop regions for which positional homology could not be inferred (872 nucleotide final alignment length) The most likely tree topology and bootstrap-based nodes support were calculated with MEGA5 [48] under a General Time Reversible (GTR+I) model, with gamma distributed rates (6 categories) and extensive subtree-prunning-regrafting (SPR level 5) heuristic search.

### SAG sequencing and assembly

Each of the five *Nanoarchaeota* SAG DNAs was purified by phenol-chloroform extraction and ethanol precipitation. Approximately 2 μg of each DNA was used for generating a 300 bp fragment size library followed by paired-end sequencing using the Illumina HiSeq platform at the HudsonAlpha Institute for Biotechnology (Huntsville, Al). For each SAG we obtained 80–110 million 100 nt long reads (8–11 Gbp sequence). To reduce the uneven sequence distribution associated with MDA [43,49,50] and reduce the sequencing artifacts we applied a digital normalization step using the Khmer package [51]. Reads above 30x coverage as well as singletons (likely to contain sequence errors and MDA artifacts) were removed (khmer scripts: normalize-by-median.py -C 30 -k 30; filter-abund.py). This resulted in 466–563 thousand reads for each dataset, with an average length of 99.7 nucleotides.

The first pass assembly was performed using Velvet (version 1.2.03) [52]. For each SAG dataset we tested the effect of the hash length (k) on the output (number of contigs, total assembly size and N50/maximum contig length), with auto settings for coverage level and no scaffolding of contigs. For all datasets, k values of 59–61 gave the best results, with an average number of contigs of 142, N50 of 15.2 kb and maximum contig size of 56 kb. A compositional analysis of the contigs revealed two distinct populations, one with a median G+C% of 25% and the other of 54% (Additional file 1, A). A sliding window tetranucleotide frequency analysis using a Java program (KmerFrequencies.jar implemented for metagenomic data binning in the JGI IMG-MER platform [53] also separated the two populations of contigs (Additional file 1, B). To improve the assembly, resolve the Nst1 and Acd1 genomes and correct artifacts we combined several approaches. Gene prediction and preliminary annotation was performed both in IMG-MER and RAST [54]. Blast tables were generated using the CLC Genomics Workbench (CLCBio.com) to analyze the taxonomic distribution of hits for each gene from each contig. The five SAGs Velvet assemblies were then merged and co-assembled using the *de novo* assembly algorithm implemented in Geneious (Biomatters Ltd, Auckland NZ). Annotation information was used to analyze the gene syntheny between the contigs across SAGs, separate the two genomic data sets and identify and correct assembly artifacts (primarily inversions and duplications at the end of contigs due to repetitive regions). To confirm and correct such errors we performed PCR and Sanger sequencing using both SAGs DNA and original environmental sample DNA. Further gap closing was achieved by gap PCR and sequencing using combinations of primers developed for the ends of each contig and contig end extension by remapping of Illumina reads in Geneious. Several small contigs (<1% of the data) were removed as they either represented likely contamination (human and bacteria DNA) based on composition and taxonomic blast hits. The final dataset (7 contigs for Nst1 and 8 contigs for Acd1) was subjected again to tetranucleotide frequency analysis in comparison to the genomes of *N. equitans* and *I. hospitalis*. Because the Nst1 and Acd1 draft genomes combine the genetic information from five separate cells isolated from the environment and also include metagenomic sequence generated during contig walking and gap closing they represent pangenomic assemblies for the two uncultured organisms present in the Obsidian Pool. While we identified the presence of nucleotide polymorphisms, primarily synonymous substitutions, the limited overlapping data and the few number of SAGs precluded an in-depth analysis of the natural variation present in the natural population. The final gene calls and annotations used the IMG_MER [53] and RAST systems and were further curated and updated based on the arCOG information [13,14]. In addition to the automated gene prediction from IMG and RAST we performed local searches for additional RNA genes. Both tRNAScan-SE [55] and SPLITS [56] were used to search for additional tRNAs and potential non-contiguous split tRNA genes. Infernal v1.0.2 [57] was used to search for RNase P RNA genes using the standard archaeal covarion model. RNA secondary structures were predicted using Mfold [58]. Small regulatory RNAs were predicted using Snoscan (v0.9b) [59]. The annotated genomic datasets are available in IMG ( http://img.jgi.doe.gov). The Whole Genome Shotgun projects have been deposited at DDBJ/EMBL/GenBank under the accession APJZ00000000 (PRJNA189432) and APJY00000000 (PRJNA189433).

### Comparative genomic and phylogenetic analyses

Nst1 and Acd1 proteins were assigned to arCOGs using the updated archaeal Clusters of Orthologous Groups database (arCOGs) [13,14], which contains 120 archaeal genomes. To calculate the frequency of paralogs in archaeal genomes, blastclust analysis was performed using the translated coding sequences and varying similarity threshold for sequence clustering.

Protein sequences were aligned using MUSCLE [60]. The PSI-BLAST program [61] was used to perform iterative sequence similarity search in the non-redundant protein sequence database at NCBI (NIH, Bethesda). Positions with more than 50% of gaps and/or homogeneity <0.1 were removed from the alignment for phylogenetic analysis [62]. Approximate maximum likelihood phylogenetic trees were reconstructed with FastTree [63,64]. To compare evolutionary models we used ProtTest [65] and the optimal model and parameters were applied to reconstruct maximum likelihood phylogenetic trees using RAxML [66].

The Count software was used to infer gene gains and losses on the branches of the 120 archaea species tree from the matrix of phyletic patterns (containing a presence/absence information for each genome in an arCOG) by the likelihood maximization method based on a phylogenetic birth-and-death model [67]. The phylogenetic tree based on concatenated r-proteins was used as the guide topology. Then, Nst1 and Acd1 were placed in this tree; maximum parsimony was employed to infer gene gain and loss for these two species relying on the previously inferred states at ancestral tree nodes.

### Genome size estimate calculation

To estimate the completeness of Nst1 and Acd1 genomes using gene families that are likely ubiquitous in *Archaea* we applied the same rationale as in [15]. Let us consider *F* protein families that we expect to be present in the Nst1 and Acd1 genomes (e.g. we expect all 138 gene families found in each closed archaeal genomes to be also present in all new genomes). Suppose that the sequenced part of a new genome contains *N* predicted genes that include members of *f* families from the universal set. Assuming that all genes are equally and independently likely to be present in the sequenced part of the genome, we estimate the completeness of the sequence as *p* = *f*/*F*; the complete genome is expected to contain *N*’ = *N*/*p* = *NF*/*f* genes.

Although *N*’ gives the most likely estimate, the actual number can be larger or smaller due to sampling fluctuations because the completeness *p* is estimated from a subset of genes. Let us consider how much we expect the estimated *p* to deviate from the “true” measure of completeness *p*’.

Suppose that *p*’ ≥ *p* (i.e. *p* underestimates *p*’). In this case, we expect to find *f*^+^ ≥ *f* families from the ubiquitous set. Assuming random independent sampling of families, the probability to sample *f* or less families out of *F* possible is $P^{+}\;=\;\sum_{i=0}^{f}\mathrm{bin}\left(p^{+},\;i,\;F\right)$ where *bin*(*p*^+^,*i*,*F*) is the binomial probability to get *i* successes out of *F* trials with the probability of success in a single trial *p*^+^. We can find numerically such *p*^+^ ≥ *p* that *P*^+^ = α where α is the desired significance level (e.g. by setting α = 0.05 we are 95% confident that *p*’ ≤ *p*^+^). Likewise, for *p*’ ≤ *p*, the expected number of sampled families is *f*^-^ ≤ *f* and the probability to sample *f* or more families out of *F* possible is $P^{-}\;=\;\sum_{i=f}^{F}\mathrm{bin}\left(p^{-},\;i,\;F\right)$. Finding such *p*^-^ ≤ *p* that *P*^-^ = α we obtain the lower estimate for *p*’ (*p*’ ≥ *p*^-^) with confidence 1-α. The 1-α confidence interval for the genome size estimate is [*N*/*p*^+^, *N*/*p*^-^].

The remaining issue is the choice of the *F* protein families that are likely to be present in the genome for which the estimate is produced. Obviously, the larger *F*, the more precise is the estimate for *p*, and the tighter the confidence interval for the total genome size estimate. Nevertheless, it stands to reason to use only such families that are represented by a single ortholog in all genomes. For this set, the plausible assumption of random independent sampling of genes translates directly to the random independent sampling of families. Families with multiple paralogs are, technically, not equally likely to be present in a random sample of genes, and this depends on the unknown distribution of paralogous family sizes.

For the purpose of this study, we chose to use the largest possible set (ubiquitous archaeal families for *Nst1* and ubiquitous crenarchaeal families for *Acd1*) regardless of the number of paralogs found in other species. The reason for this is two-fold. First, both species are relatively poor in lineage-specific gene family expansions, making the impact of paralogy less important. Second, due to the nature of sequencing and assembling shotgun metagenomic and SAG reads into contigs, the assumption of random independent sampling of genes is questionable (when reads are missing for a particular gene, the contig is disrupted and neighboring genes are also less likely to appear in the final gene set). The resulting estimation for Nst1 genome points to at least 91% genomic completeness, with 692 to 761 genes for the full gene complement (at 95% confidence). For Acd1, the assembly is estimated to be 99% complete, with the 95% confidence interval of 1696 to 1730 genes.

## Abbreviations

SSU rRNA: Small subunit ribosomal RNA; MDA: Multiple displacement amplification; SAG: Single-cell amplified genome; G+C content: Percent of guanine plus cytidine nucleotides in DNA sequence; arCOG: Archaeal cluster of orthologous genes

## Competing interests

The authors declare that they have no competing interests.

## Authors’ contributions

MP, EK and ALR designed the study. MP performed the experiments. MP, KM, DG, YW and EK analyzed the data. MP, KM, DG, YF, EK and ALR wrote the manuscript. All authors read and approved the final manuscript.

## Reviewers’ comments

Reviewer 1: Patrick Forterre, Institut Pasteur

The paper by Podar and co-workers describes the discovery, by single cell isolation, of a second nanoarchaeon, called Nst1, ten years after description of the first one, Nanoarchaeum equitans. This was a long awaited discovery, considering the interest of these unusual microbes and the long-standing controversy about their nature, origin and position in the archaeal phylogenetic tree. Importantly, this second nanoarchaeon (terrestrial) is only distantly related to the first one (marine) in term of genome sequence. Furthermore, whereas the host of N. equitans, Ignicoccus hospitalis is a Thermoproteales, the host of N. Nst1 is a deeply branching Sulfolobales. This allows the authors to obtain very interesting and important information about the origin and evolution of Nanoarchaea. Their analysis, especially the comparison of split genes in both species, confirm that Nanoarchaea are not the most primitive archaeon, as it has been sometimes suggested, but highly derived organisms that evolved by reduction from free-living ancestors with larger genomes and non split genes. The phylogenetic analyses also confirm the close relationships between Nanoarchaea and other Euryarchaeota, “Nanoarchaeota” emerging at the base of this phylum. This lets open the possibility to consider Nanoarchaea as members of a specific phylum, Nanoarchaeota, or as bona fide Euryarchaeota. In fact, there is still some confusion in the literature on this point, and the authors use the term Nanoarchaeota in that paper suggesting that they favour the phylum status for this group. It was originally proposed that Nanoarchaea represent an entire new phylum, based on rRNA tree in which they branched between Crenarchaeota and Euryarchaeota [1]. The same result was obtained later on with ribosomal protein phylogenies [6]. However, careful analyses shown that Nanoarchaea are more likely bona fide Euryarchaeota distantly but specifically related to Thermococcales. Single ribosomal protein phylogenies revealed that the intermediate position of N.equitans in the ribosomal protein tree (between Crenarchaeota and Euryarchaeota) was also due to the existence of a several ribosomal proteins with crenarchaeal affinity, especially in the large ribosomal subunit [6]. Does this observation hold on with this new genome? This should be an interesting point to test. The authors also obtained a basal position for Nanoarchaea in an RNA polymerase tree. However, it has been previously shown that the archaeal RNA polymerase tree is prone to long-branch attraction artifact [68]. In particular, this explains the basal position of Methanopyrus kandleri, whose RNA polymerase exhibit a high evolutionary rate, both in previously published RNA polymerase trees and in the Figure 3 of the supplementary (where it branch together with Nanoarchaea and Korarchaeota). Importantly, the clustering of Nanoarchaea and Thermococcales was also suggested by a strong synapomorphy, the presence of bacterial like RumA-type tRNA(uracil-54,C5)-methyltransferases whose genes having been probably acquired via a single horizontal gene transfer from a bacterial donor to the common ancestor of Thermococcales and Nanoarchaea (PAB0719 and PAB0760 in Pyrococcus abyssi) [69]. It would be interesting to know if these genes are also present in the new nanoarchaeon. More generally, is it possible for the authors to find or not in this new genome additional evidence for distant but specific relationships between Nanoarchaea and Thermococcales?

Response: *In this paper we use the term Nanoarchaeota inasmuch as it is by now widely accepted including in the NCBI taxonomy and J.P. Euzéby’s list of prokaryotic names. In itself this conventional usage is not intended to express our support of the phylum status of nanoarchaea. That noted, we see no evidence against such taxonomy. As we point out, on the weight of the combined evidence from phylogenetic analysis and, perhaps more important, gene content analysis, we are inclined to consider Nanoarchaeota a highly divergent sister group of Euryarchaeota*. *This does not invalidate Nanoarchaeota as a phylum. The probable affiliation of Korarchaeota and Thaumarchaeota with Crenarchaeota in the ‘TACK’ superphylum potentially reflects a similar special relationship between these archaeal phyla but does not invalidate the phylum status of either of them. Certainly, specific affiliation of the nanoarchaea with the Thermococcales would have been a different matter. However, we did not obtain any clear evidence of such a connection. It is not supported by any of the phylogenies that we examined, and with regard to the proposed comparison of trees for individual r-proteins, we believe that this is a complicated and risky exercise given the small size and different amino acid composition biases of these proteins. A majority of tRNA-modifying enzymes encoded by the two nanoarchaeal genomes are most similar to homologs in the Thermococcales*, including *the RumA-type tRNA(uracil-54,C5)-methyltransferase that is present in both nanoarchaeal genomes. However, this rumA homolog is the only gene shared exclusively with Thermococcales, and it is not necessarily a synapomorphy –it could be a symplesiomorphy or a case of horizontal gene transfer. Both nanoarchaea, for example, encode the rRNA (guanine(1405)-N(7))-methyltransferase that is not present in any other archaeal genomes but is shared with Bacteria instead We do not believe that this homoplasy should be interpreted as evidence that Nanoarachaea have a close affiliation with bacteria.*

We certainly cannot rule out at this time that the (near)basal position of the Nanoarchaeota in the archaeal tree is due to the long branch at the base of this group. However, we believe that a comprehensive phylogenomic analysis that is required to clarify the evolutionary history of this unusual group of Archaea is beyond the scope of the present paper. Furthermore, this analysis critically depends on a representative genome collection and has a much greater chance to succeed when additional genomes of nanoarchaea, perhaps less advanced along the route to the parasitic life style than the two current ones, become available.

Reviewer 2: Bettina Siebers, Universität Duisburg-Essen (Nominated by Michael Galperin)

In their article “Insights into archaeal evolution and symbiosis from the genomes of a Nanoarchaeon and its crenarchaeal host from Yellowstone National Park” the authors Podar et al. report the exciting finding of a new terrestrial member of the Nanoarchaeota (Nst1) associated to a novel crenarchaeal host (Acd1), a member of the Sulfolobales. Using a labeled antibody against *Nanoarchaeum equitans*, flow cytometry as well as genomic amplification the authors were able to unravel most of the genome sequence of Nst1 (ca. 91%) as well as Acd1 (ca. 92%).

Detailed bioinformatics/phylogenetic analysis were performed and revealed for Nst1 compared to *N. equitans* in accordance with its larger genome size some interesting additional metabolic and cellular features like the presence of gluconeogenesis and a complete set of genes encoding proteins of the archaellum. In addition with the increased genome size also the number of split genes decreased in Nst1 compared to N. equitans. However, despite this increased complexity Nst1 still misses essential biosynthetic properties reflecting its symbiotic/parasitic life style. Phylogenetic analysis using ribosomal sequences propose that Nst1 is a member of a distinct family within the Nanoarchaeota, which form a distinct deep branching taxon within the Archaea. Phylogenetic/bioinformatics analysis of the inferred host Acd1 point to a significantly streamlined genome compared to other members of the Sulfolobales. Intriguingly, the genome reconstruction points to an autotrophic life style of Acd1 (e.g. hydroxypropionate/hydroxybutyrate cycle for CO2 fixation, TCA cycle, gluconeogenesis, glycogen synthesis) with significantly reduced carbohydrate metabolism (e.g. homologs for the glycolytic branched ED pathway, glyoxylate shunt, two terminal oxidases are missing; reduced number of ABC transporters). In contrast to Nst1 no genes encoding the archaellum were identified.

In summary the finding of this new terrestrial symbiotic/parasitic couple offers exciting possibilities for future research in order to unravel the enigmatic relationship of Nanoarchaeota to their hosts.

The manuscript is very well written and all important information is given in the manuscript or in the Additional files, therefore I have only few minor points that the authors may want to consider.

(1) When the authors discuss the inferred physiological features I would suggest to mention again briefly that the genome is not complete and therefore some genes/features might be missed.

Response: *We reiterated that in discussing the Acd1 inferred physiology, as suggested, but also indicate that loss of entire pathways due to the genome incompleteness is unlikely.*

(2) Paragraph “physiological features of Nst1 (line 10)” what kind of “glycosyltransferase system” are the authors referring to, please specify. May be it would be worth mentioning the Nst1 seems to harbor no pathways for pentose generation (Figure 4). Did the authors analyze for homologs of the classical pentose phosphate pathway (i.e. oxidative/non-oxidative part) as well as the reversed ribulose monophosphate pathway (depicted in Figure 4).

Response: *In the subsequent paragraph, we list several enzymes involved in nucleotide sugar activation and glycosyltransfer reactions. The Nst1 genome encodes a number of predicted glycosyltransferases with ambiguous specificity; see, for example, the cluster of genes* Nst1_523-525*. Dolichol phosphates appear to be common carbohydrate carriers for archaeal post-translational protein modification, but additional biochemical research will be required to determine the specificity of divergent homologs of STT3 and dolichyl-phosphate mannose protein mannosyltransferases, as well as a putative pathway for nucleotide-activated 6-deoxyhexose biosynthesis –all predicted in Nst1.*

We did not identify enzymes of the pentose phosphate pathway in Nst1. Due to the incomplete genome sequence and the overwhelming number of genes and pathways “missing” from Nst1, we have only commented on pathways missing from one nanoarchaeon but present in the other.

(3) May be it would be interesting to add a list of Sulfolobales signature genes (absent & present) in Acd1 as Additional files in addition to gain and losses (Acd1/Acd1 and other Sulfolobales) in Additional file 7.

Response: *We added a table of absence and presence of Sulfolobales ancestral genes in Acd1 (Additional file7).*

Reviewer 3: Purification Lopez-Garcia, Universite Paris Sud

This manuscript presents the analysis of the nearly complete genomes of two archaea potentially involved in a co-specific symbiotic relationship related, respectively, to *Nanoarchaeum* and to the Sulfolobales (Crenarchaeota). The genomes of the two organisms were assembled from 5 different single-amplified genomes (SAGs) from fluorescently labeled cell-sorted cells (using fluorescently labeled antibodies against Nanoarchaeum equitans). Based on the observed number of expected genes, the genomes are estimated to be >90% complete. This work is important because it provides a second example of a highly reduced nanoarchaeal genome (although less than the so far only available sequence of N. equitans). Comparative genomic analysis provides an interesting insight on the evolutionary process of genome reduction accompanying obligatory symbioses. It also provides information about the metabolic pathways present in each of the two symbiotic partners, although it is not possible to make firm conclusions about missing functions when the genomes are not fully sequenced, especially when the number of genes involved in such functions is small and/or those genes organize in operons.

Despite the interest of this genomic analysis, I have a major concern with the phylogenetic analyses shown and their interpretation. The authors present two alternative hypotheses for the placement of Nanoarchaeota and say that their new phylogenetic analyses including the new Nst1 sequences allow distinguishing between the two. The first hypothesis places the Nanoarchaeota in a basal position in the archaeal tree; the lineage would have then retained ancestral features. The second hypothesis places the Nanoarchaeota within the Euryarchaeota, being likely related to the Thermococcales; in this case, relatively recent, rapid evolution linked to the parasitic lifestyle would have led to genome collapse (1st paragraph, page 14 in the ms). However, first, I have the impression that, from the beginning, the authors are not neutral and favor an a priori basal Nanoarchaeum position, as they state in page 5 prior to the description of their phylogenetic results: “Because Nanoarchaeota form a deep archaeal branch…”. And second, such a basal position cannot be deduced from their analyses to the exclusion of a euryarchaeotal affiliation because:

1) Most phylogenetic trees presented in the manuscript show a monophyletic grouping of the Nanoarchaeota and the Euryarchaeota. This is the case in the maximum likelihood phylogenetic tree of concatenated ribosomal proteins in Figure 3, where the two nanoarchaeota form a monophyletic group with the Euryarchaeota with 100% bootstrap value. This relationship can be even seen in the phylogenetic analysis based on SSU rRNA genes only (Figure 1), where several nanoarchaeota sequences form a monophyletic group (98% bootstrap for both ML and neighbor joining analyses) with the only euryarchaeotal sequence (Pyrococcus furiosus) included in the tree. I would be curious to see what happens when a more balanced and representative taxon sampling, including more euryarchaeotal SSU rRNA sequences is included in the analysis.

Response: *We made a new SSU rRNA tree that includes most of the taxa used in the construction of the r-proteins tree. Some of the major nodes have poor support, regardless of the phylogenetic method or software used and place Methanopyrus as a basal lineage in Euryarchaeota, a result that has been previously observed and discussed*[68].

2) The monophyly of Nanoarchaeota and Euryarchaeota is retrieved in most of the trees presented even if the phylogenetic analyses may be affected by a long-branch attraction artifact due to i) the acceleration of the evolutionary rate experienced by the genes of the symbiotic/parasitic nanoarchaeota and ii) the extreme low GC content bias (24% GC). Since the authors demonstrate that a genome reduction process accompanied by gene splitting and an increasing low GC content (typical of symbiont/parasite fast-evolving genomes) is ongoing in these nanoarchaeota, the reasonable null hypothesis in this case would be that these archaea are displaced towards the base of the archaeal tree by a long-branch attraction artifact (LBA). To truly discriminate between the two hypotheses, analyses tending to eliminate potential LBA, such as the use of sequence evolution models that accommodate better differences in evolutionary rates (e.g. CAT), the progressive elimination of fast-evolving sites from their analyses or amino acid recoding would be needed.

3) The gene content of the nanoarchaeotal genomes would reinforce a euryarchaeotal position, as the authors suggest at a given point. In this sense, it would be interesting to show a parsimony gene loss/gain analysis not (or not only) from the last universal archaeal ancestor but most of all from the last common euryarchaeotal ancestor.

Given the nanoarchaeal-euryarchaeal monophyletic signal already observed in many of the phylogenetic trees shown and the euryarchaeota-like gene content of their genomes, the most parsimonious hypothesis would be that the Nanoarchaeota are reduced, fast evolving euryarchaeota. Whether they are specifically related to the Thermococcales or to any of the several non-methanogenic euryarchaeotal lineages for which genome sequences are not available remain unresolved.

Response: *There seems to be a degree of misunderstanding involved here. We indicate in the article that “This observation [on the existence of 17 genes that are shared by Nanoarchaeota and most of the Euryarchaoeta to the exclusion of Crenarchaeota] complements the results of phylogenetic analyses and is best compatible with a common ancestry of Nanoarchaeota and Euryarchaeota.” We also agree with the reviewer that beyond the likely euryarchaeal affiliation, there is no evidence of a more specific affinity with Thermococcales or any other euryarchaeal branch (see also the response to Patrick Forterre’s review). Nowhere in the manuscript do we claim support for a basal position of the Nanoarchaeota among the Archaea let alone the retention of ancestral features in the Nanoarchaeota. Quite the contrary, we emphasize in the Abstract: “These findings imply that, rather than representing ancestral characters, the extremely compact genomes and multiple split genes of Nanoarchaeota are derived characters associated with their symbiotic or parasitic lifestyle.” The phrase “deep branch” that we use does not imply “basal branch”, it simply means that Nanoarchaeota do not belong to any of the specific groups of the archaea below the phylum level. Thus, to the best of our understanding, there is no substantial disagreement between our statements in the article and the reviewer’s position on the evolution of the Nanoarchaeota.*

Other comments/questions:

- Can the authors rule out the possibility of (partial) chimeric assembly during the amplification, SAG and metagenomic sequencing and assembly process? This might be particularly important when considering the potential acquisition of genes by horizontal gene transfer, especially when close homologs are not present in Nanoarchaeum equitans.

Response: *We assume that the reviewer refers here to the potential chimeric assembly between reads of Nst1 and Acd1. This is indeed an important question in the assembly of multi-genomes datasets. Because the two genomes have such a large difference in nucleotide composition (both G+C% and tetranucleotide composition), we are quite certain such chimeric assemblies did not occur and we did not see any evidence based on operon structure that genes belonging to either Nst1 or Acd1 were inserted in the other’s assembly. That being said, the definitive answer will come with the isolation and complete sequencing of the two organisms’ genomes.*

- Specify whether ambiguously aligned positions were removed and provide the number of non-ambiguously aligned positions used for each tree in the figure legends and/or methods.

Response: *Such information is included in the revision*.

- Please, check the use of capital letters and italics for Latin nomenclature only (not for vernacular names such as “nanoarchaeon”). Use italics only for genera and species names in phylogenetic trees.

Response: the usage was made consistent to the best of our understanding.

## Supplementary Material

Additional file 1 Presents the GC content distribution in the first pass assembly of the Illumina reads and a principal coordinates projection of the tetranucleotide frequency distribution in those contigs. Click here for file

Additional file 2 Is a table containing gene annotation information for Nst1 and Acd1. Click here for file

Additional file 3 **Shows maximum likelihood phylogenetic trees of the *Archaea* based on concatenated ribosomal protein gene sequences and RNA polymerase subunits from all the completely sequenced genomes.** Click here for file

Additional file 4 Shows a maximum likelihood phylogeny of archaeal lysyl-tRNA synthetases of class II (arCOG00408). Click here for file

Additional file 5 Shows maximum likelihood phylogenies of archaeal FlaH archaellum and 3-dehydroquinate dehydratase (AroD, arCOG2097) proteins. Click here for file

Additional file 6 **Lists the arCOGs present in both *N. equitans* and Nst1.** Click here for file

Additional file 7 **Lists the gene gain and loss in *Nanoarchaeota* and in*Sulfolobales* based on arCOG classification.** Click here for file
