# Supplementary material for: Insights into archaeal evolution and symbiosis from the genomes of a nanoarchaeon and its inferred crenarchaeal host from Obsidian Pool, Yellowstone National Park
Source: Biol Direct. 2013 Apr 22;8:9. doi: 10.1186/1745-6150-8-9 (PMC3655853; doi:10.1186/1745-6150-8-9)
Supplement: Additional file 1 — Presents the GC content distribution in the first pass assembly of the Illumina reads and a principal coordinates projection of the tetranucleotide frequency distribution in those contigs. [file 1745-6150-8-9-S1.pdf]

A

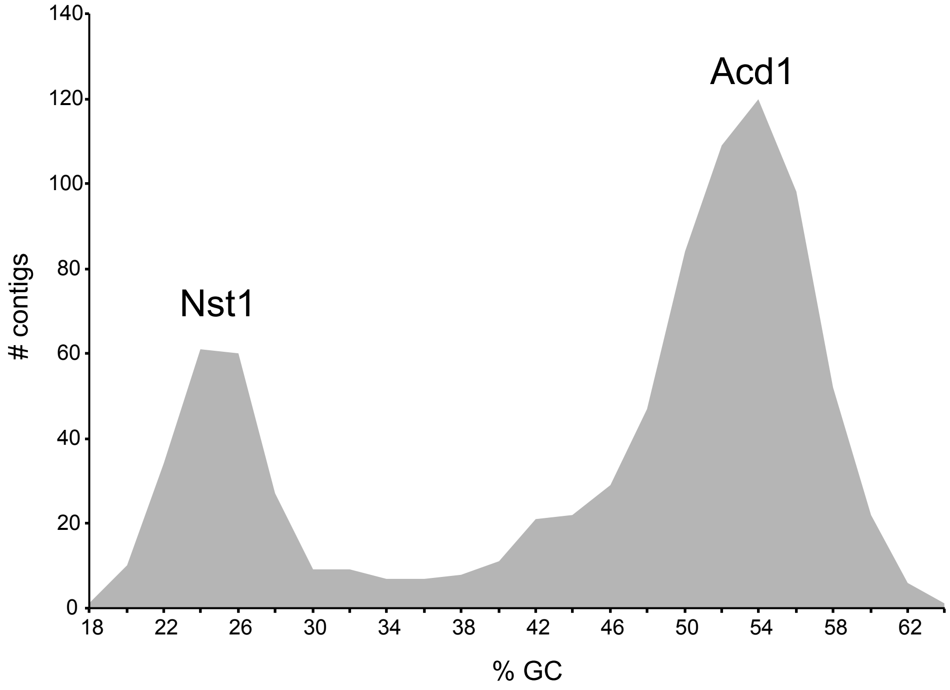

B

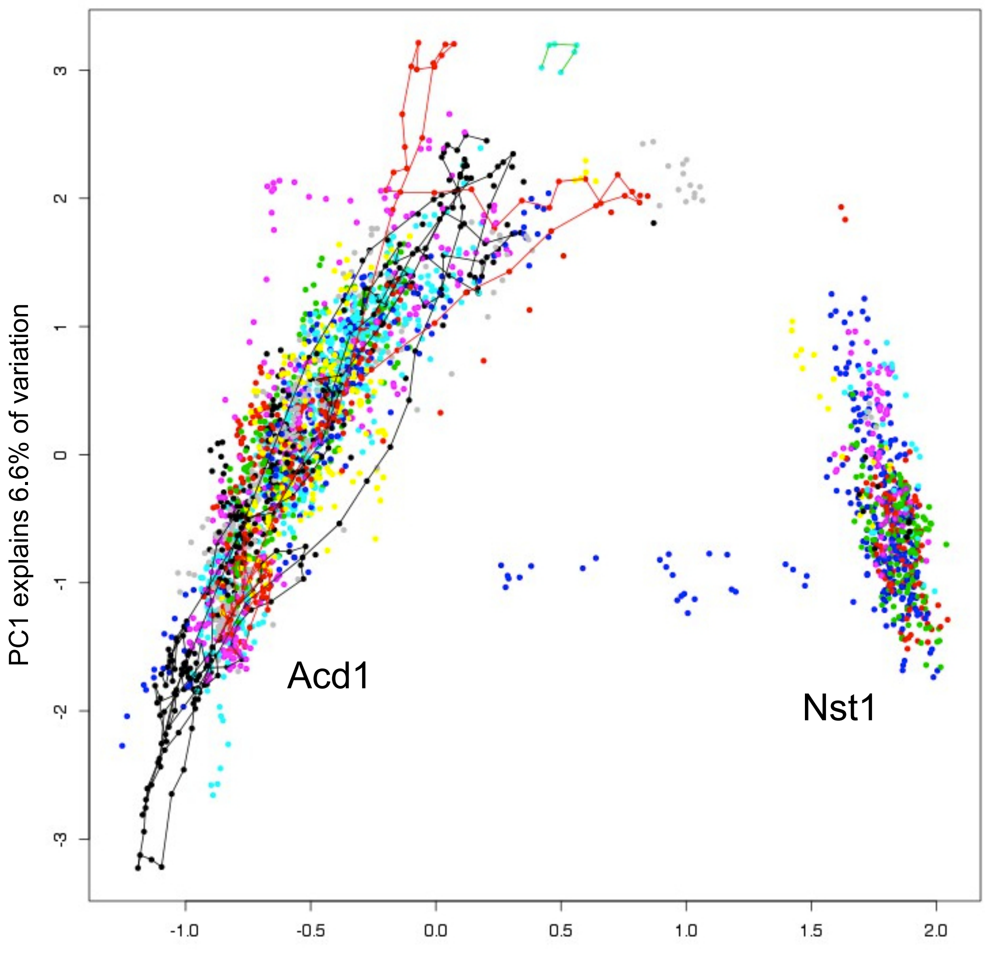

Additional file 1. Composition analysis of the raw genomic assembly of the Nst1-Acd1 SAGs based on G+C% (A) and kmer tetranucleotide frequencies (B).
