# Supplementary material for: Insights into archaeal evolution and symbiosis from the genomes of a nanoarchaeon and its inferred crenarchaeal host from Obsidian Pool, Yellowstone National Park
Source: Biol Direct. 2013 Apr 22;8:9. doi: 10.1186/1745-6150-8-9 (PMC3655853; doi:10.1186/1745-6150-8-9)
Supplement: Additional file 2 — Is a table containing gene annotation information for Nst1 and Acd1. [file 1745-6150-8-9-S2.pdf]

Additional file 2. Nst1 and Acd1 gene annotations

Nst1

Locus	Contig	arCOG	COG class	Symbol	Annotation	Best NR database hit	Blast Score	Best hit phylum	Best hit species
Nst1_001	C1	arCOG03168	R		Predicted ATPase (AAA+ superfamily)	170290072	117	Korarchaeota	Candidatus Korarchaeum cryptofilum OPF8
Nst1_002	C1	arCOG03056	I	PgpB	Membrane-associated phospholipid phosphatase	392397672	63.9	Bacteroidetes/Chlorobi group	Flexibacter litoralis DSM 6794
Nst1_003	C1				Uncharacterized protein	229579421	95.1	Crenarchaeota	Sulfolobus islandicus Y.G.57.14
Nst1_004	C1	arCOG02097	E	AroD	3-dehydroquinate dehydratase	374634112	176	Crenarchaeota	Metallosphaera yellowstonensis MK1
Nst1_005	C1				Uncharacterized protein				
Nst1_006	C1	arCOG01891	F	Tmk	Thymidylate kinase	225848089	121	Aquificae	Sulfurihydrogenibium azorense Az-Fu1
Nst1_007	C1	arCOG00543	R		Predicted metal-dependent RNase, consists of a metallo-beta-lactamase domain and an RNA-binding KH domain	41614872	525	Nanoarchaeota	Nanoarchaeum equitans Kin4-M
Nst1_008	C1	arCOG00970	O	PRE1	20S proteasome, beta subunit	242399181	156	Euryarchaeota	Thermococcus sibiricus MM 739
Nst1_009	C1	arCOG01764	K	SPT15	TATA-box binding protein (TBP), component of TFIID and TFIIIB	18977667	95.5	Euryarchaeota	Pyrococcus furiosus DSM 3638
Nst1_010	C1	arCOG00807	J	IleS	Isoleucyl-tRNA synthetase	41615024	733	Nanoarchaeota	Nanoarchaeum equitans Kin4-M
Nst1_011	C1				Uncharacterized protein				
Nst1_012	C1	arCOG02868	O	DsbG	Protein-disulfide isomerase	161529166	114	Thaumarchaeota	Nitrosopumilus maritimus SCM1
Nst1_013	C1	arCOG01561	J	TEF1	Translation elongation factor EF-1alpha (GTPase)	218884679	525	Crenarchaeota	Desulfurococcus kamchatkensis 1221n
Nst1_014	C1	arCOG01154	R		Calcineurin-like phosphoesterase superfamily protein	229579815	711	Crenarchaeota	Sulfolobus islandicus Y.G.57.14
Nst1_015	C1	arCOG00144	G	ProP	Permease of the major facilitator superfamily	70607801	732	Crenarchaeota	Sulfolobus acidocaldarius DSM 639
tRNA Ile_TAT	C1				tRNA Ile_TAT				
Nst1_016	C1	arCOG00439	L	MCM2	Predicted ATPase involved in replication control, Cdc46/Mcm family	41615073	470	Nanoarchaeota	Nanoarchaeum equitans Kin4-M
Nst1_017	C1	arCOG02261	R		Protein containing OB-fold-like and PCI domains	333909939	78.2	Euryarchaeota	Methanotorris igneus Kol 5
Nst1_018	C1	arCOG01640	J	GCD7	Translation initiation factor 2, beta subunit (eIF-2beta)/eIF-5 N-terminal domain	41615113	113	Nanoarchaeota	Nanoarchaeum equitans Kin4-M
Nst1_019	C1	arCOG04051	S		Uncharacterized conserved protein	156937176	53.5	Crenarchaeota	Ignicoccus hospitalis KIN4/I
Nst1_020	C1	arCOG04149	J	NMD3	NMD protein affecting ribosome stability and mRNA decay	41615029	77.4	Nanoarchaeota	Nanoarchaeum equitans Kin4-M
Nst1_021	C1				Uncharacterized protein	315231535	58.2	Euryarchaeota	Thermococcus barophilus MP
Nst1_022	C1	arCOG01179	J	InfA	Translation initiation factor 1 (IF-1)	18977954	105	Euryarchaeota	Pyrococcus furiosus DSM 3638
tRNA Trp_CCA	C1				tRNA Trp_CCA				
Nst1_023	C1				Uncharacterized protein				
Nst1_024	C1				Uncharacterized protein	269986439	44.3	Euryarchaeota	Candidatus Parvarchaeum acidiphilum ARMAN-4
Nst1_025	C1	arCOG00487	J	ArgS	Arginyl-tRNA synthetase	340344590	270	Thaumarchaeota	Candidatus Nitrosoarchaeum koreensis MY1
Nst1_026	C1	arCOG01758	J	RpsJ	Ribosomal protein S10	41614880	90.1	Nanoarchaeota	Nanoarchaeum equitans Kin4-M
Nst1_027	C1	arCOG01923	J	SIK1	Protein implicated in ribosomal biogenesis, Nop56p homolog	41615131	293	Nanoarchaeota	Nanoarchaeum equitans Kin4-M
Nst1_028	C1	arCOG00078	J	NOP1	Fibrillarin-like rRNA methylase	41614921	246	Nanoarchaeota	Nanoarchaeum equitans Kin4-M
Nst1_029	C1	arCOG03461	R		Uncharacterized protein containing archaeal-type C2H2 Zn-finger	337283529	47.4	Euryarchaeota	Pyrococcus yayanosii CH1
Nst1_030	C1	arCOG04130	J		Predicted RNA-binding protein	41615122	166	Nanoarchaeota	Nanoarchaeum equitans Kin4-M
Nst1_031	C1	arCOG01016	K		DNA-directed RNA polymerase, subunit F (rpoF)	296109749	64.3	Euryarchaeota	Methanocaldococcus infernus ME
Nst1_032	C1	arCOG04129	J	RPL21A	Ribosomal protein L21E	41615184	130	Nanoarchaeota	Nanoarchaeum equitans Kin4-M
Nst1_033	C1				Uncharacterized membrane protein				
Nst1_034	C1	arCOG03749	S		Uncharacterized membrane protein	325968799	85.1	Crenarchaeota	Vulcanisaeta moutnovskia 768-28
Nst1_035	C1				Uncharacterized protein				
Nst1_036	C1	arCOG10265	S		Uncharacterized conserved protein	15898780	271	Crenarchaeota	Sulfolobus solfataricus P2
Nst1_037	C1	arCOG10353	S		Uncharacterized conserved protein	15898779	191	Crenarchaeota	Sulfolobus solfataricus P2
Nst1_038	C1				Uncharacterized membrane protein	327311018	84.3	Crenarchaeota	Thermoproteus uzoniensis 768-20
Nst1_039	C1				Uncharacterized membrane protein				
Nst1_040	C1				Uncharacterized protein				
Nst1_041	C1				Uncharacterized protein				
Nst1_042	C1				Uncharacterized protein				
Nst1_043	C1				Uncharacterized protein				
Nst1_044	C1				Uncharacterized protein				
Nst1_045	C1				Uncharacterized protein				
Nst1_046	C1				Uncharacterized protein				
Nst1_047	C1				Uncharacterized membrane protein				
Nst1_048	C1	arCOG04156	J	TYW3	Wybutosine (yW) biosynthesis enzyme	41615114	126	Nanoarchaeota	Nanoarchaeum equitans Kin4-M
Nst1_049	C1				Uncharacterized protein				
Nst1_050	C1	arCOG04281	L	DnaG	DNA primase (bacterial type)	288930642	344	Euryarchaeota	Ferroglobus placidus DSM 10642
Nst1_051	C1	arCOG04180	G		Bifunctional fructose-1,6-bisphosphate aldolase/phosphatase FBPA/FBPase	284162358	464	Euryarchaeota	Archaeoglobus profundus DSM 5631
Nst1_052	C1	arCOG04287	J	RPP1A	Ribosomal protein L12E/L44/L45/RPP1/RPP2	41614996	113	Nanoarchaeota	Nanoarchaeum equitans Kin4-M
Nst1_053	C1	arCOG01159	S		Uncharacterized archaeal coiled-coil protein	41615162	138	Nanoarchaeota	Nanoarchaeum equitans Kin4-M
Nst1_054	C1	arCOG01255	J	AlaS	Alanyl-tRNA synthetase	359415684	504	Euryarchaeota	Candidatus Haloredivivus sp. G17
tRNA Leu_TAG	C1				tRNA Leu_TAG				
Nst1_055	C1				Uncharacterized protein				
Nst1_056	C1	arCOG05631	S		Uncharacterized conserved protein	171185888	218	Crenarchaeota	Pyrobaculum neutrophilum V24Sta
Nst1_057	C1	arCOG02144	L	HHT1	Histones H3 and H4	41615077	93.6	Nanoarchaeota	Nanoarchaeum equitans Kin4-M
Nst1_058	C1	arCOG00488	L	DnaN	DNA polymerase sliding clamp subunit (PCNA homolog)	41615320	213	Nanoarchaeota	Nanoarchaeum equitans Kin4-M
Nst1_059	C1				Uncharacterized membrane protein				
Nst1_060	C1	arCOG00555	R		Distinct helicase family with a unique C-terminal domain including a metal-binding cysteine cluster	289192195	649	Euryarchaeota	Methanocaldococcus sp. FS406-22
Nst1_061	C1	arCOG01347	L	CDC9	ATP-dependent DNA ligase	332157848	424	Euryarchaeota	Pyrococcus sp. NA2
tRNA Ile_GAT	C1				tRNA Ile_GAT				
Nst1_062	C1				Uncharacterized membrane protein				
Nst1_063	C1	arCOG01863	K		Predicted transcription factor, homolog of eukaryotic MBF1	41614940	97.1	Nanoarchaeota	Nanoarchaeum equitans Kin4-M
Nst1_064	C1	arCOG03119	S		DedA family membrane protein	15898947	82.4	Crenarchaeota	Sulfolobus solfataricus P2
Nst1_065	C1	arCOG00928	L		Endonuclease V homolog	312137236	172	Euryarchaeota	Methanothermus fervidus DSM 2088
Nst1_066	C1	arCOG03055	U	SecD	Preprotein translocase subunit SecD	41615222	203	Nanoarchaeota	Nanoarchaeum equitans Kin4-M
Nst1_067	C1	arCOG01894	L	Nfo	Endonuclease IV	156937880	309	Crenarchaeota	Ignicoccus hospitalis KIN4/I
Nst1_068	C1	arCOG00978	J	GCD14	tRNA(1-methyladenosine) methyltransferase	341582925	194	Euryarchaeota	Thermococcus sp. 4557
Nst1_069	C1	arCOG01997	U	MarC	Multiple antibiotic transporter	336114953	107	Firmicutes	Bacillus coagulans 2-6
Nst1_070	C1	arCOG00408	J	LysU	Lysyl-tRNA synthetase (class II)	159041752	466	Crenarchaeota	Caldivirga maquilingensis IC-167
Nst1_071	C1	arCOG00255	E	PheA	Prephenate dehydratase	332796453	168	Crenarchaeota	Acidianus hospitalis W1
Nst1_072	C1	arCOG01578	P	MgtA	Cation transport ATPase	325968004	846	Crenarchaeota	Vulcanisaeta moutnovskia 768-28
tRNA Ser_TGA	C1				tRNA Ser_TGA				
Nst1_073	C1				Uncharacterized protein				
Nst1_074	C1	arCOG00280	L		HerA helicase	15669760	151	Euryarchaeota	Methanocaldococcus jannaschii DSM 2661
Nst1_075	C1	arCOG01722	J	RpsM	Ribosomal protein S13	41615251	211	Nanoarchaeota	Nanoarchaeum equitans Kin4-M
Nst1_076	C1	arCOG04239	J	RpsD	Ribosomal protein S4 or related protein	41615041	209	Nanoarchaeota	Nanoarchaeum equitans Kin4-M
Nst1_077	C1				Uncharacterized protein				
Nst1_078	C1	arCOG00987	J	TruB	Pseudouridine synthase	41615239	441	Nanoarchaeota	Nanoarchaeum equitans Kin4-M
Nst1_079	C1	arCOG04121	L	RnhB	Ribonuclease HII	14520734	185	Euryarchaeota	Pyrococcus abyssi GE5
Nst1_080	C1	arCOG02050	R		Predicted permease	325968003	303	Crenarchaeota	Vulcanisaeta moutnovskia 768-28
Nst1_081	C1	arCOG01171	T	RAD55	RecA-superfamily ATPase implicated in signal transduction	119872031	423	Crenarchaeota	Pyrobaculum islandicum DSM 4184
Nst1_082	C1	arCOG01669	S		Uncharacterized conserved protein	15922146	82	Crenarchaeota	Sulfolobus tokodaii str. 7
Nst1_083	C1	arCOG00368	L	SbcC	ATPase involved in DNA repair, SbcC	336122538	227	Euryarchaeota	Methanothermococcus okinawensis IH1
Nst1_084	C1	arCOG00397	L	SbcD	DNA repair exonuclease, SbcD	269986422	122	Euryarchaeota	Candidatus Parvarchaeum acidiphilum ARMAN-4
Nst1_085	C1	arCOG01042	J		Exosome subunit, RNA binding protein with dsRBD fold	332158107	57	Euryarchaeota	Pyrococcus sp. NA2
Nst1_086	C1				Uncharacterized protein	269986439	46.2	Euryarchaeota	Candidatus Parvarchaeum acidiphilum ARMAN-4
Nst1_087	C1	arCOG01510	L	RFA1	Single-stranded DNA-binding replication protein A (RPA), large (70 kD) subunit or related ssDNA-binding protein	45358595	66.6	Euryarchaeota	Methanococcus maripaludis S2
Nst1_088	C1	arCOG01817	N	VirB11	Type IV secretory pathway, VirB11 component, or related ATPase involved in archaeal flagella biosynthesis	41614965	527	Nanoarchaeota	Nanoarchaeum equitans Kin4-M
Nst1_089	C1				Uncharacterized protein				
Nst1_090	C1				Uncharacterized secreted protein	359417743	46.2	Euryarchaeota	Candidatus Haloredivivus sp. G17
Nst1_091	C1				Uncharacterized protein	41615234	64.7	Nanoarchaeota	Nanoarchaeum equitans Kin4-M
Nst1_092	C1	arCOG01257	O	GroL	Chaperonin GroEL (HSP60 family)	41614938	699	Nanoarchaeota	Nanoarchaeum equitans Kin4-M
Nst1_093	C1	arCOG01038	F		Predicted nucleotide kinase (CMP/AMP kinase related)	70606278	106	Crenarchaeota	Sulfolobus acidocaldarius DSM 639
Nst1_094	C1	arCOG01688	R		Predicted transcriptional regulator, contains HTH and 4VR domain	41615238	88.6	Nanoarchaeota	Nanoarchaeum equitans Kin4-M
Nst1_095	C1				Uncharacterized protein				
Nst1_096	C1	arCOG00991	J		tRNA modification protein, contains pre-PUA and PUA domains	312137491	121	Euryarchaeota	Methanothermus fervidus DSM 2088
Nst1_097	C1	arCOG04471	U		Exosortase	289192430	56.2	Euryarchaeota	Methanocaldococcus sp. FS406-22
Nst1_098	C1				Uncharacterized protein				
Nst1_099	C1	arCOG01994	S	SpoIIM	Uncharacterized membrane protein	384177774	73.2	Firmicutes	Bacillus thuringiensis serovar finitimus YBT-020
Nst1_100	C1	arCOG04161	J	DPH5	Diphthamide biosynthesis methyltransferase	374635792	162	Euryarchaeota	Methanotorris formicicus Mc-S-70
Nst1_101	C1	arCOG04447	L		Archaeal DNA polymerase II, large subunit	269986160	889	Euryarchaeota	Candidatus Parvarchaeum acidiphilum ARMAN-4
Nst1_102	C1	arCOG00919	L		Holliday junction resolvase	333978847	52	Firmicutes	Desulfotomaculum kuznetsovii DSM 6115
Nst1_103	C1				rRNA (guanine(1405)-N(7))-methyltransferase	41615326	105	Nanoarchaeota	Nanoarchaeum equitans Kin4-M
Nst1_104	C1	arCOG01676	H	ThiF	Dinucleotide-utilizing enzyme involved in molybdopterin and thiamine biosynthesis	332796901	120	Crenarchaeota	Acidianus hospitalis W1
Nst1_105	C1	arCOG01308	O	Cdc48	ATPase of the AAA+ class , CDC48 family	41615258	913	Nanoarchaeota	Nanoarchaeum equitans Kin4-M
Nst1_106	C1	arCOG02155	R		Protein implicated in RNA metabolism, contains PRC-barrel domain	269986265	73.2	Euryarchaeota	Candidatus Parvarchaeum acidiphilum ARMAN-4
Nst1_107	C1	arCOG04243	J	RpsI	Ribosomal protein S9	327311717	143	Crenarchaeota	Thermoproteus uzoniensis 768-20
Nst1_108	C1	arCOG04242	J	RplM	Ribosomal protein L13	41615002	127	Nanoarchaeota	Nanoarchaeum equitans Kin4-M
Nst1_109	C1	arCOG00780	J	RPL18A	Ribosomal protein L18E	305662635	125	Crenarchaeota	Ignisphaera aggregans DSM 17230
Nst1_110	C1	arCOG04048	F	Dcd	Deoxycytidine deaminase	41615104	207	Nanoarchaeota	Nanoarchaeum equitans Kin4-M
Nst1_111	C1	arCOG00461	L		Uncharacterized protein related to Endonuclease III	374636071	125	Euryarchaeota	Methanotorris formicicus Mc-S-70
Nst1_112	C1	arCOG00406	J	AsnS	Aspartyl/asparaginyl-tRNA synthetase	302348258	373	Crenarchaeota	Acidilobus saccharovorans 345-15
Nst1_113	C1	arCOG01018	J	LasT	rRNA methylase	242399001	177	Euryarchaeota	Thermococcus sibiricus MM 739
Nst1_114	C1	arCOG04473	J	RPL31A	Ribosomal protein L31E	41615154	69.7	Nanoarchaeota	Nanoarchaeum equitans Kin4-M
Nst1_115	C1	arCOG01219	J	TRM1	N2,N2-dimethylguanosine tRNA methyltransferase	195953598	239	Aquificae	Hydrogenobaculum sp. Y04AAS1
Nst1_116	C1				Uncharacterized protein				
Nst1_117	C1	arCOG00872	L	MPH1	ERCC4-like helicase	41615176	528	Nanoarchaeota	Nanoarchaeum equitans Kin4-M
Nst1_118	C1	arCOG05360	S		Uncharacterized conserved protein	48477820	85.1	Euryarchaeota	Picrophilus torridus DSM 9790
Nst1_119	C1	arCOG00423	R		Predicted phosphohydrolase (DHH superfamily)	254168793	148	Euryarchaeota	Aciduliprofundum boonei T469
Nst1_120	C1	arCOG00395	K		Predicted membrane-associated trancriptional regulator	354557858	71.6	Firmicutes	Desulfitobacterium metallireducens DSM 15288

Nst1_121	C1	arCOG04032	L		Eukaryotic-type DNA primase, catalytic (small) subunit homolog, fused to HTH domain	229578659	370	Crenarchaeota	Sulfolobus islandicus Y.G.57.14
Nst1_122	C1	arCOG00312	O	AhpC	Peroxiredoxin	288819142	224	Aquificae	Hydrogenobacter thermophilus TK-6
Nst1_123	C1				Uncharacterized protein				
Nst1_124	C1	arCOG01391	M		Glycosyltransferase	350546624	94.7	Tenericutes	Mycoplasma iowae 695
Nst1_125	C1	arCOG03214	F		Thymidylate synthase-like protein fused to C-terminal DUF4346 domain and N-terminal domain homologous to N-teminal domain of tetrahydromethanopterin S-methyltransferase, subunit A	119719856	276	Crenarchaeota	Thermofilum pendens Hrk 5
Nst1_126	C1				Uncharacterized protein				
Nst1_127	C1	arCOG01770	E		Rhomboid family mebrane associated serine protease	336121537	137	Euryarchaeota	Methanothermococcus okinawensis IH1
Nst1_128	C1	arCOG02969	E	PepN	Aminopeptidase N	15920843	484	Crenarchaeota	Sulfolobus tokodaii str. 7
Nst1_129	C1	arCOG00810	J	MetG	Methionyl-tRNA synthetase	41615241	335	Nanoarchaeota	Nanoarchaeum equitans Kin4-M
Nst1_130	C1	arCOG00033	J	Trm5	Wybutosine (yW) biosynthesis enzyme, Trm5 methyltransferase	41615023	194	Nanoarchaeota	Nanoarchaeum equitans Kin4-M
Nst1_131	C1				Uncharacterized protein				
tRNA Arg_TCT	C1				tRNA Arg_TCT				
tRNA Pro_TGG	C1				tRNA Pro_TGG				
Nst1_132	C1	arCOG04070	J	RplC	Ribosomal protein L3	41615218	322	Nanoarchaeota	Nanoarchaeum equitans Kin4-M
Nst1_133	C1	arCOG04175	J	RPL20A	Ribosomal protein L20A (L18A)	296109184	65.5	Euryarchaeota	Methanocaldococcus infernus ME
Nst1_134	C1	arCOG01341	O	GIM5	Predicted prefoldin, molecular chaperone implicated in de novo protein folding	304313970	56.6	Euryarchaeota	Methanothermobacter marburgensis str. Marburg
Nst1_135	C1	arCOG04176	J	TIF6	Translation initiation factor 6 (eIF-6)	15921658	71.6	Crenarchaeota	Sulfolobus tokodaii str. 7
Nst1_136	C1	arCOG02964	N	FlaD	Putative archaeal flagellar protein D/E	336476498	86.3	Euryarchaeota	Methanosalsum zhilinae DSM 4017
Nst1_137	C1				Uncharacterized protein	307594867	40.4	Crenarchaeota	Vulcanisaeta distributa DSM 14429
Nst1_138	C1	arCOG04148	N	FlaH	Predicted ATPase involved in biogenesis of archaeal flagella	315230972	171	Euryarchaeota	Thermococcus barophilus MP
Nst1_139	C1	arCOG01817	N	VirB11	Type IV secretory pathway, VirB11 component, or related ATPase involved in archaeal flagella biosynthesis	14521686	525	Euryarchaeota	Pyrococcus abyssi GE5
Nst1_140	C1	arCOG01886	J	TyrS	Tyrosyl-tRNA synthetase	315230956	308	Euryarchaeota	Thermococcus barophilus MP
Nst1_141	C1				Uncharacterized protein				
Nst1_142	C1				Uncharacterized membrane protein				
Nst1_143	C1				Uncharacterized protein				
Nst1_144	C1				Uncharacterized protein				
Nst1_145	C1				Uncharacterized protein				
Nst1_146	C1				Uncharacterized protein				
Nst1_147	C1				Uncharacterized protein				
Nst1_148	C1	arCOG03166	R		AAA+ superfamily ATPase fused to HTH nad RecB nuclease domains	10954496	466	Euryarchaeota	Methanocaldococcus jannaschii DSM 2661
Nst1_149	C1	arCOG03521	V		Type II restriction enzyme, methylase subunit	379003658	1443	Crenarchaeota	Pyrobaculum oguniense TE7
Nst1_150	C1	arCOG00409	J	AsnS	Aspartyl/asparaginyl-tRNA synthetase	375083802	306	Euryarchaeota	Thermococcus litoralis DSM 5473
Nst1_151	C1	arCOG01580	K	Lrp	Transcriptional regulator, IclR family	119718936	129	Crenarchaeota	Thermofilum pendens Hrk 5
Nst1_152	C1				Predicted AAA family ATPase				
Nst1_153	C1				Uncharacterized membrane protein				
Nst1_154	C1				Uncharacterized membrane protein	332797585	56.2	Crenarchaeota	Acidianus hospitalis W1
Nst1_155	C1	arCOG06218	S		Uncharacterized membrane protein	255513503	89.7	Euryarchaeota	Candidatus Micrarchaeum acidiphilum ARMAN-2
Nst1_156	C1	arCOG04574	S	LemA	Uncharacterized conserved protein	255513504	208	Euryarchaeota	Candidatus Micrarchaeum acidiphilum ARMAN-2
Nst1_157	C1	arCOG03769	S		Uncharacterized conserved membrane protein	15899319	76.3	Crenarchaeota	Sulfolobus solfataricus P2
Nst1_158	C1	arCOG00420	C	GalT	Galactose-1-phosphate uridylyltransferase	288932824	92	Euryarchaeota	Ferroglobus placidus DSM 10642
Nst1_159	C1				Uncharacterized protein				
Nst1_160	C1	arCOG01663	K	RelE	Cytotoxic translational repressor of toxin-antitoxin stability system	384433983	47.8	Crenarchaeota	Sulfolobus solfataricus 98/2
Nst1_161	C1	arCOG00407	J	AsnS	Aspartyl/asparaginyl-tRNA synthetase	218883641	457	Crenarchaeota	Desulfurococcus kamchatkensis 1221n
Nst1_162	C1	arCOG01674	C	AcyP	Acylphosphatase	346224783	88.2	Bacteroidetes/Chlorobi group	Anaerophaga thermohalophila DSM 12881
Nst1_163	C1				Uncharacterized protein				
tRNA Val_GAC	C1				tRNA Val_GAC				
Nst1_164	C1				Uncharacterized protein				
Nst1_165	C1	arCOG04332	J	Ybak	Cys-tRNA(Pro)/Cys-tRNA(Cys) deacylase, ybaK family	70606921	71.2	Crenarchaeota	Sulfolobus acidocaldarius DSM 639
Nst1_166	C1	arCOG01268	K	RpoZ	DNA-directed RNA polymerase, subunit K/omega	298205310	72.4	Nanoarchaeota	Nanoarchaeum equitans Kin4-M
Nst1_167	C1	arCOG01574	J		RNase PH-related exoribonuclease	307594959	187	Crenarchaeota	Vulcanisaeta distributa DSM 14429
Nst1_168	C1				Uncharacterized protein				
Nst1_169	C1				predicted cell surface protein				
Nst1_170	C1				Uncharacterized membrane protein				
Nst1_171	C1				Uncharacterized membrane protein				
Nst1_172	C1	arCOG00557	R	Lhr	Lhr-like helicase	269987062	305	Euryarchaeota	Candidatus Parvarchaeum acidiphilum ARMAN-4
Nst1_173	C1				Uncharacterized membrane protein				
Nst1_174	C2	arCOG01527	L	TopA	Topoisomerase IA	223476951	410	Euryarchaeota	Thermococcus sp. AM4
Nst1_175	C2	arCOG04347	Q		Predicted permease fused to SAM-dependent methyltransferase	208605314	78.2		
Nst1_176	C2	arCOG01695	K		Predicted RNA-binding protein homologous to eukaryotic snRNP	290559894	121	Euryarchaeota	Candidatus Parvarchaeum acidophilus ARMAN-5
Nst1_177	C2	arCOG07442	S		Uncharacterized conserved protein	119719007	66.6	Crenarchaeota	Thermofilum pendens Hrk 5
Nst1_178	C2	arCOG00187	R		Predicted ATPase, RNase L inhibitor (RLI) homolog	41615088	531	Nanoarchaeota	Nanoarchaeum equitans Kin4-M
Nst1_179	C2	arCOG04116	R		ATPase (PilT family)	41614855	476	Nanoarchaeota	Nanoarchaeum equitans Kin4-M
Nst1_180	C2	arCOG04111	K	RPB11	DNA-directed RNA polymerase, subunit L	15922592	59.7	Crenarchaeota	Sulfolobus tokodaii str. 7
Nst1_181	C2	arCOG04455	L	HYS2	Archaeal DNA polymerase II, small subunit/DNA polymerase delta, subunit B	41615034	320	Nanoarchaeota	Nanoarchaeum equitans Kin4-M
Nst1_182	C2	arCOG00467	L	CDC6	Cdc6-related protein, AAA superfamily ATPase	385805895	176	Crenarchaeota	Fervidicoccus fontis Kam940
Nst1_183	C2	arCOG04219	R		Possible nuclease of RNase H fold, RuvC/YqgF family	385805339	113	Crenarchaeota	Fervidicoccus fontis Kam940
Nst1_184	C2	arCOG00782	J	RpsN	Ribosomal protein S14	41615021	69.7	Nanoarchaeota	Nanoarchaeum equitans Kin4-M
Nst1_185	C2	arCOG04091	J	RpsH	Ribosomal protein S8	41615066	121	Nanoarchaeota	Nanoarchaeum equitans Kin4-M
Nst1_186	C2				Uncharacterized protein	339756871	43.5	Euryarchaeota	Candidatus Nanosalinarum sp. J07AB56
Nst1_187	C2	arCOG04088	J	RplR	Ribosomal protein L18	41614871	278	Nanoarchaeota	Nanoarchaeum equitans Kin4-M
Nst1_188	C2	arCOG03167	R		Predicted ATPase (AAA+ superfamily)	288559272	255	Euryarchaeota	Methanobrevibacter ruminantium M1
Nst1_189	C2	arCOG02841	V	MdlB	ABC-type multidrug transport system, ATPase and permease component	15921353	750	Crenarchaeota	Sulfolobus tokodaii str. 7
Nst1_190	C2	arCOG02218	V		RHH/CopG DNA binding protein	124028348	48.5	Crenarchaeota	Hyperthermus butylicus DSM 5456
Nst1_191	C2	arCOG00729	V		PIN domain containing protein	385772754	84.7	Crenarchaeota	Sulfolobus islandicus HVE10/4
Nst1_0192	C2								
tRNA Ser_GGA	C2				tRNA Ser_GGA				
Nst1_193	C2	arCOG00367	L		NurA 5'-3' nuclease	124506175	48.1		
tRNA His_GTG	C2				tRNA His_GTG				
Nst1_194	C2				Uncharacterized protein	269986694	129	Euryarchaeota	Candidatus Parvarchaeum acidiphilum ARMAN-4
Nst1_195	C2	arCOG01824	N	FlaF	Putative archaeal flagellar protein F	256811149	50.1	Euryarchaeota	Methanocaldococcus fervens AG86
Nst1_196	C2	arCOG01822	N	FlaG	Putative archaeal flagellar protein G	354611354	67.8	Euryarchaeota	Halobacterium sp. DL1
Nst1_197	C2	arCOG01719	J	GatE	Archaeal Glu-tRNAGln amidotransferase subunit E (contains GAD domain)	41615038	330	Nanoarchaeota	Nanoarchaeum equitans Kin4-M
Nst1_198	C2	arCOG01728	R		Predicted dioxygenase	305663860	174	Crenarchaeota	Ignisphaera aggregans DSM 17230
Nst1_199	C2	arCOG01832	O	IbpA	Molecular chaperone (HSP20 family)	288930756	100	Euryarchaeota	Ferroglobus placidus DSM 10642
Nst1_200	C2	arCOG00352	R		Predicted GTPase	256810052	213	Euryarchaeota	Methanocaldococcus fervens AG86
Nst1_201	C2	arCOG01145	R		Icc family phosphoesterase	150400474	84.7	Euryarchaeota	Methanococcus aeolicus Nankai-3
Nst1_202	C2	arCOG03713	F	NrdA	Ribonucleotide reductase class II (contains intein)	14590272	1208	Euryarchaeota	Pyrococcus horikoshii OT3
tRNA Ala_TGC	C2				tRNA Ala_TGC				
tRNA Leu_CAA	C2				tRNA Leu_CAA				
Nst1_203	C2	arCOG01696	G		2,3-bisphosphoglycerate-independent phosphoglycerate mutase	261402895	105	Euryarchaeota	Methanocaldococcus vulcanius M7
Nst1_204	C2	arCOG04097	J	RpsC	Ribosomal protein S3	152060885	139	Nanoarchaeota	Nanoarchaeum equitans Kin4-M
Nst1_205	C2	arCOG04098	J	RplV	Ribosomal protein L22	41614997	97.1	Nanoarchaeota	Nanoarchaeum equitans Kin4-M
Nst1_206	C2				Uncharacterized protein				
Nst1_207	C2				Uncharacterized protein				
Nst1_208	C2				Uncharacterized protein				
tRNA Glu_CTC	C2				tRNA Glu_CTC				
Nst1_209	C2				Metal binding protein, Fragment of surface protease of transglutaminase family	57641827	98.6	Euryarchaeota	Thermococcus kodakarensis KOD1
Nst1_210	C2				Uncharacterized protein				
Nst1_211	C2	arCOG04089	J	RPL19A	Ribosomal protein L19E	41615168	174	Nanoarchaeota	Nanoarchaeum equitans Kin4-M
Nst1_212	C2	arCOG00779	J	RplO	Ribosomal protein L15	289597171	100	Euryarchaeota	Aciduliprofundum boonei T469
Nst1_213	C2	arCOG00404	J	HisS	Histidyl-tRNA synthetase	41614900	436	Nanoarchaeota	Nanoarchaeum equitans Kin4-M
Nst1_214	C2	arCOG00894	M	WcaA	Glycosyltransferase	170289827	280	Korarchaeota	Candidatus Korarchaeum cryptofilum OPF8
Nst1_215	C2	arCOG04126	J	RPL37A	Ribosomal protein L37E	14520858	69.3	Euryarchaeota	Pyrococcus abyssi GE5
Nst1_216	C2	arCOG04087	J	RpsE	Ribosomal protein S5	41615177	271	Nanoarchaeota	Nanoarchaeum equitans Kin4-M
Nst1_217	C2	arCOG04086	J	RpmD	Ribosomal protein L30	240104038	159	Euryarchaeota	Thermococcus gammatolerans EJ3
Nst1_218	C2	arCOG04113	J	RplP	Ribosomal protein L10AE/L16	41615235	146	Nanoarchaeota	Nanoarchaeum equitans Kin4-M
Nst1_219	C2	arCOG02900	R		Uncharacterized protein containing a von Willebrand factor type A (vWA) domain	288932694	82	Euryarchaeota	Ferroglobus placidus DSM 10642
Nst1_220	C2	arCOG00438	R		AAA family ATPase	41615187	344	Nanoarchaeota	Nanoarchaeum equitans Kin4-M
Nst1_221	C2	arCOG02293	R		HAD superfamily hydrolase	331004269	124	Firmicutes	Lachnospiraceae oral taxon 107 str. F0167
Nst1_222	C2	arCOG04112	J	DPH2	Diphthamide synthase subunit DPH2	269986418	79.3	Euryarchaeota	Candidatus Parvarchaeum acidiphilum ARMAN-4
Nst1_223	C2	arCOG00589	R		CobQ/CobB/MinD/ParA ATPase	41614915	151	Nanoarchaeota	Nanoarchaeum equitans Kin4-M
Nst1_224	C2				Uncharacterized protein				
Nst1_225	C2				Uncharacterized protein				
RNAse P RNA	C2				RNAse P RNA				
Nst1_226	C2	arCOG00402	J	ProS	Prolyl-tRNA synthetase	41615004	536	Nanoarchaeota	Nanoarchaeum equitans Kin4-M
16S_rRNA	C2				16S_rRNA				
Nst1_227	C2	arCOG01817	N	VirB11	Type IV secretory pathway, VirB11 component, or related ATPase involved in archaeal flagella biosynthesis	339757252	412	Euryarchaeota	Candidatus Nanosalinarum sp. J07AB56
Nst1_228	C2	arCOG01808	N	TadC	Flp pilus assembly protein TadC	339757248	98.6	Euryarchaeota	Candidatus Nanosalinarum sp. J07AB56
Nst1_229	C2	arCOG01808	N	TadC	Flp pilus assembly protein TadC	329765281	117	Thaumarchaeota	Candidatus Nitrosoarchaeum limnia SFB1
Nst1_230	C2	arCOG04099	J	RpsS	Ribosomal protein S19	41615264	149	Nanoarchaeota	Nanoarchaeum equitans Kin4-M
Nst1_231	C2				Uncharacterized protein				
Nst1_232	C2	arCOG00989	J	Tgt	Queuine/archaeosine tRNA-ribosyltransferase	110832762	377	Nanoarchaeota	Nanoarchaeum equitans Kin4-M
23S_rRNA	C2				23S_rRNA				
Nst1_233	C2	arCOG06624	J		Ribosomal protein L41E	374724254	44.3	Euryarchaeota	uncultured marine group II euryarchaeote
Nst1_234	C2				Uncharacterized membrane protein				
Nst1_235	C2	arCOG00419	F	Hit	HIT family hydrolase	331086790	92	Firmicutes	Lachnospiraceae bacterium 9_1_43BFAA
Nst1_236	C2	arCOG01204	R		Minimal nucleotidyltransferase fused to HEPN domain	41614845	192	Nanoarchaeota	Nanoarchaeum equitans Kin4-M
Nst1_237	C2	arCOG04304	J		Ribosomal protein L35AE/L33A	20093595	95.9	Euryarchaeota	Methanopyrus kandleri AV19
Nst1_238	C2	arCOG01150	R		Predicted ICC-like phosphoesterase	298674480	115	Euryarchaeota	Methanohalobium evestigatum Z-7303
Nst1_239	C2	arCOG00557	R	Lhr	Lhr-like helicase	298674479	361	Euryarchaeota	Methanohalobium evestigatum Z-7303

Nst1_240	C2				Uncharacterized protein	291280786	60.1		
Nst1_241	C2				Uncharacterized protein	150401409	51.6	Euryarchaeota	Methanococcus aeolicus Nankai-3
Nst1_242	C2	arCOG01575	J	Rph	Ribonuclease PH	41615042	218	Nanoarchaeota	Nanoarchaeum equitans Kin4-M
Nst1_243	C2	arCOG00678	J	RRP4	RNA-binding protein Rrp4 or related protein (contain S1 domain and KH domain)	326422490	134	Euryarchaeota	Candidatus Parvarchaeum acidophilus ARMAN-5_'5-way FS'
Nst1_244	C2	arCOG00601	R		Protein containing two CBS domains (some fused to C-terminal double-stranded RNA-binding domain of RaiA family)	11497827	140	Euryarchaeota	Archaeoglobus fulgidus DSM 4304
Nst1_245	C2				Uncharacterized membrane protein			Nanoarchaeota	Nanoarchaeum equitans Kin4-M
Nst1_246	C2				Uncharacterized membrane protein	41615012	50.4	Nanoarchaeota	Nanoarchaeum equitans Kin4-M
Nst1_247	C2				Uncharacterized membrane protein	41615012	66.6	Nanoarchaeota	Nanoarchaeum equitans Kin4-M
Nst1_248	C2	arCOG00349	C	Fer	Ferredoxin	289191932	55.8	Euryarchaeota	Methanocaldococcus sp. FS406-22
Nst1_249	C2	arCOG00349	C	Fer	Ferredoxin	158431531	68.6	Euryarchaeota	Pyrococcus furiosus DSM 3638
Nst1_250	C2	arCOG00793	R		PD-(D/E)XK superfamily nuclease	41615331	107	Nanoarchaeota	Nanoarchaeum equitans Kin4-M
Nst1_251	C2	arCOG01695	K		Predicted RNA-binding protein homologous to eukaryotic snRNP	296109018	213	Euryarchaeota	Methanocaldococcus infernus ME
Nst1_252	C2	arCOG02079	M		S-layer domain	15897592	69.7	Crenarchaeota	Sulfolobus solfataricus P2
Nst1_253	C2	arCOG00090	F	GuaA	GMP synthase - Glutamine amidotransferase domain	284161793	107	Euryarchaeota	Archaeoglobus profundus DSM 5631
Nst1_254	C2	arCOG00035	R		Predicted ATPase of PP-loop superfamily	119720014	214	Crenarchaeota	Thermofilum pendens Hrk 5
Nst1_255	C2				Uncharacterized protein				
Nst1_256	C2	arCOG01667	S		Zinc finger domain containing protein (CDGSH-type)	340345368	72.8	Thaumarchaeota	Candidatus Nitrosoarchaeum koreensis MY1
Nst1_257	C2	arCOG01559	J	FusA	Translation elongation factor G, EF-G (GTPase)	41615324	918	Nanoarchaeota	Nanoarchaeum equitans Kin4-M
Nst1_258	C2				Zn finger protein	156937280	35.4	Crenarchaeota	Ignicoccus hospitalis KIN4/I
Nst1_259	C2	arCOG01808	N	TadC	Flp pilus assembly protein TadC	91772494	120	Euryarchaeota	Methanococcoides burtonii DSM 6242
Nst1_260	C2	arCOG01817	N	VirB11	Type IV secretory pathway, VirB11 component, or related ATPase involved in archaeal flagella biosynthesis	339757252	385	Euryarchaeota	Candidatus Nanosalinarum sp. J07AB56
Nst1_261	C2				Uncharacterized protein				
Nst1_262	C2				Uncharacterized protein				
Nst1_263	C2	arCOG00546	J		mRNA degradation ribonuclease J1/J2 (metallo-beta-lactamase superfamily)	41615250	412	Nanoarchaeota	Nanoarchaeum equitans Kin4-M
Nst1_264	C2				Uncharacterized protein	290558928	119	Euryarchaeota	Candidatus Parvarchaeum acidophilus ARMAN-5
Nst1_265	C2	arCOG00405	J	GRS1	Glycyl-tRNA synthetase (class II)	41615203	427	Nanoarchaeota	Nanoarchaeum equitans Kin4-M
Nst1_266	C2	arCOG01421	G	GlgP	Glucan phosphorylase	289549001	318	Aquificae	Thermocrinis albus DSM 14484
Nst1_267	C2	arCOG04050	L	Exo	5'-3' exonuclease (including N-terminal domain of PolI)	41614884	369	Nanoarchaeota	Nanoarchaeum equitans Kin4-M
Nst1_268	C2	arCOG00729	V		PIN domain containing protein	284997231	140	Crenarchaeota	Sulfolobus islandicus L.D.8.5
Nst1_269	C2	arCOG02218	V		RHH/CopG DNA binding protein	342306582	86.7	Crenarchaeota	Sulfolobus tokodaii str. 7
Nst1_270	C2	arCOG03714	F	NrdD	ATP cone domain	327310083	293	Crenarchaeota	Thermoproteus uzoniensis 768-20
Nst1_271	C2	arCOG01254	R		Predicted metal-dependent hydrolase related to alanyl-tRNA synthetase HxxxH domain	254168839	209	Euryarchaeota	Aciduliprofundum boonei T469
Nst1_272	C2	arCOG04214	S		Uncharacterized conserved protein	375083852	122	Euryarchaeota	Thermococcus litoralis DSM 5473
Nst1_273	C2				Uncharacterized protein	148643003	38.5	Euryarchaeota	Methanobrevibacter smithii ATCC 35061
tRNA Ala_CGC	C2				tRNA Ala_CGC				
Nst1_274	C2				Uncharacterized protein				
Nst1_275	C2	arCOG04167	J	RPL14A	Ribosomal protein L14E/L6E/L27E	41614973	115	Nanoarchaeota	Nanoarchaeum equitans Kin4-M
Nst1_276	C2	arCOG04168	J	RPL34A	Ribosomal protein L34E	218884461	83.6	Crenarchaeota	Desulfurococcus kamchatkensis 1221n
Nst1_277	C2	arCOG02673	U		OxaA/SpoJ/YigC translocase/secretase, sec-independent itegration of nascent memrane proteins into membrane	145482617	44.7		
Nst1_278	C2	arCOG04169	U	SecY	Preprotein translocase subunit SecY	284162437	238	Euryarchaeota	Archaeoglobus profundus DSM 5631
Nst1_279	C2	arCOG01895	R		AP endonuclease family enzyme	41615003	89	Nanoarchaeota	Nanoarchaeum equitans Kin4-M
Nst1_280	C2				Uncharacterized protein	290559600	73.6	Euryarchaeota	Candidatus Parvarchaeum acidophilus ARMAN-5
Nst1_281	C2	arCOG00769	R	ThiJ	Putative intracellular protease/amidase	41615133	50.8	Nanoarchaeota	Nanoarchaeum equitans Kin4-M
Nst1_282	C2				Uncharacterized membrane protein				
tRNA Ser_CGA	C2				tRNA Ser_CGA				
Nst1_283	C2	arCOG01887	J	TrpS	Tryptophanyl-tRNA synthetase	41614911	507	Nanoarchaeota	Nanoarchaeum equitans Kin4-M
Nst1_284	C2	arCOG04252	J		tRNA pseudouridine synthase D	150401583	216	Euryarchaeota	Methanococcus aeolicus Nankai-3
Nst1_285	C2	arCOG04318	J		Predicted RNA-binding protein of the translin family	159041056	184	Crenarchaeota	Caldivirga maquilingensis IC-167
Nst1_286	C2	arCOG04064	M		Predicted membrane-associated Zn-dependent protease	41615327	160	Nanoarchaeota	Nanoarchaeum equitans Kin4-M
Nst1_287	C2	arCOG04049	J	RPL40A	Ribosomal protein L40E	269986484	50.8	Euryarchaeota	Candidatus Parvarchaeum acidiphilum ARMAN-4
Nst1_288	C2	arCOG01736	J	LigT	2'-5' RNA ligase	307594884	119	Crenarchaeota	Vulcanisaeta distributa DSM 14429
Nst1_289	C2	arCOG01744	R		Membrane-bound metal-dependent hydrolase	168211063	63.9	Firmicutes	Clostridium perfringens B str. ATCC 3626
Nst1_290	C2				Uncharacterized protein				
Nst1_291	C2	arCOG04228	J	pth2	Peptidyl-tRNA hydrolase	16081275	134	Euryarchaeota	Thermoplasma acidophilum DSM 1728
Nst1_292	C2				Uncharacterized protein				
Nst1_293	C2	arCOG04345	J	RPR2	RNase P subunit RPR2	390938938	80.5	Crenarchaeota	Desulfurococcus fermentans DSM 16532
5S_RNA	C2				5S_RNA				
Nst1_294	C2				Uncharacterized protein				
Nst1_295	C2				Uncharacterized protein				
Nst1_296	C2	arCOG02960	R		Predicted aminopeptidase, Iap family	41614823	152	Nanoarchaeota	Nanoarchaeum equitans Kin4-M
Nst1_297	C2	arCOG04096	J	RpsQ	Ribosomal protein S17	347524205	127	Crenarchaeota	Pyrolobus fumarii 1A
Nst1_298	C2	arCOG04095	J	RplN	Ribosomal protein L14	41614889	166	Nanoarchaeota	Nanoarchaeum equitans Kin4-M
Nst1_299	C2	arCOG04094	J	RplX	Ribosomal protein L24	41615050	126	Nanoarchaeota	Nanoarchaeum equitans Kin4-M
Nst1_300	C2	arCOG04093	J	RPS4A	Ribosomal protein S4E	41615262	169	Nanoarchaeota	Nanoarchaeum equitans Kin4-M
Nst1_301	C2	arCOG04092	J	RplE	Ribosomal protein L5	70606399	194	Crenarchaeota	Sulfolobus acidocaldarius DSM 639
Nst1_302	C2	arCOG04090	J	RplF	Ribosomal protein L6P	124028171	140	Crenarchaeota	Hyperthermus butylicus DSM 5456
Nst1_303	C2	arCOG00781	J	RPL32	Ribosomal protein L32E	20094661	129	Euryarchaeota	Methanopyrus kandleri AV19
Nst1_304	C2				Uncharacterized protein				
Nst1_305	C2				Uncharacterized protein	41615228	43.9	Nanoarchaeota	Nanoarchaeum equitans Kin4-M
Nst1_306	C2	arCOG01486	L		Ribonuclease M5 (contains TOPRIM domain)	374635331	61.2	Euryarchaeota	Methanotorris formicicus Mc-S-70
Nst1_307	C2	arCOG03013	L	PRI2	Eukaryotic-type DNA primase, large subunit	41615183	283	Nanoarchaeota	Nanoarchaeum equitans Kin4-M
Nst1_308	C2	arCOG00675	K	RPB7	DNA-directed RNA polymerase, subunit E'	333987965	171	Euryarchaeota	Methanobacterium sp. SWAN-1
Nst1_309	C2	arCOG04077	K	Spt4	Transcription elongation factor Spt4/RpoE2, zinc finger protein	41615025	79.3	Nanoarchaeota	Nanoarchaeum equitans Kin4-M
Nst1_310	C2	arCOG04302	J	GlnS	Glutamyl- or glutaminyl-tRNA synthetase	41615091	468	Nanoarchaeota	Nanoarchaeum equitans Kin4-M
Nst1_311	C2	arCOG01342	O	GimC	Prefoldin, chaperonin cofactor	14601406	76.3	Crenarchaeota	Aeropyrum pernix K1
Nst1_312	C2	arCOG02263	D		Predicted cell division protein, SepF homolog	41615068	87.8	Nanoarchaeota	Nanoarchaeum equitans Kin4-M
Nst1_313	C2	arCOG04265	R		C4-type Zn-finger protein	156937893	95.1	Crenarchaeota	Ignicoccus hospitalis KIN4/I
Nst1_314	C2				Uncharacterized protein				
Nst1_315	C2	arCOG04341	K	RPC10	DNA-directed RNA polymerase, subunit RPC10 (contains C4-type Zn-finger)	333910832	57	Euryarchaeota	Methanotorris igneus Kol 5
Nst1_316	C2	arCOG04208	J	RPL43A	Ribosomal protein L37AE/L43A	41614833	78.6	Nanoarchaeota	Nanoarchaeum equitans Kin4-M
Nst1_317	C2	arCOG01344	J	RPS19A	Ribosomal protein S19E (S16A)	161511236	148	Nanoarchaeota	Nanoarchaeum equitans Kin4-M
Nst1_318	C2				Uncharacterized protein				
Nst1_319	C2				Predicted membrane or secreted protein				
Nst1_320	C2				Uncharacterized protein				
Nst1_321	C2				Predicted membrane or secreted protein				
Nst1_322	C2	arCOG00284	L		HerA helicase	290558807	386	Euryarchaeota	Candidatus Parvarchaeum acidophilus ARMAN-5
tRNA Gly_TCC	C2				tRNA Gly_TCC				
tRNA Ala_GGC	C2				tRNA Ala_GGC				
Nst1_323	C2				Uncharacterized protein				
Nst1_324	C2	arCOG00350	R		Predicted GTPase	163785853	173	Aquificae	Hydrogenivirga sp. 128-5-R1-1
Nst1_325	C2	arCOG04142	O	DYS1	Deoxyhypusine synthase	41615189	433	Nanoarchaeota	Nanoarchaeum equitans Kin4-M
Nst1_326	C2	arCOG00810	J	MetG	Methionyl-tRNA synthetase, fragment	240103568	144	Euryarchaeota	Thermococcus gammatolerans EJ3
Nst1_327	C2	arCOG01924	E	AnsB	L-asparaginase/archaeal Glu-tRNAGln amidotransferase subunit D	341581725	323	Euryarchaeota	Thermococcus sp. 4557
Nst1_328	C2				Uncharacterized protein				
Nst1_329	C2	arCOG02201	D	FtsZ	Cell division GTPase	290559494	326	Euryarchaeota	Candidatus Parvarchaeum acidophilus ARMAN-5
Nst1_330	C2	arCOG01347	L	CDC9	ATP-dependent DNA ligase	41615291	672	Nanoarchaeota	Nanoarchaeum equitans Kin4-M
Nst1_331	C2	arCOG02037	K		Sugar-specific transcriptional regulator TrmB	41614895	202	Nanoarchaeota	Nanoarchaeum equitans Kin4-M
Nst1_332	C2	arCOG00288	C	NfnB	Nitroreductase	147921471	126	Euryarchaeota	Methanocella arvoryzae MRE50
Nst1_334	C2				Uncharacterized membrane protein				
Nst1_333	C2	arCOG00048	L		THUMP domain associated with SAM-dependent methyltransferase				
Nst1_335	C2	arCOG01989	J		Predicted Zn-ribbon RNA-binding protein with a function in translation	352681680	57.8	Crenarchaeota	Thermoproteus tenax Kra 1
Nst1_336	C2	arCOG01988	J	EFB1	Translation elongation factor EF-1beta	41615014	82	Nanoarchaeota	Nanoarchaeum equitans Kin4-M
Nst1_337	C2	arCOG01526	L		Reverse gyrase	212223477	564	Euryarchaeota	Thermococcus onnurineus NA1
Nst1_338	C2	arCOG01641	R		Predicted RNA-binding protein, contains TRAM domain	159041804	61.2	Crenarchaeota	Caldivirga maquilingensis IC-167
Nst1_339	C2	arCOG01751	J	RPL8A	Ribosomal protein L7AE	41615108	148	Nanoarchaeota	Nanoarchaeum equitans Kin4-M
Nst1_340	C2				Uncharacterized protein				
Nst1_341	C2				Uncharacterized protein				
Nst1_342	C2	arCOG01417	M	RfaG	Glycosyltransferase	238809828	61.6	Tenericutes	Mycoplasma fermentans PG18
Nst1_343	C2	arCOG01111	G	PpsA	Phosphoenolpyruvate synthase/pyruvate phosphate dikinase	20093692	787	Euryarchaeota	Methanopyrus kandleri AV19
Nst1_344	C2	arCOG01808	N	TadC	Flp pilus assembly protein TadC	288559927	92.8	Euryarchaeota	Methanobrevibacter ruminantium M1
Nst1_345	C2	arCOG04177	J		Ribosomal protein L39E				
Nst1_346	C2	arCOG04174	J	TYW3	Wybutosine (yW) biosynthesis enzyme, Fe-S oxidoreductase	41615172	364	Nanoarchaeota	Nanoarchaeum equitans Kin4-M
Nst1_347	C2				S-adenosylmethionine decarboxylase related protein				
tRNA Gln_CTG	C2				tRNA Gln_CTG				
Nst1_348	C2				Uncharacterized protein				
Nst1_349	C2	arCOG00967	J		tRNA m(1)G methyltransferase	41614801	106	Nanoarchaeota	Nanoarchaeum equitans Kin4-M
tRNA Val_CAC	C2				tRNA Val_CAC				
Nst1_350	C2	arCOG00132	G	ProP	Permease of the major facilitator superfamily	159042170	551	Crenarchaeota	Caldivirga maquilingensis IC-167
Nst1_351	C2				Uncharacterized protein				
Nst1_352	C2	arCOG00017	R		Predicted transcriptional regulator	255513363	48.5	Euryarchaeota	Candidatus Micrarchaeum acidiphilum ARMAN-2
Nst1_353	C2	arCOG01340	C		Acyl-CoA synthetase (NDP forming)	126466132	410	Crenarchaeota	Staphylothermus marinus F1
Nst1_356	C2				Uncharacterized secreted or membrane protein				
Nst1_357	C2				Uncharacterized protein				
Nst1_358	C2				Uncharacterized protein				
Nst1_359	C2				Uncharacterized membrane protein	290558928	87.4	Euryarchaeota	Candidatus Parvarchaeum acidophilus ARMAN-5
Nst1_360	C2	arCOG00561	O		Predicted membrane-bound dolichyl-phosphate-mannose-protein mannosyltransferase	70606257	370	Crenarchaeota	Sulfolobus acidocaldarius DSM 639
tRNA Arg_CCT	C2				tRNA Arg_CCT				
Nst1_361	C2				Uncharacterized secreted protein	385773946	44.7	Crenarchaeota	Sulfolobus islandicus HVE10/4
Nst1_362	C2				Uncharacterized protein	256811290	49.7	Euryarchaeota	Methanocaldococcus fervens AG86
Nst1_363	C2				Uncharacterized protein				
Nst1_364	C2				Uncharacterized protein				
Nst1_365	C2				Uncharacterized protein				

Nst1_366	C2				Uncharacterized protein				
Nst1_367	C2	arCOG00726	V		PIN domain containing protein	229582944	171	Crenarchaeota	Sulfolobus islandicus Y.N.15.51
Nst1_368	C2	arCOG02217	K		Predicted antitoxin associated with PIN domain	227829601	130	Crenarchaeota	Sulfolobus islandicus L.S.2.15
Nst1_369	C2				Uncharacterized protein				
Nst1_370	C2	arCOG01205	R		Minimal nucleotidyltransferase	119720364	94.4	Crenarchaeota	Thermofilum pendens Hrk 5
Nst1_371	C2	arCOG00196	V	CcmA	ABC-type multidrug transport system, ATPase component	385772344	525	Crenarchaeota	Sulfolobus islandicus HVE10/4
Nst1_372	C2	arCOG01467	V		ABC-type multidrug transport system, permease component	15922082	468	Crenarchaeota	Sulfolobus tokodaii str. 7
Nst1_373	C2	arCOG01943	Q	PncA	Amidase related to nicotinamidase	332797196	332	Crenarchaeota	Acidianus hospitalis W1
Nst1_374	C2	arCOG00052	G	Pgi	Glucose-6-phosphate isomerase	305663934	132	Crenarchaeota	Ignisphaera aggregans DSM 17230
Nst1_375	C2	arCOG00535	H	ThiS	Sulfur transfer protein involved in thiamine biosynthesis	291279006	55.8	Deferribacteres	Deferribacter desulfuricans SSM1
Nst1_376	C2				Uncharacterized secreted or membrane protein				
tRNA Ser_GCT	C2				tRNA Ser_GCT				
Nst1_377	C2				Uncharacterized protein				
Nst1_378	C2	arCOG01937	R		Archaeal serine protease	41614906	108	Nanoarchaeota	Nanoarchaeum equitans Kin4-M
Nst1_379	C2	arCOG00032	R		SAM-dependent methyltransferase fused to PUA domain	332158532	235	Euryarchaeota	Pyrococcus sp. NA2
Nst1_380	C2	arCOG04186	J	RPS1A	Ribosomal protein S3AE	41615164	162	Nanoarchaeota	Nanoarchaeum equitans Kin4-M
Nst1_381	C2	arCOG00809	J	LeuS	Leucyl-tRNA synthetase	119719481	797	Crenarchaeota	Thermofilum pendens Hrk 5
Nst1_382	C2				Uncharacterized protein				
tRNA Gly_GCC	C2				tRNA Gly_GCC				
Nst1_383	C2				Uncharacterized protein				
Nst1_384	C2	arCOG00501	R	ElaC	Metal-dependent hydrolase of the beta-lactamase superfamily	41614860	229	Nanoarchaeota	Nanoarchaeum equitans Kin4-M
Nst1_385	C2	arCOG01389	M		Glycosyltransferase	119719076	134	Crenarchaeota	Thermofilum pendens Hrk 5
Nst1_386	C2	arCOG01578	P	MgtA	Cation transport ATPase	153953615	358	Firmicutes	Clostridium kluyveri DSM 555
Nst1_387	C2	arCOG01087	G	TpiA	Triosephosphate isomerase	14521935	263	Euryarchaeota	Pyrococcus abyssi GE5
Nst1_388	C2	arCOG03167	R		Predicted ATPase (AAA+ superfamily)	268325204	439	environmental samples	uncultured archaeon
Nst1_389	C2	arCOG01197	V		Minimal nucleotidyltransferase	15898459	160	Crenarchaeota	Sulfolobus solfataricus P2
Nst1_390	C2	arCOG01191	V		HEPN domain containing protein	15898460	154	Crenarchaeota	Sulfolobus solfataricus P2
Nst1_391	C2	arCOG00808	J	ValS	Valyl-tRNA synthetase	12585346	426	Euryarchaeota	Pyrococcus horikoshii OT3
Nst1_392	C2				Uncharacterized membrane protein				
Nst1_393	C2	arCOG04241	K	RpoA/Rpo1	DNA-directed RNA polymerase subunit D	41615237	129	Nanoarchaeota	Nanoarchaeum equitans Kin4-M
tRNA Val_TAC	C2				tRNA Val_TAC				
Nst1_394	C2				Uncharacterized protein				
Nst1_395	C3	arCOG01702	J	SEN2	tRNA splicing endonuclease				
tRNA Thr_TGT	C3				tRNA Thr_TGT				
Nst1_396	C3	arCOG01829	N	FlaB	Archaeal flagellins	223478421	74.3	Euryarchaeota	Thermococcus sp. AM4
Nst1_397	C3	arCOG00122	J	TrmA	SAM-dependent methyltransferase related to tRNA (uracil-5-)-methyltransferase	41614849	311	Nanoarchaeota	Nanoarchaeum equitans Kin4-M
Nst1_398	C3				Uncharacterized protein				
Nst1_399	C3	arCOG01829	N	FlaB	Archaeal flagellin	14590448	67.8	Euryarchaeota	Pyrococcus horikoshii OT3
tRNA Glu_TTC	C3				tRNA Glu_TTC				
Nst1_400	C3	arCOG01715	O	sufB	Cysteine desulfurase activator SufB	383790890	399	Spirochaetes	Spirochaeta africana DSM 8902
Nst1_401	C3	arCOG01951	R		Predicted P-loop ATPase fused to an acetyltransferase	375081916	569	Euryarchaeota	Thermococcus litoralis DSM 5473
Nst1_402	C3	arCOG01526	L		Reverse gyrase	41615220	454	Nanoarchaeota	Nanoarchaeum equitans Kin4-M
Nst1_403	C3	arCOG00551	L		DNA replication initiation complex subunit, GINS15 family	341581246	66.6	Euryarchaeota	Thermococcus sp. 4557
Nst1_404	C3	arCOG04109	J	RPL42A	Ribosomal protein L44E	41614978	76.6	Nanoarchaeota	Nanoarchaeum equitans Kin4-M
Nst1_405	C3	arCOG05037	S		Uncharacterized conserved protein	41615193	48.5	Nanoarchaeota	Nanoarchaeum equitans Kin4-M
Nst1_406	C3	arCOG00348	R		Archaeal enzyme of ATP-grasp superfamily	150400664	82.4	Euryarchaeota	Methanococcus aeolicus Nankai-3
Nst1_407	C3	arCOG00906	J		Predicted Zn-ribbon RNA-binding protein	242398566	73.9	Euryarchaeota	Thermococcus sibiricus MM 739
Nst1_408	C3	arCOG04107	J	SUI2	Translation initiation factor 2, alpha subunit (eIF-2alpha)	41615191	127	Nanoarchaeota	Nanoarchaeum equitans Kin4-M
Nst1_409	C3				Uncharacterized protein				
Nst1_410	C3				Uncharacterized protein				
Nst1_411	C3				Uncharacterized secreted or membrane protein				
Nst1_412	C3	arCOG01741	J	PelA	Release factor eRF1	254168066	124	Euryarchaeota	Aciduliprofundum boonei T469
Nst1_413	C3				Uncharacterized membrane protein				
Nst1_414	C3				Uncharacterized protein				
Nst1_415	C3	arCOG00371	D	Smc	Chromosome segregation ATPase	374635895	112	Euryarchaeota	Methanotorris formicicus Mc-S-70
Nst1_416	C3								
Nst1_417	C3	arCOG00328	L	PolB	DNA polymerase elongation subunit (family B)	206890537	457	Nitrospirae	Thermodesulfovibrio yellowstonii DSM 11347
Nst1_418	C3	arCOG04246	J	RtcB	RNA 3'-P ligase, RtcB family protein	41614875	632	Nanoarchaeota	Nanoarchaeum equitans Kin4-M
Nst1_419	C3				Uncharacterized membrane protein				
Nst1_420	C3	arCOG01572	S		Uncharacterized conserved membrane protein	325968638	70.1	Crenarchaeota	Vulcanisaeta moutnovskia 768-28
tRNA Gln_TTG	C3				tRNA Gln_TTG				
Nst1_421	C3	arCOG00472	L		orc1/cdc6 family replication initiation protein fused to HTH domain	225848795	97.8	Aquificae	Sulfurihydrogenibium azorense Az-Fu1
Nst1_422	C3	arCOG00042	D	MesJ	tRNA(Ile)-lysidine synthase MesJ	41615304	273	Nanoarchaeota	Nanoarchaeum equitans Kin4-M
Nst1_423	C3				Uncharacterized protein				
Nst1_424	C3				Uncharacterized conserved membrane protein				
Nst1_425	C3	arCOG04244	K	RPB10	DNA-directed RNA polymerase, subunit N (RpoN/RPB10)	20094914	81.6	Euryarchaeota	Methanopyrus kandleri AV19
Nst1_426	C3	arCOG00357	J		Predicted GTPase, probable translation factor	41615249	551	Nanoarchaeota	Nanoarchaeum equitans Kin4-M
tRNA Met_CAT	C3				tRNA Met_CAT				
Nst1_427	C3	arCOG01753	K	Ssh10b	Archaeal DNA-binding protein	41615153	106	Nanoarchaeota	Nanoarchaeum equitans Kin4-M
Nst1_428	C3	arCOG01885	J	RPS17A	Ribosomal protein S17E	289193270	68.6	Euryarchaeota	Methanocaldococcus sp. FS406-22
Nst1_429	C3	arCOG01711	C	Ppa	Inorganic pyrophosphatase	171185208	298	Crenarchaeota	Pyrobaculum neutrophilum V24Sta
Nst1_430	C3	arCOG00973	J	Sun	tRNA or rRNA cytosine-C5-methylase	41615318	387	Nanoarchaeota	Nanoarchaeum equitans Kin4-M
tRNA Arg_TCG	C3				tRNA Arg_TCG				
Nst1_431	C3	arCOG05072	C		Aldehyde:ferredoxin oxidoreductase	150400876	118	Euryarchaeota	Methanococcus aeolicus Nankai-3
tRNA Leu_CAG	C3				tRNA Leu_CAG				
Nst1_432	C3	arCOG01039	F	AdkA	Archaeal adenylate kinase	296109296	136	Euryarchaeota	Methanocaldococcus infernus ME
tRNA Leu_GAG	C3				tRNA Leu_GAG				
Nst1_433	C3				Uncharacterized membrane protein				
Nst1_434	C3	arCOG00486	J	CysS	Cysteinyl-tRNA synthetase	41614851	519	Nanoarchaeota	Nanoarchaeum equitans Kin4-M
Nst1_435	C3	arCOG01563	J	GCD11	Translation initiation factor 2, gamma subunit (eIF-2gamma; GTPase)	41615062	419	Nanoarchaeota	Nanoarchaeum equitans Kin4-M
Nst1_436	C3	arCOG04312	R		PIN domain containing protein	359415611	50.1	Euryarchaeota	Candidatus Haloredivivus sp. G17
Nst1_437	C3	arCOG00358	R	DRG	Predicted GTPase	41614908	137	Nanoarchaeota	Nanoarchaeum equitans Kin4-M
Nst1_438	C3				Uncharacterized protein				
Nst1_439	C3	arCOG00985	J		Predicted RNA-binding protein (contains PUA domain)	41615000	100	Nanoarchaeota	Nanoarchaeum equitans Kin4-M
Nst1_440	C3	arCOG04269	S		Protein, predicted to be involved in DNA repair	327400273	209	Euryarchaeota	Archaeoglobus veneficus SNP6
Nst1_441	C3	arCOG04112	J	DPH2	Diphthamide synthase subunit DPH2	312137233	101	Euryarchaeota	Methanothermus fervidus DSM 2088
Nst1_442	C3	arCOG01814	N		Type II secretion system protein F	269986816	238	Euryarchaeota	Candidatus Parvarchaeum acidiphilum ARMAN-4
Nst1_443	C3	arCOG01818	N	VirB11	Type IV secretory pathway, VirB11 component, or related ATPase involved in archaeal flagella biosynthesis	41615211	568	Nanoarchaeota	Nanoarchaeum equitans Kin4-M
Nst1_444	C3				Uncharacterized protein	339757139	60.5	Euryarchaeota	Candidatus Nanosalinarum sp. J07AB56
Nst1_445	C3				Uncharacterized protein	41615268	87.4	Nanoarchaeota	Nanoarchaeum equitans Kin4-M
Nst1_446	C3				Uncharacterized protein	336121711	45.1	Euryarchaeota	Methanothermococcus okinawensis IH1
Nst1_447	C3	arCOG00891	L	TatD	Mg-dependent DNase	159041873	224	Crenarchaeota	Caldivirga maquilingensis IC-167
Nst1_448	C3	arCOG04270	K	TFA1	Transcription initiation factor IIE, alpha subunit	332796493	84.7	Crenarchaeota	Acidianus hospitalis W1
Nst1_449	C3	arCOG01719	J	GatE	Archaeal Glu-tRNAGln amidotransferase subunit E (contains GAD domain)	332797065	188	Crenarchaeota	Acidianus hospitalis W1
Nst1_450	C3	arCOG00419	F	Hit	HIT family hydrolase	41615301	139	Nanoarchaeota	Nanoarchaeum equitans Kin4-M
Nst1_451	C3	arCOG00417	L	RecA	RecA/RadA recombinase	312137146	198	Euryarchaeota	Methanothermus fervidus DSM 2088
Nst1_452	C3	arCOG04249	J	CCA1	tRNA nucleotidyltransferase (CCA-adding enzyme)	41614948	228	Nanoarchaeota	Nanoarchaeum equitans Kin4-M
Nst1_453	C3	arCOG01742	J	eRF1	Peptide chain release factor 1 (eRF1)	41614848	350	Nanoarchaeota	Nanoarchaeum equitans Kin4-M
Nst1_454	C3	arCOG01180	T	RIO1	Serine/threonine protein kinase involved in cell cycle control	312136522	160	Euryarchaeota	Methanothermus fervidus DSM 2088
Nst1_455	C3				Uncharacterized protein				
Nst1_456	C3				Uncharacterized protein				
Nst1_457	C3	arCOG04245	J	RpsB	Ribosomal protein S2	41615290	224	Nanoarchaeota	Nanoarchaeum equitans Kin4-M
Nst1_458	C3	arCOG04067	J	RplB	Ribosomal protein L2	41615150	200	Nanoarchaeota	Nanoarchaeum equitans Kin4-M
Nst1_459	C3	arCOG04072	J	RplW	Ribosomal protein L23	156937757	94.4	Crenarchaeota	Ignicoccus hospitalis KIN4/I
Nst1_460	C3	arCOG04071	J	RplD	Ribosomal protein L4	41614941	210	Nanoarchaeota	Nanoarchaeum equitans Kin4-M
Nst1_461	C3	arCOG01809	N	FlaJ	Archaeal flagella assembly protein J	296109771	219	Euryarchaeota	Methanocaldococcus infernus ME
Nst1_462	C3	arCOG02285	J		Ribosome biogenesis protein, NOL1/NOP2/fmu family	41615099	63.5	Nanoarchaeota	Nanoarchaeum equitans Kin4-M
Nst1_463	C3	arCOG00470	L	HolB	ATPase involved in DNA replication HolB, large subunit	41615216	367	Nanoarchaeota	Nanoarchaeum equitans Kin4-M
Nst1_464	C3				Uncharacterized protein				
Nst1_465	C3				Uncharacterized protein				
Nst1_466	C3				Uncharacterized protein				
Nst1_467	C3	arCOG05419	S		Uncharacterized conserved protein	170290400	41.6	Korarchaeota	Candidatus Korarchaeum cryptofilum OPF8
Nst1_468	C3				Uncharacterized protein				
Nst1_469	C3	arCOG00469	L	HolB	ATPase involved in DNA replication HolB, small subunit	41614964	411	Nanoarchaeota	Nanoarchaeum equitans Kin4-M
Nst1_470	C3	arCOG00109	J	HemK	Methylase of polypeptide chain release factors	41615032	111	Nanoarchaeota	Nanoarchaeum equitans Kin4-M
Nst1_471	C3	arCOG00891	L	TatD	Mg-dependent DNase	41615242	189	Nanoarchaeota	Nanoarchaeum equitans Kin4-M
Nst1_472	C3	arCOG02207	L	XthA	Exonuclease III	332797757	328	Crenarchaeota	Acidianus hospitalis W1
Nst1_473	C3	arCOG01252	C	PutA	Lactaldehyde dehydrogenase, Succinate semialdehyde dehydrogenase or other NAD-dependent aldehyde dehydrogenase	159041549	619	Crenarchaeota	Caldivirga maquilingensis IC-167
Nst1_474	C3	arCOG04253	S		Uncharacterized conserved protein	315425508	107	Thaumarchaeota	Candidatus Caldiarchaeum subterraneum
tRNA Val_TAC	C3				tRNA Val_TAC				
Nst1_475	C3	arCOG04241	K	RpoA/Rpo1	DNA-directed RNA polymerase subunit D	223477958	110	Euryarchaeota	Thermococcus sp. AM4
Nst1_476	C3	arCOG01845	R	PaaD	Predicted metal-sulfur cluster biosynthetic enzyme	41615140	96.3	Nanoarchaeota	Nanoarchaeum equitans Kin4-M
Nst1_477	C3	arCOG01296	O	TrxB	Thioredoxin reductase	41615274	324	Nanoarchaeota	Nanoarchaeum equitans Kin4-M
Nst1_478	C3				Uncharacterized protein				
Nst1_479	C3	arCOG01218	O	TrxA	Thiol-disulfide isomerase or thioredoxin	212223465	146	Euryarchaeota	Thermococcus onnurineus NA1
Nst1_480	C3	arCOG04253	S		Uncharacterized conserved protein	254166902	124	Euryarchaeota	Aciduliprofundum boonei T469
Nst1_481	C4	arCOG01397	M	WcaA	Glycosyltransferase	332795758	98.2	Crenarchaeota	Acidianus hospitalis W1
Nst1_482	C4	arCOG01367	M	RfbD	dTDP-4-dehydrorhamnose reductase	284997404	453	Crenarchaeota	Sulfolobus islandicus L.D.8.5
Nst1_483	C4	arCOG04188	M	RfbC	dTDP-4-dehydrorhamnose 3,5-epimerase or related enzyme	284997405	300	Crenarchaeota	Sulfolobus islandicus L.D.8.5
Nst1_484	C4	arCOG00663	M	GCD1	Nucleoside-diphosphate-sugar pyrophosphorylase involved in lipopolysaccharide biosynthesis/translation initiation factor 2B, gamma/epsilon subunit	307595587	306	Crenarchaeota	Vulcanisaeta distributa DSM 14429
Nst1_485	C4				Uncharacterized membrane protein	332797585	69.3	Crenarchaeota	Acidianus hospitalis W1
Nst1_486	C4				Uncharacterized protein				
Nst1_487	C4				Uncharacterized protein				
Nst1_488	C4				Uncharacterized protein				

Nst1_489	C4	arCOG03167	R		Predicted ATPase (AAA+ superfamily)	14521104	395	Euryarchaeota	Pyrococcus abyssi GE5
Nst1_490	C4				Uncharacterized protein				
Nst1_491	C4	arCOG01573	M	MscS	Small-conductance mechanosensitive channel	389861581	240	Crenarchaeota	Thermogladius cellulolyticus 1633
Nst1_492	C4	arCOG07455	S		Uncharacterized conserved protein	229582570	228	Crenarchaeota	Sulfolobus islandicus Y.N.15.51
Nst1_493	C4				Secredted protein (TAT signal)				
Nst1_494	C4	arCOG00921	K		Predicted transcriptional regulator	218883350	190	Crenarchaeota	Desulfurococcus kamchatkensis 1221n
Nst1_495	C4	arCOG03869	S		Uncharacterized membrane protein	332796630	70.5	Crenarchaeota	Acidianus hospitalis W1
Nst1_496	C4	arCOG05276	L		Pyrimidine dimer DNA glycosylase, T4 endoV family	332797517	233	Crenarchaeota	Acidianus hospitalis W1
Nst1_497	C4	arCOG01606	C	PorA	Pyruvate:ferredoxin oxidoreductase or related 2-oxoacid:ferredoxin oxidoreductase, alpha subunit and gamma	319790349	202	Aquificae	Thermovibrio ammonificans HB-1
Nst1_498	C4	arCOG01358	J	MiaB	2-methylthioadenine synthetase	41614804	361	Nanoarchaeota	Nanoarchaeum equitans Kin4-M
Nst1_499	C4				Uncharacterized protein				
Nst1_500	C4	arCOG03698	S		Uncharacterized conserved protein	15899033	91.7	Crenarchaeota	Sulfolobus solfataricus P2
Nst1_501	C4	arCOG01993	G	GpmA	Phosphoglycerate mutase 1	269986713	263	Euryarchaeota	Candidatus Parvarchaeum acidiphilum ARMAN-4
tRNA Cys_GCA	C4				tRNA Cys_GCA				
Nst1_502	C4	arCOG00403	J	SerS	Seryl-tRNA synthetase	347524046	365	Crenarchaeota	Pyrolobus fumarii 1A
Nst1_503	C4	arCOG04305	J	RPS26B	Ribosomal protein S26	41614839	39.7	Nanoarchaeota	Nanoarchaeum equitans Kin4-M
Nst1_504	C4	arCOG00412	J	PheT	Phenylalanyl-tRNA synthetase beta subunit	14590543	422	Euryarchaeota	Pyrococcus horikoshii OT3
Nst1_505	C4	arCOG00410	J	PheS	Phenylalanyl-tRNA synthetase alpha subunit	315230169	311	Euryarchaeota	Thermococcus barophilus MP
Nst1_506	C4	arCOG01204	R		Minimal nucleotidyltransferase fused to HEPN domain	41615152	131	Nanoarchaeota	Nanoarchaeum equitans Kin4-M
Nst1_507	C4	arCOG01185	T		Mn2+-dependent serine/threonine protein kinase	242399519	158	Euryarchaeota	Thermococcus sibiricus MM 739
Nst1_508	C4				Zn finger protein	333987088	41.6	Euryarchaeota	Methanobacterium sp. SWAN-1
Nst1_509	C4	arCOG00307	J	RPP1	RNase P/RNase MRP subunit p30	400404363	55.8	Proteobacteria	Candidatus Carsonella ruddii HC isolate Thao2000
Nst1_510	C4				Uncharacterized protein	290558928	137	Euryarchaeota	Candidatus Parvarchaeum acidophilus ARMAN-5
Nst1_511	C4				Uncharacterized protein				
Nst1_512	C4	arCOG03054	U	SecF	Preprotein translocase subunit SecF	269986958	122	Euryarchaeota	Candidatus Parvarchaeum acidiphilum ARMAN-4
Nst1_513	C4	arCOG04055	R		SHS2 domain protein implicated in nucleic acid metabolism	18977924	109	Euryarchaeota	Pyrococcus furiosus DSM 3638
Nst1_514	C4	arCOG04702	R		Phospholipid-binding protein	297526620	143	Crenarchaeota	Staphylothermus hellenicus DSM 12710
Nst1_515	C4	arCOG04185	J	RpsO	Ribosomal protein S15P	327310281	103	Crenarchaeota	Thermoproteus uzoniensis 768-20
Nst1_516	C4	arCOG00427	L	RecJ/Cdc45	Single-stranded DNA-specific exonuclease RecJ	148643253	118	Euryarchaeota	Methanobrevibacter smithii ATCC 35061
Nst1_517	C4	arCOG00398	S		ASCH domain, predicted RNA-binding domain	375082335	172	Euryarchaeota	Thermococcus litoralis DSM 5473
Nst1_518	C4	arCOG06946	S		Uncharacterized conserved membrane protein	255513672	133	Euryarchaeota	Candidatus Micrarchaeum acidiphilum ARMAN-2
Nst1_519	C4				Uncharacterized conserved membrane protein	41615124	52	Nanoarchaeota	Nanoarchaeum equitans Kin4-M
tRNA Thr_CGT	C4				tRNA Thr_CGT				
Nst1_520	C4	arCOG00424	L		DHH superfamily phosphohydrolase/exonuclease	41614913	206	Nanoarchaeota	Nanoarchaeum equitans Kin4-M
Nst1_521	C4				Uncharacterized protein				
Nst1_522	C5	arCOG01159	S		Uncharacterized archaeal coiled-coil protein	82595912	57.4		
Nst1_523	C5	arCOG01411	M	RfaG	Glycosyltransferase	284997423	569	Crenarchaeota	Sulfolobus islandicus L.D.8.5
Nst1_524	C5	arCOG07390	M	RfaG	Glycosyltransferase	227827286	236	Crenarchaeota	Sulfolobus islandicus M.14.25
Nst1_525	C5	arCOG01411	M	RfaG	Glycosyltransferase	41614989	472	Nanoarchaeota	Nanoarchaeum equitans Kin4-M
Nst1_526	C5	arCOG01400	Q		SAM-dependent methyltransferase	159042031	222	Crenarchaeota	Caldivirga maquilingensis IC-167
tRNA Lys_TTT	C5				tRNA Lys_TTT				
Nst1_527	C5	arCOG00767	G	{ManB}	Phosphomannomutase	18312164	261	Crenarchaeota	Pyrobaculum aerophilum str. IM2
Nst1_528	C5	arCOG04120	G	PykF	Pyruvate kinase	332797933	556	Crenarchaeota	Acidianus hospitalis W1
tRNA Tyr_GTA	C5				tRNA Tyr_GTA				
Nst1_529	C5	arCOG04069	R		RNA methyltransferase, SPOUT superfamily	18978198	134	Euryarchaeota	Pyrococcus furiosus DSM 3638
Nst1_530	C5	arCOG01338	C		Acyl-CoA synthetase, ATP-grasp containing subunit	399154774	107	Proteobacteria	gamma proteobacterium SCGC AAA007-O20
Nst1_531	C5				Uncharacterized protein				
Nst1_532	C5	arCOG01400	Q		SAM-dependent methyltransferase	159042031	218	Crenarchaeota	Caldivirga maquilingensis IC-167
Nst1_533	C5	arCOG05398	M		Glycosyltransferase	48477373	107	Euryarchaeota	Picrophilus torridus DSM 9790
Nst1_534	C5	arCOG06139	V		HEPN domain containing protein				
Nst1_535	C5	arCOG01107	E	ArgE	Acetylornithine deacetylase/Succinyl-diaminopimelate desuccinylase or related deacylase	332795886	465	Crenarchaeota	Acidianus hospitalis W1
Nst1_536	C5	arCOG04048	F	Dcd	Deoxycytidine deaminase	41615118	204	Nanoarchaeota	Nanoarchaeum equitans Kin4-M
Nst1_537	C5				Membrane protein				
Nst1_538	C5	arCOG01915	O	HflC	Membrane protease subunit, stomatin/prohibitin homolog	284161351	182	Euryarchaeota	Archaeoglobus profundus DSM 5631
Nst1_539	C5				Uncharacterized protein				
Nst1_540	C5	arCOG04179	R		DNA-binding protein	41614945	64.3	Nanoarchaeota	Nanoarchaeum equitans Kin4-M
Nst1_541	C5	arCOG04143	L		DNA topoisomerase VI, subunit A	41615325	487	Nanoarchaeota	Nanoarchaeum equitans Kin4-M
Nst1_542	C5	arCOG01165	L		DNA topoisomerase VI, subunit B	41614939	447	Nanoarchaeota	Nanoarchaeum equitans Kin4-M
Nst1_543	C5	arCOG04150	R		Predicted RNA-binding protein (contains KH domains)	305663515	94	Crenarchaeota	Ignisphaera aggregans DSM 17230
Nst1_544	C5	arCOG01599	C	PorB	Pyruvate:ferredoxin oxidoreductase or related 2-oxoacid:ferredoxin oxidoreductase, beta subunit	119720360	273	Crenarchaeota	Thermofilum pendens Hrk 5
Nst1_545	C5				Uncharacterized membrane protein	290559052	75.5	Euryarchaeota	Candidatus Parvarchaeum acidophilus ARMAN-5
Nst1_546	C5	arCOG04761	I	UppP	Undecaprenyl pyrophosphate phosphatase	15922126	318	Crenarchaeota	Sulfolobus tokodaii str. 7
Nst1_547	C5				Uncharacterized membrane protein				
Nst1_548	C5	arCOG00579	K	RPB9	DNA-directed RNA polymerase, subunit M/Transcription elongation factor TFIIS	57640468	113	Euryarchaeota	Thermococcus kodakarensis KOD1
Nst1_549	C5	arCOG01739	U	LepB	Signal peptidase I	290559188	74.7	Euryarchaeota	Candidatus Parvarchaeum acidophilus ARMAN-5
Nst1_550	C5	arCOG05412	G	BglB	Beta-glucosidase/6-phospho-beta-glucosidase/beta-galactosidase	254168937	210	Euryarchaeota	Aciduliprofundum boonei T469
Nst1_551	C5	arCOG00324	R	AcuC	Deacetylase, including yeast histone deacetylase and acetoin utilization protein	41615319	200	Nanoarchaeota	Nanoarchaeum equitans Kin4-M
Nst1_552	C5	arCOG00038	J	ThiI	tRNA S(4)U 4-thiouridine synthase	41615210	252	Nanoarchaeota	Nanoarchaeum equitans Kin4-M
Nst1_553	C5				Uncharacterized protein				
Nst1_554	C5	arCOG01263	R	DltE	Short-chain dehydrogenase	390960765	65.9	Euryarchaeota	Thermococcus sp. CL1
Nst1_555	C5	arCOG00969	R		Predicted hydrolase (metallo-beta-lactamase superfamily)	126465143	191	Crenarchaeota	Staphylothermus marinus F1
tRNA Asn_GTT	C5				tRNA Asn_GTT				
Nst1_556	C5				Uncharacterized protein				
Nst1_557	C5	arCOG04144	L		8-oxoguanine DNA glycosylase	41615297	169	Nanoarchaeota	Nanoarchaeum equitans Kin4-M
Nst1_558	C5	arCOG01224	S		Uncharacterized conserved protein	269986760	53.1	Euryarchaeota	Candidatus Parvarchaeum acidiphilum ARMAN-4
Nst1_559	C5	arCOG01169	G	Eno	Enolase	284161315	274	Euryarchaeota	Archaeoglobus profundus DSM 5631
Nst1_560	C5	arCOG03871	N		Flagellar protein G	15921688	55.5	Crenarchaeota	Sulfolobus tokodaii str. 7
Nst1_561	C5	arCOG00784	J	POP4	RNase P/RNase MRP subunit p29	261402348	70.5	Euryarchaeota	Methanocaldococcus vulcanius M7
Nst1_562	C5	arCOG04223	J	SUI1	Translation initiation factor 1 (eIF-1/SUI1)	18978189	94.7	Euryarchaeota	Pyrococcus furiosus DSM 3638
Nst1_563	C5	arCOG00785	J		Rbosomal protein L29				
Nst1_564	C5	arCOG01183	L	Kae1/QRI7	Subunit of KEOPS complex, contains a domain with ASKHA fold and RIO-type kinase (AP-endonuclease activity)	41615276	248	Nanoarchaeota	Nanoarchaeum equitans Kin4-M
Nst1_565	C5	arCOG01946	J	RPS6A	Ribosomal protein S6E (S10)	41614902	143	Nanoarchaeota	Nanoarchaeum equitans Kin4-M
Nst1_566	C5				Uncharacterized protein				
Nst1_567	C5	arCOG01560	J	InfB	Translation initiation factor 2 (IF-2; GTPase)	242398523	580	Euryarchaeota	Thermococcus sibiricus MM 739
Nst1_568	C5	arCOG00663	M	GCD1	Nucleoside-diphosphate-sugar pyrophosphorylase involved in lipopolysaccharide biosynthesis/translation initiation factor 2B, gamma/epsilon subunit	41614821	259	Nanoarchaeota	Nanoarchaeum equitans Kin4-M
Nst1_569	C5	arCOG06957	S		Uncharacterized secreted protein	333986509	127	Euryarchaeota	Methanobacterium sp. SWAN-1
tRNA Lys_TTT	C5				tRNA Lys_TTT				
Nst1_570	C5				Uncharacterized protein				
Nst1_571	C5	arCOG01808	N	TadC	Flp pilus assembly protein TadC	41615060	151	Nanoarchaeota	Nanoarchaeum equitans Kin4-M
Nst1_572	C5	arCOG01808	N	TadC	Flp pilus assembly protein TadC	41615061	171	Nanoarchaeota	Nanoarchaeum equitans Kin4-M
Nst1_573	C5				Uncharacterized protein	296109472	45.4	Euryarchaeota	Methanocaldococcus infernus ME
Nst1_574	C5	arCOG04313	F	Ndk	Nucleoside diphosphate kinase	352681785	219	Crenarchaeota	Thermoproteus tenax Kra 1
Nst1_575	C5	arCOG01950	J	RPL24A	Ribosomal protein L24E	296243107	87	Crenarchaeota	Thermosphaera aggregans DSM 11486
Nst1_576	C5	arCOG04314	J	RPS28A	Ribosomal protein S28E/S33	41615149	98.6	Nanoarchaeota	Nanoarchaeum equitans Kin4-M
Nst1_577	C5	arCOG02044	R		Uncharacterized membrane protein, required for N-linked glycosylation	41614951	172	Nanoarchaeota	Nanoarchaeum equitans Kin4-M
Nst1_578	C5	arCOG03199	M	Rfe	UDP-N-acetylmuramyl pentapeptide phosphotransferase/UDP-N-acetylglucosamine-1-phosphate transferase	297526643	341	Crenarchaeota	Staphylothermus hellenicus DSM 12710
Nst1_579	C5	arCOG02209	R	RfbX	Polysaccharide biosynthesis protein, Mvin family	41614887	99	Nanoarchaeota	Nanoarchaeum equitans Kin4-M
Nst1_580	C5	arCOG00014	G	RbsK	Sugar kinase, ribokinase family			Firmicutes	Lactobacillus gasseri ATCC 33323
tRNA Asp_GTC	C5				tRNA Asp_GTC				
Nst1_581	C5	arCOG01182	T		Predicted Ser/Thr protein kinase	327400500	79.3	Euryarchaeota	Archaeoglobus veneficus SNP6
Nst1_582	C5	arCOG00401	J	ThrS	Threonyl-tRNA synthetase	156937646	432	Crenarchaeota	Ignicoccus hospitalis KIN4/I
Nst1_583	C6	arCOG02079	M		S-layer domain	253682208	88.6	Firmicutes	Clostridium botulinum D str. 1873
Nst1_584	C6	arCOG02160	O	LonB	Predicted ATP-dependent protease	332158725	690	Euryarchaeota	Pyrococcus sp. NA2
Nst1_585	C6				Uncharacterized membrane protein				
Nst1_586	C6	arCOG01981	K	SUA7	Transcription initiation factor TFIIIB, Brf1 subunit/Transcription initiation factor TFIIB	289192865	263	Euryarchaeota	Methanocaldococcus sp. FS406-22
Nst1_587	C6	arCOG01365	J	POP5	RNase P/RNase MRP subunit POP5				
Nst1_588	C6	arCOG00971	O	PRE1	20S proteasome, alpha subunit	13541135	195	Euryarchaeota	Thermoplasma volcanium GSS1
Nst1_589	C6	arCOG04187	J		Predicted exosome subunit	288930695	162	Euryarchaeota	Ferroglobus placidus DSM 10642
Nst1_590	C6				Uncharacterized membrane protein				
Nst1_591	C6	arCOG04922	M	RfaG	Glycosyltransferase	290559635	304	Euryarchaeota	Candidatus Parvarchaeum acidophilus ARMAN-5
Nst1_592	C6	arCOG00589	R		CobQ/CobB/MinD/ParA ATPase	41614915	65.5	Nanoarchaeota	Nanoarchaeum equitans Kin4-M
Nst1_593	C6				Uncharacterized protein				
Nst1_594	C6	arCOG03278	G		Glycosyl hydrolase family 57	333911592	376	Euryarchaeota	Methanotorris igneus Kol 5
Nst1_595	C6	arCOG01410	M	RfaG	Glycosyltransferase	311745690	239	Bacteroidetes/Chlorobi group	Algoriphagus sp. PR1
Nst1_596	C6	arCOG03287	G	GDB1	Glycogen debranching enzyme	48478310	112	Euryarchaeota	Picrophilus torridus DSM 9790
Nst1_597	C6	arCOG03167	R		Predicted ATPase (AAA+ superfamily) fused to ArsR family HTH domain	149195221	65.9	Proteobacteria	Caminibacter mediatlanticus TB-2
tRNA Thr_GGT	C6				tRNA Thr_GGT				
Nst1_598	C6	arCOG03962	S		Uncharacterized protein with SCP/PR1 domains	255513899	163	Euryarchaeota	Candidatus Micrarchaeum acidiphilum ARMAN-2
Nst1_599	C6	arCOG00385	S		Uncharacterized conserved protein				
Nst1_600	C6	arCOG03580	O	STE14	Putative protein-S-isoprenylcysteine methyltransferase	374632714	100	Crenarchaeota	Metallosphaera yellowstonensis MK1
tRNA Pro_GGG	C6				tRNA Pro_GGG				
Nst1_601	C6	arCOG03672	E		Thermopsin-like protease	307595469	244	Crenarchaeota	Vulcanisaeta distributa DSM 14429
Nst1_602	C6	arCOG01239	J	RsmE	RNA base methyltransferase family enzyme	256811269	115	Euryarchaeota	Methanocaldococcus fervens AG86
Nst1_603	C6	arCOG04409	R		Predicted nuclease (RNAse H fold)	333988293	69.7	Euryarchaeota	Methanobacterium sp. SWAN-1
tRNA Arg_GCG					tRNA Arg_GCG				
Nst1_604	C7				Uncharacterized protein				
Nst1_605	C7	arCOG04147	P	SodA	Superoxide dismutase	333979524	99.4	Firmicutes	Desulfotomaculum kuznetsovii DSM 6115
Nst1_606	C7	arCOG02144	L	HHT1	Histones H3 and H4	41615138	94	Nanoarchaeota	Nanoarchaeum equitans Kin4-M
Nst1_607	C7	arCOG01001	J	Map	Methionine aminopeptidase	320101419	272	Crenarchaeota	Desulfurococcus mucosus DSM 2162

Nst1_608	C7	arCOG04231	P	CutA	Uncharacterized protein involved in tolerance to divalent cations	302348629	61.2	Crenarchaeota	Acidilobus saccharovorans 345-15
Nst1_609	C7	arCOG04449	J	TruA	Pseudouridylate synthase	41615123	120	Nanoarchaeota	Nanoarchaeum equitans Kin4-M
Nst1_610	C7	arCOG04291	S		Uncharacterized conserved protein	315230891	216	Euryarchaeota	Thermococcus barophilus MP
Nst1_611	C7				Uncharacterized conserved protein	290558928	131	Euryarchaeota	Candidatus Parvarchaeum acidophilus ARMAN-5
	C7								
Nst1_612	C7	arCOG00980	O	SlpA	FKBP-type peptidyl-prolyl cis-trans isomerase 2	48477400	118	Euryarchaeota	Picrophilus torridus DSM 9790
Nst1_613	C7	arCOG02201	D	FtsZ	Cell division GTPase	41614929	357	Nanoarchaeota	Nanoarchaeum equitans Kin4-M
Nst1_614	C7	arCOG01920	K	NusG	Transcription antiterminator NusG	41615322	116	Nanoarchaeota	Nanoarchaeum equitans Kin4-M
Nst1_615	C7	arCOG02204	U	Sss1	Preprotein translocase subunit Sss1	169861053	33.9		
Nst1_616	C7	arCOG04372	J	RplK	Ribosomal protein L11	256811381	187	Euryarchaeota	Methanocaldococcus fervens AG86
Nst1_617	C7				Uncharacterized protein				
Nst1_618	C7	arCOG04289	J	RplA	Ribosomal protein L1	41615328	191	Nanoarchaeota	Nanoarchaeum equitans Kin4-M
Nst1_619	C7	arCOG04288	J	RplJ	Ribosomal protein L10	41614888	176	Nanoarchaeota	Nanoarchaeum equitans Kin4-M
Nst1_620	C7				Uncharacterized protein				
Nst1_621	C7				Uncharacterized protein				
Nst1_622	C7	arCOG01241	L	XerC	Integrase	332158066	201	Euryarchaeota	Pyrococcus sp. NA2
Nst1_623	C7	arCOG05350	S		Uncharacterized membrane protein	13541564	95.9	Euryarchaeota	Thermoplasma volcanium GSS1
Nst1_624	C7	arCOG02721	C	CydA	Cytochrome bd-type quinol oxidase, subunit 1	269986719	276	Euryarchaeota	Candidatus Parvarchaeum acidiphilum ARMAN-4
Nst1_625	C7	arCOG04490	E		Pyruvoyl-dependent arginine decarboxylase (PvlArgDC)	284161469	149	Euryarchaeota	Archaeoglobus profundus DSM 5631
Nst1_626	C7	arCOG04254	J	RpsG	Ribosomal protein S7	41615036	253	Nanoarchaeota	Nanoarchaeum equitans Kin4-M
Nst1_627	C7	arCOG01572	S	#N/A	Uncharacterized conserved membrane protein	126465119	72.8	Crenarchaeota	Staphylothermus marinus F1
Nst1_628	C7	arCOG04255	J	RpsL	Ribosomal protein S12	41614854	213	Nanoarchaeota	Nanoarchaeum equitans Kin4-M
Nst1_629	C7	arCOG01752	J	RPL30	Ribosomal protein L30E	41614974	85.9	Nanoarchaeota	Nanoarchaeum equitans Kin4-M
Nst1_630	C7	arCOG04256	K	RpoC/Rpo11	DNA-directed RNA polymerase subunit A''	41615213	285	Nanoarchaeota	Nanoarchaeum equitans Kin4-M
Nst1_631	C7	arCOG04257	K	RpoC/Rpo3	DNA-directed RNA polymerase subunit A'	41615286	807	Nanoarchaeota	Nanoarchaeum equitans Kin4-M
Nst1_632	C7	arCOG01762	K	RpoB/Rpo2	DNA-directed RNA polymerase subunit B	41614952	658	Nanoarchaeota	Nanoarchaeum equitans Kin4-M
Nst1_633	C7	arCOG01762	K	RpoB/Rpo2	DNA-directed RNA polymerase subunit B	41614968	364	Nanoarchaeota	Nanoarchaeum equitans Kin4-M
Nst1_634	C7	arCOG04258	K	RPB5	DNA-directed RNA polymerase, subunit H, RpoH/RPB5	14520829	100	Euryarchaeota	Pyrococcus abyssi GE5
Nst1_635	C7	arCOG01589	H	RimK	Glutathione synthase/glutaminyl transferase/alpha-L-glutamate ligase	336122111	102	Euryarchaeota	Methanothermococcus okinawensis IH1
Nst1_636	C7	arCOG04171	S		Uncharacterized conserved protein	332159137	152	Euryarchaeota	Pyrococcus sp. NA2
Nst1_637	C7				Uncharacterized protein				
Nst1_638	C7	arCOG01814	S		Uncharacterized conserved membrane protein	359415798	225	Euryarchaeota	Candidatus Haloredivivus sp. G17
Nst1_639	C7				Uncharacterized protein				
Nst1_640	C7				Uncharacterized protein				
Nst1_641	C7				Membrane protein				
Nst1_642	C7				Uncharacterized protein				
Nst1_643	C7	arCOG03611	E		Peptidase C1A subfamily	118387500	76.6		
Nst1_644	C7				Uncharacterized membrane protein	11499529	46.2	Euryarchaeota	Archaeoglobus fulgidus DSM 4304
Nst1_645	C7				Uncharacterized protein				
Nst1_646	C7				Uncharacterized membrane protein				
Nst1_647	C7				Uncharacterized protein				
Nst1_648	C7				Uncharacterized protein				
Nst1_649	C7				Uncharacterized membrane protein				
Nst1_650	C7	arCOG04182	J	RPS24A	Ribosomal protein S24E	20092495	84	Euryarchaeota	Methanosarcina acetivorans C2A
Nst1_651	C7	arCOG04183	J	RPS31	Ribosomal protein S27AE	41614971	77	Nanoarchaeota	Nanoarchaeum equitans Kin4-M
Nst1_652	C7	arCOG03113	S		Uncharacterized conserved protein	14590942	54.3	Euryarchaeota	Pyrococcus horikoshii OT3
Nst1_653	C7				Uncharacterized protein				
Nst1_654	C7	arCOG00905	L		Uracil-DNA glycosylase	41615161	244	Nanoarchaeota	Nanoarchaeum equitans Kin4-M
Nst1_655	C7	arCOG01700	E	SpeB	Arginase family enzyme	41615017	151	Nanoarchaeota	Nanoarchaeum equitans Kin4-M
Nst1_656	C7				Uncharacterized membrane protein				
Nst1_657	C7				Uncharacterized protein				
Nst1_658	C7				Uncharacterized protein				
Nst1_659	C7				Uncharacterized membrane protein				

Acd1

Locus	Contig	arCOG		Symbol	Annotation	Best NR database hit	Blast Score	Best hit phylum	Best hit species
Acd1_0001	C1	arCOG04348	H	UbiE	Methylase involved in ubiquinone/menaquinone biosynthesis	227829884	567	Crenarchaeota	Sulfolobus islandicus L.S.2.15
Acd1_0002	C1	arCOG00147	G	ProP	Permease of the major facilitator superfamily	15922166	352	Crenarchaeota	Sulfolobus tokodaii str. 7
Acd1_0003	C1	arCOG05912	S		Uncharacterized conserved protein	332796186	308	Crenarchaeota	Acidianus hospitalis W1
Acd1_0004	C1	arCOG03117	S	DedA	Uncharacterized membrane-associated protein, DedA family	15899505	275	Crenarchaeota	Sulfolobus solfataricus P2
Acd1_0005	C1	arCOG00144	G	ProP	Permease of the major facilitator superfamily	374631136	255	Crenarchaeota	Metallosphaera yellowstonensis MK1
Acd1_0006	C1	arCOG01568	M	MscS	Small-conductance mechanosensitive channel	15897472	105	Crenarchaeota	Sulfolobus solfataricus P2
Acd1_0007	C1		S		Uncharacterized protein	313674004	1147	Deferribacteres	Calditerrivibrio nitroreducens DSM 19672
Acd1_0008	C1		S		Uncharacterized protein	385772957	306	Crenarchaeota	Sulfolobus islandicus HVE10/4
Acd1_0009	C1	arCOG00184	E	AppF	ABC-type oligopeptide transport system, ATPase component	302349035	93.6	Crenarchaeota	Acidilobus saccharovorans 345-15
Acd1_0010	C1	arCOG00181	E	DppD	ABC-type dipeptide/oligopeptide/nickel transport system, ATPase component	302349034	511	Crenarchaeota	Acidilobus saccharovorans 345-15
Acd1_0011	C1	arCOG00749	E	DppC	ABC-type dipeptide/oligopeptide/nickel transport system, permease component	15898117	329	Crenarchaeota	Sulfolobus solfataricus P2
Acd1_0012	C1	arCOG00751	E	DppB	ABC-type dipeptide/oligopeptide/nickel transport system, permease component	284996874	48.9	Crenarchaeota	Sulfolobus islandicus L.D.8.5
Acd1_0013	C1	arCOG01672	R	-	Predicted solute binding protein	229583991	289	Crenarchaeota	Sulfolobus islandicus M.16.27
Acd1_0014	C1	arCOG03639	R	-	Predicted glutamine amidotransferase	15922160	693	Crenarchaeota	Sulfolobus tokodaii str. 7
Acd1_0015	C1	arCOG03671	E		Thermopsin-like protease	302348440	101	Crenarchaeota	Acidilobus saccharovorans 345-15
tRNALeuCAA					tRNALeuCAA				
Acd1_0016	C1	arCOG02007	O	-	Highly conserved protein containing a thioredoxin domain	330835123	90.1	Crenarchaeota	Metallosphaera cuprina Ar-4
Acd1_0017	C1	arCOG01992	T	SixA	Phosphohistidine phosphatase SixA	227829401	209	Crenarchaeota	Sulfolobus islandicus L.S.2.15
Acd1_0018	C1	arCOG05138	F	GppA	Exopolyphosphatase	70607737	100	Crenarchaeota	Sulfolobus acidocaldarius DSM 639
Acd1_0019	C1	arCOG01891	F	Tmk	Thymidylate kinase	146303757	588	Crenarchaeota	Metallosphaera sedula DSM 5348
Acd1_0020	C1	arCOG01891	F	Tmk	Thymidylate kinase	332796138	461	Crenarchaeota	Acidianus hospitalis W1
Acd1_0021	C1	arCOG07254	S	-	Uncharacterized conserved protein	332796137	36.6	Crenarchaeota	Acidianus hospitalis W1
Acd1_0022	C1	arCOG02007	O	-	Highly conserved protein containing a thioredoxin domain	227826432	99.4	Crenarchaeota	Sulfolobus islandicus M.14.25
Acd1_0023	C1		S		Uncharacterized protein				
Acd1_0024	C1	arCOG02053	T	UspA	Nucleotide-binding protein, UspA family	332796769	260	Crenarchaeota	Acidianus hospitalis W1
Acd1_0025	C1	arCOG00196	V	CcmA	ABC-type multidrug transport system, ATPase component	332796704	124	Crenarchaeota	Acidianus hospitalis W1
Acd1_0026	C1	arCOG01467	V	-	ABC-type multidrug transport system, permease component	330835678	81.3	Crenarchaeota	Metallosphaera cuprina Ar-4
Acd1_0027	C1	arCOG03665	O	-	Subtilase family protease	15922696	280	Crenarchaeota	Sulfolobus tokodaii str. 7
Acd1_0028	C1	arCOG05967	S		Uncharacterized conserved protein	146304134	475	Crenarchaeota	Metallosphaera sedula DSM 5348
Acd1_0029	C1	arCOG01143	T	ApaH	Serine/threonine protein phosphatase PP2A family	15922699	360	Crenarchaeota	Sulfolobus tokodaii str. 7
Acd1_0030	C1	arCOG00631	R	-	CBS domain	70606127	684	Crenarchaeota	Sulfolobus acidocaldarius DSM 639
Acd1_0031	C1		S		Uncharacterized protein				
Acd1_0032	C1	arCOG07183	R	RfbX	Polysaccharide biosynthesis protein, Mvin family	330835691	192	Crenarchaeota	Metallosphaera cuprina Ar-4
Acd1_0033	C1	arCOG05398	M		Glycosyltransferase family A	227829763	172	Crenarchaeota	Sulfolobus islandicus L.S.2.15
Acd1_0034	C1	arCOG07183	R	RfbX	Polysaccharide biosynthesis protein, Mvin family	374632870	1160	Crenarchaeota	Metallosphaera yellowstonensis MK1
Acd1_0035	C1	arCOG00631	R	-	CBS domain	227831149	114	Crenarchaeota	Sulfolobus islandicus L.S.2.15
Acd1_0036	C1	arCOG00085	F	GuaA	GMP synthase, PP-ATPase domain/subunit	15922676	300	Crenarchaeota	Sulfolobus tokodaii str. 7
Acd1_0037	C1	arCOG05943	M	WcaA	Glycosyltransferase	227827727	383	Crenarchaeota	Sulfolobus islandicus M.14.25
Acd1_0038	C1	arCOG00105	H	CobQ	Cobyric acid synthase	342306699	397	Crenarchaeota	Sulfolobus tokodaii str. 7
Acd1_0039	C1	arCOG06004	Q		2-keto-4-pentenoate hydratase	15920297	157	Crenarchaeota	Sulfolobus tokodaii str. 7
Acd1_0040	C1	arCOG01459	E	Tdh	Threonine dehydrogenase or related Zn-dependent dehydrogenase	332796749	256	Crenarchaeota	Acidianus hospitalis W1
Acd1_0041	C1	arCOG04274	H	CbiB	Cobalamin biosynthesis protein CobD/CbiB	70606249	264	Crenarchaeota	Sulfolobus acidocaldarius DSM 639
Acd1_0042	C1	arCOG04338	H	CobS	Cobalamin-5-phosphate synthase	227828389	1201	Crenarchaeota	Sulfolobus islandicus M.14.25
Acd1_0043	C1	arCOG00985	J	-	Predicted RNA-binding protein (contains PUA domain)	15899938	306	Crenarchaeota	Sulfolobus solfataricus P2
Acd1_0044	C1	arCOG00643	E	SelD	Selenophosphate synthase	374632205	264	Crenarchaeota	Metallosphaera yellowstonensis MK1
Acd1_0045	C1	arCOG05961	K	TroR	Mn-dependent transcriptional regulator (DtxR family)	332796744	456	Crenarchaeota	Acidianus hospitalis W1
Acd1_0046	C1	arCOG01035	E	-	Alanine dehydrogenase, mu-crystallin homolog	332796743	45.4	Crenarchaeota	Acidianus hospitalis W1
Acd1_0047	C1	arCOG00492	K	ARO8	Transcriptional regulators containing a DNA-binding HTH domain and an aminotransferase domain (MocR family)	15922665	406	Crenarchaeota	Sulfolobus tokodaii str. 7
Acd1_0048	C1	arCOG00324	R	AcuC	Deacetylase, including yeast histone deacetylase and acetoin utilization protein	15922657	738	Crenarchaeota	Sulfolobus tokodaii str. 7
Acd1_0049	C1	arCOG00679	L	-	Transposase	15922658	461	Crenarchaeota	Sulfolobus tokodaii str. 7
Acd1_0050	C1		S		Uncharacterized protein				
Acd1_0051	C1	arCOG03639	R	-	Predicted glutamine amidotransferase	374632921	550	Crenarchaeota	Metallosphaera yellowstonensis MK1
Acd1_0052	C1	arCOG00500	R	ElaC	Metal-dependent hydrolase of the beta-lactamase superfamily	15922660	333	Crenarchaeota	Sulfolobus tokodaii str. 7
Acd1_0053	C1		S		Uncharacterized protein				
Acd1_0054	C1	arCOG03768	S	0	Uncharacterized conserved protein	70607799	449	Crenarchaeota	Sulfolobus acidocaldarius DSM 639
Acd1_0055	C1	arCOG01154	R	-	Calcineurin-like phosphoesterase superfamily protein	229579815	745	Crenarchaeota	Sulfolobus islandicus Y.G.57.14
Acd1_0056	C1	arCOG00014	G	RbsK	Sugar kinase, ribokinase family	15896975	123	Crenarchaeota	Sulfolobus solfataricus P2
Acd1_0057	C1		S		Uncharacterized protein	70606112	159	Crenarchaeota	Sulfolobus acidocaldarius DSM 639
Acd1_0058	C1	arCOG01389	M	-	Glycosyltransferase	15922664	589	Crenarchaeota	Sulfolobus tokodaii str. 7
Acd1_0059	C1	arCOG07177	S		Uncharacterized conserved protein	330835749	32	Crenarchaeota	Metallosphaera cuprina Ar-4
Acd1_0060	C1	arCOG00969	R	-	Predicted hydrolase (metallo-beta-lactamase superfamily)	15922655	942	Crenarchaeota	Sulfolobus tokodaii str. 7
Acd1_0061	C1	arCOG01096	S	-	Ferritin-like domain containing protein	146303368	88.2	Crenarchaeota	Metallosphaera sedula DSM 5348
Acd1_0062	C1	arCOG05379	R	-	Periplasmic substrate-binding protein	15922653	108	Crenarchaeota	Sulfolobus tokodaii str. 7
Acd1_0063	C1	arCOG05975	S		Uncharacterized conserved protein	15899944	474	Crenarchaeota	Sulfolobus solfataricus P2
Acd1_0064	C1	arCOG00057	M	GlmS	Glucosamine 6-phosphate synthetase	15896988	887	Crenarchaeota	Sulfolobus solfataricus P2
Acd1_0065	C1	arCOG00555	R	-	Distinct helicase family with a unique C-terminal domain including a metal-binding cysteine cluster	15922649	117	Crenarchaeota	Sulfolobus tokodaii str. 7
Acd1_0066	C1	arCOG07320	C	GlpC	Fe-S oxidoreductase	15922648	455	Crenarchaeota	Sulfolobus tokodaii str. 7
Acd1_0067	C1	arCOG00214	H	MoaB	Molybdopterin biosynthesis enzyme	15922647	136	Crenarchaeota	Sulfolobus tokodaii str. 7
Acd1_0068	C1	arCOG01830	R	-	Predicted nucleotidyltransferase	15922646	70.1	Crenarchaeota	Sulfolobus tokodaii str. 7
Acd1_0069	C1	arCOG00755	E	DadA	Glycine/D-amino acid oxidase (deaminating)	15922645	184	Crenarchaeota	Sulfolobus tokodaii str. 7
Acd1_0070	C1	arCOG05907	S		Uncharacterized conserved protein	15896994	185	Crenarchaeota	Sulfolobus solfataricus P2
Acd1_0071	C1	arCOG01879	I		Dolichol kinase family protein	15922642	277	Crenarchaeota	Sulfolobus tokodaii str. 7
Acd1_0072	C1	arCOG05905	S		Uncharacterized conserved protein	15922641	200	Crenarchaeota	Sulfolobus tokodaii str. 7
Acd1_0073	C1	arCOG01598	F	PurT	Formate-dependent phosphoribosylglycinamide formyltransferase (GAR transformylase)	70606056	390	Crenarchaeota	Sulfolobus acidocaldarius DSM 639

Acd1_0074	C1	arCOG00324	R	AcuC	Deacetylase, including yeast histone deacetylase and acetoin utilization protein	15922638	151	Crenarchaeota	Sulfolobus tokodaii str. 7
Acd1_0075	C1	arCOG00601	R	-	Protein containing two CBS domains (some fused to C-terminal double-stranded RNA-binding domain of RaiA family)	70606054	165	Crenarchaeota	Sulfolobus acidocaldarius DSM 639
Acd1_0076	C1	arCOG00062	R	-	Predicted amidohydrolase	374632286	142	Crenarchaeota	Metallosphaera yellowstonensis MK1
Acd1_0077	C1	arCOG05904	S		Uncharacterized conserved protein	70606052	452	Crenarchaeota	Sulfolobus acidocaldarius DSM 639
Acd1_0078	C1	arCOG00344	C	GlcD	FAD/FMN-containing dehydrogenase	342306689	656	Crenarchaeota	Sulfolobus tokodaii str. 7
Acd1_0079	C1	arCOG00337	C	GlcD	FAD/FMN-containing dehydrogenase	146303336	139	Crenarchaeota	Metallosphaera sedula DSM 5348
Acd1_0080	C1	arCOG00333	C	GlpC	Fe-S oxidoreductase	15922627	259	Crenarchaeota	Sulfolobus tokodaii str. 7
Acd1_0081	C1	arCOG00586	D	Soj	ATPase involved in chromosome partitioning, ParA family	70606046	424	Crenarchaeota	Sulfolobus acidocaldarius DSM 639
Acd1_0082	C1	arCOG04886	S		Uncharacterized conserved protein	70606045	133	Crenarchaeota	Sulfolobus acidocaldarius DSM 639
Acd1_0083	C1	arCOG01389	M	-	Glycosyltransferase	15922623	192	Crenarchaeota	Sulfolobus tokodaii str. 7
Acd1_0084	C1	arCOG00666	M	GCD1	N-acetylglucosamine-1-phosphate uridyltransferase	15922616	696	Crenarchaeota	Sulfolobus tokodaii str. 7
Acd1_0085	C1	arCOG04147	P	SodA	Superoxide dismutase	15922615	1218	Crenarchaeota	Sulfolobus tokodaii str. 7
Acd1_0086	C1	arCOG07278	S		Uncharacterized conserved membrane protein	15922614	574	Crenarchaeota	Sulfolobus tokodaii str. 7
Acd1_0087	C1	arCOG04235	O	-	Predicted thioredoxin/glutaredoxin	15922612	87	Crenarchaeota	Sulfolobus tokodaii str. 7
Acd1_0088	C1	arCOG00770	K	DinG	Rad3-related DNA helicase	15922611	227	Crenarchaeota	Sulfolobus tokodaii str. 7
Acd1_0089	C1	arCOG08334	S		Uncharacterized conserved protein	146304616	573	Crenarchaeota	Metallosphaera sedula DSM 5348
Acd1_0090	C1	arCOG02853	P	{NirD}	Ferredoxin subunit of nitrite reductase or ring-hydroxylating dioxygenase	15922610	264	Crenarchaeota	Sulfolobus tokodaii str. 7
Acd1_0091	C1	arCOG02097	E	AroD	3-dehydroquinate dehydratase	305664127	49.3	Crenarchaeota	Ignisphaera aggregans DSM 17230
Acd1_0092	C1	arCOG04134	E	AroA	5-enolpyruvylshikimate-3-phosphate synthase	15922608	122	Crenarchaeota	Sulfolobus tokodaii str. 7
Acd1_0093	C1	arCOG01025	E	-	Archaeal shikimate kinase	15922607	398	Crenarchaeota	Sulfolobus tokodaii str. 7
Acd1_0094	C1	arCOG04133	E	AroC	Chorismate synthase	15922605	108	Crenarchaeota	Sulfolobus tokodaii str. 7
Acd1_0095	C1	arCOG01033	E	AroE	Shikimate 5-dehydrogenase	332796895	453	Crenarchaeota	Acidianus hospitalis W1
Acd1_0096	C1	arCOG00983	E	AroB	3-dehydroquinate synthetase	15897247	598	Crenarchaeota	Sulfolobus solfataricus P2
Acd1_0097	C1	arCOG01049	E	AroA	3-deoxy-D-arabino-heptulosonate 7-phosphate (DAHP) synthase	15922602	607	Crenarchaeota	Sulfolobus tokodaii str. 7
Acd1_0098	C1	arCOG02098	E	PheA	Chorismate mutase	374634119	216	Crenarchaeota	Metallosphaera yellowstonensis MK1
Acd1_0099	C1	arCOG01051	G	-	Transketolase, C-terminal subunit	70606023	41.2	Crenarchaeota	Sulfolobus acidocaldarius DSM 639
Acd1_0100	C1	arCOG01053	G	-	Transketolase, N-terminal subunit	15897243	147	Crenarchaeota	Sulfolobus solfataricus P2
Acd1_0101	C1	arCOG01676	H	ThiF	Dinucleotide-utilizing enzyme involved in molybdopterin and thiamine biosynthesis	332796901	107	Crenarchaeota	Acidianus hospitalis W1
Acd1_0102	C1	arCOG05903	S		Uncharacterized conserved membrane protein	70606020	194	Crenarchaeota	Sulfolobus acidocaldarius DSM 639
Acd1_0103	C1	arCOG00427	L	RecJ/Cdc45	Single-stranded DNA-specific exonuclease RecJ	70606019	171	Crenarchaeota	Sulfolobus acidocaldarius DSM 639
Acd1_0104	C1	arCOG01354	J	-	Subunit of KEOPS complex (Cgi121BUD32KAE1)	146304631	75.9	Crenarchaeota	Metallosphaera sedula DSM 5348
Acd1_0105	C1	arCOG04113	J	RplP	Ribosomal protein L10AE/L16	15922595	337	Crenarchaeota	Sulfolobus tokodaii str. 7
Acd1_0106	C1	arCOG04112	J	DPH2	Diphthamide synthase subunit DPH2	374634179	270	Crenarchaeota	Metallosphaera yellowstonensis MK1
Acd1_0107	C1	arCOG00676	J	-	Predicted RNA-binding protein (consists of S1 domain and a Zn-ribbon domain)	15922593	336	Crenarchaeota	Sulfolobus tokodaii str. 7
Acd1_0108	C1	arCOG04111	K	RPB11	DNA-directed RNA polymerase, subunit L	15922592	803	Crenarchaeota	Sulfolobus tokodaii str. 7
Acd1_0109	C1	arCOG07176	S		Uncharacterized conserved protein	70606014	92.4	Crenarchaeota	Sulfolobus acidocaldarius DSM 639
Acd1_0110	C1	arCOG00579	K	RPB9	DNA-directed RNA polymerase, subunit M/Transcription elongation factor TFIIS	332796909	52	Crenarchaeota	Acidianus hospitalis W1
tRNAArgTCT					tRNAArgTCT				
Acd1_0111	C1	arCOG02298	N	PulO	Peptidase A24A, prepilin type IV	15922589	38.9	Crenarchaeota	Sulfolobus tokodaii str. 7
Acd1_0112	C1	arCOG01707	I	CaiA	Acyl-CoA dehydrogenase	15922588	119	Crenarchaeota	Sulfolobus tokodaii str. 7
Acd1_0114	C1	arCOG00238	P	ArsB	Na+/H+ antiporter NhaD or related arsenite permease	15922587	598	Crenarchaeota	Sulfolobus tokodaii str. 7
Acd1_0113	C1		S		Uncharacterized protein				
Acd1_0115	C1	arCOG00131	G	ProP	Permease of the major facilitator superfamily	70605988	270	Crenarchaeota	Sulfolobus acidocaldarius DSM 639
Acd1_0116	C1	arCOG00773	I	-	Acyl-CoA hydrolase	15922583	419	Crenarchaeota	Sulfolobus tokodaii str. 7
Acd1_0117	C1	arCOG05897	S		Uncharacterized conserved protein	15922582	40.8	Crenarchaeota	Sulfolobus tokodaii str. 7
Acd1_0118	C1	arCOG00284	L	-	HerA helicase	342306681	116	Crenarchaeota	Sulfolobus tokodaii str. 7
Acd1_0119	C1	arCOG00287	L		Predicted NurA-like nuclease	70605992	239	Crenarchaeota	Sulfolobus acidocaldarius DSM 639
Acd1_0120	C1	arCOG01069	P		CoA-dependent NAD(P)H Sulfur Oxidoreductase	15922579	404	Crenarchaeota	Sulfolobus tokodaii str. 7
Acd1_0121	C1	arCOG05898	S		Uncharacterized conserved protein	227826504	301	Crenarchaeota	Sulfolobus islandicus M.14.25
Acd1_0122	C1	arCOG00052	G	Pgi	Glucose-6-phosphate isomerase	70605995	355	Crenarchaeota	Sulfolobus acidocaldarius DSM 639
Acd1_0123	C1	arCOG00486	J	CysS	Cysteinyl-tRNA synthetase	15922576	797	Crenarchaeota	Sulfolobus tokodaii str. 7
Acd1_0124	C1	arCOG01075	F	-	NUDIX family hydrolase	15922575	534	Crenarchaeota	Sulfolobus tokodaii str. 7
Acd1_0125	C1	arCOG05899	S		Cell suface protein	15899045	211	Crenarchaeota	Sulfolobus solfataricus P2
Acd1_0126	C1	arCOG01352	E	GdhA	Glutamate dehydrogenase/leucine dehydrogenase	332796180	257	Crenarchaeota	Acidianus hospitalis W1
Acd1_0127	C1	arCOG02718	S	-	Predicted membrane protein	70606000	75.1	Crenarchaeota	Sulfolobus acidocaldarius DSM 639
Acd1_0128	C1	arCOG03239	R	-	ATPase, predicted component of phage defense system	15922571	81.3	Crenarchaeota	Sulfolobus tokodaii str. 7
Acd1_0129	C1	arCOG01260	I	FabG	Short-chain alcohol dehydrogenase	146304820	142	Crenarchaeota	Metallosphaera sedula DSM 5348
Acd1_0130	C1	arCOG00905	L	-	Uracil-DNA glycosylase	70606003	97.1	Crenarchaeota	Sulfolobus acidocaldarius DSM 639
Acd1_0131	C1	arCOG08341	S		Uncharacterized conserved protein	227826492	100	Crenarchaeota	Sulfolobus islandicus M.14.25
Acd1_0132	C1	arCOG02050	R	-	Predicted permease	15922568	564	Crenarchaeota	Sulfolobus tokodaii str. 7
Acd1_0133	C1	arCOG02100	K	TroR	Mn-dependent transcriptional regulator (DtxR family)	374633922	519	Crenarchaeota	Metallosphaera yellowstonensis MK1
Acd1_0134	C1	arCOG01927	C	CoxG	Carbon monoxide dehydrogenase subunit G, CoxG	70606705	362	Crenarchaeota	Sulfolobus acidocaldarius DSM 639
Acd1_0135	C1	arCOG04172	E	DapA	Dihydrodipicolinate synthase/N-acetylneuraminate lyase	15922566	121	Crenarchaeota	Sulfolobus tokodaii str. 7
Acd1_0136	C1		S		Uncharacterized protein				
Acd1_0137	C1	arCOG05900	K	-	Predicted transcriptional regulator	284175093	427	Crenarchaeota	Sulfolobus solfataricus 98/2
Acd1_0138	C1	arCOG08479	S		Uncharacterized conserved protein	330835136	77.4	Crenarchaeota	Metallosphaera cuprina Ar-4
Acd1_0139	C1	arCOG01749	C	FumC	Fumarase	15922343	160	Crenarchaeota	Sulfolobus tokodaii str. 7
Acd1_0140	C1	arCOG01074	L	MutT	NUDIX family hydrolase	15922344	680	Crenarchaeota	Sulfolobus tokodaii str. 7
Acd1_0141	C1	arCOG01955	P	KefB	Kef-type K+ transport system, membrane component fused TrkA K+ transport system	15922347	316	Crenarchaeota	Sulfolobus tokodaii str. 7
Acd1_0142	C1	arCOG04409	R	-	Predicted nuclease (RNAse H fold)	70605965	122	Crenarchaeota	Sulfolobus acidocaldarius DSM 639
Acd1_0143	C1	arCOG01087	G	TpiA	Triosephosphate isomerase	332796584	380	Crenarchaeota	Acidianus hospitalis W1
Acd1_0144	C1	arCOG07174	S		Uncharacterized conserved protein	15899322	139	Crenarchaeota	Sulfolobus solfataricus P2
Acd1_0145	C1	arCOG00219	P	ModA	ABC-type molybdate transport system, periplasmic component	146304543	122	Crenarchaeota	Metallosphaera sedula DSM 5348
Acd1_0146	C1	arCOG04170	G	GckA	Putative glycerate kinase	15922355	105	Crenarchaeota	Sulfolobus tokodaii str. 7
Acd1_0147	C1	arCOG05388	K		Predicted transciptional regulator, contains HTH domain	15922356	58.9	Crenarchaeota	Sulfolobus tokodaii str. 7
Acd1_0148	C1	arCOG02099	K	TroR	Mn-dependent transcriptional regulator (DtxR family)	146304764	131	Crenarchaeota	Metallosphaera sedula DSM 5348
Acd1_0149	C1	arCOG05894	S		Uncharacterized conserved protein	15897578	174	Crenarchaeota	Sulfolobus solfataricus P2
Acd1_0150	C1	arCOG01776	Q		SAM-dependent methyltransferase	15922360	478	Crenarchaeota	Sulfolobus tokodaii str. 7
Acd1_0151	C1	arCOG00532	H	MobB	Molybdopterin-guanine dinucleotide biosynthesis protein	15922362	151	Crenarchaeota	Sulfolobus tokodaii str. 7
Acd1_0152	C1	arCOG00216	H	MoeA	Molybdopterin biosynthesis enzyme	70605954	485	Crenarchaeota	Sulfolobus acidocaldarius DSM 639
Acd1_0153	C1	arCOG01872	H	MobA	Molybdopterin-guanine dinucleotide biosynthesis protein A	15897584	347	Crenarchaeota	Sulfolobus solfataricus P2
Acd1_0154	C1	arCOG01631	Q		SAM-dependent methyltransferase	70605952	190	Crenarchaeota	Sulfolobus acidocaldarius DSM 639
Acd1_0155	C1	arCOG00035	R	-	Predicted ATPase of PP-loop superfamily	332796243	280	Crenarchaeota	Acidianus hospitalis W1
Acd1_0156	C1	arCOG00695	F	SsnA	Cytosine deaminase or related metal-dependent hydrolase	332796244	144	Crenarchaeota	Acidianus hospitalis W1
Acd1_0157	C1	arCOG02242	K	-	Transcriptional regulator, contains HTH domain	332796245	64.7	Crenarchaeota	Acidianus hospitalis W1
Acd1_0158	C1	arCOG00817	K	AbrB	Transcriptional regulator AbrB	15922369	758	Crenarchaeota	Sulfolobus tokodaii str. 7
Acd1_0159	C1	arCOG00891	L	TatD	Mg-dependent DNase	332796247	638	Crenarchaeota	Acidianus hospitalis W1
Acd1_0160	C1	arCOG05892	C		Terminal oxidase, subunit doxE	15922371	267	Crenarchaeota	Sulfolobus tokodaii str. 7
Acd1_0161	C1	arCOG05891	C		Terminal oxidase, subunit doxC	15922372	664	Crenarchaeota	Sulfolobus tokodaii str. 7
Acd1_0162	C1	arCOG01238	C	CyoB/DoxB	Heme/copper-type cytochrome/quinol oxidase, subunit 1	15922373	373	Crenarchaeota	Sulfolobus tokodaii str. 7
tRNASerGCT					tRNASerGCT				
Acd1_0163	C1	arCOG01456	C	AdhC	Zn-dependent alcohol dehydrogenase	15922376	479	Crenarchaeota	Sulfolobus tokodaii str. 7
Acd1_0164	C1	arCOG03199	M	Rfe	UDP-N-acetylmuramyl pentapeptide phosphotransferase/UDP-N-acetylglucosamine-1-phosphate transferase	330834020	324	Crenarchaeota	Metallosphaera cuprina Ar-4
Acd1_0165	C1	arCOG01726	H	IspA	Geranylgeranyl pyrophosphate synthase	70605942	630	Crenarchaeota	Sulfolobus acidocaldarius DSM 639
Acd1_0166	C1	arCOG00613	H	idi	Isopentenyl diphosphate isomerase	227828316	225	Crenarchaeota	Sulfolobus islandicus M.14.25
Acd1_0167	C1	arCOG00613	H	idi	Isopentenyl diphosphate isomerase	15922379	250	Crenarchaeota	Sulfolobus tokodaii str. 7
Acd1_0168	C1	arCOG05890	S		Uncharacterized conserved protein	15922381	93.2	Crenarchaeota	Sulfolobus tokodaii str. 7
Acd1_0169	C1	arCOG01728	R	-	Predicted dioxygenase	15922382	65.5	Crenarchaeota	Sulfolobus tokodaii str. 7
Acd1_0170	C1	arCOG04245	J	RpsB	Ribosomal protein S2	70605938	520	Crenarchaeota	Sulfolobus acidocaldarius DSM 639
Acd1_0171	C1	arCOG04244	K	RPB10	DNA-directed RNA polymerase, subunit N (RpoN/RPB10)	15897034	87	Crenarchaeota	Sulfolobus solfataricus P2
Acd1_0172	C1	arCOG04243	J	RpsI	Ribosomal protein S9	15922385	158	Crenarchaeota	Sulfolobus tokodaii str. 7
Acd1_0173	C1	arCOG04242	J	RplM	Ribosomal protein L13	332796344	291	Crenarchaeota	Acidianus hospitalis W1
Acd1_0174	C1	arCOG00780	J	RPL18A	Ribosomal protein L18E	15922387	487	Crenarchaeota	Sulfolobus tokodaii str. 7
Acd1_0175	C1	arCOG04241	K	RpoA/Rpo1	DNA-directed RNA polymerase subunit D	15897038	198	Crenarchaeota	Sulfolobus solfataricus P2
Acd1_0176	C1	arCOG04240	J	RpsK	Ribosomal protein S11	15922389	196	Crenarchaeota	Sulfolobus tokodaii str. 7
Acd1_0177	C1	arCOG04239	J	RpsD	Ribosomal protein S4 or related protein	15922390	29.6	Crenarchaeota	Sulfolobus tokodaii str. 7
Acd1_0178	C1	arCOG01722	J	RpsM	Ribosomal protein S13	15922391	148	Crenarchaeota	Sulfolobus tokodaii str. 7
Acd1_0179	C1	arCOG00637	O	HypE	Hydrogenase maturation factor	332796350	298	Crenarchaeota	Acidianus hospitalis W1
Acd1_0180	C1	arCOG04214	S	-	Uncharacterized conserved protein	70605928	273	Crenarchaeota	Sulfolobus acidocaldarius DSM 639
Acd1_0181	C1	arCOG04214	S	-	Uncharacterized conserved protein	330834004	203	Crenarchaeota	Metallosphaera cuprina Ar-4
Acd1_0182	C1	arCOG01886	J	TyrS	Tyrosyl-tRNA synthetase	363548504	296	Crenarchaeota	Sulfolobus tokodaii str. 7
Acd1_0183	C1	arCOG04281	L	DnaG	DNA primase (bacterial type)	15922396	442	Crenarchaeota	Sulfolobus tokodaii str. 7
Acd1_0184	C1	arCOG00328	L	PolB	DNA polymerase elongation subunit (family B)	15922397	638	Crenarchaeota	Sulfolobus tokodaii str. 7
Acd1_0185	C1	arCOG00833	R	RimI	Acetyltransferase (GNAT) family	15922398	54.3	Crenarchaeota	Sulfolobus tokodaii str. 7
Acd1_0186	C1	arCOG01741	J	PelA	Release factor eRF1	15922399	491	Crenarchaeota	Sulfolobus tokodaii str. 7
Acd1_0187	C1	arCOG00932	R	-	Uncharacterized conserved protein related to pyruvate formate-lyase activating enzyme	332796358	833	Crenarchaeota	Acidianus hospitalis W1
Acd1_0188	C1	arCOG01699	S	-	Uncharacterized conserved protein	70605920	183	Crenarchaeota	Sulfolobus acidocaldarius DSM 639
Acd1_0189	C1	arCOG04066	R	-	Minimal nucleotidyltransferase	15922402	70.1	Crenarchaeota	Sulfolobus tokodaii str. 7
Acd1_0190	C1	arCOG04064	M	-	Predicted membrane-associated Zn-dependent protease	70605918	385	Crenarchaeota	Sulfolobus acidocaldarius DSM 639
Acd1_0191	C1	arCOG00408	J	LysU	Lysyl-tRNA synthetase (class II)	342306641	83.6	Crenarchaeota	Sulfolobus tokodaii str. 7
tRNATrpCCA					tRNATrpCCA				
Acd1_0192	C1	arCOG00834	K	WecD	Acetyltransferase (GNAT) family	15922418	159	Crenarchaeota	Sulfolobus tokodaii str. 7
Acd1_0193	C1	arCOG00249	I	FadB	3-hydroxyacyl-CoA dehydrogenase	227828604	310	Crenarchaeota	Sulfolobus islandicus M.14.25
Acd1_0194	C1	arCOG02623	H	PaaK	Coenzyme F390 synthetase	15922420	84.3	Crenarchaeota	Sulfolobus tokodaii str. 7
Acd1_0195	C1	arCOG02143	Q	-	Aromatic ring hydroxylase	15921962	1112	Crenarchaeota	Sulfolobus tokodaii str. 7
Acd1_0196	C1	arCOG05888	L		Chromosomal protein Sac7d/Sso7d	332797929	533	Crenarchaeota	Acidianus hospitalis W1
tRNAProTGG					tRNAProTGG				
Acd1_0197	C1	arCOG01710	I	Sbm	Methylmalonyl-CoA mutase, C-terminal domain/subunit (cobalamin-binding)	15922422	198	Crenarchaeota	Sulfolobus tokodaii str. 7
Acd1_0198	C1	arCOG01226	E	ArgK	Putative periplasmic protein kinase ArgK or related GTPase of G3E family	342306644	205	Crenarchaeota	Sulfolobus tokodaii str. 7
Acd1_0199	C1	arCOG03698	K	-	Predicted transciptional regulator, contains HTH domain	332796197	107	Crenarchaeota	Acidianus hospitalis W1

Acd1_0200	C1	arCOG01648	R	MhpC	Alpha/beta superfamily hydrolase	15922425	248	Crenarchaeota	Sulfolobus tokodaii str. 7
Acd1_0201	C1	arCOG04435	G	-	Phosphoenolpyruvate carboxylase	160380584	86.7	Crenarchaeota	Sulfolobus acidocaldarius DSM 639
Acd1_0202	C1	arCOG00310	O	Bcp	Peroxiredoxin	15922428	226	Crenarchaeota	Sulfolobus tokodaii str. 7
Acd1_0203	C1	arCOG00018	G	-	Predicted sugar kinase	332797957	31.6	Crenarchaeota	Acidianus hospitalis W1
Acd1_0204	C1	arCOG01106	S		Uncharacterized conserved protein	70605908	319	Crenarchaeota	Sulfolobus acidocaldarius DSM 639
Acd1_0205	C1	arCOG01098	S		Uncharacterized conserved protein	70605907	463	Crenarchaeota	Sulfolobus acidocaldarius DSM 639
Acd1_0206	C1	arCOG00280	L	-	HerA helicase	70605906	384	Crenarchaeota	Sulfolobus acidocaldarius DSM 639
Acd1_0207	C1	arCOG00397	L	SbcD	DNA repair exonuclease, SbcD	15899023	371	Crenarchaeota	Sulfolobus solfataricus P2
Acd1_0208	C1	arCOG00368	L	SbcC	ATPase involved in DNA repair, SbcC	332797961	110	Crenarchaeota	Acidianus hospitalis W1
Acd1_0209	C1	arCOG00367	L	-	NurA 5'-3' nuclease	15922435	284	Crenarchaeota	Sulfolobus tokodaii str. 7
Acd1_0210	C1	arCOG01773	Q		SAM-dependent methyltransferase	330835099	244	Crenarchaeota	Metallosphaera cuprina Ar-4
Acd1_0211	C1	arCOG00600	R	-	CBS domain	15922439	150	Crenarchaeota	Sulfolobus tokodaii str. 7
Acd1_0212	C1	arCOG04373	S	-	Uncharacterized conserved protein	15922440	283	Crenarchaeota	Sulfolobus tokodaii str. 7
Acd1_0213	C1	arCOG01868	P	Fur	Fe2+/Zn2+ uptake regulation protein, fur/PerR	332797968	316	Crenarchaeota	Acidianus hospitalis W1
Acd1_0214	C1	arCOG04558	F	URH1	Inosine-uridine nucleoside N-ribohydrolase	15922442	224	Crenarchaeota	Sulfolobus tokodaii str. 7
Acd1_0215	C1	arCOG00373	L		DNA sulfur modification protein DndD, ATPase	332797970	234	Crenarchaeota	Acidianus hospitalis W1
Acd1_0216	C1	arCOG05886	S		Uncharacterized conserved protein	15899012	270	Crenarchaeota	Sulfolobus solfataricus P2
Acd1_0217	C1	arCOG00631	R	-	CBS domain	15922445	534	Crenarchaeota	Sulfolobus tokodaii str. 7
Acd1_0218	C1	arCOG01991	R	phoE	Broad specificity phosphatase PhoE or related phosphatase	332797974	127	Crenarchaeota	Acidianus hospitalis W1
Acd1_0219	C1	arCOG01616	J	GEK1	D-aminoacyl-tRNA deacylase, involved in ethanol tolerance	332797975	327	Crenarchaeota	Acidianus hospitalis W1
Acd1_0220	C1	arCOG01972	O	TrxA	Thiol-disulfide isomerase or thioredoxin	15922449	101	Crenarchaeota	Sulfolobus tokodaii str. 7
Acd1_0221	C1	arCOG05323	S		Uncharacterized conserved protein	15922450	650	Crenarchaeota	Sulfolobus tokodaii str. 7
Acd1_0222	C1	arCOG01221	F	-	Inosine/xanthosine triphosphatase	70605892	211	Crenarchaeota	Sulfolobus acidocaldarius DSM 639
Acd1_0223	C1		S		Uncharacterized protein				
Acd1_0224	C1	arCOG08372	S		Uncharacterized conserved protein	70605889	29.6	Crenarchaeota	Sulfolobus acidocaldarius DSM 639
Acd1_0225	C1	arCOG03765	S		Uncharacterized conserved membrane protein	70605888	146	Crenarchaeota	Sulfolobus acidocaldarius DSM 639
Acd1_0225.1	C1	arCOG04122	J	Mra1	EMG1/NEP1 rRNA methyltransferase	330835210	122	Crenarchaeota	Metallosphaera cuprina Ar-4
Acd1_0226	C1	arCOG04345	J	RPR2	RNase P subunit RPR2	227826453	62.4	Crenarchaeota	Sulfolobus islandicus M.14.25
Acd1_0227	C1	arCOG01346	J	-	Predicted RNA-binding protein containing KH domain, possibly ribosomal protein	227826452	176	Crenarchaeota	Sulfolobus islandicus M.14.25
Acd1_0228	C1	arCOG04171	S	-	Uncharacterized conserved protein	70605884	122	Crenarchaeota	Sulfolobus acidocaldarius DSM 639
Acd1_0229	C1	arCOG00033	J	Trm5	Wybutosine (yW) biosynthesis enzyme, Trm5 methyltransferase	70605883	747	Crenarchaeota	Sulfolobus acidocaldarius DSM 639
Acd1_0230	C1	arCOG01296	O	TrxB	Thioredoxin reductase	15922460	347	Crenarchaeota	Sulfolobus tokodaii str. 7
Acd1_0231	C1	arCOG05885	S		Uncharacterized conserved protein	146303525	538	Crenarchaeota	Metallosphaera sedula DSM 5348
Acd1_0232	C1	arCOG01348	H	nadF	NAD kinase	15922463	238	Crenarchaeota	Sulfolobus tokodaii str. 7
Acd1_0233	C1	arCOG00721	J	Nob1	Endonuclease Nob1, consists of a PIN domain and a Zn-ribbon module	15922464	153	Crenarchaeota	Sulfolobus tokodaii str. 7
Acd1_0234	C1	arCOG01529	I	Acs	Acyl-coenzyme A synthetase/AMP-(fatty) acid ligase	15922466	681	Crenarchaeota	Sulfolobus tokodaii str. 7
Acd1_0235	C1	arCOG00065	O	csdA	Selenocysteine lyase/Cysteine desulfurase	15922467	322	Crenarchaeota	Sulfolobus tokodaii str. 7
Acd1_0236	C1	arCOG05884	S		Uncharacterized conserved protein	227829051	622	Crenarchaeota	Sulfolobus islandicus L.S.2.15
Acd1_0237	C1	arCOG04272	H	CobT	NaMN:DMB phosphoribosyltransferase	15922470	40.8	Crenarchaeota	Sulfolobus tokodaii str. 7
Acd1_0238	C1	arCOG04479	K	-	Predicted transcriptional regulator containing an HTH domain fused to a Zn-ribbon	15898941	243	Crenarchaeota	Sulfolobus solfataricus P2
Acd1_0239	C1	arCOG02291	R	-	HAD superfamily hydrolase	15922472	464	Crenarchaeota	Sulfolobus tokodaii str. 7
Acd1_0240	C1	arCOG00910	J	-	Predicted RNA methylase	384435202	89	Crenarchaeota	Sulfolobus solfataricus 98/2
Acd1_0241	C1	arCOG01213	R	Cof	HAD superfamily hydrolase	284999293	127	Crenarchaeota	Sulfolobus islandicus L.D.8.5
Acd1_0242	C1	arCOG01894	L	Nfo	Endonuclease IV	342306655	162	Crenarchaeota	Sulfolobus tokodaii str. 7
Acd1_0243	C1	arCOG00340	C	GlcD	FAD/FMN-containing dehydrogenase fused to Heterodisulfide reductase, subunit B	229583549	37.7	Crenarchaeota	Sulfolobus islandicus Y.N.15.51
Acd1_0244	C1	arCOG00419	F	Hit	HIT family hydrolase	158428568	392	Crenarchaeota	Sulfolobus tokodaii
Acd1_0245	C1	arCOG03119	S		DedA family membrane protein	15922478	168	Crenarchaeota	Sulfolobus tokodaii str. 7
Acd1_0246	C1	arCOG01073	L	MutT	NUDIX family hydrolase	15922479	294	Crenarchaeota	Sulfolobus tokodaii str. 7
Acd1_0247	C1	arCOG08320	S		Uncharacterized conserved protein	374632136	140	Crenarchaeota	Metallosphaera yellowstonensis MK1
Acd1_0248	C1	arCOG01034	F	-	Predicted nucleotide kinase	332795951	47	Crenarchaeota	Acidianus hospitalis W1
Acd1_0249	C1	arCOG00069	H	NadE	NH3-dependent NAD+ synthetase	327310179	439	Crenarchaeota	Thermoproteus uzoniensis 768-20
Acd1_0250	C1	arCOG00731	K	ArsR	Transcriptional regulator containing HTH domain, ArsR family	70605859	778	Crenarchaeota	Sulfolobus acidocaldarius DSM 639
Acd1_0251	C1	arCOG05883	S		Uncharacterized conserved protein	15922487	103	Crenarchaeota	Sulfolobus tokodaii str. 7
Acd1_0252	C1	arCOG05489	S		Uncharacterized conserved protein	374631858	300	Crenarchaeota	Metallosphaera yellowstonensis MK1
Acd1_0253	C1		S		Uncharacterized protein				
Acd1_0254	C1	arCOG01572	S		Uncharacterized conserved membrane protein	15922490	127	Crenarchaeota	Sulfolobus tokodaii str. 7
Acd1_0255	C1	arCOG07173	S		Uncharacterized conserved protein	227826414	266	Crenarchaeota	Sulfolobus islandicus M.14.25
Acd1_0256	C1	arCOG00467	L	CDC6	Cdc6-related protein, AAA superfamily ATPase	15922492	75.1	Crenarchaeota	Sulfolobus tokodaii str. 7
Acd1_0257	C1	arCOG01164	C	Icd	Isocitrate dehydrogenase	229580721	360	Crenarchaeota	Sulfolobus islandicus Y.G.57.14
Acd1_0258	C1	arCOG07274	S		Uncharacterized conserved protein	284175462	397	Crenarchaeota	Sulfolobus solfataricus 98/2
Acd1_0259	C1	arCOG06040	S		Uncharacterized conserved protein	227831754	196	Crenarchaeota	Sulfolobus islandicus L.S.2.15
Acd1_0260	C1	arCOG00494	E	Asd	Aspartate-semialdehyde dehydrogenase	332798017	673	Crenarchaeota	Acidianus hospitalis W1
Acd1_0261	C1	arCOG04045	E	IlvD	Dihydroxyacid dehydratase/phosphogluconate dehydratase	15922499	135	Crenarchaeota	Sulfolobus tokodaii str. 7
Acd1_0262	C1	arCOG01791	Q		SAM-dependent methyltransferase	15898957	397	Crenarchaeota	Sulfolobus solfataricus P2
Acd1_0263	C1	arCOG08538	S		Uncharacterized conserved protein	332798013	77.8	Crenarchaeota	Acidianus hospitalis W1
Acd1_0264	C1	arCOG01035	E	-	Alanine dehydrogenase, mu-crystallin homolog	15922506	590	Crenarchaeota	Sulfolobus tokodaii str. 7
Acd1_0265	C1	arCOG00489	H	-	cob(I)alamin adenosyltransferase	15922507	117	Crenarchaeota	Sulfolobus tokodaii str. 7
Acd1_0266	C1	arCOG00271	G	RhaT	Permease of the drug/metabolite transporter (DMT) superfamily	342306661	230	Crenarchaeota	Sulfolobus tokodaii str. 7
Acd1_0267	C1	arCOG04060	K	-	Predicted transcriptional regulator	15922518	204	Crenarchaeota	Sulfolobus tokodaii str. 7
Acd1_0268	C1	arCOG04061	K	EGD2	Transcription factor homologous to NACalpha-BTF3	70608063	650	Crenarchaeota	Sulfolobus acidocaldarius DSM 639
Acd1_0269	C1	arCOG00541	J	YSH1	Predicted exonuclease of the beta-lactamase fold involved in RNA processing	15922516	154	Crenarchaeota	Sulfolobus tokodaii str. 7
Acd1_0270	C1	arCOG00401	J	ThrS	Threonyl-tRNA synthetase	70608065	124	Crenarchaeota	Sulfolobus acidocaldarius DSM 639
Acd1_0271	C1	arCOG01028	I	ERG12	Mevalonate kinase	15922513	50.1	Crenarchaeota	Sulfolobus tokodaii str. 7
Acd1_0272	C1	arCOG00057	M	GlmS	Glucosamine 6-phosphate synthetase	342306662	485	Crenarchaeota	Sulfolobus tokodaii str. 7
Acd1_0273	C1		S		Uncharacterized protein	229580093	81.3	Crenarchaeota	Sulfolobus islandicus Y.G.57.14
Acd1_0274	C1	arCOG00666	M	GCD1	N-acetylglucosamine-1-phosphate uridyltransferase	227827988	192	Crenarchaeota	Sulfolobus islandicus M.14.25
Acd1_0275	C1		S		Uncharacterized protein	13542043	49.7	Euryarchaeota	Thermoplasma volcanium GSS1
tRNAGlyGCC					tRNAGlyGCC				
Acd1_0276	C1	arCOG02474	C	HyaA	Ni,Fe-hydrogenase I small subunit	374631473	306	Crenarchaeota	Metallosphaera yellowstonensis MK1
Acd1_0277	C1	arCOG01550	C	HyaB	Ni,Fe-hydrogenase I large subunit	374631472	790	Crenarchaeota	Metallosphaera yellowstonensis MK1
Acd1_0278	C1	arCOG03658	F	NrdF	Ribonucleotide reductase, beta subunit	15921920	109	Crenarchaeota	Sulfolobus tokodaii str. 7
Acd1_0279	C1	arCOG01308	O	Cdc48	ATPase of the AAA+ class , CDC48 family	15922015	103	Crenarchaeota	Sulfolobus tokodaii str. 7
Acd1_0280	C1	arCOG06032	S		Uncharacterized conserved protein	15922007	295	Crenarchaeota	Sulfolobus tokodaii str. 7
Acd1_0281	C1	arCOG00235	Q	MhpD	2-keto-4-pentenoate hydratase/2-oxohepta-3-ene-1,7-dioic acid hydratase (catechol pathway)	15922009	271	Crenarchaeota	Sulfolobus tokodaii str. 7
Acd1_0282	C1	arCOG05946	S		Uncharacterized conserved protein	332796621	55.8	Crenarchaeota	Acidianus hospitalis W1
Acd1_0283	C1	arCOG03696	C	NapH	Polyferredoxin	15922005	120	Crenarchaeota	Sulfolobus tokodaii str. 7
Acd1_0284	C1		S		Uncharacterized protein				
Acd1_0285	C1	arCOG01055	K	-	Predicted transcriptional regulator	15920879	163	Crenarchaeota	Sulfolobus tokodaii str. 7
Acd1_0286	C1	arCOG02312	V	SalY	ABC-type antimicrobial peptide transport system, permease component	146304292	99.8	Crenarchaeota	Metallosphaera sedula DSM 5348
Acd1_0287	C1	arCOG02079	M	-	S-layer domain	330834570	141	Crenarchaeota	Metallosphaera cuprina Ar-4
Acd1_0288	C1	arCOG00922	V	SalX	ABC-type antimicrobial peptide transport system, ATPase component	374633508	626	Crenarchaeota	Metallosphaera yellowstonensis MK1
Acd1_0289	C1	arCOG00144	G	ProP	Permease of the major facilitator superfamily	227827350	238	Crenarchaeota	Sulfolobus islandicus M.14.25
Acd1_0290	C1	arCOG01145	R		Calcineurin-like phosphoesterase	374632183	129	Crenarchaeota	Metallosphaera yellowstonensis MK1
Acd1_0291	C1	arCOG05706	E	DAP2	Dipeptidyl aminopeptidase/acylaminoacyl-peptidase	15898272	223	Crenarchaeota	Sulfolobus solfataricus P2
Acd1_0292	C1	arCOG04331	R	-	Predicted thioesterase	15920895	615	Crenarchaeota	Sulfolobus tokodaii str. 7
Acd1_0293	C1	arCOG02741	H	ThiC	Thiamine biosynthesis protein ThiC	332796889	67.8	Crenarchaeota	Acidianus hospitalis W1
Acd1_0294	C1	arCOG00619	E	GltB	Glutamate synthase domain 2 and ferredoxin domain	342306666	605	Crenarchaeota	Sulfolobus tokodaii str. 7
Acd1_0295	C1	arCOG00095	E	GltB	Glutamate synthase domain 1	15922527	32.3	Crenarchaeota	Sulfolobus tokodaii str. 7
Acd1_0296	C1	arCOG00652	H	CbiG	Cobalamin biosynthesis protein CbiG	70608030	357	Crenarchaeota	Sulfolobus acidocaldarius DSM 639
Acd1_0297	C1	arCOG04233	P	FepB	ABC-type Fe3+-hydroxamate transport system, periplasmic component	385776578	127	Crenarchaeota	Sulfolobus islandicus REY15A
Acd1_0298	C1	arCOG07271	S		Uncharacterized conserved protein	15922530	41.6	Crenarchaeota	Sulfolobus tokodaii str. 7
Acd1_0299	C1	arCOG02092	E	LeuA	Isopropylmalate/homocitrate/citramalate synthase	15922531	193	Crenarchaeota	Sulfolobus tokodaii str. 7
Acd1_0300	C1	arCOG00477	H	UbiA	4-hydroxybenzoate polyprenyltransferase or related prenyltransferase	15922532	251	Crenarchaeota	Sulfolobus tokodaii str. 7
Acd1_0301	C1	arCOG03271	S	-	Uncharacterized conserved protein, AIG2 family	15922533	83.2	Crenarchaeota	Sulfolobus tokodaii str. 7
Acd1_0302	C1	arCOG05511	R		OB-fold containing protein	119873076	80.9	Crenarchaeota	Pyrobaculum islandicum DSM 4184
Acd1_0303	C1	arCOG00021	H	-	Predicted transcriptional regulator fused phosphomethylpyrimidine kinase, involved in the thiamin biosynthesis	332795735	304	Crenarchaeota	Acidianus hospitalis W1
Acd1_0304	C1	arCOG00002	K	-	Predicted transcriptional regulator, PadR family	15922538	252	Crenarchaeota	Sulfolobus tokodaii str. 7
Acd1_0305	C1	arCOG02994	S	-	Cupin domain containing protein	15898388	255	Crenarchaeota	Sulfolobus solfataricus P2
Acd1_0307	C1		S		Uncharacterized protein				
Acd1_0306	C1	arCOG03216	S	-	Uncharacterized conserved protein	15922540	599	Crenarchaeota	Sulfolobus tokodaii str. 7
Acd1_0308	C1	arCOG06038	S		Uncharacterized conserved protein, contains thioredoxin domain	15922541	256	Crenarchaeota	Sulfolobus tokodaii str. 7
Acd1_0309	C1	arCOG00317	S	-	Uncharacterized conserved protein	70608053	105	Crenarchaeota	Sulfolobus acidocaldarius DSM 639
Acd1_0310	C1	arCOG01883	F	THY1	Thymidylate synthase	332796879	396	Crenarchaeota	Acidianus hospitalis W1
Acd1_0311	C1	arCOG01557	C	NuoA	NADH dehydrogenase subunit A	70608051	956	Crenarchaeota	Sulfolobus acidocaldarius DSM 639
Acd1_0312	C1	arCOG01551	C	NuoC	NADH dehydrogenase subunit C	15922545	30.8	Crenarchaeota	Sulfolobus tokodaii str. 7
Acd1_0313	C1	arCOG01548	C	NuoD	NADH dehydrogenase subunit D	15922546	338	Crenarchaeota	Sulfolobus tokodaii str. 7
Acd1_0314	C1	arCOG01546	C	NuoH	NADH dehydrogenase subunit H	15922547	271	Crenarchaeota	Sulfolobus tokodaii str. 7
Acd1_0315	C1	arCOG01543	C	NuoI	NADH dehydrogenase subunit I	15922548	129	Crenarchaeota	Sulfolobus tokodaii str. 7
Acd1_0316	C1	arCOG04654	C	NuoJ	NADH dehydrogenase subunit J	15897267	635	Crenarchaeota	Sulfolobus solfataricus P2
Acd1_0317	C1	arCOG03073	C	NuoK	NADH dehydrogenase subunit 4L (K,kappa)	332796872	697	Crenarchaeota	Acidianus hospitalis W1
Acd1_0318	C1	arCOG01539	C	NuoL	NADH dehydrogenase subunit L	15922551	1434	Crenarchaeota	Sulfolobus tokodaii str. 7
Acd1_0319	C1	arCOG01540	C	NuoN	NADH dehydrogenase subunit N	227830771	491	Crenarchaeota	Sulfolobus islandicus L.S.2.15
Acd1_0320	C1	arCOG04213	I	INO1	Myo-inositol-1-phosphate synthase	385776376	147	Crenarchaeota	Sulfolobus islandicus REY15A
Acd1_0321	C1	arCOG00038	H	ThiI	Thiamine biosynthesis ATP pyrophosphatase	15922554	182	Crenarchaeota	Sulfolobus tokodaii str. 7
Acd1_0322	C1	arCOG06041	S		Uncharacterized conserved protein	15922585	179	Crenarchaeota	Sulfolobus tokodaii str. 7
Acd1_0323	C1	arCOG00600	R	-	CBS domain	332797345	142	Crenarchaeota	Acidianus hospitalis W1
Acd1_0324	C1	arCOG01529	I	Acs	Acyl-coenzyme A synthetase/AMP-(fatty) acid ligase	15922340	148	Crenarchaeota	Sulfolobus tokodaii str. 7
Acd1_0325	C1	arCOG03705	R	-	Predicted metal-dependent hydrolase	15921806	209	Crenarchaeota	Sulfolobus tokodaii str. 7
Acd1_0326	C1	arCOG04250	R	-	Solo B3/4 domain (OB-fold DNA/RNA-binding) of Phe-aaRS-beta	332796773	469	Crenarchaeota	Acidianus hospitalis W1
Acd1_0327	C1	arCOG02734	S	-	NifX family protein	146304138	111	Crenarchaeota	Metallosphaera sedula DSM 5348
Acd1_0328	C1	arCOG00283	L	-	HerA helicase	15920360	203	Crenarchaeota	Sulfolobus tokodaii str. 7
Acd1_0329	C1	arCOG05927	L		NurA family nuclease	70606272	322	Crenarchaeota	Sulfolobus acidocaldarius DSM 639

Acd1_0330	C1	arCOG00021	H	-	Predicted transcriptional regulator fused phosphomethylpyrimidine kinase, involved in the thiamin biosynthesis	15920358	387	Crenarchaeota	Sulfolobus tokodaii str. 7
Acd1_0331	C1	arCOG08476	S		Uncharacterized conserved protein	227827906	135	Crenarchaeota	Sulfolobus islandicus M.14.25
Acd1_0332	C1	arCOG05928	S		Uncharacterized conserved protein	15920357	91.7	Crenarchaeota	Sulfolobus tokodaii str. 7
Acd1_0333	C1	arCOG00614	R	SpoIVFB	Zn-dependent protease	352682179	43.1	Crenarchaeota	Thermoproteus tenax Kra 1
Acd1_0334	C1	arCOG01768	E	GlpG	Membrane associated serine protease	284173265	866	Crenarchaeota	Sulfolobus solfataricus 98/2
Acd1_0335	C1	arCOG08342	S		Uncharacterized conserved protein	374633816	633	Crenarchaeota	Metallosphaera yellowstonensis MK1
Acd1_0336	C1	arCOG01038	F	-	Predicted nucleotide kinase (CMP/AMP kinase related)	146304919	941	Crenarchaeota	Metallosphaera sedula DSM 5348
Acd1_0337	C1	arCOG00585	D	Mrp	Mrp family protein, ATPase, contains iron-sulfur cluster	146304920	159	Crenarchaeota	Metallosphaera sedula DSM 5348
Acd1_0338	C1	arCOG08343	S		Uncharacterized conserved protein	374633813	149	Crenarchaeota	Metallosphaera yellowstonensis MK1
Acd1_0339	C1	arCOG02242	K	-	Transcriptional regulator, contains HTH domain	15920352	872	Crenarchaeota	Sulfolobus tokodaii str. 7
Acd1_0340	C1	arCOG00474	H	-	5-formyltetrahydrofolate cyclo-ligase	15920351	410	Crenarchaeota	Sulfolobus tokodaii str. 7
Acd1_0341	C1	arCOG01947	E	RhtB	Putative threonine efflux protein	15920350	304	Crenarchaeota	Sulfolobus tokodaii str. 7
Acd1_0342	C1	arCOG04291	S	-	Uncharacterized conserved protein	332797513	99	Crenarchaeota	Acidianus hospitalis W1
Acd1_0343	C1	arCOG01887	J	TrpS	Tryptophanyl-tRNA synthetase	161751105	729	Crenarchaeota	Sulfolobus tokodaii str. 7
Acd1_0344	C1		S		Uncharacterized protein	15921778	274	Crenarchaeota	Sulfolobus tokodaii str. 7
Acd1_0345	C1	arCOG00452	D	ESCRTIII	Crenarchaeal division protein ESCRT-III	70606286	209	Crenarchaeota	Sulfolobus acidocaldarius DSM 639
Acd1_0346	C1	arCOG02228	S	-	Predicted membrane protein	15897380	187	Crenarchaeota	Sulfolobus solfataricus P2
Acd1_0347	C1	arCOG04273	E	HisC	Histidinol-phosphate/aromatic aminotransferase or cobyric acid decarboxylase	70606288	201	Crenarchaeota	Sulfolobus acidocaldarius DSM 639
Acd1_0348	C1	arCOG02271	K	-	Predicted transcriptional regulator, C-terminal HTH-like domain	15920343	419	Crenarchaeota	Sulfolobus tokodaii str. 7
Acd1_0349	C1	arCOG04548	C	-	Ferredoxin	15899306	300	Crenarchaeota	Sulfolobus solfataricus P2
Acd1_0350	C1	arCOG01603	C	PorG	Pyruvate:ferredoxin oxidoreductase or related 2-oxoacid:ferredoxin oxidoreductase, gamma subunit	15921824	47	Crenarchaeota	Sulfolobus tokodaii str. 7
Acd1_0351	C1	arCOG01605	C	-	Pyruvate:ferredoxin oxidoreductase or related 2-oxoacid:ferredoxin oxidoreductase, delta subunit	15898060	162	Crenarchaeota	Sulfolobus solfataricus P2
Acd1_0352	C1	arCOG01608	C	PorA	Pyruvate:ferredoxin oxidoreductase or related 2-oxoacid:ferredoxin oxidoreductase, alpha subunit	15921825	486	Crenarchaeota	Sulfolobus tokodaii str. 7
Acd1_0353	C1	arCOG01601	C	PorB	Pyruvate:ferredoxin oxidoreductase or related 2-oxoacid:ferredoxin oxidoreductase, beta subunit	15921826	107	Crenarchaeota	Sulfolobus tokodaii str. 7
Acd1_0354	C1		S		Uncharacterized protein				
Acd1_0355	C1	arCOG07198	S	0	Uncharacterized conserved membrane protein	332795818	499	Crenarchaeota	Acidianus hospitalis W1
Acd1_0356	C1	arCOG02089	M	-	S-layer domain	70606953	206	Crenarchaeota	Sulfolobus acidocaldarius DSM 639
Acd1_0357	C1	arCOG02207	L	XthA	Exonuclease III	332797485	87	Crenarchaeota	Acidianus hospitalis W1
Acd1_0358	C1	arCOG01995	R		Oligoketide cyclase/lipid transport protein family	15899686	110	Crenarchaeota	Sulfolobus solfataricus P2
Acd1_0359	C1	arCOG01951	R	-	Predicted P-loop ATPase fused to an acetyltransferase	385773685	414	Crenarchaeota	Sulfolobus islandicus HVE10/4
Acd1_0360	C1	arCOG02175	R	-	Predicted transporter of the RND superfamily	15922229	337	Crenarchaeota	Sulfolobus tokodaii str. 7
Acd1_0361	C1	arCOG04762	M	WcaG	Nucleoside-diphosphate-sugar epimerase	15922684	111	Crenarchaeota	Sulfolobus tokodaii str. 7
Acd1_0362	C1	arCOG03699	S		Uncharacterized conserved protein	146303032	285	Crenarchaeota	Metallosphaera sedula DSM 5348
Acd1_0363	C1	arCOG02691	G	ProP	Permease of the major facilitator superfamily	15922140	37.7	Crenarchaeota	Sulfolobus tokodaii str. 7
Acd1_0364	C1	arCOG00769	R	ThiJ	Putative intracellular protease/amidase	70607987	34.3	Crenarchaeota	Sulfolobus acidocaldarius DSM 639
Acd1_0365	C1	arCOG01564	J	SelB	Selenocysteine-specific translation elongation factor or SelB-II domain	15922799	422	Crenarchaeota	Sulfolobus tokodaii str. 7
tRNAGlyTCC					tRNAGlyTCC				
Acd1_0366	C1	arCOG03664	G		Glycosyltransferase	240102646	373	Euryarchaeota	Thermococcus gammatolerans EJ3
Acd1_0367	C1	arCOG01411	M	RfaG	Glycosyltransferase	15920364	55.5	Crenarchaeota	Sulfolobus tokodaii str. 7
Acd1_0368	C1	arCOG01615	S	-	Predicted membrane protein	70606075	1273	Crenarchaeota	Sulfolobus acidocaldarius DSM 639
Acd1_0369	C1	arCOG03871	N	0	Flagellar protein G	229580679	45.4	Crenarchaeota	Sulfolobus islandicus Y.G.57.14
Acd1_0370	C1	arCOG01590	I	AccC	Biotin carboxylase	15920812	376	Crenarchaeota	Sulfolobus tokodaii str. 7
Acd1_0371	C1	arCOG02699	I	AccB	Biotin carboxyl carrier protein	27877098	56.2	Crenarchaeota	Acidianus brierleyi
Acd1_0372	C1	arCOG02705	I	-	Acetyl-CoA carboxylase, carboxyltransferase component	15920810	99.8	Crenarchaeota	Sulfolobus tokodaii str. 7
Acd1_0373	C1	arCOG00929	L	Nfi	Deoxyinosine 3'endonuclease (endonuclease V)	332797774	96.3	Crenarchaeota	Acidianus hospitalis W1
Acd1_0374	C1	arCOG03413	T	CDC14	Protein-tyrosine phosphatase	15920797	1481	Crenarchaeota	Sulfolobus tokodaii str. 7
Acd1_0375	C1	arCOG00790	V	Cas4	CRISPR-associated protein Cas4, RecB family exonuclease	385772775	417	Crenarchaeota	Sulfolobus islandicus HVE10/4
Acd1_0376	C1	arCOG01369	M	WcaG	Nucleoside-diphosphate-sugar epimerase	15920365	155	Crenarchaeota	Sulfolobus tokodaii str. 7
tRNALeuGAG					tRNALeuGAG				
Acd1_0377	C1	arCOG02694	U	TatA	Sec-independent protein secretion pathway component	332797334	125	Crenarchaeota	Acidianus hospitalis W1
Acd1_0378	C1	arCOG05911	U	TatA	Sec-independent protein secretion pathway component	374631942	182	Crenarchaeota	Metallosphaera yellowstonensis MK1
Acd1_0379	C1	arCOG01919	U	TatC	Sec-independent protein secretion pathway component TatC	22204170	326	Crenarchaeota	Acidianus ambivalens
Acd1_0380	C1	arCOG01171	T	RAD55	RecA-superfamily ATPase implicated in signal transduction	284176049	424	Crenarchaeota	Sulfolobus solfataricus 98/2
Acd1_0381	C1	arCOG04349	S	-	Predicted membrane protein	15921832	211	Crenarchaeota	Sulfolobus tokodaii str. 7
Acd1_0382	C1		C		Glycolate dehydrogenase (EC 1.1.99.14), iron-sulfur subunit GlcF (fragement)	227828462	315	Crenarchaeota	Sulfolobus islandicus M.14.25
Acd1_0383	C1	arCOG01943	Q	PncA	Amidase related to nicotinamidase	332797775	384	Crenarchaeota	Acidianus hospitalis W1
Acd1_0384	C1	arCOG00423	R	-	Predicted phosphohydrolase (DHH superfamily)	15920800	88.2	Crenarchaeota	Sulfolobus tokodaii str. 7
Acd1_0385	C1	arCOG00976	O	Pcm	Protein-L-isoaspartate carboxylmethyltransferase	15899326	321	Crenarchaeota	Sulfolobus solfataricus P2
Acd1_0386	C1	arCOG00174	P	-	ABC-type anion transport system, duplicated permease component	15920813	590	Crenarchaeota	Sulfolobus tokodaii str. 7
Acd1_0387	C1	arCOG00193	P	TauB	ABC-type nitrate/sulfonate/bicarbonate transport system, ATPase component	332797790	115	Crenarchaeota	Acidianus hospitalis W1
Acd1_0388	C1	arCOG04247	E	-	Zn-dependent carboxypeptidase	15921953	591	Crenarchaeota	Sulfolobus tokodaii str. 7
Acd1_0389	C1	arCOG08322	S		Zn finger protein	332797745	233	Crenarchaeota	Acidianus hospitalis W1
Acd1_0390	C1	arCOG01189	R	AarF	Predicted unusual protein kinase	15921955	43.5	Crenarchaeota	Sulfolobus tokodaii str. 7
Acd1_0391	C1	arCOG01832	O	IbpA	Molecular chaperone (HSP20 family)	15899330	150	Crenarchaeota	Sulfolobus solfataricus P2
Acd1_0392	C1	arCOG00332	C	GlpC	Membrane associated Fe-S oxidoreductase	15921957	300	Crenarchaeota	Sulfolobus tokodaii str. 7
Acd1_0393	C1	arCOG00335	C	-	Uncharacterized conserved protein containing ferredoxin-like domain	15921958	356	Crenarchaeota	Sulfolobus tokodaii str. 7
Acd1_0394	C1	arCOG01242	L	XerC	Integrase	15921979	67	Crenarchaeota	Sulfolobus tokodaii str. 7
Acd1_0395	C1	arCOG03182	K	MarR	Transcriptional regulator, MarR family	342306706	352	Crenarchaeota	Sulfolobus tokodaii str. 7
Acd1_0396	C1	arCOG04048	F	Dcd	Deoxycytidine deaminase	70607405	885	Crenarchaeota	Sulfolobus acidocaldarius DSM 639
Acd1_0397	C1	arCOG00975	J	Sun	tRNA or rRNA cytosine-C5-methylase	70606823	369	Crenarchaeota	Sulfolobus acidocaldarius DSM 639
Acd1_0398	C1	arCOG07936	K	-	HTH containing transcriptional regulator	227830932	263	Crenarchaeota	Sulfolobus islandicus L.S.2.15
Acd1_0399	C1	arCOG02738	S	-	NifX family protein	374632238	87	Crenarchaeota	Metallosphaera yellowstonensis MK1
Acd1_0400	C1	arCOG06207	O	-	Rdx family selenoprotein	374632239	424	Crenarchaeota	Metallosphaera yellowstonensis MK1
Acd1_0401	C1	arCOG01992	T	SixA	Phosphohistidine phosphatase SixA	227826767	174	Crenarchaeota	Sulfolobus islandicus M.14.25
Acd1_0402	C1	arCOG00503	R	-	Metal-dependent hydrolase of the beta-lactamase superfamily II	15920876	186	Crenarchaeota	Sulfolobus tokodaii str. 7
Acd1_0403	C1	arCOG05546	S		Uncharacterized conserved protein	146303760	40.4	Crenarchaeota	Metallosphaera sedula DSM 5348
Acd1_0404	C1	arCOG04321	S	-	Uncharacterized conserved protein with similarity to Ribonucleotide reductase and Pyruvate formate lyase	15920428	176	Crenarchaeota	Sulfolobus tokodaii str. 7
Acd1_0405	C1	arCOG04941	T	-	ACT domain-containing protein	342306119	971	Crenarchaeota	Sulfolobus tokodaii str. 7
Acd1_0406	C1	arCOG02730	V	-	PIN domain containing protein	374631820	140	Crenarchaeota	Metallosphaera yellowstonensis MK1
Acd1_0407	C1	arCOG00815	K	AbrB	Transcriptional regulator AbrB	374631821	147	Crenarchaeota	Metallosphaera yellowstonensis MK1
Acd1_0408	C1	arCOG03854	S		Uncharacterized conserved protein	229581219	272	Crenarchaeota	Sulfolobus islandicus Y.N.15.51
Acd1_0409	C1	arCOG06002	S		Cell suface protein	227831234	251	Crenarchaeota	Sulfolobus islandicus L.S.2.15
Acd1_0410	C1	arCOG06002	S		Cell suface protein	15899842	360	Crenarchaeota	Sulfolobus solfataricus P2
Acd1_0411	C1	arCOG06003	S		Uncharacterized conserved protein	374633022	102	Crenarchaeota	Metallosphaera yellowstonensis MK1
Acd1_0412	C1	arCOG00168	P	PstA	ABC-type phosphate transport system, permease component	229581826	114	Crenarchaeota	Sulfolobus islandicus Y.N.15.51
Acd1_0413	C1	arCOG00167	P	PstC	ABC-type phosphate transport system, permease component	238620108	284	Crenarchaeota	Sulfolobus islandicus M.16.4
Acd1_0414	C1	arCOG00213	P	PstS	ABC-type phosphate transport system, periplasmic component	227830605	384	Crenarchaeota	Sulfolobus islandicus L.S.2.15
Acd1_0415	C1	arCOG00231	P	PstB	ABC-type phosphate transport system, ATPase component	15897414	392	Crenarchaeota	Sulfolobus solfataricus P2
Acd1_0416	C1	arCOG06008	R		Predicted metal permease	15899807	202	Crenarchaeota	Sulfolobus solfataricus P2
Acd1_0417	C1	arCOG04233	P	FepB	ABC-type Fe3+-hydroxamate transport system, periplasmic component	227827887	92	Crenarchaeota	Sulfolobus islandicus M.14.25
Acd1_0418	C1	arCOG01007	P	FepD	ABC-type Fe3+-siderophore transport system, permease component	284173801	41.6	Crenarchaeota	Sulfolobus solfataricus 98/2
Acd1_0419	C1	arCOG00198	P	FepC	ABC-type cobalamin/Fe3+-siderophores transport system, ATPase component	284173800	429	Crenarchaeota	Sulfolobus solfataricus 98/2
Acd1_0420	C1	arCOG03169	R	-	Predicted ATPase (AAA+ superfamily)	238619467	260	Crenarchaeota	Sulfolobus islandicus M.16.4
Acd1_0421	C1		S		Uncharacterized protein				
Acd1_0422	C1	arCOG10002	S		Uncharacterized conserved protein	374634140	104	Crenarchaeota	Metallosphaera yellowstonensis MK1
Acd1_0423	C1		S		Uncharacterized protein	374634141	163	Crenarchaeota	Metallosphaera yellowstonensis MK1
Acd1_0424	C1	arCOG07847	S		Uncharacterized conserved protein	229584960	232	Crenarchaeota	Sulfolobus islandicus M.16.27
Acd1_0425	C1	arCOG09993	S		Uncharacterized conserved protein	284175525	662	Crenarchaeota	Sulfolobus solfataricus 98/2
Acd1_0426	C1		S		Uncharacterized protein	374633367	87.8	Crenarchaeota	Metallosphaera yellowstonensis MK1
Acd1_0427	C1	arCOG00393	K	-	Predicted membrane-associated trancriptional regulator	146303959	115	Crenarchaeota	Metallosphaera sedula DSM 5348
Acd1_0428	C1	arCOG07269	S		Uncharacterized conserved protein	284998774	115	Crenarchaeota	Sulfolobus islandicus L.D.8.5
Acd1_0429	C1	arCOG06029	S		Uncharacterized conserved protein	15921310	483	Crenarchaeota	Sulfolobus tokodaii str. 7
Acd1_0430	C1	arCOG01411	M	RfaG	Glycosyltransferase	146304523	97.8	Crenarchaeota	Metallosphaera sedula DSM 5348
Acd1_0431	C1	arCOG03164	L	-	Predicted site-specific integrase-resolvase	229578345	0	Crenarchaeota	Sulfolobus islandicus Y.G.57.14
Acd1_0432	C1	arCOG00679	L	-	Transposase	227829564	273	Crenarchaeota	Sulfolobus islandicus L.S.2.15
Acd1_0433	C1	arCOG05321	S		Uncharacterized conserved protein	15922564	753	Crenarchaeota	Sulfolobus tokodaii str. 7
Acd1_0434	C1	arCOG06011	S		Uncharacterized conserved protein	15899337	45.4	Crenarchaeota	Sulfolobus solfataricus P2
Acd1_0435	C1	arCOG01492	R	-	Uncharacterized anaerobic dehydrogenase	332797682	789	Crenarchaeota	Acidianus hospitalis W1
Acd1_0436	C1	arCOG03855	S		Uncharacterized conserved protein	15899844	546	Crenarchaeota	Sulfolobus solfataricus P2
Acd1_0437	C1	arCOG04115	R	SfsA	DNA-binding protein, stimulates sugar fermentation	15921305	73.2	Crenarchaeota	Sulfolobus tokodaii str. 7
Acd1_0438	C1	arCOG03657	E		Acetolactate synthase large subunit or other thiamine pyrophosphate-requiring enzyme	332797498	338	Crenarchaeota	Acidianus hospitalis W1
Acd1_0439	C1	arCOG03000	Q	-	Gentisate 1,2-dioxygenase	332796784	211	Crenarchaeota	Acidianus hospitalis W1
Acd1_0440	C1	arCOG00264	R	-	Sulfite oxidase or related enzyme	15921054	281	Crenarchaeota	Sulfolobus tokodaii str. 7
Acd1_0441	C1	arCOG03436	V	-	CRISPR system related protein, COG1517 family	15899332	478	Crenarchaeota	Sulfolobus solfataricus P2
Acd1_0442	C1		S		Uncharacterized protein				
Acd1_0443	C1	arCOG00463	L	-	Uri superfamily endonuclease	15921224	102	Crenarchaeota	Sulfolobus tokodaii str. 7
Acd1_0444	C1	arCOG00891	L	TatD	Mg-dependent DNase	70607058	37	Crenarchaeota	Sulfolobus acidocaldarius DSM 639
Acd1_0445	C1	arCOG03738	R		P-loop NTPase superfamily protein	332797615	413	Crenarchaeota	Acidianus hospitalis W1
Acd1_0446	C1	arCOG03737	K		Transcriptional regulator of GntR family	374631221	380	Crenarchaeota	Metallosphaera yellowstonensis MK1
Acd1_0447	C1	arCOG05401	V		Ribbon-helix-helix protein, copG family	15922780	379	Crenarchaeota	Sulfolobus tokodaii str. 7
tRNAAsnGTT					tRNAAsnGTT				
Acd1_0448	C1	arCOG03853	S		Uncharacterized conserved protein	15922145	657	Crenarchaeota	Sulfolobus tokodaii str. 7
Acd1_0449	C1	arCOG01421	G	GlgP	Glucan phosphorylase	15921129	30.8	Crenarchaeota	Sulfolobus tokodaii str. 7
Acd1_0450	C1	arCOG07199	S		Uncharacterized conserved protein	15920903	68.9	Crenarchaeota	Sulfolobus tokodaii str. 7
Acd1_0451	C1	arCOG06000	S		Uncharacterized conserved protein	229578532	82	Crenarchaeota	Sulfolobus islandicus Y.G.57.14
Acd1_0452	C1	arCOG05999	S		Uncharacterized conserved protein	146303047	286	Crenarchaeota	Metallosphaera sedula DSM 5348
Acd1_0453	C1	arCOG05998	S		Uncharacterized conserved protein	70607388	275	Crenarchaeota	Sulfolobus acidocaldarius DSM 639
Acd1_0454	C1	arCOG00758	E	DadA	Glycine/D-amino acid oxidase (deaminating)	342306537	132	Crenarchaeota	Sulfolobus tokodaii str. 7

Acd1_0455	C1	arCOG03852	S		Uncharacterized conserved protein	15921924	187	Crenarchaeota	Sulfolobus tokodaii str. 7
Acd1_0456	C1	arCOG00535	H	ThiS	Sulfur transfer protein involved in thiamine biosynthesis	70607385	164	Crenarchaeota	Sulfolobus acidocaldarius DSM 639
Acd1_0457	C1		S		Uncharacterized protein	330835315	394	Crenarchaeota	Metallosphaera cuprina Ar-4
Acd1_0458	C1	arCOG01713	S	-	Uncharacterized conserved protein	327310657	195	Crenarchaeota	Thermoproteus uzoniensis 768-20
Acd1_0459	C1	arCOG00744	K		Transcriptional regulator containing HTH domain	15922155	442	Crenarchaeota	Sulfolobus tokodaii str. 7
Acd1_0460	C1	arCOG02027	P	NrfD	Formate-dependent nitrite reductase, membrane component	15922154	456	Crenarchaeota	Sulfolobus tokodaii str. 7
Acd1_0461	C1	arCOG03428	R	NrfG	TPR repeats containing protein	15922153	608	Crenarchaeota	Sulfolobus tokodaii str. 7
Acd1_0462	C1	arCOG01496	C	BisC	Molybdopterin oxidoreductase, contains molybdopterin-binding domain	15922152	499	Crenarchaeota	Sulfolobus tokodaii str. 7
Acd1_0463	C1	arCOG01500	C	HybA	Fe-S-cluster-containing dehydrogenase component	15922151	558	Crenarchaeota	Sulfolobus tokodaii str. 7
Acd1_0464	C1	arCOG01505	R	TorD	Uncharacterized component of anaerobic dehydrogenase	15922150	486	Crenarchaeota	Sulfolobus tokodaii str. 7
Acd1_0465	C1	arCOG02187	C	NapF	Ferredoxin domain containing protein	342306590	165	Crenarchaeota	Sulfolobus tokodaii str. 7
Acd1_0466	C1	arCOG06026	R		HAD superfamily hydrolase	15922148	154	Crenarchaeota	Sulfolobus tokodaii str. 7
Acd1_0467	C1	arCOG01714	S	-	Uncharacterized conserved protein	15922147	176	Crenarchaeota	Sulfolobus tokodaii str. 7
Acd1_0468	C1	arCOG01340	C	-	Acyl-CoA synthetase (NDP forming)	15921831	592	Crenarchaeota	Sulfolobus tokodaii str. 7
Acd1_0469	C1		S		Uncharacterized protein	15921915	817	Crenarchaeota	Sulfolobus tokodaii str. 7
Acd1_0470	C1	arCOG01096	S	-	Ferritin-like domain containing protein	227828966	587	Crenarchaeota	Sulfolobus islandicus M.14.25
Acd1_0471	C1	arCOG01529	I	Acs	Acyl-coenzyme A synthetase/AMP-(fatty) acid ligase	15921017	98.2	Crenarchaeota	Sulfolobus tokodaii str. 7
Acd1_0472	C1	arCOG05971	G		MFS family permease	227827438	701	Crenarchaeota	Sulfolobus islandicus M.14.25
Acd1_0473	C1	arCOG03696	C	NapH	Polyferredoxin	15922172	327	Crenarchaeota	Sulfolobus tokodaii str. 7
Acd1_0474	C1	arCOG07329	S		Uncharacterized conserved protein	385774561	352	Crenarchaeota	Sulfolobus islandicus HVE10/4
Acd1_0475	C1	arCOG00345	C	GlcD	FAD/FMN-containing dehydrogenase	332796589	134	Crenarchaeota	Acidianus hospitalis W1
Acd1_0476	C1	arCOG05380	S	-	Predicted membrane protein	332797181	197	Crenarchaeota	Acidianus hospitalis W1
Acd1_0477	C1	arCOG00194	V	CcmA	ABC-type multidrug transport system, ATPase component	15921875	315	Crenarchaeota	Sulfolobus tokodaii str. 7
Acd1_0478	C1	arCOG03849	S		Uncharacterized conserved protein	15921876	118	Crenarchaeota	Sulfolobus tokodaii str. 7
Acd1_0479	C1	arCOG02464	F	PurE	Phosphoribosylcarboxyaminoimidazole (NCAIR) mutase	70606960	39.7	Crenarchaeota	Sulfolobus acidocaldarius DSM 639
Acd1_0480	C1	arCOG03287	G	GDB1	Glycogen debranching enzyme	15921049	456	Crenarchaeota	Sulfolobus tokodaii str. 7
Acd1_0481	C1	arCOG03285	G	SGA1	Glycosyl hydrolase family 15	15921050	236	Crenarchaeota	Sulfolobus tokodaii str. 7
Acd1_0482	C1	arCOG00666	M	GCD1	N-acetylglucosamine-1-phosphate uridyltransferase	15921051	150	Crenarchaeota	Sulfolobus tokodaii str. 7
Acd1_0483	C1	arCOG03278	G	-	Glycosyl hydrolase family 57	70606970	507	Crenarchaeota	Sulfolobus acidocaldarius DSM 639
Acd1_0484	C1	arCOG00600	R	-	CBS domain	15921048	962	Crenarchaeota	Sulfolobus tokodaii str. 7
Acd1_0485	C1	arCOG01630	J	TdcF	Putative translation initiation inhibitor, yjgF family	145591472	1406	Crenarchaeota	Pyrobaculum arsenaticum DSM 13514
Acd1_0486	C1	arCOG00601	R	-	Protein containing two CBS domains (some fused to C-terminal double-stranded RNA-binding domain of RaiA family)	15922637	174	Crenarchaeota	Sulfolobus tokodaii str. 7
Acd1_0487	C1	arCOG02689	G	ProP	Permease of the major facilitator superfamily	307594709	250	Crenarchaeota	Vulcanisaeta distributa DSM 14429
Acd1_0488	C1	arCOG02881	P	ECM27	Ca2+/Na+ antiporter	332797182	293	Crenarchaeota	Acidianus hospitalis W1
Acd1_0489	C1	arCOG03016	M	WcaG	Nucleoside-diphosphate-sugar epimerase	15922179	226	Crenarchaeota	Sulfolobus tokodaii str. 7
Acd1_0490	C1	arCOG04307	J	CafA	Ribonuclease G and E	70606982	52.8	Crenarchaeota	Sulfolobus acidocaldarius DSM 639
Acd1_0491	C1	arCOG05972	S		Uncharacterized conserved protein	15921056	421	Crenarchaeota	Sulfolobus tokodaii str. 7
Acd1_0492	C1	arCOG05973	H	UbiE	Methylase involved in ubiquinone/menaquinone biosynthesis	15897943	415	Crenarchaeota	Sulfolobus solfataricus P2
Acd1_0493	C1	arCOG00631	R	-	CBS domain	15921060	160	Crenarchaeota	Sulfolobus tokodaii str. 7
Acd1_0494	C1	arCOG02869	Q	FrnE	Predicted dithiol-disulfide isomerase involved in polyketide biosynthesis	15921061	583	Crenarchaeota	Sulfolobus tokodaii str. 7
Acd1_0495	C1	arCOG00600	R	-	CBS domain	15922213	191	Crenarchaeota	Sulfolobus tokodaii str. 7
Acd1_0496	C1	arCOG02900	R	-	Uncharacterized protein containing a von Willebrand factor type A (vWA) domain	15921062	190	Crenarchaeota	Sulfolobus tokodaii str. 7
Acd1_0497	C1	arCOG05332	T	-	FHA domain containing protein	15921063	82.4	Crenarchaeota	Sulfolobus tokodaii str. 7
Acd1_0498	C1	arCOG02900	R	-	Uncharacterized protein containing a von Willebrand factor type A (vWA) domain	15897956	387	Crenarchaeota	Sulfolobus solfataricus P2
Acd1_0499	C1	arCOG01389	M	-	Glycosyltransferase	146303143	129	Crenarchaeota	Metallosphaera sedula DSM 5348
Acd1_0500	C1	arCOG01698	E	LeuC	Homoaconitate hydratase/3-isopropylmalate dehydratase large subunit family protein	15920819	379	Crenarchaeota	Sulfolobus tokodaii str. 7
Acd1_0501	C1	arCOG02230	E	LeuD	3-isopropylmalate dehydratase small subunit	15920820	128	Crenarchaeota	Sulfolobus tokodaii str. 7
Acd1_0502	C1	arCOG07348	S		Uncharacterized conserved protein	15921948	45.1	Crenarchaeota	Sulfolobus tokodaii str. 7
Acd1_0504	C1		S		Uncharacterized protein				
Acd1_0503	C1	arCOG03660	G	ProP	Permease of the major facilitator superfamily	15898686	161	Crenarchaeota	Sulfolobus solfataricus P2
Acd1_0505	C1	arCOG00271	G	RhaT	permease of the drug/metabolite transporter (DMT) superfamily	15921909	221	Crenarchaeota	Sulfolobus tokodaii str. 7
Acd1_0506	C1	arCOG02266	P	CorA	Mg2+ and Co2+ transporter	15898148	358	Crenarchaeota	Sulfolobus solfataricus P2
Acd1_0507	C1	arCOG01554	C	NuoB	F420H2 dehydrogenase subunit, related to NADH:ubiquinone oxidoreductase 20 kD subunit	146304760	832	Crenarchaeota	Metallosphaera sedula DSM 5348
Acd1_0508	C1	arCOG01952	J	SUA5	Putative translation factor (SUA5)	15921819	201	Crenarchaeota	Sulfolobus tokodaii str. 7
Acd1_0509	C1	arCOG01940	H	BirA	Biotin-(acetyl-CoA carboxylase) ligase	70607375	80.9	Crenarchaeota	Sulfolobus acidocaldarius DSM 639
Acd1_0510	C1	arCOG02053	T	UspA	Nucleotide-binding protein, UspA family	15921817	441	Crenarchaeota	Sulfolobus tokodaii str. 7
Acd1_0511	C1	arCOG00322	R	TldD	Predicted Zn-dependent protease or their inactivated homolog	330834144	312	Crenarchaeota	Metallosphaera cuprina Ar-4
Acd1_0512	C1	arCOG00321	R	TldD	Predicted Zn-dependent protease or their inactivated homolog	332796771	81.6	Crenarchaeota	Acidianus hospitalis W1
Acd1_0513	C1	arCOG01351	E	ThrA	Homoserine dehydrogenase	342306522	164	Crenarchaeota	Sulfolobus tokodaii str. 7
Acd1_0514	C1	arCOG00479	O	CyoE	Polyprenyltransferase (cytochrome oxidase assembly factor)	15921811	304	Crenarchaeota	Sulfolobus tokodaii str. 7
Acd1_0515	C1	arCOG07286	S		Uncharacterized conserved protein	15897565	941	Crenarchaeota	Sulfolobus solfataricus P2
Acd1_0517	C1		S		Uncharacterized protein				
Acd1_0516	C1	arCOG00946	O	PflA	Pyruvate-formate lyase-activating enzyme	15921808	341	Crenarchaeota	Sulfolobus tokodaii str. 7
Acd1_0518	C1	arCOG00249	I	FadB	3-hydroxyacyl-CoA dehydrogenase	15921809	454	Crenarchaeota	Sulfolobus tokodaii str. 7
Acd1_0519	C1	arCOG07226	S		Uncharacterized conserved protein	15921807	342	Crenarchaeota	Sulfolobus tokodaii str. 7
Acd1_0520	C1	arCOG04298	L	-	Predicted adenosine-specific kinase	15921805	530	Crenarchaeota	Sulfolobus tokodaii str. 7
Acd1_0521	C1	arCOG05997	S		Uncharacterized conserved protein	70607362	205	Crenarchaeota	Sulfolobus acidocaldarius DSM 639
Acd1_0522	C1	arCOG01091	S	-	Uncharacterized membrane protein	15921803	150	Crenarchaeota	Sulfolobus tokodaii str. 7
Acd1_0523	C1		S		Uncharacterized protein	70607360	327	Crenarchaeota	Sulfolobus acidocaldarius DSM 639
Acd1_0524	C1	arCOG00251	I	FadB	3-hydroxyacyl-CoA dehydrogenase	15921801	523	Crenarchaeota	Sulfolobus tokodaii str. 7
Acd1_0525	C1	arCOG04318	J	-	Predicted RNA-binding protein of the translin family	146304740	303	Crenarchaeota	Metallosphaera sedula DSM 5348
Acd1_0526	C1	arCOG01589	H	RimK	Glutathione synthase/glutaminyl transferase/alpha-L-glutamate ligase	15921799	369	Crenarchaeota	Sulfolobus tokodaii str. 7
Acd1_0527	C1	arCOG01594	E	CarB	Carbamoylphosphate synthase large subunit	332796788	288	Crenarchaeota	Acidianus hospitalis W1
Acd1_0528	C1	arCOG00064	E	CarA	Carbamoylphosphate synthase small subunit	15921797	635	Crenarchaeota	Sulfolobus tokodaii str. 7
Acd1_0529	C1	arCOG01748	E	ArgH	Argininosuccinate lyase	374634004	32.3	Crenarchaeota	Metallosphaera yellowstonensis MK1
Acd1_0530	C1	arCOG00112	E	ArgG	Argininosuccinate synthase	374634005	99	Crenarchaeota	Metallosphaera yellowstonensis MK1
Acd1_0531	C1	arCOG01009	V	-	Ribbon-helix-helix protein, copG family	18312663	105	Crenarchaeota	Pyrobaculum aerophilum str. IM2
Acd1_0532	C1	arCOG05996	S		Uncharacterized conserved protein	15897550	735	Crenarchaeota	Sulfolobus solfataricus P2
Acd1_0533	C1	arCOG00639	F	PurM	Phosphoribosylaminoimidazole (AIR) synthetase	15921793	382	Crenarchaeota	Sulfolobus tokodaii str. 7
Acd1_0534	C1	arCOG04415	F	PurD	Phosphoribosylamine-glycine ligase	15921792	42.7	Crenarchaeota	Sulfolobus tokodaii str. 7
Acd1_0535	C1	arCOG00094	F	PurF	Glutamine phosphoribosylpyrophosphate amidotransferase	15921791	286	Crenarchaeota	Sulfolobus tokodaii str. 7
Acd1_0536	C1	arCOG00093	F	PurF	Glutamine phosphoribosylpyrophosphate amidotransferase	332796796	207	Crenarchaeota	Acidianus hospitalis W1
Acd1_0537	C1	arCOG00641	F	PurL	Phosphoribosylformylglycinamidine (FGAM) synthase, synthetase domain	15921789	221	Crenarchaeota	Sulfolobus tokodaii str. 7
Acd1_0538	C1	arCOG00102	F	PurL	Phosphoribosylformylglycinamidine (FGAM) synthase, glutamine amidotransferase domain	342306512	109	Crenarchaeota	Sulfolobus tokodaii str. 7
Acd1_0539	C1	arCOG04462	F	PurS	Phosphoribosylformylglycinamidine (FGAM) synthase, PurS component	15921787	156	Crenarchaeota	Sulfolobus tokodaii str. 7
Acd1_0540	C1	arCOG04421	F	PurC	Phosphoribosylaminoimidazolesuccinocarboxamide (SAICAR) synthase	15921786	672	Crenarchaeota	Sulfolobus tokodaii str. 7
Acd1_0541	C1	arCOG01136	R	ARC1	EMAP domain RNA-binding protein	15921785	215	Crenarchaeota	Sulfolobus tokodaii str. 7
Acd1_0542	C1	arCOG00352	R	-	Predicted GTPase	15921784	49.7	Crenarchaeota	Sulfolobus tokodaii str. 7
Acd1_0543	C1	arCOG02173	O	NrdG	Organic radical activating enzyme	15921783	458	Crenarchaeota	Sulfolobus tokodaii str. 7
Acd1_0544	C1	arCOG04156	J	TYW3	Wybutosine (yW) biosynthesis enzyme	15921782	139	Crenarchaeota	Sulfolobus tokodaii str. 7
Acd1_0545	C1	arCOG07225	S		Uncharacterized conserved protein	15921781	415	Crenarchaeota	Sulfolobus tokodaii str. 7
Acd1_0546	C1	arCOG01068	C	Lpd	Pyruvate/2-oxoglutarate dehydrogenase complex, dihydrolipoamide dehydrogenase (E3) component or related enzyme	146304719	473	Crenarchaeota	Metallosphaera sedula DSM 5348
Acd1_0547	C1	arCOG00454	D	ESCRTIII	Crenarchaeal division protein ESCRT-III	15921778	303	Crenarchaeota	Sulfolobus tokodaii str. 7
Acd1_0548	C1	arCOG00318	P	PhoU	Phosphate uptake regulator	374634024	738	Crenarchaeota	Metallosphaera yellowstonensis MK1
Acd1_0549	C1	arCOG03882	R		RadC related protein, contains PAD1/JAB1 superfamily protase domain and ENDO3 nuclease domain	332796808	952	Crenarchaeota	Acidianus hospitalis W1
Acd1_0550	C1	arCOG00081	F	PyrF	Orotidine-5'-phosphate decarboxylase	332796809	178	Crenarchaeota	Acidianus hospitalis W1
Acd1_0551	C1	arCOG00029	F	PyrE	Orotate phosphoribosyltransferase	15921774	358	Crenarchaeota	Sulfolobus tokodaii str. 7
Acd1_0552	C1	arCOG00911	F	PyrB	Aspartate carbamoyltransferase, catalytic chain	15921773	216	Crenarchaeota	Sulfolobus tokodaii str. 7
Acd1_0553	C1	arCOG04229	F	PyrI	Aspartate carbamoyltransferase, regulatory subunit	15897529	526	Crenarchaeota	Sulfolobus solfataricus P2
Acd1_0554	C1	arCOG02199	H	UbiB	2-polyprenylphenol hydroxylase or related flavodoxin oxidoreductase	332796813	463	Crenarchaeota	Acidianus hospitalis W1
Acd1_0555	C1	arCOG00689	F	PyrC	Dihydroorotase or related cyclic amidohydrolase	22001887	419	Crenarchaeota	Sulfolobus tokodaii str. 7
Acd1_0556	C1	arCOG00603	F	PyrD	Dihydroorotate dehydrogenase	70607328	301	Crenarchaeota	Sulfolobus acidocaldarius DSM 639
Acd1_0557	C1	arCOG05995	S		Uncharacterized conserved protein	15921768	164	Crenarchaeota	Sulfolobus tokodaii str. 7
Acd1_0558	C1	arCOG05994	S		Uncharacterized conserved protein	15921767	107	Crenarchaeota	Sulfolobus tokodaii str. 7
Acd1_0559	C1	arCOG01997	U	MarC	Multiple antibiotic transporter	15921766	131	Crenarchaeota	Sulfolobus tokodaii str. 7
Acd1_0560	C1	arCOG01580	K	Lrp	Transcriptional regulator, IclR family	15921765	613	Crenarchaeota	Sulfolobus tokodaii str. 7
Acd1_0561	C1	arCOG00580	K	RPB9	DNA-directed RNA polymerase, subunit M/Transcription elongation factor TFIIS	15921764	113	Crenarchaeota	Sulfolobus tokodaii str. 7
Acd1_0562	C1	arCOG08337	J		Ribosome associated protein L47a	343773666	36.2	Crenarchaeota	Sulfolobus tokodaii str. 7
Acd1_0563	C1	arCOG01357	C	-	Radical SAM superfamily enzyme	15921762	117	Crenarchaeota	Sulfolobus tokodaii str. 7
Acd1_0564	C1	arCOG00403	J	SerS	Seryl-tRNA synthetase	332796823	479	Crenarchaeota	Acidianus hospitalis W1
Acd1_0565	C1	arCOG01845	R	PaaD	Predicted metal-sulfur cluster biosynthetic enzyme	15921760	92.8	Crenarchaeota	Sulfolobus tokodaii str. 7
Acd1_0566	C1	arCOG02676	E	HisI	Phosphoribosyl-AMP cyclohydrolase	70607318	108	Crenarchaeota	Sulfolobus acidocaldarius DSM 639
Acd1_0567	C1	arCOG00089	E	HisH	Glutamine amidotransferase	332796826	36.2	Crenarchaeota	Acidianus hospitalis W1
Acd1_0568	C1	arCOG02677	E	HisI	Phosphoribosyl-ATP pyrophosphohydrolase	146304696	168	Crenarchaeota	Metallosphaera sedula DSM 5348
Acd1_0569	C1	arCOG04352	E	HisD	Histidinol dehydrogenase	332796828	449	Crenarchaeota	Acidianus hospitalis W1
Acd1_0570	C1	arCOG00617	E	HisF	Imidazoleglycerol-phosphate synthase	15921755	300	Crenarchaeota	Sulfolobus tokodaii str. 7
Acd1_0571	C1	arCOG04398	E	HisB	Imidazoleglycerol-phosphate dehydratase	374634047	904	Crenarchaeota	Metallosphaera yellowstonensis MK1
Acd1_0572	C1	arCOG00618	E	HisA	Phosphoribosylformimino-5-aminoimidazole carboxamide ribonucleotide (ProFAR) isomerase	15921753	123	Crenarchaeota	Sulfolobus tokodaii str. 7
Acd1_0573	C1	arCOG02208	E	HisG	ATP phosphoribosyltransferase	70607311	209	Crenarchaeota	Sulfolobus acidocaldarius DSM 639
Acd1_0574	C1	arCOG04273	E	HisC	Histidinol-phosphate/aromatic aminotransferase or cobyric acid decarboxylase	363548423	93.6	Crenarchaeota	Sulfolobus tokodaii str. 7
Acd1_0575	C1	arCOG05993	K		Transcriptional regulator, contains wHTH domain	15921750	741	Crenarchaeota	Sulfolobus tokodaii str. 7
Acd1_0576	C1	arCOG00809	J	LeuS	Leucyl-tRNA synthetase	48428670	330	Crenarchaeota	Sulfolobus tokodaii str. 7
Acd1_0577	C1	arCOG00489	H	-	cob(I)alamin adenosyltransferase	15921748	234	Crenarchaeota	Sulfolobus tokodaii str. 7
Acd1_0578	C1	arCOG00042	D	MesJ	tRNA(Ile)-lysidine synthase MesJ	15921747	137	Crenarchaeota	Sulfolobus tokodaii str. 7
Acd1_0579	C1	arCOG04140	S	-	Uncharacterized conserved protein	146304685	311	Crenarchaeota	Metallosphaera sedula DSM 5348
Acd1_0580	C1	arCOG00279	E	SpeD	S-adenosylmethionine decarboxylase/arginine decarboxylase	15921745	69.7	Crenarchaeota	Sulfolobus tokodaii str. 7
Acd1_0581	C1	arCOG00570	C	FixC	Dehydrogenase (flavoprotein)	70607303	593	Crenarchaeota	Sulfolobus acidocaldarius DSM 639
Acd1_0582	C1	arCOG08842	K	PhnF	Transcriptional regulator, GntR family	70607302	241	Crenarchaeota	Sulfolobus acidocaldarius DSM 639

Acd1_0583	C1	arCOG00476	H	UbiA	4-hydroxybenzoate polyprenyltransferase or related prenyltransferase	15921742	176	Crenarchaeota	Sulfolobus tokodaii str. 7
Acd1_0584	C1	arCOG05992	S		Uncharacterized conserved protein	70607300	392	Crenarchaeota	Sulfolobus acidocaldarius DSM 639
Acd1_0585	C1	arCOG00350	R	-	Predicted GTPase	227827818	315	Crenarchaeota	Sulfolobus islandicus M.14.25
Acd1_0586	C1	arCOG01667	S	-	Zinc finger domain containing protein (CDGSH-type)	332797198	65.5	Crenarchaeota	Acidianus hospitalis W1
Acd1_0587	C1	arCOG04046	R	-	Predicted phosphatase	15921739	665	Crenarchaeota	Sulfolobus tokodaii str. 7
Acd1_0588	C1		S		Uncharacterized protein				
Acd1_0589	C1	arCOG01998	E	IlvB	Acetolactate synthase large subunit or other thiamine pyrophosphate-requiring enzyme	15921738	201	Crenarchaeota	Sulfolobus tokodaii str. 7
Acd1_0590	C1	arCOG05991	E		ACT domain	15897496	322	Crenarchaeota	Sulfolobus solfataricus P2
Acd1_0591	C1	arCOG04465	E	IlvC	Ketol-acid reductoisomerase	15921736	373	Crenarchaeota	Sulfolobus tokodaii str. 7
Acd1_0592	C1	arCOG00919	L	-	Holliday junction resolvase	342306492	60.5	Crenarchaeota	Sulfolobus tokodaii str. 7
Acd1_0593	C1	arCOG04116	R	-	ATPase (PilT family)	332796849	176	Crenarchaeota	Acidianus hospitalis W1
Acd1_0594	C1	arCOG00034	H	PDX2	Predicted glutamine amidotransferase involved in pyridoxine biosynthesis	15921733	114	Crenarchaeota	Sulfolobus tokodaii str. 7
Acd1_0595	C1	arCOG04075	H	SNZ1	Pyridoxine biosynthesis enzyme	374634069	348	Crenarchaeota	Metallosphaera yellowstonensis MK1
Acd1_0596	C1	arCOG04305	J	RPS26B	Ribosomal protein S26	161751093	202	Crenarchaeota	Sulfolobus tokodaii str. 7
Acd1_0597	C1	arCOG00402	J	ProS	Prolyl-tRNA synthetase	70607289	290	Crenarchaeota	Sulfolobus acidocaldarius DSM 639
Acd1_0598	C1	arCOG02455	C	AtpK	Archaeal/vacuolar-type Na+/H+-ATPase, subunit K	70607288	238	Crenarchaeota	Sulfolobus acidocaldarius DSM 639
Acd1_0599	C1	arCOG07285	C	NtpF	Archaeal/vacuolar-type H+-ATPase subunit H	15897487	893	Crenarchaeota	Sulfolobus solfataricus P2
Acd1_0600	C1	arCOG04101	C	NtpD	Archaeal/vacuolar-type H+-ATPase subunit D	15921727	77.8	Crenarchaeota	Sulfolobus tokodaii str. 7
Acd1_0601	C1	arCOG00865	C	NtpB	Archaeal/vacuolar-type H+-ATPase subunit B	15921726	110	Crenarchaeota	Sulfolobus tokodaii str. 7
Acd1_0602	C1	arCOG00868	C	NtpA	Archaeal/vacuolar-type H+-ATPase subunit A	15921725	310	Crenarchaeota	Sulfolobus tokodaii str. 7
Acd1_0603	C1	arCOG00869	C	NtpE	Archaeal/vacuolar-type H+-ATPase subunit E	330834233	212	Crenarchaeota	Metallosphaera cuprina Ar-4
Acd1_0604	C1	arCOG04102	C	NtpG	Archaeal/vacuolar-type H+-ATPase subunit F	227827835	46.6	Crenarchaeota	Sulfolobus islandicus M.14.25
Acd1_0605	C1	arCOG04138	C	NtpI	Archaeal/vacuolar-type H+-ATPase subunit I	15921722	262	Crenarchaeota	Sulfolobus tokodaii str. 7
Acd1_0606	C1	arCOG00810	J	MetG	Methionyl-tRNA synthetase	15921721	65.5	Crenarchaeota	Sulfolobus tokodaii str. 7
Acd1_0607	C1	arCOG03013	L	PRI2	Eukaryotic-type DNA primase, large subunit	332796862	331	Crenarchaeota	Acidianus hospitalis W1
Acd1_0608	C1	arCOG00670	I	PgsA	Phosphatidylglycerophosphate synthase	330834238	0	Crenarchaeota	Metallosphaera cuprina Ar-4
Acd1_0609	C1	arCOG04283	S		Uncharacterized conserved protein	15921718	555	Crenarchaeota	Sulfolobus tokodaii str. 7
Acd1_0610	C1	arCOG01631	Q		SAM-dependent methyltransferase	332796865	385	Crenarchaeota	Acidianus hospitalis W1
Acd1_0611	C1	arCOG04251	S	-	Uncharacterized conserved protein	15921716	520	Crenarchaeota	Sulfolobus tokodaii str. 7
tRNAArgGCG					tRNAArgGCG				
Acd1_0612	C1	arCOG00328	L	PolB	DNA polymerase elongation subunit (family B)	15921715	75.1	Crenarchaeota	Sulfolobus tokodaii str. 7
Acd1_0613	C1	arCOG01397	M	WcaA	Glycosyltransferase	171184954	939	Crenarchaeota	Pyrobaculum neutrophilum V24Sta
Acd1_0614	C1	arCOG07214	S		Uncharacterized conserved protein	146304383	735	Crenarchaeota	Metallosphaera sedula DSM 5348
Acd1_0615	C1	arCOG01751	J	RPL8A	Ribosomal protein L7AE	15921713	285	Crenarchaeota	Sulfolobus tokodaii str. 7
Acd1_0616	C1	arCOG04302	J	GlnS	Glutamyl- or glutaminyl-tRNA synthetase	332796279	514	Crenarchaeota	Acidianus hospitalis W1
Acd1_0617	C1	arCOG01213	R	Cof	HAD superfamily hydrolase	342306475	661	Crenarchaeota	Sulfolobus tokodaii str. 7
Acd1_0618	C1	arCOG04430	R	-	HD superfamily phosphohydrolase	15921710	585	Crenarchaeota	Sulfolobus tokodaii str. 7
5S RNA					5S RNA				
Acd1_0619	C1	arCOG03770	S		Uncharacterized conserved protein	70607258	186	Crenarchaeota	Sulfolobus acidocaldarius DSM 639
Acd1_0620	C1	arCOG04181	S	-	Uncharacterized conserved protein	284173403	243	Crenarchaeota	Sulfolobus solfataricus 98/2
Acd1_0621	C1	arCOG01001	J	Map	Methionine aminopeptidase	146304873	271	Crenarchaeota	Metallosphaera sedula DSM 5348
Acd1_0622	C1	arCOG00497	R	-	Zn-dependent hydrolase of the beta-lactamase fold	15921707	538	Crenarchaeota	Sulfolobus tokodaii str. 7
Acd1_0623	C1	arCOG00410	J	PheS	Phenylalanyl-tRNA synthetase alpha subunit	15921705	408	Crenarchaeota	Sulfolobus tokodaii str. 7
Acd1_0624	C1	arCOG00412	J	PheT	Phenylalanyl-tRNA synthetase beta subunit	15921704	880	Crenarchaeota	Sulfolobus tokodaii str. 7
Acd1_0625	C1	arCOG01648	R	MhpC	Alpha/beta superfamily hydrolase	15921703	341	Crenarchaeota	Sulfolobus tokodaii str. 7
Acd1_0626	C1	arCOG00215	R	CinA	Predicted nucleotide-utilizing enzyme related to molybdopterin-biosynthesis enzyme MoeA	332796269	351	Crenarchaeota	Acidianus hospitalis W1
Acd1_0627	C1	arCOG00492	K	ARO8	Transcriptional regulators containing a DNA-binding HTH domain and an aminotransferase domain (MocR family)	15921700	289	Crenarchaeota	Sulfolobus tokodaii str. 7
Acd1_0628	C1	arCOG05990	C	Qor	NADPH:quinone reductase or related Zn-dependent oxidoreductase	332796267	107	Crenarchaeota	Acidianus hospitalis W1
Acd1_0629	C1	arCOG05989	K		Transcriptional regulator, xre family	332796266	73.9	Crenarchaeota	Acidianus hospitalis W1
Acd1_0630	C1	arCOG05988	S		Uncharacterized conserved protein	15921697	183	Crenarchaeota	Sulfolobus tokodaii str. 7
Acd1_0631	C1	arCOG04166	S	-	Uncharacterized conserved protein	15897069	55.1	Crenarchaeota	Sulfolobus solfataricus P2
Acd1_0632	C1	arCOG00610	K	-	Predicted transcriptional regulator, contains C-terminal CBS domains	70607246	82	Crenarchaeota	Sulfolobus acidocaldarius DSM 639
Acd1_0633	C1	arCOG01138	R	-	Predicted metal-dependent protease of the PAD1/JAB1 superfamily	146304861	194	Crenarchaeota	Metallosphaera sedula DSM 5348
Acd1_0634	C1	arCOG00557	R	Lhr	Lhr-like helicase	15921693	65.1	Crenarchaeota	Sulfolobus tokodaii str. 7
Acd1_0635	C1	arCOG01391	M	-	Glycosyltransferase	332796260	424	Crenarchaeota	Acidianus hospitalis W1
Acd1_0636	C1	arCOG01875	K		ParB-like nuclease domain	70607242	172	Crenarchaeota	Sulfolobus acidocaldarius DSM 639
Acd1_0637	C1	arCOG00459	L	Nth	EndoIII-related endonuclease	15921690	285	Crenarchaeota	Sulfolobus tokodaii str. 7
Acd1_0638	C1	arCOG07276	N		Pilin/Flagellin	227831025	135	Crenarchaeota	Sulfolobus islandicus L.S.2.15
Acd1_0639	C1	arCOG05987	N	0	Pilin/Flagellin	70607240	41.6	Crenarchaeota	Sulfolobus acidocaldarius DSM 639
Acd1_0640	C1	arCOG01815	N	TadC	Flp pilus assembly protein TadC	70607239	190	Crenarchaeota	Sulfolobus acidocaldarius DSM 639
Acd1_0641	C1		S		Uncharacterized protein				
Acd1_0642	C1	arCOG01817	N	VirB11	Type IV secretory pathway, VirB11 component, or related ATPase involved in archaeal flagella biosynthesis	15921686	146	Crenarchaeota	Sulfolobus tokodaii str. 7
Acd1_0643	C1	arCOG05986	N		Predicted component of pili system	15921685	97.4	Crenarchaeota	Sulfolobus tokodaii str. 7
Acd1_0644	C1	arCOG05985	R		Predicted periplasmic solute-binding protein	15921684	56.6	Crenarchaeota	Sulfolobus tokodaii str. 7
Acd1_0645	C1	arCOG05348	R	HcaD	NAD(FAD)-dependent dehydrogenase	15897084	247	Crenarchaeota	Sulfolobus solfataricus P2
Acd1_0646	C1	arCOG05348	R	HcaD	NAD(FAD)-dependent dehydrogenase	227828260	600	Crenarchaeota	Sulfolobus islandicus M.14.25
Acd1_0647	C1	arCOG01241	L	XerC	Integrase	15921682	534	Crenarchaeota	Sulfolobus tokodaii str. 7
Acd1_0648	C1	arCOG01849	R	PaaY	Isoleucine patch superfamily protein	385773702	89.7	Crenarchaeota	Sulfolobus islandicus HVE10/4
Acd1_0649	C1	arCOG00986	J	Sun	RNA methylase associated PUA domain	15921679	543	Crenarchaeota	Sulfolobus tokodaii str. 7
Acd1_0650	C1	arCOG01026	R	-	Predicted sugar kinase	15921678	33.1	Crenarchaeota	Sulfolobus tokodaii str. 7
Acd1_0651	C1	arCOG00857	I	CaiC	Acyl-CoA synthetase (AMP-forming)/AMP-acid ligase II	15921677	60.5	Crenarchaeota	Sulfolobus tokodaii str. 7
Acd1_0652	C1	arCOG01972	O	TrxA	Thiol-disulfide isomerase or thioredoxin	342306464	472	Crenarchaeota	Sulfolobus tokodaii str. 7
Acd1_0653	C1	arCOG05705	G	MalK	ABC-type sugar transport system, ATPase component	332796952	278	Crenarchaeota	Acidianus hospitalis W1
Acd1_0654	C1	arCOG01909	E	GlnA	Glutamine synthetase	332796953	615	Crenarchaeota	Acidianus hospitalis W1
Acd1_0655	C1	arCOG04542	H	FolE	GTP cyclohydrolase I	146304374	239	Crenarchaeota	Metallosphaera sedula DSM 5348
Acd1_0656	C1	arCOG01000	E	PepP	Xaa-Pro aminopeptidase	332796956	212	Crenarchaeota	Acidianus hospitalis W1
Acd1_0657	C1	arCOG00930	H	MoaA	Molybdenum cofactor biosynthesis enzyme	15897297	553	Crenarchaeota	Sulfolobus solfataricus P2
Acd1_0658	C1	arCOG06088	S		Uncharacterized conserved protein	332796958	152	Crenarchaeota	Acidianus hospitalis W1
Acd1_0659	C1	arCOG01186	T	-	Mn2+-dependent serine/threonine protein kinase	146304371	288	Crenarchaeota	Metallosphaera sedula DSM 5348
Acd1_0660	C1	arCOG01430	E	CysK	Cysteine synthase	70607219	147	Crenarchaeota	Sulfolobus acidocaldarius DSM 639
Acd1_0661	C1	arCOG02062	O	SirA	Predicted redox protein, regulator of disulfide bond formation	15921668	551	Crenarchaeota	Sulfolobus tokodaii str. 7
Acd1_0662	C1	arCOG02067	R	-	Predicted peroxiredoxin	332796962	94.4	Crenarchaeota	Acidianus hospitalis W1
Acd1_0663	C1	arCOG04321	S	-	Uncharacterized conserved protein with similarity to Ribonucleotide reductase and Pyruvate formate lyase	284174542	551	Crenarchaeota	Sulfolobus solfataricus 98/2
Acd1_0664	C1	arCOG00318	P	PhoU	Phosphate uptake regulator	374633603	233	Crenarchaeota	Metallosphaera yellowstonensis MK1
Acd1_0665	C1	arCOG01481	H	PncB	Nicotinic acid phosphoribosyltransferase	15921664	561	Crenarchaeota	Sulfolobus tokodaii str. 7
Acd1_0666	C1	arCOG01344	J	RPS19A	Ribosomal protein S19E (S16A)	15921663	498	Crenarchaeota	Sulfolobus tokodaii str. 7
Acd1_0667	C1	arCOG04179	R	-	DNA-binding protein	374633606	32.7	Crenarchaeota	Metallosphaera yellowstonensis MK1
Acd1_0668	C1	arCOG04177	J	RPL39	Ribosomal protein L39E	15897288	246	Crenarchaeota	Sulfolobus solfataricus P2
Acd1_0669	C1	arCOG04473	J	RPL31A	Ribosomal protein L31E	15921660	126	Crenarchaeota	Sulfolobus tokodaii str. 7
Acd1_0670	C1	arCOG04176	J	TIF6	Translation initiation factor 6 (eIF-6)	15921658	73.9	Crenarchaeota	Sulfolobus tokodaii str. 7
Acd1_0671	C1	arCOG04175	J	RPL20A	Ribosomal protein L20A (L18A)	332796971	169	Crenarchaeota	Acidianus hospitalis W1
Acd1_0672	C1	arCOG01341	O	GIM5	Predicted prefoldin, molecular chaperone implicated in de novo protein folding	146304393	257	Crenarchaeota	Metallosphaera sedula DSM 5348
Acd1_0673	C1	arCOG01227	U	FtsY	Signal recognition particle GTPase	60623873	301	Crenarchaeota	Sulfolobus tokodaii str. 7
Acd1_0674	C1	arCOG02204	U	Sss1	Preprotein translocase subunit Sss1	15921654	36.6	Crenarchaeota	Sulfolobus tokodaii str. 7
Acd1_0675	C1	arCOG01920	K	NusG	Transcription antiterminator NusG	15921653	191	Crenarchaeota	Sulfolobus tokodaii str. 7
Acd1_0676	C1	arCOG04372	J	RplK	Ribosomal protein L11	227830761	362	Crenarchaeota	Sulfolobus islandicus L.S.2.15
Acd1_0677	C1	arCOG04289	J	RplA	Ribosomal protein L1	15921651	190	Crenarchaeota	Sulfolobus tokodaii str. 7
Acd1_0678	C1	arCOG04288	J	RplJ	Ribosomal protein L10	15897278	153	Crenarchaeota	Sulfolobus solfataricus P2
Acd1_0679	C1	arCOG04287	J	RPP1A	Ribosomal protein L12E/L44/L45/RPP1/RPP2	15921649	667	Crenarchaeota	Sulfolobus tokodaii str. 7
Acd1_0680	C1	arCOG01255	J	AlaS	Alanyl-tRNA synthetase	15921648	553	Crenarchaeota	Sulfolobus tokodaii str. 7
Acd1_0681	C1	arCOG03229	S	-	Uncharacterized conserved protein	15921647	33.1	Crenarchaeota	Sulfolobus tokodaii str. 7
tRNAArgCCT					tRNAArgCCT				
Acd1_0682	C1	arCOG00288	C	NfnB	Nitroreductase	15920926	639	Crenarchaeota	Sulfolobus tokodaii str. 7
tRNAMetCAT					tRNAMetCAT				
Acd1_0683	C1	arCOG09929	S		Uncharacterized conserved protein	385775517	223	Crenarchaeota	Sulfolobus islandicus REY15A
Acd1_0684	C1	arCOG01663	K	RelE	Cytotoxic translational repressor of toxin-antitoxin stability system	15920339	358	Crenarchaeota	Sulfolobus tokodaii str. 7
Acd1_0685	C1	arCOG07934	S	0	Uncharacterized protein	332796911	253	Crenarchaeota	Acidianus hospitalis W1
Acd1_0686	C1		S		Uncharacterized protein	374632512	844	Crenarchaeota	Metallosphaera yellowstonensis MK1
Acd1_0687	C1		S		Uncharacterized protein	374632513	493	Crenarchaeota	Metallosphaera yellowstonensis MK1
Acd1_0688	C1		S		Uncharacterized protein	374632514	825	Crenarchaeota	Metallosphaera yellowstonensis MK1
Acd1_0689	C1	arCOG00143	G	ProP	Permease of the major facilitator superfamily	284998068	859	Crenarchaeota	Sulfolobus islandicus L.D.8.5
Acd1_0690	C1	arCOG06513	R	-	Predicted ester cyclase	257075598	95.5	Euryarchaeota	Ferroplasma acidarmanus fer1
Acd1_0691	C1		S		Uncharacterized protein	15898320	492	Crenarchaeota	Sulfolobus solfataricus P2
Acd1_0692	C1	arCOG04708	S	-	Uncharacterized conserved protein	385773424	119	Crenarchaeota	Sulfolobus islandicus HVE10/4
Acd1_0693	C1		S		Uncharacterized protein	229578377	362	Crenarchaeota	Sulfolobus islandicus Y.G.57.14
Acd1_0694	C1		S		Uncharacterized protein	284996978	167	Crenarchaeota	Sulfolobus islandicus L.D.8.5
Acd1_0695	C1		S		Uncharacterized protein				
Acd1_0696	C1		S		Uncharacterized protein				
Acd1_0697	C1	arCOG07309	V	PlrA	Plasmid regulatory DNA-binding protein	228288725	196	Crenarchaeota	Sulfolobus islandicus Y.N.15.51
Acd1_0698	C1		S		Uncharacterized protein	374631839	301	Crenarchaeota	Metallosphaera yellowstonensis MK1
Acd1_0699	C1	arCOG07283	S		Uncharacterized conserved protein	332796999	84.3	Crenarchaeota	Acidianus hospitalis W1
Acd1_0700	C1	arCOG02632	V	HsdM	Type I restriction-modification system methyltransferase subunit	296242623	803	Crenarchaeota	Thermosphaera aggregans DSM 11486
Acd1_0701	C1	arCOG00792	L	-	Superfamily I DNA/RNA helicase	374328315	312	Crenarchaeota	Pyrobaculum sp. 1860
Acd1_0702	C1		S		Uncharacterized protein	374326890	79.7	Crenarchaeota	Pyrobaculum sp. 1860
Acd1_0703	C1	arCOG00555	R	-	Distinct helicase family with a unique C-terminal domain including a metal-binding cysteine cluster	374326889	41.6	Crenarchaeota	Pyrobaculum sp. 1860
Acd1_0704	C1		S		Uncharacterized protein	30575704	607	environmental samples	uncultured bacterium
Acd1_0705	C1	arCOG02218	V		RHH/CopG DNA binding protein	332797413	309	Crenarchaeota	Acidianus hospitalis W1
Acd1_0706	C1	arCOG00729	V	-	PIN domain containing protein	332797412	103	Crenarchaeota	Acidianus hospitalis W1
Acd1_0707	C1	arCOG01545	C	HyfC	Formate hydrogenlyase subunit 4	229582264	194	Crenarchaeota	Sulfolobus islandicus Y.N.15.51
Acd1_0708	C1	arCOG03626	C	HyfE	Hydrogenase 4 membrane component (E)	15897893	404	Crenarchaeota	Sulfolobus solfataricus P2
Acd1_0709	C1	arCOG01537	C	NuoM	NADH:ubiquinone oxidoreductase subunit 4 (chain M)	385773155	94.7	Crenarchaeota	Sulfolobus islandicus HVE10/4
Acd1_0710	C1	arCOG01547	C	HycE	Ni,Fe-hydrogenase III large subunit and subunit G	227827479	107	Crenarchaeota	Sulfolobus islandicus M.14.25

Acd1_0711	C1	arCOG01553	C	-	Ni,Fe-hydrogenase III small subunit	15897890	272	Crenarchaeota	Sulfolobus solfataricus P2
Acd1_0712	C1	arCOG01537	C	NuoM	NADH:ubiquinone oxidoreductase subunit 4 (chain M)	15897889	1795	Crenarchaeota	Sulfolobus solfataricus P2
Acd1_0713	C1	arCOG08457	S		Uncharacterized conserved protein	15897888	295	Crenarchaeota	Sulfolobus solfataricus P2
Acd1_0714	C1	arCOG00812	K	AbrB	Transcriptional regulator AbrB	229578543	238	Crenarchaeota	Sulfolobus islandicus Y.G.57.14
Acd1_0715	C1	arCOG00713	V	-	PIN domain containing protein	15898764	1102	Crenarchaeota	Sulfolobus solfataricus P2
Acd1_0716	C1	arCOG05276	L		Pyrimidine dimer DNA glycosylase, T4 endoV family	229582386	874	Crenarchaeota	Sulfolobus islandicus Y.N.15.51
Acd1_0717	C1	arCOG07088	S		Uncharacterized conserved protein	15922955	271	Crenarchaeota	Sulfolobus tokodaii str. 7
Acd1_0718	C1	arCOG04217	R	-	Predicted metallopeptidase	15921157	187	Crenarchaeota	Sulfolobus tokodaii str. 7
Acd1_0719	C1	arCOG01939	H	LplA	Lipoate-protein ligase A	332797360	606	Crenarchaeota	Acidianus hospitalis W1
Acd1_0720	C1	arCOG01303	E	GcvH	Glycine cleavage system H protein (lipoate-binding)	332797361	65.9	Crenarchaeota	Acidianus hospitalis W1
Acd1_0721	C1	arCOG05920	S		Uncharacterized conserved protein	332797363	98.6	Crenarchaeota	Acidianus hospitalis W1
Acd1_0722	C1	arCOG03686	O	-	Predicted redox protein, regulator of disulfide bond formation	330834625	220	Crenarchaeota	Metallosphaera cuprina Ar-4
Acd1_0723	C1	arCOG05921	S		Uncharacterized conserved protein	332797365	183	Crenarchaeota	Acidianus hospitalis W1
Acd1_0724	C1	arCOG00338	C	HdrB	Heterodisulfide reductase, subunit B	332797366	252	Crenarchaeota	Acidianus hospitalis W1
Acd1_0725	C1	arCOG00966	C	HdrC	Heterodisulfide reductase, subunit C	15922184	109	Crenarchaeota	Sulfolobus tokodaii str. 7
Acd1_0726	C1	arCOG05916	C		Predicted heterodisulfide reductase subunit	15922185	340	Crenarchaeota	Sulfolobus tokodaii str. 7
Acd1_0727	C1	arCOG02235	O	HdrA	Heterodisulfide reductase, subunit A	332797369	494	Crenarchaeota	Acidianus hospitalis W1
Acd1_0728	C1	arCOG00342	C	HdrB	Heterodisulfide reductase, subunit B	332797370	76.6	Crenarchaeota	Acidianus hospitalis W1
Acd1_0729	C1	arCOG00965	C	HdrC	Heterodisulfide reductase, subunit C	332797371	232	Crenarchaeota	Acidianus hospitalis W1
Acd1_0730	C1		S		Uncharacterized protein				
Acd1_0731	C1		S		Uncharacterized protein	15921429	309	Crenarchaeota	Sulfolobus tokodaii str. 7
Acd1_0732	C1	arCOG07335	Q		SAM-dependent methyltransferase	15921104	221	Crenarchaeota	Sulfolobus tokodaii str. 7
Acd1_0733	C1	arCOG04507	Q	-	Methane/Phenol/Toluene Hydroxylase component	332797382	308	Crenarchaeota	Acidianus hospitalis W1
Acd1_0734	C1	arCOG05895	S	-	Uncharacterized conserved protein	330834537	315	Crenarchaeota	Metallosphaera cuprina Ar-4
Acd1_0735	C1	arCOG07180	S		Uncharacterized conserved protein	15922196	267	Crenarchaeota	Sulfolobus tokodaii str. 7
Acd1_0736	C1	arCOG01218	O	TrxA	Thiol-disulfide isomerase or thioredoxin	146304316	541	Crenarchaeota	Metallosphaera sedula DSM 5348
Acd1_0737	C1	arCOG05917	S		Uncharacterized conserved protein	332797376	359	Crenarchaeota	Acidianus hospitalis W1
Acd1_0738	C1		S		Uncharacterized protein				
Acd1_0739	C1	arCOG01303	E	GcvH	Glycine cleavage system H protein (lipoate-binding)	15922210	31.2	Crenarchaeota	Sulfolobus tokodaii str. 7
Acd1_0740	C1	arCOG05919	R	Gph	HAD superfamily hydrolase	15922209	336	Crenarchaeota	Sulfolobus tokodaii str. 7
Acd1_0741	C1	arCOG00039	R	-	Predicted PP-loop superfamily ATPase	15922208	367	Crenarchaeota	Sulfolobus tokodaii str. 7
Acd1_0742	C1	arCOG05918	S		Uncharacterized conserved protein	385775707	281	Crenarchaeota	Sulfolobus islandicus REY15A
Acd1_0743	C1	arCOG02072	O	SirA	Predicted redox protein, regulator of disulfide bond formation	70605976	98.2	Crenarchaeota	Sulfolobus acidocaldarius DSM 639
Acd1_0744	C1	arCOG01939	H	LplA	Lipoate-protein ligase A	15922202	633	Crenarchaeota	Sulfolobus tokodaii str. 7
Acd1_0745	C1	arCOG05825	R	-	Radical SAM superfamily enzyme	15922201	435	Crenarchaeota	Sulfolobus tokodaii str. 7
Acd1_0746	C1	arCOG00662	R	-	Biotin synthase-related enzyme	15922200	46.2	Crenarchaeota	Sulfolobus tokodaii str. 7
Acd1_0747	C1	arCOG02979	S		Uncharacterized conserved protein	227830085	184	Crenarchaeota	Sulfolobus islandicus L.S.2.15
Acd1_0748	C1	arCOG00326	R	AcuC	Deacetylase, including yeast histone deacetylase and acetoin utilization protein	15922198	144	Crenarchaeota	Sulfolobus tokodaii str. 7
Acd1_0749	C1	arCOG01068	C	Lpd	Pyruvate/2-oxoglutarate dehydrogenase complex, dihydrolipoamide dehydrogenase (E3) component or related enzyme	15922193	139	Crenarchaeota	Sulfolobus tokodaii str. 7
Acd1_0750	C1	arCOG02066	R	-	Predicted peroxiredoxin	15922191	79.3	Crenarchaeota	Sulfolobus tokodaii str. 7
Acd1_0751	C1	arCOG02064	R	-	Peroxiredoxin family protein	15922190	78.2	Crenarchaeota	Sulfolobus tokodaii str. 7
Acd1_0752	C1	arCOG02062	O	SirA	Predicted redox protein, regulator of disulfide bond formation	15922189	100	Crenarchaeota	Sulfolobus tokodaii str. 7
Acd1_0753	C1	arCOG02860	P	CynT	Carbonic anhydrase	18181617	647	Crenarchaeota	Acidianus ambivalens
Acd1_0754	C1	arCOG00279	E	SpeD	S-adenosylmethionine decarboxylase/arginine decarboxylase	15921633	274	Crenarchaeota	Sulfolobus tokodaii str. 7
Acd1_0755	C1	arCOG01767	I	PksG	3-hydroxy-3-methylglutaryl CoA synthase	15921634	35.4	Crenarchaeota	Sulfolobus tokodaii str. 7
Acd1_0756	C1	arCOG01278	I	PaaJ	Acetyl-CoA acetyltransferase	70607119	114	Crenarchaeota	Sulfolobus acidocaldarius DSM 639
Acd1_0757	C1	arCOG01285	R	-	Predicted nucleic-acid-binding protein containing a Zn-ribbon	342306448	184	Crenarchaeota	Sulfolobus tokodaii str. 7
Acd1_0758	C1	arCOG04260	I	HMG1	Hydroxymethylglutaryl-CoA reductase	70607117	163	Crenarchaeota	Sulfolobus acidocaldarius DSM 639
Acd1_0759	C1	arCOG00070	E	GlyA	Glycine/serine hydroxymethyltransferase	15921638	459	Crenarchaeota	Sulfolobus tokodaii str. 7
Acd1_0760	C1	arCOG02053	T	UspA	Nucleotide-binding protein, UspA family	374633630	174	Crenarchaeota	Metallosphaera yellowstonensis MK1
Acd1_0761	C1	arCOG00493	G	GapA	Glyceraldehyde-3-phosphate dehydrogenase/erythrose-4-phosphate dehydrogenase	15921640	249	Crenarchaeota	Sulfolobus tokodaii str. 7
Acd1_0762	C1	arCOG00496	G	Pgk	3-phosphoglycerate kinase	15921641	127	Crenarchaeota	Sulfolobus tokodaii str. 7
Acd1_0763	C1	arCOG05981	S		Uncharacterized conserved protein	146304417	31.6	Crenarchaeota	Metallosphaera sedula DSM 5348
tRNAGlnTTG					tRNAGlnTTG				
tRNAGlnCTG					tRNAGlnCTG				
tRNAThrCGT					tRNAThrCGT				
Acd1_0764	C1	arCOG03846	V	-	Predicted antitoxin, copG family	284175617	932	Crenarchaeota	Sulfolobus solfataricus 98/2
Acd1_0765	C1	arCOG01715	O	sufB	Cysteine desulfurase activator SufB	330834435	261	Crenarchaeota	Metallosphaera cuprina Ar-4
Acd1_0766	C1	arCOG01715	O	sufB	Cysteine desulfurase activator SufB	15921463	468	Crenarchaeota	Sulfolobus tokodaii str. 7
Acd1_0767	C1		S		Uncharacterized protein	332797001	213	Crenarchaeota	Acidianus hospitalis W1
Acd1_0768	C1	arCOG00318	P	PhoU	Phosphate uptake regulator	332797003	580	Crenarchaeota	Acidianus hospitalis W1
Acd1_0769	C1	arCOG01840	O	IbpA	Molecular chaperone (HSP20 family)	15921466	224	Crenarchaeota	Sulfolobus tokodaii str. 7
Acd1_0770	C1	arCOG00347	R	-	Archaeal enzyme of ATP-grasp superfamily	332797005	110	Crenarchaeota	Acidianus hospitalis W1
Acd1_0771	C1	arCOG01303	E	GcvH	Glycine cleavage system H protein (lipoate-binding)	332797006	33.5	Crenarchaeota	Acidianus hospitalis W1
Acd1_0772	C1	arCOG00756	E	GcvT	Glycine cleavage system T protein (aminomethyltransferase)	332797007	132	Crenarchaeota	Acidianus hospitalis W1
Acd1_0773	C1	arCOG00077	E	GcvP	Glycine cleavage system protein P (pyridoxal-binding), N-terminal domain	15921470	506	Crenarchaeota	Sulfolobus tokodaii str. 7
Acd1_0774	C1	arCOG00076	E	GcvP	Glycine cleavage system protein P (pyridoxal-binding), C-terminal domain	332797009	184	Crenarchaeota	Acidianus hospitalis W1
Acd1_0775	C1	arCOG01739	U	LepB	Signal peptidase I	70607136	449	Crenarchaeota	Sulfolobus acidocaldarius DSM 639
Acd1_0776	C1	arCOG04323	S		Zn-finger protein	15921473	350	Crenarchaeota	Sulfolobus tokodaii str. 7
Acd1_0777	C1		S		Uncharacterized protein	14602152	188	Crenarchaeota	Aeropyrum pernix K1
Acd1_0778	C1	arCOG01169	G	Eno	Enolase	332797012	270	Crenarchaeota	Acidianus hospitalis W1
Acd1_0779	C1	arCOG08458	S		Uncharacterized conserved protein	342306397	185	Crenarchaeota	Sulfolobus tokodaii str. 7
Acd1_0780	C1	arCOG04054	R		Potential cell division protein, a component of ECSRT system	15897798	1177	Crenarchaeota	Sulfolobus solfataricus P2
Acd1_0781	C1	arCOG00453	D	ESCRTIII	Crenarchaeal division protein ESCRT-III	146304434	164	Crenarchaeota	Metallosphaera sedula DSM 5348
Acd1_0782	C1	arCOG01307	D	Vps4	Cell division ATPase of the AAA+ class, ESCRT system component	15897796	102	Crenarchaeota	Sulfolobus solfataricus P2
Acd1_0783	C1	arCOG01527	L	TopA	Topoisomerase IA	15921479	203	Crenarchaeota	Sulfolobus tokodaii str. 7
Acd1_0784	C1	arCOG01268	K	RpoZ	DNA-directed RNA polymerase, subunit K/omega	15921480	1287	Crenarchaeota	Sulfolobus tokodaii str. 7
Acd1_0785	C1	arCOG00082	E	-	Serine-pyruvate aminotransferase/archaeal aspartate aminotransferase	15921481	208	Crenarchaeota	Sulfolobus tokodaii str. 7
Acd1_0786	C1	arCOG01754	H	SerA	Phosphoglycerate dehydrogenase or related dehydrogenase	70607125	85.1	Crenarchaeota	Sulfolobus acidocaldarius DSM 639
Acd1_0787	C1	arCOG04357	L	-	Thermostable 8-oxoguanine DNA glycosylase	70607124	138	Crenarchaeota	Sulfolobus acidocaldarius DSM 639
Acd1_0788	C1	arCOG07219	S		Uncharacterized conserved protein				
Acd1_0789	C1	arCOG04103	S		Uncharacterized conserved protein	342306399	186	Crenarchaeota	Sulfolobus tokodaii str. 7
Acd1_0790	C1	arCOG08333	S		Uncharacterized conserved protein	229579105	192	Crenarchaeota	Sulfolobus islandicus Y.G.57.14
Acd1_0791	C1	arCOG04062	S		Uncharacterized conserved protein	70607184	263	Crenarchaeota	Sulfolobus acidocaldarius DSM 639
Acd1_0792	C1	arCOG00570	C	FixC	Dehydrogenase (flavoprotein)	15921487	58.9	Crenarchaeota	Sulfolobus tokodaii str. 7
Acd1_0793	C1	arCOG00808	J	ValS	Valyl-tRNA synthetase	161751094	368	Crenarchaeota	Sulfolobus tokodaii str. 7
Acd1_0794	C1	arCOG01130	E	-	Aspartate/tyrosine/aromatic aminotransferase	15921489	45.1	Crenarchaeota	Sulfolobus tokodaii str. 7
Acd1_0795	C1	arCOG01088	E	TrpC	Indole-3-glycerol phosphate synthase	374633692	121	Crenarchaeota	Metallosphaera yellowstonensis MK1
Acd1_0796	C1	arCOG00086	E	PabA	Anthranilate/para-aminobenzoate synthase component II	70607179	491	Crenarchaeota	Sulfolobus acidocaldarius DSM 639
Acd1_0797	C1	arCOG02014	E	TrpE	Anthranilate/para-aminobenzoate synthase component I	70607178	157	Crenarchaeota	Sulfolobus acidocaldarius DSM 639
Acd1_0798	C1	arCOG01983	E	TrpF	Phosphoribosylanthranilate isomerase	342306402	623	Crenarchaeota	Sulfolobus tokodaii str. 7
Acd1_0799	C1	arCOG02012	E	TrpD	Anthranilate phosphoribosyltransferase	15897778	940	Crenarchaeota	Sulfolobus solfataricus P2
Acd1_0800	C1	arCOG01086	E	TrpA	Tryptophan synthase alpha chain	15921495	306	Crenarchaeota	Sulfolobus tokodaii str. 7
Acd1_0801	C1	arCOG01432	R	-	Tryptophan synthase beta-subunit	15921496	65.1	Crenarchaeota	Sulfolobus tokodaii str. 7
Acd1_0802	C1	arCOG01674	C	AcyP	Acylphosphatase	15897775	274	Crenarchaeota	Sulfolobus solfataricus P2
Acd1_0803	C1	arCOG04213	I	INO1	Myo-inositol-1-phosphate synthase	15921498	592	Crenarchaeota	Sulfolobus tokodaii str. 7
Acd1_0804	C1	arCOG01111	G	PpsA	Phosphoenolpyruvate synthase/pyruvate phosphate dikinase	342306404	179	Crenarchaeota	Sulfolobus tokodaii str. 7
Acd1_0805	C1	arCOG00452	D	ESCRTIII	Crenarchaeal division protein ESCRT-III	15921500	173	Crenarchaeota	Sulfolobus tokodaii str. 7
Acd1_0806	C1	arCOG07220	S		Uncharacterized conserved protein	332797041	174	Crenarchaeota	Acidianus hospitalis W1
Acd1_0807	C1	arCOG01434	E	ThrC	Threonine synthase and cysteate synthase	70607166	184	Crenarchaeota	Sulfolobus acidocaldarius DSM 639
Acd1_0808	C1	arCOG00861	E	LysC	Aspartokinase	70607165	490	Crenarchaeota	Sulfolobus acidocaldarius DSM 639
Acd1_0809	C1	arCOG00494	E	Asd	Aspartate-semialdehyde dehydrogenase	15921505	207	Crenarchaeota	Sulfolobus tokodaii str. 7
Acd1_0810	C1	arCOG00302	R	-	Metal-dependent phosphoesterase (PHP family)	330834389	32	Crenarchaeota	Metallosphaera cuprina Ar-4
Acd1_0811	C1	arCOG04269	S	-	Protein, predicted to be involved in DNA repair	15921507	63.2	Crenarchaeota	Sulfolobus tokodaii str. 7
Acd1_0812	C1	arCOG00912	E	ArgF	Ornithine carbamoyltransferase	332797048	244	Crenarchaeota	Acidianus hospitalis W1
Acd1_0813	C1	arCOG02103	K		Transcriptional regulator, contains wHTH domain	15921509	193	Crenarchaeota	Sulfolobus tokodaii str. 7
Acd1_0814	C1	arCOG01350	R	-	Predicted inorganic polyphosphate/ATP-NAD kinase	227827619	923	Crenarchaeota	Sulfolobus islandicus M.14.25
Acd1_0815	C1	arCOG04268	L	Cdt1	Replication initiator protein WhiP, Cdt1-like protein, contains HTH domain	146304469	71.6	Crenarchaeota	Metallosphaera sedula DSM 5348
Acd1_0816	C1	arCOG04266	S		Uncharacterized conserved protein	15921512	520	Crenarchaeota	Sulfolobus tokodaii str. 7
Acd1_0817	C1	arCOG04160	S		Uncharacterized conserved protein	15921513	140	Crenarchaeota	Sulfolobus tokodaii str. 7
Acd1_0818	C1	arCOG05984	S		Uncharacterized conserved protein	15921514	155	Crenarchaeota	Sulfolobus tokodaii str. 7
Acd1_0819	C1	arCOG01257	O	GroL	Chaperonin GroEL (HSP60 family)	15921515	322	Crenarchaeota	Sulfolobus tokodaii str. 7
Acd1_0820	C1	arCOG00569	S		Uncharacterized conserved protein	332797056	120	Crenarchaeota	Acidianus hospitalis W1
Acd1_0821	C1	arCOG03879	K	-	Predicted transcriptional regulator	70607152	796	Crenarchaeota	Sulfolobus acidocaldarius DSM 639
Acd1_0822	C1	arCOG05983	S		Uncharacterized conserved protein	332797059	97.8	Crenarchaeota	Acidianus hospitalis W1
Acd1_0823	C1	arCOG00487	J	ArgS	Arginyl-tRNA synthetase	70607149	160	Crenarchaeota	Sulfolobus acidocaldarius DSM 639
tRNAArgTCG					tRNAArgTCG				
Acd1_0824	C1	arCOG03713	F	NrdA	Ribonucleotide reductase associated ATP-cone domain	332797062	162	Crenarchaeota	Acidianus hospitalis W1
Acd1_0825	C1	arCOG00017	R	-	Predicted transcriptional regulator	332797063	272	Crenarchaeota	Acidianus hospitalis W1
Acd1_0826	C1	arCOG05980	S		Uncharacterized conserved protein	146304481	684	Crenarchaeota	Metallosphaera sedula DSM 5348
Acd1_0827	C1	arCOG01719	J	GatE	Archaeal Glu-tRNAGln amidotransferase subunit E (contains GAD domain)	15921525	50.1	Crenarchaeota	Sulfolobus tokodaii str. 7
Acd1_0828	C1	arCOG01924	E	AnsB	L-asparaginase/archaeal Glu-tRNAGln amidotransferase subunit D	15921527	91.3	Crenarchaeota	Sulfolobus tokodaii str. 7
Acd1_0830	C1	arCOG04293	J	-	Ribosomal protein S30	15921528	249	Crenarchaeota	Sulfolobus tokodaii str. 7
Acd1_0829	C1	arCOG01923	J	SIK1	Protein implicated in ribosomal biogenesis, Nop56p homolog	15921529	322	Crenarchaeota	Sulfolobus tokodaii str. 7
Acd1_0831	C1	arCOG00078	J	NOP1	Fibrillarin-like rRNA methylase	332797069	274	Crenarchaeota	Acidianus hospitalis W1
Acd1_0832	C1	arCOG01921	S	-	Uncharacterized conserved protein	15921531	190	Crenarchaeota	Sulfolobus tokodaii str. 7
Acd1_0833	C1	arCOG04152	K	-	Predicted transcriptional regulator	330834366	201	Crenarchaeota	Metallosphaera cuprina Ar-4
Acd1_0834	C1	arCOG01219	J	TRM1	N2,N2-dimethylguanosine tRNA methyltransferase	15921533	57	Crenarchaeota	Sulfolobus tokodaii str. 7
Acd1_0835	C1	arCOG04153	K		RNA polymerase III subunit C34 (Lrp/AsnC family)	15921534	94.7	Crenarchaeota	Sulfolobus tokodaii str. 7
Acd1_0836	C1	arCOG01981	K	SUA7	Transcription initiation factor TFIIIB, Brf1 subunit/Transcription initiation factor TFIIB	227827554	536	Crenarchaeota	Sulfolobus islandicus M.14.25
Acd1_0837	C1	arCOG02466	J	GAR1	RNA-binding protein involved in rRNA processing	70607098	98.2	Crenarchaeota	Sulfolobus acidocaldarius DSM 639
Acd1_0838	C1	arCOG01159	S	-	Uncharacterized archaeal coiled-coil protein	15897827	305	Crenarchaeota	Sulfolobus solfataricus P2
Acd1_0839	C1	arCOG04151	R	-	ABC-type multidrug transport system, permease component	15921538	42.7	Crenarchaeota	Sulfolobus tokodaii str. 7
Acd1_0840	C1	arCOG04104	J		Ribosome associated protein L46a	227827550	300	Crenarchaeota	Sulfolobus islandicus M.14.25
Acd1_0841	C1	arCOG01764	K	SPT15	TATA-box binding protein (TBP), component of TFIID and TFIIIB	14423939	388	Crenarchaeota	Sulfolobus solfataricus P2

Acd1_0842	C1	arCOG01150	R	-	Predicted ICC-like phosphoesterase	15921540	332	Crenarchaeota	Sulfolobus tokodaii str. 7
Acd1_0843	C1	arCOG04161	J	DPH5	Diphthamide biosynthesis methyltransferase	332797081	154	Crenarchaeota	Acidianus hospitalis W1
Acd1_0844	C1	arCOG01224	M	TadA	Cytidylyltransferase fused to conserved domain of DUF357 family	15921542	269	Crenarchaeota	Sulfolobus tokodaii str. 7
Acd1_0845	C1	arCOG01904	H	-	CTP-dependent Riboflavin kinase	330834354	85.9	Crenarchaeota	Metallosphaera cuprina Ar-4
Acd1_0846	C1	arCOG04174	J	TYW3	Wybutosine (yW) biosynthesis enzyme, Fe-S oxidoreductase	15897835	519	Crenarchaeota	Sulfolobus solfataricus P2
Acd1_0847	C1	arCOG01717	J	GatA	Asp-tRNAAsn/Glu-tRNAGln amidotransferase A subunit or related amidase	15921546	196	Crenarchaeota	Sulfolobus tokodaii str. 7
Acd1_0848	C1	arCOG02726	J	GatC	Asp-tRNAAsn/Glu-tRNAGln amidotransferase C subunit	15921548	137	Crenarchaeota	Sulfolobus tokodaii str. 7
Acd1_0849	C1	arCOG04231	P	CutA	Uncharacterized protein involved in tolerance to divalent cations	163784194	30.8	Aquificae	Hydrogenivirga sp. 128-5-R1-1
Acd1_0850	C1	arCOG07217	S		Uncharacterized conserved protein	227827540	38.1	Crenarchaeota	Sulfolobus islandicus M.14.25
Acd1_0851	C1	arCOG00874	K	xpb	ssDNA-dependent ATPase, helicase superfamily II	15921551	294	Crenarchaeota	Sulfolobus tokodaii str. 7
Acd1_0852	C1	arCOG05979	S		Uncharacterized conserved protein	70607083	189	Crenarchaeota	Sulfolobus acidocaldarius DSM 639
Acd1_0853	C1	arCOG07216	S		Uncharacterized conserved protein	374633752	115	Crenarchaeota	Metallosphaera yellowstonensis MK1
Acd1_0854	C1	arCOG04324	S		Uncharacterized conserved protein	70607081	288	Crenarchaeota	Sulfolobus acidocaldarius DSM 639
Acd1_0855	C1	arCOG01753	K	Ssh10b	Archaeal DNA-binding protein	15921554	270	Crenarchaeota	Sulfolobus tokodaii str. 7
Acd1_0856	C1	arCOG01526	L	-	Reverse gyrase	332797094	52	Crenarchaeota	Acidianus hospitalis W1
Acd1_0857	C1	arCOG01753	K	Ssh10b	Archaeal DNA-binding protein	227827533	236	Crenarchaeota	Sulfolobus islandicus M.14.25
Acd1_0858	C1	arCOG00557	R	Lhr	Lhr-like helicase	15921557	169	Crenarchaeota	Sulfolobus tokodaii str. 7
Acd1_0859	C1	arCOG04135	R		Zn-finger protein	332797097	439	Crenarchaeota	Acidianus hospitalis W1
Acd1_0860	C1	arCOG04246	J	RtcB	RNA 3'-P ligase, RtcB family protein	15921558	424	Crenarchaeota	Sulfolobus tokodaii str. 7
Acd1_0861	C1	arCOG04142	O	DYS1	Deoxyhypusine synthase	70607075	444	Crenarchaeota	Sulfolobus acidocaldarius DSM 639
Acd1_0862	C1	arCOG01165	L	-	DNA topoisomerase VI, subunit B	70607074	366	Crenarchaeota	Sulfolobus acidocaldarius DSM 639
Acd1_0863	C1	arCOG04143	L	-	DNA topoisomerase VI, subunit A	70607073	163	Crenarchaeota	Sulfolobus acidocaldarius DSM 639
Acd1_0864	C1	arCOG08093	S		Zn zinger protein	332797102	649	Crenarchaeota	Acidianus hospitalis W1
Acd1_0865	C1	arCOG04277	J	Efp	Translation elongation factor P (EF-P)/translation initiation factor 5A (eIF-5A)	332797103	198	Crenarchaeota	Acidianus hospitalis W1
Acd1_0866	C1	arCOG01228	U	Ffh	Signal recognition particle GTPase	70607071	893	Crenarchaeota	Sulfolobus acidocaldarius DSM 639
Acd1_0867	C1	arCOG01015	J	-	Predicted pseudouridylate synthase	342306425	387	Crenarchaeota	Sulfolobus tokodaii str. 7
Acd1_0868	C1	arCOG01260	I	FabG	Short-chain alcohol dehydrogenase	227827522	426	Crenarchaeota	Sulfolobus islandicus M.14.25
Acd1_0869	C1	arCOG04114	L		Chromatin protein Cren7	15897854	373	Crenarchaeota	Sulfolobus solfataricus P2
Acd1_0870	C1	arCOG00858	F	PyrH	Uridylate kinase	15897855	163	Crenarchaeota	Sulfolobus solfataricus P2
Acd1_0871	C1	arCOG07215	S		Uncharacterized conserved protein	332797111	154	Crenarchaeota	Acidianus hospitalis W1
Acd1_0872	C1	arCOG02092	E	LeuA	Isopropylmalate/homocitrate/citramalate synthase	146304528	378	Crenarchaeota	Metallosphaera sedula DSM 5348
tRNALeuTAA					tRNALeuTAA				
Acd1_0873	C1	arCOG01122	G	RpiA	Ribose 5-phosphate isomerase	15897858	182	Crenarchaeota	Sulfolobus solfataricus P2
16s SSU rRNA					16s SSU rRNA				
23s LSU rRNA					23s LSU rRNA				
Acd1_0874	C1	arCOG00312	O	AhpC	Peroxiredoxin	302348384	334	Crenarchaeota	Acidilobus saccharovorans 345-15
Acd1_0875	C1	arCOG01943	Q	PncA	Amidase related to nicotinamidase	307596537	681	Crenarchaeota	Vulcanisaeta distributa DSM 14429
Acd1_0876	C1	arCOG01521	Q	-	Phytoene dehydrogenase or related enzyme	70607009	197	Crenarchaeota	Sulfolobus acidocaldarius DSM 639
Acd1_0877	C1	arCOG00777	Q	PaaI	HGG motif-containing thioesterase, possibly involved in aromatic compounds catabolism	332795875	226	Crenarchaeota	Acidianus hospitalis W1
Acd1_0878	C1	arCOG02937	I	MVD1	Mevalonate pyrophosphate decarboxylase	342306345	166	Crenarchaeota	Sulfolobus tokodaii str. 7
Acd1_0879	C1	arCOG01032	I	ERG8	Phosphomevalonate kinase	15921220	34.7	Crenarchaeota	Sulfolobus tokodaii str. 7
Acd1_0880	C1	arCOG01619	R	ARA1	Aldo/keto reductase, related to diketogulonate reductase	146303237	55.1	Crenarchaeota	Metallosphaera sedula DSM 5348
Acd1_0881	C1	arCOG05974	S		Uncharacterized conserved protein	229585838	440	Crenarchaeota	Sulfolobus islandicus M.16.27
Acd1_0882	C1	arCOG01055	K	-	Predicted transcriptional regulator	15921222	288	Crenarchaeota	Sulfolobus tokodaii str. 7
Acd1_0883	C1		S		Uncharacterized protein				
Acd1_0884	C1	arCOG05895	S	-	Uncharacterized conserved protein	15922197	58.5	Crenarchaeota	Sulfolobus tokodaii str. 7
Acd1_0885	C1	arCOG04227	R	-	Predicted CoA-binding protein	15920295	618	Crenarchaeota	Sulfolobus tokodaii str. 7
Acd1_0886	C1	arCOG02242	K	-	Transcriptional regulator, contains HTH domain	15921223	140	Crenarchaeota	Sulfolobus tokodaii str. 7
Acd1_0887	C1	arCOG00968	R	-	Alpha/beta-knot SAM-dependent RNA methyltransferase	156937971	223	Crenarchaeota	Ignicoccus hospitalis KIN4/I
Acd1_0888	C1	arCOG04430	R	-	HD superfamily phosphohydrolase	15921218	569	Crenarchaeota	Sulfolobus tokodaii str. 7
Acd1_0889	C1	arCOG01324	F	Udp	Uridine phosphorylase	15921217	406	Crenarchaeota	Sulfolobus tokodaii str. 7
Acd1_0890	C1	arCOG08212	S		Uncharacterized conserved protein	332797167	127	Crenarchaeota	Acidianus hospitalis W1
Acd1_0891	C1	arCOG01065	R	HcaD	NAD(FAD)-dependent dehydrogenase	330834931	55.8	Crenarchaeota	Metallosphaera cuprina Ar-4
Acd1_0892	C1	arCOG00940	R	-	Radical SAM superfamily enzyme	146303294	506	Crenarchaeota	Metallosphaera sedula DSM 5348
Acd1_0893	C1	arCOG00504	R	GloB	Metal-dependent hydrolase of the beta-lactamase superfamily II	374632342	209	Crenarchaeota	Metallosphaera yellowstonensis MK1
Acd1_0894	C1	arCOG03202	O	-	Collagenase family protease	332797312	43.9	Crenarchaeota	Acidianus hospitalis W1
tRNAThrGGT					tRNAThrGGT				
Acd1_0895	C1	arCOG02062	O	SirA	Predicted redox protein, regulator of disulfide bond formation	15899355	72	Crenarchaeota	Sulfolobus solfataricus P2
Acd1_0896	C1	arCOG01064	R	HcaD	NAD(FAD)-dependent dehydrogenase	227831644	452	Crenarchaeota	Sulfolobus islandicus L.S.2.15
Acd1_0897	C1	arCOG02114	S	-	Uncharacterized conserved protein	284999195	651	Crenarchaeota	Sulfolobus islandicus L.D.8.5
tRNAGlyCCC					tRNAGlyCCC				
Acd1_0898	C1	arCOG03674	E		Thermopsin-like protease	332795700	121	Crenarchaeota	Acidianus hospitalis W1
Acd1_0899	C1	arCOG04761	I	UppP	Undecaprenyl pyrophosphate phosphatase	374631172	559	Crenarchaeota	Metallosphaera yellowstonensis MK1
Acd1_0900	C1		S		Uncharacterized protein	255513851	320	Euryarchaeota	Candidatus Micrarchaeum acidiphilum ARMAN-2
Acd1_0901	C1	arCOG07213	S	0	Uncharacterized conserved protein	70607059	483	Crenarchaeota	Sulfolobus acidocaldarius DSM 639
Acd1_0902	C1	arCOG01263	R	DltE	Short-chain dehydrogenase	332796939	66.2	Crenarchaeota	Acidianus hospitalis W1
Acd1_0903	C1	arCOG00164	O	CysU	ABC-type sulfate transport system, permease component	374633265	118	Crenarchaeota	Metallosphaera yellowstonensis MK1
Acd1_0904	C1	arCOG07536	S	-	Secreted protein, containing kelch and fibronectin type 3 domains	15921172	148	Crenarchaeota	Sulfolobus tokodaii str. 7
tRNAProGGG					tRNAProGGG				
Acd1_0905	C1	arCOG01182	T	-	Predicted Ser/Thr protein kinase	15921189	85.1	Crenarchaeota	Sulfolobus tokodaii str. 7
Acd1_0906	C1	arCOG04249	J	CCA1	tRNA nucleotidyltransferase (CCA-adding enzyme)	15921188	318	Crenarchaeota	Sulfolobus tokodaii str. 7
Acd1_0907	C1	arCOG01736	J	LigT	2'-5' RNA ligase	342306332	385	Crenarchaeota	Sulfolobus tokodaii str. 7
Acd1_0908	C1	arCOG01045	H	CoaE	Dephospho-CoA kinase	15921186	486	Crenarchaeota	Sulfolobus tokodaii str. 7
Acd1_0909	C1	arCOG01043	R	-	Predicted RNA binding protein with dsRBD fold	15921185	1234	Crenarchaeota	Sulfolobus tokodaii str. 7
Acd1_0910	C1	arCOG00501	R	ElaC	Metal-dependent hydrolase of the beta-lactamase superfamily	15921184	452	Crenarchaeota	Sulfolobus tokodaii str. 7
Acd1_0911	C1	arCOG00047	L	-	Predicted DNA modification methylase	374633803	35	Crenarchaeota	Metallosphaera yellowstonensis MK1
Acd1_0912	C1	arCOG00067	F	PrsA	Phosphoribosylpyrophosphate synthetase	70607047	167	Crenarchaeota	Sulfolobus acidocaldarius DSM 639
Acd1_0913	C1	arCOG01225	R	-	GTPase SAR1 or related small G protein	332796927	400	Crenarchaeota	Acidianus hospitalis W1
Acd1_0914	C1	arCOG00488	L	DnaN	DNA polymerase sliding clamp subunit (PCNA homolog)	15921180	534	Crenarchaeota	Sulfolobus tokodaii str. 7
Acd1_0915	C1	arCOG04110	L	PRI1	Eukaryotic-type DNA primase, catalytic (small) subunit	15921179	94	Crenarchaeota	Sulfolobus tokodaii str. 7
Acd1_0916	C1	arCOG00551	L		DNA replication initiation complex subunit, GINS15 family	15921178	258	Crenarchaeota	Sulfolobus tokodaii str. 7
Acd1_0917	C1	arCOG04109	J	RPL42A	Ribosomal protein L44E	15921177	201	Crenarchaeota	Sulfolobus tokodaii str. 7
Acd1_0918	C1	arCOG04108	J	RPS27A	Ribosomal protein S27E	15921176	643	Crenarchaeota	Sulfolobus tokodaii str. 7
Acd1_0919	C1	arCOG04107	J	SUI2	Translation initiation factor 2, alpha subunit (eIF-2alpha)	332796933	384	Crenarchaeota	Acidianus hospitalis W1
Acd1_0920	C1	arCOG00906	J	-	Predicted Zn-ribbon RNA-binding protein	15921174	263	Crenarchaeota	Sulfolobus tokodaii str. 7
Acd1_0921	C1	arCOG02044	R	-	Uncharacterized membrane protein, required for N-linked glycosylation	15921173	207	Crenarchaeota	Sulfolobus tokodaii str. 7
tRNAIleGAT					tRNAIleGAT				
tRNAAlaTGC					tRNAAlaTGC				
Acd1_0922	C1	arCOG10437	S		Uncharacterized conserved protein	284175516	221	Crenarchaeota	Sulfolobus solfataricus 98/2
Acd1_0923	C1	arCOG00663	M	GCD1	Nucleoside-diphosphate-sugar pyrophosphorylase involved in lipopolysaccharide biosynthesis/translation initiation factor 2B, gamma/epsilon subunit	15921209	534	Crenarchaeota	Sulfolobus tokodaii str. 7
tRNAProCGG					tRNAProCGG				
Acd1_0924	C1	arCOG07210	S		Uncharacterized conserved protein	70607034	134	Crenarchaeota	Sulfolobus acidocaldarius DSM 639
Acd1_0925	C1	arCOG01339	C	SucD	Succinyl-CoA synthetase, alpha subunit	374633561	71.2	Crenarchaeota	Metallosphaera yellowstonensis MK1
Acd1_0926	C1	arCOG01337	C	SucC	Succinyl-CoA synthetase, beta subunit	332797165	187	Crenarchaeota	Acidianus hospitalis W1
Acd1_0927	C1	arCOG00461	L	-	Uncharacterized protein related to Endonuclease III	15921205	263	Crenarchaeota	Sulfolobus tokodaii str. 7
Acd1_0928	C1	arCOG01394	M	MurG	UDP-N-acetylglucosamine:LPS N-acetylglucosamine transferase	15921206	64.3	Crenarchaeota	Sulfolobus tokodaii str. 7
Acd1_0929	C1	arCOG00401	J	ThrS	Threonyl-tRNA synthetase	15921207	102	Crenarchaeota	Sulfolobus tokodaii str. 7
Acd1_0930	C1	arCOG02724	L	Ada	Methylated DNA-protein cysteine methyltransferase	70607028	102	Crenarchaeota	Sulfolobus acidocaldarius DSM 639
tRNAAlaCGC					tRNAAlaCGC				
Acd1_0931	C1	arCOG01755	C	LdhA	Lactate dehydrogenase or related 2-hydroxyacid dehydrogenase	374632850	643	Crenarchaeota	Metallosphaera yellowstonensis MK1
Acd1_0932	C1		S		Uncharacterized protein				
Acd1_0933	C1	arCOG06017	S		Uncharacterized conserved protein	146303289	241	Crenarchaeota	Metallosphaera sedula DSM 5348
Acd1_0934	C1		S		Uncharacterized protein				
Acd1_0935	C1		S		Uncharacterized protein				
Acd1_0936	C1		R		Predicted ATPase	14601042	314	Crenarchaeota	Aeropyrum pernix K1
Acd1_0937	C1		S		Uncharacterized protein	118431239	204	Crenarchaeota	Aeropyrum pernix K1
Acd1_0938	C1		S		Uncharacterized protein	14601040	508	Crenarchaeota	Aeropyrum pernix K1
Acd1_0939	C1	arCOG00462	L	MutY	A/G-specific DNA glycosylase	118431238	427	Crenarchaeota	Aeropyrum pernix K1
Acd1_0940	C1	arCOG06089	S		Uncharacterized conserved protein	374326871	125	Crenarchaeota	Pyrobaculum sp. 1860
Acd1_0941	C1	arCOG06089	S		Uncharacterized conserved protein	118431237	388	Crenarchaeota	Aeropyrum pernix K1
Acd1_0942	C1	arCOG04157	L	Dcm	Site-specific DNA methylase	118431236	697	Crenarchaeota	Aeropyrum pernix K1
Acd1_0943	C1	arCOG01064	R	HcaD	NAD(FAD)-dependent dehydrogenase	15922539	137	Crenarchaeota	Sulfolobus tokodaii str. 7
Acd1_0944	C2	arCOG00940	R	-	Radical SAM superfamily enzyme	332797245	288	Crenarchaeota	Acidianus hospitalis W1
Acd1_0945	C2	arCOG01671	H	UbiD	3-polyprenyl-4-hydroxybenzoate decarboxylase or related decarboxylase	307595548	248	Crenarchaeota	Vulcanisaeta distributa DSM 14429
Acd1_0946	C2	arCOG03682	R	SPS1	Membrane associated serine/threonine protein kinase	146303114	216	Crenarchaeota	Metallosphaera sedula DSM 5348
Acd1_0947	C2	arCOG01397	M	WcaA	Glycosyltransferase	284997381	41.2	Crenarchaeota	Sulfolobus islandicus L.D.8.5
Acd1_0948	C2	arCOG01400	Q		SAM-dependent methyltransferase	284997365	43.5	Crenarchaeota	Sulfolobus islandicus L.D.8.5
Acd1_0949	C2		S		Uncharacterized protein	284997365	256	Crenarchaeota	Sulfolobus islandicus L.D.8.5
Acd1_0950	C2	arCOG01389	M	-	Glycosyltransferase	284997361	273	Crenarchaeota	Sulfolobus islandicus L.D.8.5
Acd1_0951	C2		S		Uncharacterized protein	284997361	121	Crenarchaeota	Sulfolobus islandicus L.D.8.5
Acd1_0952	C2	arCOG01371	M	RfbB	dTDP-D-glucose 4,6-dehydratase	284997400	408	Crenarchaeota	Sulfolobus islandicus L.D.8.5
Acd1_0953	C2	arCOG00667	M	RfbA	dTDP-glucose pyrophosphorylase	385775562	682	Crenarchaeota	Sulfolobus islandicus REY15A
Acd1_0954	C2	arCOG01367	M	RfbD	dTDP-4-dehydrorhamnose reductase	229578479	442	Crenarchaeota	Sulfolobus islandicus Y.G.57.14
Acd1_0955	C2	arCOG04188	M	RfbC	dTDP-4-dehydrorhamnose 3,5-epimerase or related enzyme	227829530	94	Crenarchaeota	Sulfolobus islandicus L.S.2.15
Acd1_0956	C2	arCOG01697	C	AcnA	Aconitase A	15897959	295	Crenarchaeota	Sulfolobus solfataricus P2
Acd1_0957	C2	arCOG01212	S		Uncharacterized conserved protein	15897960	114	Crenarchaeota	Sulfolobus solfataricus P2
Acd1_0958	C2	arCOG03772	S	-	Uncharacterized conserved protein	374633826	264	Crenarchaeota	Metallosphaera yellowstonensis MK1
Acd1_0959	C2	arCOG01331	O	HtpX	Zn-dependent protease with chaperone function	146303788	321	Crenarchaeota	Metallosphaera sedula DSM 5348
Acd1_0960	C2	arCOG04811	S		Uncharacterized conserved membrane protein, Fun14 family	284173937	153	Crenarchaeota	Sulfolobus solfataricus 98/2
Acd1_0961	C2	arCOG07849	J		Ribosome associated protein L45a	330834700	246	Crenarchaeota	Metallosphaera cuprina Ar-4
Acd1_0962	C2	arCOG02242	K	-	Transcriptional regulator, contains HTH domain	374633363	127	Crenarchaeota	Metallosphaera yellowstonensis MK1
Acd1_0963	C2	arCOG05482	T	RAD55	RecA-superfamily ATPase implicated in signal transduction	15921072	390	Crenarchaeota	Sulfolobus tokodaii str. 7
Acd1_0964	C2		S		Uncharacterized protein	374631132	617	Crenarchaeota	Metallosphaera yellowstonensis MK1
Acd1_0965	C2	arCOG03721	V		HEPN domain	70606066	486	Crenarchaeota	Sulfolobus acidocaldarius DSM 639
Acd1_0966	C2	arCOG03682	R	SPS1	Membrane associated serine/threonine protein kinase	146303644	394	Crenarchaeota	Metallosphaera sedula DSM 5348

Acd1_0967	C2	arCOG02021	P	PspE	Rhodanese-related sulfurtransferase	15920833	96.7	Crenarchaeota	Sulfolobus tokodaii str. 7
Acd1_0968	C2	arCOG03672	E		Thermopsin-like protease	385772646	97.8	Crenarchaeota	Sulfolobus islandicus HVE10/4
Acd1_0969	C2	arCOG00216	H	MoeA	Molybdopterin biosynthesis enzyme	325968768	78.6	Crenarchaeota	Vulcanisaeta moutnovskia 768-28
Acd1_0970	C2	arCOG07323	S		Uncharacterized conserved protein	374632223	277	Crenarchaeota	Metallosphaera yellowstonensis MK1
Acd1_0971	C2	arCOG01578	P	MgtA	Cation transport ATPase	346430357	296	environmental samples	uncultured archaeon
Acd1_0972	C2	arCOG01400	Q		SAM-dependent methyltransferase	385772551	336	Crenarchaeota	Sulfolobus islandicus HVE10/4
Acd1_0973	C2	arCOG01252	C	PutA	Lactaldehyde dehydrogenase, Succinate semialdehyde dehydrogenase or other NAD-dependent aldehyde dehydrogenase	15922809	147	Crenarchaeota	Sulfolobus tokodaii str. 7
Acd1_0974	C2	arCOG00651	H	CbiG	Cobalamin biosynthesis protein CbiG	374632905	46.6	Crenarchaeota	Metallosphaera yellowstonensis MK1
Acd1_0975	C2	arCOG00650	H	CobL	Precorrin-6B methylase 1	374632904	776	Crenarchaeota	Metallosphaera yellowstonensis MK1
Acd1_0976	C2	arCOG02246	P	-	Sirohydrochlorin ferrochelatase	374632903	270	Crenarchaeota	Metallosphaera yellowstonensis MK1
Acd1_0977	C2	arCOG00647	H	CobJ	Precorrin-3B methylase	374632902	599	Crenarchaeota	Metallosphaera yellowstonensis MK1
Acd1_0978	C2	arCOG04383	H	CbiD	Cobalamin biosynthesis protein CbiD	374632901	181	Crenarchaeota	Metallosphaera yellowstonensis MK1
Acd1_0979	C2	arCOG00977	H	CobL	Precorrin-6B methylase 2	374632900	85.5	Crenarchaeota	Metallosphaera yellowstonensis MK1
Acd1_0980	C2	arCOG00648	H	CobF	Precorrin-2 methylase	374632899	417	Crenarchaeota	Metallosphaera yellowstonensis MK1
Acd1_0981	C2	arCOG00645	H	CobM	Precorrin-4 methylase	374632898	117	Crenarchaeota	Metallosphaera yellowstonensis MK1
Acd1_0982	C2	arCOG00009	E	PotE	Amino acid transporter	374633400	258	Crenarchaeota	Metallosphaera yellowstonensis MK1
tRNAHisGTG					tRNAHisGTG				
Acd1_0983	C2	arCOG01704	H	Dfp	Phosphopantothenoylcysteine synthetase/decarboxylase	15920445	130	Crenarchaeota	Sulfolobus tokodaii str. 7
Acd1_0984	C2	arCOG00833	R	RimI	Acetyltransferase (GNAT) family	15920446	42.4	Crenarchaeota	Sulfolobus tokodaii str. 7
Acd1_0985	C2	arCOG01145	R	-	Icc family phosphoesterase	15920447	353	Crenarchaeota	Sulfolobus tokodaii str. 7
Acd1_0986	C2	arCOG04238	H	CitG	Triphosphoribosyl-dephospho-CoA synthetase	332796330	122	Crenarchaeota	Acidianus hospitalis W1
Acd1_0987	C2	arCOG01831	R	-	Predicted nucleotidyltransferase	70606295	51.6	Crenarchaeota	Sulfolobus acidocaldarius DSM 639
Acd1_0988	C2	arCOG04329	C	-	Predicted butyrate kinase	332796328	140	Crenarchaeota	Acidianus hospitalis W1
Acd1_0989	C2	arCOG03765	K	K	CopG family transcriptional regulator	330835793	1211	Crenarchaeota	Metallosphaera cuprina Ar-4
Acd1_0990	C2	arCOG03765	S		Uncharacterized conserved membrane protein	124027863	267	Crenarchaeota	Hyperthermus butylicus DSM 5456
tRNAMetCAT					tRNAMetCAT				
tRNALysCTT					tRNALysCTT				
Acd1_0991	C2	arCOG00767	G	{ManB}	Phosphomannomutase	15920425	168	Crenarchaeota	Sulfolobus tokodaii str. 7
Acd1_0992	C2	arCOG04124	S	-	Uncharacterized conserved protein	332796282	345	Crenarchaeota	Acidianus hospitalis W1
Acd1_0993	C2	arCOG04123	S	-	Uncharacterized conserved protein	342306118	278	Crenarchaeota	Sulfolobus tokodaii str. 7
Acd1_0994	C2	arCOG04105	S		Uncharacterized conserved protein	15920422	260	Crenarchaeota	Sulfolobus tokodaii str. 7
Acd1_0995	C2	arCOG00053	G	SgbH	3-hexulose-6-phosphate synthase or related protein	15920421	275	Crenarchaeota	Sulfolobus tokodaii str. 7
Acd1_0996	C2	arCOG00063	F	PyrG	CTP synthase (UTP-ammonia lyase)	161751103	110	Crenarchaeota	Sulfolobus tokodaii str. 7
Acd1_0997	C2	arCOG01679	K	ArsR	Transcriptional regulator containing HTH domain, ArsR family	330835701	280	Crenarchaeota	Metallosphaera cuprina Ar-4
Acd1_0998	C2	arCOG00998	K	LSM1	Small nuclear ribonucleoprotein (snRNP) homolog	374632978	570	Crenarchaeota	Metallosphaera yellowstonensis MK1
Acd1_0999	C2	arCOG01678	E	MetK	Archaeal S-adenosylmethionine synthetase	15920416	236	Crenarchaeota	Sulfolobus tokodaii str. 7
Acd1_1000	C2	arCOG04219	R	-	Possible nuclease of RNase H fold, RuvC/YqgF family	70606590	143	Crenarchaeota	Sulfolobus acidocaldarius DSM 639
Acd1_1001	C2	arCOG01181	T	-	RIO-like serine/threonine protein kinase fused to N-terminal HTH domain	15920414	198	Crenarchaeota	Sulfolobus tokodaii str. 7
Acd1_1002	C2	arCOG01695	K	-	Predicted RNA-binding protein homologous to eukaryotic snRNP	15920412	157	Crenarchaeota	Sulfolobus tokodaii str. 7
Acd1_1003	C2	arCOG05042	S	0	Uncharacterized conserved protein	15920411	104	Crenarchaeota	Sulfolobus tokodaii str. 7
Acd1_1004	C2	arCOG04290	R	-	PIN-domain and Zn ribbon	332796294	38.5	Crenarchaeota	Acidianus hospitalis W1
Acd1_1005	C2	arCOG01336	S	AMMECR1	Uncharacterized conserved protein	332796295	358	Crenarchaeota	Acidianus hospitalis W1
Acd1_1006	C2	arCOG01218	O	TrxA	Thiol-disulfide isomerase or thioredoxin	15920408	258	Crenarchaeota	Sulfolobus tokodaii str. 7
Acd1_1007	C2	arCOG01531	K		Transcriptional regulator, contains wHTH domain	15920407	876	Crenarchaeota	Sulfolobus tokodaii str. 7
Acd1_1008	C2	arCOG04048	F	Dcd	Deoxycytidine deaminase	70606582	154	Crenarchaeota	Sulfolobus acidocaldarius DSM 639
Acd1_1009	C2	arCOG01347	L	CDC9	ATP-dependent DNA ligase	15920405	664	Crenarchaeota	Sulfolobus tokodaii str. 7
Acd1_1010	C2	arCOG00545	J	YSH1	Predicted exonuclease of the beta-lactamase fold involved in RNA processing	15920404	205	Crenarchaeota	Sulfolobus tokodaii str. 7
Acd1_1011	C2	arCOG01377	R	-	Phosphodiesterase of AP superfamily	342306112	521	Crenarchaeota	Sulfolobus tokodaii str. 7
Acd1_1012	C2	arCOG08308	S		Uncharacterized conserved protein	342306111	31.6	Crenarchaeota	Sulfolobus tokodaii str. 7
Acd1_1013	C2	arCOG02048	H	HemD	Uroporphyrinogen-III synthase	374632956	627	Crenarchaeota	Metallosphaera yellowstonensis MK1
Acd1_1014	C2	arCOG04299	H	HemC	Porphobilinogen deaminase	15920398	670	Crenarchaeota	Sulfolobus tokodaii str. 7
Acd1_1015	C2	arCOG00918	H	HemL	Glutamate-1-semialdehyde aminotransferase	363548517	45.1	Crenarchaeota	Sulfolobus tokodaii str. 7
Acd1_1016	C2	arCOG04300	H	HemB	Delta-aminolevulinic acid dehydratase	15920396	112	Crenarchaeota	Sulfolobus tokodaii str. 7
Acd1_1017	C2	arCOG01036	H	HemA	Glutamyl-tRNA reductase	146302999	232	Crenarchaeota	Metallosphaera sedula DSM 5348
Acd1_1018	C2	arCOG01044	H	CysG	Siroheme synthase (precorrin-2 oxidase/ferrochelatase domain)	70606572	401	Crenarchaeota	Sulfolobus acidocaldarius DSM 639
Acd1_1019	C2	arCOG04050	L	Exo	5'-3' exonuclease (including N-terminal domain of PolI)	116668192	132	Crenarchaeota	Sulfolobus solfataricus
Acd1_1020	C2	arCOG07192	K		Predicted transcriptional regulator, PadR family	342306105	145	Crenarchaeota	Sulfolobus tokodaii str. 7
Acd1_1021	C2	arCOG01308	O	Cdc48	ATPase of the AAA+ class , CDC48 family	342306104	302	Crenarchaeota	Sulfolobus tokodaii str. 7
Acd1_1022	C2	arCOG01988	J	EFB1	Translation elongation factor EF-1beta	15897128	244	Crenarchaeota	Sulfolobus solfataricus P2
Acd1_1023	C2	arCOG01989	J	-	Predicted Zn-ribbon RNA-binding protein with a function in translation	15920390	149	Crenarchaeota	Sulfolobus tokodaii str. 7
Acd1_1024	C2	arCOG04228	J	pth2	Peptidyl-tRNA hydrolase	15920389	854	Crenarchaeota	Sulfolobus tokodaii str. 7
Acd1_1025	C2	arCOG04252	J	-	tRNA pseudouridine synthase D	15920388	169	Crenarchaeota	Sulfolobus tokodaii str. 7
Acd1_1026	C2	arCOG01761	K	NusA	Transcription elongation factor	227828227	982	Crenarchaeota	Sulfolobus islandicus M.14.25
Acd1_1027	C2	arCOG04049	J	RPL40A	Ribosomal protein L40E	15920384	181	Crenarchaeota	Sulfolobus tokodaii str. 7
Acd1_1028	C2	arCOG02687	G	ProP	Permease of the major facilitator superfamily	15922786	1082	Crenarchaeota	Sulfolobus tokodaii str. 7
tRNAValTAC					tRNAValTAC				
Acd1_1029	C2	arCOG08309	S		Uncharacterized conserved protein	374632872	57.8	Crenarchaeota	Metallosphaera yellowstonensis MK1
Acd1_1030	C2		S		Uncharacterized protein				
Acd1_1031	C2		S		Uncharacterized protein				
Acd1_1032	C2	arCOG05933	R		Rossmann-fold NAD(P)(+)-binding protein	15920380	768	Crenarchaeota	Sulfolobus tokodaii str. 7
Acd1_1033	C2	arCOG00016	G	RbsK	Sugar kinase, ribokinase family	146302944	514	Crenarchaeota	Metallosphaera sedula DSM 5348
Acd1_1034	C2	arCOG05934	S		C2H2-type zinc finger	15920378	212	Crenarchaeota	Sulfolobus tokodaii str. 7
Acd1_1035	C2	arCOG00068	M	GutQ	Predicted sugar phosphate isomerase involved in capsule formation	330835694	479	Crenarchaeota	Metallosphaera cuprina Ar-4
Acd1_1036	C2	arCOG05935	R	-	Predicted ATPase/Helicase	15920376	474	Crenarchaeota	Sulfolobus tokodaii str. 7
Acd1_1037	C2	arCOG05463	S		Uncharacterized conserved protein	70606548	142	Crenarchaeota	Sulfolobus acidocaldarius DSM 639
Acd1_1038	C2	arCOG00495	E	ArgC	Acetylglutamate semialdehyde dehydrogenase	161751104	669	Crenarchaeota	Sulfolobus tokodaii str. 7
Acd1_1039	C2	arCOG00862	E	ArgB	Acetylglutamate kinase	70606550	75.5	Crenarchaeota	Sulfolobus acidocaldarius DSM 639
Acd1_1040	C2	arCOG01580	K	Lrp	Transcriptional regulator, IclR family	332796569	492	Crenarchaeota	Acidianus hospitalis W1
Acd1_1041	C2	arCOG01588	E	LysW	lysine biosynthesis protein LysW	146302952	81.6	Crenarchaeota	Metallosphaera sedula DSM 5348
Acd1_1042	C2	arCOG01589	H	RimK	Glutathione synthase/glutaminyl transferase/alpha-L-glutamate ligase	15920370	96.3	Crenarchaeota	Sulfolobus tokodaii str. 7
Acd1_1043	C2	arCOG00914	E	ArgD	Ornithine/acetylornithine aminotransferase	15920369	144	Crenarchaeota	Sulfolobus tokodaii str. 7
Acd1_1044	C2	arCOG01107	E	ArgE	Acetylornithine deacetylase/Succinyl-diaminopimelate desuccinylase or related deacylase	332796573	1166	Crenarchaeota	Acidianus hospitalis W1
Acd1_1045	C2	arCOG01532	I	UppS	Undecaprenyl pyrophosphate synthase	15920367	680	Crenarchaeota	Sulfolobus tokodaii str. 7
Acd1_1046	C2	arCOG04154	J	RPS8A	Ribosomal protein S8E	70606557	124	Crenarchaeota	Sulfolobus acidocaldarius DSM 639
Acd1_1047	C2	arCOG01217	U	SEC65	Signal recognition particle 19 kDa protein	332796576	561	Crenarchaeota	Acidianus hospitalis W1
tRNALysTTT					tRNALysTTT				
Acd1_1048	C2	arCOG05937	E	IlvB	Acetolactate synthase large subunit or other thiamine pyrophosphate-requiring enzyme	15920382	38.5	Crenarchaeota	Sulfolobus tokodaii str. 7
tRNACysGCA					tRNACysGCA				
Acd1_1049	C2	arCOG04119	R	-	Predicted nucleotidyltransferase	15920383	324	Crenarchaeota	Sulfolobus tokodaii str. 7
Acd1_1050	C2	arCOG07272	M	SlaB	S-layer protein SlaB	15922525	306	Crenarchaeota	Sulfolobus tokodaii str. 7
Acd1_1051	C2	arCOG06039	M	SlaA	S-layer protein SlaA	70608059	644	Crenarchaeota	Sulfolobus acidocaldarius DSM 639
Acd1_1052	C2	arCOG07273	R		Predicted periplasmic protein	229579620	224	Crenarchaeota	Sulfolobus islandicus Y.G.57.14
Acd1_1053	C2	arCOG04137	H	SAM1	S-adenosylhomocysteine hydrolase	15920541	187	Crenarchaeota	Sulfolobus tokodaii str. 7
tRNAMetCAT					tRNAMetCAT				
Acd1_1054	C2	arCOG00980	O	SlpA	FKBP-type peptidyl-prolyl cis-trans isomerase 2	15920545	452	Crenarchaeota	Sulfolobus tokodaii str. 7
Acd1_1055	C2	arCOG00982	C	GldA	Glycerol dehydrogenase or related enzyme	15920544	232	Crenarchaeota	Sulfolobus tokodaii str. 7
Acd1_1056	C2	arCOG00543	R	-	Predicted metal-dependent RNase, consists of a metallo-beta-lactamase domain and an RNA-binding KH domain	15920543	215	Crenarchaeota	Sulfolobus tokodaii str. 7
tRNAValGAC					tRNAValGAC				
Acd1_1057	C2	arCOG02862	S	-	Predicted membrane protein, DoxD family	22213646	630	Crenarchaeota	Acidianus ambivalens
Acd1_1058	C2	arCOG05342	C		Thiosulphate:quinone oxidoreductase (TQO) small subunit DoxA	229578710	211	Crenarchaeota	Sulfolobus islandicus Y.G.57.14
Acd1_1059	C2	arCOG06050	S	-	Predicted membrane protein	332797149	1102	Crenarchaeota	Acidianus hospitalis W1
Acd1_1060	C2	arCOG08475	S		Uncharacterized conserved protein	342306268	184	Crenarchaeota	Sulfolobus tokodaii str. 7
Acd1_1062	C2	arCOG05923	S		Uncharacterized conserved protein	70606215	332	Crenarchaeota	Sulfolobus acidocaldarius DSM 639
Acd1_1061	C2		S		Uncharacterized protein				
tRNAValCAC					tRNAValCAC				
Acd1_1063	C2	arCOG01019	R	-	SpoU rRNA Methylase family enzyme	70606457	340	Crenarchaeota	Sulfolobus acidocaldarius DSM 639
Acd1_1064	C2	arCOG04157	L	Dcm	Site-specific DNA methylase	332796494	94	Crenarchaeota	Acidianus hospitalis W1
Acd1_1065	C2	arCOG04270	K	TFA1	Transcription initiation factor IIE, alpha subunit	15920533	224	Crenarchaeota	Sulfolobus tokodaii str. 7
Acd1_1066	C2	arCOG01857	R	-	SpoU rRNA Methylase family enzyme	15920532	112	Crenarchaeota	Sulfolobus tokodaii str. 7
Acd1_1067	C2	arCOG00353	R	HflX	GTP-binding protein protease modulator	15920531	286	Crenarchaeota	Sulfolobus tokodaii str. 7
Acd1_1068	C2	arCOG01863	K	-	Predicted transcription factor, homolog of eukaryotic MBF1	70606462	298	Crenarchaeota	Sulfolobus acidocaldarius DSM 639
Acd1_1069	C2	arCOG01306	O	RPT1	ATP-dependent 26S proteasome regulatory subunit	15897214	117	Crenarchaeota	Sulfolobus solfataricus P2
Acd1_1070	C2	arCOG00991	J	-	tRNA modification protein, contains pre-PUA and PUA domains	15920528	130	Crenarchaeota	Sulfolobus tokodaii str. 7
Acd1_1071	C2	arCOG00347	R	-	Archaeal enzyme of ATP-grasp superfamily	332796488	240	Crenarchaeota	Acidianus hospitalis W1
Acd1_1072	C2	arCOG00989	J	Tgt	Queuine/archaeosine tRNA-ribosyltransferase	15920526	577	Crenarchaeota	Sulfolobus tokodaii str. 7
Acd1_1073	C2	arCOG00999	K	LSM1	Small nuclear ribonucleoprotein (snRNP) homolog	70606467	131	Crenarchaeota	Sulfolobus acidocaldarius DSM 639
Acd1_1074	C2	arCOG04271	K	RPB8	DNA-directed RNA polymerase, subunit RPB8	15920524	826	Crenarchaeota	Sulfolobus tokodaii str. 7
Acd1_1075	C2	arCOG00970	O	PRE1	20S proteasome, beta subunit	332796484	263	Crenarchaeota	Acidianus hospitalis W1
Acd1_1076	C2	arCOG00404	J	HisS	Histidyl-tRNA synthetase	15920522	154	Crenarchaeota	Sulfolobus tokodaii str. 7
Acd1_1077	C2	arCOG01702	J	SEN2	tRNA splicing endonuclease	332796482	276	Crenarchaeota	Acidianus hospitalis W1
Acd1_1078	C2	arCOG05931	K	0	Homolog of transcription initiation factor TFIIIB, contains Zn-ribbon domain	123475449	196	Parabasalia	Trichomonas vaginalis G3
Acd1_1079	C2	arCOG01257	O	GroL	Chaperonin GroEL (HSP60 family)	15920519	530	Crenarchaeota	Sulfolobus tokodaii str. 7
Acd1_1080	C2	arCOG00286	L	-	HerA helicase	70606474	172	Crenarchaeota	Sulfolobus acidocaldarius DSM 639
Acd1_1081	C2	arCOG07188	S		Uncharacterized conserved protein	146305008	77	Crenarchaeota	Metallosphaera sedula DSM 5348
Acd1_1082	C2	arCOG00536	H	MoaD	Molybdopterin converting factor, small subunit	374633128	396	Crenarchaeota	Metallosphaera yellowstonensis MK1
Acd1_1083	C2	arCOG01885	J	RPS17A	Ribosomal protein S17E	332796476	191	Crenarchaeota	Acidianus hospitalis W1
Acd1_1084	C2	arCOG04180	G	-	Bifunctional fructose-1,6-bisphosphate aldolase/phosphatase FBPA/FBPase	15920515	136	Crenarchaeota	Sulfolobus tokodaii str. 7
Acd1_1085	C2	arCOG00187	R	-	Predicted ATPase, RNase L inhibitor (RLI) homolog	332796474	171	Crenarchaeota	Acidianus hospitalis W1
Acd1_1086	C2		S		Uncharacterized protein				
tRNAArgCCG					tRNAArgCCG				
Acd1_1087	C2	arCOG01120	S	-	SWIM zinc finger	15920499	123	Crenarchaeota	Sulfolobus tokodaii str. 7
Acd1_1088	C2		S		Uncharacterized protein				
Acd1_1089	C2	arCOG04136	J	-	Transcription elongation factor Elf1, Zn ribbon-containing protein	15920500	61.6	Crenarchaeota	Sulfolobus tokodaii str. 7
Acd1_1090	C2	arCOG01085	R	-	Predicted phosphate-binding enzyme, TIM-barrel fold	330835891	215	Crenarchaeota	Metallosphaera cuprina Ar-4

Acd1_1091	C2	arCOG02957	U	SBH1	Preprotein translocase subunit Sec61beta	15920503	227	Crenarchaeota	Sulfolobus tokodaii str. 7
RNaseP RNA					RNaseP RNA				
Acd1_1092	C2	arCOG04733	S	-	Uncharacterized conserved protein, contains Fer4-like metal-binding domain ortholog of eukaryotic RNase L inhibitor RLI N-terminal domain	15897473	62	Crenarchaeota	Sulfolobus solfataricus P2
Acd1_1093	C2	arCOG01117	K	Lrp	Lrp/AsnC family C-terminal domain	15920505	97.4	Crenarchaeota	Sulfolobus tokodaii str. 7
Acd1_1094	C2	arCOG01115	J	TiaS	tRNA(Ile2) 2-agmatinylcytidine synthetase; containing Zn-ribbon domain and OB-fold domain	374633141	338	Crenarchaeota	Metallosphaera yellowstonensis MK1
tRNAAlaGGC					tRNAAlaGGC				
Acd1_1095	C2	arCOG00467	L	CDC6	Cdc6-related protein, AAA superfamily ATPase	332796464	537	Crenarchaeota	Acidianus hospitalis W1
Acd1_1096	C2	arCOG00928	L	-	Endonuclease V homolog	363548495	316	Crenarchaeota	Sulfolobus tokodaii str. 7
Acd1_1097	C2	arCOG00773	I	-	Acyl-CoA hydrolase	15920495	154	Crenarchaeota	Sulfolobus tokodaii str. 7
Acd1_1098	C2	arCOG00972	H	NadR	Nicotinamide mononucleotide adenylyltransferase	332796461	122	Crenarchaeota	Acidianus hospitalis W1
Acd1_1099	C2	arCOG04309	F	-	S-adenosyl-l-methionine hydroxide adenosyltransferase	374633147	42	Crenarchaeota	Metallosphaera yellowstonensis MK1
Acd1_1100	C2	arCOG01723	F	CyaB	Adenylate cyclase, class 2 (thermophilic)	15920492	114	Crenarchaeota	Sulfolobus tokodaii str. 7
Acd1_1101	C2	arCOG00973	J	Sun	tRNA or rRNA cytosine-C5-methylase	332796458	99.4	Crenarchaeota	Acidianus hospitalis W1
Acd1_1102	C2	arCOG00993	J	NIP7	Protein involved in ribosomal biogenesis, contains PUA domain	342306136	327	Crenarchaeota	Sulfolobus tokodaii str. 7
Acd1_1103	C2	arCOG00415	L	RecA	RecA/RadA recombinase	15920489	646	Crenarchaeota	Sulfolobus tokodaii str. 7
Acd1_1104	C2	arCOG00419	F	Hit	HIT family hydrolase	227828153	353	Crenarchaeota	Sulfolobus islandicus M.14.25
Acd1_1105	C2	arCOG01431	E	IlvA	Threonine dehydratase	15920487	327	Crenarchaeota	Sulfolobus tokodaii str. 7
Acd1_1106	C2	arCOG00255	E	PheA	Prephenate dehydratase	15920486	414	Crenarchaeota	Sulfolobus tokodaii str. 7
Acd1_1107	C2	arCOG01628	K	Lrp	Transcriptional regulator (Lrp/AsnC family)	332796452	488	Crenarchaeota	Acidianus hospitalis W1
Acd1_1108	C2	arCOG02231	R	-	Uncharacterized FAD-dependent dehydrogenase	15920484	575	Crenarchaeota	Sulfolobus tokodaii str. 7
Acd1_1109	C2	arCOG04387	F	PurA	Adenylosuccinate synthase	15920483	300	Crenarchaeota	Sulfolobus tokodaii str. 7
Acd1_1110	C2	arCOG04346	F	-	5-formaminoimidazole-4-carboxamide-1-beta-D-ribofuranosyl 5'-monophosphate synthetase (purine biosynthesis)	15920482	456	Crenarchaeota	Sulfolobus tokodaii str. 7
Acd1_1111	C2	arCOG04346	F	-	5-formaminoimidazole-4-carboxamide-1-beta-D-ribofuranosyl 5'-monophosphate synthetase (purine biosynthesis)	15920481	514	Crenarchaeota	Sulfolobus tokodaii str. 7
Acd1_1112	C2	arCOG01747	F	PurB	Adenylosuccinate lyase	15920480	379	Crenarchaeota	Sulfolobus tokodaii str. 7
tRNAThrTGT					tRNAThrTGT				
Acd1_1113	C2	arCOG00638	H	ThiL	Thiamine monophosphate kinase	15920479	92	Crenarchaeota	Sulfolobus tokodaii str. 7
Acd1_1114	C2	arCOG00933	R	-	Radical SAM superfamily enzyme	15920478	575	Crenarchaeota	Sulfolobus tokodaii str. 7
Acd1_1115	C2	arCOG05932	R	GloB	Zn-dependent hydrolase, glyoxylase family	70606505	372	Crenarchaeota	Sulfolobus acidocaldarius DSM 639
Acd1_1116	C2	arCOG00031	F	Apt	Adenine/guanine phosphoribosyltransferase or related PRPP-binding protein	70606504	388	Crenarchaeota	Sulfolobus acidocaldarius DSM 639
Acd1_1117	C2	arCOG01718	J	GatB	Asp-tRNAAsn/Glu-tRNAGln amidotransferase B subunit	15920474	325	Crenarchaeota	Sulfolobus tokodaii str. 7
Acd1_1118	C2	arCOG04314	J	RPS28A	Ribosomal protein S28E/S33	332796438	558	Crenarchaeota	Acidianus hospitalis W1
Acd1_1119	C2	arCOG01950	J	RPL24A	Ribosomal protein L24E	15897177	286	Crenarchaeota	Sulfolobus solfataricus P2
Acd1_1120	C2	arCOG04313	F	Ndk	Nucleoside diphosphate kinase	119364621	1279	Crenarchaeota	Sulfolobus tokodaii str. 7
Acd1_1121	C2	arCOG01560	J	InfB	Translation initiation factor 2 (IF-2; GTPase)	15920469	449	Crenarchaeota	Sulfolobus tokodaii str. 7
Acd1_1122	C2	arCOG00853	C	SfcA	Malic enzyme	15920468	117	Crenarchaeota	Sulfolobus tokodaii str. 7
Acd1_1123	C2	arCOG04258	K	RPB5	DNA-directed RNA polymerase, subunit H, RpoH/RPB5	46668	331	Crenarchaeota	Sulfolobus acidocaldarius
Acd1_1124	C2	arCOG01762	K	RpoB/Rpo2	DNA-directed RNA polymerase subunit B	15920466	197	Crenarchaeota	Sulfolobus tokodaii str. 7
Acd1_1125	C2	arCOG04257	K	RpoC/Rpo3	DNA-directed RNA polymerase subunit A'	15897171	486	Crenarchaeota	Sulfolobus solfataricus P2
Acd1_1126	C2	arCOG04256	K	RpoC/Rpo11	DNA-directed RNA polymerase subunit A''	227828175	183	Crenarchaeota	Sulfolobus islandicus M.14.25
Acd1_1127	C2	arCOG01752	J	RPL30	Ribosomal protein L30E	15920463	368	Crenarchaeota	Sulfolobus tokodaii str. 7
Acd1_1128	C2	arCOG01760	K	NusA	Transcription elongation factor	161751102	215	Crenarchaeota	Sulfolobus tokodaii str. 7
Acd1_1129	C2	arCOG04255	J	RpsL	Ribosomal protein S12	15920461	227	Crenarchaeota	Sulfolobus tokodaii str. 7
Acd1_1130	C2	arCOG01759	S	-	Uncharacterized conserved protein	15920460	249	Crenarchaeota	Sulfolobus tokodaii str. 7
Acd1_1131	C2	arCOG04254	J	RpsG	Ribosomal protein S7	15920459	142	Crenarchaeota	Sulfolobus tokodaii str. 7
Acd1_1132	C2	arCOG01561	J	TEF1	Translation elongation factor EF-1alpha (GTPase)	15920458	129	Crenarchaeota	Sulfolobus tokodaii str. 7
Acd1_1133	C2	arCOG01758	J	RpsJ	Ribosomal protein S10	15920457	168	Crenarchaeota	Sulfolobus tokodaii str. 7
tRNASerGGA					tRNASerGGA				
tRNAAspGTC					tRNAAspGTC				
Acd1_1134	C2		S		Uncharacterized protein				
Acd1_1135	C2	arCOG03708	R		HEPN domain	332797315	107	Crenarchaeota	Acidianus hospitalis W1
Acd1_1136	C2	arCOG04397	P	AmtB	Ammonia permease	15920882	124	Crenarchaeota	Sulfolobus tokodaii str. 7
Acd1_1137	C2	arCOG00571	C	SdhA	Succinate dehydrogenase/fumarate reductase, flavoprotein subunit	15920713	220	Crenarchaeota	Sulfolobus tokodaii str. 7
Acd1_1138	C2	arCOG00962	C	FrdB	Succinate dehydrogenase/fumarate reductase, Fe-S protein subunit	70606765	299	Crenarchaeota	Sulfolobus acidocaldarius DSM 639
Acd1_1139	C2	arCOG00338	C	SdhC	Succinate dehydrogenase subunit C	3378542	135	Crenarchaeota	Acidianus ambivalens
Acd1_1140	C2	arCOG05375	C		Archaeal succinate dehydrogenase subunit D	15920716	291	Crenarchaeota	Sulfolobus tokodaii str. 7
tRNATyrGTA					tRNATyrGTA				
Acd1_1141	C2	arCOG00998	K	LSM1	Small nuclear ribonucleoprotein (snRNP) homolog	332796496	50.4	Crenarchaeota	Acidianus hospitalis W1
Acd1_1142	C2	arCOG04126	J	RPL37A	Ribosomal protein L37E	15920546	223	Crenarchaeota	Sulfolobus tokodaii str. 7
Acd1_1143	C2	arCOG00050	E	SpeE	Spermidine synthase	15920547	115	Crenarchaeota	Sulfolobus tokodaii str. 7
tRNASerCGA					tRNASerCGA				
tRNALeuCAG					tRNALeuCAG				
Acd1_1144	C2	arCOG04129	J	RPL21A	Ribosomal protein L21E	15897652	84	Crenarchaeota	Sulfolobus solfataricus P2
Acd1_1145	C2	arCOG01016	K	-	DNA-directed RNA polymerase, subunit F (rpoF)	15897651	93.2	Crenarchaeota	Sulfolobus solfataricus P2
Acd1_1146	C2	arCOG04130	J	-	Predicted RNA-binding protein	227830360	120	Crenarchaeota	Sulfolobus islandicus L.S.2.15
Acd1_1147	C2	arCOG04131	J	KsgA	Dimethyladenosine transferase (rRNA methylation)	15920668	270	Crenarchaeota	Sulfolobus tokodaii str. 7
Acd1_1148	C2	arCOG00109	J	HemK	Methylase of polypeptide chain release factors	332796503	43.9	Crenarchaeota	Acidianus hospitalis W1
Acd1_1149	C2	arCOG01018	J	LasT	rRNA methylase	15920666	140	Crenarchaeota	Sulfolobus tokodaii str. 7
Acd1_1150	C2	arCOG04186	J	RPS1A	Ribosomal protein S3AE	15897646	344	Crenarchaeota	Sulfolobus solfataricus P2
Acd1_1151	C2	arCOG00666	M	GCD1	N-acetylglucosamine-1-phosphate uridyltransferase	15920664	358	Crenarchaeota	Sulfolobus tokodaii str. 7
Acd1_1152	C2	arCOG00357	J	-	Predicted GTPase, probable translation factor	15920663	355	Crenarchaeota	Sulfolobus tokodaii str. 7
Acd1_1153	C2	arCOG04209	J	RPL15A	Ribosomal protein L15E	15920662	226	Crenarchaeota	Sulfolobus tokodaii str. 7
Acd1_1154	C2	arCOG01042	J	-	Exosome subunit, RNA binding protein with dsRBD fold	332796509	587	Crenarchaeota	Acidianus hospitalis W1
Acd1_1155	C2	arCOG00307	J	RPP1	RNase P/RNase MRP subunit p30	342306200	355	Crenarchaeota	Sulfolobus tokodaii str. 7
Acd1_1156	C2	arCOG01365	J	POP5	RNase P/RNase MRP subunit POP5	15920659	143	Crenarchaeota	Sulfolobus tokodaii str. 7
Acd1_1157	C2	arCOG00971	O	PRE1	20S proteasome, alpha subunit	21362819	252	Crenarchaeota	Sulfolobus tokodaii str. 7
Acd1_1158	C2	arCOG04187	J	-	Predicted exosome subunit	15920657	748	Crenarchaeota	Sulfolobus tokodaii str. 7
Acd1_1159	C2	arCOG00678	J	RRP4	RNA-binding protein Rrp4 or related protein (contain S1 domain and KH domain)	15920656	312	Crenarchaeota	Sulfolobus tokodaii str. 7
Acd1_1160	C2	arCOG01575	J	Rph	Ribonuclease PH	15920655	198	Crenarchaeota	Sulfolobus tokodaii str. 7
Acd1_1161	C2	arCOG01574	J	-	RNase PH-related exoribonuclease	15920654	202	Crenarchaeota	Sulfolobus tokodaii str. 7
Acd1_1162	C2	arCOG04208	J	RPL43A	Ribosomal protein L37AE/L43A	227827679	250	Crenarchaeota	Sulfolobus islandicus M.14.25
Acd1_1163	C2	arCOG03247	J	IMP4	Predicted exosome subunit/U3 small nucleolar ribonucleoprotein (snoRNP) component, contains IMP4 domain	70606422	151	Crenarchaeota	Sulfolobus acidocaldarius DSM 639
Acd1_1164	C2	arCOG04414	J	Pcc1	Subunit of KEOPS complex (Cgi121BUD32KAE1)	146302866	116	Crenarchaeota	Metallosphaera sedula DSM 5348
Acd1_1165	C2	arCOG01342	O	GimC	Prefoldin, chaperonin cofactor	15920651	250	Crenarchaeota	Sulfolobus tokodaii str. 7
Acd1_1166	C2	arCOG04206	L	MUS81	ERCC4-type nuclease	15920650	198	Crenarchaeota	Sulfolobus tokodaii str. 7
Acd1_1167	C2	arCOG01296	O	TrxB	Thioredoxin reductase	15920649	245	Crenarchaeota	Sulfolobus tokodaii str. 7
Acd1_1168	C2	arCOG01559	J	FusA	Translation elongation factor G, EF-G (GTPase)	21263534	484	Crenarchaeota	Sulfolobus tokodaii str. 7
Acd1_1169	C2	arCOG02293	R	-	HAD superfamily hydrolase	15920647	412	Crenarchaeota	Sulfolobus tokodaii str. 7
Acd1_1170	C2	arCOG02960	R	-	Predicted aminopeptidase, lap family	15920646	402	Crenarchaeota	Sulfolobus tokodaii str. 7
Acd1_1171	C2	arCOG04458	R	-	Uncharacterized protein of DIM6/NTAB family	227827687	340	Crenarchaeota	Sulfolobus islandicus M.14.25
Acd1_1172	C2	arCOG01163	C	LeuB	Isocitrate/isopropylmalate dehydrogenase	15920644	242	Crenarchaeota	Sulfolobus tokodaii str. 7
Acd1_1173	C2	arCOG00807	J	IleS	Isoleucyl-tRNA synthetase	161751096	412	Crenarchaeota	Sulfolobus tokodaii str. 7
Acd1_1174	C2	arCOG04069	S	-	Uncharacterized conserved protein	227827690	309	Crenarchaeota	Sulfolobus islandicus M.14.25
Acd1_1175	C2	arCOG04070	J	RplC	Ribosomal protein L3	15920641	568	Crenarchaeota	Sulfolobus tokodaii str. 7
Acd1_1176	C2	arCOG04071	J	RplD	Ribosomal protein L4	70606411	605	Crenarchaeota	Sulfolobus acidocaldarius DSM 639
Acd1_1177	C2	arCOG04072	J	RplW	Ribosomal protein L23	18313003	279	Crenarchaeota	Pyrobaculum aerophilum str. IM2
Acd1_1178	C2	arCOG04067	J	RplB	Ribosomal protein L2	161751097	426	Crenarchaeota	Sulfolobus tokodaii str. 7
Acd1_1179	C2	arCOG04099	J	RpsS	Ribosomal protein S19	15920637	130	Crenarchaeota	Sulfolobus tokodaii str. 7
Acd1_1180	C2	arCOG04098	J	RplV	Ribosomal protein L22	15920636	408	Crenarchaeota	Sulfolobus tokodaii str. 7
Acd1_1181	C2	arCOG04097	J	RpsC	Ribosomal protein S3	15920635	30	Crenarchaeota	Sulfolobus tokodaii str. 7
Acd1_1182	C2	arCOG00785	J	RpmC	Ribosomal protein L29	20808652	1390	Firmicutes	Thermoanaerobacter tengcongensis MB4
Acd1_1183	C2	arCOG00784	J	POP4	RNase P/RNase MRP subunit p29	218884478	120	Crenarchaeota	Desulfurococcus kamchatkensis 1221n
Acd1_1184	C2	arCOG04096	J	RpsQ	Ribosomal protein S17	332796537	127	Crenarchaeota	Acidianus hospitalis W1
Acd1_1185	C2	arCOG04095	J	RplN	Ribosomal protein L14	15920632	84	Crenarchaeota	Sulfolobus tokodaii str. 7
Acd1_1186	C2	arCOG04094	J	RplX	Ribosomal protein L24	70606401	84.7	Crenarchaeota	Sulfolobus acidocaldarius DSM 639
Acd1_1187	C2	arCOG04093	J	RPS4A	Ribosomal protein S4E	161751098	161	Crenarchaeota	Sulfolobus tokodaii str. 7
Acd1_1188	C2	arCOG04092	J	RplE	Ribosomal protein L5	15920629	112	Crenarchaeota	Sulfolobus tokodaii str. 7
Acd1_1189	C2	arCOG00782	J	RpsN	Ribosomal protein S14	161751099	148	Crenarchaeota	Sulfolobus tokodaii str. 7
Acd1_1190	C2	arCOG04091	J	RpsH	Ribosomal protein S8	15920627	291	Crenarchaeota	Sulfolobus tokodaii str. 7
Acd1_1191	C2	arCOG04090	J	RplF	Ribosomal protein L6P	15920626	1158	Crenarchaeota	Sulfolobus tokodaii str. 7
Acd1_1192	C2	arCOG00781	J	RPL32	Ribosomal protein L32E	3914750	269	Crenarchaeota	Sulfolobus acidocaldarius DSM 639
Acd1_1193	C2	arCOG04089	J	RPL19A	Ribosomal protein L19E	374633051	89.7	Crenarchaeota	Metallosphaera yellowstonensis MK1
Acd1_1194	C2	arCOG04088	J	RplR	Ribosomal protein L18	15920623	374	Crenarchaeota	Sulfolobus tokodaii str. 7
Acd1_1195	C2	arCOG04087	J	RpsE	Ribosomal protein S5	15920622	255	Crenarchaeota	Sulfolobus tokodaii str. 7
Acd1_1196	C2	arCOG04086	J	RpmD	Ribosomal protein L30	15920621	137	Crenarchaeota	Sulfolobus tokodaii str. 7
Acd1_1197	C2	arCOG00779	J	RplO	Ribosomal protein L15	15920620	691	Crenarchaeota	Sulfolobus tokodaii str. 7
Acd1_1198	C2	arCOG04169	U	SecY	Preprotein translocase subunit SecY	15920619	46.6	Crenarchaeota	Sulfolobus tokodaii str. 7
Acd1_1199	C2	arCOG01039	F	AdkA	Archaeal adenylate kinase	15920618	437	Crenarchaeota	Sulfolobus tokodaii str. 7
Acd1_1200	C2	arCOG07287	S		Uncharacterized conserved protein	15920617	538	Crenarchaeota	Sulfolobus tokodaii str. 7
Acd1_1201	C2	arCOG01037	F	Cmk	Cytidylate kinase	15920615	113	Crenarchaeota	Sulfolobus tokodaii str. 7
Acd1_1202	C2	arCOG04068	S		Uncharacterized conserved protein	284174920	385	Crenarchaeota	Sulfolobus solfataricus 98/2
Acd1_1203	C2	arCOG05930	S		Uncharacterized conserved protein	146302905	417	Crenarchaeota	Metallosphaera sedula DSM 5348
Acd1_1204	C2		S		Uncharacterized protein	70606382	35	Crenarchaeota	Sulfolobus acidocaldarius DSM 639
Acd1_1205	C2	arCOG04055	R	-	SHS2 domain protein implicated in nucleic acid metabolism	15897595	115	Crenarchaeota	Sulfolobus solfataricus P2
tRNAPheGAA					tRNAPheGAA				
Acd1_1206	C2	arCOG00458	R	-	Predicted Zn-dependent protease	342306179	249	Crenarchaeota	Sulfolobus tokodaii str. 7
Acd1_1207	C2	arCOG01147	R	Icc	Predicted phosphohydrolase	15920802	862	Crenarchaeota	Sulfolobus tokodaii str. 7
Acd1_1208	C2	arCOG04253	S	-	Uncharacterized conserved protein	15920803	227	Crenarchaeota	Sulfolobus tokodaii str. 7
Acd1_1209	C2	arCOG04237	C	GltA	Citrate synthase	15920804	122	Crenarchaeota	Sulfolobus tokodaii str. 7
Acd1_1210	C2	arCOG02640	P	-	Phosphate transport regulator (distant homolog of PhoU)	332797781	475	Crenarchaeota	Acidianus hospitalis W1
Acd1_1211	C2	arCOG02267	P	PitA	Phosphate/sulphate permease	330834582	372	Crenarchaeota	Metallosphaera cuprina Ar-4
Acd1_1212	C2	arCOG01010	K	StbD	Antitoxin (DNA-binding domain) of toxin-antitoxin stability system	124028117	192	Crenarchaeota	Hyperthermus butylicus DSM 5456
Acd1_1213	C2	arCOG00553	L	-	Replicative SFII helicase	15920808	173	Crenarchaeota	Sulfolobus tokodaii str. 7

Acd1_1214	C2	arCOG00087	F	GuaA	GMP synthase - Glutamine amidotransferase domain	15920795	259	Crenarchaeota	Sulfolobus tokodaii str. 7
Acd1_1215	C2	arCOG00087	F	GuaA	GMP synthase - Glutamine amidotransferase domain	70606367	403	Crenarchaeota	Sulfolobus acidocaldarius DSM 639
Acd1_1216	C2	arCOG01072	L	MutT	NUDIX family hydrolase	70606369	976	Crenarchaeota	Sulfolobus acidocaldarius DSM 639
Acd1_1217	C2	arCOG04081	K	TIP49	DNA helicase TIP49, TBP-interacting protein	70606370	109	Crenarchaeota	Sulfolobus acidocaldarius DSM 639
Acd1_1218	C2	arCOG00014	G	RbsK	Sugar kinase, ribokinase family	15920792	191	Crenarchaeota	Sulfolobus tokodaii str. 7
Acd1_1219	C2	arCOG05929	S		Uncharacterized conserved protein	15899192	414	Crenarchaeota	Sulfolobus solfataricus P2
Acd1_1220	C2	arCOG01457	R	AdhP	D-arabinose 1-dehydrogenase or another Zn-dependent alcohol dehydrogenase	15920787	97.8	Crenarchaeota	Sulfolobus tokodaii str. 7
Acd1_1221	C2	arCOG04125	J	RCL1	RNA 3'-terminal phosphate cyclase	15920788	632	Crenarchaeota	Sulfolobus tokodaii str. 7
Acd1_1222	C2	arCOG01909	E	GlnA	Glutamine synthetase	374631992	575	Crenarchaeota	Metallosphaera yellowstonensis MK1
Acd1_1223	C2	arCOG00033	J	Trm5	Wybutosine (yW) biosynthesis enzyme, Trm5 methyltransferase	15899187	146	Crenarchaeota	Sulfolobus solfataricus P2
Acd1_1224	C2	arCOG00695	F	SsnA	Cytosine deaminase or related metal-dependent hydrolase	15920784	701	Crenarchaeota	Sulfolobus tokodaii str. 7
Acd1_1225	C2		S		Uncharacterized protein				
Acd1_1226	C2	arCOG07321	S		Uncharacterized conserved protein	146303390	35.8	Crenarchaeota	Metallosphaera sedula DSM 5348
Acd1_1227	C2	arCOG00644	H	CysG	Uroporphyrinogen-III methylase	332797896	236	Crenarchaeota	Acidianus hospitalis W1
Acd1_1228	C2	arCOG01926	C	CoxM	Aerobic-type carbon monoxide dehydrogenase, middle subunit CoxM/CutM homolog	374632001	467	Crenarchaeota	Metallosphaera yellowstonensis MK1
Acd1_1229	C2	arCOG01925	C	CoxS	Aerobic-type carbon monoxide dehydrogenase, small subunit CoxS/CutS homolog	15920779	116	Crenarchaeota	Sulfolobus tokodaii str. 7
Acd1_1230	C2	arCOG01873	H	MobA	GT-A family glycosyltransferase involved in molybdopterin guanine dinucleotide biosynthesis	307595190	304	Crenarchaeota	Vulcanisaeta distributa DSM 14429
Acd1_1231	C2	arCOG01928	C	-	Carbon monoxide dehydrogenase subunit G, CoxG	332797890	109	Crenarchaeota	Acidianus hospitalis W1
Acd1_1232	C2	arCOG01562	R	GTPBP1	GTPase	15899178	134	Crenarchaeota	Sulfolobus solfataricus P2
Acd1_1233	C2	arCOG04265	R	-	C4-type Zn-finger protein	15920774	467	Crenarchaeota	Sulfolobus tokodaii str. 7
Acd1_1234	C2	arCOG01833	O	IbpA	Molecular chaperone (HSP20 family)	15920773	572	Crenarchaeota	Sulfolobus tokodaii str. 7
Acd1_1235	C2	arCOG02706	E	GloA	Lactoylglutathione lyase or related enzyme	15920772	124	Crenarchaeota	Sulfolobus tokodaii str. 7
Acd1_1236	C2	arCOG04232	I	Sbm	Methylmalonyl-CoA mutase	15920771	238	Crenarchaeota	Sulfolobus tokodaii str. 7
Acd1_1237	C2	arCOG00040	R	-	Predicted phosphoribosyltransferase	227826623	329	Crenarchaeota	Sulfolobus islandicus M.14.25
Acd1_1238	C2	arCOG01378	R	-	Uncharacterized protein of the AP superfamily	332797883	192	Crenarchaeota	Acidianus hospitalis W1
Acd1_1239	C2		S		Uncharacterized protein				
Acd1_1240	C2	arCOG01308	O	Cdc48	ATPase of the AAA+ class , CDC48 family	15920767	428	Crenarchaeota	Sulfolobus tokodaii str. 7
Acd1_1241	C2	arCOG01349	G	SuhB	Archaeal fructose-1,6-bisphosphatase or related enzyme of inositol monophosphatase family	15920766	478	Crenarchaeota	Sulfolobus tokodaii str. 7
Acd1_1242	C2	arCOG01296	O	TrxB	Thioredoxin reductase	15920765	107	Crenarchaeota	Sulfolobus tokodaii str. 7
Acd1_1243	C2	arCOG01978	H	FolP	Dihydropteroate synthase or related enzyme	15899167	272	Crenarchaeota	Sulfolobus solfataricus P2
Acd1_1244	C2	arCOG05949	S		Uncharacterized conserved protein	15920763	58.9	Crenarchaeota	Sulfolobus tokodaii str. 7
Acd1_1245	C2	arCOG02172	H	-	6-pyruvoyl-tetrahydropterin synthase	15899165	478	Crenarchaeota	Sulfolobus solfataricus P2
Acd1_1247	C2	arCOG05950	S		Uncharacterized conserved protein	70606720	621	Crenarchaeota	Sulfolobus acidocaldarius DSM 639
Acd1_1246	C2	arCOG05951	S		Uncharacterized conserved protein	15920760	136	Crenarchaeota	Sulfolobus tokodaii str. 7
Acd1_1248	C2	arCOG05952	S		Uncharacterized conserved protein	15920759	168	Crenarchaeota	Sulfolobus tokodaii str. 7
Acd1_1249	C2	arCOG07195	S		Uncharacterized conserved protein	15899161	242	Crenarchaeota	Sulfolobus solfataricus P2
Acd1_1250	C2	arCOG04303	R	-	Uncharacterized Rossmann fold enzyme	15899160	436	Crenarchaeota	Sulfolobus solfataricus P2
Acd1_1251	C2	arCOG02092	E	LeuA	Isopropylmalate/homocitrate/citramalate synthase	70606725	129	Crenarchaeota	Sulfolobus acidocaldarius DSM 639
Acd1_1252	C2	arCOG00497	R	-	Zn-dependent hydrolase of the beta-lactamase fold	15899158	82.4	Crenarchaeota	Sulfolobus solfataricus P2
Acd1_1253	C2	arCOG03845	V	-	Predicted antitoxin, copG family	332797868	0	Crenarchaeota	Acidianus hospitalis W1
Acd1_1254	C2	arCOG04165	S		Uncharacterized conserved protein	342306234	80.1	Crenarchaeota	Sulfolobus tokodaii str. 7
Acd1_1255	C2	arCOG01117	K	Lrp	Lrp/AsnC family C-terminal domain	15920753	59.3	Crenarchaeota	Sulfolobus tokodaii str. 7
Acd1_1256	C2	arCOG01462	C	NatB	ABC-type Na+ efflux pump, permease component	15920752	128	Crenarchaeota	Sulfolobus tokodaii str. 7
Acd1_1257	C2	arCOG00194	V	CcmA	ABC-type multidrug transport system, ATPase component	146303400	228	Crenarchaeota	Metallosphaera sedula DSM 5348
Acd1_1258	C2	arCOG04261	S		Uncharacterized conserved protein	70606732	640	Crenarchaeota	Sulfolobus acidocaldarius DSM 639
Acd1_1259	C2	arCOG00584	H	PanB	Ketopantoate hydroxymethyltransferase	15899151	219	Crenarchaeota	Sulfolobus solfataricus P2
Acd1_1260	C2	arCOG04262	H		Phosphopantothenate synthetase	15920748	677	Crenarchaeota	Sulfolobus tokodaii str. 7
Acd1_1261	C2	arCOG04263	H		Pantoate kinase	374632006	124	Crenarchaeota	Metallosphaera yellowstonensis MK1
Acd1_1262	C2	arCOG07320	C	GlpC	Fe-S oxidoreductase	332797860	72	Crenarchaeota	Acidianus hospitalis W1
Acd1_1263	C2	arCOG05953	S		Uncharacterized conserved protein	15899147	683	Crenarchaeota	Sulfolobus solfataricus P2
Acd1_1264	C2	arCOG00536	H	MoaD	Molybdopterin converting factor, small subunit	374632045	400	Crenarchaeota	Metallosphaera yellowstonensis MK1
Acd1_1265	C2	arCOG00020	H	ThiD	Hydroxymethylpyrimidine/phosphomethylpyrimidine kinase	374632046	36.2	Crenarchaeota	Metallosphaera yellowstonensis MK1
Acd1_1266	C2	arCOG04311	R	-	Predicted hydrolase of HD superfamily	15899144	127	Crenarchaeota	Sulfolobus solfataricus P2
Acd1_1267	C2	arCOG01711	C	Ppa	Inorganic pyrophosphatase	15920741	50.1	Crenarchaeota	Sulfolobus tokodaii str. 7
Acd1_1268	C2	arCOG01817	N	VirB11	Type IV secretory pathway, VirB11 component, or related ATPase involved in archaeal flagella biosynthesis	15920739	75.5	Crenarchaeota	Sulfolobus tokodaii str. 7
Acd1_1269	C2	arCOG01808	N	TadC	Flp pilus assembly protein TadC	15920738	53.9	Crenarchaeota	Sulfolobus tokodaii str. 7
Acd1_1270	C2	arCOG00358	R	DRG	Predicted GTPase	15920737	189	Crenarchaeota	Sulfolobus tokodaii str. 7
Acd1_1271	C2	arCOG04121	L	RnhB	Ribonuclease HII	227826588	46.6	Crenarchaeota	Sulfolobus islandicus M.14.25
Acd1_1272	C2	arCOG04149	J	NMD3	NMD protein affecting ribosome stability and mRNA decay	15920735	208	Crenarchaeota	Sulfolobus tokodaii str. 7
Acd1_1273	C2	arCOG04051	S	-	Uncharacterized conserved protein	374632055	252	Crenarchaeota	Metallosphaera yellowstonensis MK1
Acd1_1274	C2	arCOG01640	J	GCD7	Translation initiation factor 2, beta subunit (eIF-2beta)/eIF-5 N-terminal domain	15920733	337	Crenarchaeota	Sulfolobus tokodaii str. 7
Acd1_1275	C2	arCOG01358	J	MiaB	2-methylthioadenine synthetase	385772200	142	Crenarchaeota	Sulfolobus islandicus HVE10/4
Acd1_1276	C2	arCOG01282	I	PaaJ	Acetyl-CoA acetyltransferase	15920731	124	Crenarchaeota	Sulfolobus tokodaii str. 7
Acd1_1277	C2	arCOG01179	J	InfA	Translation initiation factor 1 (IF-1)	20138674	903	Crenarchaeota	Sulfolobus tokodaii str. 7
Acd1_1278	C2	arCOG01180	T	RIO1	Serine/threonine protein kinase involved in cell cycle control	342306224	523	Crenarchaeota	Sulfolobus tokodaii str. 7
Acd1_1279	C2	arCOG04150	R	-	Predicted RNA-binding protein (contains KH domains)	15920728	247	Crenarchaeota	Sulfolobus tokodaii str. 7
Acd1_1280	C2	arCOG05954	S		Uncharacterized conserved protein	15920727	152	Crenarchaeota	Sulfolobus tokodaii str. 7
Acd1_1281	C2	arCOG04164	S	-	Uncharacterized conserved protein	227829220	995	Crenarchaeota	Sulfolobus islandicus L.S.2.15
Acd1_1282	C2	arCOG05955	S		Uncharacterized conserved protein	15899126	214	Crenarchaeota	Sulfolobus solfataricus P2
Acd1_1283	C2	arCOG05956	S		Uncharacterized conserved protein	70606755	134	Crenarchaeota	Sulfolobus acidocaldarius DSM 639
Acd1_1284	C2	arCOG00060	E	MetC	Cystathionine beta-lyase/cystathionine gamma-synthase	374632075	286	Crenarchaeota	Metallosphaera yellowstonensis MK1
Acd1_1285	C2	arCOG01027	E	ThrB	Homoserine kinase	15920722	642	Crenarchaeota	Sulfolobus tokodaii str. 7
Acd1_1286	C2	arCOG03838	H	PqqD	Coenzyme PQQ synthesis protein D	332797834	109	Crenarchaeota	Acidianus hospitalis W1
Acd1_1287	C2	arCOG01510	L	RFA1	Single-stranded DNA-binding replication protein A (RPA), large (70 kD) subunit or related ssDNA-binding protein	15920719	575	Crenarchaeota	Sulfolobus tokodaii str. 7
Acd1_1288	C2	arCOG00436	R	-	MoxR-like ATPase	332797832	415	Crenarchaeota	Acidianus hospitalis W1
Acd1_1289	C2	arCOG00442	R	-	Uncharacterized protein containing a von Willebrand factor type A (vWA) domain	146303450	116	Crenarchaeota	Metallosphaera sedula DSM 5348
Acd1_1290	C2	arCOG04221	G	NagD	Phosphatase of the HAD superfamily	374632080	541	Crenarchaeota	Metallosphaera yellowstonensis MK1
Acd1_1291	C2	arCOG05957	S		Uncharacterized conserved protein	70606768	430	Crenarchaeota	Sulfolobus acidocaldarius DSM 639
Acd1_1292	C2	arCOG04351	S	-	Predicted membrane protein	70606769	495	Crenarchaeota	Sulfolobus acidocaldarius DSM 639
Acd1_1293	C2	arCOG00570	C	FixC	Dehydrogenase (flavoprotein)	332797823	179	Crenarchaeota	Acidianus hospitalis W1
Acd1_1294	C2	arCOG05958	S		Uncharacterized conserved protein	146303465	562	Crenarchaeota	Metallosphaera sedula DSM 5348
Acd1_1295	C2	arCOG05959	R		Predicted ICC-like phosphoesterase	227826558	543	Crenarchaeota	Sulfolobus islandicus M.14.25
Acd1_1296	C2	arCOG04779	E	-	Asparaginase	70606241	55.1	Crenarchaeota	Sulfolobus acidocaldarius DSM 639
Acd1_1297	C2	arCOG07197	S		Uncharacterized conserved protein	342306213	224	Crenarchaeota	Sulfolobus tokodaii str. 7
Acd1_1298	C2	arCOG00570	C	FixC	Dehydrogenase (flavoprotein)	70606775	291	Crenarchaeota	Sulfolobus acidocaldarius DSM 639
Acd1_1299	C2	arCOG01580	K	Lrp	Transcriptional regulator, IclR family	15920704	359	Crenarchaeota	Sulfolobus tokodaii str. 7
Acd1_1300	C2	arCOG01589	H	RimK	Glutathione synthase/glutaminyl transferase/alpha-L-glutamate ligase	284996550	69.3	Crenarchaeota	Sulfolobus islandicus L.D.8.5
Acd1_1301	C2	arCOG01727	H	IspA	Geranylgeranyl pyrophosphate synthase	15920702	317	Crenarchaeota	Sulfolobus tokodaii str. 7
Acd1_1302	C2	arCOG05960	S		Uncharacterized conserved protein	15920701	198	Crenarchaeota	Sulfolobus tokodaii str. 7
Acd1_1303	C2	arCOG01327	F	Pnp	Purine nucleoside phosphorylase	70606780	562	Crenarchaeota	Sulfolobus acidocaldarius DSM 639
Acd1_1304	C2	arCOG00040	R	-	Predicted phosphoribosyltransferase	342306210	226	Crenarchaeota	Sulfolobus tokodaii str. 7
Acd1_1305	C2	arCOG01742	J	eRF1	Peptide chain release factor 1 (eRF1)	15920698	299	Crenarchaeota	Sulfolobus tokodaii str. 7
Acd1_1306	C2	arCOG03694	S		Uncharacterized conserved protein	146304260	443	Crenarchaeota	Metallosphaera sedula DSM 5348
Acd1_1307	C2	arCOG01316	E	PutP	Na+/proline symporter	374633466	600	Crenarchaeota	Metallosphaera yellowstonensis MK1
Acd1_1308	C2	arCOG06048	S		Uncharacterized conserved protein	227828973	295	Crenarchaeota	Sulfolobus islandicus M.14.25
tRNALeuTAG					tRNALeuTAG				
Acd1_1309	C2	arCOG04308	S	-	Uncharacterized conserved protein	15920674	136	Crenarchaeota	Sulfolobus tokodaii str. 7
Acd1_1310	C2	arCOG00578	R	-	Uncharacterized Zn-finger containing protein	15920676	453	Crenarchaeota	Sulfolobus tokodaii str. 7
Acd1_1311	C2	arCOG01891	F	Tmk	Thymidylate kinase	284175009	179	Crenarchaeota	Sulfolobus solfataricus 98/2
Acd1_1312	C2	arCOG04127	R	-	Predicted GTPase or GTP-binding protein	284175008	147	Crenarchaeota	Sulfolobus solfataricus 98/2
Acd1_1313	C2	arCOG03142	S		Uncharacterized conserved protein	70606681	342	Crenarchaeota	Sulfolobus acidocaldarius DSM 639
Acd1_1314	C2	arCOG00417	L	RecA	RecA/RadA recombinase	284997756	427	Crenarchaeota	Sulfolobus islandicus L.D.8.5
Acd1_1315	C2	arCOG04106	I	CdsA	CDP-diglyceride synthetase	70606683	435	Crenarchaeota	Sulfolobus acidocaldarius DSM 639
Acd1_1316	C2	arCOG07194	S		Uncharacterized conserved protein	15920682	173	Crenarchaeota	Sulfolobus tokodaii str. 7
Acd1_1317	C2	arCOG04059	S		Uncharacterized conserved protein	70606685	74.3	Crenarchaeota	Sulfolobus acidocaldarius DSM 639
Acd1_1318	C2	arCOG00439	L	MCM2	Predicted ATPase involved in replication control, Cdc46/Mcm family	342306205	338	Crenarchaeota	Sulfolobus tokodaii str. 7
Acd1_1319	C2	arCOG00552	L		DNA replication initiation complex subunit, GINS23 family	15920685	192	Crenarchaeota	Sulfolobus tokodaii str. 7
Acd1_1320	C2	arCOG00084	R	-	Predicted RNA-binding protein, contains THUMP domain	70606688	95.5	Crenarchaeota	Sulfolobus acidocaldarius DSM 639
Acd1_1321	C2	arCOG00467	L	CDC6	Cdc6-related protein, AAA superfamily ATPase	15920687	555	Crenarchaeota	Sulfolobus tokodaii str. 7
Acd1_1322	C2		S		Uncharacterized protein	256391161	79.7	Actinobacteria	Catenulispora acidiphila DSM 44928
Acd1_1323	C2	arCOG01530	H	MoaC	Molybdenum cofactor biosynthesis enzyme	374633942	222	Crenarchaeota	Metallosphaera yellowstonensis MK1
Acd1_1324	C2	arCOG00470	L	HolB	ATPase involved in DNA replication HolB, large subunit	42559517	126	Crenarchaeota	Sulfolobus tokodaii str. 7
Acd1_1325	C2	arCOG00469	L	HolB	ATPase involved in DNA replication HolB, small subunit	15920690	1107	Crenarchaeota	Sulfolobus tokodaii str. 7
Acd1_1326	C2	arCOG04702	R	-	Phospholipid-binding protein	227830343	114	Crenarchaeota	Sulfolobus islandicus L.S.2.15
Acd1_1327	C2	arCOG00970	O	PRE1	20S proteasome, beta subunit	146304800	213	Crenarchaeota	Metallosphaera sedula DSM 5348
Acd1_1328	C2	arCOG01717	J	GatA	Asp-tRNAAsn/Glu-tRNAGln amidotransferase A subunit or related amidase	15920693	477	Crenarchaeota	Sulfolobus tokodaii str. 7
Acd1_1329	C2	arCOG04234	S		Uncharacterized conserved protein	15920694	154	Crenarchaeota	Sulfolobus tokodaii str. 7
Acd1_1330	C2	arCOG01455	R	AdhP	Zn-dependent alcohol dehydrogenase	15920695	167	Crenarchaeota	Sulfolobus tokodaii str. 7
Acd1_1331	C2	arCOG05947	S		Uncharacterized conserved protein	70606698	319	Crenarchaeota	Sulfolobus acidocaldarius DSM 639
Acd1_1332	C2	arCOG05948	S		Uncharacterized conserved protein	70606699	39.3	Crenarchaeota	Sulfolobus acidocaldarius DSM 639
Acd1_1333	C2	arCOG07314	S		Uncharacterized conserved protein	146303572	556	Crenarchaeota	Metallosphaera sedula DSM 5348
Acd1_1334	C2	arCOG03768	S	0	Uncharacterized conserved protein	330835046	138	Crenarchaeota	Metallosphaera cuprina Ar-4
Acd1_1335	C2	arCOG09993	S		Uncharacterized conserved protein	284997832	166	Crenarchaeota	Sulfolobus islandicus L.D.8.5
Acd1_1336	C2	arCOG07716	S		SWIM Zn-finger	284997802	144	Crenarchaeota	Sulfolobus islandicus L.D.8.5
Acd1_1337	C2	arCOG10132	S		Uncharacterized conserved protein	227830582	100	Crenarchaeota	Sulfolobus islandicus L.S.2.15
Acd1_1338	C2	arCOG06831	R	Doc	Prophage maintenance system killer protein	342306439	257	Crenarchaeota	Sulfolobus tokodaii str. 7
Acd1_1339	C2	arCOG00770	K	DinG	Rad3-related DNA helicase	284997808	143	Crenarchaeota	Sulfolobus islandicus L.D.8.5
Acd1_1340	C2		S		Uncharacterized protein	284997808	182	Crenarchaeota	Sulfolobus islandicus L.D.8.5
Acd1_1341	C2	arCOG03169	R	-	Predicted ATPase (AAA+ superfamily)	229580434	209	Crenarchaeota	Sulfolobus islandicus Y.G.57.14
Acd1_1342	C2		S		Uncharacterized protein	379004852	731	Crenarchaeota	Pyrobaculum oguniense TE7

Acd1_1343	C2	arCOG00138	G	ProP	Permease of the major facilitator superfamily	15899717	70.5	Crenarchaeota	Sulfolobus solfataricus P2
Acd1_1344	C2	arCOG05320	R		Predicted membrane-bound metal-dependent hydrolase	229584225	471	Crenarchaeota	Sulfolobus islandicus M.16.27
Acd1_1345	C2	arCOG02683	G	ProP	Permease of the major facilitator superfamily	15920919	35.4	Crenarchaeota	Sulfolobus tokodaii str. 7
Acd1_1346	C2	arCOG02115	S	-	Uncharacterized conserved protein	15922817	555	Crenarchaeota	Sulfolobus tokodaii str. 7
Acd1_1347	C2	arCOG01065	R	HcaD	NAD(FAD)-dependent dehydrogenase	15922815	313	Crenarchaeota	Sulfolobus tokodaii str. 7
Acd1_1348	C2	arCOG03465	E	PotE	Amino acid transporter	15922813	600	Crenarchaeota	Sulfolobus tokodaii str. 7
Acd1_1349	C2	arCOG01302	S	-	Uncharacterized conserved protein	227828856	707	Crenarchaeota	Sulfolobus islandicus M.14.25
Acd1_1350	C2	arCOG01167	C	CoxL	Aerobic-type carbon monoxide dehydrogenase, large subunit CoxL/CutL homolog	15922814	372	Crenarchaeota	Sulfolobus tokodaii str. 7
Acd1_1351	C2	arCOG01482	H	NadC	Nicotinate-nucleotide pyrophosphorylase	15921461	196	Crenarchaeota	Sulfolobus tokodaii str. 7
Acd1_1352	C2	arCOG00572	H	NadB	Aspartate oxidase	332797910	114	Crenarchaeota	Acidianus hospitalis W1
Acd1_1353	C2	arCOG04459	H	NadA	Quinolinate synthase	363548443	115	Crenarchaeota	Sulfolobus tokodaii str. 7
Acd1_1354	C2	arCOG03671	E		Thermopsin-like protease	15922807	221	Crenarchaeota	Sulfolobus tokodaii str. 7
Acd1_1355	C2	arCOG03861	S		Uncharacterized conserved protein	146303446	177	Crenarchaeota	Metallosphaera sedula DSM 5348
Acd1_1356	C2	arCOG06008	R		Predicted metal permease	352682566	559	Crenarchaeota	Thermoproteus tenax Kra 1
Acd1_1357	C2		S		Uncharacterized protein				
Acd1_1358	C2	arCOG08324	S		Uncharacterized conserved protein	374634157	420	Crenarchaeota	Metallosphaera yellowstonensis MK1
Acd1_1359	C2	arCOG07731	S		Uncharacterized conserved protein	15922804	272	Crenarchaeota	Sulfolobus tokodaii str. 7
Acd1_1360	C2	arCOG00196	V	CcmA	ABC-type multidrug transport system, ATPase component	332797692	265	Crenarchaeota	Acidianus hospitalis W1
Acd1_1361	C2	arCOG01491	C	BisC	Anaerobic dehydrogenase	15920859	196	Crenarchaeota	Sulfolobus tokodaii str. 7
Acd1_1362	C2	arCOG01929	O	XdhC	Xanthine and CO dehydrogenase maturation factor, XdhC/CoxF family	229581025	185	Crenarchaeota	Sulfolobus islandicus Y.N.15.51
Acd1_1363	C2	arCOG00972	H	NadR	Nicotinamide mononucleotide adenylyltransferase	70606139	412	Crenarchaeota	Sulfolobus acidocaldarius DSM 639
Acd1_1364	C2	arCOG08315	S		Uncharacterized conserved protein	227828967	603	Crenarchaeota	Sulfolobus islandicus M.14.25
Acd1_1365	C2	arCOG04053	E	Ggt	Gamma-glutamyltransferase	15922686	154	Crenarchaeota	Sulfolobus tokodaii str. 7
Acd1_1366	C2	arCOG03652	E	PotE	Amino acid transporter	15922692	532	Crenarchaeota	Sulfolobus tokodaii str. 7
Acd1_1367	C2	arCOG05926	S		Uncharacterized conserved protein	15899890	42.7	Crenarchaeota	Sulfolobus solfataricus P2
Acd1_1368	C2	arCOG01951	R	-	Predicted P-loop ATPase fused to an acetyltransferase	15922689	132	Crenarchaeota	Sulfolobus tokodaii str. 7
Acd1_1369	C2	arCOG01084	R	MazG	Predicted pyrophosphatase	284175864	635	Crenarchaeota	Sulfolobus solfataricus 98/2
Acd1_1370	C2	arCOG05925	S		Uncharacterized conserved protein	15922687	912	Crenarchaeota	Sulfolobus tokodaii str. 7
Acd1_1371	C2		S		Uncharacterized protein				
Acd1_1372	C2	arCOG03659	G	-	Predicted N-acetylglucosamine kinase	70606260	365	Crenarchaeota	Sulfolobus acidocaldarius DSM 639
Acd1_1373	C2	arCOG00664	M	RfbA	dTDP-glucose pyrophosphorylase	15922683	320	Crenarchaeota	Sulfolobus tokodaii str. 7
Acd1_1374	C2	arCOG00561	O	-	Predicted membrane-bound dolichyl-phosphate-mannose-protein mannosyltransferase	15922682	100	Crenarchaeota	Sulfolobus tokodaii str. 7
Acd1_1375	C2	arCOG04044	E		2-amino-3,7-dideoxy-D-threo-hept-6-ulosonic acid synthase, DhnA-aldolase family	15922681	132	Crenarchaeota	Sulfolobus tokodaii str. 7
Acd1_1376	C2	arCOG03654	E	PotE	Amino acid transporter	327310005	998	Crenarchaeota	Thermoproteus uzoniensis 768-20
Acd1_1377	C2	arCOG01068	C	Lpd	Pyruvate/2-oxoglutarate dehydrogenase complex, dihydrolipoamide dehydrogenase (E3) component or related enzyme	15922178	30.8	Crenarchaeota	Sulfolobus tokodaii str. 7
Acd1_1378	C2		S		Uncharacterized protein	313206167	57.8	Bacteroidetes/Chlorobi group	Riemerella anatipestifer ATCC 11845 = DSM 15868
Acd1_1379	C2	arCOG03851	S		Uncharacterized conserved protein	146304946	512	Crenarchaeota	Metallosphaera sedula DSM 5348
Acd1_1380	C2	arCOG00975	J	Sun	tRNA or rRNA cytosine-C5-methylase	15922756	432	Crenarchaeota	Sulfolobus tokodaii str. 7
Acd1_1381	C2	arCOG02881	P	ECM27	Ca2+/Na+ antiporter	229582403	617	Crenarchaeota	Sulfolobus islandicus Y.N.15.51
Acd1_1382	C2		S		Uncharacterized protein	15922523	327	Crenarchaeota	Sulfolobus tokodaii str. 7
Acd1_1383	C2		S		Uncharacterized protein				
Acd1_1384	C2	arCOG02441	R	NosY	ABC-type transport system involved in multi-copper enzyme maturation, permease component	70607513	258	Crenarchaeota	Sulfolobus acidocaldarius DSM 639
Acd1_1385	C2	arCOG00194	V	CcmA	ABC-type multidrug transport system, ATPase component	332797654	541	Crenarchaeota	Acidianus hospitalis W1
Acd1_1386	C2	arCOG01167	C	CoxL	Aerobic-type carbon monoxide dehydrogenase, large subunit CoxL/CutL homolog	15920967	262	Crenarchaeota	Sulfolobus tokodaii str. 7
Acd1_1387	C2	arCOG06001	R		Predicted periplasmic protein	15899878	29.6	Crenarchaeota	Sulfolobus solfataricus P2
Acd1_1388	C2	arCOG01606	C	PorA	Pyruvate:ferredoxin oxidoreductase or related 2-oxoacid:ferredoxin oxidoreductase, alpha subunit and gamma	15922767	228	Crenarchaeota	Sulfolobus tokodaii str. 7
Acd1_1389	C2	arCOG01599	C	PorB	Pyruvate:ferredoxin oxidoreductase or related 2-oxoacid:ferredoxin oxidoreductase, beta subunit	15922765	138	Crenarchaeota	Sulfolobus tokodaii str. 7
Acd1_1390	C2	arCOG03879	K	-	Predicted transcriptional regulator	70608017	233	Crenarchaeota	Sulfolobus acidocaldarius DSM 639
Acd1_1391	C2		S		Uncharacterized protein				
Acd1_1392	C2	arCOG05913	S		Uncharacterized conserved protein	332795709	268	Crenarchaeota	Acidianus hospitalis W1
Acd1_1393	C2	arCOG05710	S		Uncharacterized conserved protein	15898755	90.1	Crenarchaeota	Sulfolobus solfataricus P2
Acd1_1394	C2	arCOG01918	O	HflC	Membrane protease subunit, stomatin/prohibitin homolog	70607478	164	Crenarchaeota	Sulfolobus acidocaldarius DSM 639
Acd1_1395	C2	arCOG06007	S		Uncharacterized conserved protein	70607477	102	Crenarchaeota	Sulfolobus acidocaldarius DSM 639
Acd1_1396	C2		S		Uncharacterized protein				
Acd1_1397	C2	arCOG01187	O	HypF	Hydrogenase maturation factor	385772914	54.7	Crenarchaeota	Sulfolobus islandicus HVE10/4
Acd1_1398	C2	arCOG03778	S		Uncharacterized conserved protein	15922754	87	Crenarchaeota	Sulfolobus tokodaii str. 7
Acd1_1399	C2	arCOG06035	S		Uncharacterized conserved protein	332797308	533	Crenarchaeota	Acidianus hospitalis W1
Acd1_1400	C2		S		Uncharacterized protein	126459618	210	Crenarchaeota	Pyrobaculum calidifontis JCM 11548
Acd1_1401	C2	arCOG04480	K	AbrB	Transcriptional regulator AbrB	227831197	94	Crenarchaeota	Sulfolobus islandicus L.S.2.15
Acd1_1402	C2	arCOG00715	V	-	PIN domain containing protein	227831196	956	Crenarchaeota	Sulfolobus islandicus L.S.2.15
Acd1_1403	C2	arCOG06023	P	HoxN	High-affinity nickel permease	327312060	67	Crenarchaeota	Thermoproteus uzoniensis 768-20
Acd1_1404	C2	arCOG10002	S		Uncharacterized conserved protein	332797121	144	Crenarchaeota	Acidianus hospitalis W1
Acd1_1405	C2	arCOG01663	K	RelE	Cytotoxic translational repressor of toxin-antitoxin stability system	284997851	232	Crenarchaeota	Sulfolobus islandicus L.D.8.5
Acd1_1406	C2	arCOG07934	S		Uncharacterized protein	15921577	249	Crenarchaeota	Sulfolobus tokodaii str. 7
Acd1_1407	C2		S		Uncharacterized protein	227826447	1224	Crenarchaeota	Sulfolobus islandicus M.14.25
Acd1_1408	C2	arCOG00143	G	ProP	Permease of the major facilitator superfamily	15922738	689	Crenarchaeota	Sulfolobus tokodaii str. 7
Acd1_1409	C2	arCOG06023	P	HoxN	High-affinity nickel permease	327312060	70.9	Crenarchaeota	Thermoproteus uzoniensis 768-20
Acd1_1410	C3	arCOG01446	K	ArsR	Transcriptional regulator containing HTH domain, ArsR family	385772763	296	Crenarchaeota	Sulfolobus islandicus HVE10/4
Acd1_1411	C3	arCOG00823	K	AbrB	Transcriptional regulator AbrB	332796045	109	Crenarchaeota	Acidianus hospitalis W1
Acd1_1412	C3	arCOG03672	E		Thermopsin-like protease	332795892	586	Crenarchaeota	Acidianus hospitalis W1
Acd1_1413	C3	arCOG01204	R	-	Minimal nucleotidyltransferase	227826731	793	Crenarchaeota	Sulfolobus islandicus M.14.25
Acd1_1414	C3	arCOG01573	M	MscS	Small-conductance mechanosensitive channel	374632213	221	Crenarchaeota	Metallosphaera yellowstonensis MK1
Acd1_1415	C3	arCOG03672	E		Thermopsin-like protease	385772646	275	Crenarchaeota	Sulfolobus islandicus HVE10/4
Acd1_1416	C3	arCOG03712	R		HEPN domain	332797704	221	Crenarchaeota	Acidianus hospitalis W1
Acd1_1417	C3	arCOG03167	R	-	Predicted ATPase (AAA+ superfamily)	332796111	278	Crenarchaeota	Acidianus hospitalis W1
Acd1_1418	C3	arCOG06034	S		Uncharacterized conserved protein	15922700	2182	Crenarchaeota	Sulfolobus tokodaii str. 7
Acd1_1419	C3	arCOG00343	C	GlpC	Fe-S oxidoreductase	15899369	51.6	Crenarchaeota	Sulfolobus solfataricus P2
Acd1_1420	C3	arCOG01097	C	-	Rubrerythrin	15899368	638	Crenarchaeota	Sulfolobus solfataricus P2
Acd1_1421	C3	arCOG05713	VL		McrBC 5-methylcytosine restriction system component	229579809	141	Crenarchaeota	Sulfolobus islandicus Y.G.57.14
Acd1_1422	C3	arCOG03779	V		GTPase subunit of restriction endonuclease	229579810	335	Crenarchaeota	Sulfolobus islandicus Y.G.57.14
tRNA Pseudo CTC					tRNA Pseudo CTC				
Acd1_1423	C3	arCOG00579	K	RPB9	DNA-directed RNA polymerase, subunit M/Transcription elongation factor TFIIS	332795967	374	Crenarchaeota	Acidianus hospitalis W1
Acd1_1424	C3	arCOG01357	C	-	Radical SAM superfamily enzyme	146303676	31.2	Crenarchaeota	Metallosphaera sedula DSM 5348
Acd1_1425	C3	arCOG04641	S	-	Predicted membrane protein	332797719	595	Crenarchaeota	Acidianus hospitalis W1
Acd1_1426	C3	arCOG01367	M	RfbD	dTDP-4-dehydrorhamnose reductase	332795820	196	Crenarchaeota	Acidianus hospitalis W1
Acd1_1427	C3	arCOG08305	S		Uncharacterized conserved protein	385776201	43.1	Crenarchaeota	Sulfolobus islandicus REY15A
Acd1_1428	C3		S		Uncharacterized protein	374631976	123	Crenarchaeota	Metallosphaera yellowstonensis MK1
Acd1_1429	C3		S		Uncharacterized protein	315231869	42.4	Euryarchaeota	Thermococcus barophilus MP
Acd1_1430	C3	arCOG02782	V	-	Endonuclease, HJR/Mrr/RecB family	145591469	315	Crenarchaeota	Pyrobaculum arsenaticum DSM 13514
Acd1_1431	C3	arCOG01491	C	BisC	Anaerobic dehydrogenase	332797254	371	Crenarchaeota	Acidianus hospitalis W1
Acd1_1432	C3	arCOG06016	C	CbsB	Cytochrome b558/566, subunit B	374632362	76.6	Crenarchaeota	Metallosphaera yellowstonensis MK1
Acd1_1433	C3	arCOG06015	C	CbsA	Cytochrome b558/566, subunit A	15921967	121	Crenarchaeota	Sulfolobus tokodaii str. 7
Acd1_1434	C3		S		Uncharacterized protein	159042150	99.8	Crenarchaeota	Caldivirga maquilingensis IC-167
Acd1_1435	C3	arCOG03721	R		HEPN domain	332795970	260	Crenarchaeota	Acidianus hospitalis W1
Acd1_1436	C3	arCOG03721	R		HEPN domain	332795969	112	Crenarchaeota	Acidianus hospitalis W1
Acd1_1437	C3	arCOG01191	V	-	HEPN domain containing protein	332797208	625	Crenarchaeota	Acidianus hospitalis W1
Acd1_1438	C3	arCOG07292	R	-	Minimal nucleotidyltransferase	332797209	219	Crenarchaeota	Acidianus hospitalis W1
Acd1_1439	C3	arCOG03169	R	-	Predicted ATPase (AAA+ superfamily)	302348489	460	Crenarchaeota	Acidilobus saccharovorans 345-15
Acd1_1440	C3	arCOG07305	S		Uncharacterized conserved protein	332797237	187	Crenarchaeota	Acidianus hospitalis W1
Acd1_1441	C3	arCOG06017	S		Uncharacterized conserved protein	15921966	378	Crenarchaeota	Sulfolobus tokodaii str. 7
Acd1_1442	C3	arCOG01720	C	SoxL	Rieske Fe-S protein	374632363	857	Crenarchaeota	Metallosphaera yellowstonensis MK1
Acd1_1443	C3	arCOG01721	C	SoxN	Cytochrome b subunit of the bc complex	146303284	128	Crenarchaeota	Metallosphaera sedula DSM 5348
Acd1_1444	C3	arCOG05403	R	OdsN	Predicted heme-degrading monooxygenase	146303283	260	Crenarchaeota	Metallosphaera sedula DSM 5348
Acd1_1445	C3	arCOG03164	L	-	Predicted site-specific integrase-resolvase	229578503	89.7	Crenarchaeota	Sulfolobus islandicus Y.G.57.14
Acd1_1446	C3		S		Uncharacterized protein				
Acd1_1447	C3	arCOG01123	J	-	Translation initiation factor eIF-2B alpha subunit	15921156	66.6	Crenarchaeota	Sulfolobus tokodaii str. 7
Acd1_1448	C3	arCOG05396	S	-	Predicted membrane protein	15896986	763	Crenarchaeota	Sulfolobus solfataricus P2
Acd1_1449	C3	arCOG02050	R	-	Predicted permease	227828357	246	Crenarchaeota	Sulfolobus islandicus M.14.25
Acd1_1450	C3	arCOG04291	S	-	Uncharacterized conserved protein	332796127	515	Crenarchaeota	Acidianus hospitalis W1
Acd1_1451	C3	arCOG01420	G	GlgA	Glycogen synthase	15921053	901	Crenarchaeota	Sulfolobus tokodaii str. 7
Acd1_1452	C3	arCOG05922	S		Uncharacterized conserved protein	15920857	108	Crenarchaeota	Sulfolobus tokodaii str. 7
Acd1_1453	C3		S		Uncharacterized protein				
Acd1_1454	C3	arCOG02702	Q		SAM-dependent methyltransferase	10639797	312	Euryarchaeota	Thermoplasma acidophilum
Acd1_1455	C3		S		Uncharacterized protein	227828621	172	Crenarchaeota	Sulfolobus islandicus M.14.25
Acd1_1456	C3	arCOG03682	R	SPS1	Membrane associated serine/threonine protein kinase	15921012	210	Crenarchaeota	Sulfolobus tokodaii str. 7
Acd1_1457	C3	arCOG00967	J	-	tRNA m(1)G methyltransferase	15921088	796	Crenarchaeota	Sulfolobus tokodaii str. 7
Acd1_1458	C3	arCOG00130	G	ProP	Permease of the major facilitator superfamily	229582368	449	Crenarchaeota	Sulfolobus islandicus Y.N.15.51
Acd1_1459	C3	arCOG00223	R	ModE	N-terminal domain of molybdenum-binding protein	70606962	178	Crenarchaeota	Sulfolobus acidocaldarius DSM 639
Acd1_1460	C3	arCOG03038	R	NrfG	TPR repeats containing protein	15921045	418	Crenarchaeota	Sulfolobus tokodaii str. 7
Acd1_1461	C3	arCOG08332	S		Uncharacterized conserved protein	332796597	39.3	Crenarchaeota	Acidianus hospitalis W1
Acd1_1462	C3	arCOG01597	F	PurK	Phosphoribosylaminoimidazole carboxylase (NCAIR synthetase)	15921042	397	Crenarchaeota	Sulfolobus tokodaii str. 7
Acd1_1463	C3	arCOG02969	E	PepN	Aminopeptidase N	15920843	69.3	Crenarchaeota	Sulfolobus tokodaii str. 7
Acd1_1464	C3	arCOG00809	J	LeuS	Leucyl-tRNA synthetase	70606212	1086	Crenarchaeota	Sulfolobus acidocaldarius DSM 639
Acd1_1465	C3	arCOG00945	R	AslB	Radical SAM superfamily enzyme	302349206	359	Crenarchaeota	Acidilobus saccharovorans 345-15
Acd1_1466	C3	arCOG00475	E	MetF	5,10-methylenetetrahydrofolate reductase	15920852	403	Crenarchaeota	Sulfolobus tokodaii str. 7
Acd1_1467	C3	arCOG00042	D	MesJ	tRNA(Ile)-lysidine synthase MesJ	15920850	62	Crenarchaeota	Sulfolobus tokodaii str. 7
Acd1_1468	C3	arCOG07934	S		Uncharacterized protein	284998406	422	Crenarchaeota	Sulfolobus islandicus L.D.8.5
Acd1_1469	C3	arCOG01663	K	RelE	Cytotoxic translational repressor of toxin-antitoxin stability system	229581518	68.2	Crenarchaeota	Sulfolobus islandicus Y.N.15.51
Acd1_1470	C3	arCOG05924	S		Uncharacterized conserved protein	15920849	97.4	Crenarchaeota	Sulfolobus tokodaii str. 7

Acd1_1471	C3	arCOG01110	E	ArgE	Acetylornithine deacetylase/Succinyl-diaminopimelate desuccinylase or related deacylase	15920847	196	Crenarchaeota	Sulfolobus tokodaii str. 7
Acd1_1472	C3	arCOG03861	S		Uncharacterized conserved protein	146303446	728	Crenarchaeota	Metallosphaera sedula DSM 5348
Acd1_1473	C3	arCOG00082	E	-	Serine-pyruvate aminotransferase/archaeal aspartate aminotransferase	15920822	769	Crenarchaeota	Sulfolobus tokodaii str. 7
Acd1_1474	C3		S		Uncharacterized protein				
Acd1_1475	C3	arCOG00662	R	-	Biotin synthase-related enzyme	146304273	214	Crenarchaeota	Metallosphaera sedula DSM 5348
Acd1_1476	C3	arCOG05825	R	-	Radical SAM superfamily enzyme	146304272	325	Crenarchaeota	Metallosphaera sedula DSM 5348
Acd1_1477	C3	arCOG01939	H	LplA	Lipoate-protein ligase A	374633478	353	Crenarchaeota	Metallosphaera yellowstonensis MK1
Acd1_1478	C3	arCOG03837	H		Lipoate-protein ligase A associated domain	374633477	340	Crenarchaeota	Metallosphaera yellowstonensis MK1
Acd1_1479	C3	arCOG02727	R	-	Predicted RNA-binding protein containing PUA-domain	47117615	448	Crenarchaeota	Sulfolobus solfataricus P2
Acd1_1480	C3		S		Uncharacterized protein	70606232	53.9	Crenarchaeota	Sulfolobus acidocaldarius DSM 639
Acd1_1481	C3	arCOG02159	S	-	Predicted membrane protein	332795733	322	Crenarchaeota	Acidianus hospitalis W1
Acd1_1482	C3	arCOG01871	H	-	GTP:adenosylcobinamide-phosphate guanylyltransferase	70606230	191	Crenarchaeota	Sulfolobus acidocaldarius DSM 639
Acd1_1483	C3	arCOG00194	V	CcmA	ABC-type multidrug transport system, ATPase component	15920828	286	Crenarchaeota	Sulfolobus tokodaii str. 7
Acd1_1484	C3	arCOG01463	V	-	ABC-type multidrug transport system, permease component	70606227	340	Crenarchaeota	Sulfolobus acidocaldarius DSM 639
Acd1_1485	C3	arCOG00242	I	CaiD	Enoyl-CoA hydratase/carnithine racemase	15920830	317	Crenarchaeota	Sulfolobus tokodaii str. 7
Acd1_1486	C3	arCOG00247	I	MmsB	3-hydroxyisobutyrate dehydrogenase or related beta-hydroxyacid dehydrogenase	15920839	407	Crenarchaeota	Sulfolobus tokodaii str. 7
Acd1_1487	C3	arCOG01320	H	RibB	3,4-dihydroxy-2-butanone 4-phosphate synthase	70606612	358	Crenarchaeota	Sulfolobus acidocaldarius DSM 639
Acd1_1488	C3	arCOG01322	H	-	Archaeal riboflavin synthase	70606611	200	Crenarchaeota	Sulfolobus acidocaldarius DSM 639
Acd1_1489	C3	arCOG01323	H	RibH	Riboflavin synthase beta-chain	70606610	101	Crenarchaeota	Sulfolobus acidocaldarius DSM 639
Acd1_1490	C3	arCOG04202	F	-	Archaeal GTP cyclohydrolase III	70606609	130	Crenarchaeota	Sulfolobus acidocaldarius DSM 639
Acd1_1491	C3	arCOG00692	F	SsnA	Cytosine deaminase or related metal-dependent hydrolase	70606608	228	Crenarchaeota	Sulfolobus acidocaldarius DSM 639
Acd1_1492	C3	arCOG04536	H	-	Fe2+ dependent formamide hydrolase, cofactor Fo biosynthesis enzyme	70605987	306	Crenarchaeota	Sulfolobus acidocaldarius DSM 639
Acd1_1493	C3	arCOG01484	H	RibD	Pyrimidine reductase, riboflavin biosynthesis	15898882	548	Crenarchaeota	Sulfolobus solfataricus P2
Acd1_1494	C3		S		Uncharacterized protein	374631083	211	Crenarchaeota	Metallosphaera yellowstonensis MK1
Acd1_1495	C3	arCOG03233	R		Predicted ATPase	374631084	379	Crenarchaeota	Metallosphaera yellowstonensis MK1
Acd1_1496	C3	arCOG05495	S	-	Uncharacterized conserved protein, contains N-terminal coiled-coil domain	15920840	112	Crenarchaeota	Sulfolobus tokodaii str. 7
Acd1_1497	C3		S		Uncharacterized protein	229579620	219	Crenarchaeota	Sulfolobus islandicus Y.G.57.14
Acd1_1501	C3	arCOG00406	J	AsnS	Aspartyl/asparaginyl-tRNA synthetase	118431776	268	Crenarchaeota	Aeropyrum pernix K1
Acd1_1502	C3	arCOG00824	K	AbrB	Transcriptional regulator AbrB	227829537	307	Crenarchaeota	Sulfolobus islandicus L.S.2.15
Acd1_1503	C3	arCOG02221	V	-	PIN domain containing protein	332795954	162	Crenarchaeota	Acidianus hospitalis W1
Acd1_1504	C3	arCOG00934	C	PflX	Radical SAM superfamily enzyme	315426687	311	Thaumarchaeota	Candidatus Caldiarchaeum subterraneum
Acd1_1505	C3		S		Uncharacterized protein				
Acd1_1506	C3	arCOG04294	L	RecA	RecA/RadA recombinase	146304210	170	Crenarchaeota	Metallosphaera sedula DSM 5348
Acd1_1507	C3	arCOG00329	L	PolB	DNA polymerase elongation subunit (family B) , inactivated	332796088	561	Crenarchaeota	Acidianus hospitalis W1
Acd1_1508	C3		S		Uncharacterized protein	374633403	85.9	Crenarchaeota	Metallosphaera yellowstonensis MK1
Acd1_1509	C3	arCOG04507	Q	-	Methane/Phenol/Toluene Hydroxylase component	374632693	63.5	Crenarchaeota	Metallosphaera yellowstonensis MK1
Acd1_1510	C3	arCOG02763	P		Heavy-metal-associated domain (HMA)	374631718	414	Crenarchaeota	Metallosphaera yellowstonensis MK1
Acd1_1511	C3		S		Uncharacterized protein				
Acd1_1512	C3	arCOG01953	P	KefB	Kef-type K+ transport system, membrane component	385773051	291	Crenarchaeota	Sulfolobus islandicus HVE10/4
Acd1_1513	C3	arCOG03169	R	-	Predicted ATPase (AAA+ superfamily)	332796072	416	Crenarchaeota	Acidianus hospitalis W1
Acd1_1514	C3		S		Uncharacterized protein	56407101	417	Crenarchaeota	Sulfolobus sp. Y00 58-82
Acd1_1515	C3		S		Uncharacterized protein	15898779	132	Crenarchaeota	Sulfolobus solfataricus P2
Acd1_1516	C3	arCOG10265	S		Uncharacterized conserved protein	15898780	177	Crenarchaeota	Sulfolobus solfataricus P2
Acd1_1517	C3		S		Uncharacterized protein	374632512	100	Crenarchaeota	Metallosphaera yellowstonensis MK1
Acd1_1518	C3		S		Uncharacterized protein	229584977	484	Crenarchaeota	Sulfolobus islandicus M.16.27
Acd1_1519	C3		S		Uncharacterized protein	229584976	248	Crenarchaeota	Sulfolobus islandicus M.16.27
Acd1_1520	C3		S		Uncharacterized protein				
Acd1_1521	C3	arCOG02134	L	-	Transposase	15922323	71.2	Crenarchaeota	Sulfolobus tokodaii str. 7
Acd1_1522	C3	arCOG03721	R		HEPN domain	332797290	363	Crenarchaeota	Acidianus hospitalis W1
Acd1_1523	C3	arCOG03708	R		HEPN domain	15922333	71.6	Crenarchaeota	Sulfolobus tokodaii str. 7
Acd1_1524	C3	arCOG04502	V	-	PIN domain containing protein	227829529	246	Crenarchaeota	Sulfolobus islandicus L.S.2.15
Acd1_1525	C3	arCOG07990	S		Uncharacterized protein	227829528	231	Crenarchaeota	Sulfolobus islandicus L.S.2.15
Acd1_1526	C3	arCOG03167	R	-	Predicted ATPase (AAA+ superfamily)	15922904	197	Crenarchaeota	Sulfolobus tokodaii str. 7
Acd1_1527	C3	arCOG00916	E	GabT	4-aminobutyrate aminotransferase or related aminotransferase	332796117	550	Crenarchaeota	Acidianus hospitalis W1
Acd1_1528	C3	arCOG00916	E	GabT	4-aminobutyrate aminotransferase or related aminotransferase	332796117	476	Crenarchaeota	Acidianus hospitalis W1
Acd1_1529	C3	arCOG07589	V		PIN domain containing protein	332797287	214	Crenarchaeota	Acidianus hospitalis W1
Acd1_1530	C3	arCOG01200	V	-	Minimal nucleotidyltransferase	284997159	176	Crenarchaeota	Sulfolobus islandicus L.D.8.5
Acd1_1531	C3	arCOG01191	V	-	HEPN domain containing protein	15920977	184	Crenarchaeota	Sulfolobus tokodaii str. 7
Acd1_1532	C3	arCOG03844	R		TlpA/TrxA-like family protein	15922004	215	Crenarchaeota	Sulfolobus tokodaii str. 7
Acd1_1533	C3	arCOG07852	R		Predicted regulatory ATPase	15898778	315	Crenarchaeota	Sulfolobus solfataricus P2
Acd1_1534	C3		S		Uncharacterized protein	296242623	236	Crenarchaeota	Thermosphaera aggregans DSM 11486
Acd1_1535	C3		S		Uncharacterized protein	284997840	357	Crenarchaeota	Sulfolobus islandicus L.D.8.5
Acd1_1536	C3		S		Uncharacterized protein	284175793	132	Crenarchaeota	Sulfolobus solfataricus 98/2
Acd1_1537	C3		S		Uncharacterized protein	284175794	154	Crenarchaeota	Sulfolobus solfataricus 98/2
Acd1_1538	C3		S		Uncharacterized protein				
Acd1_1539	C3	arCOG08560	S		Uncharacterized conserved protein	55701195	0	Crenarchaeota	Sulfolobus islandicus
Acd1_1540	C3	arCOG03712	R		HEPN domain	229582786	693	Crenarchaeota	Sulfolobus islandicus Y.N.15.51
Acd1_1541	C3	arCOG07296	S		Uncharacterized conserved protein	332797718	423	Crenarchaeota	Acidianus hospitalis W1
Acd1_1542	C3	arCOG01646	E	DAP2	Dipeptidyl aminopeptidase/acylaminoacyl-peptidase	15922055	109	Crenarchaeota	Sulfolobus tokodaii str. 7
Acd1_1543	C3	arCOG07852	R		Predicted regulatory ATPase	284997824	263	Crenarchaeota	Sulfolobus islandicus L.D.8.5
Acd1_1544	C3	arCOG10353	S		Uncharacterized conserved protein	15898779	259	Crenarchaeota	Sulfolobus solfataricus P2
Acd1_1545	C4		S		Uncharacterized protein	385772766	211	Crenarchaeota	Sulfolobus islandicus HVE10/4
Acd1_1546	C4		S		Uncharacterized protein				
Acd1_1547	C4		S		Uncharacterized protein	157929294	685	no RNA stage	Acidianus filamentous virus 2
Acd1_1548	C4	arCOG07237	V		Uncharacterized protein, contains PIN domain	332797129	593	Crenarchaeota	Acidianus hospitalis W1
Acd1_1549	C4	arCOG03712	R		HEPN domain	229582786	815	Crenarchaeota	Sulfolobus islandicus Y.N.15.51
Acd1_1550	C4	arCOG07296	S		Uncharacterized conserved protein	332797718	91.3	Crenarchaeota	Acidianus hospitalis W1
Acd1_1551	C4		S		Uncharacterized protein	374634173	74.7	Crenarchaeota	Metallosphaera yellowstonensis MK1
Acd1_1552	C4	arCOG07184	S		Uncharacterized conserved protein	284998099	711	Crenarchaeota	Sulfolobus islandicus L.D.8.5
Acd1_1553	C4	arCOG00724	K		Predicted transcriptional regulator, PadR family	227830911	299	Crenarchaeota	Sulfolobus islandicus L.S.2.15
Acd1_1554	C4	arCOG07718	S		Uncharacterized conserved protein	227830912	318	Crenarchaeota	Sulfolobus islandicus L.S.2.15
Acd1_1555	C4	arCOG08216	V		Antitoxin MazE superfamily protein	332797286	33.1	Crenarchaeota	Acidianus hospitalis W1
Acd1_1556	C4	arCOG07589	V		PIN domain containing protein	332797287	59.3	Crenarchaeota	Acidianus hospitalis W1
Acd1_1557	C4	arCOG03239	R	-	ATPase, predicted component of phage defense system	229582903	299	Crenarchaeota	Sulfolobus islandicus Y.N.15.51
Acd1_1558	C4	arCOG03239	R	-	ATPase, predicted component of phage defense system	229582903	137	Crenarchaeota	Sulfolobus islandicus Y.N.15.51
Acd1_1559	C4	arCOG02681	V	-	Predicted antitoxin, copG family	229578552	372	Crenarchaeota	Sulfolobus islandicus Y.G.57.14
Acd1_1560	C4	arCOG02219	V	VapC	PIN domain containing protein	15898771	659	Crenarchaeota	Sulfolobus solfataricus P2
Acd1_1561	C4	arCOG01192	V	-	HEPN domain containing protein	284997157	218	Crenarchaeota	Sulfolobus islandicus L.D.8.5
Acd1_1562	C4	arCOG01203	R	-	Minimal nucleotidyltransferase	227829753	263	Crenarchaeota	Sulfolobus islandicus L.S.2.15
Acd1_1563	C4	arCOG03721	R		HEPN domain	284997160	243	Crenarchaeota	Sulfolobus islandicus L.D.8.5
Acd1_1564	C4		S		Uncharacterized protein	15898786	334	Crenarchaeota	Sulfolobus solfataricus P2
Acd1_1565	C4	arCOG04429	C	HyaD	Ni,Fe-hydrogenase maturation factor	22204184	762	Crenarchaeota	Acidianus ambivalens
Acd1_1566	C4	arCOG06056	C	HyaD	Ni,Fe-hydrogenase maturation factor	22204183	206	Crenarchaeota	Acidianus ambivalens
Acd1_1567	C4		S		Membrane-bound NiFe hydrogenase subunit	22204182	1264	Crenarchaeota	Acidianus ambivalens
Acd1_1568	C4	arCOG00636	O	HypE	Hydrogenase maturation factor	22204181	871	Crenarchaeota	Acidianus ambivalens
Acd1_1569	C4	arCOG04427	O	HypC	Hydrogenase maturation factor	22204180	108	Crenarchaeota	Acidianus ambivalens
Acd1_1570	C4	arCOG04428	O	HypD	Hydrogenase maturation factor	22204179	145	Crenarchaeota	Acidianus ambivalens
Acd1_1571	C4	arCOG06057	C		Membrane-bound NiFe hydrogenase subunit	22204178	838	Crenarchaeota	Acidianus ambivalens
Acd1_1572	C4	arCOG02852	P	{NirD}	Ferredoxin subunit of nitrite reductase or ring-hydroxylating dioxygenase	22204177	244	Crenarchaeota	Acidianus ambivalens
Acd1_1573	C4	arCOG01550	C	HyaB	Ni,Fe-hydrogenase I large subunit	22204176	182	Crenarchaeota	Acidianus ambivalens
Acd1_1574	C4	arCOG00333	C	GlpC	Fe-S oxidoreductase	22204175	63.2	Crenarchaeota	Acidianus ambivalens
Acd1_1575	C4	arCOG06072	S		Uncharacterized conserved protein	22204174	214	Crenarchaeota	Acidianus ambivalens
Acd1_1576	C4	arCOG02474	C	HyaA	Ni,Fe-hydrogenase I small subunit	22204173	699	Crenarchaeota	Acidianus ambivalens
Acd1_1577	C5	arCOG01385	M	WcaA	Glycosyltransferase	284997362	285	Crenarchaeota	Sulfolobus islandicus L.D.8.5
Acd1_1578	C5	arCOG06000	S		Uncharacterized conserved protein	325968616	318	Crenarchaeota	Vulcanisaeta moutnovskia 768-28
Acd1_1579	C5	arCOG05999	S		Uncharacterized conserved protein	325968615	307	Crenarchaeota	Vulcanisaeta moutnovskia 768-28
Acd1_1580	C5	arCOG03721	R		HEPN domain	229582420	270	Crenarchaeota	Sulfolobus islandicus Y.N.15.51
Acd1_1581	C5		S		Uncharacterized protein	229579803	74.7	Crenarchaeota	Sulfolobus islandicus Y.G.57.14
Acd1_1582	C5	arCOG03042	R	NrfG	TPR repeats containing protein	22204169	153	Crenarchaeota	Acidianus ambivalens
Acd1_1583	C5	arCOG10353	S		Uncharacterized conserved protein	15898779	811	Crenarchaeota	Sulfolobus solfataricus P2
Acd1_1584	C5	arCOG10265	S		Uncharacterized conserved protein	15898780	143	Crenarchaeota	Sulfolobus solfataricus P2
Acd1_1585	C5	arCOG00585	D	Mrp	Mrp family protein, ATPase, contains iron-sulfur cluster	332795908	212	Crenarchaeota	Acidianus hospitalis W1
Acd1_1586	C5	arCOG04426	R	HybF	Zn finger protein HypA/HybF (possibly regulating hydrogenase expression)	332795909	1336	Crenarchaeota	Acidianus hospitalis W1
Acd1_1587	C5	arCOG01981	K	SUA7	Transcription initiation factor TFIIIB, Brf1 subunit/Transcription initiation factor TFIIB	15920551	69.7	Crenarchaeota	Sulfolobus tokodaii str. 7
Acd1_1588	C5	arCOG04223	J	SUI1	Translation initiation factor 1 (eIF-1/SUI1)	15920552	416	Crenarchaeota	Sulfolobus tokodaii str. 7
Acd1_1589	C5	arCOG04341	K	RPC10	DNA-directed RNA polymerase, subunit RPC10 (contains C4-type Zn-finger)	15897375	291	Crenarchaeota	Sulfolobus solfataricus P2
Acd1_1590	C5	arCOG01700	E	SpeB	Arginase family enzyme	15920554	194	Crenarchaeota	Sulfolobus tokodaii str. 7
Acd1_1591	C5	arCOG00405	J	GRS1	Glycyl-tRNA synthetase (class II)	332796370	81.3	Crenarchaeota	Acidianus hospitalis W1
Acd1_1592	C5	arCOG04224	S		Uncharacterized conserved protein	15920556	316	Crenarchaeota	Sulfolobus tokodaii str. 7
Acd1_1593	C5	arCOG05942	S		Uncharacterized conserved protein	15920557	330	Crenarchaeota	Sulfolobus tokodaii str. 7
Acd1_1594	C5	arCOG02640	P	-	Phosphate transport regulator (distant homolog of PhoU)	15920558	105	Crenarchaeota	Sulfolobus tokodaii str. 7
Acd1_1595	C5	arCOG01701	J	SEN2	tRNA splicing endonuclease	227827933	616	Crenarchaeota	Sulfolobus islandicus M.14.25
Acd1_1596	C5	arCOG05464	S		Uncharacterized conserved protein	146304964	423	Crenarchaeota	Metallosphaera sedula DSM 5348
Acd1_1597	C5	arCOG01703	H	UbiX	3-polyprenyl-4-hydroxybenzoate decarboxylase	70606646	96.3	Crenarchaeota	Sulfolobus acidocaldarius DSM 639
Acd1_1598	C5	arCOG05424	S		Zn-ribbon protein	146304966	344	Crenarchaeota	Metallosphaera sedula DSM 5348
Acd1_1599	C5	arCOG00574	G		Ribulose 1,5-bisphosphate synthetase, converts PRPP to RuBP, flavoprotein	15920562	636	Crenarchaeota	Sulfolobus tokodaii str. 7
Acd1_1600	C5	arCOG04182	J	RPS24A	Ribosomal protein S24E	15920563	498	Crenarchaeota	Sulfolobus tokodaii str. 7
Acd1_1601	C5	arCOG04183	J	RPS31	Ribosomal protein S27AE	15920564	281	Crenarchaeota	Sulfolobus tokodaii str. 7
Acd1_1602	C5	arCOG01183	L	Kae1/QRI7	Subunit of KEOPS complex, contains a domain with ASKHA fold and RIO-type kinase (AP-endonuclease activity)	332796380	176	Crenarchaeota	Acidianus hospitalis W1
Acd1_1603	C5	arCOG01185	T	-	Mn2+-dependent serine/threonine protein kinase	15920566	280	Crenarchaeota	Sulfolobus tokodaii str. 7
Acd1_1604	C5	arCOG04184	F	-	Xanthosine triphosphate pyrophosphatase	15920567	560	Crenarchaeota	Sulfolobus tokodaii str. 7
Acd1_1605	C5	arCOG01617	C	Tas	Predicted oxidoreductase (related to aryl-alcohol dehydrogenase)	227827943	232	Crenarchaeota	Sulfolobus islandicus M.14.25
Acd1_1606	C5	arCOG05941	S		Uncharacterized conserved protein	146304974	32	Crenarchaeota	Metallosphaera sedula DSM 5348
Acd1_1607	C5	arCOG02459	C	NtpC	Archaeal/vacuolar-type H+-ATPase subunit C	332796385	85.9	Crenarchaeota	Acidianus hospitalis W1
Acd1_1608	C5	arCOG05939	S		Uncharacterized conserved protein	70606635	34.7	Crenarchaeota	Sulfolobus acidocaldarius DSM 639

Acd1_1609	C5	arCOG00978	J	GCD14	tRNA(1-methyladenosine) methyltransferase	15920572	308	Crenarchaeota	Sulfolobus tokodaii str. 7
Acd1_1610	C5	arCOG01009	V	-	Ribbon-helix-helix protein, copG family	70606633	147	Crenarchaeota	Sulfolobus acidocaldarius DSM 639
Acd1_1611	C5	arCOG02037	K	-	Sugar-specific transcriptional regulator TrmB	15920573	39.3	Crenarchaeota	Sulfolobus tokodaii str. 7
Acd1_1612	C5	arCOG04327	J	-	Ribosomal protein S25	15920574	692	Crenarchaeota	Sulfolobus tokodaii str. 7
Acd1_1613	C5	arCOG00062	R	-	Predicted amidohydrolase	15920575	34.3	Crenarchaeota	Sulfolobus tokodaii str. 7
Acd1_1614	C5	arCOG01526	L	-	Reverse gyrase	42559518	1061	Crenarchaeota	Sulfolobus tokodaii str. 7
Acd1_1615	C5	arCOG01308	O	Cdc48	ATPase of the AAA+ class , CDC48 family	332796393	144	Crenarchaeota	Acidianus hospitalis W1
Acd1_1616	C5	arCOG01696	G	-	2,3-bisphosphoglycerate-independent phosphoglycerate mutase	15920579	192	Crenarchaeota	Sulfolobus tokodaii str. 7
Acd1_1617	C5		V		Small Multidrug Resistance (SMR) family protein	288924912	229	Bacteroidetes/Chlorobi group	Prevotella buccae D17
Acd1_1618	C5	arCOG01373	M	Gmd	GDP-D-mannose dehydratase	270008787	164	Opisthokonta	Tribolium castaneum
Acd1_1619	C5	arCOG03812	S	-	Predicted membrane protein	325968342	164	Crenarchaeota	Vulcanisaeta moutnovskia 768-28
Acd1_1620	C5	arCOG01419	M	RfaG	Glycosyltransferase	70607630	702	Crenarchaeota	Sulfolobus acidocaldarius DSM 639
Acd1_1621	C5		S		Uncharacterized protein	15920191	186	Crenarchaeota	Sulfolobus tokodaii str. 7
Acd1_1622	C5	arCOG00895	M	WcaA	Glycosyltransferase	374328281	245	Crenarchaeota	Pyrobaculum sp. 1860
Acd1_1623	C5	arCOG04076	S	-	Uncharacterized conserved protein	70606626	577	Crenarchaeota	Sulfolobus acidocaldarius DSM 639
Acd1_1624	C5	arCOG04077	K	Spt4	Transcription elongation factor Spt4/RpoE2, zinc finger protein	15920581	182	Crenarchaeota	Sulfolobus tokodaii str. 7
Acd1_1625	C5	arCOG00675	K	RPB7	DNA-directed RNA polymerase, subunit E'	15920582	131	Crenarchaeota	Sulfolobus tokodaii str. 7
Acd1_1626	C5	arCOG04312	R	-	PIN domain containing protein	70606623	394	Crenarchaeota	Sulfolobus acidocaldarius DSM 639
Acd1_1627	C5	arCOG01563	J	GCD11	Translation initiation factor 2, gamma subunit (eIF-2gamma; GTPase)	70606622	120	Crenarchaeota	Sulfolobus acidocaldarius DSM 639
Acd1_1628	C5	arCOG01946	J	RPS6A	Ribosomal protein S6E (S10)	161751100	59.7	Crenarchaeota	Sulfolobus tokodaii str. 7
Acd1_1629	C5	arCOG02246	P	-	Sirohydrochlorin ferrochelatase	332796400	39.7	Crenarchaeota	Acidianus hospitalis W1
Acd1_1630	C5	arCOG04185	J	RpsO	Ribosomal protein S15P	15920587	536	Crenarchaeota	Sulfolobus tokodaii str. 7
Acd1_1631	C5	arCOG01876	E	MetE	Methionine synthase II (cobalamin-independent)	15920588	526	Crenarchaeota	Sulfolobus tokodaii str. 7
Acd1_1632	C5	arCOG01877	E	MetE	Methionine synthase II (cobalamin-independent)	70606617	74.3	Crenarchaeota	Sulfolobus acidocaldarius DSM 639
Acd1_1633	C5	arCOG00488	L	DnaN	DNA polymerase sliding clamp subunit (PCNA homolog)	13124404	898	Crenarchaeota	Sulfurisphaera ohwakuensis
Acd1_1634	C5	arCOG00106	H	CobB	Cobyrinic acid a,c-diamide synthase	15920592	311	Crenarchaeota	Sulfolobus tokodaii str. 7
Acd1_1635	C5	arCOG04319	S	-	Predicted membrane protein	15920593	246	Crenarchaeota	Sulfolobus tokodaii str. 7
Acd1_1636	C5		S		Uncharacterized protein				
Acd1_1637	C5	arCOG00488	L	DnaN	DNA polymerase sliding clamp subunit (PCNA homolog)	15920600	792	Crenarchaeota	Sulfolobus tokodaii str. 7
Acd1_1638	C5	arCOG05938	K	Rpo13	DNA-directed RNA polymerase subunit 13	15920601	94.7	Crenarchaeota	Sulfolobus tokodaii str. 7
Acd1_1639	C5	arCOG00285	L	-	HerA helicase	15920603	48.9	Crenarchaeota	Sulfolobus tokodaii str. 7
Acd1_1640	C5	arCOG00557	R	Lhr	Lhr-like helicase	15920604	173	Crenarchaeota	Sulfolobus tokodaii str. 7
Acd1_1641	C5	arCOG04167	J	RPL14A	Ribosomal protein L14E/L6E/L27E	146302840	48.9	Crenarchaeota	Metallosphaera sedula DSM 5348
Acd1_1642	C5	arCOG06042	J	TruB	DKCLD domain, TruB/PUA associated	70606602	949	Crenarchaeota	Sulfolobus acidocaldarius DSM 639
Acd1_1643	C5	arCOG00987	J	TruB	Pseudouridine synthase	15920607	76.3	Crenarchaeota	Sulfolobus tokodaii str. 7
Acd1_1644	C5	arCOG00110	J	RsmC	16S RNA G1207 methylase RsmC	15920608	346	Crenarchaeota	Sulfolobus tokodaii str. 7
tRNASerTGA					tRNASerTGA				
Acd1_1645	C5	arCOG02960	R	-	Predicted aminopeptidase, Iap family	15922218	158	Crenarchaeota	Sulfolobus tokodaii str. 7
Acd1_1646	C5	arCOG03692	S		Uncharacterized conserved protein	330835516	180	Crenarchaeota	Metallosphaera cuprina Ar-4
Acd1_1647	C5		S		Uncharacterized protein				
Acd1_1648	C5	arCOG07284	S		Uncharacterized conserved protein	238620076	465	Crenarchaeota	Sulfolobus islandicus M.16.4
Acd1_1649	C5	arCOG08462	S		Uncharacterized conserved protein	284175218	171	Crenarchaeota	Sulfolobus solfataricus 98/2
Acd1_1650	C5		S		Uncharacterized protein				
Acd1_1651	C5	arCOG09897	S		Uncharacterized conserved protein	284807120	85.1	Crenarchaeota	Sulfolobus islandicus L.D.8.5
Acd1_1652	C5		S		Uncharacterized protein	284807119	140	Crenarchaeota	Sulfolobus islandicus L.D.8.5
Acd1_1653	C5	arCOG00144	G	ProP	Permease of the major facilitator superfamily	332796720	279	Crenarchaeota	Acidianus hospitalis W1
Acd1_1654	C5		S		Uncharacterized protein				
Acd1_1655	C5	arCOG08380	S		Uncharacterized conserved protein	238618752	638	Crenarchaeota	Sulfolobus islandicus M.16.4
Acd1_1656	C5	arCOG09871	S		SWIM Zn-finger	332796124	280	Crenarchaeota	Acidianus hospitalis W1
Acd1_1657	C5	arCOG01068	C	Lpd	Pyruvate/2-oxoglutarate dehydrogenase complex, dihydrolipoamide dehydrogenase (E3) component or related enzyme	307595489	199	Crenarchaeota	Vulcanisaeta distributa DSM 14429
Acd1_1658	C5	arCOG08078	S		TRASH domain-containing protein, metal-binding	384433025	197	Crenarchaeota	Sulfolobus solfataricus 98/2
Acd1_1659	C5		S		Uncharacterized protein				
Acd1_1660	C5		S		Uncharacterized protein	15920319	264	Crenarchaeota	Sulfolobus tokodaii str. 7
Acd1_1661	C5	arCOG02900	R	-	Uncharacterized protein containing a von Willebrand factor type A (vWA) domain	374632747	670	Crenarchaeota	Metallosphaera yellowstonensis MK1
Acd1_1662	C6	arCOG07245	S	0	Uncharacterized conserved protein	15898779	215	Crenarchaeota	Sulfolobus solfataricus P2
Acd1_1663	C6	arCOG01907	S	-	Uncharacterized conserved protein	332797638	202	Crenarchaeota	Acidianus hospitalis W1
Acd1_1664	C6	arCOG01064	R	HcaD	NAD(FAD)-dependent dehydrogenase	15922539	214	Crenarchaeota	Sulfolobus tokodaii str. 7
Acd1_1665	C6	arCOG04531	P	MntH	Mn2+ and Fe2+ transporter of the NRAMP family	15922217	305	Crenarchaeota	Sulfolobus tokodaii str. 7
Acd1_1667	C6	arCOG02682	G	ProP	Permease of the major facilitator superfamily	15922695	139	Crenarchaeota	Sulfolobus tokodaii str. 7
Acd1_1666	C6		S		Uncharacterized protein				
Acd1_1668	C6		S		Uncharacterized protein	302349294	275	Crenarchaeota	Acidilobus saccharovorans 345-15
Acd1_1669	C6		S		Uncharacterized protein				
Acd1_1670	C6	arCOG01423	R	-	RecB-like nuclease fused to coiled-coil domain	332797444	60.1	Crenarchaeota	Acidianus hospitalis W1
Acd1_1671	C6		S		Uncharacterized protein	374632567	47	Crenarchaeota	Metallosphaera yellowstonensis MK1
Acd1_1672	C6		S		Uncharacterized protein	227827418	287	Crenarchaeota	Sulfolobus islandicus M.14.25
Acd1_1673	C6	arCOG03169	R	-	Predicted ATPase (AAA+ superfamily)	374632567	273	Crenarchaeota	Metallosphaera yellowstonensis MK1
Acd1_1674	C6	arCOG01423	R	-	RecB-like nuclease fused to coiled-coil domain	332797444	31.2	Crenarchaeota	Acidianus hospitalis W1
Acd1_1675	C7	arCOG02657	V	Cas7/Cmr4	CRISPR system related protein, RAMP superfamily Cas7 group	124027590	42.7	Crenarchaeota	Hyperthermus butylicus DSM 5456
Acd1_1676	C7	arCOG03483	V	Cas3d	CRISPR system related heleicase, Cas3 (C-terminal HD nuclease domain)	385772759	189	Crenarchaeota	Sulfolobus islandicus HVE10/4
Acd1_1677	C7	arCOG05134	V	Cas5/Csc1	CRISPR associated protein, RAMP family Cas5 group	385772760	933	Crenarchaeota	Sulfolobus islandicus HVE10/4
Acd1_1678	C7	arCOG03482	K	Cas7/Csc2	CRISPR system related protein, RAMP superfamily Cas7 group	385772761	35.4	Crenarchaeota	Sulfolobus islandicus HVE10/4
Acd1_1679	C7		V	Csc3	CRISPR system related protein, predicted large subunit of Cascade complex	385772762	817	Crenarchaeota	Sulfolobus islandicus HVE10/4
Acd1_1680	C7	arCOG01440	V	Cas6	CRISPR system related protein, RAMP superfamily Cas6 group	385772764	111	Crenarchaeota	Sulfolobus islandicus HVE10/4
Acd1_1681	C7	arCOG01452	V	Cas1	CRISPR-associated protein Cas1	385772765	550	Crenarchaeota	Sulfolobus islandicus HVE10/4
Acd1_1682	C8	arCOG01671	H	UbiD	3-polyprenyl-4-hydroxybenzoate decarboxylase or related decarboxylase	307595548	157	Crenarchaeota	Vulcanisaeta distributa DSM 14429
Acd1_1683	C8		S		Uncharacterized protein				
Acd1_1684	C8	arCOG01669	S	-	Uncharacterized conserved protein	15922146	124	Crenarchaeota	Sulfolobus tokodaii str. 7
Acd1_1685	C8	arCOG03869	S		Uncharacterized conserved protein	15922718	149	Crenarchaeota	Sulfolobus tokodaii str. 7
Acd1_1686	C8	arCOG00132	G	ProP	Permease of the major facilitator superfamily	330834864	189	Crenarchaeota	Metallosphaera cuprina Ar-4
Acd1_1687	C8	arCOG02687	G	ProP	Permease of the major facilitator superfamily	15921095	155	Crenarchaeota	Sulfolobus tokodaii str. 7
Acd1_1688	C8	arCOG06025	S		Uncharacterized conserved protein	229578762	155	Crenarchaeota	Sulfolobus islandicus Y.G.57.14
Acd1_1689	C8	arCOG06046	S	-	Hemerythrin HHE cation binding domain containing protein	146304008	67	Crenarchaeota	Metallosphaera sedula DSM 5348
Acd1_1690	C8	arCOG01254	R		Predicted metal-dependent hydrolase related to alanyl-tRNA synthetase HxxxH domain and second additional domain	146304928	306	Crenarchaeota	Metallosphaera sedula DSM 5348
Acd1_1691	C8	arCOG00648	H	CobF	Precorrin-2 methylase	167038979	343	Firmicutes	Thermoanaerobacter sp. X514
Acd1_1692	C8		S		Uncharacterized protein				
Acd1_1693	C8	arCOG11297	S		Uncharacterized conserved protein	15922505	215	Crenarchaeota	Sulfolobus tokodaii str. 7
Acd1_1694	C8	arCOG00506	C	FpaA	Uncharacterized flavoprotein	15922504	246	Crenarchaeota	Sulfolobus tokodaii str. 7
Acd1_1695	C9		S		Uncharacterized protein				
Acd1_1696	C9		S		Uncharacterized protein				
Acd1_1697	C9		S		Uncharacterized protein				
Acd1_1698	C9		S		Uncharacterized protein				
Acd1_1699	C9		S		Uncharacterized protein				
