# Supplementary material for: Insights into archaeal evolution and symbiosis from the genomes of a nanoarchaeon and its inferred crenarchaeal host from Obsidian Pool, Yellowstone National Park
Source: Biol Direct. 2013 Apr 22;8:9. doi: 10.1186/1745-6150-8-9 (PMC3655853; doi:10.1186/1745-6150-8-9)
Supplement: Additional file 4 — Shows a maximum likelihood phylogeny of archaeal lysyl-tRNA synthetases of class II (arCOG00408). [file 1745-6150-8-9-S4.pdf]

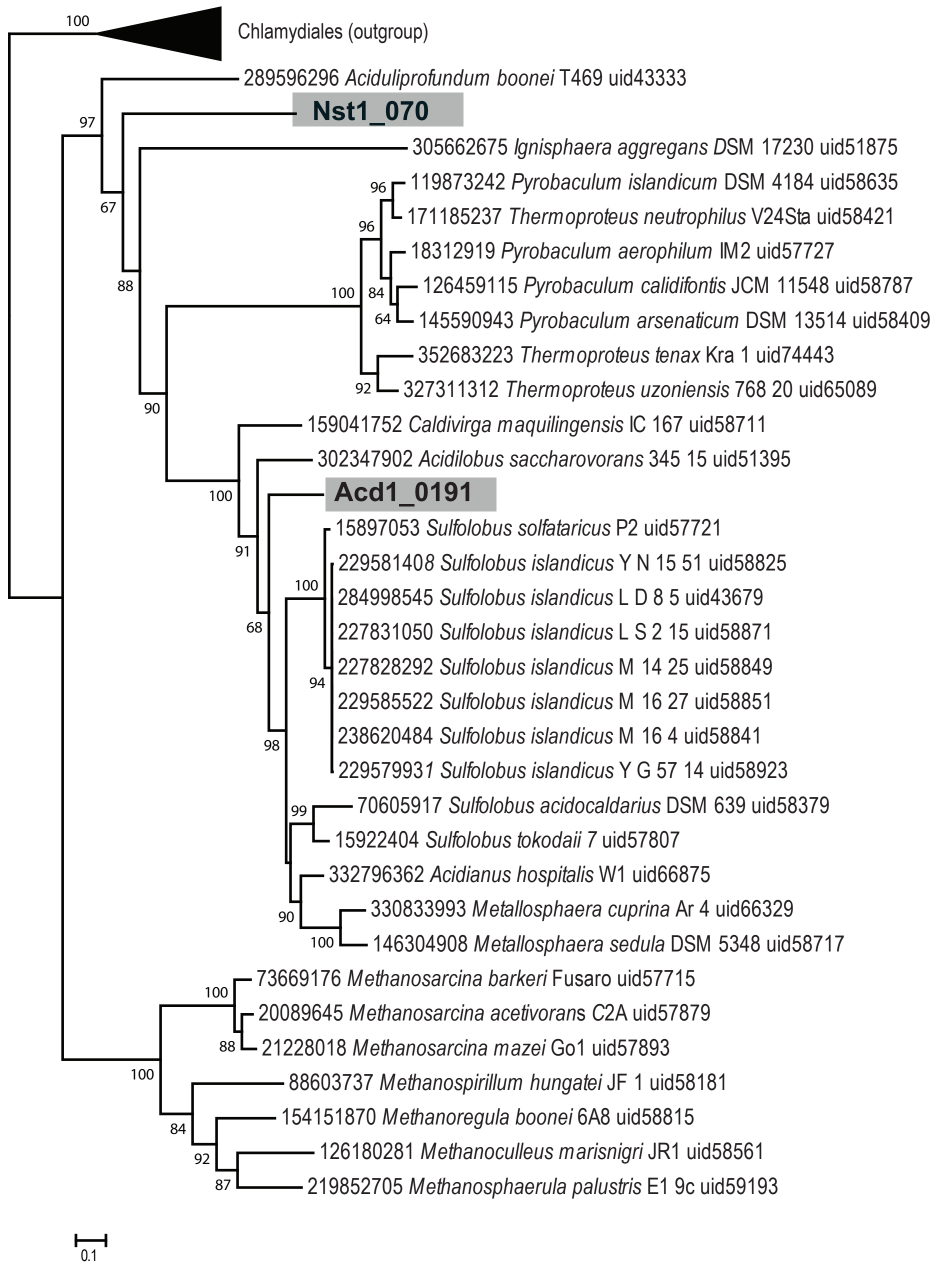

Additional file 4. Maximum likelihood phylogeny of archaeal lysyl-tRNA synthetases of class II (arCOG00408, COG1190).
