# Supplementary material for: Insights into archaeal evolution and symbiosis from the genomes of a nanoarchaeon and its inferred crenarchaeal host from Obsidian Pool, Yellowstone National Park
Source: Biol Direct. 2013 Apr 22;8:9. doi: 10.1186/1745-6150-8-9 (PMC3655853; doi:10.1186/1745-6150-8-9)
Supplement: Additional file 5 — Shows maximum likelihood phylogenies of archaeal FlaH archaellum and 3-dehydroquinate dehydratase (AroD, arCOG2097) proteins. [file 1745-6150-8-9-S5.pdf]

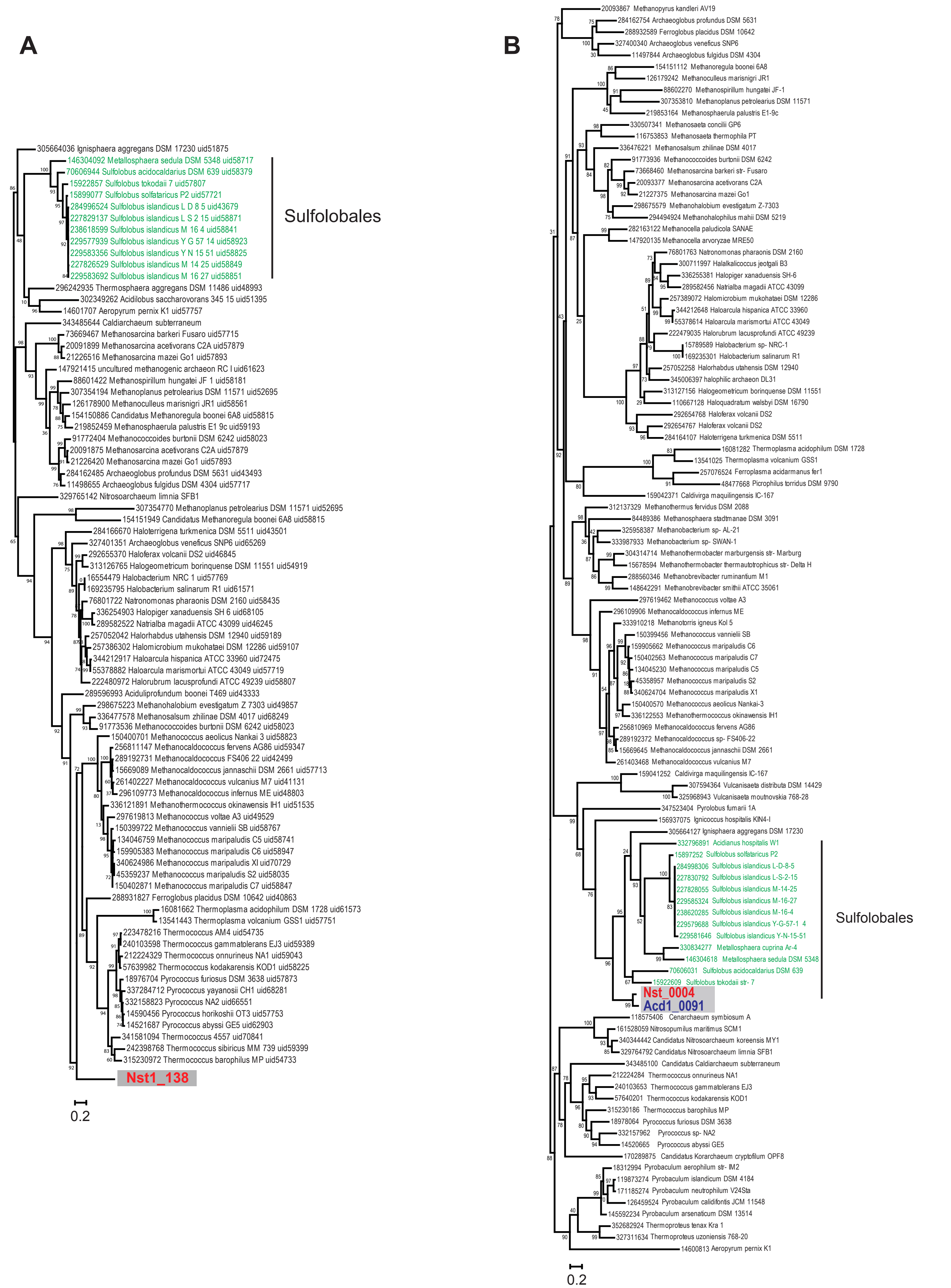

Additional file 5. Maximum likelihood phylogeny of archaeal FlaH archaellum (**A**) and 3-dehydroquinate dehydratase (AroD, arCOG2097)(**B**) proteins.
