# Supplementary material for: Insights into archaeal evolution and symbiosis from the genomes of a nanoarchaeon and its inferred crenarchaeal host from Obsidian Pool, Yellowstone National Park
Source: Biol Direct. 2013 Apr 22;8:9. doi: 10.1186/1745-6150-8-9 (PMC3655853; doi:10.1186/1745-6150-8-9)
Supplement: Additional file 3 — Shows maximum likelihood phylogenetic trees of the Archaea based on concatenated ribosomal protein gene sequences and RNA polymerase subunits from all the completely sequenced genomes. [file 1745-6150-8-9-S3.pdf]

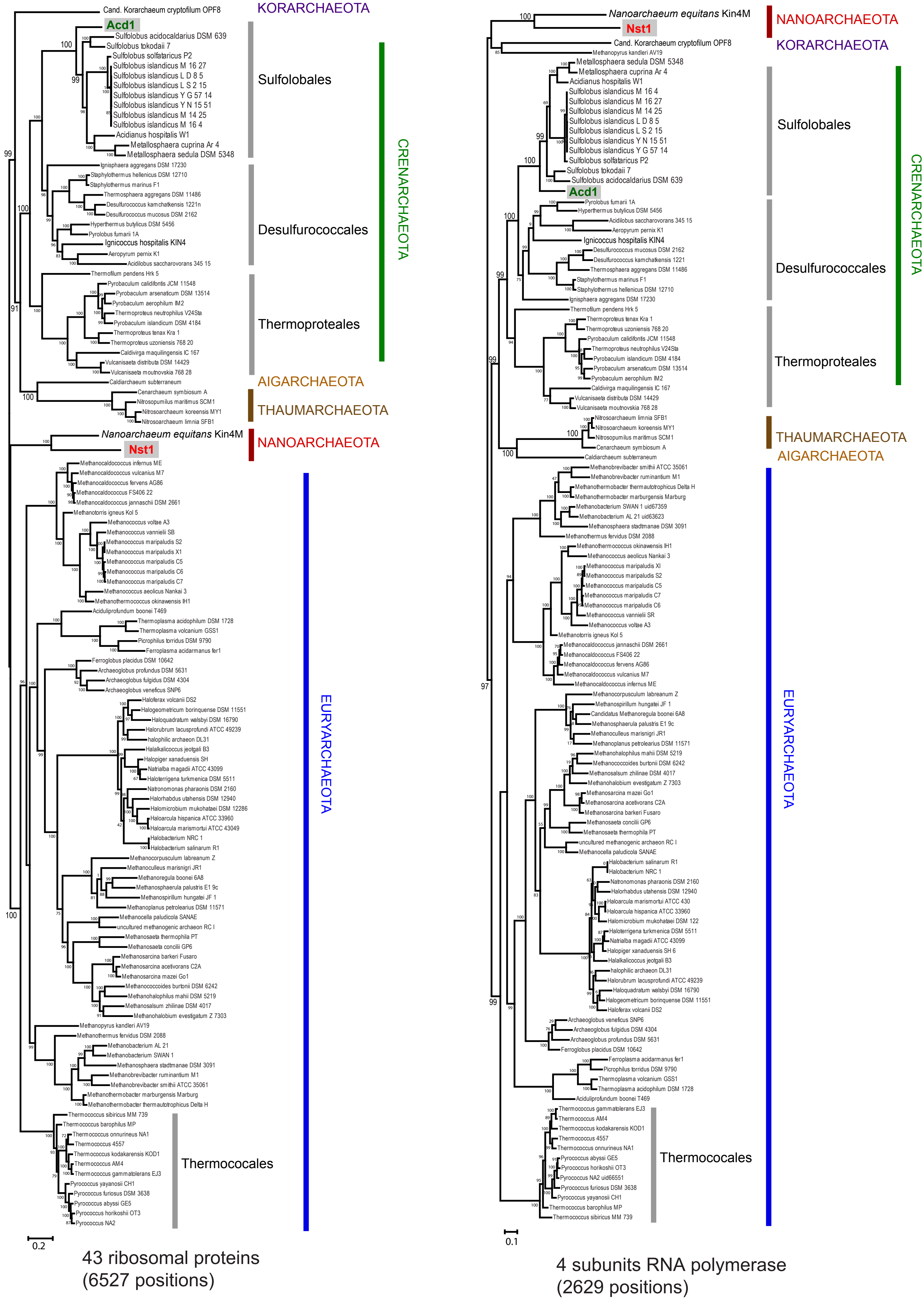

Additional file 3. Maximum likelihood phylogenies of the *Archaea* based on concatenated ribosomal protein gene sequences and RNA polymerase subunits.
