# Supplementary material for: Insights into archaeal evolution and symbiosis from the genomes of a nanoarchaeon and its inferred crenarchaeal host from Obsidian Pool, Yellowstone National Park
Source: Biol Direct. 2013 Apr 22;8:9. doi: 10.1186/1745-6150-8-9 (PMC3655853; doi:10.1186/1745-6150-8-9)
Supplement: Additional file 7 — Lists the gene gain and loss in Nanoarchaeota and inSulfolobales based on arCOG classification. [file 1745-6150-8-9-S7.pdf]

Additional file 7. Gene gain and loss in Nanoarchaeota and in Sulfolobales

	arCOG Family	COG correspondence	Gene Name	Functional group	Annotation
**Gain in both Nanoarchaeota**					
	arCOG00122	COG02265	TrmA	J	SAM-dependent methyltransferase related to tRNA (uracil-5-)-methyltransferase
	arCOG00284	COG00433		L	HerA helicase
	arCOG00424	COG00608		L	DHH superfamily phosphohydrolase/exonuclease
	arCOG01814			N	Type II secretion system protein F
	arCOG01818	COG00630	VirB11	N	Type IV secretory pathway, VirB11 component, or related ATPase involved in archaeal flagella biosynthesis
	arCOG00438	COG01239		R	AAA family ATPase
	arCOG02261			R	Protein containing OB-fold-like and PCI domains
	arCOG06946			S	Uncharacterized conserved membrane protein
	arCOG01572			S	Uncharacterized conserved membrane protein
	arCOG05037			S	Uncharacterized conserved protein
	arCOG00793	COG01468		V	CRISPR-associated protein, Cas4 family, PD-(D/E)XK superfamily nuclease
	arCOG04471	COG04083		U	Exosortase
**Loss in both Nanoarchaeota**					
	arCOG00570	COG00644	FixC	C	Dehydrogenase (flavoprotein)
	arCOG01068	COG01249	Lpd	C	Pyruvate/2-oxoglutarate dehydrogenase complex, dihydrolipoamide dehydrogenase (E3) component or related enzyme
	arCOG00333	COG00247	GlpC	C	Fe-S oxidoreductase
	arCOG00335	COG01139	-	C	Uncharacterized conserved protein containing ferredoxin-like domain
	arCOG00338	COG02048	SdhC	C	Succinate dehydrogenase subunit C
	arCOG01357	COG01032	-	C	Radical SAM superfamily enzyme
	arCOG01491	COG00243	BisC	C	Anaerobic dehydrogenase
	arCOG01537	COG00651	NuoM	C	NADH:ubiquinone oxidoreductase subunit 4 (chain M)
	arCOG01539	COG01009	NuoL	C	NADH dehydrogenase subunit L
	arCOG01554	COG00377	NuoB	C	F420H2 dehydrogenase subunit, related to NADH:ubiquinone oxidoreductase 20 kD subunit
	arCOG00337	COG00277	GlcD	C	FAD/FMN-containing dehydrogenase
	arCOG00506	COG00426	FpaA	C	Uncharacterized flavoprotein
	arCOG00571	COG01053	SdhA	C	Succinate dehydrogenase/fumarate reductase, flavoprotein subunit
	arCOG00853	COG00281	SfcA	C	Malic enzyme
	arCOG00869	COG01390	NtpE	C	Archaeal/vacuolar-type H+-ATPase subunit E
	arCOG00934	COG01313	PflX	C	Radical SAM superfamily enzyme
	arCOG00982	COG00371	GldA	C	Glycerol dehydrogenase or related enzyme
	arCOG01097	COG01592	-	C	Rubrerythrin
	arCOG01163	COG00473	LeuB	C	Isocitrate/isopropylmalate dehydrogenase
	arCOG01337	COG00045	SucC	C	Succinyl-CoA synthetase, beta subunit
	arCOG01339	COG00074	SucD	C	Succinyl-CoA synthetase, alpha subunit
	arCOG01462	COG01668	NatB	C	ABC-type Na+ efflux pump, permease component
	arCOG01500	COG00437	HybA	C	Fe-S-cluster-containing dehydrogenase component
	arCOG01540	COG01007	NuoN	C	NADH dehydrogenase subunit N
	arCOG01543	COG01143	NuoI	C	NADH dehydrogenase subunit I
	arCOG01545	COG00650	HyfC	C	Formate hydrogenlyase subunit 4
	arCOG01546	COG01005	NuoH	C	NADH dehydrogenase subunit H
	arCOG01547	COG03261	HycE	C	Ni,Fe-hydrogenase III large subunit and subunit G
	arCOG01548	COG00649	NuoD	C	NADH dehydrogenase subunit D
	arCOG01551	COG00852	NuoC	C	NADH dehydrogenase subunit C
	arCOG01553	COG03260	-	C	Ni,Fe-hydrogenase III small subunit
	arCOG01557	COG00838	NuoA	C	NADH dehydrogenase subunit A

arCOG01601	COG01013	PorB	C	Pyruvate:ferredoxin oxidoreductase or related 2-oxoacid:ferredoxin oxidoreductase, beta subunit
arCOG01603	COG01014	PorG	C	Pyruvate:ferredoxin oxidoreductase or related 2-oxoacid:ferredoxin oxidoreductase, gamma subunit
arCOG01605	COG01144	-	C	Pyruvate:ferredoxin oxidoreductase or related 2-oxoacid:ferredoxin oxidoreductase, delta subunit
arCOG01608	COG00674	PorA	C	Pyruvate:ferredoxin oxidoreductase or related 2-oxoacid:ferredoxin oxidoreductase, alpha subunit
arCOG01755	COG01052	LdhA	C	Lactate dehydrogenase or related 2-hydroxyacid dehydrogenase
arCOG02235	COG01148	HdrA	C	Heterodisulfide reductase, subunit A or related polyferredoxin
arCOG02459	COG01527	NtpC	C	Archaeal/vacuolar-type H+-ATPase subunit C
arCOG03073	COG00713	NuoK	C	NADH dehydrogenase subunit 4L (K,kappa)
arCOG04102	COG01436	NtpG	C	Archaeal/vacuolar-type H+-ATPase subunit F
arCOG04237	COG00372	GltA	C	Citrate synthase
arCOG04329	COG02441	-	C	Predicted butyrate kinase
arCOG04429	COG00680	HyaD	C	Ni,Fe-hydrogenase maturation factor
arCOG00024	COG00554	GlpK	C	Glycerol kinase
arCOG00104	COG01795	-	C	Formaldehyde-activating enzyme nesessary for methanogenesis
arCOG00246	COG00039	Mdh	C	Malate/lactate dehydrogenase
arCOG00296	COG01145	NapF	C	Ferredoxin
arCOG00509	COG00426	FpaA	C	Uncharacterized flavoprotein
arCOG00701	COG00584	UgpQ	C	Glycerophosphoryl diester phosphodiesterase
arCOG00706	COG02414	-	C	Aldehyde:ferredoxin oxidoreductase
arCOG00762	COG02191	-	C	Formylmethanofuran dehydrogenase subunit E
arCOG00958	COG01145	NapF	C	Ferredoxin
arCOG00959	COG01146	-	C	Ferredoxin
arCOG00964	COG01150	HdrC	C	Heterodisulfide reductase, subunit C
arCOG00984	COG01454	EutG	C	Alcohol dehydrogenase, class IV
arCOG01355	COG01032	-	C	Radical SAM superfamily enzyme
arCOG01359	COG01031	-	C	Radical SAM superfamily enzyme
arCOG01502	COG01142	HycB	C	Fe-S-cluster-containing hydrogenase component 2
arCOG01549	COG03259	FrhA	C	Coenzyme F420-reducing hydrogenase, alpha subunit
arCOG01602	COG01014	PorG	C	Pyruvate:ferredoxin oxidoreductase or related 2-oxoacid:ferredoxin oxidoreductase, gamma subunit
arCOG01607	COG00674	PorA	C	Pyruvate:ferredoxin oxidoreductase or related 2-oxoacid:ferredoxin oxidoreductase, alpha subunit
arCOG01609	COG04231	-	C	Indolepyruvate ferredoxin oxidoreductase, alpha and beta subunit
arCOG01705	COG01036	-	C	Archaeal flavoprotein
arCOG02077	COG00822	IscU	C	NifU homolog involved in Fe-S cluster formation
arCOG02095	COG05016	-	C	Pyruvate/oxaloacetate carboxyltransferase
arCOG02236	COG01148	HdrA	C	Heterodisulfide reductase, subunit A or related polyferredoxin
arCOG02429	COG01151	-	C	6Fe-6S prismane cluster-containing protein
arCOG02461	COG01146	-	C	Ferredoxin
arCOG02472	COG01941	FrhG	C	Coenzyme F420-reducing hydrogenase, gamma subunit
arCOG02475	COG01908	FrhD	C	Coenzyme F420-reducing hydrogenase, delta subunit
arCOG02740	COG01600	-	C	Uncharacterized Fe-S protein
arCOG03363	COG02811	NtpF	C	Archaeal/vacuolar-type H+-ATPase subunit H
arCOG04073	COG01149	-	C	MinD superfamily P-loop ATPase containing an inserted ferredoxin domain
arCOG04279	COG01786	-	C	Swiveling domain associated with predicted aconitase

arCOG04358	COG01526	FdhD	C	Uncharacterized protein required for formate dehydrogenase activity
arCOG04386	COG01620	LldP	C	L-lactate permease
arCOG04391	COG01773	-	C	Rubredoxin
arCOG04406	COG01838	FumA	C	Tartrate dehydratase beta subunit/Fumarate hydratase class I, C-terminal domain
arCOG04407	COG01951	TtdA	C	Tartrate dehydratase alpha subunit/Fumarate hydratase class I, N-terminal domain
arCOG04537	COG01894	NuoF	C	NADH:ubiquinone oxidoreductase, NADH-binding (51 kD) subunit
arCOG04874	COG02055	-	C	Malate/L-lactate dehydrogenase
arCOG04890	COG01905	NuoE	C	NADH:ubiquinone oxidoreductase 24 kD subunit
arCOG05014	COG02181	HdrE	C	CoB--CoM heterodisulfide reductase subunit E
arCOG05128	COG01145	NapF	C	Ferredoxin
arCOG06130	COG01882	PflD	C	Pyruvate-formate lyase
arCOG03483	COG01203	V	Cas3d	CRISPR system related heleicase, Cas3 (C-terminal HD nuclease domain)
arCOG00585	COG00489	Mrp	D	Mrp family protein, ATPase, contains iron-sulfur cluster
arCOG00586	COG01192	Soj	D	ATPase involved in chromosome partitioning, ParA family
arCOG01307	COG00464	Vps4	D	Cell division ATPase of the AAA+ class, ESCRT system component
arCOG00370	COG01196	Smc	D	Chromosome segregation ATPase
arCOG00587	COG03640	CooC	D	CO dehydrogenase maturation factor
arCOG00588	COG03640	CooC	D	CO dehydrogenase maturation factor
arCOG04701	COG00239	CrcB	D	Integral membrane protein possibly involved in chromosome condensation
arCOG01303	COG00509	GcvH	E	Glycine cleavage system H protein (lipoate-binding)
arCOG01698	COG00065	LeuC	E	Homoaconitate hydratase/3-isopropylmalate dehydratase large subunit family protein
arCOG02092	COG00119	LeuA	E	Isopropylmalate/homocitrate/citramalate synthase
arCOG00082	COG00075	-	E	Serine-pyruvate aminotransferase/archaeal aspartate aminotransferase
arCOG00279	COG01586	SpeD	E	S-adenosylmethionine decarboxylase/arginine decarboxylase
arCOG00494	COG00136	Asd	E	Aspartate-semialdehyde dehydrogenase
arCOG00619	COG00069	GltB	E	Glutamate synthase domain 2 and ferredoxin domain
arCOG00912	COG00078	ArgF	E	Ornithine carbamoyltransferase
arCOG01035	COG02423	-	E	Alanine dehydrogenase, mu-crystallin homolog
arCOG01351	COG00460	ThrA	E	Homoserine dehydrogenase
arCOG01594	COG00458	CarB	E	Carbamoylphosphate synthase large subunit
arCOG01909	COG00174	GlnA	E	Glutamine synthetase
arCOG02230	COG00066	LeuD	E	3-isopropylmalate dehydratase small subunit
arCOG04273	COG00079	HisC	E	Histidinol-phosphate/aromatic aminotransferase or cobyric acid decarboxylase
arCOG00009	COG00531	PotE	E	Amino acid transporter
arCOG00050	COG00421	SpeE	E	Spermidine synthase
arCOG00060	COG00626	MetC	E	Cystathionine beta-lyase/cystathionine gamma-synthase
arCOG00064	COG00505	CarA	E	Carbamoylphosphate synthase small subunit
arCOG00070	COG00112	GlyA	E	Glycine/serine hydroxymethyltransferase
arCOG00076	COG01003	GcvP	E	Glycine cleavage system protein P (pyridoxal-binding), C-terminal domain
arCOG00077	COG00403	GcvP	E	Glycine cleavage system protein P (pyridoxal-binding), N-terminal domain
arCOG00086	COG00512	PabA	E	Anthranilate/para-aminobenzoate synthase component II
arCOG00089	COG00118	HisH	E	Glutamine amidotransferase
arCOG00095	COG00067	GltB	E	Glutamate synthase domain 1

arCOG00096	COG00070	GltB	E	Glutamate synthase domain 3
arCOG00112	COG00137	ArgG	E	Argininosuccinate synthase
arCOG00181	COG00444	DppD	E	ABC-type dipeptide/oligopeptide/nickel transport system, ATPase component
arCOG00184	COG04608	AppF	E	ABC-type oligopeptide transport system, ATPase component
arCOG00495	COG00002	ArgC	E	Acetylglutamate semialdehyde dehydrogenase
arCOG00617	COG00107	HisF	E	Imidazoleglycerol-phosphate synthase
arCOG00618	COG00106	HisA	E	Phosphoribosylformimino-5-aminoimidazole carboxamide ribonucleotide (ProFAR) isomerase
arCOG00749	COG01173	DppC	E	ABC-type dipeptide/oligopeptide/nickel transport system, permease component
arCOG00751	COG00601	DppB	E	ABC-type dipeptide/oligopeptide/nickel transport system, permease component
arCOG00755	COG00665	DadA	E	Glycine/D-amino acid oxidase (deaminating)
arCOG00756	COG00404	GcvT	E	Glycine cleavage system T protein (aminomethyltransferase)
arCOG00861	COG00527	LysC	E	Aspartokinase
arCOG00862	COG00548	ArgB	E	Acetylglutamate kinase
arCOG00914	COG04992	ArgD	E	Ornithine/acetylornithine aminotransferase
arCOG01000	COG00006	PepP	E	Xaa-Pro aminopeptidase
arCOG01025	COG01685	-	E	Archaeal shikimate kinase
arCOG01027	COG00083	ThrB	E	Homoserine kinase
arCOG01033	COG00169	AroE	E	Shikimate 5-dehydrogenase
arCOG01086	COG00159	TrpA	E	Tryptophan synthase alpha chain
arCOG01088	COG00134	TrpC	E	Indole-3-glycerol phosphate synthase
arCOG01130	COG00436	-	E	Aspartate/tyrosine/aromatic aminotransferase
arCOG01226	COG01703	ArgK	E	Putative periplasmic protein kinase ArgK or related GTPase of G3E family
arCOG01316	COG00591	PutP	E	Na+/proline symporter
arCOG01430	COG00031	CysK	E	Cysteine synthase
arCOG01434	COG00498	ThrC	E	Threonine synthase and cysteate synthase
arCOG01459	COG01063	Tdh	E	Threonine dehydrogenase or related Zn-dependent dehydrogenase
arCOG01588		LysW	E	lysine biosynthesis protein LysW
arCOG01646	COG01506	DAP2	E	Dipeptidyl aminopeptidase/acylaminoacyl-peptidase
arCOG01678	COG01812	MetK	E	Archaeal S-adenosylmethionine synthetase
arCOG01748	COG00165	ArgH	E	Argininosuccinate lyase
arCOG01768	COG00705	GlpG	E	Membrane associated serine protease
arCOG01876	COG00620	MetE	E	Methionine synthase II (cobalamin-independent)
arCOG01947	COG01280	RhtB	E	Putative threonine efflux protein
arCOG01983	COG00135	TrpF	E	Phosphoribosylanthranilate isomerase
arCOG01998	COG00028	IlvB	E	Acetolactate synthase large subunit or other thiamine pyrophosphate-requiring enzyme
arCOG02012	COG00547	TrpD	E	Anthranilate phosphoribosyltransferase
arCOG02014	COG00147	TrpE	E	Anthranilate/para-aminobenzoate synthase component I
arCOG02208	COG00040	HisG	E	ATP phosphoribosyltransferase
arCOG02676	COG00139	HisI	E	Phosphoribosyl-AMP cyclohydrolase
arCOG02677	COG00140	HisI	E	Phosphoribosyl-ATP pyrophosphohydrolase
arCOG02706	COG00346	GloA	E	Lactoylglutathione lyase or related enzyme
arCOG04044	COG01830		E	2-amino-3,7-dideoxy-D-threo-hept-6-ulosonic acid synthase, DhnA-aldolase family
arCOG04045	COG00129	IlvD	E	Dihydroxyacid dehydratase/phosphogluconate dehydratase
arCOG04133	COG00082	AroC	E	Chorismate synthase
arCOG04134	COG00128	AroA	E	5-enolpyruvylshikimate-3-phosphate synthase
arCOG04172	COG00329	DapA	E	Dihydrodipicolinate synthase/N-acetylneuraminate lyase
arCOG04352	COG00141	HisD	E	Histidinol dehydrogenase
arCOG04398	COG00131	HisB	E	Imidazoleglycerol-phosphate dehydratase
arCOG04465	COG00059	IlvC	E	Ketol-acid reductoisomerase

arCOG04779	COG01446	-	E	Asparaginase
arCOG00066	COG01104	NifS	E	Cysteine sulfinate desulfinase/cysteine desulfurase or related enzyme
arCOG00071	COG00367	AsnB	E	Asparagine synthase (glutamine-hydrolyzing)
arCOG00073	COG00175	CysH	E	PAPS reductase related enzyme fused to RNA-binding PUA domain and ferredoxin domain
arCOG00177	COG03842	PotA	E	ABC-type spermidine/putrescine transport system, ATPase component
arCOG00182	COG00444	DppD	E	ABC-type dipeptide/oligopeptide/nickel transport system, ATPase component
arCOG00455	COG00345	ProC	E	Pyrroline-5-carboxylate reductase
arCOG00748	COG01173	DppC	E	ABC-type dipeptide/oligopeptide/nickel transport system, permease component
arCOG00771	COG01680		E	CubicO group peptidase, beta-lactamase class C family
arCOG00811	COG00440	IlvH	E	Acetolactate synthase, small subunit
arCOG00863	COG00549	ArcC	E	Carbamate kinase
arCOG00915	COG00160	GabT	E	4-aminobutyrate aminotransferase or related aminotransferase
arCOG00923	COG01126	GlnQ	E	ABC-type polar amino acid transport system, ATPase component
arCOG01021	COG00683	LivK	E	ABC-type branched-chain amino acid transport system, periplasmic component
arCOG01158	COG00560	SerB	E	Phosphoserine phosphatase
arCOG01292	COG00493	GltD	E	NADPH-dependent glutamate synthase beta chain or related oxidoreductase
arCOG01511	COG00145	HyuA	E	N-methylhydantoinase A/acetone carboxylase, beta subunit
arCOG01512	COG00146	HyuB	E	N-methylhydantoinase B/acetone carboxylase, alpha subunit
arCOG01534	COG00747	DdpA	E	ABC-type transport system, periplasmic component
arCOG01798	COG00765	HisM	E	ABC-type amino acid transport system, permease component
arCOG01799	COG00834	HisJ	E	ABC-type amino acid transport/signal transduction system, periplasmic component/domain
arCOG01890	COG02309	AmpS	E	Leucyl aminopeptidase (aminopeptidase T)
arCOG02165	COG01305	-	E	Transglutaminase-like enzyme, putative cysteine protease
arCOG02167	COG01305	-	E	Transglutaminase-like enzyme, putative cysteine protease
arCOG02169	COG01305	-	E	Transglutaminase-like enzyme, putative cysteine protease
arCOG02255	COG00253	DapF	E	Diaminopimelate epimerase
arCOG02268	COG00019	LysA	E	Diaminopimelate decarboxylase
arCOG02305	COG00347	GlnK	E	Nitrogen regulatory protein PII
arCOG02462	COG01878	-	E	Kynurenine formamidase
arCOG02486	COG01305	-	E	Transglutaminase-like enzyme, putative cysteine protease
arCOG03109	COG01834	-	E	N-Dimethylarginine dimethylaminohydrolase
arCOG03377			E	Serine protease
arCOG03602	COG04690	PepD	E	Dipeptidase
arCOG04333	COG00436	-	E	Aspartate/tyrosine/aromatic aminotransferase
arCOG04353	COG01465	-	E	3-dehydroquinate synthase
arCOG00087	COG00518	GuaA	F	GMP synthase - Glutamine amidotransferase domain
arCOG00695	COG00402	SsnA	F	Cytosine deaminase or related metal-dependent hydrolase
arCOG00858	COG00528	PyrH	F	Uridylate kinase
arCOG01723	COG01437	CyaB	F	Adenylate cyclase, class 2 (thermophilic)
arCOG02464	COG00041	PurE	F	Phosphoribosylcarboxyaminoimidazole (NCAIR) mutase
arCOG04309	COG01912	-	F	S-adenosyl-l-methionine hydroxide adenosyltransferase
arCOG04346	COG01759	-	F	5-formaminoimidazole-4-carboxamide-1-beta-D-ribofuranosyl 5'-monophosphate synthetase (purine biosynthesis)
arCOG00029	COG00461	PyrE	F	Orotate phosphoribosyltransferase

arCOG00031	COG00503	Apt	F	Adenine/guanine phosphoribosyltransferase or related PRPP-binding protein
arCOG00063	COG00504	PyrG	F	CTP synthase (UTP-ammonia lyase)
arCOG00067	COG00462	PrsA	F	Phosphoribosylpyrophosphate synthetase
arCOG00081	COG00284	PyrF	F	Orotidine-5'-phosphate decarboxylase
arCOG00085	COG00519	GuaA	F	GMP synthase, PP-ATPase domain/subunit
arCOG00093	COG00034	PurF	F	Glutamine phosphoribosylpyrophosphate amidotransferase
arCOG00102	COG00047	PurL	F	Phosphoribosylformylglycinamidine (FGAM) synthase, glutamine amidotransferase domain
arCOG00603	COG00167	PyrD	F	Dihydroorotate dehydrogenase
arCOG00639	COG00150	PurM	F	Phosphoribosylaminoimidazole (AIR) synthetase
arCOG00641	COG00046	PurL	F	Phosphoribosylformylglycinamidine (FGAM) synthase, synthetase domain
arCOG00689	COG00044	PyrC	F	Dihydroorotase or related cyclic amidohydrolase
arCOG00692	COG00402	SsnA	F	Cytosine deaminase or related metal-dependent hydrolase
arCOG00911	COG00540	PyrB	F	Aspartate carbamoyltransferase, catalytic chain
arCOG01034	COG01618	-	F	Predicted nucleotide kinase
arCOG01037	COG01102	Cmk	F	Cytidylate kinase
arCOG01075	COG01051	-	F	NUDIX family hydrolase
arCOG01221	COG01986	-	F	Inosine/xanthosine triphosphatase
arCOG01324	COG02820	Udp	F	Uridine phosphorylase
arCOG01327	COG00005	Pnp	F	Purine nucleoside phosphorylase
arCOG01747	COG00015	PurB	F	Adenylosuccinate lyase
arCOG04184	COG00127	-	F	Xanthosine triphosphate pyrophosphatase
arCOG04202	COG02429	-	F	Archaeal GTP cyclohydrolase III
arCOG04229	COG01781	PyrI	F	Aspartate carbamoyltransferase, regulatory subunit
arCOG04387	COG00104	PurA	F	Adenylosuccinate synthase
arCOG04415	COG00151	PurD	F	Phosphoribosylamine-glycine ligase
arCOG04421	COG00152	PurC	F	Phosphoribosylaminoimidazolesuccinocarbo xamide (SAICAR) synthase
arCOG04462	COG01828	PurS	F	Phosphoribosylformylglycinamidine (FGAM) synthase, PurS component
arCOG00612	COG00516	GuaB	F	IMP dehydrogenase/GMP reductase
arCOG00693	COG01001	AdeC	F	Adenine deaminase
arCOG01046	COG00563	Adk	F	Adenylate kinase or related kinase
arCOG02013	COG00213	DeoA	F	Thymidine phosphorylase
arCOG03447	COG01457	CodB	F	Purine-cytosine permease or related protein
arCOG04128	COG00035	Upp	F	Uracil phosphoribosyltransferase
arCOG04320	COG00274	DeoC	F	Deoxyribose-phosphate aldolase
arCOG05252	COG04741	-	F	Predicted secreted endonuclease distantly related to archaeal Holliday junction resolvase
arCOG00271	COG00697	RhaT	G	Permease of the drug/metabolite transporter (DMT) superfamily
arCOG02682	COG00477	ProP	G	Permease of the major facilitator superfamily
arCOG00143	COG00477	ProP	G	Permease of the major facilitator superfamily
arCOG03285	COG03387	SGA1	G	Glycosyl hydrolase family 15
arCOG00016	COG00524	RbsK	G	Sugar kinase, ribokinase family
arCOG00018	COG00063	-	G	Predicted sugar kinase
arCOG00053	COG00269	SgbH	G	3-hexulose-6-phosphate synthase or related protein
arCOG00130	COG00477	ProP	G	Permease of the major facilitator superfamily
arCOG00131	COG00477	ProP	G	Permease of the major facilitator superfamily
arCOG00493	COG00057	GapA	G	Glyceraldehyde-3-phosphate dehydrogenase/erythrose-4-phosphate dehydrogenase
arCOG00496	COG00126	Pgk	G	3-phosphoglycerate kinase
arCOG00574	COG01635		G	Ribulose 1,5-bisphosphate synthetase, converts PRPP to RuBP, flavoprotein

arCOG01051	COG03958	-	G	Transketolase, C-terminal subunit
arCOG01053	COG03959	-	G	Transketolase, N-terminal subunit
arCOG01122	COG00120	RpiA	G	Ribose 5-phosphate isomerase
arCOG01349	COG00483	SuhB	G	Archaeal fructose-1,6-bisphosphatase or related enzyme of inositol monophosphatase family
arCOG04170	COG02379	GckA	G	Putative glycerate kinase
arCOG04221	COG00647	NagD	G	Phosphatase of the HAD superfamily
arCOG04435	COG01892	-	G	Phosphoenolpyruvate carboxylase
arCOG00135	COG00477	ProP	G	Permease of the major facilitator superfamily
arCOG00149	COG01653	UgpB	G	ABC-type sugar transport system, periplasmic component
arCOG00151	COG01653	UgpB	G	ABC-type sugar transport system, periplasmic component
arCOG00153	COG01653	UgpB	G	ABC-type trehalose transport system, periplasmic component
arCOG00175	COG03839	MalK	G	ABC-type sugar transport system, ATPase component
arCOG00180	COG03839	MalK	G	ABC-type sugar transport system, ATPase component
arCOG00272	COG00697	RhaT	G	permease of the drug/metabolite transporter (DMT) superfamily
arCOG01114	COG00574	PpsA	G	Phosphoenolpyruvate synthase/pyruvate phosphate dikinase
arCOG01393	COG01819	-	G	Glycosyl transferase, related to UDP-glucuronosyltransferase
arCOG01518	COG01363	FrvX	G	Cellulase M or related protein
arCOG01772	COG02407	FucI	G	L-fucose isomerase or related protein
arCOG02602	COG02140	-	G	Thermophilic glucose-6-phosphate isomerase or related metalloenzyme
arCOG02831	COG00380	OtsA	G	Trehalose-6-phosphate synthase
arCOG02876	COG00726	CDA1	G	Predicted xylanase/chitin deacetylase
arCOG02878	COG00726	CDA1	G	Predicted xylanase/chitin deacetylase
arCOG03003	COG00662		G	Protein containing cupin domain
arCOG03661	COG00383	AMS1	G	Alpha-mannosidase
arCOG04084	COG02152	-	G	Predicted glycosylase
arCOG04158			G	Mannosyl-3-phosphoglycerate synthase
arCOG04226	COG00235		G	Fuculose-1-phosphate aldolase
arCOG04443	COG01850	RbcL	G	Ribulose 1,5-bisphosphate carboxylase, large subunit
arCOG04974	COG03836	HpcH	G	2,4-dihydroxyhept-2-ene-1,7-dioic acid aldolase
arCOG05856	COG01874	LacA	G	Beta-galactosidase
arCOG06070	COG01486	CelF	G	Alpha-galactosidase/6-phospho-beta-glucosidase, family 4 of glycosyl hydrolase
arCOG07581	COG03325	ChiA	G	Chitinase
arCOG01939	COG00095	LplA	H	Lipoate-protein ligase A
arCOG00021	COG01992	-	H	Predicted transcriptional regulator fused phosphomethylpyrimidine kinase, involved in the thiamin biosynthesis
arCOG00216	COG00303	MoeA	H	Molybdopterin biosynthesis enzyme
arCOG00489	COG02096	-	H	cob(I)alamin adenosyltransferase
arCOG00536	COG01977	MoaD	H	Molybdopterin converting factor, small subunit
arCOG00613	COG01304	idi	H	Isopentenyl diphosphate isomerase
arCOG01671	COG00043	UbiD	H	3-polyprenyl-4-hydroxybenzoate decarboxylase or related decarboxylase
arCOG01940	COG00340	BirA	H	Biotin-(acetyl-CoA carboxylase) ligase
arCOG00020	COG00351	ThiD	H	Hydroxymethylpyrimidine/phosphomethylpyrimidine kinase
arCOG00034	COG00311	PDX2	H	Predicted glutamine amidotransferase involved in pyridoxine biosynthesis
arCOG00069	COG00171	NadE	H	NH3-dependent NAD+ synthetase
arCOG00105	COG01492	CobQ	H	Cobyric acid synthase
arCOG00106	COG01797	CobB	H	Cobyrinic acid a,c-diamide synthase
arCOG00214	COG00521	MoaB	H	Molybdopterin biosynthesis enzyme
arCOG00474	COG00212	-	H	5-formyltetrahydrofolate cyclo-ligase
arCOG00476	COG00382	UbiA	H	4-hydroxybenzoate polyprenyltransferase or related prenyltransferase
arCOG00532	COG01763	MobB	H	Molybdopterin-guanine dinucleotide biosynthesis protein

arCOG00534	COG00314	MoaE	H	Molybdopterin converting factor, large subunit
arCOG00584	COG00413	PanB	H	Ketopantoate hydroxymethyltransferase
arCOG00638	COG00611	ThiL	H	Thiamine monophosphate kinase
arCOG00644	COG00007	CysG	H	Uroporphyrinogen-III methylase
arCOG00650	COG02241	CobL	H	Precorrin-6B methylase 1
arCOG00918	COG00001	HemL	H	Glutamate-1-semialdehyde aminotransferase
arCOG00930	COG02896	MoaA	H	Molybdenum cofactor biosynthesis enzyme
arCOG01036	COG00373	HemA	H	Glutamyl-tRNA reductase
arCOG01044	COG01648	CysG	H	Siroheme synthase (precorrin-2 oxidase/ferrochelatase domain)
arCOG01045	COG00237	CoaE	H	Dephospho-CoA kinase
arCOG01320	COG00108	RibB	H	3,4-dihydroxy-2-butanone 4-phosphate synthase
arCOG01322	COG01731	-	H	Archaeal riboflavin synthase
arCOG01323	COG00054	RibH	H	Riboflavin synthase beta-chain
arCOG01348	COG00061	nadF	H	NAD kinase
arCOG01481	COG01488	PncB	H	Nicotinic acid phosphoribosyltransferase
arCOG01482	COG00157	NadC	H	Nicotinate-nucleotide pyrophosphorylase
arCOG01484	COG01985	RibD	H	Pyrimidine reductase, riboflavin biosynthesis
arCOG01530	COG00315	MoaC	H	Molybdenum cofactor biosynthesis enzyme
arCOG01703	COG00163	UbiX	H	3-polyprenyl-4-hydroxybenzoate decarboxylase
arCOG01704	COG00452	Dfp	H	Phosphopantothenoylcysteine synthetase/decarboxylase
arCOG01726	COG00142	IspA	H	Geranylgeranyl pyrophosphate synthase
arCOG01754	COG00111	SerA	H	Phosphoglycerate dehydrogenase or related dehydrogenase
arCOG01871	COG02266	-	H	GTP:adenosylcobinamide-phosphate guanylyltransferase
arCOG01872	COG00746	MobA	H	Molybdopterin-guanine dinucleotide biosynthesis protein A
arCOG01904	COG01339	-	H	CTP-dependent Riboflavin kinase
arCOG02048	COG01587	HemD	H	Uroporphyrinogen-III synthase
arCOG02172	COG00720	-	H	6-pyruvoyl-tetrahydropterin synthase
arCOG02199	COG00543	UbiB	H	2-polyprenylphenol hydroxylase or related flavodoxin oxidoreductase
arCOG02741	COG00422	ThiC	H	Thiamine biosynthesis protein ThiC
arCOG03838		PqqD	H	Coenzyme PQQ synthesis protein D
arCOG04075	COG00214	SNZ1	H	Pyridoxine biosynthesis enzyme
arCOG04137	COG00499	SAM1	H	S-adenosylhomocysteine hydrolase
arCOG04238	COG01767	CitG	H	Triphosphoribosyl-dephospho-CoA synthetase
arCOG04262	COG01701	-	H	Phosphopantothenate synthetase
arCOG04263	COG01829	-	H	Pantoate kinase
arCOG04272	COG02038	CobT	H	NaMN:DMB phosphoribosyltransferase
arCOG04274	COG01270	CbiB	H	Cobalamin biosynthesis protein CobD/CbiB
arCOG04299	COG00181	HemC	H	Porphobilinogen deaminase
arCOG04300	COG00113	HemB	H	Delta-aminolevulinic acid dehydratase
arCOG04338	COG00368	CobS	H	Cobalamin-5-phosphate synthase
arCOG04459	COG00379	NadA	H	Quinolinate synthase
arCOG04536	COG01402	-	H	Fe2+ dependent formamide hydrolase, cofactor Fo biosynthesis enzyme
arCOG00113	COG00156	BioF	H	7-keto-8-aminopelargonate synthetase or related enzyme
arCOG00166	COG04662	TupA	H	ABC-type tungstate transport system, periplasmic component
arCOG00217	COG00303	MoeA	H	Molybdopterin biosynthesis enzyme
arCOG00228	COG03585	MopI	H	Molybdopterin-binding protein
arCOG00229	COG02998	TupB	H	ABC-type tungstate transport system, permease component
arCOG00656	COG01060	ThiH	H	Thiamine biosynthesis enzyme ThiH, FO synthase or related uncharacterized enzyme
arCOG00954	COG02896	MoaA	H	Molybdenum cofactor biosynthesis enzyme
arCOG01089	COG00352	ThiE	H	Thiamine monophosphate synthase
arCOG01483	COG00157	NadC	H	Nicotinate-nucleotide pyrophosphorylase
arCOG01870	COG01865	cbiZ	H	Adenosylcobinamide amidohydrolase
arCOG02620	COG01541	PaaK	H	Coenzyme F390 synthetase

arCOG02714	COG01478	CofE	H	F(420)-0:gamma-glutamyl ligase, F420 coenzyme biosynthesis enzyme
arCOG02817	COG00285	FolC	H	Folylpolyglutamate synthase and Dihydropteroate synthase
arCOG04139	COG01893	ApbA	H	Ketopantoate reductase
arCOG04301	COG01469	MptA	H	Fe(2+)-dependent GTP cyclohydrolase
arCOG04336	COG01962	MtrH	H	Tetrahydromethanopterin S-methyltransferase, subunit H
arCOG04678	COG02109	BtuR	H	ATP:corrinoid adenosyltransferase
arCOG06472	COG01240		H	Mg-chelatase subunit ChlD
arCOG01529	COG00365	Acs	I	Acyl-coenzyme A synthetase/AMP-(fatty) acid ligase
arCOG01590	COG00439	AccC	I	Biotin carboxylase
arCOG02705	COG04799	-	I	Acetyl-CoA carboxylase, carboxyltransferase component
arCOG04213	COG01260	INO1	I	Myo-inositol-1-phosphate synthase
arCOG00247	COG02084	MmsB	I	3-hydroxyisobutyrate dehydrogenase or related beta-hydroxyacid dehydrogenase
arCOG00670	COG00558	PgsA	I	Phosphatidylglycerophosphate synthase
arCOG01028	COG01577	ERG12	I	Mevalonate kinase
arCOG01278	COG00183	PaaJ	I	Acetyl-CoA acetyltransferase
arCOG01532	COG00020	UppS	I	Undecaprenyl pyrophosphate synthase
arCOG01710	COG02185	Sbm	I	Methylmalonyl-CoA mutase, C-terminal domain/subunit (cobalamin-binding)
arCOG01767	COG03425	PksG	I	3-hydroxy-3-methylglutaryl CoA synthase
arCOG01879	COG00170		I	Dolichol kinase family protein
arCOG04106	COG00575	CdsA	I	CDP-diglyceride synthetase
arCOG04232	COG01884	Sbm	I	Methylmalonyl-CoA mutase
arCOG04260	COG01257	HMG1	I	Hydroxymethylglutaryl-CoA reductase
arCOG00674	COG00558	PgsA	I	Phosphatidylglycerophosphate synthase
arCOG00856	COG00318	CaiC	I	Acyl-CoA synthetase (AMP-forming)/AMP-acid ligase II
arCOG00860	COG01608	-	I	Isopentenyl phosphate kinase, enzyme of modified mevalonate pathway
arCOG01650	COG02267	PldB	I	Lysophospholipase, alpha-beta hydrolase superfamily
arCOG01842	COG03255	-	I	Putative sterol carrier protein
arCOG01869	COG01267	PgpA	I	Phosphatidylglycerophosphatase A fused to adenosylcobinamide amidohydrolase, CbiZ
arCOG01986	COG04670	-	I	Acyl CoA:acetate/3-ketoacid CoA transferase
arCOG02039	COG01502	Cls	I	Phosphatidylserine/phosphatidylglycerophosphate/cardiolipin synthase or related enzyme
arCOG02041	COG01502	Cls	I	Phosphatidylserine/phosphatidylglycerophosphate/cardiolipin synthase or related enzyme
arCOG02678	COG01924	-	I	Activator of 2-hydroxyglutaryl-CoA dehydratase (HSP70-class ATPase domain)
arCOG04438	COG01689	-	I	Class II terpene cyclase family protein
arCOG07147	COG01657	SqhC	I	Squalene cyclase
arCOG00975	COG00144	Sun	J	tRNA or rRNA cytosine-C5-methylase
arCOG01115	COG01571	TiaS	J	tRNA(Ile2) 2-agmatinylcytidine synthetase; containing Zn-ribbon domain and OB-fold domain
arCOG01630	COG00251	TdcF	J	Putative translation initiation inhibitor, yjgF family
arCOG01952	COG00009	SUA5	J	Putative translation factor (SUA5)
arCOG04307	COG01530	CafA	J	Ribonuclease G and E
arCOG00110	COG02813	RsmC	J	16S RNA G1207 methylase RsmC
arCOG00541	COG01236	YSH1	J	Predicted exonuclease of the beta-lactamase fold involved in RNA processing
arCOG00545	COG01236	YSH1	J	Predicted exonuclease of the beta-lactamase fold involved in RNA processing
arCOG00676	COG01096	-	J	Predicted RNA-binding protein (consists of S1 domain and a Zn-ribbon domain)
arCOG00986	COG00144	Sun	J	RNA methylase associated PUA domain
arCOG01015	COG01258	-	J	Predicted pseudouridylate synthase
arCOG01123	COG00182	-	J	Translation initiation factor eIF-2B alpha subunit
arCOG01354	COG02892	-	J	Subunit of KEOPS complex (Cgi121BUD32KAE1)

arCOG01564	COG03276	SelB	J	Selenocysteine-specific translation elongation factor or SelB-II domain
arCOG01616	COG01650	GEK1	J	D-aminoacyl-tRNA deacylase, involved in ethanol tolerance
arCOG02466	COG03277	GAR1	J	RNA-binding protein involved in rRNA processing
arCOG03247	COG02136	IMP4	J	Predicted exosome subunit/U3 small nucleolar ribonucleoprotein (snoRNP) component, contains IMP4 domain
arCOG04122	COG01756	Mra1	J	EMG1/NEP1 rRNA methyltransferase
arCOG04125	COG00430	RCL1	J	RNA 3'-terminal phosphate cyclase
arCOG04131	COG00030	KsgA	J	Dimethyladenosine transferase (rRNA methylation)
arCOG00547	COG00595	-	J	mRNA degradation ribonuclease J1/J2 (metallo-beta-lactamase superfamily)
arCOG00953	COG00731	TYW3	J	Homolog of Wybutosine (yW) biosynthesis enzyme, Fe-S oxidoreductase
arCOG01124	COG01184	GCD2	J	Translation initiation factor 2B subunit, eIF-2B alpha/beta/delta family
arCOG01125	COG01184	GCD2	J	Translation initiation factor 2B subunit, eIF-2B alpha/beta/delta family
arCOG02197	COG01617	Cgi121	J	Subunit of KEOPS complex, involved (Cgi121BUD32KAE1)
arCOG03218	COG04021	Thg1	J	tRNAHis guanylyltransferase
arCOG04063	COG01859	KptA	J	RNA:NAD 2'-phosphotransferase
arCOG04414	COG02892	Pcc1	J	Subunit of KEOPS complex (Cgi121BUD32KAE1)
arCOG00815	COG02002	AbrB	K	Transcriptional regulator AbrB
arCOG00492	COG01167	ARO8	K	Transcriptional regulators containing a DNA-binding HTH domain and an aminotransferase domain (MocR family)
arCOG00770	COG01199	DinG	K	Rad3-related DNA helicase
arCOG01055	COG03432	-	K	Predicted transcriptional regulator
arCOG02103			K	Transcriptional regulator, contains wHTH domain
arCOG03182	COG01846	MarR	K	Transcriptional regulator, MarR family
arCOG00002	COG01695	-	K	Predicted transcriptional regulator, PadR family
arCOG00610	COG02524	-	K	Predicted transcriptional regulator, contains C-terminal CBS domains
arCOG00724	COG01695		K	Predicted transcriptional regulator, PadR family
arCOG00812	COG02002	AbrB	K	Transcriptional regulator AbrB
arCOG00874	COG01061	xpb	K	ssDNA-dependent ATPase, helicase superfamily II
arCOG01446	COG00640	ArsR	K	Transcriptional regulator containing HTH domain, ArsR family
arCOG01679	COG00640	ArsR	K	Transcriptional regulator containing HTH domain, ArsR family
arCOG01761	COG00195	NusA	K	Transcription elongation factor
arCOG02099	COG01321	TroR	K	Mn-dependent transcriptional regulator (DtxR family)
arCOG02100	COG01321	TroR	K	Mn-dependent transcriptional regulator (DtxR family)
arCOG02271	COG03413	-	K	Predicted transcriptional regulator, C-terminal HTH-like domain
arCOG04060	COG01709	-	K	Predicted transcriptional regulator
arCOG04061	COG01308	EGD2	K	Transcription factor homologous to NACalpha-BTF3
arCOG04081	COG01224	TIP49	K	DNA helicase TIP49, TBP-interacting protein
arCOG04152	COG01395	-	K	Predicted transcriptional regulator
arCOG00001	COG01695	-	K	Predicted transcriptional regulator, PadR family
arCOG00374	COG02512	-	K	Predicted membrane-associated trancriptional regulator
arCOG00394	COG00640	ArsR	K	Transcriptional regulator containing HTH domain, ArsR family
arCOG00608	COG03620	-	K	Predicted transcriptional regulator with C-terminal CBS domains
arCOG00732	COG01846	MarR	K	Transcriptional regulator, MarR family
arCOG00818	COG02002	AbrB	K	Transcriptional regulator AbrB
arCOG00826	COG00454	WecD	K	Acetyltransferase (GNAT) family
arCOG00844	COG00454	WecD	K	Acetyltransferase (GNAT) family

arCOG00871	COG00553	HepA	K	Superfamily II DNA/RNA helicase, SNF2 family
arCOG01057	COG01733	-	K	Predicted transcriptional regulator
arCOG01361	COG01243	ELP3	K	Histone acetyltransferase
arCOG01665	COG02026	RelE	K	Cytotoxic translational repressor of toxin-antitoxin stability system
arCOG01680	COG00640	ArsR	K	Transcriptional regulator containing HTH domain, ArsR family
arCOG01681	COG00640	ArsR	K	Transcriptional regulator containing HTH domain, ArsR family
arCOG01683	COG00640	ArsR	K	Transcriptional regulator containing HTH domain, ArsR family
arCOG01684	COG01777	-	K	Predicted transcriptional regulator
arCOG01686	COG00640	ArsR	K	Transcriptional regulator containing HTH domain, ArsR family
arCOG01687	COG00640	ArsR	K	Transcriptional regulator containing HTH domain, ArsR family
arCOG01804	COG03888	-	K	Predicted transcriptional regulator
arCOG02038	COG01378	-	K	Sugar-specific transcriptional regulator TrmB
arCOG02611	COG03398	-	K	Predicted transcriptional regulator, containd two HTH domains
arCOG02613	COG01386	-	K	Chromosome segregation and condensation protein B
arCOG02795	COG01510	-	K	Predicted transcriptional regulator
arCOG03880	COG02880	-	K	DNA-binding protein, potential antitoxin AbrB/MazE fold
arCOG03936	COG02002	AbrB	K	Transcriptional regulator AbrB
arCOG04248	COG00846	SIR2	K	NAD-dependent protein deacetylase, SIR2 family
arCOG04280	COG01940	NagC	K	Transcriptional regulator/sugar kinase
arCOG04362	COG04742	-	K	Predicted transcriptional regulator
arCOG04518			K	Predicted transcriptional regulator, contains HTH domain
arCOG04554	COG01356	tfx	K	Transcriptional regulator
arCOG04818	COG00553	HepA	K	Superfamily II DNA/RNA helicase, SNF2 family
arCOG07152	COG02880	-	K	DNA-binding protein, potential antitoxin AbrB/MazE fold
arCOG07495	COG01497	-	K	Predicted transcriptional regulator
arCOG07561	COG02512	-	K	Predicted regulatory protein containing HTH domain
arCOG10049	COG02880		K	DNA-binding protein, potential antitoxin AbrB/MazE fold
arCOG04157	COG00270	Dcm	L	Site-specific DNA methylase
arCOG00679	COG00675	-	L	Transposase
arCOG03164	COG02452	-	L	Predicted site-specific integrase-resolvase
arCOG04298	COG01839	-	L	Predicted adenosine-specific kinase
arCOG00553	COG01204	-	L	Replicative SFII helicase
arCOG00792	COG01112	-	L	Superfamily I DNA/RNA helicase
arCOG04110	COG01467	PRI1	L	Eukaryotic-type DNA primase, catalytic (small) subunit
arCOG04357	COG01059	-	L	Thermostable 8-oxoguanine DNA glycosylase
arCOG00115	COG00863	-	L	DNA modification methylase
arCOG00426	COG00608	RecJ	L	DHH superfamily phosphohydrolase/exonuclease
arCOG00464	COG00122	AlkA	L	3-methyladenine DNA glycosylase/8-oxoguanine DNA glycosylase
arCOG00558	COG00513	SrmB	L	Superfamily II DNA and RNA helicase
arCOG00806	COG01112	-	L	Superfamily I DNA/RNA helicase fused to RecB family endonuclease
arCOG00890	COG00863	-	L	DNA modification methylase
arCOG01244	COG04342	-	L	Intergrase/Recombinase
arCOG01290	COG01533	SplB	L	DNA repair photolyase
arCOG01304	COG01637	NucS	L	RecB family endonuclease acting on branched DNA substrates
arCOG01442	COG02254	-	L	HD superfamily nuclease
arCOG02129	COG03335	-	L	Transposase
arCOG02610	COG01354	-	L	Rec8/ScpA/Scc1-like protein (kleisin family)
arCOG02636	COG00827	-	L	Adenine-specific DNA methylase
arCOG02759	COG01943	-	L	Transposase
arCOG03145	COG01372	-	L	Intein/homing endonuclease

arCOG03146	COG01372		L	Intein/homing endonuclease
arCOG03147	COG01372		L	Intein/homing endonuclease
arCOG03151	COG01372	-	L	Intein/homing endonuclease
arCOG03154	COG01372	-	L	Intein/homing endonuclease
arCOG03155	COG01241	MCM2	L	Cdc46/Mcm family protein (inactivated)
arCOG03156	COG03780	-	L	DNA endonuclease related to intein-encoded endonuclease
arCOG03162	COG01961	PinR	L	Site-specific recombinase, DNA invertase Pin homolog
arCOG03235	COG03593	-	L	Predicted ATP-dependent endonuclease of the OLD family
arCOG04218	COG01423	-	L	ATP-dependent RNA ligase
arCOG04306	COG04998	-	L	Predicted endonuclease (RecB family)
arCOG04356	COG03372	Bax1	L	Nuclease of restriction endonuclease-like fold, implicated in nucleotide excision repair
arCOG07683	COG01637	-	L	Predicted nuclease of the RecB family
arCOG00373	COG01196		L	DNA sulfur modification protein DndD, ATPase
arCOG00666	COG01208	GCD1	M	N-acetylglucosamine-1-phosphate uridyltransferase
arCOG00057	COG00449	GlmS	M	Glucosamine 6-phosphate synthetase
arCOG01369	COG00451	WcaG	M	Nucleoside-diphosphate-sugar epimerase
arCOG00068	COG00794	GutQ	M	Predicted sugar phosphate isomerase involved in capsule formation
arCOG00667	COG01209	RfbA	M	dTDP-glucose pyrophosphorylase
arCOG00895	COG00463	WcaA	M	Glycosyltransferase
arCOG01222	COG00615	TagD	M	Cytidylyltransferase fused to conserved domain of DUF357 family
arCOG01371	COG01088	RfbB	M	dTDP-D-glucose 4,6-dehydratase
arCOG01385	COG00463	WcaA	M	Glycosyltransferase
arCOG04400	COG01361	-	M	S-layer domain
arCOG00118	COG00399	WecE	M	Predicted pyridoxal phosphate-dependent enzyme apparently involved in regulation of cell wall biogenesis
arCOG00252	COG00677	WecC	M	UDP-N-acetyl-D-mannosaminuronate dehydrogenase
arCOG00253	COG01004	Ugd	M	Predicted UDP-glucose 6-dehydrogenase
arCOG00566	COG01807	ArnT	M	4-amino-4-deoxy-L-arabinose transferase or related glycosyltransferase of PMT family
arCOG00609	COG00750	-	M	Predicted membrane-associated Zn-dependent protease
arCOG00665	COG01210	GalU	M	UDP-glucose pyrophosphorylase
arCOG00673	COG01213	-	M	Predicted sugar nucleotidyltransferase
arCOG00896	COG00463	WcaA	M	Glycosyltransferase
arCOG01168	COG04948	-	M	L-alanine-DL-glutamate epimerase or related enzyme of enolase superfamily
arCOG01376	COG00451	WcaG	M	Nucleoside-diphosphate-sugar epimerase
arCOG01386	COG01215	-	M	Glycosyltransferase
arCOG01392	COG00381	WecB	M	UDP-N-acetylglucosamine 2-epimerase
arCOG01405	COG00438	RfaG	M	Glycosyltransferase
arCOG01407	COG00438	RfaG	M	Glycosyltransferase
arCOG01408	COG00438	RfaG	M	Glycosyltransferase
arCOG01409	COG00438	RfaG	M	Glycosyltransferase
arCOG02427	COG00836	{ManC}	M	Mannose-1-phosphate guanylyltransferase fused to cupin domain
arCOG04468	COG00451	WcaG	M	Nucleoside-diphosphate-sugar epimerase
arCOG04827	COG01887	TagB	M	Putative glycosyl/glycerophosphate transferase involved in teichoic acid biosynthesis TagF/TagB/EpsJ/RodC
arCOG02298	COG01989	PulO	N	Peptidase A24A, prepilin type IV
arCOG04921	COG01352	CheR	N	Methylase of chemotaxis methyl-accepting protein, contains Pas and CheB domains
arCOG02062	COG00425	SirA	O	Predicted redox protein, regulator of disulfide bond formation
arCOG00479	COG00109	CyoE	O	Polyprenyltransferase (cytochrome oxidase assembly factor)
arCOG00976	COG02518	Pcm	O	Protein-L-isoaspartate carboxylmethyltransferase
arCOG01972	COG00526	TrxA	O	Thiol-disulfide isomerase or thioredoxin
arCOG02007	COG01331	-	O	Highly conserved protein containing a thioredoxin domain

arCOG00065	COG00520	csdA	O	Selenocysteine lyase/Cysteine desulfurase
arCOG00164	COG00555	CysU	O	ABC-type sulfate transport system, permease component
arCOG00310	COG01225	Bcp	O	Peroxiredoxin
arCOG00636	COG00309	HypE	O	Hydrogenase maturation factor
arCOG01187	COG00068	HypF	O	Hydrogenase maturation factor
arCOG01331	COG00501	HtpX	O	Zn-dependent protease with chaperone function
arCOG01833	COG00071	IbpA	O	Molecular chaperone (HSP20 family)
arCOG02173	COG00602	NrdG	O	Organic radical activating enzyme
arCOG03686	COG01765	-	O	Predicted redox protein, regulator of disulfide bond formation
arCOG04427	COG00298	HypC	O	Hydrogenase maturation factor
arCOG04428	COG00409	HypD	O	Hydrogenase maturation factor
arCOG00702	COG01404	AprE	O	Subtilisin-like serine protease
arCOG00936	COG01180	PflA	O	Pyruvate-formate lyase-activating enzyme
arCOG00947	COG01180	PflA	O	Pyruvate-formate lyase-activating enzyme
arCOG01333	COG00501	HtpX	O	Zn-dependent protease with chaperone function
arCOG01334	COG00501	HtpX	O	Zn-dependent protease with chaperone function
arCOG01910	COG01030	NfeD	O	Short-chain alcohol dehydrogenase
arCOG01911	COG00616	SppA	O	Periplasmic serine protease (ClpP class)
arCOG01912	COG01585	-	O	Membrane protein implicated in regulation of membrane protease activity
arCOG02163	COG04930	-	O	Predicted ATP-dependent Lon-type protease
arCOG02833	COG00265	DegQ	O	Serine protease Do (heat-shock protein)
arCOG04933	COG04826	-	O	Serine protease inhibitor
arCOG06880	COG00484	DnaJ	O	DnaJ type Zn finger domain
arCOG00318	COG00704	PhoU	P	Phosphate uptake regulator
arCOG00167	COG00573	PstC	P	ABC-type phosphate transport system, permease component
arCOG00168	COG00581	PstA	P	ABC-type phosphate transport system, permease component
arCOG00193	COG01116	TauB	P	ABC-type nitrate/sulfonate/bicarbonate transport system, ATPase component
arCOG00213	COG00226	PstS	P	ABC-type phosphate transport system, periplasmic component
arCOG01576	COG02217	ZntA	P	Cation transport ATPase
arCOG01868	COG00735	Fur	P	Fe2+/Zn2+ uptake regulation protein, fur/PerR
arCOG02640	COG01392	-	P	Phosphate transport regulator (distant homolog of PhoU)
arCOG04233	COG00614	FepB	P	ABC-type Fe3+-hydroxamate transport system, periplasmic component
arCOG04531	COG01914	MntH	P	Mn2+ and Fe2+ transporter of the NRAMP family
arCOG00198	COG01120	FepC	P	ABC-type cobalamin/Fe3+-siderophores transport system, ATPase component
arCOG00219	COG00725	ModA	P	ABC-type molybdate transport system, periplasmic component
arCOG00231	COG01117	PstB	P	ABC-type phosphate transport system, ATPase component
arCOG00238	COG01055	ArsB	P	Na+/H+ antiporter NhaD or related arsenite permease
arCOG01007	COG00609	FepD	P	ABC-type Fe3+-siderophore transport system, permease component
arCOG01069	COG00446		P	CoA-dependent NAD(P)H Sulfur Oxidoreductase
arCOG01953	COG00475	KefB	P	Kef-type K+ transport system, membrane component
arCOG02021	COG00607	PspE	P	Rhodanese-related sulfurtransferase
arCOG02267	COG00306	PitA	P	Phosphate/sulphate permease
arCOG02763	COG02217		P	Heavy-metal-associated domain (HMA)
arCOG02852	COG02146	{NirD}	P	Ferredoxin subunit of nitrite reductase or ring-hydroxylating dioxygenase
arCOG04397	COG00004	AmtB	P	Ammonia permease
arCOG00163	COG01178	ThiP	P	ABC-type Fe3+ transport system, permease component
arCOG00169	COG00600	TauC	P	ABC-type nitrate/sulfonate/bicarbonate transport system, permease component

arCOG00188	COG01122	CbiO	P	ABC-type cobalt transport system, ATPase component
arCOG00201	COG01121	ZnuC	P	ABC-type Mn/Zn transport system, ATPase component
arCOG00202	COG01122	CbiO	P	ABC-type cobalt transport system, ATPase component
arCOG00230	COG01910	-	P	Periplasmic molybdate-binding protein/domain
arCOG00232	COG00704	PhoU	P	Phosphate uptake regulator
arCOG00237	COG00471	CitT	P	Di- and tricarboxylate transporter
arCOG00359	COG00370	FeoB	P	Fe2+ transport system protein B
arCOG00576	COG00428	-	P	Predicted divalent heavy-metal cations transporter
arCOG00624	COG01824	-	P	Permease, similar to cation transporter
arCOG00625	COG02239	MgtE	P	Mg/Co/Ni transporter MgtE (contains CBS domain)
arCOG01005	COG00803	LraI	P	ABC-type metal ion transport system, periplasmic component/surface adhesin
arCOG01006	COG01108	ZnuB	P	ABC-type Mn2+/Zn2+ transport system, permease component
arCOG01040	COG00529	CysC	P	Adenylylsulfate kinase or related kinase
arCOG01094	COG02193	Bfr	P	Bacterioferritin (cytochrome b1)
arCOG01095	COG01528	Ftn	P	Ferritin
arCOG01474	COG00053	MMT1	P	Predicted Co/Zn/Cd cation transporter
arCOG01477	COG01230	CzcD	P	Co/Zn/Cd efflux system component
arCOG01585	COG01522	Lrp	P	Transcriptional regulator (Lrp/AsnC family)
arCOG01803	COG00715	TauA	P	ABC-type nitrate/sulfonate/bicarbonate transport system, periplasmic component
arCOG01957	COG00569	TrkA	P	K+ transport system, NAD-binding component
arCOG01958	COG01226	Kch	P	Kef-type K+ transport system, predicted NAD binding component
arCOG02102	COG01918	FeoA	P	Fe2+ transport system protein A
arCOG02190	COG00798	ACR3	P	Arsenite efflux pump ACR3 or related permease
arCOG02248	COG00310	CbiM	P	ABC-type Co2+ transport system, permease component
arCOG02250	COG00619	CbiQ	P	ABC-type cobalt transport system, permease component CbiQ or related transporter
arCOG02569	COG00038	EriC	P	Chloride channel protein EriC
arCOG02764	COG02608	CopZ	P	Copper-ion-binding protein
arCOG02849	COG00003	ArsA	P	Oxyanion-translocating ATPase
arCOG03072	COG01006	MnhC	P	Multisubunit Na+/H+ antiporter, MnhC subunit
arCOG03076	COG01563	-	P	Predicted subunit of the Multisubunit Na+/H+ antiporter
arCOG03077	COG02111	MnhB	P	Multisubunit Na+/H+ antiporter, MnhB subunit
arCOG03078	COG01563	-	P	Predicted subunit of the Multisubunit Na+/H+ antiporter
arCOG03079	COG02111	MnhB	P	Multisubunit Na+/H+ antiporter, MnhB subunit
arCOG03082	COG01320	MnhG	P	Multisubunit Na+/H+ antiporter, MnhG subunit
arCOG03099	COG01863	MnhE	P	Multisubunit Na+/H+ antiporter, MnhE subunit
arCOG03127	COG01226	Kch	P	Ion channel fused to ion transporting domain
arCOG03159	COG00310	CbiM	P	ABC-type Co2+ transport system, permease component
arCOG03303	COG00614	FepB	P	ABC-type Fe3+-hydroxamate transport system, periplasmic component
arCOG03454	COG02116	FocA	P	Formate/nitrite family of transporter
arCOG04145	COG00168	TrkG	P	Trk-type K+ transport system, membrane component
arCOG04191	COG02046	MET3	P	ATP sulfurylase (sulfate adenylyltransferase)
arCOG04330	COG00672	FTR1	P	High-affinity Fe2+/Pb2+ permease
arCOG05758			P	TrkA-C domain contaning protein
arCOG00777	COG02050	PaaI	Q	HGG motif-containing thioesterase, possibly involved in aromatic compounds catabolism

arCOG01521	COG01233	-	Q	Phytoene dehydrogenase or related enzyme
arCOG04507	COG03350	-	Q	Methane/Phenol/Toluene Hydroxylase component
arCOG00235	COG00179	MhpD	Q	2-keto-4-pentenoate hydratase/2-oxohepta-3-ene-1,7-dioic acid hydratase (catechol pathway)
arCOG01773	COG00500		Q	SAM-dependent methyltransferase
arCOG01791	COG00500		Q	SAM-dependent methyltransferase
arCOG02702	COG00500		Q	SAM-dependent methyltransferase
arCOG00696	COG01228	HutI	Q	Imidazolonepropionase or related amidohydrolase
arCOG01402	COG00500		Q	SAM-dependent methyltransferase
arCOG01790	COG00500		Q	SAM-dependent methyltransferase
arCOG00600	COG00517	-	R	CBS domain
arCOG03169	COG01672	-	R	Predicted ATPase (AAA+ superfamily)
arCOG00662	COG02516	-	R	Biotin synthase-related enzyme
arCOG03682	COG00515	SPS1	R	Membrane associated serine/threonine protein kinase
arCOG00039	COG00603	-	R	Predicted PP-loop superfamily ATPase
arCOG00503	COG01237	-	R	Metal-dependent hydrolase of the beta-lactamase superfamily II
arCOG01350	COG03199	-	R	Predicted inorganic polyphosphate/ATP-NAD kinase
arCOG03239	COG01106	-	R	ATPase, predicted component of phage defense system
arCOG05825	COG01856	-	R	Radical SAM superfamily enzyme
arCOG00040	COG02236	-	R	Predicted phosphoribosyltransferase
arCOG00062	COG00388	-	R	Predicted amidohydrolase
arCOG00321	COG00312	TldD	R	Predicted Zn-dependent protease or their inactivated homolog
arCOG00322	COG00312	TldD	R	Predicted Zn-dependent protease or their inactivated homolog
arCOG00347	COG01938	-	R	Archaeal enzyme of ATP-grasp superfamily
arCOG00833	COG00456	RimI	R	Acetyltransferase (GNAT) family
arCOG00940	COG00535	-	R	Radical SAM superfamily enzyme
arCOG00968	COG02428	-	R	Alpha/beta-knot SAM-dependent RNA methyltransferase
arCOG01085	COG01646	-	R	Predicted phosphate-binding enzyme, TIM-barrel fold
arCOG01213	COG00561	Cof	R	HAD superfamily hydrolase
arCOG01492	COG03383	-	R	Uncharacterized anaerobic dehydrogenase
arCOG01648	COG00596	MhpC	R	Alpha/beta superfamily hydrolase
arCOG02231	COG02509	-	R	Uncharacterized FAD-dependent dehydrogenase
arCOG03639	COG00121	-	R	Predicted glutamine amidotransferase
arCOG04115	COG01489	SfsA	R	DNA-binding protein, stimulates sugar fermentation
arCOG04227	COG01832	-	R	Predicted CoA-binding protein
arCOG07416	COG00596	MhpC	R	Alpha/beta superfamily hydrolase
arCOG00084	COG01818	-	R	Predicted RNA-binding protein, contains THUMP domain
arCOG00215	COG01058	CinA	R	Predicted nucleotide-utilizing enzyme related to molybdopterin-biosynthesis enzyme MoeA
arCOG00223	COG02005	ModE	R	N-terminal domain of molybdenum-binding protein
arCOG00302	COG00613	-	R	Metal-dependent phosphoesterase (PHP family)
arCOG00353	COG02262	HflX	R	GTP-binding protein protease modulator
arCOG00436	COG00714	-	R	MoxR-like ATPase
arCOG00442	COG02425	-	R	Uncharacterized protein containing a von Willebrand factor type A (vWA) domain
arCOG00500	COG01234	ElaC	R	Metal-dependent hydrolase of the beta-lactamase superfamily
arCOG00504	COG00491	GloB	R	Metal-dependent hydrolase of the beta-lactamase superfamily II
arCOG00578	COG01645	-	R	Uncharacterized Zn-finger containing protein
arCOG00932	COG02108	-	R	Uncharacterized conserved protein related to pyruvate formate-lyase activating enzyme
arCOG00933	COG01964	-	R	Radical SAM superfamily enzyme

arCOG01019	COG04080	-	R	SpoU rRNA Methylase family enzyme
arCOG01026	COG01907	-	R	Predicted sugar kinase
arCOG01043	COG01931	-	R	Predicted RNA binding protein with dsRBD fold
arCOG01084	COG01694	MazG	R	Predicted pyrophosphatase
arCOG01138	COG01310	-	R	Predicted metal-dependent protease of the PAD1/JAB1 superfamily
arCOG01225	COG01100	-	R	GTPase SAR1 or related small G protein
arCOG01285	COG01545	-	R	Predicted nucleic-acid-binding protein containing a Zn-ribbon
arCOG01377	COG03379	-	R	Phosphodiesterase of AP superfamily
arCOG01432	COG01350	-	R	Tryptophan synthase beta-subunit
arCOG01562	COG05258	GTPBP1	R	GTPase
arCOG01619	COG00656	ARA1	R	Aldo/keto reductase, related to diketogulonate reductase
arCOG01672	COG03889	-	R	Predicted solute binding protein
arCOG01831	COG01665	-	R	Predicted nucleotidyltransferase
arCOG01849	COG00663	PaaY	R	Isoleucine patch superfamily protein
arCOG01857	COG01303	-	R	SpoU rRNA Methylase family enzyme
arCOG02064	COG02210	-	R	Peroxiredoxin family protein
arCOG02291	COG01011	-	R	HAD superfamily hydrolase
arCOG02727	COG01673	-	R	Predicted RNA-binding protein containing PUA-domain
arCOG03042	COG00457	NrfG	R	TPR repeats containing protein
arCOG03233	COG04938	-	R	Predicted ATPase
arCOG04066	COG02413	-	R	Minimal nucleotidyltransferase
arCOG04127	COG01341	-	R	Predicted GTPase or GTP-binding protein
arCOG04151	COG02237	-	R	ABC-type multidrug transport system, permease component
arCOG04250	COG03382	-	R	Solo B3/4 domain (OB-fold DNA/RNA-binding) of Phe-aaRS-beta
arCOG04290	COG01656	-	R	PIN-domain and Zn ribbon
arCOG04303	COG01634	-	R	Uncharacterized Rossmann fold enzyme
arCOG04311	COG01896	-	R	Predicted hydrolase of HD superfamily
arCOG04426	COG00375	HybF	R	Zn finger protein HypA/HybF (possibly regulating hydrogenase expression)
arCOG04458	COG02457	-	R	Uncharacterized protein of DIM6/NTAB family
arCOG00036	COG02102	-	R	Predicted ATPase of PP-loop superfamily
arCOG00043	COG01606	-	R	ATP-utilizing enzyme of the PP-loop superfamily
arCOG00054	COG02521	-	R	Predicted archaeal methyltransferase
arCOG00056	COG01818	-	R	Predicted RNA-binding protein, contains THUMP domain
arCOG00185	COG01123	-	R	ATPase component of various ABC-type transport system, contain duplicated ATPase
arCOG00260	COG04603	-	R	ABC-type uncharacterized transport system, permease component
arCOG00261	COG01079	-	R	Uncharacterized ABC-type transport system, permease component
arCOG00266	COG02041	-	R	Sulfite oxidase or related enzyme
arCOG00354	COG01100	-	R	GTPase SAR1 or related small G protein
arCOG00362	COG02229	-	R	Predicted GTPase
arCOG00441	COG00714	-	R	MoxR-like ATPase
arCOG00498	COG00491	GloB	R	Metal-dependent hydrolase of the beta-lactamase superfamily II
arCOG00606	COG00517	-	R	CBS domain
arCOG00691	COG01574	-	R	Predicted metal-dependent hydrolase with the TIM-barrel fold
arCOG00754	COG00579	-	R	Malate/quinone oxidoreductase or related dehydrogenase
arCOG00887	COG01483	-	R	Predicted ATPase (AAA+ superfamily)
arCOG00888	COG01483	-	R	Predicted ATPase (AAA+ superfamily)
arCOG00893	COG01831	-	R	Predicted metal-dependent hydrolase (urease superfamily)
arCOG00951	COG02100	-	R	Radical SAM superfamily enzyme
arCOG01093	COG02406	-	R	Protein distantly related to bacterial ferritins
arCOG01108	COG01473	AbgB	R	Metal-dependent amidase/aminoacylase/carboxypeptidase

arCOG01139	COG01310	-	R	Predicted metal-dependent protease of the PAD1/JAB1 superfamily
arCOG01141	COG00622	-	R	Phosphoesterase
arCOG01153	COG01409	Icc	R	Predicted phosphohydrolase
arCOG01156	COG01408	-	R	Calcineurin-like phosphohydrolase
arCOG01195	COG01708	-	R	Minimal nucleotidyltransferase
arCOG01201	COG01708	-	R	Minimal nucleotidyltransferase
arCOG01202	COG01708	-	R	Minimal nucleotidyltransferase
arCOG01208	COG01708	-	R	Minimal nucleotidyltransferase
arCOG01211	COG01708	-	R	Minimal nucleotidyltransferase
arCOG01215	COG03769	-	R	HAD superfamily hydrolase
arCOG01223	COG01019	-	R	Predicted nucleotidyltransferase
arCOG01229	COG02403	-	R	Predicted GTPase
arCOG01383	COG01216	-	R	Predicted glycosyltransferase
arCOG01395	COG01817	-	R	Lipid-A-disaccharide synthase related glycosyltransferase
arCOG01425			R	RecB family nuclease with coiled-coil N-terminal domain
arCOG01565	COG00618	-	R	Exopolyphosphatase-related protein
arCOG01622	COG00673	MviM	R	Predicted dehydrogenase
arCOG01801	COG02358	Imp	R	TRAP-type uncharacterized transport system, periplasmic component
arCOG01806			R	ACT domain containing protein
arCOG01848	COG00110	WbbJ	R	Acetyltransferase (isoleucine patch superfamily)
arCOG01860	COG01418	-	R	Predicted HD superfamily hydrolase
arCOG01906	COG04666	-	R	TRAP-type uncharacterized transport system, fused permease component
arCOG01931	COG02159	-	R	Predicted metal-dependent hydrolase of the TIM-barrel fold
arCOG01963	COG03273	-	R	PhoU-like domain fused to TrkA-C domain
arCOG01982	COG00434	SgcQ	R	Predicted TIM-barrel enzyme
arCOG02016	COG01853	-	R	Conserved protein/domain typically associated with flavoprotein oxygenase, DIM6/NTAB family
arCOG02017	COG01853	-	R	Conserved protein/domain typically associated with flavoprotein oxygenase, DIM6/NTAB family
arCOG02028	COG05012	-	R	Predicted cobalamin binding protein
arCOG02035	COG03894	-	R	Uncharacterized metal-binding protein
arCOG02105	COG01708	-	R	Minimal nucleotidyltransferase
arCOG02107	COG01708	-	R	Minimal nucleotidyltransferase
arCOG02238	COG01342	-	R	Predicted DNA-binding protein
arCOG02240	COG01942	-	R	4-oxalocrotonate tautomerase related protein
arCOG02258	COG03390	-	R	RPA family protein, a subunit of RPA complex in P.uriosus
arCOG02284	COG04880	-	R	Secreted protein containing C-terminal beta-propeller domain distantly related to WD-40 repeats
arCOG02303	COG00496	SurE	R	Predicted acid phosphatase
arCOG02412	COG01598	-	R	Predicted nuclease of the RNAse H fold, HicB family
arCOG02431	COG01611	-	R	Predicted Rossmann fold nucleotide-binding protein
arCOG02465	COG01691	-	R	NCAIR mutase (PurE)-related protein
arCOG02579	COG00727	-	R	Predicted Fe-S-cluster oxidoreductase
arCOG02616	COG00535	-	R	Radical SAM superfamily enzyme
arCOG02767	COG01266	-	R	Metal-dependent membrane protease, CAAX family
arCOG02768	COG01266	-	R	Metal-dependent membrane protease, CAAX family
arCOG02828	COG03367	-	R	NAD dependent epimerase/dehydratase family
arCOG02902	COG02304	-	R	Uncharacterized protein containing a von Willebrand factor type A (vWA) domain
arCOG02959	COG02234	Iap	R	Predicted aminopeptidase
arCOG02986	COG01268	BioY	R	Uncharacterized conserved protein
arCOG02999	COG01917	-	R	Cupin domain containing protein
arCOG03045	COG00457	NrfG	R	TPR repeats containing protein
arCOG03086	COG01724	-	R	Predicted RNA binding protein (dsRBD-like fold), HicA family

arCOG03241	COG04637	-	R	Predicted ATPase
arCOG03509	COG03291	-	R	Cell suface protein, containing PKD repeats
arCOG03647	COG02388	-	R	Acetyltransferase (GNAT) family
arCOG03850	COG04122		R	Predicted O-methyltransferase
arCOG04004	COG00666	Arp	R	Ankyrin repeats containing protein
arCOG04052	COG04756	-	R	Predicted cation transporter
arCOG04197			R	Transposase related protein
arCOG04212	COG01661	-	R	Predicted DNA-binding protein with PD1-like DNA-binding motif
arCOG04225	COG02110	-	R	Predicted phosphatase homologous to the C-terminal domain of histone macroH2A1
arCOG04230	COG03294	-	R	HD supefamily hydrolase
arCOG04278	COG01679	-	R	Predicted aconitase
arCOG04297	COG02401	-	R	ABC-type ATPase fused to a predicted acetyltransferase domain
arCOG04359	COG02517	-	R	Predicted RNA-binding protein containing a C-terminal EMAP domain
arCOG04410	COG01578	-	R	Predicted ATP-grasp domain fused to redox center
arCOG04463	COG03389	-	R	Presenilin-like membrane protease, A22 family
arCOG04756	COG00679	-	R	Predicted permease
arCOG05120	COG05014	-	R	Radical SAM superfamily enzyme
arCOG05346			R	PP-loop superfamily ATPase fused to Zn-finger domain
arCOG05613			R	Predicted DEAD-box helicase/ATPase
arCOG05741			R	Nucleic acid binding, OB-fold
arCOG06126	COG01811	-	R	Uncharacterized membrane protein, possible Na+ channel or pump
arCOG06897	COG03919	-	R	Predicted ATP-grasp enzyme
arCOG07501	COG01994	SpoIVFB	R	Zn-dependent protease
arCOG07614	COG00121	-	R	Predicted glutamine amidotransferase
arCOG07790			R	D-glucuronyl C5-epimerase C-terminal domain related protein
arCOG07957	COG02342	-	R	Uncharacterized TIM barrel protein
arCOG07960	COG02607	-	R	ATPase (AAA+ superfamily)
arCOG08578			R	P-loop NTPase
arCOG04321	COG02848	-	S	Uncharacterized conserved protein with similarity to Ribonucleotide reductase and Pyruvate formate lyase
arCOG01096	COG01814	-	S	Ferritin-like domain containing protein
arCOG01091	COG01814	-	S	Uncharacterized membrane protein
arCOG04733	COG02042	-	S	Uncharacterized conserved protein, contains Fer4-like metal-binding domain ortholog of eukaryotic RNase L inhibitor RLI N-terminal domain
arCOG01302	COG01531	-	S	Uncharacterized conserved protein
arCOG01907	COG02013	-	S	Uncharacterized conserved protein
arCOG01921	COG02083	-	S	Uncharacterized conserved protein
arCOG02159	COG04089	-	S	Predicted membrane protein
arCOG02734	COG01433	-	S	NifX family protein
arCOG02994	COG01917	-	S	Cupin domain containing protein
arCOG03117	COG00586	DedA	S	Uncharacterized membrane-associated protein, DedA family
arCOG03142			S	Uncharacterized conserved protein
arCOG03216	COG02014	-	S	Uncharacterized conserved protein
arCOG03229	COG02454	-	S	Uncharacterized conserved protein
arCOG04076	COG01909	-	S	Uncharacterized conserved protein
arCOG04124	COG02835	-	S	Uncharacterized conserved protein
arCOG04140	COG01888	-	S	Uncharacterized conserved protein
arCOG04308	COG01698	-	S	Uncharacterized conserved protein
arCOG04351	COG03356	-	S	Predicted membrane protein
arCOG04373	COG00011	-	S	Uncharacterized conserved protein
arCOG05978			S	Extracellular protein containing Kelch and FN3 domains
arCOG06027	COG03189	-	S	Uncharacterized conserved protein
arCOG07088			S	Uncharacterized conserved protein
arCOG00369	COG04717	-	S	Uncharacterized conserved protein
arCOG00372			S	Uncharacterized conserved protein
arCOG00723	COG05483	-	S	Uncharacterized conserved protein
arCOG00761	COG01720	-	S	Uncharacterized conserved protein

arCOG00899	COG00392	-	S	Uncharacterized conserved membrane protein
arCOG00908	COG03286	-	S	Uncharacterized conserved protein
arCOG01119	COG04274	-	S	GYD domain, alpha/beta barrel superfamily
arCOG01314			S	Uncharacterized conserved membrane protein
arCOG01471	COG03945	-	S	Hemerythrin HHE cation binding domain containing protein
arCOG01472	COG02461	-	S	Hemerythrin HHE cation binding domain containing protein
arCOG01668	COG03370	-	S	Uncharacterized conserved protein
arCOG01724	COG04072	-	S	Uncharacterized conserved protein
arCOG01734	COG04933	-	S	Uncharacterized conserved protein
arCOG01917			S	Zn-ribbon domain containing protein
arCOG01996	COG01300	SpoIIM	S	Uncharacterized membrane protein
arCOG02008	COG03371	-	S	Predicted membrane protein
arCOG02024	COG04881	-	S	Predicted membrane protein
arCOG02078	COG01852	-	S	Uncharacterized conserved protein
arCOG02081	COG01470	-	S	Predicted membrane protein
arCOG02087	COG01470	-	S	Predicted membrane protein
arCOG02108	COG02445	-	S	HEPN domain containing protein
arCOG02109	COG02445	-	S	HEPN domain containing protein
arCOG02245	COG01836	-	S	Predicted membrane protein
arCOG02264	COG01808	-	S	Predicted membrane protein
arCOG02270	COG05428	-	S	Uncharacterized conserved small protein
arCOG02287	COG00393	-	S	Uncharacterized beta/alpha-propeller protein, likely forms oligomers
arCOG02396			S	Uncharacterized conserved protein
arCOG02408	COG01432	-	S	Uncharacterized conserved protein
arCOG02487			S	Cell suface protein
arCOG02488			S	Cell suface protein
arCOG02491	COG02319	-	S	WD40 repeats containing protein
arCOG02497	COG03420		S	S-layer protein
arCOG02508	COG03291	-	S	PKD repeats containing protein
arCOG02510	COG03291	-	S	PKD repeats containing protein
arCOG02532	COG03291	-	S	beta-propeller repeat protein fused to CARDB-like adhesion domain
arCOG02540	COG03291	-	S	PKD repeats containing protein
arCOG02545	COG03291		S	NosD-like periplasmic protein fused to PKD repeat domain
arCOG02556	COG01520	-	S	WD40-like repeat
arCOG02559			S	Cell suface protein
arCOG02701	COG01641	-	S	Uncharacterized conserved protein
arCOG02835	COG02324	-	S	Uncharacterized conserved membrane protein
arCOG02879			S	Uncharacterized conserved protein
arCOG02884	COG04743	-	S	Predicted membrane protein
arCOG03005	COG04997	-	S	Uncharacterized conserved protein, contains double-stranded beta-helix domain
arCOG03188			S	Uncharacterized conserved protein
arCOG03206	COG01822	-	S	Predicted archaeal membrane protein
arCOG03270			S	Uncharacterized conserved protein, contains DUF11 domain
arCOG03333	COG04273	-	S	DGC domain implicated in zinc binding
arCOG03414			S	Uncharacterized conserved protein
arCOG03427	COG02510	-	S	Predicted membrane protein
arCOG03442			S	Uncharacterized conserved membrane protein
arCOG03460	COG02120	-	S	Uncharacterized protein, LmbE homolog
arCOG03511	COG05306	-	S	Uncharacterized conserved protein
arCOG03633	COG01714	-	S	Predicted membrane protein/domain
arCOG03754			S	Uncharacterized conserved protein
arCOG03794	COG03601	-	S	Predicted membrane protein
arCOG03829			S	Uncharacterized conserved protein
arCOG03835			S	Uncharacterized conserved membrane protein
arCOG03841	COG03535	-	S	Uncharacterized conserved protein
arCOG03911	COG03462	-	S	Predicted membrane protein
arCOG03942			S	Uncharacterized conserved protein
arCOG03957	COG04902	-	S	Uncharacterized conserved protein

arCOG04002			S	Uncharacterized conserved protein
arCOG04024			S	Uncharacterized conserved protein
arCOG04025			S	Uncharacterized conserved protein
arCOG04039			S	Uncharacterized conserved protein
arCOG04058	COG01872	-	S	Uncharacterized conserved protein
arCOG04317	COG04732	-	S	Predicted membrane protein
arCOG04337	COG01504	-	S	Uncharacterized conserved protein
arCOG04343	COG01751	-	S	Uncharacterized conserved protein
arCOG04344	COG04818	-	S	Predicted membrane protein
arCOG04354	COG01906	-	S	Uncharacterized conserved protein
arCOG04394	COG01416	-	S	Uncharacterized conserved protein
arCOG04437	COG03863	-	S	Uncharacterized distant relative of cell wall-associated hydrolase
arCOG04467			S	Uncharacterized conserved protein
arCOG04477	COG01860	-	S	Uncharacterized conserved protein
arCOG04483	COG04739	-	S	Uncharacterized protein containing a ferredoxin domain
arCOG04484	COG05658	-	S	Predicted integral membrane protein
arCOG04488	COG02164	-	S	Uncharacterized conserved protein
arCOG04492	COG02028	-	S	Uncharacterized conserved protein
arCOG04500			S	Uncharacterized conserved protein
arCOG04609			S	Predicted lipoprotein
arCOG04612	COG04044	-	S	Uncharacterized conserved protein
arCOG04705	COG02098	-	S	Uncharacterized conserved protein
arCOG04797			S	Uncharacterized conserved protein
arCOG04807	COG01297	-	S	Predicted membrane protein
arCOG04811	COG02383	-	S	Uncharacterized conserved membrane protein, Fun14 family
arCOG04849			S	Uncharacterized conserved protein
arCOG04883			S	Uncharacterized conserved protein
arCOG04893	COG04022	-	S	Uncharacterized conserved protein
arCOG04938			S	Uncharacterized conserved protein
arCOG05048			S	Uncharacterized conserved protein
arCOG05079	COG01542	-	S	Uncharacterized conserved protein
arCOG05763	COG04003	-	S	Uncharacterized conserved protein
arCOG05783			S	Uncharacterized conserved protein
arCOG05791			S	Uncharacterized cell suface protein, containing DUF11 domain
arCOG05908	COG01633	-	S	Ferritin-like domain
arCOG06053			S	Uncharacterized conserved protein
arCOG06115			S	Uncharacterized conserved protein
arCOG06133			S	Uncharacterized conserved protein
arCOG06147			S	Uncharacterized conserved protein
arCOG06398			S	Uncharacterized conserved protein
arCOG06529			S	Uncharacterized conserved protein
arCOG06566			S	Uncharacterized conserved membrane protein
arCOG06655			S	Uncharacterized conserved protein
arCOG06883			S	Zn-ribbon domain containing protein
arCOG06986			S	Uncharacterized conserved protein
arCOG07110	COG05617	-	S	Predicted integral membrane protein
arCOG07169			S	Uncharacterized conserved protein
arCOG07425			S	Uncharacterized conserved protein
arCOG07491			S	Uncharacterized conserved protein
arCOG07492			S	Uncharacterized conserved protein
arCOG07520	COG02442	-	S	Uncharacterized conserved protein
arCOG07527			S	Predicted metal-binding protein
arCOG07532			S	Uncharacterized conserved protein
arCOG07571			S	Uncharacterized conserved membrane protein
arCOG07626			S	Uncharacterized conserved protein
arCOG07703			S	Uncharacterized conserved protein
arCOG07842			S	Uncharacterized conserved protein
arCOG08221			S	Uncharacterized conserved protein
arCOG08303	COG02880	-	S	Uncharacterized conserved protein
arCOG08358			S	Uncharacterized conserved protein
arCOG09164	COG01266		S	Uncharacterized conserved protein
arCOG09425			S	Uncharacterized conserved protein
arCOG09431			S	Uncharacterized conserved protein
arCOG09434			S	Uncharacterized conserved protein

arCOG09748			S	Uncharacterized conserved protein
arCOG10126	COG01422		S	Uncharacterized conserved protein
arCOG10150			S	Uncharacterized conserved protein
arCOG10161			S	Uncharacterized conserved protein
arCOG10167			S	Uncharacterized conserved protein
arCOG10269			S	Uncharacterized conserved protein
arCOG02053	COG00589	UspA	T	Nucleotide-binding protein, UspA family
arCOG04941	COG03830	-	T	ACT domain-containing protein
arCOG03413	COG02453	CDC14	T	Protein-tyrosine phosphatase
arCOG01143	COG00639	ApaH	T	Serine/threonine protein phosphatase PP2A family
arCOG00449	COG00589	UspA	T	Nucleotide-binding protein, UspA family
arCOG01174	COG00467	RAD55	T	RecA-superfamily ATPase implicated in signal transduction
arCOG05302	COG00631	PTC1	T	Serine/threonine protein phosphatase
arCOG01919	COG00805	TatC	U	Sec-independent protein secretion pathway component TatC
arCOG02694	COG01826	TatA	U	Sec-independent protein secretion pathway component
arCOG02957	COG04023	SBH1	U	Preprotein translocase subunit Sec61beta
arCOG01217	COG01400	SEC65	U	Signal recognition particle 19 kDa protein
arCOG01227	COG00552	FtsY	U	Signal recognition particle GTPase
arCOG01228	COG00541	Ffh	U	Signal recognition particle GTPase
arCOG03383	COG00823	TolB	U	Periplasmic component of the Tol biopolymer transport system
arCOG04035	COG03451	VirB4	U	Type IV secretory pathway, VirB4 component
arCOG07496	COG03451	VirB4	U	Type IV secretory pathway, VirB4 component
arCOG00194	COG01131	CcmA	V	ABC-type multidrug transport system, ATPase component
arCOG02730	COG01848	-	V	PIN domain containing protein
arCOG00790	COG01468	Cas4	V	CRISPR-associated protein Cas4, RecB family exonuclease
arCOG01009	COG03609	-	V	Ribbon-helix-helix protein, copG family
arCOG03779	COG01401	McrB	V	GTPase subunit of restriction endonuclease
arCOG07589	COG01848		V	PIN domain containing protein
arCOG00713	COG01848	-	V	PIN domain containing protein
arCOG00715	COG01848	-	V	PIN domain containing protein
arCOG01463	COG00842	-	V	ABC-type multidrug transport system, permease component
arCOG02219	COG01487	VapC	V	PIN domain containing protein
arCOG02632	COG00286	HsdM	V	Type I restriction-modification system methyltransferase subunit
arCOG02681	COG01753	-	V	Predicted antitoxin, copG family
arCOG03482		Cas7/Csc2	V	CRISPR system related protein, RAMP superfamily Cas7 group
arCOG04502	COG02402	-	V	PIN domain containing protein
arCOG08216			V	Antitoxin MazE superfamily protein
arCOG00710	COG01848	-	V	PIN domain containing protein
arCOG00711	COG01848	-	V	PIN domain containing protein
arCOG00717	COG02405	-	V	PIN domain containing protein
arCOG00719	COG02405	-	V	PIN domain containing protein
arCOG00720	COG01458	-	V	PIN domain containing protein
arCOG00722	COG02886	-	V	Predicted antitoxins containing the HTH domain
arCOG00730	COG01848	-	V	PIN domain containing protein
arCOG00786	COG01468	Cas4	V	CRISPR-associated protein Cas4, RecB family exonuclease
arCOG01008	COG00864	NikR	V	Transcriptional regulator, CopG/Arc/MetJ family (DNA-binding and a metal-binding domains)
arCOG01441		Cas8a2	V	CRISPR system related protein, large subunit of CASCADE complex
arCOG01444	COG01203	Cas3	V	CRISPR-associated helicase Cas3
arCOG01731	COG00534	NorM	V	Na+-driven multidrug efflux pump
arCOG02121	COG05378	-	V	PIN domain containing protein
arCOG02635	COG01002	-	V	Type II restriction enzyme, methylase subunit
arCOG02654	COG03337	-	V	CRISPR system related protein, small subunit of CASCADE complex

	arCOG02658	COG01337	Cas7/Csm3	V	CRISPR system related protein, RAMP superfamily Cas7 group
	arCOG02666	COG01353	Cas10	V	CRISPR associated protein, polymerase-related domain and HD family nuclease
	arCOG02672	COG01688	Cas5	V	CRISPR system related protein Cas5, RAMP superfamily
	arCOG03222	COG01567	Cas5/Csm4	V	CRISPR system related protein, RAMP superfamily Cas5 group
	arCOG03590			V	Addiction module toxin, Txe/YoeB family
	arCOG03718	COG01332	Cas7/Csm5	V	CRISPR system related protein, RAMP superfamily Cas7 group
	arCOG03788	COG05608		V	LEA14-like dessication related protein
	arCOG03823			V	CRISPR system associated protein, predicted small subunit of CASCADE complex
	arCOG03839	COG02253	-	V	Predicted component of viral defense system
	arCOG03847	COG01517		V	CRISPR system related protein, COG1517 family
	arCOG03886			V	CopG family DNA-binding protein
	arCOG03891	COG01367	Cas7/Cmr1	V	CRISPR system related protein, RAMP superfamily Cas7 group
	arCOG04793	COG05340	-	V	Transcriptional regulator, predicted component of viral defense system
	arCOG05206			V	Addiction module toxin, Txe/YoeB family, similar to arCOG03590
	arCOG05269		Cas8a1	V	CRISPR-associated protein, large subunit of CASCADE complex, MTH1090 family
	arCOG05297	COG02402	-	V	PIN domain containing protein
	arCOG05724	COG02810	-	V	Predicted type IV restriction endonuclease
	arCOG06487	COG01421		V	CRISPR system related protein, small subunit of CASCADE complex
	arCOG06948			V	PIN domain
	arCOG06987			V	CopG-family DNA-binding protein
	arCOG07235			V	RHH/CopG DNA binding protein
	arCOG07514			V	RHH/CopG family DNA-binding protein
	arCOG07641	COG01517		V	CRISPR system related protein, COG1517 family
	arCOG07794	COG01401	McrB	V	GTPase subunit of restriction endonuclease
	arCOG07904	COG04634		V	PIN domain
	arCOG10147			V	CRISPR-associated endoribonuclease Cas6
**Gain in Nst1**					
	arCOG00420	COG01085	GalT	C	Galactose-1-phosphate uridylyltransferase
	arCOG02721	COG01271	CydA	C	Cytochrome bd-type quinol oxidase, subunit 1
	arCOG05072	COG02414	-	C	Aldehyde:ferredoxin oxidoreductase
	arCOG03672			E	Thermopsin-like protease
	arCOG01770	COG00705		E	Rhomboid family mebrane associated serine protease
	arCOG03611			E	Peptidase C1A subfamily
	arCOG04490	COG01945	-	E	Pyruvoyl-dependent arginine decarboxylase (PvlArgDC)
	arCOG03713	COG00209	NrdA	F	Ribonucleotide reductase associated ATP-cone domain
	arCOG00090	COG00518	GuaA	F	GMP synthase - Glutamine amidotransferase domain
	arCOG03214	COG00207	ThyA	F	Thymidylate synthase
	arCOG03287	COG03408	GDB1	G	Glycogen debranching enzyme
	arCOG00052	COG00166	Pgi	G	Glucose-6-phosphate isomerase
	arCOG01993	COG00588	GpmA	G	Phosphoglycerate mutase 1
	arCOG05412	COG02723	BglB	G	Beta-glucosidase/6-phospho-beta-glucosidase/beta-galactosidase
	arCOG00408	COG01190	LysU	J	Lysyl-tRNA synthetase (class II)
	arCOG03698	COG04344	-	K	Predicted transciptional regulator, contains HTH domain
	arCOG00395	COG02512	-	K	Predicted membrane-associated trancriptional regulator
	arCOG02217			K	Predicted antitoxin associated with PIN domain
	arCOG04032			L	Eukaryotic-type DNA primase, catalytic (small) subunit homolog, fused to HTH domain

	arCOG00461	COG02231	-	L	Uncharacterized protein related to Endonuclease III
	arCOG02207	COG00708	XthA	L	Exonuclease III
	arCOG01397	COG00463	WcaA	M	Glycosyltransferase
	arCOG01391	COG01215	-	M	Glycosyltransferase
	arCOG01573	COG00668	MscS	M	Small-conductance mechanosensitive channel
	arCOG05398	COG00463		M	Glycosyltransferase family A
	arCOG01417	COG00438	RfaG	M	Glycosyltransferase
	arCOG04922	COG00438	RfaG	M	Glycosyltransferase
	arCOG07390	COG00438	RfaG	M	Glycosyltransferase
	arCOG02964	COG03351	FlaD	N	Putative archaeal flagellar protein D/E
	arCOG03871			N	Flagellar protein G
	arCOG02868	COG01651	DsbG	O	Protein-disulfide isomerase
	arCOG01400	COG00500		Q	SAM-dependent methyltransferase
	arCOG00423	COG02404	-	R	Predicted phosphohydrolase (DHH superfamily)
	arCOG01154	COG04186	-	R	Calcineurin-like phosphoesterase superfamily protein
	arCOG00607	COG01994	-	R	Zn-dependent protease fused to CBS domain
	arCOG01197	COG01708	-	R	Minimal nucleotidyltransferase
	arCOG03461	COG04049	-	R	Uncharacterized protein containing archaeal-type C2H2 Zn-finger
	arCOG10265			S	Uncharacterized conserved protein
	arCOG10353			S	Uncharacterized conserved protein
	arCOG01667	COG03369	-	S	Zinc finger domain containing protein (CDGSH-type)
	arCOG01669	COG03370	-	S	Uncharacterized conserved protein
	arCOG03119			S	DedA family membrane protein
	arCOG03869			S	Uncharacterized conserved protein
	arCOG03113	COG01430	-	S	Uncharacterized conserved protein
	arCOG03749	COG04243	-	S	Predicted membrane protein
	arCOG03962	COG02340	-	S	Uncharacterized protein with SCP/PR1 domains
	arCOG04574	COG01704	LemA	S	Uncharacterized conserved protein
	arCOG05350			S	Uncharacterized conserved protein
	arCOG05360			S	Uncharacterized conserved protein
	arCOG05419			S	Uncharacterized conserved protein
	arCOG05534			S	Uncharacterized conserved protein
	arCOG05631			S	Uncharacterized conserved protein
	arCOG06218			S	Uncharacterized conserved protein
	arCOG06957			S	Uncharacterized conserved protein
	arCOG07442			S	Uncharacterized conserved protein
	arCOG07455			S	Uncharacterized conserved protein
	arCOG00729	COG04113	-	V	PIN domain containing protein
	arCOG03521	COG01002	-	V	Type II restriction enzyme, methylase subunit
	arCOG02218			V	RHH/CopG DNA binding protein
	arCOG00726	COG04113	-	V	PIN domain containing protein
	arCOG06139	COG02250		V	HEPN domain containing protein
	arCOG03769			S	Uncharacterized conserved membrane protein
**Loss in Nst1**					
	arCOG00865	COG01156	NtpB	C	Archaeal/vacuolar-type H+-ATPase subunit B
	arCOG00868	COG01155	NtpA	C	Archaeal/vacuolar-type H+-ATPase subunit A
	arCOG02455	COG00636	AtpK	C	Archaeal/vacuolar-type Na+/H+-ATPase, subunit K
	arCOG04101	COG01394	NtpD	C	Archaeal/vacuolar-type H+-ATPase subunit D
	arCOG04138	COG01269	NtpI	C	Archaeal/vacuolar-type H+-ATPase subunit I
	arCOG01356	COG01032	-	C	Radical SAM superfamily enzyme
	arCOG02146	COG02033	-	C	Desulfoferredoxin
	arCOG04247	COG02317	-	E	Zn-dependent carboxypeptidase
	arCOG00245	COG00287	TyrA	E	Prephenate dehydrogenase
	arCOG01352	COG00334	GdhA	E	Glutamate dehydrogenase/leucine dehydrogenase
	arCOG02098	COG01605	PheA	E	Chorismate mutase

arCOG02297	COG00115	IlvE	E	Branched-chain amino acid aminotransferase/4-amino-4-deoxychorismate lyase
arCOG01883	COG01351	THY1	F	Thymidylate synthase
arCOG04889	COG01328	NrdD	F	Oxygen-sensitive ribonucleoside-triphosphate reductase
arCOG07813			G	Glycosyl hydrolases family 43
arCOG00972	COG01056	NadR	H	Nicotinamide mononucleotide adenylyltransferase
arCOG01259	COG01028	FabG	I	Short-chain alcohol dehydrogenase
arCOG01717	COG00154	GatA	J	Asp-tRNAAsn/Glu-tRNAGln amidotransferase A subunit or related amidase
arCOG00910	COG02263	-	J	Predicted RNA methylase
arCOG00993	COG01374	NIP7	J	Protein involved in ribosomal biogenesis, contains PUA domain
arCOG01346	COG01534	-	J	Predicted RNA-binding protein containing KH domain, possibly ribosomal protein
arCOG01718	COG00064	GatB	J	Asp-tRNAAsn/Glu-tRNAGln amidotransferase B subunit
arCOG02726	COG00721	GatC	J	Asp-tRNAAsn/Glu-tRNAGln amidotransferase C subunit
arCOG04108	COG02051	RPS27A	J	Ribosomal protein S27E
arCOG04154	COG02007	RPS8A	J	Ribosomal protein S8E
arCOG04209	COG01632	RPL15A	J	Ribosomal protein L15E
arCOG04240	COG00100	RpsK	J	Ribosomal protein S11
arCOG04277	COG00231	Efp	J	Translation elongation factor P (EF-P)/translation initiation factor 5A (eIF-5A)
arCOG00485	COG01384	LysS	J	Lysyl-tRNA synthetase (class I)
arCOG00990	COG01549	-	J	Queuine tRNA-ribosyltransferase, contain PUA domain
arCOG01117	COG01522	Lrp	K	Lrp/AsnC family C-terminal domain
arCOG02242	COG03355	-	K	Transcriptional regulator, contains HTH domain
arCOG00998	COG01958	LSM1	K	Small nuclear ribonucleoprotein (snRNP) homolog
arCOG01760	COG00195	NusA	K	Transcription elongation factor
arCOG04479	COG03357	-	K	Predicted transcriptional regulator containing an HTH domain fused to a Zn-ribbon
arCOG02724	COG00350	Ada	L	Methylated DNA-protein cysteine methyltransferase
arCOG00463	COG01833	-	L	Uri superfamily endonuclease
arCOG00929	COG01515	Nfi	L	Deoxyinosine 3'endonuclease (endonuclease V)
arCOG00047	COG01041	-	L	Predicted DNA modification methylase
arCOG00415	COG00468	RecA	L	RecA/RadA recombinase
arCOG00459	COG00177	Nth	L	EndoIII-related endonuclease
arCOG03192	COG01525	-	L	Micrococcal nuclease (thermonuclease)
arCOG01568	COG00668	MscS	M	Small-conductance mechanosensitive channel
arCOG01403	COG00438	RfaG	M	Glycosyltransferase
arCOG01381	COG00463	WcaA	M	Glycosyltransferase
arCOG01811	COG02064	TadC	N	Flp pilus assembly protein TadC
arCOG01812	COG02064	TadC	N	Flp pilus assembly protein TadC
arCOG00946	COG01180	PflA	O	Pyruvate-formate lyase-activating enzyme
arCOG01306	COG01222	RPT1	O	ATP-dependent 26S proteasome regulatory subunit
arCOG00952	COG01180	PflA	O	Pyruvate-formate lyase-activating enzyme
arCOG01311	COG00616	SppA	O	Periplasmic serine protease (ClpP class)
arCOG04236	COG00396	sufC	O	Cysteine desulfurase activator ATPase
arCOG02881	COG00530	ECM27	P	Ca2+/Na+ antiporter
arCOG02265	COG00598	CorA	P	Mg2+ and Co2+ transporter
arCOG04430	COG01078	-	R	HD superfamily phosphohydrolase
arCOG00497	COG02220	-	R	Zn-dependent hydrolase of the beta-lactamase fold
arCOG00458	COG01913	-	R	Predicted Zn-dependent protease
arCOG00614	COG01994	SpoIVFB	R	Zn-dependent protease
arCOG00434	COG00714	-	R	MoxR-like ATPase
arCOG00499	COG01235	PhnP	R	Metal-dependent hydrolase of the beta-lactamase superfamily
arCOG00938	COG00535	-	R	Radical SAM superfamily enzyme

	arCOG01360	COG01244	-	R	MiaB family, Radical SAM enzyme
	arCOG02292	COG00546	Gph	R	HAD superfamily hydrolase
	arCOG02642	COG00628	yhhT	R	Predicted permease, member of the PurR regulon
	arCOG02742	COG01721	-	R	Uncharacterized conserved protein, DUF58 family, contains vWF domain
	arCOG03450			R	Predicted surface protease of transglutaminase family
	arCOG01336	COG02078	AMMECR1	S	Uncharacterized conserved protein
	arCOG02177	COG01967	-	S	Predicted membrane protein
	arCOG04469	COG01784	-	S	Predicted membrane protein
	arCOG01181	COG00478	-	T	RIO-like serine/threonine protein kinase fused to N-terminal HTH domain
	arCOG00721	COG01439	-	V	Predicted nucleic acid-binding protein, consists of a PIN domain and a Zn-ribbon module
	arCOG00922	COG01136	SalX	V	ABC-type antimicrobial peptide transport system, ATPase component
	arCOG01452	COG01518	Cas1	V	CRISPR-associated protein Cas1
	arCOG02312	COG00577	SalY	V	ABC-type antimicrobial peptide transport system, permease component
	arCOG00794	COG01468	Cas4	V	CRISPR-associated protein Cas4, RecB family exonuclease
	arCOG03617	COG01857	Cas7	V	CRISPR system related protein Cas7, RAMP superfamily
	arCOG04194	COG01343	Cas2	V	CRISPR-associated protein Cas2
**Gain in N.equitans**					
	arCOG02200	COG01018	Hmp	C	Flavodoxin reductase (ferredoxin-NADPH reductase) family 1
	arCOG00072	COG00175	CysH	E	3'-phosphoadenosine 5'-phosphosulfate sulfotransferase (PAPS reductase)/FAD synthetase or related enzyme
	arCOG04322	COG00260	PepB	E	Leucyl aminopeptidase
	arCOG00014	COG00524	RbsK	G	Sugar kinase, ribokinase family
	arCOG00877	COG01061	SSL2	K	DNA or RNA helicase of superfamily II
	arCOG06583	COG00648	Nfo	L	Endonuclease IV
	arCOG00331	COG00417	PolB	L	DNA polymerase elongation subunit (family B), C-terminal domain
	arCOG01188	COG02192	-	O	Predicted carbamoyl transferase, NodU family
	arCOG01716	COG00719	sufB	O	Cysteine desulfurase activator SufB
	arCOG02404	COG00785	CcdA	O	Cytochrome c biogenesis protein
	arCOG04355	COG01275	TehA	P	Tellurite resistance protein or related permease
	arCOG09607	COG02761	FrnE	Q	Predicted dithiol-disulfide isomerase involved in polyketide biosynthesis
	arCOG01065	COG00446	HcaD	R	NAD(FAD)-dependent dehydrogenase
	arCOG01378	COG01524	-	R	Uncharacterized protein of the AP superfamily
	arCOG00626	COG01253	TlyC	R	Hemolysins or related protein containing CBS domains
	arCOG04065	COG00612	PqqL	R	Predicted Zn-dependent peptidase
	arCOG00428	COG00608	Cdc45	S	Protein similar to exonuclease RecJ central domain
	arCOG02737	COG01433	-	S	NifX family protein
	arCOG03648			S	Uncharacterized conserved protein
	arCOG05748			S	Uncharacterized conserved protein
	arCOG06945			S	Uncharacterized conserved protein
	arCOG09421			S	Uncharacterized conserved protein
	arCOG01445	COG01203	Cas3	V	CRISPR-associated helicase Cas3
	arCOG02758	COG01688	Cas5	V	CRISPR system related protein Cas5, RAMP superfamily
	arCOG04342	COG01583	-	V	CRISPR system related protein, RAMP superfamily
**Loss in N.equitans**					
	arCOG01340	COG01042	-	C	Acyl-CoA synthetase (NDP forming)
	arCOG01338	COG01042	-	C	Acyl-CoA synthetase, ATP-grasp containing subunit
	arCOG00288	COG00778	NfnB	C	Nitroreductase

arCOG01252	COG01012	PutA	C	Lactaldehyde dehydrogenase, Succinate semialdehyde dehydrogenase or other NAD-dependent aldehyde dehydrogenase
arCOG01599	COG01013	PorB	C	Pyruvate:ferredoxin oxidoreductase or related 2-oxoacid:ferredoxin oxidoreductase, beta subunit
arCOG01606	COG00674	PorA	C	Pyruvate:ferredoxin oxidoreductase or related 2-oxoacid:ferredoxin oxidoreductase, alpha subunit and gamma
arCOG01674	COG01254	AcyP	C	Acylphosphatase
arCOG00371	COG01196	Smc	D	Chromosome segregation ATPase
arCOG02097	COG00710	AroD	E	3-dehydroquinate dehydratase
arCOG02969	COG00308	PepN	E	Aminopeptidase N
arCOG04276	COG00209	NrdA	F	Ribonucleotide reductase, alpha subunit
arCOG01891	COG00125	Tmk	F	Thymidylate kinase
arCOG03714	COG01328	NrdD	F	ATP cone domain
arCOG01696	COG03635	-	G	2,3-bisphosphoglycerate-independent phosphoglycerate mutase
arCOG00144	COG00477	ProP	G	Permease of the major facilitator superfamily
arCOG01421	COG00058	GlgP	G	Glucan phosphorylase
arCOG03278	COG01449	-	G	Glycosyl hydrolase family 57
arCOG00132	COG00477	ProP	G	Permease of the major facilitator superfamily
arCOG00767	COG01109	{ManB}	G	Phosphomannomutase
arCOG01087	COG00149	TpiA	G	Triosephosphate isomerase
arCOG01169	COG00148	Eno	G	Enolase
arCOG04180	COG01980	-	G	Bifunctional fructose-1,6-bisphosphate aldolase/phosphatase FBPA/FBPase
arCOG01895	COG01082	IolE	G	Sugar phosphate isomerase/epimerase
arCOG04120	COG00469	PykF	G	Pyruvate kinase
arCOG01589	COG00189	RimK	H	Glutathione synthase/glutaminyl transferase/alpha-L-glutamate ligase
arCOG01978	COG00294	FolP	H	Dihydropteroate synthase or related enzyme
arCOG04761	COG01968	UppP	I	Undecaprenyl pyrophosphate phosphatase
arCOG03056	COG00671	PgpB	I	Membrane-associated phospholipid phosphatase
arCOG01365	COG01369	POP5	J	RNase P/RNase MRP subunit POP5
arCOG00784	COG01588	POP4	J	RNase P/RNase MRP subunit p29
arCOG04345	COG02023	RPR2	J	RNase P subunit RPR2
arCOG00307	COG01603	RPP1	J	RNase P/RNase MRP subunit p30
arCOG00407	COG00017	AsnS	J	Aspartyl/asparaginyl-tRNA synthetase
arCOG00409	COG00017	AsnS	J	Aspartyl/asparaginyl-tRNA synthetase
arCOG01239	COG01901	RsmE	J	RNA base methyltransferase family enzyme
arCOG04332	COG02606	Ybak	J	Cys-tRNA(Pro)/Cys-tRNA(Cys) deacylase, ybaK family
arCOG01663	COG02026	RelE	K	Cytotoxic translational repressor of toxin-antitoxin stability system
arCOG00367	COG01630	-	L	NurA 5'-3' nuclease
arCOG00417	COG00468	RecA	L	RecA/RadA recombinase
arCOG00427	COG00608	RecJ/Cdc45	L	Single-stranded DNA-specific exonuclease RecJ
arCOG01241	COG00582	XerC	L	Integrase
arCOG05276			L	Pyrimidine dimer DNA glycosylase, T4 endoV family
arCOG00472	COG01474		L	orc1/cdc6 family replication initiation protein fused to HTH domain
arCOG01486	COG01658	-	L	Ribonuclease M5 (contains TOPRIM domain)
arCOG03158	COG01372	-	L	Intein/homing endonuclease
arCOG01411	COG00438	RfaG	M	Glycosyltransferase
arCOG01389	COG01215	-	M	Glycosyltransferase
arCOG01367	COG01091	RfbD	M	dTDP-4-dehydrorhamnose reductase
arCOG04188	COG01898	RfbC	M	dTDP-4-dehydrorhamnose 3,5-epimerase or related enzyme
arCOG00894	COG00463	WcaA	M	Glycosyltransferase
arCOG01410	COG00438	RfaG	M	Glycosyltransferase
arCOG01808	COG02064	TadC	N	Flp pilus assembly protein TadC
arCOG01829	COG01681	FlaB	N	Archaeal flagellins
arCOG01809	COG01955	FlaJ	N	Archaeal flagella assembly protein J
arCOG01822	COG03354	FlaG	N	Putative archaeal flagellar protein G

	arCOG01824	COG03353	FlaF	N	Putative archaeal flagellar protein F
	arCOG04148	COG02874	FlaH	N	Predicted ATPase involved in biogenesis of archaeal flagella
	arCOG01715	COG00719	sufB	O	Cysteine desulfurase activator SufB
	arCOG00561	COG04346	-	O	Predicted membrane-bound dolichyl-phosphate-mannose-protein mannosyltransferase
	arCOG01915	COG00330	HflC	O	Membrane protease subunit, stomatin/prohibitin homolog
	arCOG03580	COG02020	STE14	O	Putative protein-S-isoprenylcysteine methyltransferase
	arCOG01578	COG00474	MgtA	P	Cation transport ATPase
	arCOG04147	COG00605	SodA	P	Superoxide dismutase
	arCOG04231	COG01324	CutA	P	Uncharacterized protein involved in tolerance to divalent cations
	arCOG01943	COG01335	PncA	Q	Amidase related to nicotinamidase
	arCOG03167	COG01373	-	R	Predicted ATPase (AAA+ superfamily)
	arCOG00555	COG01205	-	R	Distinct helicase family with a unique C-terminal domain including a metal-binding cysteine cluster
	arCOG02050	COG00730	-	R	Predicted permease
	arCOG01254	COG02872	-	R	Predicted metal-dependent hydrolase related to alanyl-tRNA synthetase HxxxH domain and second additional domain
	arCOG01263	COG00300	DltE	R	Short-chain dehydrogenase
	arCOG02293	COG00637	-	R	HAD superfamily hydrolase
	arCOG04069	COG02106	-	R	RNA methyltransferase, SPOUT superfamily
	arCOG04409	COG02410	-	R	Predicted nuclease (RNAse H fold)
	arCOG00032	COG01092	-	R	SAM-dependent methyltransferase fused to PUA domain
	arCOG00348	COG02047	-	R	Archaeal enzyme of ATP-grasp superfamily
	arCOG01205	COG01708	-	R	Minimal nucleotidyltransferase
	arCOG01641	COG03269	-	R	Predicted RNA-binding protein, contains TRAM domain
	arCOG03166	COG01672	-	R	AAA+ superfamily ATPase fused to HTH nad RecB nuclease domains
	arCOG03168	COG01373	-	R	Predicted ATPase (AAA+ superfamily)
	arCOG04253	COG01415	-	S	Uncharacterized conserved protein
	arCOG04269	COG01602	-	S	Protein, predicted to be involved in DNA repair
	arCOG04291	COG01801	-	S	Uncharacterized conserved protein
	arCOG01224	COG01849	-	S	Uncharacterized conserved protein
	arCOG02228	COG02246	-	S	Predicted membrane protein
	arCOG04051	COG02412	-	S	Uncharacterized conserved protein
	arCOG04171	COG02090	-	S	Uncharacterized conserved protein
	arCOG00385			S	Uncharacterized conserved protein
	arCOG02123	COG01895	-	S	HEPN domain containing protein
	arCOG02499	COG03420		S	NosD-like periplasmic protein
	arCOG03881			S	Uncharacterized conserved protein
	arCOG01997	COG02095	MarC	U	Multiple antibiotic transporter
	arCOG02673	COG01422	-	U	OxaA/SpoJ/YigC translocase/secretase, sec-independent itegration of nascent memrane proteins into membrane
	arCOG00196	COG01131	CcmA	V	ABC-type multidrug transport system, ATPase component
	arCOG01191	COG02250	-	V	HEPN domain containing protein
	arCOG01467	COG00842	-	V	ABC-type multidrug transport system, permease component
	arCOG02841	COG01132	MdlB	V	ABC-type multidrug transport system, ATPase and permease component
**Gain in Acd1 and other Sulfolobales**					
	arCOG00340	COG00277	GlcD	C	FAD/FMN-containing dehydrogenase fused to Heterodisulfide reductase, subunit B
	arCOG00342	COG02048	HdrB	C	Heterodisulfide reductase, subunit B
	arCOG00343	COG00247	GlpC	C	Fe-S oxidoreductase
	arCOG00345	COG00277	GlcD	C	FAD/FMN-containing dehydrogenase
	arCOG00965	COG01150	HdrC	C	Heterodisulfide reductase, subunit C
	arCOG00966	COG01150	HdrC	C	Heterodisulfide reductase, subunit C
	arCOG01456	COG01062	AdhC	C	Zn-dependent alcohol dehydrogenase
	arCOG01496	COG00243	BisC	C	Molybdopterin oxidoreductase, contains molybdopterin-binding domain

arCOG01928	COG03427	-	C	Carbon monoxide dehydrogenase subunit G, CoxG
arCOG03626	COG04237	HyfE	C	Hydrogenase 4 membrane component (E)
arCOG03696	COG00348	NapH	C	Polyferredoxin
arCOG04548	COG01146	-	C	Ferredoxin
arCOG05375			C	Archaeal succinate dehydrogenase subunit D
arCOG05891			C	Terminal oxidase, subunit doxC
arCOG05892			C	Terminal oxidase, subunit doxE
arCOG05916			C	Predicted heterodisulfide reductase subunit
arCOG06015			C	Cytochrome b558/566, subunit A
arCOG06016			C	Cytochrome b558/566, subunit B
arCOG02234	COG01148	HdrA	C	Heterodisulfide reductase, subunit A, polyferredoxin
arCOG07285		NtpF	C	Archaeal/vacuolar-type H+-ATPase subunit H
arCOG00454	COG05491	ESCRTIII	D	Crenarchaeal division protein ESCRT-III
arCOG00758	COG00665	DadA	E	Glycine/D-amino acid oxidase (deaminating)
arCOG01110	COG00624	ArgE	E	Acetylornithine deacetylase/Succinyl-diaminopimelate desuccinylase or related deacylase
arCOG03465	COG00531	PotE	E	Amino acid transporter
arCOG03652	COG00531	PotE	E	Amino acid transporter
arCOG03657	COG00028		E	Acetolactate synthase large subunit or other thiamine pyrophosphate-requiring enzyme
arCOG03670			E	Thermopsin-like protease
arCOG03674			E	Thermopsin-like protease
arCOG05937	COG00028	IlvB	E	Acetolactate synthase large subunit or other thiamine pyrophosphate-requiring enzyme
arCOG05991			E	ACT domain
arCOG01597	COG00026	PurK	F	Phosphoribosylaminoimidazole carboxylase (NCAIR synthetase)
arCOG03658	COG00208	NrdF	F	Ribonucleotide reductase, beta subunit
arCOG03713	COG00209	NrdA	F	Ribonucleotide reductase associated ATP-cone domain
arCOG04558	COG01957	URH1	F	Inosine-uridine nucleoside N-ribohydrolase
arCOG05138	COG00248	GppA	F	Exopolyphosphatase
arCOG02683	COG00477	ProP	G	Permease of the major facilitator superfamily
arCOG02687	COG00477	ProP	G	Permease of the major facilitator superfamily
arCOG02689	COG00477	ProP	G	Permease of the major facilitator superfamily
arCOG03287	COG03408	GDB1	G	Glycogen debranching enzyme
arCOG05705	COG03839	MalK	G	ABC-type sugar transport system, ATPase component
arCOG05971	COG00477		G	MFS family permease
arCOG00572	COG00029	NadB	H	Aspartate oxidase
arCOG00645	COG02875	CobM	H	Precorrin-4 methylase
arCOG00647	COG01010	CobJ	H	Precorrin-3B methylase
arCOG00648	COG02243	CobF	H	Precorrin-2 methylase
arCOG00651	COG02073	CbiG	H	Cobalamin biosynthesis protein CbiG
arCOG00652	COG02073	CbiG	H	Cobalamin biosynthesis protein CbiG
arCOG00977	COG02242	CobL	H	Precorrin-6B methylase 2
arCOG02247	COG02082	CobH	H	Precorrin isomerase
arCOG04383	COG01903	CbiD	H	Cobalamin biosynthesis protein CbiD
arCOG05973	COG02226	UbiE	H	Methylase involved in ubiquinone/menaquinone biosynthesis
arCOG00251	COG01250	FadB	I	3-hydroxyacyl-CoA dehydrogenase
arCOG01032	COG03890	ERG8	I	Phosphomevalonate kinase
arCOG02937	COG03407	MVD1	I	Mevalonate pyrophosphate decarboxylase
arCOG01702	COG01676	SEN2	J	tRNA splicing endonuclease
arCOG07849			J	Ribosome associated protein L45a
arCOG00817	COG02002	AbrB	K	Transcriptional regulator AbrB
arCOG01010	COG02161	StbD	K	Antitoxin (DNA-binding domain) of toxin-antitoxin stability system
arCOG03737			K	Transcriptional regulator of GntR family
arCOG04480	COG02002	AbrB	K	Transcriptional regulator AbrB
arCOG05938		Rpo13	K	DNA-directed RNA polymerase subunit 13

arCOG05961	COG01321	TroR	K	Mn-dependent transcriptional regulator (DtxR family)
arCOG07936	COG04742	-	K	HTH containing transcriptional regulator
arCOG00283	COG00433	-	L	HerA helicase
arCOG00461	COG02231	-	L	Uncharacterized protein related to Endonuclease III
arCOG02134	COG03316	-	L	Transposase
arCOG02207	COG00708	XthA	L	Exonuclease III
arCOG04357	COG01059	-	L	Thermostable 8-oxoguanine DNA glycosylase
arCOG05888			L	Chromosomal protein Sac7d/Sso7d
arCOG05927			L	NurA family nuclease
arCOG00664	COG01209	RfbA	M	dTDP-glucose pyrophosphorylase
arCOG01397	COG00463	WcaA	M	Glycosyltransferase
arCOG04762	COG00451	WcaG	M	Nucleoside-diphosphate-sugar epimerase
arCOG05943	COG00463	WcaA	M	Glycosyltransferase
arCOG05986			N	Predicted component of pili system
arCOG07276			N	Pilin/Flagellin
arCOG00637	COG01973	HypE	O	Hydrogenase maturation factor
arCOG02072	COG00425	SirA	O	Predicted redox protein, regulator of disulfide bond formation
arCOG03202	COG00826	-	O	Collagenase family protease
arCOG03665	COG04934	-	O	Subtilase family protease
arCOG06207	COG03526	-	O	Rdx family selenoprotein
arCOG02246	COG02138	-	P	Sirohydrochlorin ferrochelatase
arCOG02853	COG02146	{NirD}	P	Ferredoxin subunit of nitrite reductase or ring-hydroxylating dioxygenase
arCOG02860	COG00288	CynT	P	Carbonic anhydrase
arCOG06003	COG00226	PstS	P	ABC-type phosphate transport system, periplasmic component
arCOG06023	COG03376	HoxN	P	High-affinity nickel permease
arCOG01776	COG00500		Q	SAM-dependent methyltransferase
arCOG02869	COG02761	FrnE	Q	Predicted dithiol-disulfide isomerase involved in polyketide biosynthesis
arCOG03000	COG03435	-	Q	Gentisate 1,2-dioxygenase
arCOG06004	COG03971		Q	2-keto-4-pentenoate hydratase
arCOG07335	COG00500		Q	SAM-dependent methyltransferase
arCOG00326	COG00123	AcuC	R	Deacetylase, including yeast histone deacetylase and acetoin utilization protein
arCOG00945	COG00641	AslB	R	Radical SAM superfamily enzyme
arCOG01154	COG04186	-	R	Calcineurin-like phosphoesterase superfamily protein
arCOG01189	COG00661	AarF	R	Predicted unusual protein kinase
arCOG01457	COG01064	AdhP	R	D-arabinose 1-dehydrogenase or another Zn-dependent alcohol dehydrogenase
arCOG01505	COG03381	TorD	R	Uncharacterized component of anaerobic dehydrogenase
arCOG01991	COG00406	phoE	R	Broad specificity phosphatase PhoE or related phosphatase
arCOG02066	COG02044	-	R	Predicted peroxiredoxin
arCOG02067	COG02044	-	R	Predicted peroxiredoxin
arCOG03038	COG00457	NrfG	R	TPR repeats containing protein
arCOG03233	COG04938	-	R	Predicted ATPase
arCOG03428	COG00457	NrfG	R	TPR repeats containing protein
arCOG03738			R	P-loop NTPase superfamily protein
arCOG03844			R	TlpA/TrxA-like family protein
arCOG05348	COG00446	HcaD	R	NAD(FAD)-dependent dehydrogenase
arCOG05403	COG02329	-	R	Uncharacterized enzyme involved in biosynthesis of extracellular polysaccharides
arCOG05919	COG00546	Gph	R	HAD superfamily hydrolase
arCOG05932	COG00491	GloB	R	Zn-dependent hydrolase, glyoxylase family
arCOG05933			R	Rossmann-fold NAD(P)(+)-binding protein
arCOG05959	COG00622		R	Predicted ICC-like phosphoesterase
arCOG05985	COG02107		R	Predicted periplasmic solute-binding protein
arCOG06001	COG03889		R	Predicted periplasmic protein
arCOG06026	COG01011		R	HAD superfamily hydrolase
arCOG07183	COG02244	RfbX	R	Polysaccharide biosynthesis protein, Mvin family
arCOG07273			R	Predicted periplasmic protein
arCOG07852			R	Predicted regulatory ATPase

arCOG01098			S	Uncharacterized conserved protein
arCOG01106			S	Uncharacterized conserved protein
arCOG01212			S	Uncharacterized conserved protein
arCOG01669	COG03370	-	S	Uncharacterized conserved protein
arCOG01714	COG05440	-	S	Uncharacterized conserved protein
arCOG02114	COG02427	-	S	Uncharacterized conserved protein
arCOG02115	COG02427	-	S	Uncharacterized conserved protein
arCOG02738	COG01433	-	S	NifX family protein
arCOG02979			S	Uncharacterized conserved protein
arCOG03692			S	Uncharacterized conserved protein
arCOG03699			S	Uncharacterized conserved protein
arCOG03778			S	Uncharacterized conserved protein
arCOG03851			S	Uncharacterized conserved protein
arCOG03852			S	Uncharacterized conserved protein
arCOG03853			S	Uncharacterized conserved protein
arCOG03854			S	Uncharacterized conserved protein
arCOG03855			S	Uncharacterized conserved protein
arCOG03861			S	Uncharacterized conserved protein
arCOG03869			S	Uncharacterized conserved protein
arCOG04886			S	Uncharacterized conserved protein
arCOG05323			S	Uncharacterized conserved protein
arCOG05380	COG02149	-	S	Predicted membrane protein
arCOG05495	COG04911	-	S	Uncharacterized conserved protein, contains N-terminal coiled-coil domain
arCOG05546			S	Uncharacterized conserved protein
arCOG05883			S	Uncharacterized conserved protein
arCOG05884			S	Uncharacterized conserved protein
arCOG05885			S	Uncharacterized conserved protein
arCOG05886			S	Uncharacterized conserved protein
arCOG05894			S	Uncharacterized conserved protein
arCOG05895	COG02427	-	S	Uncharacterized conserved protein
arCOG05897			S	Uncharacterized conserved protein
arCOG05898			S	Uncharacterized conserved protein
arCOG05899			S	Cell suface protein
arCOG05903			S	Uncharacterized conserved membrane protein
arCOG05904			S	Uncharacterized conserved protein
arCOG05905			S	Uncharacterized conserved protein
arCOG05907			S	Uncharacterized conserved protein
arCOG05912			S	Uncharacterized conserved protein
arCOG05913			S	Uncharacterized conserved protein
arCOG05917			S	Uncharacterized conserved protein
arCOG05918			S	Uncharacterized conserved protein
arCOG05920			S	Uncharacterized conserved protein
arCOG05921			S	Uncharacterized conserved protein
arCOG05922			S	Uncharacterized conserved protein
arCOG05923			S	Uncharacterized conserved protein
arCOG05925			S	Uncharacterized conserved protein
arCOG05926			S	Uncharacterized conserved protein
arCOG05928			S	Uncharacterized conserved protein
arCOG05929			S	Uncharacterized conserved protein
arCOG05939			S	Uncharacterized conserved protein
arCOG05942			S	Uncharacterized conserved protein
arCOG05946			S	Uncharacterized conserved protein
arCOG05947			S	Uncharacterized conserved protein
arCOG05948			S	Uncharacterized conserved protein
arCOG05949			S	Uncharacterized conserved protein
arCOG05950			S	Uncharacterized conserved protein
arCOG05951			S	Uncharacterized conserved protein
arCOG05952			S	Uncharacterized conserved protein
arCOG05953			S	Uncharacterized conserved protein
arCOG05954			S	Uncharacterized conserved protein
arCOG05955			S	Uncharacterized conserved protein
arCOG05958			S	Uncharacterized conserved protein
arCOG05967			S	Uncharacterized conserved protein
arCOG05972			S	Uncharacterized conserved protein
arCOG05974			S	Uncharacterized conserved protein
arCOG05975			S	Uncharacterized conserved protein
arCOG05979			S	Uncharacterized conserved protein
arCOG05980			S	Uncharacterized conserved protein

arCOG05981			S	Uncharacterized conserved protein
arCOG05983			S	Uncharacterized conserved protein
arCOG05984			S	Uncharacterized conserved protein
arCOG05997			S	Uncharacterized conserved protein
arCOG05998			S	Uncharacterized conserved helical repeat protein
arCOG05999			S	Uncharacterized conserved protein
arCOG06000			S	Uncharacterized conserved protein
arCOG06002			S	Cell suface protein
arCOG06007			S	Uncharacterized conserved protein
arCOG06029			S	Uncharacterized conserved protein
arCOG06032			S	Uncharacterized conserved protein
arCOG06034			S	Uncharacterized conserved protein
arCOG06035			S	Uncharacterized conserved protein
arCOG06038			S	Uncharacterized conserved protein, contains thioredoxin domain
arCOG06039		SlaA	M	S-layer protein SlaA
arCOG06040			S	Uncharacterized conserved protein
arCOG06041			S	Uncharacterized conserved protein
arCOG06046	COG05592	-	S	Hemerythrin HHE cation binding domain containing protein
arCOG06050	COG03431	-	S	Predicted membrane protein
arCOG07173			S	Uncharacterized conserved protein
arCOG07174			S	Uncharacterized conserved protein
arCOG07176			S	Uncharacterized conserved protein
arCOG07177			S	Uncharacterized conserved protein
arCOG07180			S	Uncharacterized conserved protein
arCOG07184			S	Uncharacterized conserved protein
arCOG07188			S	Uncharacterized conserved protein
arCOG07194			S	Uncharacterized conserved protein
arCOG07195			S	Uncharacterized conserved protein
arCOG07197			S	Uncharacterized conserved protein
arCOG07210			S	Uncharacterized conserved protein
arCOG07214			S	Uncharacterized conserved protein
arCOG07215			S	Uncharacterized conserved protein
arCOG07217			S	Uncharacterized conserved protein
arCOG07219			S	Uncharacterized conserved protein
arCOG07220			S	Uncharacterized conserved protein
arCOG07225			S	Uncharacterized conserved protein
arCOG07254	COG05607	-	S	Uncharacterized conserved protein
arCOG07262			S	Uncharacterized conserved protein
arCOG07269			S	Uncharacterized conserved protein
arCOG07271			S	Uncharacterized conserved protein
arCOG07274			S	Uncharacterized conserved protein
arCOG07278			S	Uncharacterized conserved membrane protein
arCOG07283			S	Uncharacterized conserved protein
arCOG07284			S	Uncharacterized conserved protein
arCOG07286			S	Uncharacterized conserved protein
arCOG07287			S	Uncharacterized conserved protein
arCOG07296			S	Uncharacterized conserved protein
arCOG07305			S	Uncharacterized conserved protein
arCOG07314			S	Uncharacterized conserved protein
arCOG07321			S	Uncharacterized conserved protein
arCOG07323			S	Uncharacterized conserved protein
arCOG07329			S	Uncharacterized conserved protein
arCOG07716			S	SWIM Zn-finger
arCOG07718			S	Uncharacterized conserved protein
arCOG07731			S	Uncharacterized conserved protein
arCOG07847			S	Uncharacterized conserved protein
arCOG07934			S	Uncharacterized protein
arCOG08078			S	TRASH domain-containing protein, metal-binding
arCOG08305			S	Uncharacterized conserved protein
arCOG08308			S	Uncharacterized conserved protein
arCOG08309			S	Uncharacterized conserved protein
arCOG08315			S	Uncharacterized conserved protein
arCOG08320			S	Uncharacterized conserved protein
arCOG08322			S	Zn finger protein
arCOG08324			S	Uncharacterized conserved protein

arCOG08332			S	Uncharacterized conserved protein
arCOG08333			S	Uncharacterized conserved protein
arCOG08334			S	Uncharacterized conserved protein
arCOG08341			S	Uncharacterized conserved protein
arCOG08342			S	Uncharacterized conserved protein
arCOG08372			S	Uncharacterized conserved protein
arCOG08380			S	Uncharacterized conserved protein
arCOG08458			S	Uncharacterized conserved protein
arCOG08462			S	Uncharacterized conserved protein
arCOG08475			S	Uncharacterized conserved protein
arCOG08476			S	Uncharacterized conserved protein
arCOG08479			S	Uncharacterized conserved protein
arCOG08538			S	Uncharacterized conserved protein
arCOG08560			S	Uncharacterized conserved protein
arCOG09871			S	SWIM Zn-finger
arCOG09929			S	Uncharacterized conserved protein
arCOG09970			S	Uncharacterized conserved protein
arCOG09993			S	Uncharacterized conserved protein
arCOG10002			S	Uncharacterized conserved protein
arCOG10132			S	Uncharacterized conserved protein
arCOG10265			S	Uncharacterized conserved protein
arCOG05042			S	Uncharacterized conserved protein
arCOG07198			S	Uncharacterized conserved membrane protein
arCOG07213			S	Uncharacterized conserved protein
arCOG05911	COG01826	TatA	U	Sec-independent protein secretion pathway component
arCOG00713	COG01848	-	V	PIN domain containing protein
arCOG02657	COG01336	Cas7/Cmr4	V	CRISPR system related protein, RAMP superfamily Cas7 group
arCOG02681	COG01753	-	V	Predicted antitoxin, copG family
arCOG03436	COG01517	-	V	CRISPR system related protein, COG1517 family
arCOG07237			V	Uncharacterized protein contains PIN domain
arCOG07309		PlrA	V	Plasmid regulatory DNA-binding protein

Loss in Acd1 and other Sulfolobales

arCOG00104	COG01795	-	C	Formaldehyde-activating enzyme nesessary for methanogenesis
arCOG00296	COG01145	NapF	C	Ferredoxin
arCOG00349	COG01141	Fer	C	Ferredoxin
arCOG00422	COG01085	GalT	C	Galactose-1-phosphate uridylyltransferase
arCOG00509	COG00426	FpaA	C	Uncharacterized flavoprotein
arCOG00706	COG02414	-	C	Aldehyde:ferredoxin oxidoreductase
arCOG00707	COG02414	-	C	Aldehyde:ferredoxin oxidoreductase
arCOG00709	COG02414	-	C	Aldehyde:ferredoxin oxidoreductase
arCOG00762	COG02191	-	C	Formylmethanofuran dehydrogenase subunit E
arCOG00961	COG01145	GlpC	C	Ferredoxin
arCOG01355	COG01032	-	C	Radical SAM superfamily enzyme
arCOG01359	COG01031	-	C	Radical SAM superfamily enzyme
arCOG01364	COG01032	-	C	Radical SAM superfamily enzyme
arCOG01497	COG05013	NarG	C	Nitrate reductase alpha subunit
arCOG01501	COG01140	NarY	C	Nitrate reductase beta subunit
arCOG01502	COG01142	HycB	C	Fe-S-cluster-containing hydrogenase component 2
arCOG01504	COG02180	NarJ	C	Nitrate reductase delta subunit
arCOG01607	COG00674	PorA	C	Pyruvate:ferredoxin oxidoreductase or related 2-oxoacid:ferredoxin oxidoreductase, alpha subunit
arCOG01705	COG01036	-	C	Archaeal flavoprotein
arCOG02077	COG00822	IscU	C	NifU homolog involved in Fe-S cluster formation
arCOG02095	COG05016	-	C	Pyruvate/oxaloacetate carboxyltransferase
arCOG02146	COG02033	-	C	Desulfoferredoxin
arCOG02461	COG01146	-	C	Ferredoxin
arCOG02472	COG01941	FrhG	C	Coenzyme F420-reducing hydrogenase, gamma subunit

arCOG02720	COG01271	CydA	C	Cytochrome bd-type quinol oxidase, subunit 1
arCOG02721	COG01271	CydA	C	Cytochrome bd-type quinol oxidase, subunit 1
arCOG03363	COG02811	NtpF	C	Archaeal/vacuolar-type H+-ATPase subunit H
arCOG04073	COG01149	-	C	MinD superfamily P-loop ATPase containing an inserted ferredoxin domain
arCOG04162	COG02142	SdhD	C	Succinate dehydrogenase, hydrophobic anchor subunit
arCOG04279	COG01786	-	C	Swiveling domain associated with predicted aconitase
arCOG04406	COG01838	FumA	C	Tartrate dehydratase beta subunit/Fumarate hydratase class I, C-terminal domain
arCOG04407	COG01951	TtdA	C	Tartrate dehydratase alpha subunit/Fumarate hydratase class I, N-terminal domain
arCOG04537	COG01894	NuoF	C	NADH:ubiquinone oxidoreductase, NADH-binding (51 kD) subunit
arCOG04949	COG03808	OVP1	C	Inorganic pyrophosphatase
arCOG05014	COG02181	HdrE	C	CoB--CoM heterodisulfide reductase subunit E
arCOG05072	COG02414	-	C	Aldehyde:ferredoxin oxidoreductase
arCOG06549			C	Membrane-bound multiheme cytochrome c subunit
arCOG00370	COG01196	Smc	D	Chromosome segregation ATPase
arCOG00371	COG01196	Smc	D	Chromosome segregation ATPase
arCOG04701	COG00239	CrcB	D	Integral membrane protein possibly involved in chromosome condensation
arCOG00055	COG00421	SpeE	E	Spermidine synthase
arCOG00066	COG01104	NifS	E	Cysteine sulfinate desulfinase/cysteine desulfurase or related enzyme
arCOG00071	COG00367	AsnB	E	Asparagine synthase (glutamine-hydrolyzing)
arCOG00073	COG00175	CysH	E	PAPS reductase related enzyme fused to RNA-binding PUA domain and ferredoxin domain
arCOG00075	COG00175	CysH	E	3'-phosphoadenosine 5'-phosphosulfate sulfotransferase (PAPS reductase)/FAD synthetase or related enzyme
arCOG00161	COG01176	PotB	E	ABC-type spermidine/putrescine transport system, permease component I
arCOG00162	COG01177	PotC	E	ABC-type spermidine/putrescine transport system, permease component II
arCOG00220	COG00687	PotD	E	Spermidine/putrescine-binding periplasmic protein
arCOG00243	COG01748	LYS9	E	Saccharopine dehydrogenase or related enzyme
arCOG00771	COG01680		E	CubicO group peptidase, beta-lactamase class C family
arCOG00811	COG00440	IlvH	E	Acetolactate synthase, small subunit
arCOG00863	COG00549	ArcC	E	Carbamate kinase
arCOG00923	COG01126	GlnQ	E	ABC-type polar amino acid transport system, ATPase component
arCOG00926	COG00411	LivG	E	ABC-type branched-chain amino acid transport system, ATPase component
arCOG01020	COG00683	LivK	E	ABC-type branched-chain amino acid transport system, periplasmic component
arCOG01158	COG00560	SerB	E	Phosphoserine phosphatase
arCOG01270	COG00559	LivH	E	Branched-chain amino acid ABC-type transport system, permease component
arCOG01274	COG04177	LivM	E	ABC-type branched-chain amino acid transport system, permease component
arCOG01798	COG00765	HisM	E	ABC-type amino acid transport system, permease component
arCOG01799	COG00834	HisJ	E	ABC-type amino acid transport/signal transduction system, periplasmic component/domain
arCOG02093	COG00119	LeuA	E	Isopropylmalate/homocitrate/citramalate synthase
arCOG02167	COG01305	-	E	Transglutaminase-like enzyme, putative cysteine protease

arCOG02169	COG01305	-	E	Transglutaminase-like enzyme, putative cysteine protease
arCOG02297	COG00115	IlvE	E	Branched-chain amino acid aminotransferase/4-amino-4-deoxychorismate lyase
arCOG02305	COG00347	GlnK	E	Nitrogen regulatory protein PII
arCOG02486	COG01305	-	E	Transglutaminase-like enzyme, putative cysteine protease
arCOG03109	COG01834	-	E	N-Dimethylarginine dimethylaminohydrolase
arCOG03377			E	Serine protease
arCOG03602	COG04690	PepD	E	Dipeptidase
arCOG05680	COG03978		E	Acetolactate synthase small subunit (ACT domain)
arCOG05757	COG04377		E	Metal-dependent membrane protease, CAAX family, DUF2324 subfamily
arCOG00612	COG00516	GuaB	F	IMP dehydrogenase/GMP reductase
arCOG00693	COG01001	AdeC	F	Adenine deaminase
arCOG00700	COG00402	SsnA	F	Cytosine deaminase related metal-dependent hydrolase fused to KH domain
arCOG01046	COG00563	Adk	F	Adenylate kinase or related kinase
arCOG02013	COG00213	DeoA	F	Thymidine phosphorylase
arCOG03214	COG00207	ThyA	F	Thymidylate synthase
arCOG04320	COG00274	DeoC	F	Deoxyribose-phosphate aldolase
arCOG04889	COG01328	NrdD	F	Oxygen-sensitive ribonucleoside-triphosphate reductase
arCOG00026	COG01070	XylB	G	Sugar (pentulose and hexulose) kinase
arCOG00139	COG00477	ProP	G	Permease of the major facilitator superfamily
arCOG00146	COG02211	MelB	G	Na+/melibiose symporter or related transporter
arCOG00149	COG01653	UgpB	G	ABC-type sugar transport system, periplasmic component
arCOG00151	COG01653	UgpB	G	ABC-type sugar transport system, periplasmic component
arCOG00152	COG02182	MalE	G	Maltose-binding periplasmic protein
arCOG00180	COG03839	MalK	G	ABC-type sugar transport system, ATPase component
arCOG00272	COG00697	RhaT	G	permease of the drug/metabolite transporter (DMT) superfamily
arCOG01029	COG00153	GalK	G	Galactokinase
arCOG01518	COG01363	FrvX	G	Cellulase M or related protein
arCOG01772	COG02407	FucI	G	L-fucose isomerase or related protein
arCOG01895	COG01082	IolE	G	Sugar phosphate isomerase/epimerase
arCOG01900	COG01082	IolE	G	Sugar phosphate isomerase/epimerase
arCOG02831	COG00380	OtsA	G	Trehalose-6-phosphate synthase
arCOG02876	COG00726	CDA1	G	Predicted xylanase/chitin deacetylase
arCOG02877	COG00726	CDA1	G	Predicted xylanase/chitin deacetylase
arCOG02878	COG00726	CDA1	G	Predicted xylanase/chitin deacetylase
arCOG02948	COG00366	AmyA	G	Glycosidase
arCOG03003	COG00662		G	Protein containing cupin domain
arCOG04084	COG02152	-	G	Predicted glycosylase
arCOG04158			G	Mannosyl-3-phosphoglycerate synthase
arCOG04226	COG00235		G	Fuculose-1-phosphate aldolase
arCOG04296	COG00477	ProP	G	Permease of the major facilitator superfamily
arCOG04443	COG01850	RbcL	G	Ribulose 1,5-bisphosphate carboxylase, large subunit
arCOG04934	COG00726		G	Polysaccharide deacetylase
arCOG04974	COG03836	HpcH	G	2,4-dihydroxyhept-2-ene-1,7-dioic acid aldolase
arCOG05856	COG01874	LacA	G	Beta-galactosidase
arCOG06070	COG01486	CelF	G	Alpha-galactosidase/6-phospho-beta-glucosidase, family 4 of glycosyl hydrolase
arCOG07158	COG01554	ATH1	G	Sugar hydrolase/phosphorylase
arCOG07452	COG03525	Chb	G	N-acetyl-beta-hexosaminidase
arCOG07490	COG03281	Ble	G	Uncharacterized protein, probably involved in trehalose biosynthesis
arCOG07700	COG02730	BglC	G	Endoglucanase
arCOG07813			G	Glycosyl hydrolases family 43
arCOG08882	COG00698	RpiB	G	Ribose 5-phosphate isomerase RpiB

arCOG00166	COG04662	TupA	H	ABC-type tungstate transport system, periplasmic component
arCOG00229	COG02998	TupB	H	ABC-type tungstate transport system, permease component
arCOG00480	COG01575	MenA	H	1,4-dihydroxy-2-naphthoate octaprenyltransferase
arCOG00656	COG01060	ThiH	H	Thiamine biosynthesis enzyme ThiH, FO synthase or related uncharacterized enzyme
arCOG00954	COG02896	MoaA	H	Molybdenum cofactor biosynthesis enzyme
arCOG01089	COG00352	ThiE	H	Thiamine monophosphate synthase
arCOG01483	COG00157	NadC	H	Nicotinate-nucleotide pyrophosphorylase
arCOG01861	COG03481	MptB	H	Methanopterin biosynthesis cyclic phosphodiesterase, HD-superfamily hydrolase
arCOG02049	COG01587	HemD	H	Uroporphyrinogen-III synthase
arCOG04139	COG01893	ApbA	H	Ketopantoate reductase
arCOG04301	COG01469	MptA	H	Fe(2+)-dependent GTP cyclohydrolase
arCOG04336	COG01962	MtrH	H	Tetrahydromethanopterin S-methyltransferase, subunit H
arCOG06472	COG01240		H	Mg-chelatase subunit ChlD
arCOG08631	COG01995	PdxA	H	Pyridoxal phosphate biosynthesis protein
arCOG00674	COG00558	PgsA	I	Phosphatidylglycerophosphate synthase
arCOG01650	COG02267	PldB	I	Lysophospholipase, alpha-beta hydrolase superfamily
arCOG01843	COG03255	-	I	Putative sterol carrier protein
arCOG01869	COG01267	PgpA	I	Phosphatidylglycerophosphatase A fused to adenosylcobinamide amidohydrolase, CbiZ
arCOG01882	COG00170	SEC59	I	Dolichol kinase
arCOG01986	COG04670	-	I	Acyl CoA:acetate/3-ketoacid CoA transferase
arCOG02041	COG01502	Cls	I	Phosphatidylserine/phosphatidylglycerophosphate/cardiolipin synthase or related enzyme
arCOG03058	COG00671	PgpB	I	Membrane-associated phospholipid phosphatase
arCOG08627			I	Alkaline ceramidase
arCOG00407	COG00017	AsnS	J	Aspartyl/asparaginyl-tRNA synthetase
arCOG00409	COG00017	AsnS	J	Aspartyl/asparaginyl-tRNA synthetase
arCOG00485	COG01384	LysS	J	Lysyl-tRNA synthetase (class I)
arCOG00547	COG00595	-	J	mRNA degradation ribonuclease J1/J2 (metallo-beta-lactamase superfamily)
arCOG00953	COG00731	TYW3	J	Homolog of Wybutosine (yW) biosynthesis enzyme, Fe-S oxidoreductase
arCOG00974	COG00144	Sun	J	tRNA or rRNA cytosine-C5-methylase
arCOG00990	COG01549	-	J	Queuine tRNA-ribosyltransferase, contain PUA domain
arCOG01124	COG01184	GCD2	J	Translation initiation factor 2B subunit, eIF-2B alpha/beta/delta family
arCOG01125	COG01184	GCD2	J	Translation initiation factor 2B subunit, eIF-2B alpha/beta/delta family
arCOG01239	COG01901	RsmE	J	RNA base methyltransferase family enzyme
arCOG02197	COG01617	Cgi121	J	Subunit of KEOPS complex, involved (Cgi121BUD32KAE1)
arCOG03218	COG04021	Thg1	J	tRNAHis guanylyltransferase
arCOG04057			J	Ribosomal L38E
arCOG04063	COG01859	KptA	J	RNA:NAD 2'-phosphotransferase
arCOG04304	COG02451	-	J	Ribosomal protein L35AE/L33A
arCOG04332	COG02606	Ybak	J	Cys-tRNA(Pro)/Cys-tRNA(Cys) deacylase, ybaK family
arCOG00743			K	Predicted transcriptional regulator
arCOG00844	COG00454	WecD	K	Acetyltransferase (GNAT) family
arCOG00876	COG01061	SSL2	K	DNA or RNA helicase of superfamily II
arCOG00921	COG01318	-	K	Predicted transcriptional regulator
arCOG01680	COG00640	ArsR	K	Transcriptional regulator containing HTH domain, ArsR family
arCOG01683	COG00640	ArsR	K	Transcriptional regulator containing HTH domain, ArsR family
arCOG01687	COG00640	ArsR	K	Transcriptional regulator containing HTH domain, ArsR family
arCOG02038	COG01378	-	K	Sugar-specific transcriptional regulator TrmB

arCOG02611	COG03398	-	K	Predicted transcriptional regulator, containd two HTH domains
arCOG02795	COG01510	-	K	Predicted transcriptional regulator
arCOG03880	COG02880	-	K	DNA-binding protein, potential antitoxin AbrB/MazE fold
arCOG03936	COG02002	AbrB	K	Transcriptional regulator AbrB
arCOG04280	COG01940	NagC	K	Transcriptional regulator/sugar kinase
arCOG04554	COG01356	tfx	K	Transcriptional regulator
arCOG04818	COG00553	HepA	K	Superfamily II DNA/RNA helicase, SNF2 family
arCOG07152	COG02880	-	K	DNA-binding protein, potential antitoxin AbrB/MazE fold
arCOG07561	COG02512	-	K	Predicted regulatory protein containing HTH domain
arCOG10049	COG02880		K	DNA-binding protein, potential antitoxin AbrB/MazE fold
arCOG00048	COG00116	-	L	Predicted N6-adenine-specific DNA methylase
arCOG00282	COG00433	-	L	HerA helicase
arCOG00424	COG00608		L	DHH superfamily phosphohydrolase/exonuclease
arCOG00426	COG00608	RecJ	L	DHH superfamily phosphohydrolase/exonuclease
arCOG00464	COG00122	AlkA	L	3-methyladenine DNA glycosylase/8-oxoguanine DNA glycosylase
arCOG00465	COG02231	-	L	Uncharacterized protein related to Endonuclease III
arCOG00472	COG01474		L	orc1/cdc6 family replication initiation protein fused to HTH domain
arCOG00560	COG00514	RecQ	L	Superfamily II DNA helicase
arCOG01291	COG01533	SplB	L	DNA repair photolyase
arCOG02636	COG00827	-	L	Adenine-specific DNA methylase
arCOG03145	COG01372	-	L	Intein/homing endonuclease
arCOG03151	COG01372	-	L	Intein/homing endonuclease
arCOG03154	COG01372	-	L	Intein/homing endonuclease
arCOG03155	COG01241	MCM2	L	Cdc46/Mcm family protein (inactivated)
arCOG03156	COG03780	-	L	DNA endonuclease related to intein-encoded endonuclease
arCOG03158	COG01372	-	L	Intein/homing endonuclease
arCOG03192	COG01525	-	L	Micrococcal nuclease (thermonuclease)
arCOG03242	COG01195	RecF	L	Recombinational DNA repair ATPase RecF
arCOG03727	COG01591		L	Restriction endonuclease family enzyme
arCOG04144	COG04047	-	L	8-oxoguanine DNA glycosylase
arCOG04155	COG00177		L	HhH-GPD superfamily base excision DNA repair protein
arCOG04295	COG02094	Mpg	L	3-methyladenine DNA glycosylase
arCOG04306	COG04998	-	L	Predicted endonuclease (RecB family)
arCOG00118	COG00399	WecE	M	Predicted pyridoxal phosphate-dependent enzyme apparently involved in regulation of cell wall biogenesis
arCOG00252	COG00677	WecC	M	UDP-N-acetyl-D-mannosaminuronate dehydrogenase
arCOG00566	COG01807	ArnT	M	4-amino-4-deoxy-L-arabinose transferase or related glycosyltransferase of PMT family
arCOG00609	COG00750	-	M	Predicted membrane-associated Zn-dependent protease
arCOG00673	COG01213	-	M	Predicted sugar nucleotidyltransferase
arCOG00836	COG01247	-	M	Sortase or related acyltransferase
arCOG01310			M	Uncharacterized conserved protein
arCOG01376	COG00451	WcaG	M	Nucleoside-diphosphate-sugar epimerase
arCOG01390	COG00463	WcaA	M	Glycosyltransferase
arCOG01392	COG00381	WecB	M	UDP-N-acetylglucosamine 2-epimerase
arCOG01405	COG00438	RfaG	M	Glycosyltransferase
arCOG01410	COG00438	RfaG	M	Glycosyltransferase
arCOG01418	COG00438	RfaG	M	Glycosyltransferase
arCOG04468	COG00451	WcaG	M	Nucleoside-diphosphate-sugar epimerase
arCOG01811	COG02064	TadC	N	Flp pilus assembly protein TadC
arCOG01812	COG02064	TadC	N	Flp pilus assembly protein TadC
arCOG00299			O	DsbA family protein
arCOG00702	COG01404	AprE	O	Subtilisin-like serine protease
arCOG00704	COG01404	AprE	O	Ssurface layer-associated subtilisin-like serine protease

arCOG00936	COG01180	PflA	O	Pyruvate-formate lyase-activating enzyme
arCOG00947	COG01180	PflA	O	Pyruvate-formate lyase-activating enzyme
arCOG00948	COG01180	PflA	O	Pyruvate-formate lyase-activating enzyme
arCOG00949	COG01180	PflA	O	Pyruvate-formate lyase-activating enzyme
arCOG00952	COG01180	PflA	O	Pyruvate-formate lyase-activating enzyme
arCOG01311	COG00616	SppA	O	Periplasmic serine protease (ClpP class)
arCOG01911	COG00616	SppA	O	Periplasmic serine protease (ClpP class)
arCOG02163	COG04930	-	O	Predicted ATP-dependent Lon-type protease
arCOG02406	COG00785	CcdA	O	Cytochrome c biogenesis protein
arCOG02407	COG00526	TrxA	O	Thioredoxin fused to Cytochrome c biogenesis protein
arCOG02868	COG01651	DsbG	O	Protein-disulfide isomerase
arCOG02872	COG01651	DsbG	O	Protein-disulfide isomerase
arCOG03741	COG05550	-	O	Predicted aspartyl protease
arCOG03742	COG05550	-	O	Predicted aspartyl protease
arCOG03744	COG05550	-	O	Predicted aspartyl protease
arCOG03745	COG05550	-	O	Predicted aspartyl protease
arCOG04933	COG04826	-	O	Serine protease inhibitor
arCOG05634	COG03058	FdhE	O	Uncharacterized protein involved in formate dehydrogenase formation
arCOG06075	COG00526	TrxA	O	Thiol-disulfide isomerase or thioredoxin
arCOG06823	COG01404	AprE	O	Subtilisin-like serine protease
arCOG00163	COG01178	ThiP	P	ABC-type Fe3+ transport system, permease component
arCOG00169	COG00600	TauC	P	ABC-type nitrate/sulfonate/bicarbonate transport system, permease component
arCOG00201	COG01121	ZnuC	P	ABC-type Mn/Zn transport system, ATPase component
arCOG00202	COG01122	CbiO	P	ABC-type cobalt transport system, ATPase component
arCOG00221	COG01840	AfuA	P	ABC-type Fe3+ transport system, periplasmic component
arCOG00232	COG00704	PhoU	P	Phosphate uptake regulator
arCOG00237	COG00471	CitT	P	Di- and tricarboxylate transporter
arCOG00359	COG00370	FeoB	P	Fe2+ transport system protein B
arCOG00624	COG01824	-	P	Permease, similar to cation transporter
arCOG01005	COG00803	LraI	P	ABC-type metal ion transport system, periplasmic component/surface adhesin
arCOG01006	COG01108	ZnuB	P	ABC-type Mn2+/Zn2+ transport system, permease component
arCOG01040	COG00529	CysC	P	Adenylylsulfate kinase or related kinase
arCOG01094	COG02193	Bfr	P	Bacterioferritin (cytochrome b1)
arCOG01095	COG01528	Ftn	P	Ferritin
arCOG01474	COG00053	MMT1	P	Predicted Co/Zn/Cd cation transporter
arCOG01803	COG00715	TauA	P	ABC-type nitrate/sulfonate/bicarbonate transport system, periplasmic component
arCOG01957	COG00569	TrkA	P	K+ transport system, NAD-binding component
arCOG01964	COG01226	Kch	P	Kef-type K+ transport system, predicted NAD-binding component
arCOG01970	COG00490	-	P	Putative regulatory, ligand-binding protein related to C-terminal domain of K+ channels
arCOG02102	COG01918	FeoA	P	Fe2+ transport system protein A
arCOG02248	COG00310	CbiM	P	ABC-type Co2+ transport system, permease component
arCOG02252	COG00619	CbiQ	P	ABC-type cobalt transport system, permease component CbiQ or related transporter
arCOG02265	COG00598	CorA	P	Mg2+ and Co2+ transporter
arCOG02787	COG03119	AslA	P	Arylsulfatase A or related enzyme
arCOG02849	COG00003	ArsA	P	Oxyanion-translocating ATPase
arCOG03072	COG01006	MnhC	P	Multisubunit Na+/H+ antiporter, MnhC subunit
arCOG03076	COG01563	-	P	Predicted subunit of the Multisubunit Na+/H+ antiporter
arCOG03077	COG02111	MnhB	P	Multisubunit Na+/H+ antiporter, MnhB subunit
arCOG03078	COG01563	-	P	Predicted subunit of the Multisubunit Na+/H+ antiporter

arCOG03079	COG02111	MnhB	P	Multisubunit Na+/H+ antiporter, MnhB subunit
arCOG03082	COG01320	MnhG	P	Multisubunit Na+/H+ antiporter, MnhG subunit
arCOG03099	COG01863	MnhE	P	Multisubunit Na+/H+ antiporter, MnhE subunit
arCOG03121	COG02212	MnhF	P	Multisubunit Na+/H+ antiporter, MnhF subunit
arCOG03159	COG00310	CbiM	P	ABC-type Co2+ transport system, permease component
arCOG03303	COG00614	FepB	P	ABC-type Fe3+-hydroxamate transport system, periplasmic component
arCOG03304	COG00614	FepB	P	ABC-type Fe3+-hydroxamate transport system, periplasmic component
arCOG03454	COG02116	FocA	P	Formate/nitrite family of transporter
arCOG04118			P	Mn2+/Fe2+ transporter, NRAMP family
arCOG04145	COG00168	TrkG	P	Trk-type K+ transport system, membrane component
arCOG04330	COG00672	FTR1	P	High-affinity Fe2+/Pb2+ permease
arCOG05758			P	TrkA-C domain contaning protein
arCOG06434	COG02059	ChrA	P	Chromate transport protein ChrA
arCOG07018	COG02847	-	P	Copper(I)-binding protein
arCOG01774	COG00500		Q	SAM-dependent methyltransferase
arCOG03773	COG00500		Q	SAM-dependent methyltransferase
arCOG04198			Q	Ethylbenzene dehydrogenase
arCOG04347	COG00500		Q	SAM-dependent methyltransferase
arCOG00032	COG01092	-	R	SAM-dependent methyltransferase fused to PUA domain
arCOG00036	COG02102	-	R	Predicted ATPase of PP-loop superfamily
arCOG00041	COG01926	-	R	Predicted phosphoribosyltransferase
arCOG00043	COG01606	-	R	ATP-utilizing enzyme of the PP-loop superfamily
arCOG00054	COG02521	-	R	Predicted archaeal methyltransferase
arCOG00056	COG01818	-	R	Predicted RNA-binding protein, contains THUMP domain
arCOG00186	COG03845	-	R	ABC-type uncharacterized transport system, ATPase component
arCOG00257	COG01744	Med	R	Uncharacterized ABC-type transport system, periplasmic component/surface lipoprotein
arCOG00258	COG01744	Med	R	Uncharacterized ABC-type transport system, periplasmic component/surface lipoprotein
arCOG00259	COG04603	-	R	ABC-type uncharacterized transport system, permease component
arCOG00260	COG04603	-	R	ABC-type uncharacterized transport system, permease component
arCOG00261	COG01079	-	R	Uncharacterized ABC-type transport system, permease component
arCOG00281	COG00433		R	Predicted ATPase
arCOG00348	COG02047	-	R	Archaeal enzyme of ATP-grasp superfamily
arCOG00435	COG00714	-	R	MoxR-like ATPase
arCOG00441	COG00714	-	R	MoxR-like ATPase
arCOG00655	COG01427	-	R	Predicted periplasmic solute-binding protein
arCOG00691	COG01574	-	R	Predicted metal-dependent hydrolase with the TIM-barrel fold
arCOG00754	COG00579	-	R	Malate/quinone oxidoreductase or related dehydrogenase
arCOG00828	COG03393	-	R	Predicted acetyltransferase
arCOG00893	COG01831	-	R	Predicted metal-dependent hydrolase (urease superfamily)
arCOG00951	COG02100	-	R	Radical SAM superfamily enzyme
arCOG01144	COG01409	Icc	R	Predicted phosphohydrolase
arCOG01156	COG01408	-	R	Calcineurin-like phosphohydrolase
arCOG01210			R	Predicted nucleotidyltransferase and HTH domain
arCOG01211	COG01708	-	R	Minimal nucleotidyltransferase
arCOG01215	COG03769	-	R	HAD superfamily hydrolase
arCOG01229	COG02403	-	R	Predicted GTPase
arCOG01293	COG00446	HcaD	R	NAD(FAD)-dependent dehydrogenase
arCOG01294	COG00446	HcaD	R	NAD(FAD)-dependent dehydrogenase

arCOG01395	COG01817	-	R	Lipid-A-disaccharide synthase related glycosyltransferase
arCOG01565	COG00618	-	R	Exopolyphosphatase-related protein
arCOG01641	COG03269	-	R	Predicted RNA-binding protein, contains TRAM domain
arCOG01649	COG01073	-	R	Alpha/beta superfamily hydrolase
arCOG01651	COG01073	-	R	Alpha/beta superfamily hydrolase
arCOG01688	COG01719	-	R	Predicted transcriptional regulator, contains HTH and 4VR domain
arCOG01744	COG01988	-	R	Membrane-bound metal-dependent hydrolase
arCOG01801	COG02358	Imp	R	TRAP-type uncharacterized transport system, periplasmic component
arCOG01806			R	ACT domain containing protein
arCOG01848	COG00110	WbbJ	R	Acetyltransferase (isoleucine patch superfamily)
arCOG01860	COG01418	-	R	Predicted HD superfamily hydrolase
arCOG01906	COG04666	-	R	TRAP-type uncharacterized transport system, fused permease component
arCOG01963	COG03273	-	R	PhoU-like domain fused to TrkA-C domain
arCOG02016	COG01853	-	R	Conserved protein/domain typically associated with flavoprotein oxygenase, DIM6/NTAB family
arCOG02028	COG05012	-	R	Predicted cobalamin binding protein
arCOG02035	COG03894	-	R	Uncharacterized metal-binding protein
arCOG02105	COG01708	-	R	Minimal nucleotidyltransferase
arCOG02106	COG01708	-	R	Minimal nucleotidyltransferase
arCOG02112	COG01708	-	R	Minimal nucleotidyltransferase fused to HEPN domain
arCOG02164	COG01800	-	R	Predicted transglutaminase-like protease
arCOG02210	COG02244	RfbX	R	Polysaccharide biosynthesis protein, Mvin family
arCOG02214	COG02244	RfbX	R	Polysaccharide biosynthesis protein, Mvin family
arCOG02238	COG01342	-	R	Predicted DNA-binding protein
arCOG02240	COG01942	-	R	4-oxalocrotonate tautomerase related protein
arCOG02284	COG04880	-	R	Secreted protein containing C-terminal beta-propeller domain distantly related to WD-40 repeats
arCOG02292	COG00546	Gph	R	HAD superfamily hydrolase
arCOG02295	COG01011	-	R	HAD superfamily hydrolase
arCOG02303	COG00496	SurE	R	Predicted acid phosphatase
arCOG02465	COG01691	-	R	NCAIR mutase (PurE)-related protein
arCOG02578	COG00655	WrbA	R	Multimeric flavodoxin WrbA
arCOG02579	COG00727	-	R	Predicted Fe-S-cluster oxidoreductase
arCOG02584	COG00727	-	R	Predicted Fe-S-cluster oxidoreductase
arCOG02603	COG02018	-	R	Roadblock/LC7 domain
arCOG02616	COG00535	-	R	Radical SAM superfamily enzyme
arCOG02742	COG01721	-	R	Uncharacterized conserved protein, DUF58 family, contains vWF domain
arCOG02768	COG01266	-	R	Metal-dependent membrane protease, CAAX family
arCOG02828	COG03367	-	R	NAD dependent epimerase/dehydratase family
arCOG02902	COG02304	-	R	Uncharacterized protein containing a von Willebrand factor type A (vWA) domain
arCOG02959	COG02234	Iap	R	Predicted aminopeptidase
arCOG02986	COG01268	BioY	R	Uncharacterized conserved protein
arCOG02999	COG01917	-	R	Cupin domain containing protein
arCOG03045	COG00457	NrfG	R	TPR repeats containing protein
arCOG03408	COG01672	-	R	Predicted ATPase (AAA+ superfamily)
arCOG03409			R	Predicted ATPase (AAA+ superfamily)
arCOG03450			R	Predicted surface protease of transglutaminase family
arCOG03509	COG03291	-	R	Cell suface protein, containing PKD repeats
arCOG03647	COG02388	-	R	Acetyltransferase (GNAT) family
arCOG03723			R	HEPN domain
arCOG04052	COG04756	-	R	Predicted cation transporter
arCOG04065	COG00612	PqqL	R	Predicted Zn-dependent peptidase
arCOG04159	COG00714	-	R	MoxR-like ATPase
arCOG04210	COG00780	-	R	Enzyme related to GTP cyclohydrolase I

arCOG04212	COG01661	-	R	Predicted DNA-binding protein with PD1-like DNA-binding motif
arCOG04215	COG03375	-	R	Acetyltransferase (GNAT) family
arCOG04230	COG03294	-	R	HD supefamily hydrolase
arCOG04278	COG01679	-	R	Predicted aconitase
arCOG04284	COG01738	yhhQ	R	Uncharacterized member of the PurR regulon
arCOG04297	COG02401	-	R	ABC-type ATPase fused to a predicted acetyltransferase domain
arCOG04359	COG02517	-	R	Predicted RNA-binding protein containing a C-terminal EMAP domain
arCOG04410	COG01578	-	R	Predicted ATP-grasp domain fused to redox center
arCOG05120	COG05014	-	R	Radical SAM superfamily enzyme
arCOG05346			R	PP-loop superfamily ATPase fused to Zn-finger domain
arCOG05422			R	Predicted ATPase
arCOG05529	COG02118		R	DNA-binding TFAR19-related protein
arCOG05613			R	Predicted DEAD-box helicase/ATPase
arCOG05666	COG02908	-	R	Calcineurin-like phosphoesterase
arCOG06106	COG02514	-	R	Predicted ring-cleavage extradiol dioxygenase
arCOG06126	COG01811	-	R	Uncharacterized membrane protein, possible Na+ channel or pump
arCOG06897	COG03919	-	R	Predicted ATP-grasp enzyme
arCOG06982			R	ABC-type ATPase
arCOG07159	COG00637	-	R	HAD superfamily hydrolase
arCOG07435			R	HAD superfamily hydrolase
arCOG07448	COG01719		R	Predicted hydrocarbon binding protein, contains 4VR domain
arCOG07451	COG04821	-	R	Uncharacterized protein containing SIS (Sugar ISomerase) phosphosugar binding domain
arCOG07466	COG01266	-	R	Predicted metal-dependent membrane protease
arCOG07858	COG02244	RfbX	R	Polysaccharide biosynthesis protein, Mvin family
arCOG07859	COG01708	-	R	Minimal nucleotidyltransferase
arCOG08705	COG02373	-	R	Large extracellular alpha-helical protein
arCOG00022			S	Uncharacterized conserved protein
arCOG00372			S	Uncharacterized conserved protein
arCOG00379			S	Uncharacterized conserved protein
arCOG00398	COG02411	-	S	ASCH domain, predicted RNA-binding domain
arCOG00491			S	Uncharacterized conserved protein
arCOG00653			S	Uncharacterized conserved protein
arCOG00899	COG00392	-	S	Uncharacterized conserved membrane protein
arCOG00908	COG03286	-	S	Uncharacterized conserved protein
arCOG01048			S	Uncharacterized conserved protein
arCOG01330	COG02426	-	S	Predicted membrane protein
arCOG01466			S	Uncharacterized conserved protein
arCOG01472	COG02461	-	S	Hemerythrin HHE cation binding domain containing protein
arCOG01507	COG04904	-	S	Uncharacterized conserved protein
arCOG01644	COG02245	-	S	Predicted membrane protein
arCOG01724	COG04072	-	S	Uncharacterized conserved protein
arCOG01734	COG04933	-	S	Uncharacterized conserved protein
arCOG01766	COG02430	-	S	Uncharacterized conserved protein
arCOG01994	COG01300	SpoIIM	S	Uncharacterized membrane protein
arCOG02024	COG04881	-	S	Predicted membrane protein
arCOG02081	COG01470	-	S	Predicted membrane protein
arCOG02087	COG01470	-	S	Predicted membrane protein
arCOG02088			S	Uncharacterized conserved protein
arCOG02109	COG02445	-	S	HEPN domain containing protein
arCOG02123	COG01895	-	S	HEPN domain containing protein
arCOG02216			S	Uncharacterized conserved protein
arCOG02270	COG05428	-	S	Uncharacterized conserved small protein
arCOG02396			S	Uncharacterized conserved protein
arCOG02487			S	Cell suface protein
arCOG02488			S	Cell suface protein
arCOG02499	COG03420		S	NosD-like periplasmic protein

arCOG02532	COG03291	-	S	beta-propeller repeat protein fused to CARDB-like adhesion domain
arCOG02545	COG03291		S	NosD-like periplasmic protein fused to PKD repeat domain
arCOG02559			S	Cell suface protein
arCOG02701	COG01641	-	S	Uncharacterized conserved protein
arCOG02745	COG01721	-	S	Uncharacterized conserved protein, DUF58 family, contains vWF domain
arCOG02747	COG01721	-	S	Uncharacterized conserved protein, DUF58 family, contains vWF domain
arCOG03005	COG04997	-	S	Uncharacterized conserved protein, contains double-stranded beta-helix domain
arCOG03188			S	Uncharacterized conserved protein
arCOG03270			S	Uncharacterized conserved protein, contains DUF11 domain
arCOG03333	COG04273	-	S	DGC domain implicated in zinc binding
arCOG03349	COG01633	-	S	Ferritin-like domain
arCOG03427	COG02510	-	S	Predicted membrane protein
arCOG03442			S	Uncharacterized conserved membrane protein
arCOG03511	COG05306	-	S	Uncharacterized conserved protein
arCOG03733			S	Uncharacterized conserved protein
arCOG03754			S	Uncharacterized conserved protein
arCOG03755			S	paREP6 family protein
arCOG03776			S	Zn finger protein
arCOG03777			S	Zn finger protein
arCOG03794	COG03601	-	S	Predicted membrane protein
arCOG03829			S	Uncharacterized conserved protein
arCOG03835			S	Uncharacterized conserved membrane protein
arCOG03876			S	Uncharacterized conserved protein
arCOG03877			S	Uncharacterized conserved protein
arCOG03881			S	Uncharacterized conserved protein
arCOG03889	COG03465	-	S	Uncharacterized conserved protein
arCOG03911	COG03462	-	S	Predicted membrane protein
arCOG03942			S	Uncharacterized conserved protein
arCOG04002			S	Uncharacterized conserved protein
arCOG04008			S	Uncharacterized conserved protein
arCOG04016			S	Uncharacterized conserved protein
arCOG04024			S	Uncharacterized conserved protein
arCOG04037			S	Uncharacterized conserved protein
arCOG04058	COG01872	-	S	Uncharacterized conserved protein
arCOG04079			S	Uncharacterized conserved protein
arCOG04085	COG01649	-	S	Uncharacterized protein conserved, contains glutamine amidotransferase (GATase1)-like domain
arCOG04189	COG05625	-	S	Membrane protein
arCOG04264			S	Uncharacterized conserved protein, predicted prefoldin
arCOG04267			S	Uncharacterized conserved protein
arCOG04275			S	Uncharacterized conserved protein
arCOG04316			S	Uncharacterized conserved protein
arCOG04317	COG04732	-	S	Predicted membrane protein
arCOG04337	COG01504	-	S	Uncharacterized conserved protein
arCOG04344	COG04818	-	S	Predicted membrane protein
arCOG04354	COG01906	-	S	Uncharacterized conserved protein
arCOG04374	COG03804	-	S	Uncharacterized conserved protein related to dihydrodipicolinate reductase
arCOG04437	COG03863	-	S	Uncharacterized distant relative of cell wall-associated hydrolase
arCOG04467			S	Uncharacterized conserved protein
arCOG04477	COG01860	-	S	Uncharacterized conserved protein
arCOG04483	COG04739	-	S	Uncharacterized protein containing a ferredoxin domain
arCOG04488	COG02164	-	S	Uncharacterized conserved protein
arCOG04609			S	Predicted lipoprotein
arCOG04705	COG02098	-	S	Uncharacterized conserved protein
arCOG04797			S	Uncharacterized conserved protein
arCOG04807	COG01297	-	S	Predicted membrane protein
arCOG04849			S	Uncharacterized conserved protein

arCOG04938			S	Uncharacterized conserved protein
arCOG04939			S	Uncharacterized conserved protein
arCOG05025			S	Uncharacterized conserved protein
arCOG05079	COG01542	-	S	Uncharacterized conserved protein
arCOG05429			S	Uncharacterized conserved protein
arCOG05506			S	Uncharacterized conserved protein
arCOG05517			S	Uncharacterized conserved protein
arCOG05561			S	Uncharacterized conserved protein
arCOG05631			S	Uncharacterized conserved protein
arCOG05661			S	Uncharacterized conserved protein
arCOG05752	COG04720	-	S	Predicted membrane protein
arCOG06053			S	Uncharacterized conserved protein
arCOG06071			S	Uncharacterized conserved protein
arCOG06078			S	Uncharacterized conserved protein
arCOG06086			S	Uncharacterized conserved protein
arCOG06087			S	Uncharacterized conserved protein
arCOG06093			S	Uncharacterized conserved protein
arCOG06105			S	Uncharacterized conserved protein
arCOG06107			S	Uncharacterized conserved protein
arCOG06115			S	Uncharacterized conserved protein
arCOG06133			S	Uncharacterized conserved protein
arCOG06134			S	Uncharacterized conserved protein
arCOG06398			S	Uncharacterized conserved protein
arCOG06529			S	Uncharacterized conserved protein
arCOG06655			S	Uncharacterized conserved protein
arCOG06861			S	Uncharacterized conserved protein
arCOG06883			S	Zn-ribbon domain containing protein
arCOG06957			S	Uncharacterized conserved protein
arCOG06980	COG03864		S	vWFA domain containing protein
arCOG06985			S	Zn finger protein
arCOG06986			S	Uncharacterized conserved protein
arCOG07011	COG03600		S	Uncharacterized phage-associated protein
arCOG07069			S	Uncharacterized conserved protein
arCOG07070			S	Uncharacterized conserved protein
arCOG07086			S	Cell suface protein
arCOG07110	COG05617	-	S	Predicted integral membrane protein
arCOG07425			S	Uncharacterized conserved protein
arCOG07427			S	Uncharacterized conserved protein
arCOG07428			S	Uncharacterized conserved protein
arCOG07430	COG01814	-	S	Uncharacterized membrane protein
arCOG07453			S	Uncharacterized conserved protein
arCOG07455			S	Uncharacterized conserved protein
arCOG07458			S	Uncharacterized conserved protein
arCOG07460			S	Uncharacterized conserved protein
arCOG07465			S	Uncharacterized conserved protein
arCOG07487	COG04708	-	S	Predicted membrane protein
arCOG07488			S	Uncharacterized conserved protein
arCOG07489			S	Uncharacterized conserved protein
arCOG07491			S	Uncharacterized conserved protein
arCOG07492			S	Uncharacterized conserved protein
arCOG07510			S	Uncharacterized conserved protein
arCOG07571			S	Uncharacterized conserved membrane protein
arCOG07626			S	Uncharacterized conserved protein
arCOG07632			S	Uncharacterized conserved protein
arCOG07678			S	Uncharacterized conserved protein
arCOG07690			S	Uncharacterized conserved protein
arCOG07703			S	Uncharacterized conserved protein
arCOG07746			S	Uncharacterized conserved protein
arCOG07750			S	Uncharacterized conserved protein
arCOG07752			S	Uncharacterized conserved protein
arCOG07753			S	Uncharacterized conserved protein
arCOG07756			S	Uncharacterized conserved protein
arCOG07757			S	Uncharacterized conserved protein
arCOG07758			S	Uncharacterized conserved protein
arCOG07831			S	Uncharacterized conserved protein
arCOG07843			S	Uncharacterized conserved protein
arCOG07865			S	Uncharacterized conserved protein
arCOG08060			S	transmembrane protein
arCOG08088			S	Uncharacterized conserved protein

arCOG08215			S	Uncharacterized conserved protein
arCOG08221			S	Uncharacterized conserved protein
arCOG08303	COG02880	-	S	Uncharacterized conserved protein
arCOG08358			S	Uncharacterized conserved protein
arCOG08625	COG01904		S	Uncharacterized conserved protein
arCOG08629	COG03395	-	S	Uncharacterized conserved protein
arCOG08832			S	Uncharacterized conserved protein
arCOG08858			S	Uncharacterized conserved protein
arCOG08872			S	Uncharacterized conserved protein
arCOG09389			S	Uncharacterized conserved protein
arCOG09422	COG04720		S	Predicted membrane protein
arCOG09424			S	Uncharacterized conserved protein
arCOG09425			S	Uncharacterized conserved protein
arCOG09426			S	Uncharacterized conserved membrane protein
arCOG09429			S	Uncharacterized conserved protein
arCOG09431			S	Uncharacterized conserved protein
arCOG09435			S	Uncharacterized conserved membrane protein
arCOG09748			S	Uncharacterized conserved protein
arCOG09797			S	Uncharacterized conserved protein
arCOG09808			S	Uncharacterized conserved protein
arCOG09847			S	Uncharacterized conserved protein
arCOG09922			S	Uncharacterized conserved protein
arCOG10126	COG01422		S	Uncharacterized conserved protein
arCOG10149			S	Uncharacterized conserved protein
arCOG10150			S	Uncharacterized conserved protein
arCOG10161			S	Uncharacterized conserved protein
arCOG10167			S	Uncharacterized conserved protein
arCOG10177			S	Uncharacterized conserved protein
arCOG10276			S	Uncharacterized conserved protein
arCOG10286			S	Uncharacterized conserved protein
arCOG10288			S	Uncharacterized conserved protein
arCOG10417			S	Uncharacterized conserved protein
arCOG10423			S	Uncharacterized conserved protein
arCOG10428			S	Uncharacterized conserved protein
arCOG10481			S	Uncharacterized conserved protein
arCOG10611			S	Uncharacterized conserved protein
arCOG10656			S	Uncharacterized conserved protein
arCOG10860			S	Uncharacterized conserved protein
arCOG10867			S	Uncharacterized conserved protein
arCOG10888			S	Uncharacterized conserved protein
arCOG10919			S	Uncharacterized conserved protein
arCOG10940			S	Uncharacterized conserved protein
arCOG10989			S	Uncharacterized conserved protein
arCOG10990			S	Uncharacterized conserved protein
arCOG11052			S	Uncharacterized conserved protein
arCOG11053			S	Uncharacterized conserved protein
arCOG11063			S	Uncharacterized conserved protein
arCOG11224			S	Uncharacterized conserved protein
arCOG11225			S	Uncharacterized conserved protein
arCOG11243			S	Uncharacterized conserved protein
arCOG11244			S	Uncharacterized conserved protein
arCOG00449	COG00589	UspA	T	Nucleotide-binding protein, UspA family
arCOG01174	COG00467	RAD55	T	RecA-superfamily ATPase implicated in signal transduction
arCOG01694	COG01716	-	T	FHA domain and HTH domain
arCOG04078	COG02346		T	Globin-like domain
arCOG05302	COG00631	PTC1	T	Serine/threonine protein phosphatase
arCOG03383	COG00823	TolB	U	Periplasmic component of the Tol biopolymer transport system
arCOG00710	COG01848	-	V	PIN domain containing protein
arCOG00711	COG01848	-	V	PIN domain containing protein
arCOG00717	COG02405	-	V	PIN domain containing protein
arCOG00719	COG02405	-	V	PIN domain containing protein
arCOG00720	COG01458	-	V	PIN domain containing protein
arCOG00722	COG02886	-	V	Predicted antitoxins containing the HTH domain
arCOG00730	COG01848	-	V	PIN domain containing protein

	arCOG01437	COG05551	Cas6	V	CRISPR system related protein, RAMP superfamily Cas6 group
	arCOG01441		Cas8a2	V	CRISPR system related protein, large subunit of CASCADE complex
	arCOG01731	COG00534	NorM	V	Na+-driven multidrug efflux pump
	arCOG02121	COG05378	-	V	PIN domain containing protein
	arCOG02224	COG01487	VapC	V	PIN domain containing protein
	arCOG02672	COG01688	Cas5	V	CRISPR system related protein Cas5, RAMP superfamily
	arCOG03222	COG01567	Cas5/Csm4	V	CRISPR system related protein, RAMP superfamily Cas5 group
	arCOG03223	COG01567	Cas5/Csm4	V	CRISPR system related protein, RAMP superfamily Cas5 group
	arCOG03435	COG01517	-	V	CRISPR system related protein, COG1517 family
	arCOG03521	COG01002	-	V	Type II restriction enzyme, methylase subunit
	arCOG03590			V	Addiction module toxin, Txe/YoeB family
	arCOG03718	COG01332	Cas7/Csm5	V	CRISPR system related protein, RAMP superfamily Cas7 group
	arCOG03719	COG01332	Cas7/Csm5	V	CRISPR system related protein, RAMP superfamily Cas7 group
	arCOG03788	COG05608		V	LEA14-like dessication related protein
	arCOG03886			V	CopG family DNA-binding protein
	arCOG04193			V	CRISPR system associated protein, predicted small subunit of CASCADE complex
	arCOG05297	COG02402	-	V	PIN domain containing protein
	arCOG05724	COG02810	-	V	Predicted type IV restriction endonuclease
	arCOG06143			V	CRISPR system associated protein, predicted small subunit of CASCADE complex
	arCOG06144		Cas8a2	V	CRISPR system related protein, large subunit of CASCADE complex
	arCOG06987			V	CopG-family DNA-binding protein
	arCOG07131	COG01131	CcmA	V	ABC-type multidrug transport system, ATPase component
	arCOG07514			V	RHH/CopG family DNA-binding protein
	arCOG07641	COG01517		V	CRISPR system related protein, COG1517 family
	arCOG07759	COG01487	VapC	V	PIN domain containing protein
	arCOG07904	COG04634		V	PIN domain
	arCOG08616			V	Ribbon-helix-helix protein, copG family
	arCOG08881			V	Ribbon-helix-helix protein, copG family
	arCOG10147			V	CRISPR-associated endoribonuclease Cas6
**Gain inAcd1**					
	arCOG06057			C	Membrane-bound NiFe hydrogenase subunit
	arCOG00834	COG00454	WecD	K	Acetyltransferase (GNAT) family
	arCOG01242	COG00582	XerC	L	Integrase
	arCOG01373	COG01089	Gmd	M	GDP-D-mannose dehydratase
	arCOG01955	COG00475	KefB	P	Kef-type K+ transport system, membrane component fused TrkA K+ transport system
	arCOG06513	COG05485	-	R	Predicted ester cyclase
	arCOG07416	COG00596	MhpC	R	Alpha/beta superfamily hydrolase
	arCOG05099	COG02105		S	AIG2-like family protein
	arCOG06076			S	Uncharacterized conserved protein
	arCOG07199			S	Uncharacterized conserved protein
	arCOG07226			S	Uncharacterized conserved protein
	arCOG07348			S	Uncharacterized conserved protein
	arCOG07536	COG03055	-	S	Uncharacterized conserved protein
	arCOG08456			S	Uncharacterized conserved protein
	arCOG09897			S	Uncharacterized conserved protein
	arCOG10353			S	Uncharacterized conserved protein
	arCOG10437			S	Uncharacterized conserved protein
	arCOG11297			S	Uncharacterized conserved protein
	arCOG07245			S	Uncharacterized conserved protein
	arCOG02782	COG01787	-	V	Endonuclease, HJR/Mrr/RecB family
**Loss in Acd1**					
	arCOG00024	COG00554	GlpK	C	Glycerol kinase

arCOG00246	COG00039	Mdh	C	Malate/lactate dehydrogenase
arCOG00446	COG02086	FixA	C	Electron transfer flavoprotein, beta subunit
arCOG00447	COG02025	FixB	C	Electron transfer flavoprotein, alpha subunit
arCOG00701	COG00584	UgpQ	C	Glycerophosphoryl diester phosphodiesterase
arCOG00959	COG01146	-	C	Ferredoxin
arCOG00984	COG01454	EutG	C	Alcohol dehydrogenase, class IV
arCOG01004	COG02421	-	C	Predicted acetamidase/formamidase
arCOG01052	COG00022	AcoB	C	Pyruvate/2-oxoglutarate/acetoin dehydrogenase complex, dehydrogenase (E1) component
arCOG01054	COG01071	AcoA	C	Pyruvate/2-oxoglutarate/acetoin dehydrogenase complex, dehydrogenase (E1) component, eukaryotic type, alpha subunit
arCOG01067	COG01252	Ndh	C	NADH dehydrogenase, FAD-containing subunit
arCOG01235	COG01622	CyoA	C	Heme/copper-type cytochrome/quinol oxidase, subunit 2
arCOG01236	COG01622	CyoA	C	Heme/copper-type cytochrome/quinol oxidase, subunit 2
arCOG01237	COG00843	CyoB	C	Heme/copper-type cytochrome/quinol oxidase, subunit 1 and 3
arCOG01356	COG01032	-	C	Radical SAM superfamily enzyme
arCOG01458	COG00604	Qor	C	NADPH:quinone reductase or related Zn-dependent oxidoreductase
arCOG01495	COG00243	BisC	C	Molybdopterin oxidoreductase
arCOG01503	COG00437	HybA	C	Fe-S-cluster-containing dehydrogenase component
arCOG01602	COG01014	PorG	C	Pyruvate:ferredoxin oxidoreductase or related 2-oxoacid:ferredoxin oxidoreductase, gamma subunit
arCOG01604	COG01144	-	C	Pyruvate:ferredoxin oxidoreductase or related 2-oxoacid:ferredoxin oxidoreductase, gamma and delta subunit
arCOG01609	COG04231	-	C	Indolepyruvate ferredoxin oxidoreductase, alpha and beta subunit
arCOG01706	COG00508	AceF	C	Pyruvate/2-oxoglutarate dehydrogenase complex, dihydrolipoamide acyltransferase (E2) component or related enzyme
arCOG01984	COG02440	FixX	C	Ferredoxin-like protein
arCOG02026	COG05557	-	C	Polysulphide reductase
arCOG02236	COG01148	HdrA	C	Heterodisulfide reductase, subunit A or related polyferredoxin
arCOG02244	COG02009	SdhC	C	Succinate dehydrogenase/fumarate reductase, cytochrome b subunit
arCOG02304	COG01804	Mct/CaiB	C	Succinyl-CoA:mesaconate CoA-transferase or predicted acyl-CoA transferase/carnitine dehydratase or
arCOG02700	COG00508	AceF	C	Pyruvate/2-oxoglutarate dehydrogenase complex, dihydrolipoamide acyltransferase (E2) component (C-terminal fragment)
arCOG02772	COG00348	NapH	C	Polyferredoxin
arCOG04358	COG01526	FdhD	C	Uncharacterized protein required for formate dehydrogenase activity
arCOG04386	COG01620	LldP	C	L-lactate permease
arCOG04874	COG02055	-	C	Malate/L-lactate dehydrogenase
arCOG05498	COG02225	AceB	C	Malate synthase
arCOG05636	COG02864		C	Formate dehydrogenase, cytochrome b556 subunit
arCOG05865	COG01274	PckA	C	Phosphoenolpyruvate carboxykinase (GTP)
arCOG06033			C	Predicted subunit of heme/copper-type cytochrome/quinol oxidase
arCOG02235	COG01148	HdrA	C	Heterodisulfide reductase, subunit A or related polyferredoxin
arCOG00177	COG03842	PotA	E	ABC-type spermidine/putrescine transport system, ATPase component
arCOG00182	COG00444	DppD	E	ABC-type dipeptide/oligopeptide/nickel transport system, ATPase component
arCOG00204	COG04608	AppF	E	ABC-type oligopeptide transport system, ATPase component
arCOG00455	COG00345	ProC	E	Pyrroline-5-carboxylate reductase

arCOG00748	COG01173	DppC	E	ABC-type dipeptide/oligopeptide/nickel transport system, permease component
arCOG00915	COG00160	GabT	E	4-aminobutyrate aminotransferase or related aminotransferase
arCOG00924	COG00410	LivF	E	ABC-type branched-chain amino acid transport system, ATPase component
arCOG00925	COG00411	LivG	E	ABC-type branched-chain amino acid transport system, ATPase component
arCOG01021	COG00683	LivK	E	ABC-type branched-chain amino acid transport system, periplasmic component
arCOG01269	COG00559	LivH	E	Branched-chain amino acid ABC-type transport system, permease component
arCOG01273	COG04177	LivM	E	ABC-type branched-chain amino acid transport system, permease component
arCOG01292	COG00493	GltD	E	NADPH-dependent glutamate synthase beta chain or related oxidoreductase
arCOG01511	COG00145	HyuA	E	N-methylhydantoinase A/acetone carboxylase, beta subunit
arCOG01512	COG00146	HyuB	E	N-methylhydantoinase B/acetone carboxylase, alpha subunit
arCOG01533	COG00747	DdpA	E	ABC-type transport system, periplasmic component
arCOG01534	COG00747	DdpA	E	ABC-type transport system, periplasmic component
arCOG01535	COG00747	DdpA	E	ABC-type transport system, periplasmic component
arCOG02165	COG01305	-	E	Transglutaminase-like enzyme, putative cysteine protease
arCOG02462	COG01878	-	E	Kynurenine formamidase
arCOG03462	COG00531	PotE	E	Amino acid transporter
arCOG03466	COG00531	PotE	E	Amino acid transporter
arCOG03650	COG00531	PotE	E	Amino acid transporter
arCOG03651	COG00531	PotE	E	Amino acid transporter
arCOG03673			E	Thermopsin-like protease
arCOG04029	COG05550		E	Pepsin-like aspartate protease
arCOG04083	COG02355	-	E	Zn-dependent dipeptidase, microsomal dipeptidase homolog
arCOG04333	COG00436	-	E	Aspartate/tyrosine/aromatic aminotransferase
arCOG05121	COG02755	TesA	E	Lysophospholipase L1 or related esterase
arCOG03447	COG01457	CodB	F	Purine-cytosine permease or related protein
arCOG03448	COG01953	FUI1	F	Cytosine/uracil/thiamine/allantoin permease
arCOG03714	COG01328	NrdD	F	ATP cone domain
arCOG04128	COG00035	Upp	F	Uracil phosphoribosyltransferase
arCOG04173	COG00295	Cdd	F	Cytidine deaminase
arCOG05252	COG04741	-	F	Predicted secreted endonuclease distantly related to archaeal Holliday junction resolvase
arCOG00025	COG01070	XylB	G	Sugar (pentulose and hexulose) kinase
arCOG00150	COG01653	UgpB	G	ABC-type sugar transport system, periplasmic component
arCOG00153	COG01653	UgpB	G	ABC-type trehalose transport system, periplasmic component
arCOG00154	COG02182	MalE	G	Maltose-binding periplasmic protein
arCOG00156	COG01653	UgpB	G	ABC-type sugar transport system, periplasmic component
arCOG00157	COG01175	UgpA	G	ABC-type sugar transport system, permease component
arCOG00158	COG00395	UgpE	G	ABC-type sugar transport system, permease component
arCOG00159	COG00395	UgpE	G	ABC-type sugar transport system, permease component
arCOG00160	COG03833	MalG	G	ABC-type maltose transport system, permease component
arCOG00175	COG03839	MalK	G	ABC-type sugar transport system, ATPase component
arCOG00263	COG01172	AraH	G	Ribose/xylose/arabinose/galactoside ABC-type transport system, permease component
arCOG00581	COG02513	PrpB	G	PEP phosphonomutase or related enzyme

arCOG00699	COG01820	NagA	G	N-acetylglucosamine-6-phosphate deacetylase
arCOG00760	COG02301	Mcl/CitE	G	Beta-methylmalyl-CoA lyase (Citrate lyase beta subunit family)
arCOG01114	COG00574	PpsA	G	Phosphoenolpyruvate synthase/pyruvate phosphate dikinase
arCOG02685	COG00477	ProP	G	Permease of the major facilitator superfamily
arCOG02688	COG00477	ProP	G	Permease of the major facilitator superfamily
arCOG02792	COG00477	ProP	G	Permease of the major facilitator superfamily
arCOG03284	COG01449	-	G	Glycosyl hydrolase family 57
arCOG03661	COG00383	AMS1	G	Alpha-mannosidase
arCOG03663	COG01501	-	G	Alpha-glucosidase, family 31 of glycosyl hydrolase
arCOG03858			G	Glycosyl hydrolase family 12
arCOG03863	COG04354	-	G	Predicted bile acid beta-glucosidase
arCOG03867	COG04354	-	G	Predicted bile acid beta-glucosidase
arCOG04120	COG00469	PykF	G	Pyruvate kinase
arCOG04634	COG01472	BglX	G	Bifunctional b-D-xylosidase/a-L-arabinosidase or related glycosidase
arCOG05317	COG02721	UxaA	G	Altronate dehydratase
arCOG05318	COG02721		G	SAF domain, altronate dehydratase related
arCOG05341	COG00477	ProP	G	Permease of the major facilitator superfamily
arCOG05361	COG02017	GalM	G	Galactose mutarotase or related enzyme
arCOG05411	COG03934	-	G	Endo-beta-mannanase
arCOG05412	COG02723	BglB	G	Beta-glucosidase/6-phospho-beta-glucosidase/beta-galactosidase
arCOG06049	COG03345		G	Alpha-galactosidase
arCOG07337	COG03250	LacZ	G	Beta-galactosidase/beta-glucuronidase
arCOG07338	COG03669	-	G	Alpha-L-fucosidase
arCOG07728	COG01879	RbsB	G	ABC-type sugar transport system, periplasmic component, contains N-terminal xre family HTH domain
arCOG00217	COG00303	MoeA	H	Molybdopterin biosynthesis enzyme
arCOG00660	COG00320	LipA	H	Lipoate synthase
arCOG01870	COG01865	cbiZ	H	Adenosylcobinamide amidohydrolase
arCOG02620	COG01541	PaaK	H	Coenzyme F390 synthetase
arCOG02939	COG02154	-	H	Pterin-4a-carbinolamine dehydratase
arCOG00239	COG01024	CaiD	I	Enoyl-CoA hydratase/carnithine racemase
arCOG00774	COG02030	Mch	I	Mesaconyl-CoA hydratase (Acyl dehydratase family)
arCOG00856	COG00318	CaiC	I	Acyl-CoA synthetase (AMP-forming)/AMP-acid ligase II
arCOG00860	COG01608	-	I	Isopentenyl phosphate kinase, enzyme of modified mevalonate pathway
arCOG01259	COG01028	FabG	I	Short-chain alcohol dehydrogenase
arCOG01279	COG00183	PaaJ	I	Acetyl-CoA acetyltransferase
arCOG01280	COG00183	PaaJ	I	Acetyl-CoA acetyltransferase
arCOG01842	COG03255	-	I	Putative sterol carrier protein
arCOG01987	COG01788	AtoD	I	Acyl CoA:acetate/3-ketoacid CoA transferase, alpha subunit
arCOG02638	COG00657	Aes	I	Esterase/lipase
arCOG03056	COG00671	PgpB	I	Membrane-associated phospholipid phosphatase
arCOG04100	COG01960	CaiA	I	Acyl-CoA dehydrogenase
arCOG04201	COG00365	Acs	I	Acyl-coenzyme A synthetase/AMP-(fatty) acid ligase
arCOG04310	COG01960	CaiA	I	Acyl-CoA dehydrogenase
arCOG04350	COG01960	CaiA	I	Acyl-CoA dehydrogenase
arCOG05316	COG02057	AtoA	I	Acyl CoA:acetate/3-ketoacid CoA transferase, beta subunit
arCOG06112	COG00365	Acs	I	Acyl-coenzyme A synthetase/AMP-(fatty) acid ligase
arCOG07204	COG02267	PldB	I	Lysophospholipase, alpha-beta hydrolase superfamily
arCOG07315	COG01502		I	Phospholipase D/Transphosphatidylase
arCOG00995	COG01549	-	J	Queuine tRNA-ribosyltransferase related protein

arCOG00996	COG00343	Tgt	J	Queuine/archaeosine tRNA-ribosyltransferase
arCOG01013	COG04352	-	J	Ribosomal protein L13E
arCOG04168	COG02174	RPL34A	J	Ribosomal protein L34E
arCOG00001	COG01695	-	K	Predicted transcriptional regulator, PadR family
arCOG00374	COG02512	-	K	Predicted membrane-associated trancriptional regulator
arCOG00394	COG00640	ArsR	K	Transcriptional regulator containing HTH domain, ArsR family
arCOG00608	COG03620	-	K	Predicted transcriptional regulator with C-terminal CBS domains
arCOG00732	COG01846	MarR	K	Transcriptional regulator, MarR family
arCOG00816	COG02002	AbrB	K	Transcriptional regulator AbrB
arCOG00818	COG02002	AbrB	K	Transcriptional regulator AbrB
arCOG00820	COG02002	AbrB	K	Transcriptional regulator AbrB
arCOG00825	COG02002	AbrB	K	Transcriptional regulator AbrB
arCOG00826	COG00454	WecD	K	Acetyltransferase (GNAT) family
arCOG00877	COG01061	SSL2	K	DNA or RNA helicase of superfamily II
arCOG01057	COG01733	-	K	Predicted transcriptional regulator
arCOG01128	COG00819	TenA	K	Transcriptional activator TenA
arCOG01129	COG00819	TenA	K	Transcriptional activator TenA
arCOG01361	COG01243	ELP3	K	Histone acetyltransferase
arCOG01665	COG02026	RelE	K	Cytotoxic translational repressor of toxin-antitoxin stability system
arCOG01681	COG00640	ArsR	K	Transcriptional regulator containing HTH domain, ArsR family
arCOG01804	COG03888	-	K	Predicted transcriptional regulator
arCOG02217			K	Predicted antitoxin associated with PIN domain
arCOG02272	COG03413	-	K	Predicted transcriptional regulator, C-terminal HTH-like domain
arCOG03358	COG03355	-	K	Predicted transcriptional regulator
arCOG03748	COG01846	MarR	K	Transcriptional regulator, MarR family
arCOG03859	COG00640	ArsR	K	Transcriptional regulator containing HTH domain, ArsR family
arCOG04056	COG00640	ArsR	K	Transcriptional regulator containing HTH domain, ArsR family
arCOG04248	COG00846	SIR2	K	NAD-dependent protein deacetylase, SIR2 family
arCOG04362	COG04742	-	K	Predicted transcriptional regulator
arCOG05969	COG03355	-	K	Predicted transcriptional regulator
arCOG06044	COG01802	GntR	K	Transcriptional regulators
arCOG00115	COG00863	-	L	DNA modification methylase
arCOG00558	COG00513	SrmB	L	Superfamily II DNA and RNA helicase
arCOG00890	COG00863	-	L	DNA modification methylase
arCOG01244	COG04342	-	L	Intergrase/Recombinase
arCOG01247	COG04974	XerD	L	Site-specific recombinase XerD
arCOG01290	COG01533	SplB	L	DNA repair photolyase
arCOG01304	COG01637	NucS	L	RecB family endonuclease acting on branched DNA substrates
arCOG01442	COG02254	-	L	HD superfamily nuclease
arCOG01443	COG02254	Cas3''	L	HD superfamily nuclease
arCOG02129	COG03335	-	L	Transposase
arCOG02759	COG01943	-	L	Transposase
arCOG02942	COG00328	RnhA	L	Ribonuclease HI
arCOG03162	COG01961	PinR	L	Site-specific recombinase, DNA invertase Pin homolog
arCOG04032			L	Eukaryotic-type DNA primase, catalytic (small) subunit homolog, fused to HTH domain
arCOG04356	COG03372	Bax1	L	Nuclease of restriction endonuclease-like fold, implicated in nucleotide excision repair
arCOG07735			L	HNH superfamily endonuclease
arCOG08402			L	Transposase DNA-binding domain
arCOG00059	COG02222	AgaS	M	Predicted phosphosugar isomerase
arCOG00253	COG01004	Ugd	M	Predicted UDP-glucose 6-dehydrogenase
arCOG00665	COG01210	GalU	M	UDP-glucose pyrophosphorylase
arCOG00894	COG00463	WcaA	M	Glycosyltransferase
arCOG01168	COG04948	-	M	L-alanine-DL-glutamate epimerase or related enzyme of enolase superfamily

arCOG01381	COG00463	WcaA	M	Glycosyltransferase
arCOG01398	COG00463	WcaA	M	Glycosyltransferase
arCOG01403	COG00438	RfaG	M	Glycosyltransferase
arCOG01407	COG00438	RfaG	M	Glycosyltransferase
arCOG01408	COG00438	RfaG	M	Glycosyltransferase
arCOG01409	COG00438	RfaG	M	Glycosyltransferase
arCOG01417	COG00438	RfaG	M	Glycosyltransferase
arCOG07240	COG00463	WcaA	M	Glycosyltransferase
arCOG07450			M	O-Glycosyl hydrolase family 16
arCOG01809	COG01955	FlaJ	N	Archaeal flagella assembly protein J
arCOG01822	COG03354	FlaG	N	Putative archaeal flagellar protein G
arCOG01824	COG03353	FlaF	N	Putative archaeal flagellar protein F
arCOG01829	COG01681	FlaB	N	Archaeal flagellins
arCOG03872			N	Flagellar protein G
arCOG04148	COG02874	FlaH	N	Predicted ATPase involved in biogenesis of archaeal flagella
arCOG01231	COG00378	HypB	O	Ni2+-binding GTPase involved in regulation of expression and maturation of urease and hydrogenase
arCOG01333	COG00501	HtpX	O	Zn-dependent protease with chaperone function
arCOG01334	COG00501	HtpX	O	Zn-dependent protease with chaperone function
arCOG01910	COG01030	NfeD	O	Short-chain alcohol dehydrogenase
arCOG01912	COG01585	-	O	Membrane protein implicated in regulation of membrane protease activity
arCOG01915	COG00330	HflC	O	Membrane protease subunit, stomatin/prohibitin homolog
arCOG02833	COG00265	DegQ	O	Serine protease Do (heat-shock protein)
arCOG03580	COG02020	STE14	O	Putative protein-S-isoprenylcysteine methyltransferase
arCOG03743	COG05550	-	O	Predicted aspartyl protease
arCOG04028	COG05550	-	O	Predicted aspartyl protease
arCOG04236	COG00396	sufC	O	Cysteine desulfurase activator ATPase
arCOG05850	COG02039	Pcp	O	Pyrrolidone-carboxylate peptidase (N-terminal pyroglutamyl peptidase)
arCOG07052	COG00425		O	Predicted redox protein, regulator of disulfide bond formation
arCOG00188	COG01122	CbiO	P	ABC-type cobalt transport system, ATPase component
arCOG00576	COG00428	-	P	Predicted divalent heavy-metal cations transporter
arCOG00625	COG02239	MgtE	P	Mg/Co/Ni transporter MgtE (contains CBS domain)
arCOG01477	COG01230	CzcD	P	Co/Zn/Cd efflux system component
arCOG01478	COG00053	MMT1	P	Predicted Co/Zn/Cd cation transporter
arCOG01585	COG01522	Lrp	P	Transcriptional regulator (Lrp/AsnC family)
arCOG01956	COG00475	KefB	P	Kef-type K+ transport system, membrane component fused to CBS domains
arCOG01958	COG01226	Kch	P	Kef-type K+ transport system, predicted NAD-binding component
arCOG01969	COG00475	KefB	P	Kef-type K+ transport system, membrane component
arCOG02019	COG02897	SseA	P	Rhodanese-related sulfurtransferase
arCOG02250	COG00619	CbiQ	P	ABC-type cobalt transport system, permease component CbiQ or related transporter
arCOG02569	COG00038	EriC	P	Chloride channel protein EriC
arCOG02764	COG02608	CopZ	P	Copper-ion-binding protein
arCOG02854	COG02146	{NirD}	P	Ferredoxin subunit of nitrite reductase or ring-hydroxylating dioxygenase
arCOG04191	COG02046	MET3	P	ATP sulfurylase (sulfate adenylyltransferase)
arCOG04703	COG03256	NorB	P	Nitric oxide reductase large subunit
arCOG05372	COG01121	ZnuC	P	ABC-type Mn/Zn transport system, ATPase component
arCOG00696	COG01228	HutI	Q	Imidazolonepropionase or related amidohydrolase
arCOG01401	COG00500		Q	SAM-dependent methyltransferase
arCOG01402	COG00500		Q	SAM-dependent methyltransferase
arCOG01790	COG00500		Q	SAM-dependent methyltransferase
arCOG02004	COG03473	-	Q	Maleate cis-trans isomerase

arCOG04489	COG04689	-	Q	Acetoacetate decarboxylase related enzyme
arCOG04583	COG00500		Q	SAM-dependent methyltransferase
arCOG00589	COG00455		R	CobQ/CobB/MinD/ParA ATPase
arCOG00185	COG01123	-	R	ATPase component of various ABC-type transport system, contain duplicated ATPase
arCOG00236	COG03970	-	R	Fumarylacetoacetate (FAA) hydrolase family protein
arCOG00266	COG02041	-	R	Sulfite oxidase or related enzyme
arCOG00434	COG00714	-	R	MoxR-like ATPase
arCOG00498	COG00491	GloB	R	Metal-dependent hydrolase of the beta-lactamase superfamily II
arCOG00505	COG00491	GloB	R	Metal-dependent hydrolase of the beta-lactamase superfamily II
arCOG00606	COG00517	-	R	CBS domain
arCOG00654	COG02107	-	R	Predicted periplasmic solute-binding protein
arCOG00837	COG00456	RimI	R	Acetyltransferase (GNAT) family
arCOG00938	COG00535	-	R	Radical SAM superfamily enzyme
arCOG01093	COG02406	-	R	Protein distantly related to bacterial ferritins
arCOG01108	COG01473	AbgB	R	Metal-dependent amidase/aminoacylase/carboxypeptidase
arCOG01137	COG00824	FcbC	R	Thioesterase superfamily enzyme
arCOG01141	COG00622	-	R	Phosphoesterase
arCOG01149			R	Calcineurin-like phosphoesterase
arCOG01195	COG01708	-	R	Minimal nucleotidyltransferase
arCOG01197	COG01708	-	R	Minimal nucleotidyltransferase
arCOG01202	COG01708	-	R	Minimal nucleotidyltransferase
arCOG01205	COG01708	-	R	Minimal nucleotidyltransferase
arCOG01283	COG01545	-	R	Predicted nucleic-acid-binding protein containing a Zn-ribbon
arCOG01287	COG01545	-	R	Predicted nucleic-acid-binding protein containing a Zn-ribbon
arCOG01288	COG01545	-	R	OB-fold domain and Zn-ribbon containing protein, possible acyl-CoA-binding protein
arCOG01383	COG01216	-	R	Predicted glycosyltransferase
arCOG01422	COG05493	-	R	RecB-like nuclease fused to coiled-coil domain
arCOG01424			R	RecB-nuclease fused to coiled-coil domain
arCOG01425			R	RecB family nuclease with coiled-coil N-terminal domain
arCOG01426			R	RecB family nuclease with coiled-coil N-terminal domain, paREP7 family protein
arCOG01506	COG03381	TorD	R	Uncharacterized component of anaerobic dehydrogenase
arCOG01618	COG00656	ARA1	R	Aldo/keto reductase, related to diketogulonate reductase
arCOG01622	COG00673	MviM	R	Predicted dehydrogenase
arCOG01852	COG02151	PaaD	R	Predicted metal-sulfur cluster biosynthetic enzyme
arCOG01930	COG02159	-	R	Predicted metal-dependent hydrolase of the TIM-barrel fold
arCOG01931	COG02159	-	R	Predicted metal-dependent hydrolase of the TIM-barrel fold
arCOG01937	COG01750	-	R	Archaeal serine protease
arCOG01982	COG00434	SgcQ	R	Predicted TIM-barrel enzyme
arCOG02017	COG01853	-	R	Conserved protein/domain typically associated with flavoprotein oxygenase, DIM6/NTAB family
arCOG02110	COG01708	-	R	Minimal nucleotidyltransferase
arCOG02431	COG01611	-	R	Predicted Rossmann fold nucleotide-binding protein
arCOG02586	COG00727	-	R	Predicted Fe-S-cluster oxidoreductase
arCOG02935	COG01741	-	R	Pirin-related protein
arCOG03166	COG01672	-	R	AAA+ superfamily ATPase fused to HTH nad RecB nuclease domains
arCOG03168	COG01373	-	R	Predicted ATPase (AAA+ superfamily)
arCOG03237	COG01106	-	R	ATPase, predicted component of phage defense system
arCOG03850	COG04122		R	Predicted O-methyltransferase
arCOG04004	COG00666	Arp	R	Ankyrin repeats containing protein

arCOG04082	COG02366	-	R	Protein related to penicillin acylase
arCOG04197			R	Transposase related protein
arCOG04204	COG03342	-	R	N-terminal nucleophile hydrolase superfamily protein
arCOG04225	COG02110	-	R	Predicted phosphatase homologous to the C-terminal domain of histone macroH2A1
arCOG04285	COG02079	PrpD	R	Uncharacterized protein involved in propionate catabolism
arCOG04328	COG04028	-	R	Predicted P-loop ATPase/GTPase
arCOG04756	COG00679	-	R	Predicted permease
arCOG05331	COG02391	-	R	Predicted transporter component
arCOG05632	COG02391	-	R	Predicted transporter component
arCOG06914	COG03378	-	R	Phage associated DNA primase
arCOG07054	COG03193	GlcG	R	Uncharacterized protein, possibly involved in utilization of glycolate and propanediol
arCOG07331	COG02433	-	R	Uncharacterized conserved protein
arCOG07501	COG01994	SpoIVFB	R	Zn-dependent protease
arCOG07957	COG02342	-	R	Uncharacterized TIM barrel protein
arCOG07960	COG02607	-	R	ATPase (AAA+ superfamily)
arCOG02209	COG02244	RfbX	R	Polysaccharide biosynthesis protein, Mvin family
arCOG00385			S	Uncharacterized conserved protein
arCOG00723	COG05483	-	S	Uncharacterized conserved protein
arCOG00761	COG01720	-	S	Uncharacterized conserved protein
arCOG01119	COG04274	-	S	GYD domain, alpha/beta barrel superfamily
arCOG01314			S	Uncharacterized conserved membrane protein
arCOG01471	COG03945	-	S	Hemerythrin HHE cation binding domain containing protein
arCOG01668	COG03370	-	S	Uncharacterized conserved protein
arCOG01917			S	Zn-ribbon domain containing protein
arCOG02008	COG03371	-	S	Predicted membrane protein
arCOG02113	COG02427	-	S	Uncharacterized conserved protein
arCOG02149	COG00599	-	S	Uncharacterized homolog of gamma-carboxymuconolactone decarboxylase subunit
arCOG02287	COG00393	-	S	Uncharacterized beta/alpha-propeller protein, likely forms oligomers
arCOG02527	COG03291	-	S	PKD repeats containing protein
arCOG02556	COG01520	-	S	WD40-like repeat
arCOG02562	COG03391	-	S	Beta-propeller repeat containing protein
arCOG02739	COG01433	-	S	NifX family protein
arCOG02835	COG02324	-	S	Uncharacterized conserved membrane protein
arCOG02884	COG04743	-	S	Predicted membrane protein
arCOG02887	COG04743	-	S	Predicted membrane protein
arCOG03176	COG01584	-	S	Predicted membrane protein
arCOG03384	COG04946	-	S	Uncharacterized protein related to the periplasmic component of the Tol biopolymer transport system
arCOG03460	COG02120	-	S	Uncharacterized protein, LmbE homolog
arCOG03704			S	Uncharacterized conserved protein
arCOG03724			S	KID-like repeats conatining protein, paREP15 family
arCOG03749	COG04243	-	S	Predicted membrane protein
arCOG03769			S	Uncharacterized conserved membrane protein
arCOG03824			S	Uncharacterized conserved protein
arCOG03841	COG03535	-	S	Uncharacterized conserved protein
arCOG04017			S	Uncharacterized conserved protein
arCOG04025			S	Uncharacterized conserved protein
arCOG04033			S	Uncharacterized conserved protein
arCOG04039			S	Uncharacterized conserved protein
arCOG04132	COG04697	-	S	Uncharacterized conserved protein
arCOG04216			S	Uncharacterized conserved protein
arCOG04394	COG01416	-	S	Uncharacterized conserved protein
arCOG04474			S	Uncharacterized conserved protein
arCOG04811	COG02383	-	S	Uncharacterized conserved membrane protein, Fun14 family
arCOG05127			S	Uncharacterized conserved protein
arCOG05371			S	Uncharacterized conserved protein

arCOG05400			S	Protein containing potental lipid transfer domain, START superfamily
arCOG05472			S	Uncharacterized conserved protein
arCOG05906			S	Uncharacterized conserved protein
arCOG05908	COG01633	-	S	Ferritin-like domain
arCOG06019			S	Uncharacterized conserved protein
arCOG06043			S	Uncharacterized conserved protein
arCOG06052			S	Uncharacterized conserved protein
arCOG06085			S	Uncharacterized conserved protein
arCOG06103			S	Uncharacterized conserved protein
arCOG06975			S	Uncharacterized conserved protein
arCOG07053			S	Uncharacterized conserved memrane protein
arCOG07182			S	Uncharacterized conserved protein
arCOG07221			S	Zn-finger protein
arCOG07247			S	Uncharacterized conserved protein
arCOG07288			S	Uncharacterized conserved protein
arCOG07304			S	Uncharacterized conserved protein
arCOG07332			S	Uncharacterized conserved protein
arCOG07334			S	Uncharacterized conserved protein
arCOG07347			S	Uncharacterized conserved protein
arCOG07461			S	Uncharacterized conserved protein
arCOG07493			S	Uncharacterized conserved protein
arCOG07520	COG02442	-	S	Uncharacterized conserved protein
arCOG07527			S	Predicted metal-binding protein
arCOG07930			S	Zinc finger C2H2-type
arCOG08554			S	Uncharacterized conserved protein
arCOG08585			S	Uncharacterized conserved protein
arCOG09701			S	Uncharacterized conserved protein
arCOG09893			S	Uncharacterized conserved protein
arCOG09902			S	Uncharacterized conserved protein
arCOG09905			S	Uncharacterized conserved protein
arCOG10174			S	Uncharacterized conserved protein
arCOG10215			S	Uncharacterized conserved protein
arCOG10434			S	Uncharacterized conserved protein
arCOG10508			S	Uncharacterized conserved protein
arCOG11038			S	Uncharacterized conserved protein
arCOG04325	COG01702	PhoH	T	Phosphate starvation-inducible protein PhoH, predicted ATPase
arCOG04035	COG03451	VirB4	U	Type IV secretory pathway, VirB4 component
arCOG07496	COG03451	VirB4	U	Type IV secretory pathway, VirB4 component
arCOG00726	COG04113	-	V	PIN domain containing protein
arCOG00727	COG04113	-	V	PIN domain containing protein
arCOG00786	COG01468	Cas4	V	CRISPR-associated protein Cas4, RecB family exonuclease
arCOG01008	COG00864	NikR	V	Transcriptional regulator, CopG/Arc/MetJ family (DNA-binding and a metal-binding domains)
arCOG01199	COG01708	-	V	Minimal nucleotidyltransferase
arCOG01439	COG05551	Cas6	V	CRISPR system related protein, RAMP superfamily Cas6 group
arCOG01444	COG01203	Cas3	V	CRISPR-associated helicase Cas3
arCOG01465	COG00842	-	V	ABC-type multidrug transport system, permease component
arCOG01468	COG00842	-	V	ABC-type multidrug transport system, permease component
arCOG01469	COG00842	-	V	ABC-type multidrug transport system, permease component
arCOG01935	COG04006	-	V	CRISPR system related protein, COG1517 family
arCOG02222	COG01848	-	V	PIN domain containing protein
arCOG02654	COG03337	-	V	CRISPR system related protein, small subunit of CASCADE complex
arCOG02656	COG03337	-	V	CRISPR system related protein, small subunit of CASCADE complex
arCOG02659	COG01337	Cas7/Csm3	V	CRISPR system related protein, RAMP superfamily Cas7 group
arCOG02660	COG01337	Cas7/Csm3	V	CRISPR system related protein, RAMP superfamily Cas7 group

	arCOG02665	COG01769	Cas5/Cmr3	V	CRISPR system related protein, RAMP superfamily Cas5 group
	arCOG02666	COG01353	Cas10	V	CRISPR associated protein, polymerase-related domain and HD family nuclease
	arCOG02670	COG01688	Cas5	V	CRISPR system related protein Cas5, RAMP superfamily
	arCOG02731	COG01487	VapC	V	PIN domain containing protein
	arCOG02841	COG01132	MdlB	V	ABC-type multidrug transport system, ATPase and permease component
	arCOG03433	COG01517	-	V	CRISPR system related protein, COG1517 family
	arCOG03617	COG01857	Cas7	V	CRISPR system related protein Cas7, RAMP superfamily
	arCOG03706	COG01848		V	PIN domain containing protein
	arCOG03707			V	HEPN domain
	arCOG03722			V	HEPN domain
	arCOG03823			V	CRISPR system associated protein, predicted small subunit of CASCADE complex
	arCOG03847	COG01517		V	CRISPR system related protein, COG1517 family
	arCOG04194	COG01343	Cas2	V	CRISPR-associated protein Cas2
	arCOG04195	COG04343	-	V	CRISPR-associated protein, RecB family exonuclease
	arCOG05414			V	CopG/RHH family DNA binding protein, antitoxin
	arCOG05545			V	PIN domain containing protein
	arCOG06020	COG02250		V	HEPN domain
	arCOG06028	COG01487	VapC	V	PIN domain containing protein
	arCOG06164			V	Inactivated PIWI domain containing phage-defense protein
	arCOG07235			V	RHH/CopG DNA binding protein
	arCOG07256			V	CRISPR-associated protein
	arCOG07723			V	RHH/CopG DNA binding protein
	arCOG08086	COG01848		V	PIN domain containing protein
	arCOG08357			V	CopG/RHH family DNA binding protein
**Gain in other Sulfolobales**					
	arCOG00341	COG00277	GlcD	C	FAD/FMN-containing dehydrogenase
	arCOG00448	COG02086	FixA	C	Electron transfer flavoprotein, alpha and beta subunits
	arCOG00583	COG02224	AceA	C	Isocitrate lyase
	arCOG00615	COG01902	NemA	C	NADH:flavin oxidoreductase, Old Yellow Enzyme family
	arCOG00753	COG00578	GlpA	C	Glycerol-3-phosphate dehydrogenase
	arCOG01985	COG02440	FixX	C	Ferredoxin-like protein
	arCOG03700		SoxE	C	Sulfocyanin
	arCOG06967			C	Sulphur oxygenase/reductase
	arCOG07293	COG01192	Soj	D	ATPase involved in chromosome partitioning, ParA family
	arCOG00124	COG00175	CysH	E	3'-phosphoadenosine 5'-phosphosulfate sulfotransferase (PAPS reductase)/FAD synthetase or related enzyme
	arCOG00698	COG00804	UreC	E	Urea amidohydrolase (urease) alpha subunit
	arCOG00759	COG00665	DadA	E	Glycine/D-amino acid oxidase (deaminating)
	arCOG01109	COG00624	ArgE	E	Acetylornithine deacetylase/Succinyl-diaminopimelate desuccinylase or related deacylase
	arCOG01647	COG01505	-	E	Serine protease of the peptidase family S9A
	arCOG01673	COG00747	DdpA	E	ABC-type transport system, periplasmic component
	arCOG01730	COG03384	-	E	Aromatic ring-opening dioxygenase, catalytic LigB subunit related enzyme
	arCOG02001	COG00028	IlvB	E	Acetolactate synthase large subunit or other thiamine pyrophosphate-requiring enzyme
	arCOG03108	COG02235	ArcA	E	Arginine deiminase
	arCOG03463	COG00531	PotE	E	Amino acid transporter
	arCOG03464	COG00531	PotE	E	Amino acid transporter
	arCOG03649	COG00531	PotE	E	Amino acid transporter

arCOG03656	COG00028		E	Thiamine pyrophosphate-requiring enzyme, carboxylase
arCOG04527	COG00832	UreB	E	Urea amidohydrolase (urease) beta subunit
arCOG04528	COG00831	UreA	E	Urea amidohydrolase (urease) gamma subunit
arCOG05410	COG00444	DppD	E	ABC-type dipeptide/oligopeptide/nickel transport system, ATPase component
arCOG06037	COG00346	GloA	E	Catechol 2,3-dioxygenase or other lactoylglutathione lyase family enzyme
arCOG07260	COG03191	DmpA	E	L-aminopeptidase/D-esterase
arCOG00090	COG00518	GuaA	F	GMP synthase - Glutamine amidotransferase domain
arCOG00697	COG00402	SsnA	F	Cytosine deaminase or related metal-dependent hydrolase
arCOG01325	COG00813	DeoD	F	Purine-nucleoside phosphorylase
arCOG00135	COG00477	ProP	G	Permease of the major facilitator superfamily
arCOG00140	COG00477	ProP	G	Permease of the major facilitator superfamily
arCOG01896	COG01082	IolE	G	Sugar phosphate isomerase/epimerase
arCOG01902	COG01082	IolE	G	Sugar phosphate isomerase/epimerase
arCOG01999	COG00028		G	Pyruvate oxidase
arCOG02684	COG00477	ProP	G	Permease of the major facilitator superfamily
arCOG02690	COG00477	ProP	G	Permease of the major facilitator superfamily
arCOG02692	COG00477	ProP	G	Permease of the major facilitator superfamily
arCOG02693	COG00477	ProP	G	Permease of the major facilitator superfamily
arCOG02794	COG00477	ProP	G	Permease of the major facilitator superfamily
arCOG02949	COG01523	PulA	G	Bifunctional 4-alpha-glucanotransferase/ amylo-alpha-1,6-glucosidase
arCOG02951	COG00296	GlgB	G	1,4-alpha-glucan branching enzyme
arCOG02955	COG03280	TreY	G	Maltooligosyl trehalose synthase
arCOG03286	COG03387	SGA1	G	Glycosyl hydrolase family 15
arCOG03864	COG04354	-	G	Predicted bile acid beta-glucosidase
arCOG03865	COG04354	-	G	Predicted bile acid beta-glucosidase
arCOG04431	COG00580	GlpF	G	Glycerol uptake facilitator or related permease (Major Intrinsic Protein Family)
arCOG05344	COG00477	ProP	G	Permease of the major facilitator superfamily
arCOG05370	COG03386	-	G	Gluconolactonase
arCOG06047	COG00477	ProP	G	Permease of the major facilitator superfamily
arCOG00646	COG00007	CysG	H	Uroporphyrinogen-III methylase
arCOG05415			H	Carotene hydroxylase
arCOG06031	COG05424	-	H	Pyrroloquinoline quinone (Coenzyme PQQ) biosynthesis protein C
arCOG01262	COG01028	FabG	I	Short-chain alcohol dehydrogenase
arCOG01281	COG00183	PaaJ	I	Acetyl-CoA acetyltransferase
arCOG02936	COG01562	ERG9	I	Phytoene/squalene synthetase
arCOG05964	COG01960	CaiA	I	Acyl-CoA dehydrogenase
arCOG05965	COG01960	CaiA	I	Acyl-CoA dehydrogenase
arCOG06051	COG01960	CaiA	I	Acyl-CoA dehydrogenase
arCOG06122	COG00365	Acs	I	Acyl-coenzyme A synthetase/AMP-(fatty) acid ligase
arCOG07349	COG01670	RimL	J	Acetyltransferase, RimL family
arCOG00871	COG00553	HepA	K	Superfamily II DNA/RNA helicase, SNF2 family
arCOG00883	COG00553	HepA	K	Superfamily II DNA/RNA helicase, SNF2 family
arCOG01586	COG01522	Lrp	K	Transcriptional regulator (Lrp/AsnC family)
arCOG01587	COG01522	Lrp	K	Transcriptional regulator (Lrp/AsnC family)
arCOG01905	COG04189	-	K	Predicted transcriptional regulator
arCOG02273	COG03413	-	K	Predicted transcriptional regulator, C-terminal HTH-like domain
arCOG02647	COG01309	AcrR	K	Transcriptional regulator, TetR/AcrR family
arCOG03181	COG01846	MarR	K	Transcriptional regulator, MarR family
arCOG03423	COG01846	MarR	K	Transcriptional regulator, MarR family
arCOG04027	COG01725	-	K	Transcriptional regulator (GntR family)

arCOG07233	COG03327	PaaX	K	Phenylacetic acid-responsive transcriptional repressor
arCOG07251			K	Predicted transcriptional regulator
arCOG07311			K	Predicted transcriptional regulator, HTH domain
arCOG07935	COG01475	Spo0J	K	Chromosome partitioning protein ParB
arCOG08306	COG01497	-	K	Predicted transcriptional regulator, HTH
arCOG09713	COG01846		K	Predicted transcriptional regulator, contains ATP-binding and HTH domains
arCOG00680	COG00675	-	L	Transposase
arCOG00683	COG00675	-	L	Transposase
arCOG00684	COG00675	-	L	Transposase
arCOG00889	COG01743	-	L	Adenine-specific DNA methylase containing a Zn-ribbon
arCOG02127	COG03335	-	L	Transposase
arCOG02130	COG03335	-	L	Transposase
arCOG02133	COG01662	InsB	L	Transposase and inactivated derivatives, IS1 family
arCOG02754	COG03039	-	L	Transposase, IS5 family
arCOG02840	COG00415	PhrB	L	Deoxyribodipyrimidine photolyase
arCOG03240	COG01195	RecF	L	Recombinational DNA repair ATPase RecF
arCOG03586	COG03547	-	L	Transposase
arCOG03587	COG03547	-	L	Transposase
arCOG03902	COG03385	-	L	Transposase
arCOG04582	COG00389	DinP	L	Nucleotidyltransferase/DNA polymerase involved in DNA repair
arCOG04918	COG03328	-	L	Transposase
arCOG06508	COG03039	-	L	Transposase and inactivated derivatives, IS5 family
arCOG06915			L	Primase-polymerase (primpol) domain
arCOG07300			L	Uncharacterized conserved protein associated with DNA polymerase II
arCOG07303	COG00675	-	L	Transposase
arCOG07312			L	NurA-like nuclease
arCOG07730			L	DEDD family 3'-5' exonuclease
arCOG07732	COG05558	-	L	Transposase
arCOG07922			L	Transposase ISC1217
arCOG07925			L	Transposase ISC1234
arCOG07929			L	Transposon ISC1160
arCOG08574			L	Viral/Archaeal nuclease
arCOG09995			L	Tranposon-associated protein
arCOG05431	COG00438	RfaG	M	Glycosyltransferase
arCOG05966	COG03511	PlcC	M	Phospholipase C
arCOG00540	COG05131	URM1	O	Ubiquitin-like protein
arCOG02074	COG00425	SirA	O	Predicted redox protein, regulator of disulfide bond formation
arCOG02816	COG00225	MsrA	O	Peptide methionine sulfoxide reductase
arCOG03669	COG04934	-	O	Subtilase family protease
arCOG04530	COG00830	UreF	O	Urease accessory protein UreF
arCOG06188	COG02371	UreE	O	Urease accessory protein UreE
arCOG08336	COG00425	SirA	O	Predicted redox protein, regulator of disulfide bond formation
arCOG00320	COG00704	PhoU	P	Phosphate uptake regulator
arCOG02055	COG00155	CysI	P	Sulfite reductase, beta subunit (hemoprotein)
arCOG02855	COG02146	{NirD}	P	Ferredoxin subunit of nitrite reductase or ring-hydroxylating dioxygenase
arCOG04355	COG01275	TehA	P	Tellurite resistance protein or related permease
arCOG05356	COG02382	Fes	P	Enterochelin esterase or related enzyme
arCOG07228	COG02608		P	Heavy-metal-associated domain
arCOG07339			P	DsrE/DsrF-like family
arCOG07775	COG04638	HcaE	P	Phenylpropionate dioxygenase or related ring-hydroxylating dioxygenase, large terminal subunit
arCOG01659	COG00412	-	Q	Dienelactone hydrolase or related enzyme
arCOG01786	COG00500		Q	SAM-dependent methyltransferase
arCOG02703	COG00500		Q	SAM-dependent methyltransferase
arCOG04786	COG03396	-	Q	Phenylacetic acid catabolic protein
arCOG05303	COG04829		Q	Muconolactone delta-isomerase

arCOG07263	COG01735	Php	Q	Thermostable phosphotriesterase, metal-dependent hydrolase with the TIM-barrel fold
arCOG07267	COG03733	TynA	Q	Cu2+-containing amine oxidase
arCOG08361	COG04228	TmoE	Q	Methane/Phenol/Toluene Hydroxylase component, ferritin superfamily
arCOG08362		TmoD	Q	MmoB/DmpM family, monooxygenase component
arCOG08363			Q	Methane/Phenol/Toluene Hydroxylase component
arCOG08364	COG04228	TmoA	Q	Methane/Phenol/Toluene Hydroxylase component, ferritin superfamily
arCOG08438	COG05517	-	Q	Small subunit of phenylpropionate dioxygenase
arCOG00323	COG00312	TldD	R	Predicted Zn-dependent protease or their inactivated homolog
arCOG00507	COG00491	GloB	R	Zn-dependent hydrolase, glyoxylase family
arCOG00510	COG00655	WrbA	R	Multimeric flavodoxin WrbA
arCOG00511	COG00491	GloB	R	Metal-dependent hydrolase of the beta-lactamase superfamily II
arCOG00517	COG00491	GloB	R	Rhodanese Homology Domain fused to Zn-dependent hydrolase of glyoxylase family
arCOG00887	COG01483	-	R	Predicted ATPase (AAA+ superfamily)
arCOG00931	COG00535	-	R	Radical SAM superfamily enzyme
arCOG01070	COG00446	HcaD	R	NAD(FAD)-dependent dehydrogenase
arCOG01256	COG02872	-	R	Predicted metal-dependent hydrolase related to alanyl-tRNA synthetase HxxxH domain
arCOG01284	COG01545	-	R	Predicted nucleic-acid-binding protein containing a Zn-ribbon
arCOG01289	COG01545	-	R	Predicted nucleic-acid-binding protein containing a Zn-ribbon
arCOG01299	COG00446	HcaD	R	NAD(FAD)-dependent dehydrogenase
arCOG01670	COG02044		R	Predicted peroxiredoxin
arCOG01932	COG02159	-	R	Predicted metal-dependent hydrolase of the TIM-barrel fold
arCOG01933	COG02159	-	R	Predicted metal-dependent hydrolase of the TIM-barrel fold
arCOG01934	COG02159	-	R	Predicted metal-dependent hydrolase of the TIM-barrel fold
arCOG02065	COG02210	-	R	Peroxiredoxin family protein
arCOG02076	COG02044	-	R	Predicted peroxiredoxin
arCOG03031	COG03253	ywfI	R	Chlorite dismutase
arCOG03032	COG00457	NrfG	R	TPR repeats containing protein
arCOG03431	COG04279	-	R	SWIM zinc finger containing protein
arCOG03842	COG00526		R	TlpA/TrxA-like family protein
arCOG03843			R	TlpA/TrxA-like family protein
arCOG05628	COG00730	-	R	Predicted permease
arCOG06005	COG03975	-	R	Predicted protease with the C-terminal PDZ domain
arCOG06009			R	Predicted permease
arCOG06014	COG04194	-	R	Predicted membrane protein
arCOG07181	COG03889		R	Predicted solute binding protein
arCOG07202	COG01988		R	Predicted membrane-bound metal-dependent hydrolase, DUF457
arCOG07281	COG00457	NrfG	R	TPR repeats containing protein
arCOG07313			R	ATPase, uncharacterized
arCOG07719	COG00535	-	R	Radical SAM superfamily enzyme
arCOG00023	COG02522		S	Uncharacterized conserved protein
arCOG02108	COG02445	-	S	HEPN domain containing protein
arCOG02560	COG03391	-	S	Beta-propeller repeat containing protein
arCOG02617			S	Uncharacterized conserved protein
arCOG02719	COG02034	-	S	Predicted membrane protein
arCOG02761	COG03439	-	S	Uncharacterized conserved protein
arCOG02863	COG02259	-	S	Predicted membrane protein, DoxD family
arCOG03002	COG01917	-	S	Cupin domain fused to uncharacterized domain of COG4309
arCOG03560			S	Uncharacterized conserved protein
arCOG03693			S	Uncharacterized conserved protein
arCOG03701	COG05650	-	S	Predicted integral membrane protein
arCOG03703	COG05650	-	S	Predicted integral membrane protein
arCOG03840	COG03535	-	S	Uncharacterized conserved protein

arCOG03856			S	Uncharacterized conserved protein
arCOG03857			S	Uncharacterized conserved protein
arCOG03862			S	Uncharacterized conserved protein
arCOG03868			S	Uncharacterized conserved protein
arCOG04030			S	Uncharacterized conserved protein
arCOG04031			S	Uncharacterized conserved protein
arCOG04697			S	Selenium binding protein SBP56 homolog
arCOG05211	COG04831	-	S	Roadblock/LC7 domain
arCOG05278	COG05466	-	S	Predicted small metal-binding protein
arCOG05328			S	Uncharacterized conserved protein
arCOG05339			S	Uncharacterized conserved protein
arCOG05360			S	Uncharacterized conserved protein
arCOG05362			S	Uncharacterized conserved protein
arCOG05367	COG05592	-	S	Hemerythrin HHE cation binding domain containing protein
arCOG05416			S	Uncharacterized conserved protein
arCOG05633			S	Uncharacterized conserved protein
arCOG05707			S	Uncharacterized conserved protein
arCOG05881			S	Uncharacterized conserved protein, DUF1177
arCOG05887			S	Uncharacterized conserved protein
arCOG05889			S	Uncharacterized conserved protein
arCOG05893			S	Uncharacterized conserved protein
arCOG05901			S	Uncharacterized conserved protein
arCOG05910			S	Uncharacterized conserved protein
arCOG05914			S	Uncharacterized conserved protein
arCOG05915			S	Uncharacterized conserved protein
arCOG05944			S	Uncharacterized conserved protein
arCOG05945			S	Uncharacterized conserved protein
arCOG05962			S	Uncharacterized conserved protein
arCOG05963			S	Uncharacterized conserved protein
arCOG05968			S	Uncharacterized conserved protein
arCOG05970			S	Uncharacterized conserved protein
arCOG05982			S	Uncharacterized conserved protein
arCOG06006			S	Uncharacterized conserved membrane protein
arCOG06010	COG04280	-	S	Predicted membrane protein
arCOG06012			S	Uncharacterized conserved protein
arCOG06013			S	Uncharacterized conserved protein
arCOG06018			S	Uncharacterized conserved protein
arCOG06021			S	Uncharacterized conserved protein
arCOG06030			S	Uncharacterized conserved protein
arCOG06036	COG00208		S	Uncharacterized conserved protein
arCOG06045	COG03589	-	S	Uncharacterized conserved protein
arCOG06096			S	Uncharacterized conserved protein
arCOG06946			S	Uncharacterized conserved membrane protein
arCOG06947			S	Zn finger protein
arCOG06952			S	Uncharacterized conserved protein
arCOG07178			S	Uncharacterized conserved protein
arCOG07179			S	Uncharacterized conserved protein
arCOG07185			S	Uncharacterized conserved protein
arCOG07187			S	Uncharacterized conserved protein
arCOG07189			S	Uncharacterized conserved protein
arCOG07190			S	Uncharacterized conserved protein
arCOG07191			S	Uncharacterized conserved protein
arCOG07196			S	Uncharacterized conserved protein
arCOG07200			S	Uncharacterized conserved protein
arCOG07205			S	Uncharacterized conserved protein
arCOG07206			S	Uncharacterized conserved protein
arCOG07209			S	Uncharacterized conserved protein
arCOG07211			S	Uncharacterized conserved protein
arCOG07218			S	Uncharacterized conserved protein
arCOG07222			S	Uncharacterized conserved protein
arCOG07224			S	Uncharacterized conserved protein
arCOG07227			S	Uncharacterized conserved protein
arCOG07229			S	Uncharacterized conserved protein
arCOG07230			S	Uncharacterized conserved protein
arCOG07231			S	Uncharacterized conserved protein
arCOG07232			S	Uncharacterized conserved protein

arCOG07234			S	Uncharacterized conserved protein
arCOG07238			S	Uncharacterized conserved protein
arCOG07239			S	Uncharacterized conserved protein
arCOG07243			S	Uncharacterized conserved protein
arCOG07244			S	Uncharacterized conserved protein
arCOG07246			S	Uncharacterized conserved protein
arCOG07249			S	Uncharacterized conserved protein
arCOG07250			S	Uncharacterized conserved protein
arCOG07252			S	Uncharacterized conserved protein
arCOG07253			S	Uncharacterized conserved protein
arCOG07255			S	Uncharacterized conserved protein
arCOG07257			S	Uncharacterized conserved protein
arCOG07258			S	Uncharacterized conserved protein
arCOG07259			S	Uncharacterized conserved protein
arCOG07264			S	Predicted membrane protein with C-terminal Zn ribbon domain
arCOG07265			S	Uncharacterized conserved protein
arCOG07268			S	Uncharacterized conserved protein
arCOG07275			S	Uncharacterized conserved protein
arCOG07277			S	SSV1_ORF_D-335, predicted GIY-YIG nuclease superfamily protein
arCOG07279			S	Uncharacterized conserved protein
arCOG07280			S	Uncharacterized conserved protein
arCOG07282			S	Uncharacterized conserved protein
arCOG07289			S	Uncharacterized conserved protein
arCOG07290			S	Uncharacterized conserved protein
arCOG07291			S	Uncharacterized conserved protein
arCOG07294	COG02308	-	S	Uncharacterized conserved protein
arCOG07295	COG02307		S	Uncharacterized conserved protein
arCOG07298			S	Uncharacterized conserved protein
arCOG07299			S	CRISPR-associated protein CsaX
arCOG07301			S	Uncharacterized conserved protein
arCOG07307			S	Uncharacterized conserved protein
arCOG07308			S	Uncharacterized conserved protein
arCOG07310			S	Uncharacterized conserved protein
arCOG07316			S	Uncharacterized conserved protein
arCOG07317			S	Uncharacterized conserved protein
arCOG07318			S	Uncharacterized conserved protein
arCOG07319			S	Uncharacterized conserved protein
arCOG07324			S	Uncharacterized conserved protein
arCOG07325			S	Uncharacterized conserved protein
arCOG07326			S	Uncharacterized conserved protein
arCOG07327			S	Uncharacterized conserved protein
arCOG07330			S	Uncharacterized conserved protein
arCOG07333			S	Uncharacterized conserved protein
arCOG07340			S	Uncharacterized conserved protein
arCOG07341			S	Uncharacterized conserved protein
arCOG07342			S	Uncharacterized conserved protein
arCOG07343			S	Uncharacterized conserved protein
arCOG07351			S	Uncharacterized conserved membrane protein
arCOG07523			S	Uncharacterized conserved protein
arCOG07714			S	Uncharacterized conserved protein
arCOG07715			S	Uncharacterized conserved protein
arCOG07717			S	Uncharacterized conserved protein
arCOG07720			S	Uncharacterized conserved protein
arCOG07721			S	Uncharacterized conserved protein
arCOG07722			S	Uncharacterized conserved protein
arCOG07724			S	Uncharacterized conserved protein
arCOG07726			S	Uncharacterized conserved protein
arCOG07727			S	Uncharacterized conserved protein
arCOG07733			S	Uncharacterized conserved protein
arCOG07734			S	Uncharacterized conserved protein
arCOG07844			S	PKD domain containing protein
arCOG07848			S	Uncharacterized conserved protein
arCOG07850			S	Uncharacterized conserved protein
arCOG07851			S	Uncharacterized conserved protein
arCOG07853			S	Uncharacterized conserved protein
arCOG07940			S	Uncharacterized protein
arCOG08073			S	Uncharacterized protein

arCOG08092	COG02259	-	S	Predicted membrane protein, DoxX family
arCOG08304			S	Uncharacterized conserved protein
arCOG08307			S	Uncharacterized conserved protein
arCOG08310			S	Uncharacterized conserved protein
arCOG08311			S	Uncharacterized conserved protein
arCOG08312			S	Uncharacterized conserved protein
arCOG08313			S	Uncharacterized conserved protein
arCOG08314			S	Uncharacterized conserved protein
arCOG08316			S	Uncharacterized conserved protein
arCOG08317			S	Uncharacterized conserved protein
arCOG08318			S	Uncharacterized conserved protein
arCOG08319			S	Uncharacterized conserved protein
arCOG08321			S	Uncharacterized conserved protein
arCOG08323			S	Uncharacterized conserved protein
arCOG08326			S	Uncharacterized conserved protein
arCOG08328			S	Uncharacterized conserved protein
arCOG08329			S	Uncharacterized conserved protein
arCOG08330			S	Uncharacterized conserved protein
arCOG08331			S	Uncharacterized conserved protein
arCOG08338			S	Uncharacterized conserved protein
arCOG08340			S	Uncharacterized conserved protein
arCOG08344			S	Uncharacterized conserved protein
arCOG08345			S	Uncharacterized conserved protein
arCOG08346			S	Uncharacterized conserved protein
arCOG08347			S	Uncharacterized conserved protein
arCOG08348			S	Uncharacterized conserved protein
arCOG08349			S	Uncharacterized conserved protein
arCOG08375			S	Uncharacterized conserved protein
arCOG08377			S	Uncharacterized conserved protein
arCOG08395			S	Uncharacterized conserved protein
arCOG08400			S	Uncharacterized conserved protein
arCOG08401			S	Uncharacterized conserved protein
arCOG08405			S	Uncharacterized conserved protein
arCOG08406			S	Uncharacterized conserved protein
arCOG08408			S	Uncharacterized conserved protein
arCOG08412			S	Uncharacterized conserved protein
arCOG08424			S	Uncharacterized conserved protein
arCOG08426			S	Uncharacterized conserved protein
arCOG08436			S	Uncharacterized conserved protein
arCOG08437			S	Uncharacterized conserved protein
arCOG08442			S	Uncharacterized conserved protein
arCOG08446			S	Uncharacterized conserved protein
arCOG08449			S	Uncharacterized conserved protein
arCOG08451			S	Uncharacterized conserved protein
arCOG08463	COG02433		S	Uncharacterized conserved protein, DUF904
arCOG08464			S	Uncharacterized conserved protein
arCOG08465			S	Uncharacterized conserved protein
arCOG08466			S	Uncharacterized conserved protein
arCOG08472			S	Uncharacterized conserved protein
arCOG08473			S	Uncharacterized conserved protein
arCOG08477			S	Uncharacterized conserved protein
arCOG08481			S	Uncharacterized conserved protein
arCOG08485			S	Uncharacterized conserved protein
arCOG08486			S	Uncharacterized conserved protein
arCOG08497			S	Uncharacterized conserved protein
arCOG08498			S	Uncharacterized conserved protein
arCOG08510			S	Uncharacterized conserved protein
arCOG08511			S	Uncharacterized conserved protein
arCOG08520			S	Uncharacterized conserved protein
arCOG08525			S	Uncharacterized conserved protein
arCOG08526			S	Uncharacterized conserved protein
arCOG08530			S	Uncharacterized conserved protein
arCOG08539			S	Uncharacterized conserved protein
arCOG08540			S	Uncharacterized conserved protein
arCOG08543			S	Uncharacterized conserved protein
arCOG08552			S	Uncharacterized conserved protein
arCOG08555			S	Uncharacterized conserved protein
arCOG08557			S	Uncharacterized conserved protein
arCOG08558			S	Uncharacterized conserved protein

arCOG08562			S	Uncharacterized conserved protein
arCOG08563			S	Uncharacterized conserved protein
arCOG08569			S	Virus associated protein, V1 family
arCOG08572			S	Sulfolobus virus associated uncharacterized membrane protein
arCOG08576			S	Uncharacterized conserved protein
arCOG08583			S	Uncharacterized conserved protein
arCOG08584			S	Uncharacterized conserved protein
arCOG08587			S	Uncharacterized conserved protein
arCOG08588			S	Uncharacterized conserved protein associated with transposon
arCOG09692			S	Uncharacterized conserved protein
arCOG09693			S	Uncharacterized conserved membrane protein
arCOG09694			S	Uncharacterized conserved protein
arCOG09695			S	Uncharacterized conserved protein
arCOG09696			S	Uncharacterized conserved protein
arCOG09697			S	Uncharacterized conserved protein
arCOG09698			S	Uncharacterized conserved protein
arCOG09699			S	Uncharacterized conserved protein
arCOG09703			S	Uncharacterized conserved protein
arCOG09704			S	Uncharacterized conserved protein
arCOG09705			S	Uncharacterized conserved protein
arCOG09706			S	Uncharacterized conserved protein
arCOG09707			S	Uncharacterized conserved protein
arCOG09709			S	Uncharacterized conserved protein
arCOG09710			S	Uncharacterized conserved protein
arCOG09711			S	Uncharacterized conserved protein
arCOG09712			S	Uncharacterized conserved protein
arCOG09855			S	Uncharacterized conserved protein
arCOG09856			S	Uncharacterized conserved protein
arCOG09862			S	Uncharacterized conserved protein
arCOG09866			S	Uncharacterized conserved protein
arCOG09879			S	Uncharacterized conserved protein
arCOG09882	COG02192		S	Uncharacterized conserved protein
arCOG09883			S	Uncharacterized conserved protein
arCOG09884			S	Uncharacterized conserved protein
arCOG09923			S	Uncharacterized conserved protein
arCOG09924			S	Uncharacterized conserved protein
arCOG09928			S	Uncharacterized conserved protein
arCOG09933			S	Uncharacterized conserved protein
arCOG09934			S	Uncharacterized conserved protein
arCOG09948			S	Uncharacterized conserved protein
arCOG09962			S	Uncharacterized conserved protein
arCOG09968			S	Uncharacterized conserved protein
arCOG09969			S	Uncharacterized conserved protein
arCOG09989			S	Uncharacterized conserved protein
arCOG09991			S	Uncharacterized conserved protein
arCOG10005			S	Uncharacterized conserved protein
arCOG10007	COG00829		S	Uncharacterized conserved protein
arCOG10013			S	Uncharacterized conserved protein
arCOG10014			S	Uncharacterized conserved protein
arCOG10015	COG04278	-	S	Uncharacterized conserved protein
arCOG10016			S	Uncharacterized conserved protein
arCOG10022			S	Uncharacterized conserved protein
arCOG10266			S	Uncharacterized conserved protein
arCOG10299			S	Uncharacterized conserved protein
arCOG10433			S	Uncharacterized conserved protein
arCOG10507			S	Uncharacterized conserved protein
arCOG10885			S	Uncharacterized conserved protein
arCOG11006			S	Uncharacterized conserved protein
arCOG11043			S	Uncharacterized conserved protein
arCOG11294			S	Uncharacterized conserved protein
arCOG11295			S	Uncharacterized conserved protein
arCOG11298			S	Uncharacterized conserved protein
arCOG11361			S	Uncharacterized conserved protein
arCOG01740	COG00681	LepB	U	Signal peptidase I
arCOG00208	COG01131	CcmA	V	ABC-type multidrug transport system, ATPase component
arCOG00716	COG01848	-	V	PIN domain containing protein

	arCOG02227	COG01487	VapC	V	PIN domain containing protein
	arCOG02658	COG01337	Cas7/Csm3	V	CRISPR system related protein, RAMP superfamily Cas7 group
	arCOG02661	COG01604	Cas7/Cmr6	V	CRISPR system related protein, RAMP superfamily Cas7 group
	arCOG03437	COG01517		V	CRISPR system related protein, COG1517 family
	arCOG03891	COG01367	Cas7/Cmr1	V	CRISPR system related protein, RAMP superfamily Cas7 group
	arCOG03893	COG01367	Cas7/Cmr1	V	CRISPR system related protein, RAMP superfamily Cas7 group
	arCOG06024		Cas5/Cmr3	V	CRISPR-associated protein, RAMP family Cas5 group
	arCOG07186			V	PIN domain
	arCOG07248			V	PIN domain
	arCOG07297			V	CRISPR associated protein
	arCOG07736			V	TOPRIM domain containing protein, predicted phage defense system
	arCOG07846	COG01604	Cas7/Cmr6	V	CRISPR system related protein, RAMP superfamily Cas7 group
	arCOG08378			V	CopG/RHH family DNA binding protein
	arCOG08805	COG05304		V	CopG/RHH family DNA binding protein
**Loss in other Sulfolobales**					
	arCOG00934	COG01313	PflX	C	Radical SAM superfamily enzyme
	arCOG01550	COG00374	HyaB	C	Ni,Fe-hydrogenase I large subunit
	arCOG02474	COG01740	HyaA	C	Ni,Fe-hydrogenase I small subunit
	arCOG00143	COG00477	ProP	G	Permease of the major facilitator superfamily
	arCOG03664	COG01215		G	Glycosyltransferase
	arCOG00462	COG01194	MutY	L	A/G-specific DNA glycosylase
	arCOG02079	COG01361	-	M	S-layer domain
	arCOG04400	COG01361	-	M	S-layer domain
	arCOG01187	COG00068	HypF	O	Hydrogenase maturation factor
	arCOG04427	COG00298	HypC	O	Hydrogenase maturation factor
	arCOG04428	COG00409	HypD	O	Hydrogenase maturation factor
	arCOG00940	COG00535	-	R	Radical SAM superfamily enzyme
	arCOG00968	COG02428	-	R	Alpha/beta-knot SAM-dependent RNA methyltransferase
	arCOG01423	COG05493	-	R	RecB-like nuclease fused to coiled-coil domain
	arCOG06089			R	SAM-dependent methyltransferase
	arCOG03812	COG03463	-	S	Predicted membrane protein
	arCOG06072			S	Uncharacterized conserved protein
	arCOG05482	COG00467	RAD55	T	RecA-superfamily ATPase implicated in signal transduction
	arCOG02632	COG00286	HsdM	V	Type I restriction-modification system methyltransferase subunit

Ancestral Genes in Sulfolobales

arCOG Family	present in Acd1	COG correspondence	Gene Name
arCOG00001	no	COG01695	
arCOG00002	Yes	COG01695	
arCOG00009	Yes	COG00531	PotE
arCOG00014	Yes	COG00524	RbsK
arCOG00016	Yes	COG00524	RbsK
arCOG00017	Yes	COG02522	
arCOG00018	Yes	COG00063	
arCOG00020	Yes	COG00351	ThiD
arCOG00021	Yes	COG01992	
arCOG00023	no	COG02522	
arCOG00024	no	COG00554	GlpK
arCOG00025	no	COG01070	XylB
arCOG00029	Yes	COG00461	PyrE
arCOG00031	Yes	COG00503	Apt
arCOG00033	Yes	COG02520	Trm5
arCOG00034	Yes	COG00311	PDX2
arCOG00035	Yes	COG02102	
arCOG00038	Yes	COG00301	ThiI
arCOG00039	Yes	COG00603	
arCOG00040	Yes	COG02236	
arCOG00042	Yes	COG00037	MesJ
arCOG00047	Yes	COG01041	
arCOG00050	Yes	COG00421	SpeE
arCOG00052	Yes	COG00166	Pgi
arCOG00053	Yes	COG00269	SgbH
arCOG00057	Yes	COG00449	GlmS
arCOG00059	no	COG02222	AgaS
arCOG00060	Yes	COG00626	MetC
arCOG00062	Yes	COG00388	
arCOG00063	Yes	COG00504	PyrG
arCOG00064	Yes	COG00505	CarA
arCOG00065	Yes	COG00520	csdA
arCOG00067	Yes	COG00462	PrsA
arCOG00068	Yes	COG00794	GutQ
arCOG00069	Yes	COG00171	NadE
arCOG00070	Yes	COG00112	GlyA
arCOG00076	Yes	COG01003	GcvP
arCOG00077	Yes	COG00403	GcvP
arCOG00078	Yes	COG01889	NOP1
arCOG00081	Yes	COG00284	PyrF
arCOG00082	Yes	COG00075	
arCOG00084	Yes	COG01818	
arCOG00085	Yes	COG00519	GuaA
arCOG00086	Yes	COG00512	PabA
arCOG00087	Yes	COG00518	GuaA
arCOG00089	Yes	COG00118	HisH

arCOG00090	no	COG00518	GuaA
arCOG00093	Yes	COG00034	PurF
arCOG00094	Yes	COG00034	PurF
arCOG00095	Yes	COG00067	GltB
arCOG00096	Yes	COG00070	GltB
arCOG00102	Yes	COG00047	PurL
arCOG00105	Yes	COG01492	CobQ
arCOG00106	Yes	COG01797	CobB
arCOG00109	Yes	COG02890	HemK
arCOG00110	Yes	COG02813	RsmC
arCOG00112	Yes	COG00137	ArgG
arCOG00115	no	COG00863	
arCOG00124	no	COG00175	CysH
arCOG00130	Yes	COG00477	ProP
arCOG00131	Yes	COG00477	ProP
arCOG00132	Yes	COG00477	ProP
arCOG00135	no	COG00477	ProP
arCOG00138	Yes	COG00477	ProP
arCOG00140	no	COG00477	ProP
arCOG00144	Yes	COG00477	ProP
arCOG00147	Yes	COG00477	ProP
arCOG00150	no	COG01653	UgpB
arCOG00153	no	COG01653	UgpB
arCOG00154	no	COG02182	MalE
arCOG00156	no	COG01653	UgpB
arCOG00157	no	COG01175	UgpA
arCOG00158	no	COG00395	UgpE
arCOG00159	no	COG00395	UgpE
arCOG00160	no	COG03833	MalG
arCOG00164	Yes	COG00555	CysU
arCOG00167	Yes	COG00573	PstC
arCOG00168	Yes	COG00581	PstA
arCOG00174	Yes	COG04986	
arCOG00175	no	COG03839	MalK
arCOG00177	no	COG03842	PotA
arCOG00181	Yes	COG00444	DppD
arCOG00182	no	COG00444	DppD
arCOG00184	Yes	COG04608	AppF
arCOG00185	no	COG01123	
arCOG00187	Yes	COG01245	
arCOG00188	no	COG01122	CbiO
arCOG00193	Yes	COG01116	TauB
arCOG00194	Yes	COG01131	CcmA
arCOG00196	Yes	COG01131	CcmA
arCOG00198	Yes	COG01120	FepC
arCOG00204	no	COG04608	AppF
arCOG00208	no	COG01131	CcmA
arCOG00213	Yes	COG00226	PstS

arCOG00214	Yes	COG00521	MoaB
arCOG00215	Yes	COG01058	CinA
arCOG00216	Yes	COG00303	MoeA
arCOG00217	no	COG00303	MoeA
arCOG00219	Yes	COG00725	ModA
arCOG00223	Yes	COG02005	ModE
arCOG00231	Yes	COG01117	PstB
arCOG00235	Yes	COG00179	MhpD
arCOG00236	no	COG03970	
arCOG00238	Yes	COG01055	ArsB
arCOG00239	no	COG01024	CaiD
arCOG00242	Yes	COG01024	CaiD
arCOG00245	Yes	COG00287	TyrA
arCOG00246	no	COG00039	Mdh
arCOG00247	Yes	COG02084	MmsB
arCOG00249	Yes	COG01250	FadB
arCOG00251	Yes	COG01250	FadB
arCOG00253	no	COG01004	Ugd
arCOG00255	Yes	COG00077	PheA
arCOG00263	no	COG01172	AraH
arCOG00264	Yes	COG02041	
arCOG00266	no	COG02041	
arCOG00271	Yes	COG00697	RhaT
arCOG00279	Yes	COG01586	SpeD
arCOG00280	Yes	COG00433	
arCOG00283	Yes	COG00433	
arCOG00284	Yes	COG00433	
arCOG00285	Yes	COG00433	
arCOG00286	Yes	COG00433	
arCOG00287	Yes	SC.00240	
arCOG00288	Yes	COG00778	NfnB
arCOG00302	Yes	COG00613	
arCOG00307	Yes	COG01603	RPP1
arCOG00310	Yes	COG01225	Bcp
arCOG00312	Yes	COG00450	AhpC
arCOG00317	Yes	COG04847	
arCOG00318	Yes	COG00704	PhoU
arCOG00320	no	COG00704	PhoU
arCOG00321	Yes	COG00312	TldD
arCOG00322	Yes	COG00312	TldD
arCOG00323	no	COG00312	TldD
arCOG00324	Yes	COG00123	AcuC
arCOG00326	Yes	COG00123	AcuC
arCOG00328	Yes	COG00417	PolB
arCOG00329	Yes	COG00417	PolB
arCOG00332	Yes	COG00247	GlpC
arCOG00333	Yes	COG00247	GlpC
arCOG00335	Yes	COG01139	

arCOG00337	Yes	COG00277	GlcD
arCOG00338	Yes	COG02048	SdhC
arCOG00340	Yes	COG00277	GlcD
arCOG00341	no	COG00277	GlcD
arCOG00342	Yes	COG02048	HdrB
arCOG00343	Yes	COG00247	GlpC
arCOG00344	Yes	COG00277	GlcD
arCOG00345	Yes	COG00277	GlcD
arCOG00347	Yes	COG01938	
arCOG00350	Yes	COG01161	
arCOG00352	Yes	COG01084	
arCOG00353	Yes	COG02262	HflX
arCOG00357	Yes	COG00012	
arCOG00358	Yes	COG01163	DRG
arCOG00367	Yes	COG01630	
arCOG00368	Yes	COG00419	SbcC
arCOG00373	Yes	COG01196	
arCOG00374	no	COG02512	
arCOG00385	no		
arCOG00393	Yes	COG02512	
arCOG00394	no	COG00640	ArsR
arCOG00397	Yes	COG00420	SbcD
arCOG00401	Yes	COG00441	ThrS
arCOG00402	Yes	COG00442	ProS
arCOG00403	Yes	COG00172	SerS
arCOG00404	Yes	COG00124	HisS
arCOG00405	Yes	COG00423	GRS1
arCOG00406	Yes	COG00017	AsnS
arCOG00408	Yes	COG01190	LysU
arCOG00410	Yes	COG00016	PheS
arCOG00412	Yes	COG00072	PheT
arCOG00415	Yes	COG00468	RecA
arCOG00417	Yes	COG00468	RecA
arCOG00419	Yes	COG00537	Hit
arCOG00423	Yes	COG02404	
arCOG00427	Yes	COG00608	RecJ/Cdc45
arCOG00434	no	COG00714	
arCOG00436	Yes	COG00714	
arCOG00439	Yes	COG01241	MCM2
arCOG00442	Yes	COG02425	
arCOG00446	no	COG02086	FixA
arCOG00447	no	COG02025	FixB
arCOG00448	no	COG02086	FixA
arCOG00452	Yes	COG05491	ESCRTIII
arCOG00453	Yes	COG05491	ESCRTIII
arCOG00454	Yes	COG05491	ESCRTIII
arCOG00455	no	COG00345	ProC
arCOG00458	Yes	COG01913	

arCOG00459	Yes	COG00177	Nth
arCOG00461	Yes	COG02231	
arCOG00463	Yes	COG01833	
arCOG00467	Yes	COG01474	CDC6
arCOG00469	Yes	COG00470	HolB
arCOG00470	Yes	COG00470	HolB
arCOG00474	Yes	COG00212	
arCOG00475	Yes	COG00685	MetF
arCOG00476	Yes	COG00382	UbiA
arCOG00477	Yes	COG00382	UbiA
arCOG00479	Yes	COG00109	CyoE
arCOG00486	Yes	COG00215	CysS
arCOG00487	Yes	COG00018	ArgS
arCOG00488	Yes	COG00592	DnaN
arCOG00489	Yes	COG02096	
arCOG00492	Yes	COG01167	ARO8
arCOG00493	Yes	COG00057	GapA
arCOG00494	Yes	COG00136	Asd
arCOG00495	Yes	COG00002	ArgC
arCOG00496	Yes	COG00126	Pgk
arCOG00497	Yes	COG02220	
arCOG00498	no	COG00491	GloB
arCOG00500	Yes	COG01234	ElaC
arCOG00501	Yes	COG01234	ElaC
arCOG00503	Yes	COG01237	
arCOG00504	Yes	COG00491	GloB
arCOG00505	no	COG00491	GloB
arCOG00506	Yes	COG00426	FpaA
arCOG00507	no	COG00491	GloB
arCOG00510	no	COG00655	WrbA
arCOG00511	no	COG00491	GloB
arCOG00517	no	COG00491	GloB
arCOG00532	Yes	COG01763	MobB
arCOG00534	Yes	COG00314	MoaE
arCOG00535	Yes	COG02104	ThiS
arCOG00536	Yes	COG01977	MoaD
arCOG00540	no	COG05131	URM1
arCOG00541	Yes	COG01236	YSH1
arCOG00543	Yes	COG01782	
arCOG00545	Yes	COG01236	YSH1
arCOG00551	Yes	COG01711	
arCOG00553	Yes	COG01204	
arCOG00555	Yes	COG01205	
arCOG00557	Yes	COG01201	Lhr
arCOG00558	no	COG00513	SrmB
arCOG00561	Yes	COG04346	
arCOG00569	Yes		
arCOG00570	Yes	COG00644	FixC

arCOG00571	Yes	COG01053	SdhA
arCOG00572	Yes	COG00029	NadB
arCOG00574	Yes	COG01635	
arCOG00576	no	COG00428	
arCOG00578	Yes	COG01645	
arCOG00579	Yes	COG01594	RPB9
arCOG00580	Yes	COG01594	RPB9
arCOG00581	no	COG02513	PrpB
arCOG00583	no	COG02224	AceA
arCOG00584	Yes	COG00413	PanB
arCOG00585	Yes	COG00489	Mrp
arCOG00586	Yes	COG01192	Soj
arCOG00589	no	COG00455	
arCOG00600	Yes	COG00517	
arCOG00601	Yes	COG00517	
arCOG00603	Yes	COG00167	PyrD
arCOG00606	no	COG00517	
arCOG00608	no	COG03620	
arCOG00610	Yes	COG02524	
arCOG00613	Yes	COG01304	idi
arCOG00614	Yes	COG01994	SpoIVFB
arCOG00615	no	COG01902	NemA
arCOG00617	Yes	COG00107	HisF
arCOG00618	Yes	COG00106	HisA
arCOG00619	Yes	COG00069	GltB
arCOG00625	no	COG02239	MgtE
arCOG00631	Yes	COG00517	
arCOG00636	Yes	COG00309	HypE
arCOG00637	Yes	COG01973	HypE
arCOG00638	Yes	COG00611	ThiL
arCOG00639	Yes	COG00150	PurM
arCOG00641	Yes	COG00046	PurL
arCOG00643	Yes	COG00709	SelD
arCOG00644	Yes	COG00007	CysG
arCOG00645	Yes	COG02875	CobM
arCOG00646	no	COG00007	CysG
arCOG00647	Yes	COG01010	CobJ
arCOG00648	Yes	COG02243	CobF
arCOG00650	Yes	COG02241	CobL
arCOG00651	Yes	COG02073	CbiG
arCOG00652	Yes	COG02073	CbiG
arCOG00654	no	COG02107	
arCOG00660	no	COG00320	LipA
arCOG00662	Yes	COG02516	
arCOG00663	Yes	COG01208	GCD1
arCOG00664	Yes	COG01209	RfbA
arCOG00665	no	COG01210	GalU
arCOG00666	Yes	COG01208	GCD1

arCOG00667	Yes	COG01209	RfbA
arCOG00670	Yes	COG00558	PgsA
arCOG00675	Yes	COG01095	RPB7
arCOG00676	Yes	COG01096	
arCOG00678	Yes	COG01097	RRP4
arCOG00679	Yes	COG00675	
arCOG00680	no	COG00675	
arCOG00683	no	COG00675	
arCOG00684	no	COG00675	
arCOG00689	Yes	COG00044	PyrC
arCOG00692	Yes	COG00402	SsnA
arCOG00695	Yes	COG00402	SsnA
arCOG00696	no	COG01228	HutI
arCOG00697	no	COG00402	SsnA
arCOG00698	no	COG00804	UreC
arCOG00699	no	COG01820	NagA
arCOG00701	no	COG00584	UgpQ
arCOG00713	Yes	COG01848	
arCOG00715	Yes	COG01848	
arCOG00716	no	COG01848	
arCOG00721	Yes	COG01439	Nob1
arCOG00723	no	COG05483	
arCOG00724	Yes	COG01695	
arCOG00726	no	COG04113	
arCOG00727	no	COG04113	
arCOG00729	Yes	COG04113	
arCOG00731	Yes	COG00640	ArsR
arCOG00732	no	COG01846	MarR
arCOG00744	Yes		
arCOG00748	no	COG01173	DppC
arCOG00749	Yes	COG01173	DppC
arCOG00751	Yes	COG00601	DppB
arCOG00753	no	COG00578	GlpA
arCOG00755	Yes	COG00665	DadA
arCOG00756	Yes	COG00404	GcvT
arCOG00758	Yes	COG00665	DadA
arCOG00759	no	COG00665	DadA
arCOG00760	no	COG02301	Mcl/CitE
arCOG00761	no	COG01720	
arCOG00767	Yes	COG01109	{ManB}
arCOG00769	Yes	COG00693	ThiJ
arCOG00770	Yes	COG01199	DinG
arCOG00773	Yes	COG01607	
arCOG00774	no	COG02030	Mch
arCOG00777	Yes	COG02050	PaaI
arCOG00779	Yes	COG00200	RplO
arCOG00780	Yes	COG01727	RPL18A
arCOG00781	Yes	COG01717	RPL32

arCOG00782	Yes	COG00199	RpsN
arCOG00784	Yes	COG01588	POP4
arCOG00785	Yes	COG00255	RpmC
arCOG00786	no	COG01468	Cas4
arCOG00790	Yes	COG01468	Cas4
arCOG00792	Yes	COG01112	
arCOG00807	Yes	COG00060	IleS
arCOG00808	Yes	COG00525	ValS
arCOG00809	Yes	COG00495	LeuS
arCOG00810	Yes	COG00143	MetG
arCOG00812	Yes	COG02002	AbrB
arCOG00815	Yes	COG02002	AbrB
arCOG00816	no	COG02002	AbrB
arCOG00817	Yes	COG02002	AbrB
arCOG00818	no	COG02002	AbrB
arCOG00820	no	COG02002	AbrB
arCOG00823	Yes	COG02002	AbrB
arCOG00824	Yes	COG02002	AbrB
arCOG00825	no	COG02002	AbrB
arCOG00826	no	COG00454	WecD
arCOG00833	Yes	COG00456	RimI
arCOG00837	no	COG00456	RimI
arCOG00853	Yes	COG00281	SfcA
arCOG00856	no	COG00318	CaiC
arCOG00857	Yes	COG00318	CaiC
arCOG00858	Yes	COG00528	PyrH
arCOG00860	no	COG01608	
arCOG00861	Yes	COG00527	LysC
arCOG00862	Yes	COG00548	ArgB
arCOG00865	Yes	COG01156	NtpB
arCOG00868	Yes	COG01155	NtpA
arCOG00869	Yes	COG01390	NtpE
arCOG00871	no	COG00553	HepA
arCOG00874	Yes	COG01061	xpb
arCOG00877	no	COG01061	SSL2
arCOG00883	no	COG00553	HepA
arCOG00887	no	COG01483	
arCOG00889	no	COG01743	
arCOG00890	no	COG00863	
arCOG00891	Yes	COG00084	TatD
arCOG00894	no	COG00463	WcaA
arCOG00895	Yes	COG00463	WcaA
arCOG00905	Yes	COG01573	
arCOG00906	Yes	COG02260	
arCOG00910	Yes	COG02263	
arCOG00911	Yes	COG00540	PyrB
arCOG00912	Yes	COG00078	ArgF
arCOG00914	Yes	COG04992	ArgD

arCOG00915	no	COG00160	GabT
arCOG00916	Yes	COG00160	GabT
arCOG00918	Yes	COG00001	HemL
arCOG00919	Yes	COG01591	
arCOG00922	Yes	COG01136	SalX
arCOG00924	no	COG00410	LivF
arCOG00925	no	COG00411	LivG
arCOG00928	Yes	COG01628	
arCOG00929	Yes	COG01515	Nfi
arCOG00930	Yes	COG02896	MoaA
arCOG00931	no	COG00535	
arCOG00932	Yes	COG02108	
arCOG00933	Yes	COG01964	
arCOG00938	no	COG00535	
arCOG00945	Yes	COG00641	AslB
arCOG00946	Yes	COG01180	PflA
arCOG00959	no	COG01146	
arCOG00962	Yes	COG00479	FrdB
arCOG00965	Yes	COG01150	HdrC
arCOG00966	Yes	COG01150	HdrC
arCOG00967	Yes	COG02419	
arCOG00969	Yes	COG02248	
arCOG00970	Yes	COG00638	PRE1
arCOG00971	Yes	COG00638	PRE1
arCOG00972	Yes	COG01056	NadR
arCOG00973	Yes	COG00144	Sun
arCOG00975	Yes	COG00144	Sun
arCOG00976	Yes	COG02518	Pcm
arCOG00977	Yes	COG02242	CobL
arCOG00978	Yes	COG02519	GCD14
arCOG00980	Yes	COG01047	SlpA
arCOG00982	Yes	COG00371	GldA
arCOG00983	Yes	COG00337	AroB
arCOG00984	no	COG01454	EutG
arCOG00985	Yes	COG02016	
arCOG00986	Yes	COG00144	Sun
arCOG00987	Yes	COG00130	TruB
arCOG00989	Yes	COG00343	Tgt
arCOG00991	Yes	COG01370	
arCOG00993	Yes	COG01374	NIP7
arCOG00995	no	COG01549	
arCOG00996	no	COG00343	Tgt
arCOG00998	Yes	COG01958	LSM1
arCOG00999	Yes	COG01958	LSM1
arCOG01000	Yes	COG00006	PepP
arCOG01001	Yes	COG00024	Map
arCOG01004	no	COG02421	
arCOG01007	Yes	COG00609	FepD

arCOG01008	no	COG00864	NikR
arCOG01009	Yes	COG03609	
arCOG01010	Yes	COG02161	StbD
arCOG01013	no	COG04352	
arCOG01015	Yes	COG01258	
arCOG01016	Yes	COG01460	Rpo4
arCOG01018	Yes	COG00565	LasT
arCOG01019	Yes	COG04080	
arCOG01021	no	COG00683	LivK
arCOG01025	Yes	COG01685	
arCOG01026	Yes	COG01907	
arCOG01027	Yes	COG00083	ThrB
arCOG01028	Yes	COG01577	ERG12
arCOG01032	Yes	COG03890	ERG8
arCOG01033	Yes	COG00169	AroE
arCOG01034	Yes	COG01618	
arCOG01035	Yes	COG02423	
arCOG01036	Yes	COG00373	HemA
arCOG01037	Yes	COG01102	Cmk
arCOG01038	Yes	COG01936	
arCOG01039	Yes	COG02019	AdkA
arCOG01042	Yes	COG01325	
arCOG01043	Yes	COG01931	
arCOG01044	Yes	COG01648	CysG
arCOG01045	Yes	COG00237	CoaE
arCOG01049	Yes	COG02876	AroA
arCOG01051	Yes	COG03958	
arCOG01052	no	COG00022	AcoB
arCOG01053	Yes	COG03959	
arCOG01054	no	COG01071	AcoA
arCOG01055	Yes	COG03432	
arCOG01057	no	COG01733	
arCOG01064	Yes	COG00446	HcaD
arCOG01065	Yes	COG00446	HcaD
arCOG01067	no	COG01252	Ndh
arCOG01068	Yes	COG01249	Lpd
arCOG01069	Yes	COG00446	
arCOG01070	no	COG00446	HcaD
arCOG01072	Yes	COG00494	MutT
arCOG01073	Yes	COG00494	MutT
arCOG01074	Yes	COG00494	MutT
arCOG01075	Yes	COG01051	
arCOG01084	Yes	COG01694	MazG
arCOG01085	Yes	COG01646	
arCOG01086	Yes	COG00159	TrpA
arCOG01087	Yes	COG00149	TpiA
arCOG01088	Yes	COG00134	TrpC
arCOG01091	Yes	COG01814	

arCOG01093	no	COG02406	
arCOG01096	Yes	COG01814	
arCOG01097	Yes	COG01592	
arCOG01098	Yes		
arCOG01106	Yes		
arCOG01107	Yes	COG00624	ArgE
arCOG01108	no	COG01473	AbgB
arCOG01109	no	COG00624	ArgE
arCOG01110	Yes	COG00624	ArgE
arCOG01111	Yes	COG00574	PpsA
arCOG01114	no	COG00574	PpsA
arCOG01115	Yes	COG01571	TiaS
arCOG01117	Yes	COG01522	Lrp
arCOG01119	no	COG04274	
arCOG01120	Yes	COG05431	
arCOG01122	Yes	COG00120	RpiA
arCOG01123	Yes	COG00182	
arCOG01128	no	COG00819	TenA
arCOG01129	no	COG00819	TenA
arCOG01130	Yes	COG00436	
arCOG01136	Yes	COG00073	ARC1
arCOG01137	no	COG00824	FcbC
arCOG01138	Yes	COG01310	
arCOG01141	no	COG00622	
arCOG01143	Yes	COG00639	ApaH
arCOG01145	Yes	COG02129	
arCOG01147	Yes	COG01409	
arCOG01149	no		
arCOG01150	Yes	COG01407	
arCOG01154	Yes	COG04186	
arCOG01159	Yes	COG01340	
arCOG01163	Yes	COG00473	LeuB
arCOG01164	Yes	COG00538	Icd
arCOG01165	Yes	COG01389	
arCOG01167	Yes	COG01529	CoxL
arCOG01168	no	COG04948	
arCOG01169	Yes	COG00148	Eno
arCOG01171	Yes	COG00467	RAD55
arCOG01179	Yes	COG00361	InfA
arCOG01180	Yes	COG01718	RIO1
arCOG01181	Yes	COG00478	
arCOG01182	Yes	COG02112	
arCOG01183	Yes	COG00533	Kae1/QRI7
arCOG01185	Yes	COG03642	
arCOG01186	Yes	COG03642	
arCOG01189	Yes	COG00661	AarF
arCOG01191	Yes	COG02250	
arCOG01192	Yes	COG02250	

arCOG01195	no	COG01708	
arCOG01197	no	COG01708	
arCOG01199	no	COG01708	
arCOG01200	Yes	COG01708	
arCOG01202	no	COG01708	
arCOG01203	Yes	COG01708	
arCOG01204	Yes	COG01708	
arCOG01205	no	COG01708	
arCOG01212	Yes		
arCOG01213	Yes	COG00561	Cof
arCOG01217	Yes	COG01400	SEC65
arCOG01218	Yes	COG00526	TrxA
arCOG01219	Yes	COG01867	TRM1
arCOG01221	Yes	COG01986	
arCOG01222	Yes	COG00615	TagD
arCOG01224	Yes	COG01849	
arCOG01225	Yes	COG01100	
arCOG01226	Yes	COG01703	ArgK
arCOG01227	Yes	COG00552	FtsY
arCOG01228	Yes	COG00541	Ffh
arCOG01231	no	COG00378	HypB
arCOG01235	no	COG01622	CyoA
arCOG01236	no	COG01622	CyoA
arCOG01237	no	COG00843	CyoB
arCOG01238	Yes	COG00843	CyoB/DoxB
arCOG01241	Yes	COG00582	XerC
arCOG01244	no	COG04342	
arCOG01247	no	COG04974	XerD
arCOG01252	Yes	COG01012	PutA
arCOG01254	Yes	COG02872	
arCOG01255	Yes	COG00013	AlaS
arCOG01256	no	COG02872	
arCOG01257	Yes	COG00459	GroL
arCOG01259	no	COG01028	FabG
arCOG01260	Yes	COG01028	FabG
arCOG01262	no	COG01028	FabG
arCOG01263	Yes	COG00300	DltE
arCOG01268	Yes	COG01758	Rpo6/RpoZ
arCOG01269	no	COG00559	LivH
arCOG01273	no	COG04177	LivM
arCOG01278	Yes	COG00183	PaaJ
arCOG01279	no	COG00183	PaaJ
arCOG01280	no	COG00183	PaaJ
arCOG01281	no	COG00183	PaaJ
arCOG01282	Yes	COG00183	PaaJ
arCOG01283	no	COG01545	
arCOG01284	no	COG01545	
arCOG01285	Yes	COG01545	

arCOG01287	no	COG01545	
arCOG01288	no	COG01545	
arCOG01289	no	COG01545	
arCOG01290	no	COG01533	SplB
arCOG01292	no	COG00493	GltD
arCOG01296	Yes	COG00492	TrxB
arCOG01299	no	COG00446	HcaD
arCOG01302	Yes	COG01531	
arCOG01303	Yes	COG00509	GcvH
arCOG01304	no	COG01637	NucS
arCOG01306	Yes	COG01222	RPT1
arCOG01307	Yes	COG00464	Vps4
arCOG01308	Yes	COG00464	Cdc48
arCOG01314	no		
arCOG01316	Yes	COG00591	PutP
arCOG01320	Yes	COG00108	RibB
arCOG01322	Yes	COG01731	
arCOG01323	Yes	COG00054	RibH
arCOG01324	Yes	COG02820	Udp
arCOG01325	no	COG00813	DeoD
arCOG01327	Yes	COG00005	Pnp
arCOG01331	Yes	COG00501	HtpX
arCOG01333	no	COG00501	HtpX
arCOG01334	no	COG00501	HtpX
arCOG01336	Yes	COG02078	AMMECR1
arCOG01337	Yes	COG00045	SucC
arCOG01338	Yes	COG01042	
arCOG01339	Yes	COG00074	SucD
arCOG01340	Yes	COG01042	
arCOG01341	Yes	COG01730	GIM5
arCOG01342	Yes	COG01382	GimC
arCOG01344	Yes	COG02238	RPS19A
arCOG01346	Yes	COG01534	
arCOG01347	Yes	COG01793	CDC9
arCOG01348	Yes	COG00061	nadF
arCOG01349	Yes	COG00483	SuhB
arCOG01350	Yes	COG03199	
arCOG01351	Yes	COG00460	ThrA
arCOG01352	Yes	COG00334	GdhA
arCOG01354	Yes	COG02892	
arCOG01356	no	COG01032	
arCOG01357	Yes	COG01032	
arCOG01358	Yes	COG00621	MiaB
arCOG01361	no	COG01243	ELP3
arCOG01365	Yes	COG01369	POP5
arCOG01367	Yes	COG01091	RfbD
arCOG01369	Yes	COG00451	WcaG
arCOG01371	Yes	COG01088	RfbB

arCOG01377	Yes	COG03379	
arCOG01378	Yes	COG01524	
arCOG01381	no	COG00463	WcaA
arCOG01383	no	COG01216	
arCOG01385	Yes	COG00463	WcaA
arCOG01389	Yes	COG01215	
arCOG01391	Yes	COG01215	
arCOG01394	Yes	COG00707	MurG
arCOG01397	Yes	COG00463	WcaA
arCOG01398	no	COG00463	WcaA
arCOG01400	Yes	COG00500	
arCOG01401	no	COG00500	
arCOG01402	no	COG00500	
arCOG01403	no	COG00438	RfaG
arCOG01407	no	COG00438	RfaG
arCOG01408	no	COG00438	RfaG
arCOG01409	no	COG00438	RfaG
arCOG01411	Yes	COG00438	RfaG
arCOG01417	no	COG00438	RfaG
arCOG01419	Yes	COG00438	RfaG
arCOG01420	Yes	COG00297	GlgA
arCOG01421	Yes	COG00058	GlgP
arCOG01422	no	COG05493	
arCOG01424	no	SC.00001	
arCOG01425	no	SC.00001	
arCOG01426	no	SC.00001	
arCOG01430	Yes	COG00031	CysK
arCOG01431	Yes	COG01171	IlvA
arCOG01432	Yes	COG01350	
arCOG01434	Yes	COG00498	ThrC
arCOG01439	no	COG05551	Cas6
arCOG01440	Yes	COG05551	Cas6
arCOG01442	no	COG02254	
arCOG01443	no	COG02254	Cas3''
arCOG01444	no	COG01203	Cas3
arCOG01446	Yes	COG00640	ArsR
arCOG01452	Yes	COG01518	Cas1
arCOG01455	Yes	COG01064	AdhP
arCOG01456	Yes	COG01062	AdhC
arCOG01457	Yes	COG01064	AdhP
arCOG01458	no	COG00604	Qor
arCOG01459	Yes	COG01063	Tdh
arCOG01462	Yes	COG01668	NatB
arCOG01463	Yes	COG00842	
arCOG01465	no	COG00842	
arCOG01467	Yes	COG00842	
arCOG01468	no	COG00842	
arCOG01469	no	COG00842	

arCOG01471	no	COG03945	
arCOG01477	no	COG01230	CzcD
arCOG01478	no	COG00053	MMT1
arCOG01481	Yes	COG01488	PncB
arCOG01482	Yes	COG00157	NadC
arCOG01484	Yes	COG01985	RibD
arCOG01491	Yes	COG00243	BisC
arCOG01492	Yes	COG03383	
arCOG01495	no	COG00243	BisC
arCOG01496	Yes	COG00243	BisC
arCOG01500	Yes	COG00437	HybA
arCOG01503	no	COG00437	HybA
arCOG01505	Yes	COG03381	TorD
arCOG01506	no	COG03381	TorD
arCOG01510	Yes	COG01599	RFA1
arCOG01511	no	COG00145	HyuA
arCOG01512	no	COG00146	HyuB
arCOG01521	Yes	COG01233	
arCOG01526	Yes	COG01110	
arCOG01527	Yes	COG00550	TopA
arCOG01529	Yes	COG00365	Acs
arCOG01530	Yes	COG00315	MoaC
arCOG01531	Yes		
arCOG01532	Yes	COG00020	UppS
arCOG01533	no	COG00747	DdpA
arCOG01534	no	COG00747	DdpA
arCOG01535	no	COG00747	DdpA
arCOG01537	Yes	COG00651	NuoM
arCOG01539	Yes	COG01009	NuoL
arCOG01540	Yes	COG01007	NuoN
arCOG01543	Yes	COG01143	NuoI
arCOG01545	Yes	COG00650	HyfC
arCOG01546	Yes	COG01005	NuoH
arCOG01547	Yes	COG03261	HycE
arCOG01548	Yes	COG00649	NuoD
arCOG01551	Yes	COG00852	NuoC
arCOG01553	Yes	COG03260	
arCOG01554	Yes	COG00377	NuoB
arCOG01557	Yes	COG00838	NuoA
arCOG01559	Yes	COG00480	FusA
arCOG01560	Yes	COG00532	InfB
arCOG01561	Yes	COG05256	TEF1
arCOG01562	Yes	COG05258	GTPBP1
arCOG01563	Yes	COG05257	GCD11
arCOG01564	Yes	COG03276	SelB
arCOG01568	Yes	COG00668	MscS
arCOG01572	Yes		
arCOG01573	Yes	COG00668	MscS

arCOG01574	Yes	COG02123	
arCOG01575	Yes	COG00689	Rph
arCOG01576	Yes	COG02217	ZntA
arCOG01578	Yes	COG00474	MgtA
arCOG01580	Yes	COG01522	Lrp
arCOG01585	no	COG01522	Lrp
arCOG01586	no	COG01522	Lrp
arCOG01587	no	COG01522	Lrp
arCOG01588	Yes		LysW
arCOG01589	Yes	COG00189	RimK
arCOG01590	Yes	COG00439	AccC
arCOG01594	Yes	COG00458	CarB
arCOG01597	Yes	COG00026	PurK
arCOG01598	Yes	COG00027	PurT
arCOG01599	Yes	COG01013	PorB
arCOG01601	Yes	COG01013	PorB
arCOG01602	no	COG01014	PorG
arCOG01603	Yes	COG01014	PorG
arCOG01604	no	COG01144	
arCOG01605	Yes	COG01144	
arCOG01606	Yes	COG00674	PorA
arCOG01608	Yes	COG00674	PorA
arCOG01609	no	COG04231	
arCOG01615	Yes	COG02431	
arCOG01616	Yes	COG01650	GEK1
arCOG01617	Yes	COG00667	Tas
arCOG01618	no	COG00656	ARA1
arCOG01619	Yes	COG00656	ARA1
arCOG01622	no	COG00673	MviM
arCOG01628	Yes	COG01522	Lrp
arCOG01630	Yes	COG00251	TdcF
arCOG01631	Yes	COG00500	
arCOG01640	Yes	COG01601	GCD7
arCOG01646	Yes	COG01506	DAP2
arCOG01647	no	COG01505	
arCOG01648	Yes	COG00596	MhpC
arCOG01659	no	COG00412	
arCOG01663	Yes	COG02026	RelE
arCOG01665	no	COG02026	RelE
arCOG01667	Yes	COG03369	
arCOG01668	no	COG03370	
arCOG01669	Yes	COG03370	
arCOG01670	no	COG02044	
arCOG01671	Yes	COG00043	UbiD
arCOG01672	Yes	COG03889	
arCOG01673	no	COG00747	DdpA
arCOG01674	Yes	COG01254	AcyP
arCOG01676	Yes	COG00476	ThiF

arCOG01678	Yes	COG01812	MetK
arCOG01679	Yes	COG00640	ArsR
arCOG01681	no	COG00640	ArsR
arCOG01695	Yes	COG01293	
arCOG01696	Yes	COG03635	
arCOG01697	Yes	COG01048	AcnA
arCOG01698	Yes	COG00065	LeuC
arCOG01699	Yes	COG04353	
arCOG01700	Yes	COG00010	SpeB
arCOG01701	Yes	COG01676	SEN2
arCOG01702	Yes	COG01676	SEN2
arCOG01703	Yes	COG00163	UbiX
arCOG01704	Yes	COG00452	Dfp
arCOG01706	no	COG00508	AceF
arCOG01707	Yes	COG01960	CaiA
arCOG01710	Yes	COG02185	Sbm
arCOG01711	Yes	COG00221	Ppa
arCOG01713	Yes	COG05440	
arCOG01714	Yes	COG05440	
arCOG01715	Yes	COG00719	sufB
arCOG01717	Yes	COG00154	GatA
arCOG01718	Yes	COG00064	GatB
arCOG01719	Yes	COG02511	GatE
arCOG01720	Yes	COG00723	QcrA
arCOG01721	Yes	COG01290	QcrB
arCOG01722	Yes	COG00099	RpsM
arCOG01723	Yes	COG01437	CyaB
arCOG01726	Yes	COG00142	IspA
arCOG01727	Yes	COG00142	IspA
arCOG01728	Yes	COG01355	
arCOG01730	no	COG03384	
arCOG01736	Yes	COG01514	LigT
arCOG01739	Yes	COG00681	LepB
arCOG01740	no	COG00681	LepB
arCOG01741	Yes	COG01537	PelA
arCOG01742	Yes	COG01503	eRF1
arCOG01747	Yes	COG00015	PurB
arCOG01748	Yes	COG00165	ArgH
arCOG01749	Yes	COG00114	FumC
arCOG01751	Yes	COG01358	RPL8A
arCOG01752	Yes	COG01911	RPL30
arCOG01753	Yes	COG01581	Ssh10b
arCOG01754	Yes	COG00111	SerA
arCOG01755	Yes	COG01052	LdhA
arCOG01758	Yes	COG00051	RpsJ
arCOG01759	Yes	COG01259	
arCOG01760	Yes	COG00195	NusA
arCOG01761	Yes	COG00195	NusA

arCOG01762	Yes	COG00085	RpoB/Rpo2
arCOG01764	Yes	COG02101	SPT15
arCOG01767	Yes	COG03425	PksG
arCOG01768	Yes	COG00705	GlpG
arCOG01773	Yes	COG00500	
arCOG01776	Yes	COG00500	
arCOG01786	no	COG00500	
arCOG01790	no	COG00500	
arCOG01791	Yes	COG00500	
arCOG01804	no	COG03888	
arCOG01808	Yes	COG02064	TadC
arCOG01809	no	COG01955	FlaJ
arCOG01815	Yes	COG02064	TadC
arCOG01817	Yes	COG00630	VirB11
arCOG01822	no	COG03354	FlaG
arCOG01824	no	COG03353	FlaF
arCOG01829	no	COG01681	FlaB
arCOG01830	Yes	COG01665	
arCOG01831	Yes	COG01665	
arCOG01832	Yes	COG00071	IbpA
arCOG01833	Yes	COG00071	IbpA
arCOG01840	Yes	COG00071	IbpA
arCOG01842	no	COG03255	
arCOG01845	Yes	COG02151	PaaD
arCOG01849	Yes	COG00663	PaaY
arCOG01852	no	COG02151	PaaD
arCOG01857	Yes	COG01303	
arCOG01863	Yes	COG01813	
arCOG01868	Yes	COG00735	Fur
arCOG01870	no	COG01865	cbiZ
arCOG01871	Yes	COG02266	
arCOG01872	Yes	COG00746	MobA
arCOG01873	Yes	COG02068	MobA
arCOG01875	Yes	COG01475	
arCOG01876	Yes	COG00620	MetE
arCOG01877	Yes	COG00620	MetE
arCOG01879	Yes	COG00170	
arCOG01883	Yes	COG01351	THY1
arCOG01885	Yes	COG01383	RPS17A
arCOG01886	Yes	COG00162	TyrS
arCOG01887	Yes	COG00180	TrpS
arCOG01891	Yes	COG00125	Tmk
arCOG01894	Yes	COG00648	Nfo
arCOG01896	no	COG01082	IolE
arCOG01902	no	COG01082	IolE
arCOG01904	Yes	COG01339	
arCOG01905	no	COG04189	
arCOG01907	Yes	COG02013	

arCOG01909	Yes	COG00174	GlnA
arCOG01910	no	COG01030	NfeD
arCOG01912	no	COG01585	
arCOG01915	no	COG00330	HflC
arCOG01917	no		
arCOG01918	Yes	COG00330	HflC
arCOG01919	Yes	COG00805	TatC
arCOG01920	Yes	COG00250	NusG
arCOG01921	Yes	COG02083	
arCOG01923	Yes	COG01498	SIK1
arCOG01924	Yes	COG00252	AnsB
arCOG01925	Yes	COG02080	CoxS
arCOG01926	Yes	COG01319	CoxM
arCOG01927	Yes	COG03427	CoxG
arCOG01928	Yes	COG03427	
arCOG01929	Yes	COG01975	XdhC
arCOG01930	no	COG02159	
arCOG01931	no	COG02159	
arCOG01932	no	COG02159	
arCOG01933	no	COG02159	
arCOG01934	no	COG02159	
arCOG01935	no	COG04006	
arCOG01937	no	COG01750	
arCOG01939	Yes	COG00095	LplA
arCOG01940	Yes	COG00340	BirA
arCOG01943	Yes	COG01335	PncA
arCOG01946	Yes	COG02125	RPS6A
arCOG01947	Yes	COG01280	RhtB
arCOG01950	Yes	COG02075	RPL24A
arCOG01951	Yes	COG01444	
arCOG01952	Yes	COG00009	SUA5
arCOG01953	Yes	COG00475	KefB
arCOG01956	no	COG00475	KefB
arCOG01958	no	COG01226	Kch
arCOG01969	no	COG00475	KefB
arCOG01972	Yes	COG00526	TrxA
arCOG01978	Yes	COG00294	FolP
arCOG01981	Yes	COG01405	SUA7
arCOG01982	no	COG00434	SgcQ
arCOG01983	Yes	COG00135	TrpF
arCOG01984	no	COG02440	FixX
arCOG01985	no	COG02440	FixX
arCOG01987	no	COG01788	AtoD
arCOG01988	Yes	COG02092	EFB1
arCOG01989	Yes	COG02888	
arCOG01991	Yes	COG00406	phoE
arCOG01992	Yes	COG02062	SixA
arCOG01995	Yes	COG02867	

arCOG01997	Yes	COG02095	MarC
arCOG01998	Yes	COG00028	IlvB
arCOG01999	no	COG00028	
arCOG02001	no	COG00028	IlvB
arCOG02004	no	COG03473	
arCOG02007	Yes	COG01331	
arCOG02008	no	COG03371	
arCOG02012	Yes	COG00547	TrpD
arCOG02014	Yes	COG00147	TrpE
arCOG02017	no	COG01853	
arCOG02019	no	COG02897	SseA
arCOG02021	Yes	COG00607	PspE
arCOG02026	no	COG05557	
arCOG02027	Yes	COG03301	NrfD
arCOG02037	Yes	COG01378	
arCOG02044	Yes	COG01287	
arCOG02048	Yes	COG01587	HemD
arCOG02050	Yes	COG00730	
arCOG02053	Yes	COG00589	UspA
arCOG02055	no	COG00155	CysI
arCOG02062	Yes	COG00425	SirA
arCOG02064	Yes	COG02210	
arCOG02065	no	COG02210	
arCOG02066	Yes	COG02044	
arCOG02067	Yes	COG02044	
arCOG02072	Yes	COG00425	SirA
arCOG02074	no	COG00425	SirA
arCOG02076	no	COG02044	
arCOG02089	Yes	COG01361	
arCOG02092	Yes	COG00119	LeuA
arCOG02097	Yes	COG00710	AroD
arCOG02098	Yes	COG01605	PheA
arCOG02099	Yes	COG01321	TroR
arCOG02100	Yes	COG01321	TroR
arCOG02103	Yes		
arCOG02108	no	COG02445	
arCOG02110	no	COG01708	
arCOG02113	no	COG02427	
arCOG02114	Yes	COG02427	
arCOG02115	Yes	COG02427	
arCOG02127	no	COG03335	
arCOG02129	no	COG03335	
arCOG02130	no	COG03335	
arCOG02133	no	COG01662	InsB
arCOG02134	Yes	COG03316	
arCOG02143	Yes	COG02368	
arCOG02149	no	COG00599	
arCOG02159	Yes	COG04089	

arCOG02165	no	COG01305	
arCOG02172	Yes	COG00720	
arCOG02173	Yes	COG00602	NrdG
arCOG02175	Yes	COG02409	
arCOG02187	Yes	COG01145	NapF
arCOG02199	Yes	COG00543	UbiB
arCOG02204	Yes	COG02443	Sss1
arCOG02207	Yes	COG00708	XthA
arCOG02208	Yes	COG00040	HisG
arCOG02209	no	COG02244	RfbX
arCOG02217	no	SC.00003	
arCOG02218	Yes	SC.00003	
arCOG02219	Yes	COG01487	VapC
arCOG02221	Yes	COG01848	
arCOG02222	no	COG01848	
arCOG02227	no	COG01487	VapC
arCOG02228	Yes	COG02246	
arCOG02230	Yes	COG00066	LeuD
arCOG02231	Yes	COG02509	
arCOG02234	Yes	COG01148	HdrA
arCOG02235	no	COG01148	HdrA
arCOG02236	no	COG01148	HdrA
arCOG02242	Yes	COG03355	
arCOG02244	no	COG02009	SdhC
arCOG02246	Yes	COG02138	
arCOG02247	Yes	COG02082	CobH
arCOG02250	no	COG00619	CbiQ
arCOG02266	Yes	COG00598	CorA
arCOG02267	Yes	COG00306	PitA
arCOG02271	Yes	COG03413	
arCOG02272	no	COG03413	
arCOG02273	no	COG03413	
arCOG02287	no	COG00393	
arCOG02291	Yes	COG01011	
arCOG02293	Yes	COG00637	
arCOG02298	Yes	COG01989	PulO
arCOG02304	no	COG01804	Mct/CaiB
arCOG02312	Yes	COG00577	SalY
arCOG02431	no	COG01611	
arCOG02441	Yes	COG01277	NosY
arCOG02455	Yes	COG00636	AtpK
arCOG02459	Yes	COG01527	NtpC
arCOG02462	no	COG01878	
arCOG02464	Yes	COG00041	PurE
arCOG02466	Yes	COG03277	GAR1
arCOG02527	no	COG03291	
arCOG02556	no	COG01520	
arCOG02560	no	COG03391	

arCOG02562	no	COG03391	
arCOG02569	no	COG00038	EriC
arCOG02586	no	COG00727	
arCOG02617	no		
arCOG02620	no	COG01541	PaaK
arCOG02623	Yes	COG01541	PaaK
arCOG02638	no	COG00657	Aes
arCOG02640	Yes	COG01392	
arCOG02647	no	COG01309	AcrR
arCOG02654	no	COG03337	
arCOG02656	no	COG03337	
arCOG02657	Yes	COG01336	Cas7/Cmr4
arCOG02658	no	COG01337	Cas7/Csm3
arCOG02659	no	COG01337	Cas7/Csm3
arCOG02660	no	COG01337	Cas7/Csm3
arCOG02661	no	COG01604	Cas7/Cmr6
arCOG02665	no	COG01769	Cas5/Cmr3
arCOG02666	no	COG01353	Cas10
arCOG02670	no	COG01688	Cas5
arCOG02676	Yes	COG00139	HisI
arCOG02677	Yes	COG00140	HisI
arCOG02681	Yes	COG01753	
arCOG02682	Yes	COG00477	ProP
arCOG02683	Yes	COG00477	ProP
arCOG02684	no	COG00477	ProP
arCOG02685	no	COG00477	ProP
arCOG02687	Yes	COG00477	ProP
arCOG02688	no	COG00477	ProP
arCOG02689	Yes	COG00477	ProP
arCOG02690	no	COG00477	ProP
arCOG02691	Yes	COG00477	ProP
arCOG02692	no	COG00477	ProP
arCOG02693	no	COG00477	ProP
arCOG02694	Yes	COG01826	TatA
arCOG02699	Yes	COG00511	AccB
arCOG02700	no	COG00508	AceF
arCOG02702	Yes	COG00500	
arCOG02703	no	COG00500	
arCOG02705	Yes	COG04799	
arCOG02706	Yes	COG00346	GloA
arCOG02718	Yes	COG02034	
arCOG02719	no	COG02034	
arCOG02724	Yes	COG00350	Ada
arCOG02726	Yes	COG00721	GatC
arCOG02727	Yes	COG01673	
arCOG02730	Yes	COG01848	
arCOG02731	no	COG01487	VapC
arCOG02734	Yes	COG01433	

arCOG02738	Yes	COG01433	
arCOG02739	no	COG01433	
arCOG02741	Yes	COG00422	ThiC
arCOG02754	no	COG03039	
arCOG02759	no	COG01943	
arCOG02761	no	COG03439	
arCOG02763	Yes	COG02217	
arCOG02764	no	COG02608	CopZ
arCOG02772	no	COG00348	NapH
arCOG02792	no	COG00477	ProP
arCOG02794	no	COG00477	ProP
arCOG02816	no	COG00225	MsrA
arCOG02833	no	COG00265	DegQ
arCOG02835	no	COG02324	
arCOG02840	no	COG00415	PhrB
arCOG02841	no	COG01132	MdlB
arCOG02852	Yes	COG02146	{NirD}
arCOG02853	Yes	COG02146	{NirD}
arCOG02854	no	COG02146	{NirD}
arCOG02855	no	COG02146	{NirD}
arCOG02860	Yes	COG00288	CynT
arCOG02862	Yes	COG02259	
arCOG02863	no	COG02259	
arCOG02869	Yes	COG02761	FrnE
arCOG02881	Yes	COG00530	ECM27
arCOG02884	no	COG04743	
arCOG02887	no	COG04743	
arCOG02900	Yes	COG02304	
arCOG02935	no	COG01741	
arCOG02936	no	COG01562	ERG9
arCOG02937	Yes	COG03407	MVD1
arCOG02939	no	COG02154	
arCOG02942	no	COG00328	RnhA
arCOG02949	no	COG01523	PulA
arCOG02951	no	COG00296	GlgB
arCOG02955	no	COG03280	TreY
arCOG02957	Yes	COG04023	SBH1
arCOG02960	Yes	COG04882	
arCOG02969	Yes	COG00308	PepN
arCOG02979	Yes		
arCOG02994	Yes	COG01917	
arCOG03000	Yes	COG03435	
arCOG03002	no	COG01917	
arCOG03013	Yes	COG02219	PRI2
arCOG03016	Yes	COG00451	WcaG
arCOG03031	no	COG03253	ywfI
arCOG03032	no	COG00457	NrfG
arCOG03038	Yes	COG00457	NrfG

arCOG03042	Yes	COG00457	NrfG
arCOG03056	no	COG00671	PgpB
arCOG03073	Yes	COG00713	NuoK
arCOG03108	no	COG02235	ArcA
arCOG03117	Yes	COG00586	DedA
arCOG03119	Yes		
arCOG03142	Yes		
arCOG03162	no	COG01961	PinR
arCOG03164	Yes	COG02452	
arCOG03166	no	COG01672	
arCOG03167	Yes	COG01373	
arCOG03168	no	COG01373	
arCOG03169	Yes	COG01672	
arCOG03176	no	COG01584	
arCOG03181	no	COG01846	MarR
arCOG03182	Yes	COG01846	MarR
arCOG03199	Yes	COG00472	Rfe
arCOG03202	Yes	COG00826	
arCOG03216	Yes	COG02014	
arCOG03229	Yes	COG02454	
arCOG03233	Yes	COG04938	
arCOG03237	no	COG01106	
arCOG03239	Yes	COG01106	
arCOG03240	no	COG01195	RecF
arCOG03247	Yes	COG02136	IMP4
arCOG03271	Yes	COG02105	
arCOG03278	Yes	COG01449	
arCOG03284	no	COG01449	
arCOG03285	Yes	COG03387	SGA1
arCOG03286	no	COG03387	SGA1
arCOG03287	Yes	COG03408	GDB1
arCOG03358	no	COG03355	
arCOG03384	no	COG04946	
arCOG03413	Yes	COG02453	CDC14
arCOG03423	no	COG01846	MarR
arCOG03428	Yes	COG00457	NrfG
arCOG03431	no	COG04279	
arCOG03433	no	COG01517	
arCOG03436	Yes	COG01517	
arCOG03437	no	COG01517	
arCOG03447	no	COG01457	CodB
arCOG03448	no	COG01953	FUI1
arCOG03460	no	COG02120	
arCOG03462	no	COG00531	PotE
arCOG03463	no	COG00531	PotE
arCOG03464	no	COG00531	PotE
arCOG03465	Yes	COG00531	PotE
arCOG03466	no	COG00531	PotE

arCOG03482	Yes		Cas7/Csc2
arCOG03483	Yes	COG01203	Cas3d
arCOG03560	no		
arCOG03580	no	COG02020	STE14
arCOG03586	no	COG03547	
arCOG03587	no	COG03547	
arCOG03617	no	COG01857	Cas7
arCOG03626	Yes	COG04237	HyfE
arCOG03639	Yes	COG00121	
arCOG03649	no	COG00531	PotE
arCOG03650	no	COG00531	PotE
arCOG03651	no	COG00531	PotE
arCOG03652	Yes	COG00531	PotE
arCOG03654	Yes	COG00531	PotE
arCOG03656	no	COG00028	
arCOG03657	Yes	COG00028	
arCOG03658	Yes	COG00208	NrdF
arCOG03659	Yes	COG02971	
arCOG03660	Yes	COG00477	ProP
arCOG03661	no	COG00383	AMS1
arCOG03663	no	COG01501	
arCOG03665	Yes	COG04934	
arCOG03669	no	COG04934	
arCOG03670	Yes	SC.00065	
arCOG03671	Yes		
arCOG03672	Yes	SC.00065	
arCOG03673	no		
arCOG03674	Yes	SC.00065	
arCOG03682	Yes	COG00515	SPS1
arCOG03686	Yes	COG01765	
arCOG03692	Yes	SC.00068	
arCOG03693	no	SC.00068	
arCOG03694	Yes		
arCOG03696	Yes	COG00348	NapH
arCOG03698	Yes	COG04344	
arCOG03699	Yes		
arCOG03700	no		SoxE
arCOG03701	no	COG05650	
arCOG03703	no	COG05650	
arCOG03704	no		
arCOG03705	Yes	COG01547	
arCOG03706	no	COG01848	
arCOG03707	no	SC.00069	
arCOG03708	Yes	SC.00069	
arCOG03712	Yes	SC.00069	
arCOG03713	Yes	COG00209	NrdA
arCOG03714	no	COG01328	NrdD
arCOG03721	Yes	SC.00071	

arCOG03722	no	SC.00071	
arCOG03724	no		
arCOG03737	Yes		
arCOG03738	Yes		
arCOG03743	no	COG05550	
arCOG03748	no	COG01846	MarR
arCOG03749	no	COG04243	
arCOG03765	Yes	SC.00090	
arCOG03768	Yes		
arCOG03769	no		
arCOG03770	Yes		
arCOG03772	Yes	COG04345	
arCOG03778	Yes		
arCOG03779	Yes	COG01401	McrB
arCOG03823	no	SC.00102	
arCOG03824	no		
arCOG03837	Yes	COG00095	
arCOG03838	Yes		PqqD
arCOG03840	no	COG03535	
arCOG03841	no	COG03535	
arCOG03842	no	COG00526	
arCOG03843	no	SC.00105	
arCOG03844	Yes	SC.00105	
arCOG03845	Yes	COG03609	
arCOG03846	Yes	COG03609	
arCOG03847	no	COG01517	
arCOG03849	Yes		
arCOG03850	no	COG04122	
arCOG03851	Yes		
arCOG03852	Yes		
arCOG03853	Yes		
arCOG03854	Yes		
arCOG03855	Yes		
arCOG03856	no	SC.00106	
arCOG03857	no	SC.00106	
arCOG03858	no		
arCOG03859	no	COG00640	ArsR
arCOG03861	Yes		
arCOG03862	no		
arCOG03863	no	COG04354	
arCOG03864	no	COG04354	
arCOG03865	no	COG04354	
arCOG03867	no	COG04354	
arCOG03868	no	SC.00107	
arCOG03869	Yes	SC.00107	
arCOG03871	Yes	SC.00076	
arCOG03872	no		
arCOG03879	Yes	COG03682	

arCOG03882	Yes	COG02003	
arCOG03891	no	COG01367	Cas7/Cmr1
arCOG03893	no	COG01367	Cas7/Cmr1
arCOG03902	no	COG03385	
arCOG04004	no	COG00666	Arp
arCOG04017	no		
arCOG04025	no		
arCOG04027	no	COG01725	
arCOG04028	no	COG05550	
arCOG04029	no	COG05550	
arCOG04030	no	SC.00125	
arCOG04031	no	SC.00125	
arCOG04032	no		
arCOG04033	no		
arCOG04035	no	COG03451	VirB4
arCOG04039	no		
arCOG04044	Yes	COG01830	
arCOG04045	Yes	COG00129	IlvD
arCOG04046	Yes	COG04996	
arCOG04048	Yes	COG00717	Dcd
arCOG04049	Yes	COG01552	RPL40A
arCOG04050	Yes	COG00258	Exo
arCOG04051	Yes	COG02412	
arCOG04053	Yes	COG00405	Ggt
arCOG04054	Yes		
arCOG04055	Yes	COG01371	
arCOG04056	no	COG00640	ArsR
arCOG04059	Yes		
arCOG04060	Yes	COG01709	
arCOG04061	Yes	COG01308	EGD2
arCOG04062	Yes		
arCOG04064	Yes	COG00750	
arCOG04066	Yes	COG02413	
arCOG04067	Yes	COG00090	RplB
arCOG04068	Yes		
arCOG04069	Yes	COG02106	
arCOG04070	Yes	COG00087	RplC
arCOG04071	Yes	COG00088	RplD
arCOG04072	Yes	COG00089	RplW
arCOG04075	Yes	COG00214	SNZ1
arCOG04076	Yes	COG01909	
arCOG04077	Yes	COG02093	Spt4
arCOG04081	Yes	COG01224	TIP49
arCOG04082	no	COG02366	
arCOG04083	no	COG02355	
arCOG04086	Yes	COG01841	RpmD
arCOG04087	Yes	COG00098	RpsE
arCOG04088	Yes	COG00256	RplR

arCOG04089	Yes	COG02147	RPL19A
arCOG04090	Yes	COG00097	RplF
arCOG04091	Yes	COG00096	RpsH
arCOG04092	Yes	COG00094	RplE
arCOG04093	Yes	COG01471	RPS4A
arCOG04094	Yes	COG00198	RplX
arCOG04095	Yes	COG00093	RplN
arCOG04096	Yes	COG00186	RpsQ
arCOG04097	Yes	COG00092	RpsC
arCOG04098	Yes	COG00091	RplV
arCOG04099	Yes	COG00185	RpsS
arCOG04100	no	COG01960	CaiA
arCOG04101	Yes	COG01394	NtpD
arCOG04102	Yes	COG01436	NtpG
arCOG04103	Yes		
arCOG04104	Yes		
arCOG04105	Yes		
arCOG04106	Yes	COG00575	CdsA
arCOG04107	Yes	COG01093	SUI2
arCOG04108	Yes	COG02051	RPS27A
arCOG04109	Yes	COG01631	RPL42A
arCOG04110	Yes	COG01467	PRI1
arCOG04111	Yes	COG01761	RPB11
arCOG04112	Yes	COG01736	DPH2
arCOG04113	Yes	COG00197	RplP
arCOG04114	Yes		
arCOG04115	Yes	COG01489	SfsA
arCOG04116	Yes	COG01855	
arCOG04119	Yes	COG04914	
arCOG04120	no	COG00469	PykF
arCOG04121	Yes	COG00164	RnhB
arCOG04122	Yes	COG01756	Mra1
arCOG04123	Yes	COG04755	
arCOG04124	Yes	COG02835	
arCOG04125	Yes	COG00430	RCL1
arCOG04126	Yes	COG02126	RPL37A
arCOG04127	Yes	COG01341	
arCOG04128	no	COG00035	Upp
arCOG04129	Yes	COG02139	RPL21A
arCOG04130	Yes	COG01491	
arCOG04131	Yes	COG00030	KsgA
arCOG04132	no	COG04697	
arCOG04133	Yes	COG00082	AroC
arCOG04134	Yes	COG00128	AroA
arCOG04135	Yes		
arCOG04136	Yes	COG04888	
arCOG04137	Yes	COG00499	SAM1
arCOG04138	Yes	COG01269	NtpI

arCOG04140	Yes	COG01888	
arCOG04142	Yes	COG01899	DYS1
arCOG04143	Yes	COG01697	
arCOG04147	Yes	COG00605	SodA
arCOG04148	no	COG02874	FlaH
arCOG04149	Yes	COG01499	NMD3
arCOG04150	Yes	COG01094	
arCOG04151	Yes	COG02237	
arCOG04152	Yes	COG01395	
arCOG04153	Yes	COG01522	
arCOG04154	Yes	COG02007	RPS8A
arCOG04156	Yes	COG01590	TYW3
arCOG04157	Yes	COG00270	Dcm
arCOG04160	Yes		
arCOG04161	Yes	COG01798	DPH5
arCOG04164	Yes	COG04034	
arCOG04165	Yes		
arCOG04166	Yes	COG04879	
arCOG04167	Yes	COG02163	RPL14A
arCOG04168	no	COG02174	RPL34A
arCOG04169	Yes	COG00201	SecY
arCOG04170	Yes	COG02379	GckA
arCOG04171	Yes	COG02090	
arCOG04172	Yes	COG00329	DapA
arCOG04173	no	COG00295	Cdd
arCOG04174	Yes	COG00731	TYW3
arCOG04175	Yes	COG02157	RPL20A
arCOG04176	Yes	COG01976	TIF6
arCOG04177	Yes	COG02167	RPL39
arCOG04179	Yes	COG02118	
arCOG04180	Yes	COG01980	
arCOG04181	Yes	COG04046	
arCOG04182	Yes	COG02004	RPS24A
arCOG04183	Yes	COG01998	RPS31
arCOG04184	Yes	COG00127	
arCOG04185	Yes	COG00184	RpsO
arCOG04186	Yes	COG01890	RPS1A
arCOG04187	Yes	COG01500	
arCOG04188	Yes	COG01898	RfbC
arCOG04191	no	COG02046	MET3
arCOG04194	no	COG01343	Cas2
arCOG04195	no	COG04343	
arCOG04197	no		
arCOG04201	no	COG00365	Acs
arCOG04202	Yes	COG02429	
arCOG04204	no	COG03342	
arCOG04206	Yes	COG01948	MUS81
arCOG04208	Yes	COG01997	RPL43A

arCOG04209	Yes	COG01632	RPL15A
arCOG04213	Yes	COG01260	INO1
arCOG04214	Yes	COG00432	
arCOG04216	no		
arCOG04217	Yes	COG04900	
arCOG04219	Yes	COG02433	
arCOG04221	Yes	COG00647	NagD
arCOG04223	Yes	COG00023	SUI1
arCOG04224	Yes		
arCOG04225	no	COG02110	
arCOG04227	Yes	COG01832	
arCOG04228	Yes	COG01990	pth2
arCOG04229	Yes	COG01781	PyrI
arCOG04231	Yes	COG01324	CutA
arCOG04232	Yes	COG01884	Sbm
arCOG04233	Yes	COG00614	FepB
arCOG04234	Yes		
arCOG04235	Yes	COG05494	
arCOG04236	no	COG00396	sufC
arCOG04237	Yes	COG00372	GltA
arCOG04238	Yes	COG01767	CitG
arCOG04239	Yes	COG00522	RpsD
arCOG04240	Yes	COG00100	RpsK
arCOG04241	Yes	COG00202	RpoA/Rpo1
arCOG04242	Yes	COG00102	RplM
arCOG04243	Yes	COG00103	RpsI
arCOG04244	Yes	COG01644	RPB10
arCOG04245	Yes	COG00052	RpsB
arCOG04246	Yes	COG01690	RtcB
arCOG04247	Yes	COG02317	
arCOG04248	no	COG00846	SIR2
arCOG04249	Yes	COG01746	CCA1
arCOG04250	Yes	COG03382	
arCOG04251	Yes	COG05399	
arCOG04252	Yes	COG00585	
arCOG04253	Yes	COG01415	
arCOG04254	Yes	COG00049	RpsG
arCOG04255	Yes	COG00048	RpsL
arCOG04256	Yes	COG00086	RpoC/Rpo11
arCOG04257	Yes	COG00086	RpoC/Rpo3
arCOG04258	Yes	COG02012	RPB5
arCOG04260	Yes	COG01257	HMG1
arCOG04261	Yes		
arCOG04262	Yes	COG01701	
arCOG04263	Yes	COG01829	
arCOG04265	Yes	COG01779	
arCOG04266	Yes		
arCOG04268	Yes		Cdt1

arCOG04269	Yes	COG01602	
arCOG04270	Yes	COG01675	TFA1
arCOG04271	Yes		RPB8
arCOG04272	Yes	COG02038	CobT
arCOG04273	Yes	COG00079	HisC
arCOG04274	Yes	COG01270	CbiB
arCOG04276	Yes	COG00209	NrdA
arCOG04277	Yes	COG00231	Efp
arCOG04281	Yes	COG00358	DnaG
arCOG04283	Yes		
arCOG04285	no	COG02079	PrpD
arCOG04287	Yes	COG02058	RPP1A
arCOG04288	Yes	COG00244	RplJ
arCOG04289	Yes	COG00081	RplA
arCOG04290	Yes	COG01656	
arCOG04291	Yes	COG01801	
arCOG04293	Yes	COG04919	
arCOG04294	Yes	COG00468	RecA
arCOG04298	Yes	COG01839	
arCOG04299	Yes	COG00181	HemC
arCOG04300	Yes	COG00113	HemB
arCOG04302	Yes	COG00008	GlnS
arCOG04303	Yes	COG01634	
arCOG04305	Yes	COG04830	RPS26B
arCOG04307	Yes	COG01530	CafA
arCOG04308	Yes	COG01698	
arCOG04309	Yes	COG01912	
arCOG04310	no	COG01960	CaiA
arCOG04311	Yes	COG01896	
arCOG04312	Yes	COG01412	
arCOG04313	Yes	COG00105	Ndk
arCOG04314	Yes	COG02053	RPS28A
arCOG04318	Yes	COG02178	
arCOG04319	Yes	COG04920	
arCOG04321	Yes	COG02848	
arCOG04323	Yes		
arCOG04324	Yes		
arCOG04325	no	COG01702	PhoH
arCOG04327	Yes	COG04901	
arCOG04328	no	COG04028	
arCOG04329	Yes	COG02441	
arCOG04331	Yes	COG05496	
arCOG04333	no	COG00436	
arCOG04338	Yes	COG00368	CobS
arCOG04341	Yes	COG01996	RPC12
arCOG04345	Yes	COG02023	RPR2
arCOG04346	Yes	COG01759	
arCOG04348	Yes	COG02226	UbiE

arCOG04349	Yes	COG03374	
arCOG04350	no	COG01960	CaiA
arCOG04351	Yes	COG03356	
arCOG04352	Yes	COG00141	HisD
arCOG04355	no	COG01275	TehA
arCOG04356	no	COG03372	Bax1
arCOG04357	Yes	COG01059	
arCOG04358	no	COG01526	FdhD
arCOG04362	no	COG04742	
arCOG04372	Yes	COG00080	RplK
arCOG04373	Yes	COG00011	
arCOG04383	Yes	COG01903	CbiD
arCOG04386	no	COG01620	LldP
arCOG04387	Yes	COG00104	PurA
arCOG04394	no	COG01416	
arCOG04397	Yes	COG00004	AmtB
arCOG04398	Yes	COG00131	HisB
arCOG04409	Yes	COG02410	
arCOG04414	Yes	COG02892	Pcc1
arCOG04415	Yes	COG00151	PurD
arCOG04421	Yes	COG00152	PurC
arCOG04426	Yes	COG00375	HybF
arCOG04429	Yes	COG00680	HyaD
arCOG04430	Yes	COG01078	
arCOG04431	no	COG00580	GlpF
arCOG04435	Yes	COG01892	
arCOG04458	Yes	COG02457	
arCOG04459	Yes	COG00379	NadA
arCOG04462	Yes	COG01828	PurS
arCOG04465	Yes	COG00059	IlvC
arCOG04473	Yes	COG02097	RPL31A
arCOG04474	no		
arCOG04479	Yes	COG03357	
arCOG04480	Yes	COG02002	AbrB
arCOG04489	no	COG04689	
arCOG04502	Yes	COG02402	
arCOG04507	Yes	COG03350	
arCOG04527	no	COG00832	UreB
arCOG04528	no	COG00831	UreA
arCOG04530	no	COG00830	UreF
arCOG04531	Yes	COG01914	MntH
arCOG04536	Yes	COG01402	
arCOG04542	Yes	COG00302	FolE
arCOG04548	Yes	COG01146	
arCOG04558	Yes	COG01957	URH1
arCOG04582	no	COG00389	DinP
arCOG04583	no	COG00500	
arCOG04634	no	COG01472	BglX

arCOG04641	Yes	COG02860	
arCOG04654	Yes	COG00839	NuoJ
arCOG04697	no		
arCOG04702	Yes	COG01881	
arCOG04703	no	COG03256	NorB
arCOG04708	Yes	COG03410	
arCOG04733	Yes	COG02042	
arCOG04756	no	COG00679	
arCOG04761	Yes	COG01968	UppP
arCOG04762	Yes	COG00451	WcaG
arCOG04779	Yes	COG01446	
arCOG04786	no	COG03396	
arCOG04811	Yes	COG02383	
arCOG04874	no	COG02055	
arCOG04886	Yes		
arCOG04918	no	COG03328	
arCOG04941	Yes	COG03830	
arCOG05042	Yes	SC.00133	
arCOG05121	no	COG02755	TesA
arCOG05127	no		
arCOG05134	Yes		Cas5/Csc1
arCOG05138	Yes	COG00248	GppA
arCOG05211	no	COG04831	
arCOG05252	no	COG04741	
arCOG05276	Yes		
arCOG05278	no	COG05466	
arCOG05303	no	COG04829	
arCOG05316	no	COG02057	AtoA
arCOG05317	no	COG02721	UxaA
arCOG05318	no	COG02721	
arCOG05320	Yes		
arCOG05321	Yes		
arCOG05323	Yes		
arCOG05328	no		
arCOG05331	no	COG02391	
arCOG05332	Yes	COG01716	
arCOG05339	no		
arCOG05341	no	COG00477	ProP
arCOG05342	Yes		
arCOG05344	no	COG00477	ProP
arCOG05348	Yes	COG00446	HcaD
arCOG05356	no	COG02382	Fes
arCOG05360	no		
arCOG05361	no	COG02017	GalM
arCOG05362	no		
arCOG05367	no	COG05592	
arCOG05370	no	COG03386	
arCOG05371	no		

arCOG05372	no	COG01121	ZnuC
arCOG05375	Yes		
arCOG05379	Yes	COG04754	
arCOG05380	Yes	COG02149	
arCOG05388	Yes	COG04344	
arCOG05396	Yes	COG04272	
arCOG05398	Yes	COG00463	
arCOG05400	no		
arCOG05401	Yes		
arCOG05403	Yes	COG02329	OdsN
arCOG05410	no	COG00444	DppD
arCOG05411	no	COG03934	
arCOG05412	no	COG02723	BglB
arCOG05414	no		
arCOG05415	no		
arCOG05416	no		
arCOG05424	Yes		
arCOG05431	no	COG00438	RfaG
arCOG05463	Yes		
arCOG05464	Yes		
arCOG05472	no		
arCOG05489	Yes		
arCOG05495	Yes	COG04911	
arCOG05498	no	COG02225	AceB
arCOG05511	Yes		
arCOG05545	no		
arCOG05546	Yes		
arCOG05628	no	COG00730	
arCOG05632	no	COG02391	
arCOG05633	no		
arCOG05636	no	COG02864	
arCOG05705	Yes	COG03839	MalK
arCOG05706	Yes	COG01506	DAP2
arCOG05707	no		
arCOG05710	Yes		
arCOG05713	Yes		
arCOG05825	Yes	COG01856	
arCOG05850	no	COG02039	Pcp
arCOG05865	no	COG01274	PckA
arCOG05881	no		
arCOG05883	Yes		
arCOG05884	Yes		
arCOG05885	Yes		
arCOG05886	Yes		
arCOG05887	no		
arCOG05888	Yes		
arCOG05889	no		
arCOG05890	Yes		

arCOG05891	Yes		
arCOG05892	Yes		
arCOG05893	no		
arCOG05894	Yes		
arCOG05895	Yes	COG02427	
arCOG05897	Yes		
arCOG05898	Yes		
arCOG05899	Yes		
arCOG05900	Yes	COG03355	
arCOG05901	no		
arCOG05903	Yes		
arCOG05904	Yes		
arCOG05905	Yes		
arCOG05906	no		
arCOG05907	Yes		
arCOG05908	no	COG01633	
arCOG05910	no		
arCOG05911	Yes	COG01826	TatA
arCOG05912	Yes		
arCOG05913	Yes		
arCOG05914	no		
arCOG05915	no		
arCOG05916	Yes		
arCOG05917	Yes	SC.00142	
arCOG05918	Yes		
arCOG05919	Yes	COG00546	Gph
arCOG05920	Yes		
arCOG05921	Yes		
arCOG05922	Yes		
arCOG05923	Yes		
arCOG05924	Yes		
arCOG05925	Yes		
arCOG05926	Yes		
arCOG05927	Yes		
arCOG05928	Yes		
arCOG05929	Yes		
arCOG05930	Yes		
arCOG05931	Yes	COG01405	
arCOG05932	Yes	COG00491	GloB
arCOG05933	Yes		
arCOG05934	Yes		
arCOG05935	Yes		
arCOG05937	Yes	COG00028	IlvB
arCOG05938	Yes		Rpo13
arCOG05939	Yes		
arCOG05941	Yes		
arCOG05942	Yes		
arCOG05943	Yes	COG00463	WcaA

arCOG05944	no		
arCOG05945	no		
arCOG05946	Yes		
arCOG05947	Yes		
arCOG05948	Yes		
arCOG05949	Yes		
arCOG05950	Yes		
arCOG05951	Yes		
arCOG05952	Yes		
arCOG05953	Yes		
arCOG05954	Yes		
arCOG05955	Yes		
arCOG05956	Yes		
arCOG05957	Yes		
arCOG05958	Yes		
arCOG05959	Yes	COG00622	
arCOG05960	Yes		
arCOG05961	Yes	COG01321	TroR
arCOG05962	no		
arCOG05963	no		
arCOG05964	no	COG01960	CaiA
arCOG05965	no	COG01960	CaiA
arCOG05966	no	COG03511	PlcC
arCOG05967	Yes		
arCOG05968	no		
arCOG05969	no	COG03355	
arCOG05970	no		
arCOG05971	Yes	COG00477	
arCOG05972	Yes		
arCOG05973	Yes	COG02226	UbiE
arCOG05974	Yes		
arCOG05975	Yes		
arCOG05976	no	#N/A	#N/A
arCOG05978	Yes		
arCOG05979	Yes		
arCOG05980	Yes		
arCOG05981	Yes		
arCOG05982	no		
arCOG05983	Yes		
arCOG05984	Yes		
arCOG05985	Yes	COG02107	
arCOG05986	Yes		
arCOG05987	Yes		
arCOG05988	Yes		
arCOG05989	Yes		
arCOG05990	Yes	COG00604	Qor
arCOG05991	Yes		
arCOG05992	Yes		

arCOG05993	Yes		
arCOG05994	Yes		
arCOG05995	Yes		
arCOG05996	Yes		
arCOG05997	Yes		
arCOG05998	Yes		
arCOG05999	Yes		
arCOG06000	Yes		
arCOG06001	Yes	COG03889	
arCOG06002	Yes		
arCOG06003	Yes	COG00226	PstS
arCOG06004	Yes	COG03971	
arCOG06005	no	COG03975	
arCOG06006	no		
arCOG06007	Yes		
arCOG06008	Yes		
arCOG06009	no		
arCOG06010	no	COG04280	
arCOG06011	Yes		
arCOG06012	no		
arCOG06013	no		
arCOG06014	no	COG04194	
arCOG06015	Yes		
arCOG06016	Yes		
arCOG06017	Yes		
arCOG06018	no		
arCOG06019	no		Csa3
arCOG06020	no	COG02250	
arCOG06021	no		
arCOG06023	Yes	COG03376	HoxN
arCOG06024	no		Cas5/Cmr3
arCOG06025	Yes		
arCOG06026	Yes	COG01011	
arCOG06027	Yes	COG03189	
arCOG06028	no	COG01487	VapC
arCOG06029	Yes		
arCOG06030	no		
arCOG06031	no	COG05424	
arCOG06032	Yes		
arCOG06033	no		
arCOG06034	Yes		
arCOG06035	Yes		
arCOG06036	no	COG00208	
arCOG06037	no	COG00346	GloA
arCOG06038	Yes		
arCOG06039	Yes		SlaA
arCOG06040	Yes		
arCOG06041	Yes		

arCOG06042	Yes	COG00130	TruB
arCOG06043	no		
arCOG06044	no	COG01802	GntR
arCOG06045	no	COG03589	
arCOG06046	Yes	COG05592	
arCOG06047	no	COG00477	ProP
arCOG06048	Yes		
arCOG06049	no	COG03345	
arCOG06050	Yes	COG03431	
arCOG06051	no	COG01960	CaiA
arCOG06052	no		
arCOG06056	Yes	COG00680	HyaD
arCOG06085	no		
arCOG06088	Yes		
arCOG06096	no		
arCOG06103	no		
arCOG06112	no	COG00365	Acs
arCOG06122	no	COG00365	Acs
arCOG06164	no		
arCOG06188	no	COG02371	UreE
arCOG06207	Yes	COG03526	
arCOG06508	no	COG03039	
arCOG06831	Yes	COG03654	Doc
arCOG06914	no	COG03378	
arCOG06915	no		
arCOG06946	no		
arCOG06947	no		
arCOG06952	no		
arCOG06967	no		
arCOG06975	no		
arCOG07052	no	COG00425	
arCOG07053	no		
arCOG07054	no	COG03193	GlcG
arCOG07088	Yes		
arCOG07173	Yes		
arCOG07174	Yes		
arCOG07176	Yes		
arCOG07177	Yes		
arCOG07178	no		
arCOG07179	no		
arCOG07180	Yes		
arCOG07181	no	COG03889	
arCOG07182	no		
arCOG07183	Yes	COG02244	RfbX
arCOG07184	Yes		
arCOG07185	no		
arCOG07186	no		
arCOG07187	no		

arCOG07188	Yes		
arCOG07189	no		
arCOG07190	no		
arCOG07191	no		
arCOG07192	Yes	COG01695	
arCOG07194	Yes		
arCOG07195	Yes		
arCOG07196	no		
arCOG07197	Yes		
arCOG07198	Yes		
arCOG07200	no		
arCOG07202	no	COG01988	
arCOG07204	no	COG02267	PldB
arCOG07205	no		
arCOG07206	no		
arCOG07209	no		
arCOG07210	Yes		
arCOG07211	no		
arCOG07213	Yes		
arCOG07214	Yes		
arCOG07215	Yes		
arCOG07216	Yes		
arCOG07217	Yes		
arCOG07218	no		
arCOG07219	Yes		
arCOG07220	Yes		
arCOG07221	no		
arCOG07222	no		
arCOG07224	no		
arCOG07225	Yes		
arCOG07227	no		
arCOG07228	no	COG02608	
arCOG07229	no		
arCOG07230	no		
arCOG07231	no		
arCOG07232	no		
arCOG07233	no	COG03327	PaaX
arCOG07234	no		
arCOG07235	no	SC.00003	
arCOG07237	Yes		
arCOG07238	no		
arCOG07239	no		
arCOG07240	no	COG00463	WcaA
arCOG07243	no		
arCOG07244	no		
arCOG07246	no		
arCOG07247	no		
arCOG07248	no		

arCOG07249	no		
arCOG07250	no		
arCOG07251	no		
arCOG07252	no		
arCOG07253	no		
arCOG07254	Yes	COG05607	
arCOG07255	no		
arCOG07256	no		
arCOG07257	no		
arCOG07258	no		
arCOG07259	no		
arCOG07260	no	COG03191	DmpA
arCOG07262	Yes		
arCOG07263	no	COG01735	Php
arCOG07264	no		
arCOG07265	no		
arCOG07267	no	COG03733	TynA
arCOG07268	no		
arCOG07269	Yes		
arCOG07271	Yes		
arCOG07272	Yes		SlaB
arCOG07273	Yes		
arCOG07274	Yes		
arCOG07275	no		
arCOG07276	Yes		
arCOG07277	no		
arCOG07278	Yes		
arCOG07279	no		
arCOG07280	no		
arCOG07281	no	COG00457	NrfG
arCOG07282	no		
arCOG07283	Yes		
arCOG07284	Yes		
arCOG07285	Yes	COG02811	NtpF
arCOG07286	Yes		
arCOG07287	Yes		
arCOG07288	no		
arCOG07289	no		
arCOG07290	no		
arCOG07291	no		
arCOG07292	Yes	COG01708	
arCOG07293	no	COG01192	Soj
arCOG07294	no	COG02308	
arCOG07295	no	COG02307	
arCOG07296	Yes		
arCOG07297	no		
arCOG07298	no		
arCOG07299	no		

arCOG07300	no		
arCOG07301	no		
arCOG07303	no	COG00675	
arCOG07304	no		
arCOG07305	Yes		
arCOG07307	no		
arCOG07308	no		
arCOG07309	Yes		PlrA
arCOG07310	no		
arCOG07311	no		
arCOG07312	no	SC.00240	
arCOG07313	no		
arCOG07314	Yes		
arCOG07315	no	COG01502	
arCOG07316	no		
arCOG07317	no		
arCOG07318	no		
arCOG07319	no		
arCOG07320	Yes	COG00247	GlpC
arCOG07321	Yes		
arCOG07323	Yes		
arCOG07324	no		
arCOG07325	no		
arCOG07326	no		
arCOG07327	no		
arCOG07329	Yes		
arCOG07330	no		
arCOG07331	no	COG02433	
arCOG07332	no		
arCOG07333	no		
arCOG07334	no		
arCOG07335	Yes	COG00500	
arCOG07337	no	COG03250	LacZ
arCOG07338	no	COG03669	
arCOG07339	no		
arCOG07340	no		
arCOG07341	no		
arCOG07342	no		
arCOG07343	no		
arCOG07347	no		
arCOG07349	no	COG01670	RimL
arCOG07351	no		
arCOG07450	no		
arCOG07461	no	SC.00158	
arCOG07493	no		
arCOG07496	no	COG03451	VirB4
arCOG07501	no	COG01994	SpoIVFB
arCOG07520	no	COG02442	

arCOG07523	no		
arCOG07527	no		
arCOG07589	Yes	COG01848	
arCOG07714	no		
arCOG07715	no		
arCOG07716	Yes		
arCOG07717	no		
arCOG07718	Yes		
arCOG07719	no	COG00535	
arCOG07720	no		
arCOG07721	no		
arCOG07722	no		
arCOG07723	no		
arCOG07724	no		
arCOG07726	no		
arCOG07727	no		
arCOG07728	no	COG01879	RbsB
arCOG07730	no		
arCOG07731	Yes		
arCOG07732	no	COG05558	
arCOG07733	no	SC.00162	
arCOG07734	no		
arCOG07735	no		
arCOG07736	no		
arCOG07775	no	COG04638	HcaE
arCOG07844	no		
arCOG07846	no	COG01604	Cas7/Cmr6
arCOG07847	Yes		
arCOG07848	no		
arCOG07849	Yes		
arCOG07850	no		
arCOG07851	no		
arCOG07852	Yes		
arCOG07853	no		
arCOG07922	no	SC.00238	
arCOG07925	no	SC.00164	
arCOG07929	no		
arCOG07930	no		
arCOG07934	Yes	SC.00165	
arCOG07935	no	COG01475	Spo0J
arCOG07936	Yes	COG04742	
arCOG07940	no		
arCOG07957	no	COG02342	
arCOG07960	no	COG02607	
arCOG07990	Yes		
arCOG08073	no		
arCOG08078	Yes		
arCOG08086	no	COG01848	

arCOG08092	no	COG02259	
arCOG08093	Yes		
arCOG08212	Yes		
arCOG08216	Yes		
arCOG08304	no		
arCOG08305	Yes		
arCOG08306	no	COG01497	
arCOG08307	no		
arCOG08308	Yes		
arCOG08309	Yes		
arCOG08310	no		
arCOG08311	no		
arCOG08312	no		
arCOG08313	no		
arCOG08314	no		
arCOG08315	Yes		
arCOG08316	no		
arCOG08317	no		
arCOG08318	no		
arCOG08319	no		
arCOG08320	Yes		
arCOG08321	no		
arCOG08322	Yes		
arCOG08323	no		
arCOG08324	Yes		
arCOG08326	no		
arCOG08328	no		
arCOG08329	no		
arCOG08330	no		
arCOG08331	no		
arCOG08332	Yes		
arCOG08333	Yes		
arCOG08334	Yes		
arCOG08336	no	COG00425	SirA
arCOG08337	Yes		
arCOG08338	no		
arCOG08340	no		
arCOG08341	Yes		
arCOG08342	Yes		
arCOG08343	Yes		
arCOG08344	no		
arCOG08345	no		
arCOG08346	no		
arCOG08347	no		
arCOG08348	no		
arCOG08349	no		
arCOG08357	no	SC.00156	
arCOG08361	no	COG04228	TmoE

arCOG08362	no		TmoD
arCOG08363	no		
arCOG08364	no	COG04228	TmoA
arCOG08372	Yes		
arCOG08375	no		
arCOG08377	no		
arCOG08378	no		
arCOG08380	Yes		
arCOG08395	no		
arCOG08400	no		
arCOG08401	no		
arCOG08402	no		
arCOG08405	no		
arCOG08406	no		
arCOG08408	no		
arCOG08412	no		
arCOG08424	no		
arCOG08426	no		
arCOG08436	no		
arCOG08437	no		
arCOG08438	no	COG05517	
arCOG08442	no		
arCOG08446	no		
arCOG08449	no		
arCOG08451	no		
arCOG08457	Yes		
arCOG08458	Yes		
arCOG08460	no	#N/A	#N/A
arCOG08462	Yes	SC.00185	
arCOG08463	no	COG02433	
arCOG08464	no		
arCOG08465	no		
arCOG08466	no		
arCOG08472	no		
arCOG08473	no		
arCOG08475	Yes		
arCOG08476	Yes		
arCOG08477	no		
arCOG08479	Yes		
arCOG08481	no	SC.00133	
arCOG08485	no		
arCOG08486	no		
arCOG08497	no		
arCOG08498	no		
arCOG08510	no		
arCOG08511	no		
arCOG08520	no		
arCOG08525	no		

arCOG08526	no		
arCOG08530	no		
arCOG08538	Yes		
arCOG08539	no		
arCOG08540	no		
arCOG08543	no		
arCOG08552	no		
arCOG08554	no		
arCOG08555	no		
arCOG08557	no		
arCOG08558	no		
arCOG08560	Yes		
arCOG08562	no		
arCOG08563	no		
arCOG08569	no		
arCOG08572	no		
arCOG08574	no		
arCOG08576	no		
arCOG08583	no		
arCOG08584	no		
arCOG08585	no		
arCOG08587	no		
arCOG08588	no		
arCOG08805	no	COG05304	
arCOG08842	Yes	COG02188	PhnF
arCOG09692	no		
arCOG09693	no		
arCOG09694	no		
arCOG09695	no		
arCOG09696	no		
arCOG09697	no		
arCOG09698	no		
arCOG09699	no		
arCOG09701	no		
arCOG09703	no		
arCOG09704	no		
arCOG09705	no		
arCOG09706	no		
arCOG09707	no		
arCOG09709	no		
arCOG09710	no		
arCOG09711	no		
arCOG09712	no		
arCOG09713	no	COG01846	
arCOG09855	no		
arCOG09856	no		
arCOG09862	no		
arCOG09866	no	SC.00162	

arCOG09871	Yes	
arCOG09879	no	
arCOG09882	no	COG02192
arCOG09883	no	
arCOG09884	no	
arCOG09893	no	
arCOG09902	no	
arCOG09905	no	
arCOG09923	no	
arCOG09924	no	
arCOG09928	no	
arCOG09929	Yes	
arCOG09933	no	
arCOG09934	no	
arCOG09948	no	
arCOG09962	no	
arCOG09968	no	
arCOG09969	no	
arCOG09970	Yes	
arCOG09989	no	
arCOG09991	no	SC.00185
arCOG09993	Yes	
arCOG09995	no	
arCOG10002	Yes	SC.00165
arCOG10005	no	
arCOG10007	no	COG00829
arCOG10013	no	
arCOG10014	no	
arCOG10015	no	COG04278
arCOG10016	no	
arCOG10022	no	
arCOG10132	Yes	
arCOG10174	no	
arCOG10215	no	
arCOG10265	Yes	
arCOG10266	no	
arCOG10299	no	
arCOG10433	no	
arCOG10434	no	
arCOG10507	no	
arCOG10508	no	
arCOG10885	no	
arCOG11006	no	
arCOG11038	no	
arCOG11043	no	
arCOG11294	no	
arCOG11295	no	
arCOG11298	no	

arCOG11361	no	SC.00155	
arCOG00143	Yes	COG00477	ProP
arCOG00462	Yes	COG01194	MutY
arCOG00552	Yes	COG01711	
arCOG00834	Yes	COG00454	WecD
arCOG00934	Yes	COG01313	PflX
arCOG00940	Yes	COG00535	
arCOG00968	Yes	COG02428	
arCOG01187	Yes	COG00068	HypF
arCOG01242	Yes	COG00582	XerC
arCOG01373	Yes	COG01089	Gmd
arCOG01423	Yes	COG05493	
arCOG01550	Yes	COG00374	HyaB
arCOG01955	Yes	COG00475	KefB
arCOG02079	Yes	COG01361	
arCOG02474	Yes	COG01740	HyaA
arCOG02632	Yes	COG00286	HsdM
arCOG02782	Yes	COG01787	
arCOG03664	Yes	COG01215	
arCOG03812	Yes	COG03463	
arCOG04400	Yes	COG01361	
arCOG04427	Yes	COG00298	HypC
arCOG04428	Yes	COG00409	HypD
arCOG05099	Yes	COG02105	
arCOG05482	Yes	COG00467	RAD55
arCOG06057	Yes		
arCOG06072	Yes		
arCOG06076	Yes		
arCOG06089	Yes		
arCOG06513	Yes	COG05485	
arCOG07199	Yes		
arCOG07226	Yes		
arCOG07245	Yes		
arCOG07348	Yes		
arCOG07416	Yes	COG00596	MhpC
arCOG07536	Yes	COG03055	
arCOG09897	Yes		
arCOG10353	Yes		
arCOG10437	Yes		
arCOG11297	Yes		

Annotation
Predicted transcriptional regulator, PadR family
Predicted transcriptional regulator, PadR family
Amino acid transporter
Sugar kinase, ribokinase family
Sugar kinase, ribokinase family
Predicted transcriptional regulator
Predicted sugar kinase
Hydroxymethylpyrimidine/phosphomethylpyrimidine kinase
Predicted transcriptional regulator fused phosphomethylpyrimidine kinase, involved in the thiamin biosynthesis
Uncharacterized conserved protein
Glycerol kinase
Sugar (pentulose and hexulose) kinase
Orotate phosphoribosyltransferase
Adenine/guanine phosphoribosyltransferase or related PRPP-binding protein
Wybutosine (yW) biosynthesis enzyme, Trm5 methyltransferase
Predicted glutamine amidotransferase involved in pyridoxine biosynthesis
Predicted ATPase of PP-loop superfamily
tRNA S(4)U 4-thiouridine synthase
Predicted PP-loop superfamily ATPase
Predicted phosphoribosyltransferase
tRNA(Ile)-lysidine synthase MesJ
Predicted DNA modification methylase
Spermidine synthase
Glucose-6-phosphate isomerase
3-hexulose-6-phosphate synthase or related protein
Glucosamine 6-phosphate synthetase
Predicted phosphosugar isomerase
Cystathionine beta-lyase/cystathionine gamma-synthase
Predicted amidohydrolase
CTP synthase (UTP-ammonia lyase)
Carbamoylphosphate synthase small subunit
Selenocysteine lyase/Cysteine desulfurase
Phosphoribosylpyrophosphate synthetase
Predicted sugar phosphate isomerase involved in capsule formation
NH3-dependent NAD+ synthetase
Glycine/serine hydroxymethyltransferase
Glycine cleavage system protein P (pyridoxal-binding), C-terminal domain
Glycine cleavage system protein P (pyridoxal-binding), N-terminal domain
Fibrillarin-like rRNA methylase
Orotidine-5'-phosphate decarboxylase
Serine-pyruvate aminotransferase/archaeal aspartate aminotransferase
Predicted RNA-binding protein, contains THUMP domain
GMP synthase, PP-ATPase domain/subunit
Anthranilate/para-aminobenzoate synthase component II
GMP synthase - Glutamine amidotransferase domain
Glutamine amidotransferase

GMP synthase - Glutamine amidotransferase domain
Glutamine phosphoribosylpyrophosphate amidotransferase
Glutamine phosphoribosylpyrophosphate amidotransferase
Glutamate synthase domain 1
Glutamate synthase domain 3
Phosphoribosylformylglycinamidine (FGAM) synthase, glutamine amidotransferase domain
Cobyric acid synthase
Cobyrinic acid a,c-diamide synthase
Methylase of polypeptide chain release factors
16S RNA G1207 methylase RsmC
Argininosuccinate synthase
DNA modification methylase
3'-phosphoadenosine 5'-phosphosulfate sulfotransferase (PAPS reductase)/FAD synthetase or related enzyme
Permease of the major facilitator superfamily
Permease of the major facilitator superfamily
Permease of the major facilitator superfamily
Permease of the major facilitator superfamily
Permease of the major facilitator superfamily
Permease of the major facilitator superfamily
Permease of the major facilitator superfamily
Permease of the major facilitator superfamily
ABC-type sugar transport system, periplasmic component
ABC-type trehalose transport system, periplasmic component
Maltose-binding periplasmic protein
ABC-type sugar transport system, periplasmic component
ABC-type sugar transport system, permease component
ABC-type sugar transport system, permease component
ABC-type sugar transport system, permease component
ABC-type maltose transport system, permease component
ABC-type sulfate transport system, permease component
ABC-type phosphate transport system, permease component
ABC-type phosphate transport system, permease component
ABC-type anion transport system, duplicated permease component
ABC-type sugar transport system, ATPase component
ABC-type spermidine/putrescine transport system, ATPase component
ABC-type dipeptide/oligopeptide/nickel transport system, ATPase component
ABC-type dipeptide/oligopeptide/nickel transport system, ATPase component
ABC-type oligopeptide transport system, ATPase component
ATPase component of various ABC-type transport system, contain duplicated ATPase
Predicted ATPase, RNase L inhibitor (RLI) homolog
ABC-type cobalt transport system, ATPase component
ABC-type nitrate/sulfonate/bicarbonate transport system, ATPase component
ABC-type multidrug transport system, ATPase component
ABC-type multidrug transport system, ATPase component
ABC-type cobalamin/Fe3+-siderophores transport system, ATPase component
ABC-type oligopeptide transport system, ATPase component
ABC-type multidrug transport system, ATPase component
ABC-type phosphate transport system, periplasmic component

Molybdopterin biosynthesis enzyme
Predicted nucleotide-utilizing enzyme related to molybdopterin-biosynthesis enzyme MoeA
Molybdopterin biosynthesis enzyme
Molybdopterin biosynthesis enzyme
ABC-type molybdate transport system, periplasmic component
N-terminal domain of molybdenum-binding protein
ABC-type phosphate transport system, ATPase component
2-keto-4-pentenoate hydratase/2-oxohepta-3-ene-1,7-dioic acid hydratase (catechol pathway)
Fumarylacetoacetate (FAA) hydrolase family protein
Na+/H+ antiporter NhaD or related arsenite permease
Enoyl-CoA hydratase/carnithine racemase
Enoyl-CoA hydratase/carnithine racemase
Prephenate dehydrogenase
Malate/lactate dehydrogenase
3-hydroxyisobutyrate dehydrogenase or related beta-hydroxyacid dehydrogenase
3-hydroxyacyl-CoA dehydrogenase
3-hydroxyacyl-CoA dehydrogenase
Predicted UDP-glucose 6-dehydrogenase
Prephenate dehydratase
Ribose/xylose/arabinose/galactoside ABC-type transport system, permease component
Sulfite oxidase or related enzyme
Sulfite oxidase or related enzyme
Permease of the drug/metabolite transporter (DMT) superfamily
S-adenosylmethionine decarboxylase/arginine decarboxylase
HerA helicase
HerA helicase
HerA helicase
HerA helicase
HerA helicase
Predicted NurA-like nuclease
Nitroreductase
Metal-dependent phosphoesterase (PHP family)
RNase P/RNase MRP subunit p30
Peroxiredoxin
Peroxiredoxin
Uncharacterized conserved protein
Phosphate uptake regulator
Phosphate uptake regulator
Predicted Zn-dependent protease or their inactivated homolog
Predicted Zn-dependent protease or their inactivated homolog
Predicted Zn-dependent protease or their inactivated homolog
Deacetylase, including yeast histone deacetylase and acetoin utilization protein
Deacetylase, including yeast histone deacetylase and acetoin utilization protein
DNA polymerase elongation subunit (family B)
DNA polymerase elongation subunit (family B) , inactivated
Membrane associated Fe-S oxidoreductase
Fe-S oxidoreductase
Uncharacterized conserved protein containing ferredoxin-like domain

FAD/FMN-containing dehydrogenase
Succinate dehydrogenase subunit C
FAD/FMN-containing dehydrogenase fused to Heterodisulfide reductase, subunit B
FAD/FMN-containing dehydrogenase
Heterodisulfide reductase, subunit B
Fe-S oxidoreductase
FAD/FMN-containing dehydrogenase
FAD/FMN-containing dehydrogenase
Archaeal enzyme of ATP-grasp superfamily
Predicted GTPase
Predicted GTPase
GTP-binding protein protease modulator
Predicted GTPase, probable translation factor
Predicted GTPase
NurA 5'-3' nuclease
ATPase involved in DNA repair, SbcC
DNA sulfur modification protein DndD, ATPase
Predicted membrane-associated trancriptional regulator
Uncharacterized conserved protein
Predicted membrane-associated trancriptional regulator
Transcriptional regulator containing HTH domain, ArsR family
DNA repair exonuclease, SbcD
Threonyl-tRNA synthetase
Prolyl-tRNA synthetase
Seryl-tRNA synthetase
Histidyl-tRNA synthetase
Glycyl-tRNA synthetase (class II)
Aspartyl/asparaginyl-tRNA synthetase
Lysyl-tRNA synthetase (class II)
Phenylalanyl-tRNA synthetase alpha subunit
Phenylalanyl-tRNA synthetase beta subunit
RecA/RadA recombinase
RecA/RadA recombinase
HIT family hydrolase
Predicted phosphohydrolase (DHH superfamily)
Single-stranded DNA-specific exonuclease RecJ
MoxR-like ATPase
MoxR-like ATPase
Predicted ATPase involved in replication control, Cdc46/Mcm family
Uncharacterized protein containing a von Willebrand factor type A (vWA) domain
Electron transfer flavoprotein, beta subunit
Electron transfer flavoprotein, alpha subunit
Electron transfer flavoprotein, alpha and beta subunits
Crenarchaeal division protein ESCRT-III
Crenarchaeal division protein ESCRT-III
Crenarchaeal division protein ESCRT-III
Pyrroline-5-carboxylate reductase
Predicted Zn-dependent protease

EndoIII-related endonuclease
Uncharacterized protein related to Endonuclease III
Uri superfamily endonuclease
Cdc6-related protein, AAA superfamily ATPase
ATPase involved in DNA replication HolB, small subunit
ATPase involved in DNA replication HolB, large subunit
5-formyltetrahydrofolate cyclo-ligase
5,10-methylenetetrahydrofolate reductase
4-hydroxybenzoate polyprenyltransferase or related prenyltransferase
4-hydroxybenzoate polyprenyltransferase or related prenyltransferase
Polyprenyltransferase (cytochrome oxidase assembly factor)
Cysteinyl-tRNA synthetase
Arginyl-tRNA synthetase
DNA polymerase sliding clamp subunit (PCNA homolog)
cob(I)alamin adenosyltransferase
Transcriptional regulators containing a DNA-binding HTH domain and an aminotransferase domain (MocR family)
Glyceraldehyde-3-phosphate dehydrogenase/erythrose-4-phosphate dehydrogenase
Aspartate-semialdehyde dehydrogenase
Acetylglutamate semialdehyde dehydrogenase
3-phosphoglycerate kinase
Zn-dependent hydrolase of the beta-lactamase fold
Metal-dependent hydrolase of the beta-lactamase superfamily II
Metal-dependent hydrolase of the beta-lactamase superfamily
Metal-dependent hydrolase of the beta-lactamase superfamily
Metal-dependent hydrolase of the beta-lactamase superfamily II
Metal-dependent hydrolase of the beta-lactamase superfamily II
Metal-dependent hydrolase of the beta-lactamase superfamily II
Uncharacterized flavoprotein
Zn-dependent hydrolase, glyoxylase family
Multimeric flavodoxin WrbA
Metal-dependent hydrolase of the beta-lactamase superfamily II
Rhodanese Homology Domain fused to Zn-dependent hydrolase of glyoxylase family
Molybdopterin-guanine dinucleotide biosynthesis protein
Molybdopterin converting factor, large subunit
Sulfur transfer protein involved in thiamine biosynthesis
Molybdopterin converting factor, small subunit
Ubiquitin-like protein
Predicted exonuclease of the beta-lactamase fold involved in RNA processing
Predicted metal-dependent RNase, consists of a metallo-beta-lactamase domain and an RNA-binding KH domain
Predicted exonuclease of the beta-lactamase fold involved in RNA processing
DNA replication initiation complex subunit, GINS15 family
Replicative SFII helicase
Distinct helicase family with a unique C-terminal domain including a metal-binding cysteine cluster
Lhr-like helicase
Superfamily II DNA and RNA helicase
Predicted membrane-bound dolichyl-phosphate-mannose-protein mannosyltransferase
Uncharacterized conserved protein
Dehydrogenase (flavoprotein)

Succinate dehydrogenase/fumarate reductase, flavoprotein subunit
Aspartate oxidase
Ribulose 1,5-bisphosphate synthetase, converts PRPP to RuBP, flavoprotein
Predicted divalent heavy-metal cations transporter
Uncharacterized Zn-finger containing protein
DNA-directed RNA polymerase, subunit M/Transcription elongation factor TFIIS
DNA-directed RNA polymerase, subunit M/Transcription elongation factor TFIIS
PEP phosphonomutase or related enzyme
Isocitrate lyase
Ketopantoate hydroxymethyltransferase
Mrp family protein, ATPase, contains iron-sulfur cluster
ATPase involved in chromosome partitioning, ParA family
CobQ/CobB/MinD/ParA ATPase
CBS domain
Protein containing two CBS domains (some fused to C-terminal double-stranded RNA-binding domain of RaiA fam
Dihydroorotate dehydrogenase
CBS domain
Predicted transcriptional regulator with C-terminal CBS domains
Predicted transcriptional regulator, contains C-terminal CBS domains
Isopentenyl diphosphate isomerase
Zn-dependent protease
NADH:flavin oxidoreductase, Old Yellow Enzyme family
Imidazoleglycerol-phosphate synthase
Phosphoribosylformimino-5-aminoimidazole carboxamide ribonucleotide (ProFAR) isomerase
Glutamate synthase domain 2 and ferredoxin domain
Mg/Co/Ni transporter MgtE (contains CBS domain)
CBS domain
Hydrogenase maturation factor
Hydrogenase maturation factor
Thiamine monophosphate kinase
Phosphoribosylaminoimidazole (AIR) synthetase
Phosphoribosylformylglycinamidine (FGAM) synthase, synthetase domain
Selenophosphate synthase
Uroporphyrinogen-III methylase
Precorrin-4 methylase
Uroporphyrinogen-III methylase
Precorrin-3B methylase
Precorrin-2 methylase
Precorrin-6B methylase 1
Cobalamin biosynthesis protein CbiG
Cobalamin biosynthesis protein CbiG
Predicted periplasmic solute-binding protein
Lipoate synthase
Biotin synthase-related enzyme
Nucleoside-diphosphate-sugar pyrophosphorylase involved in lipopolysaccharide biosynthesis/translation initiatio
dTDP-glucose pyrophosphorylase
UDP-glucose pyrophosphorylase
N-acetylglucosamine-1-phosphate uridyltransferase

dTDP-glucose pyrophosphorylase
Phosphatidylglycerophosphate synthase
DNA-directed RNA polymerase, subunit E'
Predicted RNA-binding protein (consists of S1 domain and a Zn-ribbon domain)
RNA-binding protein Rrp4 or related protein (contain S1 domain and KH domain)
Transposase
Transposase
Transposase
Transposase
Dihydroorotase or related cyclic amidohydrolase
Cytosine deaminase or related metal-dependent hydrolase
Cytosine deaminase or related metal-dependent hydrolase
Imidazolonepropionase or related amidohydrolase
Cytosine deaminase or related metal-dependent hydrolase
Urea amidohydrolase (urease) alpha subunit
N-acetylglucosamine-6-phosphate deacetylase
Glycerophosphoryl diester phosphodiesterase
PIN domain containing protein
PIN domain containing protein
PIN domain containing protein
Endonuclease Nob1, consists of a PIN domain and a Zn-ribbon module
Uncharacterized conserved protein
Predicted transcriptional regulator, PadR family
PIN domain containing protein
PIN domain containing protein
PIN domain containing protein
Transcriptional regulator containing HTH domain, ArsR family
Transcriptional regulator, MarR family
Transcriptional regulator containing HTH domain
ABC-type dipeptide/oligopeptide/nickel transport system, permease component
ABC-type dipeptide/oligopeptide/nickel transport system, permease component
ABC-type dipeptide/oligopeptide/nickel transport system, permease component
Glycerol-3-phosphate dehydrogenase
Glycine/D-amino acid oxidase (deaminating)
Glycine cleavage system T protein (aminomethyltransferase)
Glycine/D-amino acid oxidase (deaminating)
Glycine/D-amino acid oxidase (deaminating)
Beta-methylmalyl-CoA lyase (Citrate lyase beta subunit family)
Uncharacterized conserved protein
Phosphomannomutase
Putative intracellular protease/amidase
Rad3-related DNA helicase
Acyl-CoA hydrolase
Mesaconyl-CoA hydratase (Acyl dehydratase family)
HGG motif-containing thioesterase, possibly involved in aromatic compounds catabolism
Ribosomal protein L15
Ribosomal protein L18E
Ribosomal protein L32E

Ribosomal protein S14
RNase P/RNase MRP subunit p29
Ribosomal protein L29
CRISPR-associated protein Cas4, RecB family exonuclease
CRISPR-associated protein Cas4, RecB family exonuclease
Superfamily I DNA/RNA helicase
Isoleucyl-tRNA synthetase
Valyl-tRNA synthetase
Leucyl-tRNA synthetase
Methionyl-tRNA synthetase
Transcriptional regulator AbrB
Transcriptional regulator AbrB
Transcriptional regulator AbrB
Transcriptional regulator AbrB
Transcriptional regulator AbrB
Transcriptional regulator AbrB
Transcriptional regulator AbrB
Transcriptional regulator AbrB
Transcriptional regulator AbrB
Acetyltransferase (GNAT) family
Acetyltransferase (GNAT) family
Acetyltransferase (GNAT) family
Malic enzyme
Acyl-CoA synthetase (AMP-forming)/AMP-acid ligase II
Acyl-CoA synthetase (AMP-forming)/AMP-acid ligase II
Uridylate kinase
Isopentenyl phosphate kinase, enzyme of modified mevalonate pathway
Aspartokinase
Acetylglutamate kinase
Archaeal/vacuolar-type H+-ATPase subunit B
Archaeal/vacuolar-type H+-ATPase subunit A
Archaeal/vacuolar-type H+-ATPase subunit E
Superfamily II DNA/RNA helicase, SNF2 family
ssDNA-dependent ATPase, helicase superfamily II
DNA or RNA helicase of superfamily II
Superfamily II DNA/RNA helicase, SNF2 family
Predicted ATPase (AAA+ superfamily)
Adenine-specific DNA methylase containing a Zn-ribbon
DNA modification methylase
Mg-dependent DNase
Glycosyltransferase
Glycosyltransferase
Uracil-DNA glycosylase
Predicted Zn-ribbon RNA-binding protein
Predicted RNA methylase
Aspartate carbamoyltransferase, catalytic chain
Ornithine carbamoyltransferase
Ornithine/acetylornithine aminotransferase

4-aminobutyrate aminotransferase or related aminotransferase
4-aminobutyrate aminotransferase or related aminotransferase
Glutamate-1-semialdehyde aminotransferase
Holliday junction resolvase
ABC-type antimicrobial peptide transport system, ATPase component
ABC-type branched-chain amino acid transport system, ATPase component
ABC-type branched-chain amino acid transport system, ATPase component
Endonuclease V homolog
Deoxyinosine 3'endonuclease (endonuclease V)
Molybdenum cofactor biosynthesis enzyme
Radical SAM superfamily enzyme
Uncharacterized conserved protein related to pyruvate formate-lyase activating enzyme
Radical SAM superfamily enzyme
Radical SAM superfamily enzyme
Radical SAM superfamily enzyme
Pyruvate-formate lyase-activating enzyme
Ferredoxin
Succinate dehydrogenase/fumarate reductase, Fe-S protein subunit
Heterodisulfide reductase, subunit C
Heterodisulfide reductase, subunit C
tRNA m(1)G methyltransferase
Predicted hydrolase (metallo-beta-lactamase superfamily)
20S proteasome, beta subunit
20S proteasome, alpha subunit
Nicotinamide mononucleotide adenylyltransferase
tRNA or rRNA cytosine-C5-methylase
tRNA or rRNA cytosine-C5-methylase
Protein-L-isoaspartate carboxylmethyltransferase
Precorrin-6B methylase 2
tRNA(1-methyladenosine) methyltransferase
FKBP-type peptidyl-prolyl cis-trans isomerase 2
Glycerol dehydrogenase or related enzyme
3-dehydroquinate synthetase
Alcohol dehydrogenase, class IV
Predicted RNA-binding protein (contains PUA domain)
RNA methylase associated PUA domain
Pseudouridine synthase
Queuine/archaeosine tRNA-ribosyltransferase
tRNA modification protein, contains pre-PUA and PUA domains
Protein involved in ribosomal biogenesis, contains PUA domain
Queuine tRNA-ribosyltransferase related protein
Queuine/archaeosine tRNA-ribosyltransferase
Small nuclear ribonucleoprotein (snRNP) homolog
Small nuclear ribonucleoprotein (snRNP) homolog
Xaa-Pro aminopeptidase
Methionine aminopeptidase
Predicted acetamidase/formamidase
ABC-type Fe3+-siderophore transport system, permease component

Transcriptional regulator, CopG/Arc/MetJ family (DNA-binding and a metal-binding domains)
Ribbon-helix-helix protein, copG family
Antitoxin (DNA-binding domain) of toxin-antitoxin stability system
Ribosomal protein L13E
Predicted pseudouridylate synthase
DNA-directed RNA polymerase, subunit F (rpoF)
rRNA methylase
SpoU rRNA Methylase family enzyme
ABC-type branched-chain amino acid transport system, periplasmic component
Archaeal shikimate kinase
Predicted sugar kinase
Homoserine kinase
Mevalonate kinase
Phosphomevalonate kinase
Shikimate 5-dehydrogenase
Predicted nucleotide kinase
Alanine dehydrogenase, mu-crystallin homolog
Glutamyl-tRNA reductase
Cytidylate kinase
Predicted nucleotide kinase (CMP/AMP kinase related)
Archaeal adenylate kinase
Exosome subunit, RNA binding protein with dsRBD fold
Predicted RNA binding protein with dsRBD fold
Siroheme synthase (precorrin-2 oxidase/ferrochelatase domain)
Dephospho-CoA kinase
3-deoxy-D-arabino-heptulosonate 7-phosphate (DAHP) synthase
Transketolase, C-terminal subunit
Pyruvate/2-oxoglutarate/acetoin dehydrogenase complex, dehydrogenase (E1) component
Transketolase, N-terminal subunit
Pyruvate/2-oxoglutarate/acetoin dehydrogenase complex, dehydrogenase (E1) component, eukaryotic type, alph[illegible]
Predicted transcriptional regulator
Predicted transcriptional regulator
NAD(FAD)-dependent dehydrogenase
NAD(FAD)-dependent dehydrogenase
NADH dehydrogenase, FAD-containing subunit
Pyruvate/2-oxoglutarate dehydrogenase complex, dihydrolipoamide dehydrogenase (E3) component or related e[illegible]
CoA-dependent NAD(P)H Sulfur Oxidoreductase
NAD(FAD)-dependent dehydrogenase
NUDIX family hydrolase
NUDIX family hydrolase
NUDIX family hydrolase
NUDIX family hydrolase
Predicted pyrophosphatase
Predicted phosphate-binding enzyme, TIM-barrel fold
Tryptophan synthase alpha chain
Triosephosphate isomerase
Indole-3-glycerol phosphate synthase
Uncharacterized membrane protein

Protein distantly related to bacterial ferritins
Ferritin-like domain containing protein
Rubrerythrin
Uncharacterized conserved protein
Uncharacterized conserved protein
Acetylornithine deacetylase/Succinyl-diaminopimelate desuccinylase or related deacylase
Metal-dependent amidase/aminoacylase/carboxypeptidase
Acetylornithine deacetylase/Succinyl-diaminopimelate desuccinylase or related deacylase
Acetylornithine deacetylase/Succinyl-diaminopimelate desuccinylase or related deacylase
Phosphoenolpyruvate synthase/pyruvate phosphate dikinase
Phosphoenolpyruvate synthase/pyruvate phosphate dikinase
tRNA(Ile2) 2-agmatinylcytidine synthetase; containing Zn-ribbon domain and OB-fold domain
Lrp/AsnC family C-terminal domain
GYD domain, alpha/beta barrel superfamily
SWIM zinc finger
Ribose 5-phosphate isomerase
Translation initiation factor eIF-2B alpha subunit
Transcriptional activator TenA
Transcriptional activator TenA
Aspartate/tyrosine/aromatic aminotransferase
EMAP domain RNA-binding protein
Thioesterase superfamily enzyme
Predicted metal-dependent protease of the PAD1/JAB1 superfamily
Phosphoesterase
Serine/threonine protein phosphatase PP2A family
Icc family phosphoesterase
Metallophosphoesterase superfamily enzyme
Calcineurin-like phosphoesterase
Metallophosphoesterase superfamily enzyme
Calcineurin-like phosphoesterase superfamily protein
Uncharacterized archaeal coiled-coil protein
Isocitrate/isopropylmalate dehydrogenase
Isocitrate dehydrogenase
DNA topoisomerase VI, subunit B
Aerobic-type carbon monoxide dehydrogenase, large subunit CoxL/CutL homolog
L-alanine-DL-glutamate epimerase or related enzyme of enolase superfamily
Enolase
RecA-superfamily ATPase implicated in signal transduction
Translation initiation factor 1 (IF-1)
Serine/threonine protein kinase involved in cell cycle control
RIO-like serine/threonine protein kinase fused to N-terminal HTH domain
Predicted Ser/Thr protein kinase
Subunit of KEOPS complex, contains a domain with ASKHA fold and RIO-type kinase (AP-endonuclease activity)
Mn2+-dependent serine/threonine protein kinase
Mn2+-dependent serine/threonine protein kinase
Predicted unusual protein kinase
HEPN domain containing protein
HEPN domain containing protein

Minimal nucleotidyltransferase
Minimal nucleotidyltransferase
Minimal nucleotidyltransferase
Minimal nucleotidyltransferase
Minimal nucleotidyltransferase
Minimal nucleotidyltransferase
Minimal nucleotidyltransferase
Minimal nucleotidyltransferase
Uncharacterized conserved protein
HAD superfamily hydrolase
Signal recognition particle 19 kDa protein
Thiol-disulfide isomerase or thioredoxin
N2,N2-dimethylguanosine tRNA methyltransferase
Inosine/xanthosine triphosphatase
Cytidylyltransferase fused to conserved domain of DUF357 family
Uncharacterized conserved protein
GTPase SAR1 or related small G protein
Putative periplasmic protein kinase ArgK or related GTPase of G3E family
Signal recognition particle GTPase
Signal recognition particle GTPase
Ni2+-binding GTPase involved in regulation of expression and maturation of urease and hydrogenase
Heme/copper-type cytochrome/quinol oxidase, subunit 2
Heme/copper-type cytochrome/quinol oxidase, subunit 2
Heme/copper-type cytochrome/quinol oxidase, subunit 1 and 3
Heme/copper-type cytochrome/quinol oxidase, subunit 1
Integrase
Intergrase/Recombinase
Site-specific recombinase XerD
Lactaldehyde dehydrogenase, Succinate semialdehyde dehydrogenase or other NAD-dependent aldehyde dehydr
Predicted metal-dependent hydrolase related to alanyl-tRNA synthetase HxxxH domain and second additional dor
Alanyl-tRNA synthetase
Predicted metal-dependent hydrolase related to alanyl-tRNA synthetase HxxxH domain
Chaperonin GroEL (HSP60 family)
Short-chain alcohol dehydrogenase
Short-chain alcohol dehydrogenase
Short-chain alcohol dehydrogenase
Short-chain dehydrogenase
DNA-directed RNA polymerase subunit K/omega
Branched-chain amino acid ABC-type transport system, permease component
ABC-type branched-chain amino acid transport system, permease component
Acetyl-CoA acetyltransferase
Acetyl-CoA acetyltransferase
Acetyl-CoA acetyltransferase
Acetyl-CoA acetyltransferase
Acetyl-CoA acetyltransferase
Predicted nucleic-acid-binding protein containing a Zn-ribbon
Predicted nucleic-acid-binding protein containing a Zn-ribbon
Predicted nucleic-acid-binding protein containing a Zn-ribbon

Predicted nucleic-acid-binding protein containing a Zn-ribbon
OB-fold domain and Zn-ribbon containing protein, possible acyl-CoA-binding protein
Predicted nucleic-acid-binding protein containing a Zn-ribbon
DNA repair photolyase
NADPH-dependent glutamate synthase beta chain or related oxidoreductase
Thioredoxin reductase
NAD(FAD)-dependent dehydrogenase
Uncharacterized conserved protein
Glycine cleavage system H protein (lipoate-binding)
RecB family endonuclease acting on branched DNA substrates
ATP-dependent 26S proteasome regulatory subunit
Cell division ATPase of the AAA+ class, ESCRT system component
ATPase of the AAA+ class , CDC48 family
Uncharacterized conserved membrane protein
Na+/proline symporter
3,4-dihydroxy-2-butanone 4-phosphate synthase
Archaeal riboflavin synthase
Riboflavin synthase beta-chain
Uridine phosphorylase
Purine-nucleoside phosphorylase
Purine nucleoside phosphorylase
Zn-dependent protease with chaperone function
Zn-dependent protease with chaperone function
Zn-dependent protease with chaperone function
Uncharacterized conserved protein
Succinyl-CoA synthetase, beta subunit
Acyl-CoA synthetase, ATP-grasp containing subunit
Succinyl-CoA synthetase, alpha subunit
Acyl-CoA synthetase (NDP forming)
Predicted prefoldin, molecular chaperone implicated in de novo protein folding
Prefoldin, chaperonin cofactor
Ribosomal protein S19E (S16A)
Predicted RNA-binding protein containing KH domain, possibly ribosomal protein
ATP-dependent DNA ligase
NAD kinase
Archaeal fructose-1,6-bisphosphatase or related enzyme of inositol monophosphatase family
Predicted inorganic polyphosphate/ATP-NAD kinase
Homoserine dehydrogenase
Glutamate dehydrogenase/leucine dehydrogenase
Subunit of KEOPS complex (Cgi121BUD32KAE1)
Radical SAM superfamily enzyme
Radical SAM superfamily enzyme
2-methylthioadenine synthetase
Histone acetyltransferase
RNase P/RNase MRP subunit POP5
dTDP-4-dehydrorhamnose reductase
Nucleoside-diphosphate-sugar epimerase
dTDP-D-glucose 4,6-dehydratase

Phosphodiesterase of AP superfamily
Uncharacterized protein of the AP superfamily
Glycosyltransferase
Predicted glycosyltransferase
Glycosyltransferase
Glycosyltransferase
Glycosyltransferase
UDP-N-acetylglucosamine:LPS N-acetylglucosamine transferase
Glycosyltransferase
Glycosyltransferase
SAM-dependent methyltransferase
SAM-dependent methyltransferase
SAM-dependent methyltransferase
Glycosyltransferase
Glycosyltransferase
Glycosyltransferase
Glycosyltransferase
Glycosyltransferase
Glycosyltransferase
Glycosyltransferase
Glycogen synthase
Glucan phosphorylase
RecB-like nuclease fused to coiled-coil domain
RecB-nuclease fused to coiled-coil domain
RecB family nuclease with coiled-coil N-terminal domain
RecB family nuclease with coiled-coil N-terminal domain, paREP7 family protein
Cysteine synthase
Threonine dehydratase
Tryptophan synthase beta-subunit
Threonine synthase and cysteate synthase
CRISPR system related protein, RAMP superfamily Cas6 group
CRISPR system related protein, RAMP superfamily Cas6 group
HD superfamily nuclease
HD superfamily nuclease
CRISPR-associated helicase Cas3
Transcriptional regulator containing HTH domain, ArsR family
CRISPR-associated protein Cas1
Zn-dependent alcohol dehydrogenase
Zn-dependent alcohol dehydrogenase
D-arabinose 1-dehydrogenase or another Zn-dependent alcohol dehydrogenase
NADPH:quinone reductase or related Zn-dependent oxidoreductase
Threonine dehydrogenase or related Zn-dependent dehydrogenase
ABC-type Na+ efflux pump, permease component
ABC-type multidrug transport system, permease component
ABC-type multidrug transport system, permease component
ABC-type multidrug transport system, permease component
ABC-type multidrug transport system, permease component
ABC-type multidrug transport system, permease component

Hemerythrin HHE cation binding domain containing protein
Co/Zn/Cd efflux system component
Predicted Co/Zn/Cd cation transporter
Nicotinic acid phosphoribosyltransferase
Nicotinate-nucleotide pyrophosphorylase
Pyrimidine reductase, riboflavin biosynthesis
Anaerobic dehydrogenase
Uncharacterized anaerobic dehydrogenase
Molybdopterin oxidoreductase
Molybdopterin oxidoreductase, contains molybdopterin-binding domain
Fe-S-cluster-containing dehydrogenase component
Fe-S-cluster-containing dehydrogenase component
Uncharacterized component of anaerobic dehydrogenase
Uncharacterized component of anaerobic dehydrogenase
Single-stranded DNA-binding replication protein A (RPA), large (70 kD) subunit or related ssDNA-binding protein
N-methylhydantoinase A/acetone carboxylase, beta subunit
N-methylhydantoinase B/acetone carboxylase, alpha subunit
Phytoene dehydrogenase or related enzyme
Reverse gyrase
Topoisomerase IA
Acyl-coenzyme A synthetase/AMP-(fatty) acid ligase
Molybdenum cofactor biosynthesis enzyme
Transcriptional regulator, contains wHTH domain
Undecaprenyl pyrophosphate synthase
ABC-type transport system, periplasmic component
ABC-type transport system, periplasmic component
ABC-type transport system, periplasmic component
NADH:ubiquinone oxidoreductase subunit 4 (chain M)
NADH dehydrogenase subunit L
NADH dehydrogenase subunit N
NADH dehydrogenase subunit I
Formate hydrogenlyase subunit 4
NADH dehydrogenase subunit H
Ni,Fe-hydrogenase III large subunit and subunit G
NADH dehydrogenase subunit D
NADH dehydrogenase subunit C
Ni,Fe-hydrogenase III small subunit
F420H2 dehydrogenase subunit, related to NADH:ubiquinone oxidoreductase 20 kD subunit
NADH dehydrogenase subunit A
Translation elongation factor G, EF-G (GTPase)
Translation initiation factor 2 (IF-2; GTPase)
Translation elongation factor EF-1alpha (GTPase)
GTPase
Translation initiation factor 2, gamma subunit (eIF-2gamma; GTPase)
Selenocysteine-specific translation elongation factor or SelB-II domain
Small-conductance mechanosensitive channel
Uncharacterized conserved membrane protein
Small-conductance mechanosensitive channel

RNase PH-related exoribonuclease
Ribonuclease PH
Cation transport ATPase
Cation transport ATPase
Transcriptional regulator, IclR family
Transcriptional regulator (Lrp/AsnC family)
Transcriptional regulator (Lrp/AsnC family)
Transcriptional regulator (Lrp/AsnC family)
lysine biosynthesis protein LysW
Glutathione synthase/glutaminyl transferase/alpha-L-glutamate ligase
Biotin carboxylase
Carbamoylphosphate synthase large subunit
Phosphoribosylaminoimidazole carboxylase (NCAIR synthetase)
Formate-dependent phosphoribosylglycinamide formyltransferase (GAR transformylase)
Pyruvate:ferredoxin oxidoreductase or related 2-oxoacid:ferredoxin oxidoreductase, beta subunit
Pyruvate:ferredoxin oxidoreductase or related 2-oxoacid:ferredoxin oxidoreductase, beta subunit
Pyruvate:ferredoxin oxidoreductase or related 2-oxoacid:ferredoxin oxidoreductase, gamma subunit
Pyruvate:ferredoxin oxidoreductase or related 2-oxoacid:ferredoxin oxidoreductase, gamma subunit
Pyruvate:ferredoxin oxidoreductase or related 2-oxoacid:ferredoxin oxidoreductase, gamma and delta subunit
Pyruvate:ferredoxin oxidoreductase or related 2-oxoacid:ferredoxin oxidoreductase, delta subunit
Pyruvate:ferredoxin oxidoreductase or related 2-oxoacid:ferredoxin oxidoreductase, alpha subunit and gamma
Pyruvate:ferredoxin oxidoreductase or related 2-oxoacid:ferredoxin oxidoreductase, alpha subunit
Indolepyruvate ferredoxin oxidoreductase, alpha and beta subunit
Predicted membrane protein
D-aminoacyl-tRNA deacylase, involved in ethanol tolerance
Predicted oxidoreductase (related to aryl-alcohol dehydrogenase)
Aldo/keto reductase, related to diketogulonate reductase
Aldo/keto reductase, related to diketogulonate reductase
Predicted dehydrogenase
Transcriptional regulator (Lrp/AsnC family)
Putative translation initiation inhibitor, yjgF family
SAM-dependent methyltransferase
Translation initiation factor 2, beta subunit (eIF-2beta)/eIF-5 N-terminal domain
Dipeptidyl aminopeptidase/acylaminoacyl-peptidase
Serine protease of the peptidase family S9A
Alpha/beta superfamily hydrolase
Dienelactone hydrolase or related enzyme
Cytotoxic translational repressor of toxin-antitoxin stability system
Cytotoxic translational repressor of toxin-antitoxin stability system
Zinc finger domain containing protein (CDGSH-type)
Uncharacterized conserved protein
Uncharacterized conserved protein
Predicted peroxiredoxin
3-polyprenyl-4-hydroxybenzoate decarboxylase or related decarboxylase
Predicted solute binding protein
ABC-type transport system, periplasmic component
Acylphosphatase
Dinucleotide-utilizing enzyme involved in molybdopterin and thiamine biosynthesis

Archaeal S-adenosylmethionine synthetase
Transcriptional regulator containing HTH domain, ArsR family
Transcriptional regulator containing HTH domain, ArsR family
Predicted RNA-binding protein homologous to eukaryotic snRNP
2,3-bisphosphoglycerate-independent phosphoglycerate mutase
Aconitase A
Homoaconitate hydratase/3-isopropylmalate dehydratase large subunit family protein
Uncharacterized conserved protein
Arginase family enzyme
tRNA splicing endonuclease
tRNA splicing endonuclease
3-polyprenyl-4-hydroxybenzoate decarboxylase
Phosphopantothenoylcysteine synthetase/decarboxylase
Pyruvate/2-oxoglutarate dehydrogenase complex, dihydrolipoamide acyltransferase (E2) component or related er
Acyl-CoA dehydrogenase
Methylmalonyl-CoA mutase, C-terminal domain/subunit (cobalamin-binding)
Inorganic pyrophosphatase
Uncharacterized conserved protein
Uncharacterized conserved protein
Cysteine desulfurase activator SufB
Asp-tRNAAsn/Glu-tRNAGln amidotransferase A subunit or related amidase
Asp-tRNAAsn/Glu-tRNAGln amidotransferase B subunit
Archaeal Glu-tRNAGln amidotransferase subunit E (contains GAD domain)
Rieske Fe-S protein
Cytochrome b subunit of the bc complex
Ribosomal protein S13
Adenylate cyclase, class 2 (thermophilic)
Geranylgeranyl pyrophosphate synthase
Geranylgeranyl pyrophosphate synthase
Predicted dioxygenase
Aromatic ring-opening dioxygenase, catalytic LigB subunit related enzyme
2'-5' RNA ligase
Signal peptidase I
Signal peptidase I
Release factor eRF1
Peptide chain release factor 1 (eRF1)
Adenylosuccinate lyase
Argininosuccinate lyase
Fumarase
Ribosomal protein L7AE
Ribosomal protein L30E
Archaeal DNA-binding protein
Phosphoglycerate dehydrogenase or related dehydrogenase
Lactate dehydrogenase or related 2-hydroxyacid dehydrogenase
Ribosomal protein S10
Uncharacterized conserved protein
Transcription elongation factor
Transcription elongation factor

DNA-directed RNA polymerase subunit B
TATA-box binding protein (TBP), component of TFIID and TFIIIB
3-hydroxy-3-methylglutaryl CoA synthase
Membrane associated serine protease
SAM-dependent methyltransferase
SAM-dependent methyltransferase
SAM-dependent methyltransferase
SAM-dependent methyltransferase
SAM-dependent methyltransferase
Predicted transcriptional regulator
Flp pilus assembly protein TadC
Archaeal flagella assembly protein J
Flp pilus assembly protein TadC
Type IV secretory pathway, VirB11 component, or related ATPase involved in archaeal flagella biosynthesis
Putative archaeal flagellar protein G
Putative archaeal flagellar protein F
Archaeal flagellins
Predicted nucleotidyltransferase
Predicted nucleotidyltransferase
Molecular chaperone (HSP20 family)
Molecular chaperone (HSP20 family)
Molecular chaperone (HSP20 family)
Putative sterol carrier protein
Predicted metal-sulfur cluster biosynthetic enzyme
Isoleucine patch superfamily protein
Predicted metal-sulfur cluster biosynthetic enzyme
SpoU rRNA Methylase family enzyme
Predicted transcription factor, homolog of eukaryotic MBF1
Fe2+/Zn2+ uptake regulation protein, fur/PerR
Adenosylcobinamide amidohydrolase
GTP:adenosylcobinamide-phosphate guanylyltransferase
Molybdopterin-guanine dinucleotide biosynthesis protein A
GT-A family glycosyltransferase involved in molybdopterin guanine dinucleotide biosynthesis
ParB-like nuclease domain
Methionine synthase II (cobalamin-independent)
Methionine synthase II (cobalamin-independent)
Dolichol kinase family protein
Thymidylate synthase
Ribosomal protein S17E
Tyrosyl-tRNA synthetase
Tryptophanyl-tRNA synthetase
Thymidylate kinase
Endonuclease IV
Sugar phosphate isomerase/epimerase
Sugar phosphate isomerase/epimerase
CTP-dependent Riboflavin kinase
Predicted transcriptional regulator
Uncharacterized conserved protein

Glutamine synthetase
Short-chain alcohol dehydrogenase
Membrane protein implicated in regulation of membrane protease activity
Membrane protease subunit, stomatin/prohibitin homolog
Zn-ribbon domain containing protein
Membrane protease subunit, stomatin/prohibitin homolog
Sec-independent protein secretion pathway component TatC
Transcription antiterminator NusG
Uncharacterized conserved protein
Protein implicated in ribosomal biogenesis, Nop56p homolog
L-asparaginase/archaeal Glu-tRNAGln amidotransferase subunit D
Aerobic-type carbon monoxide dehydrogenase, small subunit CoxS/CutS homolog
Aerobic-type carbon monoxide dehydrogenase, middle subunit CoxM/CutM homolog
Carbon monoxide dehydrogenase subunit G, CoxG
Carbon monoxide dehydrogenase subunit G, CoxG
Xanthine and CO dehydrogenase maturation factor, XdhC/CoxF family
Predicted metal-dependent hydrolase of the TIM-barrel fold
Predicted metal-dependent hydrolase of the TIM-barrel fold
Predicted metal-dependent hydrolase of the TIM-barrel fold
Predicted metal-dependent hydrolase of the TIM-barrel fold
Predicted metal-dependent hydrolase of the TIM-barrel fold
CRISPR system related protein, COG1517 family
Archaeal serine protease
Lipoate-protein ligase A
Biotin-(acetyl-CoA carboxylase) ligase
Amidase related to nicotinamidase
Ribosomal protein S6E (S10)
Putative threonine efflux protein
Ribosomal protein L24E
Predicted P-loop ATPase fused to an acetyltransferase
Putative translation factor (SUA5)
Kef-type K+ transport system, membrane component
Kef-type K+ transport system, membrane component fused to CBS domains
Kef-type K+ transport system, predicted NAD-binding component
Kef-type K+ transport system, membrane component
Thiol-disulfide isomerase or thioredoxin
Dihydropteroate synthase or related enzyme
Transcription initiation factor TFIIB, Brf1 subunit/Transcription initiation factor TFIIB
Predicted TIM-barrel enzyme
Phosphoribosylanthranilate isomerase
Ferredoxin-like protein
Ferredoxin-like protein
Acyl CoA:acetate/3-ketoacid CoA transferase, alpha subunit
Translation elongation factor EF-1beta
Predicted Zn-ribbon RNA-binding protein with a function in translation
Broad specificity phosphatase PhoE or related phosphatase
Phosphohistidine phosphatase SixA
Oligoketide cyclase/lipid transport protein family

Multiple antibiotic transporter
Acetolactate synthase large subunit or other thiamine pyrophosphate-requiring enzyme
Pyruvate oxidase
Acetolactate synthase large subunit or other thiamine pyrophosphate-requiring enzyme
Maleate cis-trans isomerase
Highly conserved protein containing a thioredoxin domain
Predicted membrane protein
Anthranilate phosphoribosyltransferase
Anthranilate/para-aminobenzoate synthase component I
Conserved protein/domain typically associated with flavoprotein oxygenase, DIM6/NTAB family
Rhodanese-related sulfurtransferase
Rhodanese-related sulfurtransferase
Polysulphide reductase
Formate-dependent nitrite reductase, membrane component
Sugar-specific transcriptional regulator TrmB
Uncharacterized membrane protein, required for N-linked glycosylation
Uroporphyrinogen-III synthase
Predicted permease
Nucleotide-binding protein, UspA family
Sulfite reductase, beta subunit (hemoprotein)
Predicted redox protein, regulator of disulfide bond formation
Peroxiredoxin family protein
Peroxiredoxin family protein
Predicted peroxiredoxin
Predicted peroxiredoxin
Predicted redox protein, regulator of disulfide bond formation
Predicted redox protein, regulator of disulfide bond formation
Predicted peroxiredoxin
S-layer domain
Isopropylmalate/homocitrate/citramalate synthase
3-dehydroquinate dehydratase
Chorismate mutase
Mn-dependent transcriptional regulator (DtxR family)
Mn-dependent transcriptional regulator (DtxR family)
Transcriptional regulator, contains wHTH domain
HEPN domain containing protein
Minimal nucleotidyltransferase
Uncharacterized conserved protein
Uncharacterized conserved protein
Uncharacterized conserved protein
Transposase
Transposase
Transposase
Transposase and inactivated derivatives, IS1 family
Transposase
Aromatic ring hydroxylase
Uncharacterized homolog of gamma-carboxymuconolactone decarboxylase subunit
Predicted membrane protein

Transglutaminase-like enzyme, putative cysteine protease
6-pyruvoyl-tetrahydropterin synthase
Organic radical activating enzyme
Predicted transporter of the RND superfamily
Ferredoxin domain containing protein
2-polyprenylphenol hydroxylase or related flavodoxin oxidoreductase
Preprotein translocase subunit Sss1
Exonuclease III
ATP phosphoribosyltransferase
Polysaccharide biosynthesis protein, Mvin family
RHH/CopG DNA binding protein
RHH/CopG DNA binding protein
PIN domain containing protein
PIN domain containing protein
PIN domain containing protein
PIN domain containing protein
Predicted membrane protein
3-isopropylmalate dehydratase small subunit
Uncharacterized FAD-dependent dehydrogenase
Heterodisulfide reductase, subunit A, polyferredoxin
Heterodisulfide reductase, subunit A or related polyferredoxin
Heterodisulfide reductase, subunit A or related polyferredoxin
Transcriptional regulator, contains HTH domain
Succinate dehydrogenase/fumarate reductase, cytochrome b subunit
Sirohydrochlorin ferrochelatase
Precorrin isomerase
ABC-type cobalt transport system, permease component CbiQ or related transporter
Mg2+ and Co2+ transporter
Phosphate/sulphate permease
Predicted transcriptional regulator, C-terminal HTH-like domain
Predicted transcriptional regulator, C-terminal HTH-like domain
Predicted transcriptional regulator, C-terminal HTH-like domain
Uncharacterized beta/alpha-propeller protein, likely forms oligomers
HAD superfamily hydrolase
HAD superfamily hydrolase
Peptidase A24A, prepilin type IV
Succinyl-CoA:mesaconate CoA-transferase or predicted acyl-CoA transferase/carnitine dehydratase or
ABC-type antimicrobial peptide transport system, permease component
Predicted Rossmann fold nucleotide-binding protein
ABC-type transport system involved in multi-copper enzyme maturation, permease component
Archaeal/vacuolar-type Na+/H+-ATPase, subunit K
Archaeal/vacuolar-type H+-ATPase subunit C
Kynurenine formamidase
Phosphoribosylcarboxyaminoimidazole (NCAIR) mutase
RNA-binding protein involved in rRNA processing
PKD repeats containing protein
WD40-like repeat
Beta-propeller repeat containing protein

Beta-propeller repeat containing protein
Chloride channel protein EriC
Fe-S-cluster containining protein
Uncharacterized conserved protein
Coenzyme F390 synthetase
Coenzyme F390 synthetase
Esterase/lipase
Phosphate transport regulator (distant homolog of PhoU)
Transcriptional regulator, TetR/AcrR family
CRISPR system related protein, small subunit of CASCADE complex
CRISPR system related protein, small subunit of CASCADE complex
CRISPR system related protein, RAMP superfamily Cas7 group
CRISPR system related protein, RAMP superfamily Cas7 group
CRISPR system related protein, RAMP superfamily Cas7 group
CRISPR system related protein, RAMP superfamily Cas7 group
CRISPR system related protein, RAMP superfamily Cas7 group
CRISPR system related protein, RAMP superfamily Cas5 group
CRISPR associated protein, polymerase-related domain and HD family nuclease
CRISPR system related protein Cas5, RAMP superfamily
Phosphoribosyl-AMP cyclohydrolase
Phosphoribosyl-ATP pyrophosphohydrolase
Predicted antitoxin, copG family
Permease of the major facilitator superfamily
Permease of the major facilitator superfamily
Permease of the major facilitator superfamily
Permease of the major facilitator superfamily
Permease of the major facilitator superfamily
Permease of the major facilitator superfamily
Permease of the major facilitator superfamily
Permease of the major facilitator superfamily
Permease of the major facilitator superfamily
Permease of the major facilitator superfamily
Permease of the major facilitator superfamily
Sec-independent protein secretion pathway component
Biotin carboxyl carrier protein
Pyruvate/2-oxoglutarate dehydrogenase complex, dihydrolipoamide acyltransferase (E2) component (C-terminal f
SAM-dependent methyltransferase
SAM-dependent methyltransferase
Acetyl-CoA carboxylase, carboxyltransferase component
Lactoylglutathione lyase or related enzyme
Predicted membrane protein
Predicted membrane protein
Methylated DNA-protein cysteine methyltransferase
Asp-tRNAAsn/Glu-tRNAGln amidotransferase C subunit
Predicted RNA-binding protein containing PUA-domain
PIN domain containing protein
PIN domain containing protein
NifX family protein

NifX family protein
NifX family protein
Thiamine biosynthesis protein ThiC
Transposase, IS5 family
Transposase
Uncharacterized conserved protein
Heavy-metal-associated domain (HMA)
Copper-ion-binding protein
Polyferredoxin
Permease of the major facilitator superfamily
Permease of the major facilitator superfamily
Peptide methionine sulfoxide reductase
Serine protease Do (heat-shock protein)
Uncharacterized conserved membrane protein
Deoxyribodipyrimidine photolyase
ABC-type multidrug transport system, ATPase and permease component
Ferredoxin subunit of nitrite reductase or ring-hydroxylating dioxygenase
Ferredoxin subunit of nitrite reductase or ring-hydroxylating dioxygenase
Ferredoxin subunit of nitrite reductase or ring-hydroxylating dioxygenase
Ferredoxin subunit of nitrite reductase or ring-hydroxylating dioxygenase
Carbonic anhydrase
Predicted membrane protein, DoxD family
Predicted membrane protein, DoxD family
Predicted dithiol-disulfide isomerase involved in polyketide biosynthesis
Ca2+/Na+ antiporter
Predicted membrane protein
Predicted membrane protein
Uncharacterized protein containing a von Willebrand factor type A (vWA) domain
Pirin-related protein
Phytoene/squalene synthetase
Mevalonate pyrophosphate decarboxylase
Pterin-4a-carbinolamine dehydratase
Ribonuclease HI
Bifunctional 4-alpha-glucanotransferase/ amylo-alpha-1,6-glucosidase
1,4-alpha-glucan branching enzyme
Maltooligosyl trehalose synthase
Preprotein translocase subunit Sec61beta
Predicted aminopeptidase, Iap family
Aminopeptidase N
Uncharacterized conserved protein
Cupin domain containing protein
Gentisate 1,2-dioxygenase
Cupin domain fused to uncharacterized domain of COG4309
Eukaryotic-type DNA primase, large subunit
Nucleoside-diphosphate-sugar epimerase
Chlorite dismutase
TPR repeats containing protein
TPR repeats containing protein

TPR repeats containing protein
Membrane-associated phospholipid phosphatase
NADH dehydrogenase subunit 4L (K,kappa)
Arginine deiminase
Uncharacterized membrane-associated protein, DedA family
DedA family membrane protein
Uncharacterized conserved protein
Site-specific recombinase, DNA invertase Pin homolog
Predicted site-specific integrase-resolvase
AAA+ superfamily ATPase fused to HTH nad RecB nuclease domains
Predicted ATPase (AAA+ superfamily)
Predicted ATPase (AAA+ superfamily)
Predicted ATPase (AAA+ superfamily)
Predicted membrane protein
Transcriptional regulator, MarR family
Transcriptional regulator, MarR family
UDP-N-acetylmuramyl pentapeptide phosphotransferase/UDP-N-acetylglucosamine-1-phosphate transferase
Collagenase family protease
Uncharacterized conserved protein
Uncharacterized conserved protein
Predicted ATPase
ATPase, predicted component of phage defense system
ATPase, predicted component of phage defense system
Recombinational DNA repair ATPase RecF
Predicted exosome subunit/U3 small nucleolar ribonucleoprotein (snoRNP) component, contains IMP4 domain
Uncharacterized conserved protein, AIG2 family
Glycosyl hydrolase family 57
Glycosyl hydrolase family 57
Glycosyl hydrolase family 15
Glycosyl hydrolase family 15
Glycogen debranching enzyme
Predicted transcriptional regulator
Uncharacterized protein related to the periplasmic component of the Tol biopolymer transport system
Protein-tyrosine phosphatase
Transcriptional regulator, MarR family
TPR repeats containing protein
SWIM zinc finger containing protein
CRISPR system related protein, COG1517 family
CRISPR system related protein, COG1517 family
CRISPR system related protein, COG1517 family
Purine-cytosine permease or related protein
Cytosine/uracil/thiamine/allantoin permease
Uncharacterized protein, LmbE homolog
Amino acid transporter
Amino acid transporter
Amino acid transporter
Amino acid transporter
Amino acid transporter

CRISPR system related protein, RAMP superfamily Cas7 group
CRISPR system related heleicase, Cas3 (C-terminal HD nuclease domain)
Uncharacterized conserved protein
Putative protein-S-isoprenylcysteine methyltransferase
Transposase
Transposase
CRISPR system related protein Cas7, RAMP superfamily
Hydrogenase 4 membrane component (E)
Predicted glutamine amidotransferase
Amino acid transporter
Amino acid transporter
Amino acid transporter
Amino acid transporter
Amino acid transporter
Thiamine pyrophosphate-requiring enzyme, carboxylase
Acetolactate synthase large subunit or other thiamine pyrophosphate-requiring enzyme
Ribonucleotide reductase, beta subunit (ferritin domain)
Predicted N-acetylglucosamine kinase
Permease of the major facilitator superfamily
Alpha-mannosidase
Alpha-glucosidase, family 31 of glycosyl hydrolase
Subtilase family protease
Subtilase family protease
Thermopsin-like protease
Thermopsin-like protease
Thermopsin-like protease
Thermopsin-like protease
Thermopsin-like protease
Membrane associated serine/threonine protein kinase
Predicted redox protein, regulator of disulfide bond formation
Uncharacterized conserved protein
Uncharacterized conserved protein
Uncharacterized conserved protein
Polyferredoxin
Predicted transciptional regulator, contains HTH domain
Uncharacterized conserved protein
Sulfocyanin
Predicted integral membrane protein
Predicted integral membrane protein
Uncharacterized conserved protein
Predicted metal-dependent hydrolase
PIN domain containing protein
HEPN domain containing protein
HEPN domain containing protein
HEPN domain containing protein
Ribonucleotide reductase associated ATP-cone domain
ATP cone domain
HEPN domain containing protein

HEPN domain containing protein
KID-like repeats conatining protein, paREP15 family
Transcriptional regulator of GntR family
P-loop NTPase superfamily protein
Predicted aspartyl protease
Transcriptional regulator, MarR family
Predicted membrane protein
CopG family transcriptional regulator
Uncharacterized conserved protein
Uncharacterized conserved membrane protein
Uncharacterized conserved protein
Uncharacterized conserved protein
Uncharacterized conserved protein
GTPase subunit of restriction endonuclease
CRISPR system associated protein, predicted small subunit of CASCADE complex
Uncharacterized conserved protein
Lipoate-protein ligase A associated domain
Coenzyme PQQ synthesis protein D
Uncharacterized conserved protein
Uncharacterized conserved protein
TlpA/TrxA-like family protein
TlpA/TrxA-like family protein
TlpA/TrxA-like family protein
Predicted antitoxin, copG family
Predicted antitoxin, copG family
CRISPR system related protein, COG1517 family
Uncharacterized conserved protein
Predicted O-methyltransferase
Uncharacterized conserved protein
Uncharacterized conserved protein
Uncharacterized conserved protein
Uncharacterized conserved protein
Uncharacterized conserved protein
Uncharacterized conserved protein
Uncharacterized conserved protein
Glycosyl hydrolase family 12
Transcriptional regulator containing HTH domain, ArsR family
Uncharacterized conserved protein
Uncharacterized conserved protein
Predicted bile acid beta-glucosidase
Predicted bile acid beta-glucosidase
Predicted bile acid beta-glucosidase
Predicted bile acid beta-glucosidase
Uncharacterized conserved protein
Uncharacterized conserved protein
Flagellar protein G
Flagellar protein G
Predicted transcriptional regulator

RadC related protein, contains PAD1/JAB1 superfamily protase domain and ENDO3 nuclease domain
CRISPR system related protein, RAMP superfamily Cas7 group
CRISPR system related protein, RAMP superfamily Cas7 group
Transposase
Ankyrin repeats containing protein
Uncharacterized conserved protein
Uncharacterized conserved protein
Transcriptional regulator (GntR family)
Predicted aspartyl protease
Pepsin-like aspartate protease
Uncharacterized conserved protein
Uncharacterized conserved protein
Eukaryotic-type DNA primase, catalytic (small) subunit homolog, fused to HTH domain
Uncharacterized conserved protein
Type IV secretory pathway, VirB4 component
Uncharacterized conserved protein
2-amino-3,7-dideoxy-D-threo-hept-6-ulosonic acid synthase, DhnA-aldolase family
Dihydroxyacid dehydratase/phosphogluconate dehydratase
Predicted phosphatase
Deoxycytidine deaminase
Ribosomal protein L40E
5'-3' exonuclease (including N-terminal domain of PolI)
Uncharacterized conserved protein
Gamma-glutamyltransferase
Potential cell division protein, a component of ESCRT system
SHS2 domain protein implicated in nucleic acid metabolism
Transcriptional regulator containing HTH domain, ArsR family
Uncharacterized conserved protein
Predicted transcriptional regulator
Transcription factor homologous to NACalpha-BTF3
Uncharacterized conserved protein
Predicted membrane-associated Zn-dependent protease
Minimal nucleotidyltransferase
Ribosomal protein L2
Membrane protein
RNA methyltransferase, SPOUT superfamily
Ribosomal protein L3
Ribosomal protein L4
Ribosomal protein L23
Pyridoxine biosynthesis enzyme
Uncharacterized conserved protein
Transcription elongation factor Spt4/RpoE2, zinc finger protein
DNA helicase TIP49, TBP-interacting protein
Protein related to penicillin acylase
Zn-dependent dipeptidase, microsomal dipeptidase homolog
Ribosomal protein L30
Ribosomal protein S5
Ribosomal protein L18

Ribosomal protein L19E
Ribosomal protein L6P
Ribosomal protein S8
Ribosomal protein L5
Ribosomal protein S4E
Ribosomal protein L24
Ribosomal protein L14
Ribosomal protein S17
Ribosomal protein S3
Ribosomal protein L22
Ribosomal protein S19
Acyl-CoA dehydrogenase
Archaeal/vacuolar-type H+-ATPase subunit D
Archaeal/vacuolar-type H+-ATPase subunit F
Uncharacterized conserved protein
Ribosome associated protein L46a
Uncharacterized conserved protein
CDP-diglyceride synthetase
Translation initiation factor 2, alpha subunit (eIF-2alpha)
Ribosomal protein S27E
Ribosomal protein L44E
Eukaryotic-type DNA primase, catalytic (small) subunit
DNA-directed RNA polymerase, subunit L
Diphthamide synthase subunit DPH2
Ribosomal protein L10AE/L16
Chromatin protein Cren7
DNA-binding protein, stimulates sugar fermentation
ATPase (PilT family)
Predicted nucleotidyltransferase
Pyruvate kinase
Ribonuclease HII
EMG1/NEP1 rRNA methyltransferase
Uncharacterized conserved protein
Uncharacterized conserved protein
RNA 3'-terminal phosphate cyclase
Ribosomal protein L37E
Predicted GTPase or GTP-binding protein
Uracil phosphoribosyltransferase
Ribosomal protein L21E
Predicted RNA-binding protein
Dimethyladenosine transferase (rRNA methylation)
Uncharacterized conserved protein
Chorismate synthase
5-enolpyruvylshikimate-3-phosphate synthase
Zn-finger protein
Transcription elongation factor Elf1, Zn ribbon-containing protein
S-adenosylhomocysteine hydrolase
Archaeal/vacuolar-type H+-ATPase subunit I

Uncharacterized conserved protein
Deoxyhypusine synthase
DNA topoisomerase VI, subunit A
Superoxide dismutase
Predicted ATPase involved in biogenesis of archaeal flagella
NMD protein affecting ribosome stability and mRNA decay
Predicted RNA-binding protein (contains KH domains)
ABC-type multidrug transport system, permease component
Predicted transcriptional regulator
RNA polymerase III subunit C34 (Lrp/AsnC family)
Ribosomal protein S8E
Wybutosine (yW) biosynthesis enzyme
Site-specific DNA methylase
Uncharacterized conserved protein
Diphthamide biosynthesis methyltransferase
Uncharacterized conserved protein
Uncharacterized conserved protein
Uncharacterized conserved protein
Ribosomal protein L14E/L6E/L27E
Ribosomal protein L34E
Preprotein translocase subunit SecY
Putative glycerate kinase
Uncharacterized conserved protein
Dihydrodipicolinate synthase/N-acetylneuraminate lyase
Cytidine deaminase
Wybutosine (yW) biosynthesis enzyme, Fe-S oxidoreductase
Ribosomal protein L20A (L18A)
Translation initiation factor 6 (eIF-6)
Ribosomal protein L39E
DNA-binding protein
Bifunctional fructose-1,6-bisphosphate aldolase/phosphatase FBPA/FBPase
Uncharacterized conserved protein
Ribosomal protein S24E
Ribosomal protein S27AE
Xanthosine triphosphate pyrophosphatase
Ribosomal protein S15P
Ribosomal protein S3AE
Predicted exosome subunit
dTDP-4-dehydrorhamnose 3,5-epimerase or related enzyme
ATP sulfurylase (sulfate adenylyltransferase)
CRISPR-associated protein Cas2
CRISPR-associated protein, RecB family exonuclease
Transposase related protein
Acyl-coenzyme A synthetase/AMP-(fatty) acid ligase
Archaeal GTP cyclohydrolase III
N-terminal nucleophile hydrolase superfamily protein
ERCC4-type nuclease
Ribosomal protein L37AE/L43A

Ribosomal protein L15E
Myo-inositol-1-phosphate synthase
Uncharacterized conserved protein
Uncharacterized conserved protein
Predicted metallopeptidase
Possible nuclease of RNase H fold, RuvC/YqgF family
Phosphatase of the HAD superfamily
Translation initiation factor 1 (eIF-1/SUI1)
Uncharacterized conserved protein
Predicted phosphatase homologous to the C-terminal domain of histone macroH2A1
Predicted CoA-binding protein
Peptidyl-tRNA hydrolase
Aspartate carbamoyltransferase, regulatory subunit
Uncharacterized protein involved in tolerance to divalent cations
Methylmalonyl-CoA mutase
ABC-type Fe3+-hydroxamate transport system, periplasmic component
Uncharacterized conserved protein
Predicted thioredoxin/glutaredoxin
Cysteine desulfurase activator ATPase
Citrate synthase
Triphosphoribosyl-dephospho-CoA synthetase
Ribosomal protein S4 or related protein
Ribosomal protein S11
DNA-directed RNA polymerase subunit D
Ribosomal protein L13
Ribosomal protein S9
DNA-directed RNA polymerase, subunit N (RpoN/RPB10)
Ribosomal protein S2
RNA 3'-P ligase, RtcB family protein
Zn-dependent carboxypeptidase
NAD-dependent protein deacetylase, SIR2 family
tRNA nucleotidyltransferase (CCA-adding enzyme)
Solo B3/4 domain (OB-fold DNA/RNA-binding) of Phe-aaRS-beta
Uncharacterized conserved protein
tRNA pseudouridine synthase D
Uncharacterized conserved protein
Ribosomal protein S7
Ribosomal protein S12
DNA-directed RNA polymerase subunit A''
DNA-directed RNA polymerase subunit A'
DNA-directed RNA polymerase, subunit H, RpoH/RPB5
Hydroxymethylglutaryl-CoA reductase
Uncharacterized conserved protein
Phosphopantothenate synthetase
Pantoate kinase
C4-type Zn-finger protein
Uncharacterized conserved protein
Replication initiator protein WhiP, Cdt1-like protein, contains HTH domain

Protein, predicted to be involved in DNA repair
Transcription initiation factor IIE, alpha subunit
DNA-directed RNA polymerase, subunit RPB8
NaMN:DMB phosphoribosyltransferase
Histidinol-phosphate/aromatic aminotransferase or cobyric acid decarboxylase
Cobalamin biosynthesis protein CobD/CbiB
Ribonucleotide reductase, alpha subunit
Translation elongation factor P (EF-P)/translation initiation factor 5A (eIF-5A)
DNA primase (bacterial type)
Uncharacterized conserved protein
Uncharacterized protein involved in propionate catabolism
Ribosomal protein L12E/L44/L45/RPP1/RPP2
Ribosomal protein L10
Ribosomal protein L1
PIN-domain and Zn ribbon
Uncharacterized conserved protein
Ribosomal protein S30
RecA/RadA recombinase
Predicted adenosine-specific kinase
Porphobilinogen deaminase
Delta-aminolevulinic acid dehydratase
Glutamyl- or glutaminyl-tRNA synthetase
Uncharacterized Rossmann fold enzyme
Ribosomal protein S26
Ribonuclease G and E
Uncharacterized conserved protein
S-adenosyl-l-methionine hydroxide adenosyltransferase
Acyl-CoA dehydrogenase
Predicted hydrolase of HD superfamily
PIN domain containing protein
Nucleoside diphosphate kinase
Ribosomal protein S28E/S33
Predicted RNA-binding protein of the translin family
Predicted membrane protein
Uncharacterized conserved protein with similarity to Ribonucleotide reductase and Pyruvate formate lyase
Zn-finger protein
Uncharacterized conserved protein
Phosphate starvation-inducible protein PhoH, predicted ATPase
Ribosomal protein S25
Predicted P-loop ATPase/GTPase
Predicted butyrate kinase
Predicted thioesterase
Aspartate/tyrosine/aromatic aminotransferase
Cobalamin-5-phosphate synthase
DNA-directed RNA polymerase, subunit RPC12/RpoP (contains C4-type Zn-finger)
RNase P subunit RPR2
5-formaminoimidazole-4-carboxamide-1-beta-D-ribofuranosyl 5'-monophosphate synthetase (purine biosynthesis
Methylase involved in ubiquinone/menaquinone biosynthesis

Predicted membrane protein
Acyl-CoA dehydrogenase
Predicted membrane protein
Histidinol dehydrogenase
Tellurite resistance protein or related permease
Nuclease of restriction endonuclease-like fold, implicated in nucleotide excision repair
Thermostable 8-oxoguanine DNA glycosylase
Uncharacterized protein required for formate dehydrogenase activity
Predicted transcriptional regulator
Ribosomal protein L11
Uncharacterized conserved protein
Cobalamin biosynthesis protein CbiD
L-lactate permease
Adenylosuccinate synthase
Uncharacterized conserved protein
Ammonia permease
Imidazoleglycerol-phosphate dehydratase
Predicted nuclease (RNAse H fold)
Subunit of KEOPS complex (Cgi121BUD32KAE1)
Phosphoribosylamine-glycine ligase
Phosphoribosylaminoimidazolesuccinocarboxamide (SAICAR) synthase
Zn finger protein HypA/HybF (possibly regulating hydrogenase expression)
Ni,Fe-hydrogenase maturation factor
HD superfamily phosphohydrolase
Glycerol uptake facilitator or related permease (Major Intrinsic Protein Family)
Phosphoenolpyruvate carboxylase
Uncharacterized protein of DIM6/NTAB family
Quinolinate synthase
Phosphoribosylformylglycinamidine (FGAM) synthase, PurS component
Ketol-acid reductoisomerase
Ribosomal protein L31E
Uncharacterized conserved protein
Predicted transcriptional regulator containing an HTH domain fused to a Zn-ribbon
Transcriptional regulator AbrB
Acetoacetate decarboxylase related enzyme
PIN domain containing protein
Methane/Phenol/Toluene Hydroxylase component
Urea amidohydrolase (urease) beta subunit
Urea amidohydrolase (urease) gamma subunit
Urease accessory protein UreF
Mn2+ and Fe2+ transporter of the NRAMP family
Fe2+ dependent formamide hydrolase, cofactor Fo biosynthesis enzyme
GTP cyclohydrolase I
Ferredoxin
Inosine-uridine nucleoside N-ribohydrolase
Nucleotidyltransferase/DNA polymerase involved in DNA repair
SAM-dependent methyltransferase
Bifunctional b-D-xylosidase/a-L-arabinosidase or related glycosidase

Predicted membrane protein
NADH dehydrogenase subunit J
Selenium binding protein SBP56 homolog
Phospholipid-binding protein
Nitric oxide reductase large subunit
Uncharacterized conserved protein
Uncharacterized conserved protein, contains Fer4-like metal-binding domain ortholog of eukaryotic RNase L inhib
Predicted permease
Undecaprenyl pyrophosphate phosphatase
Nucleoside-diphosphate-sugar epimerase
Asparaginase
Phenylacetic acid catabolic protein
Uncharacterized conserved membrane protein, Fun14 family
Malate/L-lactate dehydrogenase
Uncharacterized conserved protein
Transposase
ACT domain-containing protein
Uncharacterized conserved protein
Lysophospholipase L1 or related esterase
Uncharacterized conserved protein
CRISPR associated protein, RAMP family Cas5 group
Exopolyphosphatase
Roadblock/LC7 domain
Predicted secreted endonuclease distantly related to archaeal Holliday junction resolvase
Pyrimidine dimer DNA glycosylase, T4 endoV family
Predicted small metal-binding protein
Muconolactone delta-isomerase
Acyl CoA:acetate/3-ketoacid CoA transferase, beta subunit
Altronate dehydratase
SAF domain, altronate dehydratase related
Predicted membrane-bound metal-dependent hydrolase
Uncharacterized conserved protein
Uncharacterized conserved protein
Uncharacterized conserved protein
Predicted transporter component
FHA domain containing protein
Uncharacterized conserved protein
Permease of the major facilitator superfamily
Thiosulphate:quinone oxidoreductase (TQO) small subunit DoxA
Permease of the major facilitator superfamily
NAD(FAD)-dependent dehydrogenase
Enterochelin esterase or related enzyme
Uncharacterized conserved protein
Galactose mutarotase or related enzyme
Uncharacterized conserved protein
Hemerythrin HHE cation binding domain containing protein
Gluconolactonase
Uncharacterized conserved protein

ABC-type Mn/Zn transport system, ATPase component
Archaeal succinate dehydrogenase subunit D
Periplasmic substrate-binding protein
Predicted membrane protein
Predicted transciptional regulator, contains HTH domain
Predicted membrane protein
Glycosyltransferase family A
Protein containing potental lipid transfer domain, START superfamily
Ribbon-helix-helix protein, copG family
Predicted heme-degrading monooxygenase
ABC-type dipeptide/oligopeptide/nickel transport system, ATPase component
Endo-beta-mannanase
Beta-glucosidase/6-phospho-beta-glucosidase/beta-galactosidase
CopG/RHH family DNA binding protein, antitoxin
Carotene hydroxylase
Uncharacterized conserved protein
Zn-ribbon protein
Glycosyltransferase
Uncharacterized conserved protein
Uncharacterized conserved protein
Uncharacterized conserved protein
Uncharacterized conserved protein
Uncharacterized conserved protein, contains N-terminal coiled-coil domain
Malate synthase
OB-fold containing protein
PIN domain containing protein
Uncharacterized conserved protein
Predicted permease
Predicted transporter component
Uncharacterized conserved protein
Formate dehydrogenase, cytochrome b556 subunit
ABC-type sugar transport system, ATPase component
Dipeptidyl aminopeptidase/acylaminoacyl-peptidase
Uncharacterized conserved protein
Uncharacterized conserved protein
McrBC 5-methylcytosine restriction system component
Radical SAM superfamily enzyme
Pyrrolidone-carboxylate peptidase (N-terminal pyroglutamyl peptidase)
Phosphoenolpyruvate carboxykinase (GTP)
Uncharacterized conserved protein, DUF1177
Uncharacterized conserved protein
Uncharacterized conserved protein
Uncharacterized conserved protein
Uncharacterized conserved protein
Uncharacterized conserved protein
Chromosomal protein Sac7d/Sso7d
Uncharacterized conserved protein
Uncharacterized conserved protein

Terminal oxidase, subunit doxC
Terminal oxidase, subunit doxE
Uncharacterized conserved protein
Uncharacterized conserved protein
Uncharacterized conserved protein
Uncharacterized conserved protein
Uncharacterized conserved protein
Cell suface protein
Predicted transcriptional regulator
Uncharacterized conserved protein
Uncharacterized conserved membrane protein
Uncharacterized conserved protein
Uncharacterized conserved protein
Uncharacterized conserved protein
Uncharacterized conserved protein
Ferritin-like domain
Uncharacterized conserved protein
Sec-independent protein secretion pathway component
Uncharacterized conserved protein
Uncharacterized conserved protein
Uncharacterized conserved protein
Uncharacterized conserved protein
Predicted heterodisulfide reductase subunit
Uncharacterized conserved protein
Uncharacterized conserved protein
HAD superfamily hydrolase
Uncharacterized conserved protein
Uncharacterized conserved protein
Uncharacterized conserved protein
Uncharacterized conserved protein
Uncharacterized conserved protein
Uncharacterized conserved protein
Uncharacterized conserved protein
NurA family nuclease
Uncharacterized conserved protein
Uncharacterized conserved protein
Uncharacterized conserved protein
Homolog of transcription initiation factor TFIIB, contains Zn-ribbon domain
Zn-dependent hydrolase, glyoxylase family
Rossmann-fold NAD(P)(+)-binding protein
C2H2-type zinc finger
Predicted ATPase/Helicase of FtsK superfamily
Acetolactate synthase large subunit or other thiamine pyrophosphate-requiring enzyme
DNA-directed RNA polymerase subunit 13
Uncharacterized conserved protein
Uncharacterized conserved protein
Uncharacterized conserved protein
Glycosyltransferase

Uncharacterized conserved protein
Uncharacterized conserved protein
Uncharacterized conserved protein
Uncharacterized conserved protein
Uncharacterized conserved protein
Uncharacterized conserved protein
Uncharacterized conserved protein
Uncharacterized conserved protein
Uncharacterized conserved protein
Uncharacterized conserved protein
Uncharacterized conserved protein
Uncharacterized conserved protein
Metal-binding protein with CxxC..HxxxxH signature
Uncharacterized conserved protein
Uncharacterized conserved protein
Predicted ICC-like phosphoesterase
Uncharacterized conserved protein
Mn-dependent transcriptional regulator (DtxR family)
Uncharacterized conserved protein
Uncharacterized conserved protein
Acyl-CoA dehydrogenase
Acyl-CoA dehydrogenase
Phospholipase C
Uncharacterized conserved protein
Uncharacterized conserved protein
Predicted transcriptional regulator
Uncharacterized conserved protein
MFS family permease
Uncharacterized conserved protein
Methylase involved in ubiquinone/menaquinone biosynthesis
Uncharacterized conserved protein
Uncharacterized conserved protein
Uncharacterized conserved protein
Extracellular protein containing Kelch and FN3 domains
Uncharacterized conserved protein
Uncharacterized conserved protein
Uncharacterized conserved protein
Uncharacterized conserved protein
Uncharacterized conserved protein
Uncharacterized conserved protein
Predicted periplasmic solute-binding protein
Predicted component of pili system
Pilin/Flagellin
Uncharacterized conserved protein
Transcriptional regulator, xre family
NADPH:quinone reductase or related Zn-dependent oxidoreductase
ACT domain
Uncharacterized conserved protein

Transcriptional regulator, contains wHTH domain
Uncharacterized conserved protein
Uncharacterized conserved protein
Uncharacterized conserved protein
Uncharacterized conserved protein
Uncharacterized conserved helical repeat protein
Uncharacterized conserved protein
Uncharacterized conserved protein
Predicted periplasmic protein
Cell suface protein
ABC-type phosphate transport system, periplasmic component
2-keto-4-pentenoate hydratase
Predicted protease with the C-terminal PDZ domain
Uncharacterized conserved membrane protein
Uncharacterized conserved protein
Predicted metal permease
Predicted permease
Predicted membrane protein
Uncharacterized conserved protein
Uncharacterized conserved protein
Uncharacterized conserved protein
Predicted membrane protein
Cytochrome b558/566, subunit A
Cytochrome b558/566, subunit B
Uncharacterized conserved protein
Uncharacterized conserved protein
CRISPR system associated transcriptional regulator, containing Rossmann-like and HTH domain
HEPN domain containing protein
Uncharacterized conserved protein
High-affinity nickel permease
CRISPR-associated protein, RAMP family Cas5 group
Uncharacterized conserved protein
HAD superfamily hydrolase
Uncharacterized conserved protein
PIN domain containing protein
Uncharacterized conserved protein
Uncharacterized conserved protein
Pyrroloquinoline quinone (Coenzyme PQQ) biosynthesis protein C
Uncharacterized conserved protein
Predicted subunit of heme/copper-type cytochrome/quinol oxidase
Uncharacterized conserved protein
Uncharacterized conserved protein
Ferritin domain containing protein
Catechol 2,3-dioxygenase or other lactoylglutathione lyase family enzyme
Uncharacterized conserved protein, contains thioredoxin domain
S-layer protein SlaA
Uncharacterized conserved protein
Uncharacterized conserved protein

DKCLD domain, TruB/PUA associated
Uncharacterized conserved protein
Transcriptional regulators
Uncharacterized conserved protein
Hemerythrin HHE cation binding domain containing protein
Permease of the major facilitator superfamily
Uncharacterized conserved protein
Alpha-galactosidase
Predicted membrane protein
Acyl-CoA dehydrogenase
Uncharacterized conserved protein
Ni,Fe-hydrogenase maturation factor
Uncharacterized conserved protein
Uncharacterized conserved protein
Uncharacterized conserved protein
Predicted viral immunity protein, COG1517 superfamily, contains C-terminal PIN domain
Acyl-coenzyme A synthetase/AMP-(fatty) acid ligase
Acyl-coenzyme A synthetase/AMP-(fatty) acid ligase
Inactivated PIWI domain containing phage-defense protein
Urease accessory protein UreE
Rdx family selenoprotein
Transposase and inactivated derivatives, IS5 family
Prophage maintenance system killer protein
Phage or plasmid associated DNA primase
Primase-polymerase (primpol) domain
Uncharacterized conserved membrane protein
Zn finger protein
Uncharacterized conserved protein
Sulphur oxygenase/reductase
Uncharacterized conserved protein
Predicted redox protein, regulator of disulfide bond formation
Uncharacterized conserved memrane protein
Uncharacterized protein, possibly involved in utilization of glycolate and propanediol
Uncharacterized conserved protein
Uncharacterized conserved protein
Uncharacterized conserved protein
Uncharacterized conserved protein
Uncharacterized conserved protein
Uncharacterized conserved protein
Uncharacterized conserved protein
Uncharacterized conserved protein
Predicted solute binding protein
Uncharacterized conserved protein
Polysaccharide biosynthesis protein, Mvin family
Uncharacterized conserved protein
Uncharacterized conserved protein
PIN domain
Uncharacterized conserved protein

Uncharacterized conserved protein
Uncharacterized conserved protein
Uncharacterized conserved protein
Uncharacterized conserved protein
Predicted transcriptional regulator, PadR family
Uncharacterized conserved protein
Uncharacterized conserved protein
Uncharacterized conserved protein
Uncharacterized conserved protein
Uncharacterized conserved membrane protein
Uncharacterized conserved protein
Predicted membrane-bound metal-dependent hydrolase, DUF457
Lysophospholipase, alpha-beta hydrolase superfamily
Uncharacterized conserved protein
Uncharacterized conserved protein
Uncharacterized conserved protein
Uncharacterized conserved protein
Uncharacterized conserved protein
Uncharacterized conserved protein
Uncharacterized conserved protein
Uncharacterized conserved protein
Uncharacterized conserved protein
Uncharacterized conserved protein
Uncharacterized conserved protein
Uncharacterized conserved protein
Uncharacterized conserved protein
Zn-finger protein
Uncharacterized conserved protein
Uncharacterized conserved protein
Uncharacterized conserved protein
Uncharacterized conserved protein
Heavy-metal-associated domain
Uncharacterized conserved protein
Uncharacterized conserved protein
Uncharacterized conserved protein
Uncharacterized conserved protein
Phenylacetic acid-responsive transcriptional repressor
Uncharacterized conserved protein
RHH/CopG DNA binding protein
Uncharacterized protein contains PIN domain
Uncharacterized conserved protein
Uncharacterized conserved protein
Glycosyltransferase
Uncharacterized conserved protein
Uncharacterized conserved protein
Uncharacterized conserved protein
Uncharacterized conserved protein
PIN domain

Uncharacterized conserved protein
Uncharacterized conserved protein
Predicted transcriptional regulator
Uncharacterized conserved protein
Uncharacterized conserved protein
Uncharacterized conserved protein
Uncharacterized conserved protein
CRISPR-associated protein
Uncharacterized conserved protein
Uncharacterized conserved protein
Uncharacterized conserved protein
L-aminopeptidase/D-esterase
Uncharacterized conserved protein
Thermostable phosphotriesterase, metal-dependent hydrolase with the TIM-barrel fold
Predicted membrane protein with C-terminal Zn ribbon domain
Uncharacterized conserved protein
Cu2+-containing amine oxidase
Uncharacterized conserved protein
Uncharacterized conserved protein
Uncharacterized conserved protein
S-layer protein SlaB
Predicted periplasmic protein
Uncharacterized conserved protein
Uncharacterized conserved protein
Pilin/Flagellin
SSV1_ORF_D-335, predicted GIY-YIG nuclease superfamily protein
Uncharacterized conserved membrane protein
Uncharacterized conserved protein
Uncharacterized conserved protein
TPR repeats containing protein
Uncharacterized conserved protein
Uncharacterized conserved protein
Uncharacterized conserved protein
Archaeal/vacuolar-type H+-ATPase subunit H
Uncharacterized conserved protein
Uncharacterized conserved protein
Uncharacterized conserved protein
Uncharacterized conserved protein
Uncharacterized conserved protein
Uncharacterized conserved protein
Minimal nucleotidyltransferase
ATPase involved in chromosome partitioning, ParA family
Uncharacterized conserved protein
Uncharacterized conserved protein
Uncharacterized conserved protein
CRISPR associated protein
Uncharacterized conserved protein
CRISPR-associated protein CsaX

Uncharacterized conserved protein associated with DNA polymerase II
Uncharacterized conserved protein
Transposase
Uncharacterized conserved protein
Uncharacterized conserved protein
Uncharacterized conserved protein
Uncharacterized conserved protein
Plasmid regulatory DNA-binding protein
Uncharacterized conserved protein
Predicted transcriptional regulator, HTH domain
NurA-like nuclease
ATPase, uncharacterized
Uncharacterized conserved protein
Phospholipase D/Transphosphatidylase
Uncharacterized conserved protein
Uncharacterized conserved protein
Uncharacterized conserved protein
Uncharacterized conserved protein
Fe-S oxidoreductase
Uncharacterized conserved protein
Uncharacterized conserved protein
Uncharacterized conserved protein
Uncharacterized conserved protein
Uncharacterized conserved protein
Uncharacterized conserved protein
Uncharacterized conserved protein
Uncharacterized conserved protein
Uncharacterized conserved protein
Uncharacterized conserved protein
Uncharacterized conserved protein
Uncharacterized conserved protein
SAM-dependent methyltransferase
Beta-galactosidase/beta-glucuronidase
Alpha-L-fucosidase
DsrE/DsrF-like family
Uncharacterized conserved protein
Uncharacterized conserved protein
Uncharacterized conserved protein
Uncharacterized conserved protein
Uncharacterized conserved protein
Acetyltransferase, RimL family
Uncharacterized conserved membrane protein
O-Glycosyl hydrolase family 16
Uncharacterized conserved protein
Uncharacterized conserved protein
Type IV secretory pathway, VirB4 component
Zn-dependent protease
Uncharacterized conserved protein

Uncharacterized conserved protein
Predicted metal-binding protein
PIN domain containing protein
Uncharacterized conserved protein
Uncharacterized conserved protein
SWIM Zn-finger
Uncharacterized conserved protein
Uncharacterized conserved protein
Radical SAM superfamily enzyme
Uncharacterized conserved protein
Uncharacterized conserved protein
Uncharacterized conserved protein
RHH/CopG DNA binding protein
Uncharacterized conserved protein
Uncharacterized conserved protein
Uncharacterized conserved protein
ABC-type sugar transport system, periplasmic component, contains N-terminal xre family HTH domain
DEDD family 3'-5' exonuclease
Uncharacterized conserved protein
Transposase
Uncharacterized conserved protein
Uncharacterized conserved protein
HNH superfamily endonuclease
TOPRIM domain containing protein, predicted phage defense system
Phenylpropionate dioxygenase or related ring-hydroxylating dioxygenase, large terminal subunit
PKD domain containing protein
CRISPR system related protein, RAMP superfamily Cas7 group
Uncharacterized conserved protein
Uncharacterized conserved protein
Ribosome associated protein L45a
Uncharacterized conserved protein
Uncharacterized conserved protein
Predicted regulatory ATPase
Uncharacterized conserved protein
Transposase ISC1217
Transposase ISC1234
Transposon ISC1160
Zinc finger C2H2-type
Uncharacterized protein
Chromosome partitioning protein ParB
HTH containing transcriptional regulator
Uncharacterized protein
Uncharacterized TIM barrel protein
ATPase (AAA+ superfamily)
Uncharacterized protein
Uncharacterized protein
TRASH domain-containing protein, metal-binding
PIN domain containing protein

Predicted membrane protein, DoxX family
Zn zinger protein
Uncharacterized conserved protein
Antitoxin MazE superfamily protein
Uncharacterized conserved protein
Uncharacterized conserved protein
Predicted transcriptional regulator, HTH
Uncharacterized conserved protein
Uncharacterized conserved protein
Uncharacterized conserved protein
Uncharacterized conserved protein
Uncharacterized conserved protein
Uncharacterized conserved protein
Uncharacterized conserved protein
Uncharacterized conserved protein
Uncharacterized conserved protein
Uncharacterized conserved protein
Uncharacterized conserved protein
Uncharacterized conserved protein
Uncharacterized conserved protein
Uncharacterized conserved protein
Uncharacterized conserved protein
Zn finger protein
Uncharacterized conserved protein
Uncharacterized conserved protein
Uncharacterized conserved protein
Uncharacterized conserved protein
Uncharacterized conserved protein
Uncharacterized conserved protein
Uncharacterized conserved protein
Uncharacterized conserved protein
Uncharacterized conserved protein
Uncharacterized conserved protein
Predicted redox protein, regulator of disulfide bond formation
Ribosome associated protein L47a
Uncharacterized conserved protein
Uncharacterized conserved protein
Uncharacterized conserved protein
Uncharacterized conserved protein
Uncharacterized conserved protein
Uncharacterized conserved protein
Uncharacterized conserved protein
Uncharacterized conserved protein
Uncharacterized conserved protein
Uncharacterized conserved protein
Uncharacterized conserved protein
CopG/RHH family DNA binding protein
Methane/Phenol/Toluene Hydroxylase component, ferritin superfamily

MmoB/DmpM family, monooxygenase component
Methane/Phenol/Toluene Hydroxylase component
Methane/Phenol/Toluene Hydroxylase component, ferritin superfamily
Uncharacterized conserved protein
Uncharacterized conserved protein
Uncharacterized conserved protein
CopG/RHH family DNA binding protein
Uncharacterized conserved protein
Uncharacterized conserved protein
Uncharacterized conserved protein
Uncharacterized conserved protein
Transposase DNA-binding domain
Uncharacterized conserved protein
Uncharacterized conserved protein
Uncharacterized conserved protein
Uncharacterized conserved protein
Uncharacterized conserved protein
Uncharacterized conserved protein
Uncharacterized conserved protein
Uncharacterized conserved protein
Small subunit of phenylpropionate dioxygenase
Uncharacterized conserved protein
Uncharacterized conserved protein
Uncharacterized conserved protein
Uncharacterized conserved protein
Uncharacterized conserved protein
Uncharacterized conserved protein
Uncharacterized conserved protein
Uncharacterized conserved protein
Uncharacterized conserved protein, DUF904
Uncharacterized conserved protein
Uncharacterized conserved protein
Uncharacterized conserved protein
Uncharacterized conserved protein
Uncharacterized conserved protein
Uncharacterized conserved protein
Uncharacterized conserved protein
Uncharacterized conserved protein
Uncharacterized conserved protein
Uncharacterized conserved protein
Uncharacterized conserved protein
Uncharacterized conserved protein
Uncharacterized conserved protein
Uncharacterized conserved protein
Uncharacterized conserved protein
Uncharacterized conserved protein
Uncharacterized conserved protein
Uncharacterized conserved protein

Uncharacterized conserved protein
Uncharacterized conserved protein
Uncharacterized conserved protein
Uncharacterized conserved protein
Uncharacterized conserved protein
Uncharacterized conserved protein
Uncharacterized conserved protein
Uncharacterized conserved protein
Uncharacterized conserved protein
Uncharacterized conserved protein
Uncharacterized conserved protein
Uncharacterized conserved protein
Uncharacterized conserved protein
Uncharacterized conserved protein
Virus associated protein, V1 family
Sulfolobus virus associated uncharacterized membrane protein
Viral/Archaeal nuclease
Uncharacterized conserved protein
Uncharacterized conserved protein
Uncharacterized conserved protein
Uncharacterized conserved protein
Uncharacterized conserved protein
Uncharacterized conserved protein associated with transposon
CopG/RHH family DNA binding protein
Transcriptional regulator, GntR family
Uncharacterized conserved protein
Uncharacterized conserved membrane protein
Uncharacterized conserved protein
Uncharacterized conserved protein
Uncharacterized conserved protein
Uncharacterized conserved protein
Uncharacterized conserved protein
Uncharacterized conserved protein
Uncharacterized conserved protein
Uncharacterized conserved protein
Uncharacterized conserved protein
Uncharacterized conserved protein
Uncharacterized conserved protein
Uncharacterized conserved protein
Uncharacterized conserved protein
Uncharacterized conserved protein
Uncharacterized conserved protein
Uncharacterized conserved protein
Predicted transcriptional regulator, contains ATP-binding and HTH domains
Uncharacterized conserved protein
Uncharacterized conserved protein
Uncharacterized conserved protein
Uncharacterized conserved protein

SWIM Zn-finger
Uncharacterized conserved protein
Uncharacterized conserved protein
Uncharacterized conserved protein
Uncharacterized conserved protein
Uncharacterized conserved protein
Uncharacterized conserved protein
Uncharacterized conserved protein
Uncharacterized conserved protein
Uncharacterized conserved protein
Uncharacterized conserved protein
Uncharacterized conserved protein
Uncharacterized conserved protein
Uncharacterized conserved protein
Uncharacterized conserved protein
Uncharacterized conserved protein
Uncharacterized conserved protein
Uncharacterized conserved protein
Uncharacterized conserved protein
Uncharacterized conserved protein
Uncharacterized conserved protein
Uncharacterized conserved protein
Tranposon-associated protein
Uncharacterized conserved protein
Uncharacterized conserved protein
Uncharacterized conserved protein
Uncharacterized conserved protein
Uncharacterized conserved protein
Uncharacterized conserved protein
Uncharacterized conserved protein
Uncharacterized conserved protein
Uncharacterized conserved protein
Uncharacterized conserved protein
Uncharacterized conserved protein
Uncharacterized conserved protein
Uncharacterized conserved protein
Uncharacterized conserved protein
Uncharacterized conserved protein
Uncharacterized conserved protein
Uncharacterized conserved protein
Uncharacterized conserved protein
Uncharacterized conserved protein
Uncharacterized conserved protein
Uncharacterized conserved protein
Uncharacterized conserved protein
Uncharacterized conserved protein
Uncharacterized conserved protein
Uncharacterized conserved protein
Uncharacterized conserved protein

Uncharacterized conserved protein

Permease of the major facilitator superfamily
A/G-specific DNA glycosylase
DNA replication initiation complex subunit, GINS23 family
Acetyltransferase (GNAT) family
Radical SAM superfamily enzyme
Radical SAM superfamily enzyme
Alpha/beta-knot SAM-dependent RNA methyltransferase
Hydrogenase maturation factor
Integrase
GDP-D-mannose dehydratase
RecB-like nuclease fused to coiled-coil domain
Ni,Fe-hydrogenase I large subunit
Kef-type K+ transport system, membrane component fused TrkA K+ transport system
S-layer domain
Ni,Fe-hydrogenase I small subunit
Type I restriction-modification system methyltransferase subunit
Endonuclease, HJR/Mrr/RecB family
Glycosyltransferase
Predicted membrane protein
S-layer domain
Hydrogenase maturation factor
Hydrogenase maturation factor
AIG2-like family protein
RecA-superfamily ATPase implicated in signal transduction
Membrane-bound NiFe hydrogenase subunit
Membrane-bound NiFe hydrogenase subunit
Uncharacterized conserved protein
SAM-dependent methyltransferase
Predicted ester cyclase
Uncharacterized conserved protein
Uncharacterized conserved protein
Uncharacterized conserved protein
Uncharacterized conserved protein
Alpha/beta superfamily hydrolase
Uncharacterized conserved protein
Uncharacterized conserved protein
Uncharacterized conserved protein
Uncharacterized conserved protein
Uncharacterized conserved protein
