# Supplementary material for: Insights into archaeal evolution and symbiosis from the genomes of a nanoarchaeon and its inferred crenarchaeal host from Obsidian Pool, Yellowstone National Park
Source: Biol Direct. 2013 Apr 22;8:9. doi: 10.1186/1745-6150-8-9 (PMC3655853; doi:10.1186/1745-6150-8-9)
Supplement: Additional file 6 — Lists the arCOGs present in both N. equitans and Nst1. [file 1745-6150-8-9-S6.pdf]

Additional file 6. The 283 arCOGs present in both Nanoarchaeaota

arCOG	COG	Gene	Function code (as in COGs)	Annotation	Acd1	# genomes in Crenarchaeaota (35 max)	# genomes in Euryarchaeaota (78 max)
arCOG00349	COG01141	Fer	C	Ferredoxin	**0**	**19**	**42**
arCOG01711	COG00221	Ppa	C	Inorganic pyrophosphatase	**1**	**27**	**47**
arCOG02201	COG00206	FtsZ	D	Cell division GTPase	**0**	**0**	**76**
arCOG02263	COG02450	-	D	Predicted cell division protein, SepF homolog	**0**	**0**	**76**
arCOG01924	COG00252	AnsB	E	L-asparaginase/archaeal Glu-tRNAGln amidotransferase subunit D	**1**	**35**	**78**
arCOG01700	COG00010	SpeB	E	Arginase family enzyme	**1**	**35**	**75**
arCOG01107	COG00624	ArgE	E	Acetylornithine deacetylase/Succinyl-diaminopimelate desuccinylase or related deacylase	**1**	**35**	**62**
arCOG00255	COG00077	PheA	E	Prephenate dehydratase	**1**	**26**	**65**
arCOG04048	COG00717	Dcd	F	Deoxycytidine deaminase	**1**	**35**	**78**
arCOG00419	COG00537	Hit	F	HIT family hydrolase	**1**	**35**	**71**
arCOG01038	COG01936	-	F	Predicted nucleotide kinase (CMP/AMP kinase related)	**1**	**33**	**78**
arCOG04313	COG00105	Ndk	F	Nucleoside diphosphate kinase	**1**	**30**	**78**
arCOG01039	COG02019	AdkA	F	Archaeal adenylate kinase	**1**	**28**	**48**
arCOG01111	COG00574	PpsA	G	Phosphoenolpyruvate synthase/pyruvate phosphate dikinase	**1**	**34**	**78**
arCOG01676	COG00476	ThiF	H	Dinucleotide-utilizing enzyme involved in molybdopterin and thiamine biosynthesis	**1**	**35**	**68**
arCOG00535	COG02104	ThiS	H	Sulfur transfer protein involved in thiamine biosynthesis	**1**	**14**	**64**
arCOG00807	COG00060	IleS	J	Isoleucyl-tRNA synthetase	**1**	**35**	**78**
arCOG01719	COG02511	GatE	J	Archaeal Glu-tRNAGln amidotransferase subunit E (contains GAD domain)	**1**	**35**	**78**
arCOG01701	COG01676	SEN2	J	tRNA splicing endonuclease	**1**	**35**	**78**
arCOG00810	COG00143	MetG	J	Methionyl-tRNA synthetase	**1**	**35**	**78**
arCOG01255	COG00013	AlaS	J	Alanyl-tRNA synthetase	**1**	**35**	**78**
arCOG00042	COG00037	MesJ	J	tRNA(Ile)-lysidine synthase MesJ	**1**	**35**	**77**
arCOG00809	COG00495	LeuS	J	Leucyl-tRNA synthetase	**1**	**35**	**71**
arCOG00973	COG00144	Sun	J	tRNA or rRNA cytosine-C5-methylase	**1**	**35**	**54**
arCOG00357	COG00012	-	J	Predicted GTPase, probable translation factor	**1**	**35**	**78**
arCOG00402	COG00442	ProS	J	Prolyl-tRNA synthetase	**1**	**35**	**78**
arCOG00405	COG00423	GRS1	J	Glycyl-tRNA synthetase (class II)	**1**	**35**	**78**
arCOG01887	COG00180	TrpS	J	Tryptophanyl-tRNA synthetase	**1**	**35**	**78**
arCOG04112	COG01736	DPH2	J	Diphthamide synthase subunit DPH2	**1**	**35**	**78**
arCOG00401	COG00441	ThrS	J	Threonyl-tRNA synthetase	**1**	**35**	**78**
arCOG00033	COG02520	Trm5	J	Wybutosine (yW) biosynthesis enzyme, Trm5 methyltransferase	**1**	**35**	**78**
arCOG00078	COG01889	NOP1	J	Fibrillarin-like rRNA methylase	**1**	**35**	**78**
arCOG00403	COG00172	SerS	J	Seryl-tRNA synthetase	**1**	**35**	**78**
arCOG00404	COG00124	HisS	J	Histidyl-tRNA synthetase	**1**	**35**	**78**
arCOG00406	COG00017	AsnS	J	Aspartyl/asparaginyl-tRNA synthetase	**1**	**35**	**78**
arCOG00410	COG00016	PheS	J	Phenylalanyl-tRNA synthetase alpha subunit	**1**	**35**	**78**
arCOG00412	COG00072	PheT	J	Phenylalanyl-tRNA synthetase beta subunit	**1**	**35**	**78**
arCOG00487	COG00018	ArgS	J	Arginyl-tRNA synthetase	**1**	**35**	**78**
arCOG00779	COG00200	RplO	J	Ribosomal protein L15	**1**	**35**	**78**
arCOG00780	COG01727	RPL18A	J	Ribosomal protein L18E	**1**	**35**	**78**
arCOG00782	COG00199	RpsN	J	Ribosomal protein S14	**1**	**35**	**78**
arCOG00808	COG00525	ValS	J	Valyl-tRNA synthetase	**1**	**35**	**78**
arCOG01001	COG00024	Map	J	Methionine aminopeptidase	**1**	**35**	**78**
arCOG01179	COG00361	InfA	J	Translation initiation factor 1 (IF-1)	**1**	**35**	**78**
arCOG01219	COG01867	TRM1	J	N2,N2-dimethylguanosine tRNA methyltransferase	**1**	**35**	**78**
arCOG01344	COG02238	RPS19A	J	Ribosomal protein S19E (S16A)	**1**	**35**	**78**
arCOG01559	COG00480	FusA	J	Translation elongation factor G, EF-G (GTPase)	**1**	**35**	**78**
arCOG01560	COG00532	InfB	J	Translation initiation factor 2 (IF-2; GTPase)	**1**	**35**	**78**
arCOG01561	COG05256	TEF1	J	Translation elongation factor EF-1alpha (GTPase)	**1**	**35**	**78**
arCOG01563	COG05257	GCD11	J	Translation initiation factor 2, gamma subunit (eIF-2gamma; GTPase)	**1**	**35**	**78**
arCOG01640	COG01601	GCD7	J	Translation initiation factor 2, beta subunit (eIF-2beta)/eIF-5 N-terminal domain	**1**	**35**	**78**
arCOG01722	COG00099	RpsM	J	Ribosomal protein S13	**1**	**35**	**78**
arCOG01741	COG01537	PelA	J	Release factor eRF1	**1**	**35**	**78**
arCOG01751	COG01358	RPL8A	J	Ribosomal protein L7AE	**1**	**35**	**78**
arCOG01758	COG00051	RpsJ	J	Ribosomal protein S10	**1**	**35**	**78**
arCOG01885	COG01383	RPS17A	J	Ribosomal protein S17E	**1**	**35**	**78**
arCOG01886	COG00162	TyrS	J	Tyrosyl-tRNA synthetase	**1**	**35**	**78**
arCOG01923	COG01498	SIK1	J	Protein implicated in ribosomal biogenesis, Nop56p homolog	**1**	**35**	**78**
arCOG01946	COG02125	RPS6A	J	Ribosomal protein S6E (S10)	**1**	**35**	**78**
arCOG01988	COG02092	EFB1	J	Translation elongation factor EF-1beta	**1**	**35**	**78**
arCOG04067	COG00090	RplB	J	Ribosomal protein L2	**1**	**35**	**78**
arCOG04070	COG00087	RplC	J	Ribosomal protein L3	**1**	**35**	**78**
arCOG04071	COG00088	RplD	J	Ribosomal protein L4	**1**	**35**	**78**
arCOG04072	COG00089	RplW	J	Ribosomal protein L23	**1**	**35**	**78**
arCOG04086	COG01841	RpmD	J	Ribosomal protein L30	**1**	**35**	**78**
arCOG04087	COG00098	RpsE	J	Ribosomal protein S5	**1**	**35**	**78**
arCOG04088	COG00256	RplR	J	Ribosomal protein L18	**1**	**35**	**78**
arCOG04089	COG02147	RPL19A	J	Ribosomal protein L19E	**1**	**35**	**78**
arCOG04090	COG00097	RplF	J	Ribosomal protein L6P	**1**	**35**	**78**
arCOG04091	COG00096	RpsH	J	Ribosomal protein S8	**1**	**35**	**78**
arCOG04092	COG00094	RplE	J	Ribosomal protein L5	**1**	**35**	**78**
arCOG04093	COG01471	RPS4A	J	Ribosomal protein S4E	**1**	**35**	**78**

arCOG04094	COG00198	RplX	J	Ribosomal protein L24	**1**	**35**	**78**
arCOG04095	COG00093	RplN	J	Ribosomal protein L14	**1**	**35**	**78**
arCOG04096	COG00186	RpsQ	J	Ribosomal protein S17	**1**	**35**	**78**
arCOG04097	COG00092	RpsC	J	Ribosomal protein S3	**1**	**35**	**78**
arCOG04098	COG00091	RplV	J	Ribosomal protein L22	**1**	**35**	**78**
arCOG04099	COG00185	RpsS	J	Ribosomal protein S19	**1**	**35**	**78**
arCOG04107	COG01093	SUI2	J	Translation initiation factor 2, alpha subunit (eIF-2alpha)	**1**	**35**	**78**
arCOG04109	COG01631	RPL42A	J	Ribosomal protein L44E	**1**	**35**	**78**
arCOG04113	COG00197	RplP	J	Ribosomal protein L10AE/L16	**1**	**35**	**78**
arCOG04129	COG02139	RPL21A	J	Ribosomal protein L21E	**1**	**35**	**78**
arCOG04182	COG02004	RPS24A	J	Ribosomal protein S24E	**1**	**35**	**78**
arCOG04185	COG00184	RpsO	J	Ribosomal protein S15P	**1**	**35**	**78**
arCOG04208	COG01997	RPL43A	J	Ribosomal protein L37AE/L43A	**1**	**35**	**78**
arCOG04223	COG00023	SUI1	J	Translation initiation factor 1 (eIF-1/SUI1)	**1**	**35**	**78**
arCOG04228	COG01990	pth2	J	Peptidyl-tRNA hydrolase	**1**	**35**	**78**
arCOG04239	COG00522	RpsD	J	Ribosomal protein S4 or related protein	**1**	**35**	**78**
arCOG04242	COG00102	RplM	J	Ribosomal protein L13	**1**	**35**	**78**
arCOG04243	COG00103	RpsI	J	Ribosomal protein S9	**1**	**35**	**78**
arCOG04245	COG00052	RpsB	J	Ribosomal protein S2	**1**	**35**	**78**
arCOG04249	COG01746	CCA1	J	tRNA nucleotidyltransferase (CCA-adding enzyme)	**1**	**35**	**78**
arCOG04254	COG00049	RpsG	J	Ribosomal protein S7	**1**	**35**	**78**
arCOG04255	COG00048	RpsL	J	Ribosomal protein S12	**1**	**35**	**78**
arCOG04287	COG02058	RPP1A	J	Ribosomal protein L12E/L44/L45/RPP1/RPP2	**1**	**35**	**78**
arCOG04288	COG00244	RplJ	J	Ribosomal protein L10	**1**	**35**	**78**
arCOG04289	COG00081	RplA	J	Ribosomal protein L1	**1**	**35**	**78**
arCOG04302	COG00008	GlnS	J	Glutamyl- or glutaminyl-tRNA synthetase	**1**	**35**	**78**
arCOG04314	COG02053	RPS28A	J	Ribosomal protein S28E/S33	**1**	**35**	**78**
arCOG04372	COG00080	RplK	J	Ribosomal protein L11	**1**	**35**	**78**
arCOG00781	COG01717	RPL32	J	Ribosomal protein L32E	**1**	**35**	**78**
arCOG01950	COG02075	RPL24A	J	Ribosomal protein L24E	**1**	**35**	**78**
arCOG04049	COG01552	RPL40A	J	Ribosomal protein L40E	**1**	**35**	**78**
arCOG04186	COG01890	RPS1A	J	Ribosomal protein S3AE	**1**	**35**	**78**
arCOG04473	COG02097	RPL31A	J	Ribosomal protein L31E	**1**	**35**	**78**
arCOG04177	COG02167	RPL39	J	Ribosomal protein L39E	**1**	**35**	**78**
arCOG04126	COG02126	RPL37A	J	Ribosomal protein L37E	**1**	**35**	**78**
arCOG01742	COG01503	eRF1	J	Peptide chain release factor 1 (eRF1)	**1**	**35**	**78**
arCOG04161	COG01798	DPH5	J	Diphthamide biosynthesis methyltransferase	**1**	**35**	**78**
arCOG04149	COG01499	NMD3	J	NMD protein affecting ribosome stability and mRNA decay	**1**	**35**	**78**
arCOG00109	COG02890	HemK	J	Methylase of polypeptide chain release factors	**1**	**35**	**77**
arCOG00985	COG02016	-	J	Predicted RNA-binding protein (contains PUA domain)	**1**	**35**	**77**
arCOG00989	COG00343	Tgt	J	Queuine/archaeosine tRNA-ribosyltransferase	**1**	**35**	**77**
arCOG01358	COG00621	MiaB	J	2-methylthioadenine synthetase	**1**	**35**	**77**
arCOG04130	COG01491	-	J	Predicted RNA-binding protein	**1**	**35**	**77**
arCOG04176	COG01976	TIF6	J	Translation initiation factor 6 (eIF-6)	**1**	**35**	**77**
arCOG04187	COG01500	-	J	Predicted exosome subunit	**1**	**35**	**77**
arCOG00991	COG01370	-	J	tRNA modification protein, contains pre-PUA and PUA domains	**1**	**35**	**77**
arCOG00906	COG02260	-	J	Predicted Zn-ribbon RNA-binding protein	**1**	**35**	**77**
arCOG01989	COG02888	-	J	Predicted Zn-ribbon RNA-binding protein with a function in translation	**1**	**35**	**76**
arCOG01736	COG01514	LigT	J	2'-5' RNA ligase	**1**	**35**	**75**
arCOG00978	COG02519	GCD14	J	tRNA(1-methyladenosine) methyltransferase	**1**	**35**	**73**
arCOG04174	COG00731	TYW3	J	Wybutosine (yW) biosynthesis enzyme, Fe-S oxidoreductase	**1**	**35**	**73**
arCOG01042	COG01325	-	J	Exosome subunit, RNA binding protein with dsRBD fold	**1**	**35**	**68**
arCOG04175	COG02157	RPL20A	J	Ribosomal protein L20A (L18A)	**1**	**35**	**67**
arCOG00486	COG00215	CysS	J	Cysteinyl-tRNA synthetase	**1**	**35**	**62**
arCOG01752	COG01911	RPL30	J	Ribosomal protein L30E	**1**	**35**	**58**
arCOG04318	COG02178	-	J	Predicted RNA-binding protein of the translin family	**1**	**35**	**43**
arCOG01574	COG02123	-	J	RNase PH-related exoribonuclease	**1**	**35**	**41**
arCOG01575	COG00689	Rph	J	Ribonuclease PH	**1**	**35**	**40**
arCOG04167	COG02163	RPL14A	J	Ribosomal protein L14E/L6E/L27E	**1**	**35**	**36**
arCOG04305	COG04830	RPS26B	J	Ribosomal protein S26	**1**	**35**	**3**
arCOG04246	COG01690	RtcB	J	RNA 3'-P ligase, RtcB family protein	**1**	**34**	**78**
arCOG00785	COG00255	RpmC	J	Ribosomal protein L29	**1**	**34**	**78**
arCOG00678	COG01097	RRP4	J	RNA-binding protein Rrp4 or related protein (contain S1 domain and KH domain)	**1**	**34**	**39**
arCOG04252	COG00585	-	J	tRNA pseudouridine synthase D	**1**	**33**	**78**
arCOG00038	COG00301	ThiI	J	tRNA S(4)U 4-thiouridine synthase	**1**	**33**	**76**
arCOG04183	COG01998	RPS31	J	Ribosomal protein S27AE	**1**	**33**	**73**
arCOG04156	COG01590	TYW3	J	Wybutosine (yW) biosynthesis enzyme	**1**	**33**	**33**
arCOG04168	COG02174	RPL34A	J	Ribosomal protein L34E	**0**	**32**	**36**
arCOG00987	COG00130	TruB	J	Pseudouridine synthase	**1**	**30**	**78**
arCOG00967	COG02419	-	J	tRNA m(1)G methyltransferase	**1**	**29**	**17**
arCOG01018	COG00565	LasT	J	rRNA methylase	**1**	**25**	**72**
arCOG04304	COG02451	-	J	Ribosomal protein L35AE/L33A	**0**	**10**	**13**
arCOG04449	COG00101	TruA	J	Pseudouridylate synthase	**0**	**9**	**77**
arCOG00546	COG00595	-	J	mRNA degradation ribonuclease J1/J2 (metallo-beta-lactamase superfamily)	**0**	**0**	**69**
arCOG02285	COG03270		J	Ribosome biogenesis protein, NOL1/NOP2/fmu family	**0**	**0**	**15**
arCOG00122	COG02265	TrmA	J	SAM-dependent methyltransferase related to tRNA (uracil-5-)-methyltransferase	**0**	**0**	**12**
arCOG06624			J	Ribosomal protein L41E	**0**	**0**	**10**

arCOG01695	COG01293	-	K	Predicted RNA-binding protein homologous to eukaryotic snRNP	**1**	**35**	**78**
arCOG01762	COG00085	RpoB/Rpo2	K	DNA-directed RNA polymerase subunit B	**1**	**35**	**78**
arCOG04241	COG00202	RpoA/Rpo1	K	DNA-directed RNA polymerase subunit D	**1**	**35**	**78**
arCOG01981	COG01405	SUA7	K	Transcription initiation factor TFIIIB, Brf1 subunit/Transcription initiation factor TFIIB	**1**	**35**	**78**
arCOG00675	COG01095	RPB7	K	DNA-directed RNA polymerase, subunit E'	**1**	**35**	**78**
arCOG01016	COG01460	-	K	DNA-directed RNA polymerase, subunit F (rpoF)	**1**	**35**	**78**
arCOG01268	COG01758	RpoZ	K	DNA-directed RNA polymerase, subunit K/omega	**1**	**35**	**78**
arCOG01920	COG00250	NusG	K	Transcription antiterminator NusG	**1**	**35**	**78**
arCOG04244	COG01644	RPB10	K	DNA-directed RNA polymerase, subunit N (RpoN/RPB10)	**1**	**35**	**78**
arCOG04256	COG00086	RpoC/Rpo11	K	DNA-directed RNA polymerase subunit A''	**1**	**35**	**78**
arCOG04257	COG00086	RpoC/Rpo3	K	DNA-directed RNA polymerase subunit A'	**1**	**35**	**78**
arCOG04111	COG01761	RPB11	K	DNA-directed RNA polymerase, subunit L	**1**	**35**	**78**
arCOG04341	COG01996	RPC10	K	DNA-directed RNA polymerase, subunit RPC10 (contains C4-type Zn-finger)	**1**	**35**	**78**
arCOG01863	COG01813	-	K	Predicted transcription factor, homolog of eukaryotic MBF1	**1**	**35**	**78**
arCOG01580	COG01522	Lrp	K	Transcriptional regulator, IclR family	**1**	**35**	**77**
arCOG00579	COG01594	RPB9	K	DNA-directed RNA polymerase, subunit M/Transcription elongation factor TFIIS	**1**	**35**	**77**
arCOG04258	COG02012	RPB5	K	DNA-directed RNA polymerase, subunit H, RpoH/RPB5	**1**	**35**	**77**
arCOG01764	COG02101	SPT15	K	TATA-box binding protein (TBP), component of TFIID and TFIIIB	**1**	**35**	**74**
arCOG04270	COG01675	TFA1	K	Transcription initiation factor IIE, alpha subunit	**1**	**35**	**73**
arCOG01753	COG01581	Ssh10b	K	Archaeal DNA-binding protein	**1**	**35**	**53**
arCOG04077	COG02093	Spt4	K	Transcription elongation factor Spt4/RpoE2, zinc finger protein	**1**	**32**	**78**
arCOG02037	COG01378	-	K	Sugar-specific transcriptional regulator TrmB	**1**	**25**	**61**
arCOG00921	COG01318	-	K	Predicted transcriptional regulator	**0**	**19**	**22**
arCOG01526	COG01110	-	L	Reverse gyrase	**1**	**35**	**28**
arCOG02144	COG02036	HHT1	L	Histones H3 and H4	**0**	**4**	**73**
arCOG01527	COG00550	TopA	L	Topoisomerase IA	**1**	**35**	**78**
arCOG01183	COG00533	Kae1/QRI7	L	Subunit of KEOPS complex, contains a domain with ASKHA fold and RIO-type kinase (AP-endonuclease activity)	**1**	**35**	**78**
arCOG01347	COG01793	CDC9	L	ATP-dependent DNA ligase	**1**	**35**	**76**
arCOG00891	COG00084	TatD	L	Mg-dependent DNase	**1**	**34**	**46**
arCOG00488	COG00592	DnaN	L	DNA polymerase sliding clamp subunit (PCNA homolog)	**1**	**35**	**78**
arCOG00328	COG00417	PolB	L	DNA polymerase elongation subunit (family B)	**1**	**35**	**78**
arCOG00439	COG01241	MCM2	L	Predicted ATPase involved in replication control, Cdc46/Mcm family	**1**	**35**	**78**
arCOG00469	COG00470	HolB	L	ATPase involved in DNA replication HolB, small subunit	**1**	**35**	**78**
arCOG00470	COG00470	HolB	L	ATPase involved in DNA replication HolB, large subunit	**1**	**35**	**78**
arCOG00551	COG01711	-	L	DNA replication initiation complex subunit, GINS15 family	**1**	**35**	**78**
arCOG03013	COG02219	PRI2	L	Eukaryotic-type DNA primase, large subunit	**1**	**35**	**78**
arCOG04121	COG00164	RnhB	L	Ribonuclease HII	**1**	**35**	**78**
arCOG04281	COG00358	DnaG	L	DNA primase (bacterial type)	**1**	**35**	**78**
arCOG04050	COG00258	Exo	L	5'-3' exonuclease (including N-terminal domain of PolI)	**1**	**35**	**78**
arCOG00280	COG00433	-	L	HerA helicase	**1**	**35**	**77**
arCOG00397	COG00420	SbcD	L	DNA repair exonuclease, SbcD	**1**	**35**	**77**
arCOG01165	COG01389	-	L	DNA topoisomerase VI, subunit B	**1**	**35**	**74**
arCOG04143	COG01697	-	L	DNA topoisomerase VI, subunit A	**1**	**35**	**74**
arCOG00928	COG01628	-	L	Endonuclease V homolog	**1**	**35**	**71**
arCOG00467	COG01474	CDC6	L	Cdc6-related protein, AAA superfamily ATPase	**1**	**35**	**62**
arCOG00919	COG01591	-	L	Holliday junction resolvase	**1**	**35**	**61**
arCOG00905	COG01573	-	L	Uracil-DNA glycosylase	**1**	**35**	**47**
arCOG00368	COG00419	SbcC	L	ATPase involved in DNA repair, SbcC	**1**	**34**	**77**
arCOG01894	COG00648	Nfo	L	Endonuclease IV	**1**	**34**	**76**
arCOG00284	COG00433	-	L	HerA helicase	**1**	**27**	**7**
arCOG01510	COG01599	RFA1	L	Single-stranded DNA-binding replication protein A (RPA), large (70 kD) subunit or related ssDNA-binding protein	**1**	**24**	**78**
arCOG04144	COG04047	-	L	8-oxoguanine DNA glycosylase	**0**	**22**	**24**
arCOG00048	COG00116	-	L	Predicted N6-adenine-specific DNA methylase	**0**	**17**	**26**
arCOG00424	COG00608		L	DHH superfamily phosphohydrolase/exonuclease	**0**	**12**	**8**
arCOG04447	COG01933	-	L	Archaeal DNA polymerase II, large subunit	**0**	**0**	**78**
arCOG04455	COG01311	HYS2	L	Archaeal DNA polymerase II, small subunit/DNA polymerase delta, subunit B	**0**	**0**	**77**
arCOG00872	COG01111	MPH1	L	ERCC4-like helicase	**0**	**0**	**76**
arCOG00663	COG01208	GCD1	M	Nucleoside-diphosphate-sugar pyrophosphorylase involved in lipopolysaccharide biosynthesis/translation initiation factor 2B, gamma/epsilon subunit	**1**	**35**	**20**
arCOG02079	COG01361	-	M	S-layer domain	**1**	**5**	**55**
arCOG04064	COG00750	-	M	Predicted membrane-associated Zn-dependent protease	**1**	**35**	**78**
arCOG03199	COG00472	Rfe	M	UDP-N-acetylmuramyl pentapeptide phosphotransferase/UDP-N-acetylglucosamine-1-phosphate transferase	**1**	**35**	**39**
arCOG01817	COG00630	VirB11	N	Type IV secretory pathway, VirB11 component, or related ATPase involved in archaeal flagella biosynthesis	**1**	**35**	**64**
arCOG01814			N	Type II secretion system protein F	**0**	**0**	**19**
arCOG01818	COG00630	VirB11	N	Type IV secretory pathway, VirB11 component, or related ATPase involved in archaeal flagella biosynthesis	**0**	**7**	**25**
arCOG01308	COG00464	Cdc48	O	ATPase of the AAA+ class , CDC48 family	**4**	**35**	**78**
arCOG00970	COG00638	PRE1	O	20S proteasome, beta subunit	**1**	**35**	**78**
arCOG01257	COG00459	GroL	O	Chaperonin GroEL (HSP60 family)	**1**	**35**	**78**
arCOG00980	COG01047	SlpA	O	FKBP-type peptidyl-prolyl cis-trans isomerase 2	**1**	**35**	**78**

arCOG01341	COG01730	GIM5	O	Predicted prefoldin, molecular chaperone implicated in de novo protein folding	**1**	**35**	**78**
arCOG01342	COG01382	GimC	O	Prefoldin, chaperonin cofactor	**1**	**35**	**78**
arCOG04142	COG01899	DYS1	O	Deoxyhypusine synthase	**1**	**35**	**78**
arCOG00971	COG00638	PRE1	O	20S proteasome, alpha subunit	**1**	**35**	**77**
arCOG01296	COG00492	TrxB	O	Thioredoxin reductase	**1**	**35**	**74**
arCOG01218	COG00526	TrxA	O	Thiol-disulfide isomerase or thioredoxin	**1**	**35**	**32**
arCOG00312	COG00450	AhpC	O	Peroxiredoxin	**1**	**34**	**56**
arCOG01832	COG00071	IbpA	O	Molecular chaperone (HSP20 family)	**1**	**27**	**78**
arCOG02160	COG01067	LonB	O	Predicted ATP-dependent protease	**0**	**0**	**77**
arCOG04347	COG00500		Q	SAM-dependent methyltransferase	**0**	**3**	**49**
arCOG01937	COG01750	-	R	Archaeal serine protease	**0**	**25**	**33**
arCOG00557	COG01201	Lhr	R	Lhr-like helicase	**1**	**35**	**75**
arCOG01204	COG01708	-	R	Minimal nucleotidyltransferase	**1**	**25**	**11**
arCOG01951	COG01444	-	R	Predicted P-loop ATPase fused to an acetyltransferase	**1**	**35**	**36**
arCOG02155	COG01873	-	R	Protein implicated in RNA metabolism, contains PRC-barrel domain	**0**	**0**	**76**
arCOG00589	COG00455		R	CobQ/CobB/MinD/ParA ATPase	**0**	**8**	**60**
arCOG00187	COG01245	-	R	Predicted ATPase, RNase L inhibitor (RLI) homolog	**1**	**35**	**78**
arCOG00358	COG01163	DRG	R	Predicted GTPase	**1**	**35**	**78**
arCOG00501	COG01234	ElaC	R	Metal-dependent hydrolase of the beta-lactamase superfamily	**1**	**35**	**78**
arCOG00543	COG01782	-	R	Predicted metal-dependent RNase, consists of a metallo-beta-lactamase domain and an RNA-binding KH domain	**1**	**35**	**78**
arCOG04116	COG01855	-	R	ATPase (PilT family)	**1**	**35**	**78**
arCOG04150	COG01094	-	R	Predicted RNA-binding protein (contains KH domains)	**1**	**35**	**78**
arCOG04179	COG02118	-	R	DNA-binding protein	**1**	**35**	**78**
arCOG00035	COG02102	-	R	Predicted ATPase of PP-loop superfamily	**1**	**35**	**78**
arCOG00352	COG01084	-	R	Predicted GTPase	**1**	**35**	**78**
arCOG04312	COG01412	-	R	PIN domain containing protein	**1**	**35**	**77**
arCOG01150	COG01407	-	R	Predicted ICC-like phosphoesterase	**1**	**35**	**74**
arCOG04055	COG01371	-	R	SHS2 domain protein implicated in nucleic acid metabolism	**1**	**35**	**74**
arCOG01728	COG01355	-	R	Predicted dioxygenase	**1**	**35**	**63**
arCOG01845	COG02151	PaaD	R	Predicted metal-sulfur cluster biosynthetic enzyme	**1**	**35**	**56**
arCOG04219	COG02433	-	R	Possible nuclease of RNase H fold, RuvC/YqgF family	**1**	**35**	**53**
arCOG00969	COG02248	-	R	Predicted hydrolase (metallo-beta-lactamase superfamily)	**1**	**35**	**30**
arCOG04265	COG01779	-	R	C4-type Zn-finger protein	**1**	**34**	**62**
arCOG00324	COG00123	AcuC	R	Deacetylase, including yeast histone deacetylase and acetoin utilization protein	**1**	**34**	**51**
arCOG02044	COG01287	-	R	Uncharacterized membrane protein, required for N-linked glycosylation	**1**	**34**	**39**
arCOG00350	COG01161	-	R	Predicted GTPase	**1**	**34**	**33**
arCOG01145	COG02129	-	R	Icc family phosphoesterase	**1**	**33**	**65**
arCOG00017	COG02522	-	R	Predicted transcriptional regulator	**1**	**32**	**33**
arCOG02960	COG04882	-	R	Predicted aminopeptidase, Iap family	**1**	**32**	**0**
arCOG04702	COG01881	-	R	Phospholipid-binding protein	**1**	**25**	**46**
arCOG00769	COG00693	ThiJ	R	Putative intracellular protease/amidase	**1**	**24**	**69**
arCOG02209	COG02244	RfbX	R	Polysaccharide biosynthesis protein, Mvin family	**0**	**21**	**76**
arCOG02900	COG02304	-	R	Uncharacterized protein containing a von Willebrand factor type A (vWA) domain	**1**	**19**	**21**
arCOG00601	COG00517	-	R	Protein containing two CBS domains (some fused to C-terminal double-stranded RNA-binding domain of RaiA family)	**1**	**18**	**57**
arCOG01744	COG01988	-	R	Membrane-bound metal-dependent hydrolase	**0**	**9**	**29**
arCOG01688	COG01719	-	R	Predicted transcriptional regulator, contains HTH and 4VR domain	**0**	**7**	**40**
arCOG00438	COG01239		R	AAA family ATPase	**0**	**5**	**50**
arCOG02261			R	Protein containing OB-fold-like and PCI domains	**0**	**0**	**16**
arCOG01159	COG01340	-	S	Uncharacterized archaeal coiled-coil protein	**1**	**35**	**66**
arCOG01572			S	Uncharacterized conserved membrane protein	**1**	**28**	**17**
arCOG04214	COG00432	-	S	Uncharacterized conserved protein	**1**	**35**	**65**
arCOG00398	COG02411	-	S	ASCH domain, predicted RNA-binding domain	**0**	**20**	**24**
arCOG01994	COG01300	SpoIIM	S	Uncharacterized membrane protein	**0**	**6**	**64**
arCOG06946			S	Uncharacterized conserved membrane protein	**0**	**3**	**0**
arCOG05037			S	Uncharacterized conserved protein	**0**	**0**	**27**
arCOG01180	COG01718	RIO1	T	Serine/threonine protein kinase involved in cell cycle control	**1**	**35**	**78**
arCOG01185	COG03642	-	T	Mn2+-dependent serine/threonine protein kinase	**1**	**35**	**78**
arCOG01182	COG02112	-	T	Predicted Ser/Thr protein kinase	**1**	**34**	**31**
arCOG01171	COG00467	RAD55	T	RecA-superfamily ATPase implicated in signal transduction	**1**	**33**	**58**
arCOG04471	COG04083	-	U	Exosortase	**0**	**2**	**55**
arCOG04169	COG00201	SecY	U	Preprotein translocase subunit SecY	**1**	**35**	**78**
arCOG02204	COG02443	Sss1	U	Preprotein translocase subunit Sss1	**1**	**35**	**78**
arCOG01739	COG00681	LepB	U	Signal peptidase I	**1**	**33**	**77**
arCOG03054	COG00341	SecF	U	Preprotein translocase subunit SecF	**0**	**0**	**68**
arCOG03055	COG00342	SecD	U	Preprotein translocase subunit SecD	**0**	**0**	**68**
arCOG00793	COG01468	-	V	CRISPR-associated protein, Cas4 family, PD-(D/E)XK superfamily nuclease	**0**	**0**	**28**
